# Enantioselective Synthesis of Axially Chiral Spiro[3.3]heptanes by Site-Selective C–H Functionalization

**DOI:** 10.1021/acscatal.6c00560

**Published:** 2026-03-04

**Authors:** Duc Ly, Ziyi Chen, Djamaladdin G. Musaev, Huw M. L. Davies

**Affiliations:** † Department of Chemistry, 1371 Emory University, 1515 Dickey Drive, Atlanta, Georgia 30322, United States; ‡ Cherry L. Emerson Center for Scientific Computation, Emory University, Atlanta, Georgia 30322, United States

**Keywords:** spiro[3.3]heptane, axially chiral, chiral spiranes, rhodium carbene, C−H functionalization, desymmetrization, asymmetric catalysis

## Abstract

The enantioselective synthesis of axially chiral 2,6-disubstituted spiro[3.3]­heptanes is challenging because the differentiating functionalities are far apart from each other. The known enantioselective methods to generate these compounds have relied on the use of enzymatic processes. The current study achieves a highly regio-, diastereo-, and enantioselective entry to the 2,6-disubstituted spiro[3.3]­heptanes by desymmetrizing 2-substituted spiro[3.3]­heptanes using rhodium-catalyzed C–H functionalization by donor/acceptor carbenes derived from aryldiazoacetates and styryldiazoacetates. The optimum catalyst is dirhodium tetrakis­(4,4′-(3,5-ditertbutylphenyl)-6,6′-dichlorobinaphthylphosphate) (Rh_2_(*S*-MegaBNP)_4_), which adopts a D_4_-symmetric structure. The optimum functionality on the spiro[3.3]­heptane is the *N*-phthalimido group, which is ideally suited for further derivatization to a range of amine and amide derivatives. Under the optimized conditions, the C–H functionalization products can be generated in up to 92% yield, >20:1 rr, >20:1 dr, and 99% ee. Computational studies revealed that the catalyst is relatively rigid and both the orientation of the bound carbene and the approaching substrate are controlled by their necessary alignment in hydrophobic grooves between *tert*-butyl groups of adjacent ligands. The diastereoselectivity is controlled by selective C–H functionalization of one of the equilibrating enantiomers of the 2-substituted spiro[3.3]­heptane, hence achieving conformation sorting. These studies reveal that bowl-shaped dirhodium catalysts are capable of subtle site selectivity caused by secondary noncovalent interactions with the catalyst wall.

## Graphical abstract

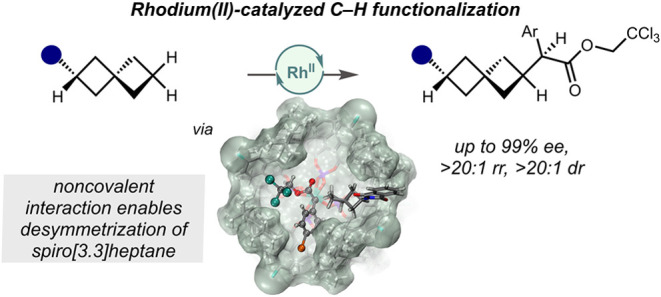

## Introduction

2,6-Disubstituted spiro[3.3]­heptane (**1**) is an interesting scaffold because when appropriately substituted it is axially chiral,^1^ although it is less developed compared with related systems with axial chirality, such as biaryl **2**,^2^ allene **3**,^3,4^ and cyclohexylidene **4** ^5–7^ (Figure 1A). It has gained further interest because of its potential application in medicinal chemistry as a bioisostere for benzene.^1,8–12^ Two recent examples incorporating this scaffold into drug candidates are the NAMPT modulator **5** ^13^ and indoleamine 2,3-dioxygenase inhibitor **6** ^14^ (Figure 1B). The first example of an axially chiral spiro[3.3]­heptane was reported in 1907,^15^ and its absolute configuration was unambiguously determined by X-ray crystallography almost 70 years later.^16^ Despite the early discovery, the difficulty in the asymmetric synthesis of axially chiral spiro[3.3]­heptanes has limited their broad utility. Their synthesis typically relies on chiral separation,^13,14,17,18^ as can be seen with the drug targets **5** and **6**, which were prepared as racemates and then resolved by chiral chromatography.^13,14^ The distal location of the functionality in 2,6-disubstituted spiro[3.3]­heptanes means that many of the regular transition metal-catalyzed asymmetric methods are not suitable, and so, the available methods rely on enzymatic processes (Figure 1C). The first asymmetric synthesis of axially chiral disubstituted spiro[3.3]­heptanes used pig-liver esterase to catalyze the hydrolysis of racemic or meso esters **7**, but relatively low levels of enantioselectivity were obtained (40% ee).^19,20^ Recently, an asymmetric reduction of a prochiral ketone **8** catalyzed by a ketoreductase afforded either enantiomer of axially chiral spiro[3.3]­heptanes with high levels of asymmetric induction.^21^ An intriguing strategy to access the 2,6-disubstituted spiro[3.3]­heptane would be by means of the C–H functionalization of 2-substituted spiro[3.3]­heptanes. Recently, an enzymatic C–H hydroxylation of a single substrate (**9**) was reported.^22^ Even though the reaction proceeded with high levels of asymmetric induction, competing C5 hydroxylation (**11**) was prevalent, resulting in the formation of a mixture of products (**10** and **11**) with low site selectivity (3:1 rr). Considering that the only reported catalytic methods for the enantioselective synthesis of spiro[3.3]­heptanes have relied on enzymatic processes, operating with a narrow range of substrates, the development of an effective nonenzymatic C–H functionalization process would be highly desirable.

**Figure 1:**
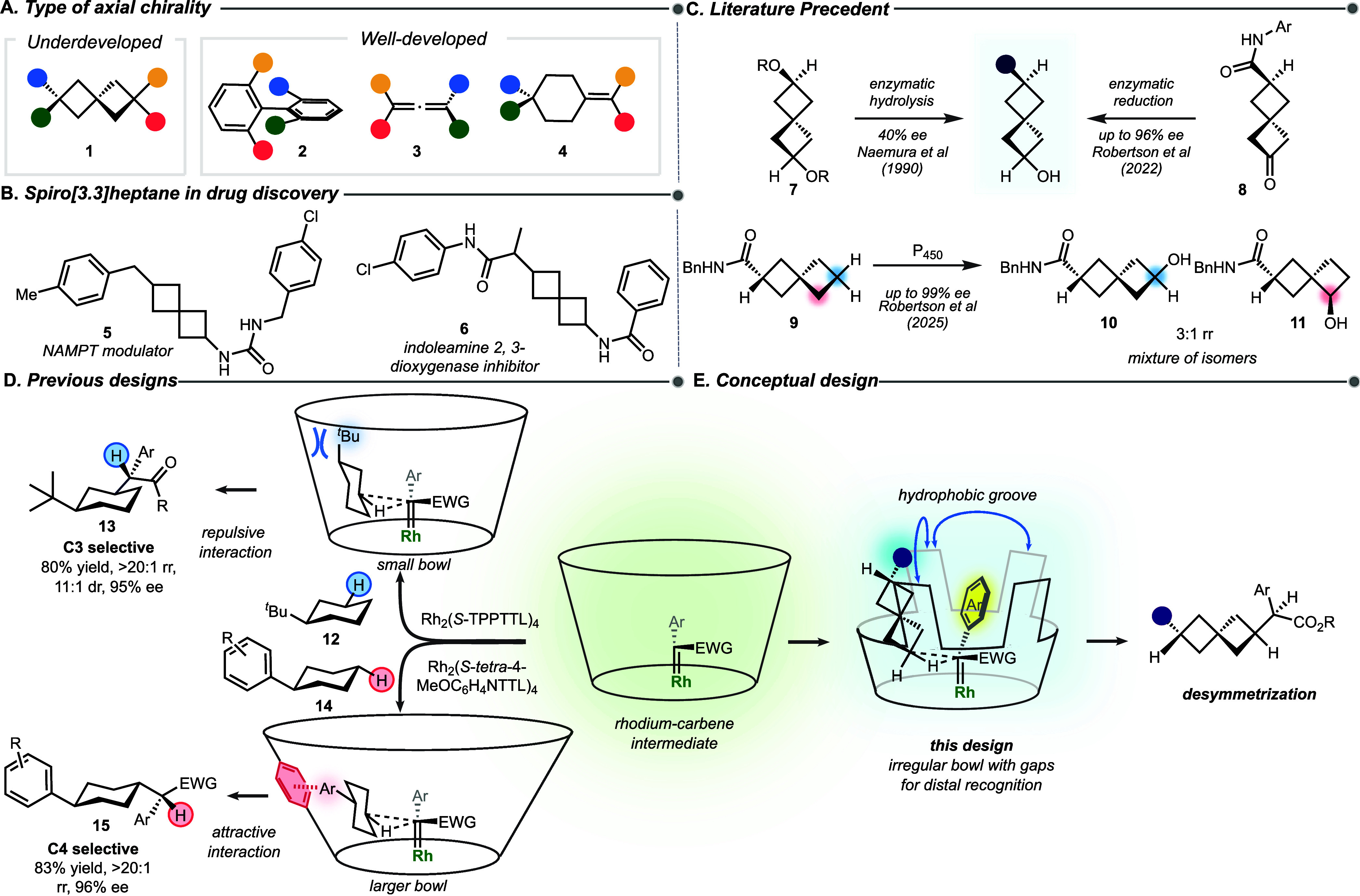
Background and current work. (A) Type of axial chirality. (B) The use of spiro[3.3]­heptane in medicinal chemistry. (C) State-of-the-art approaches for the synthesis of axially chiral spiro[3.3]­heptane. (D) Previous designs in site-selective C–H functionalization. (E) Current design using an irregular bowl-shaped catalyst with a hydrophobic groove for subtle selectivity.

Site-selective and enantioselective C–H functionalization is a major challenge in organic synthesis.^23,24^ The reagents need to be reactive enough to react with relatively inert C–H bonds, but they also need to distinguish between many similar sites.^24^ We have shown that rhodium-stabilized donor–acceptor carbenes exhibit exceptional selectivity in C–H functionalization reactions.^25^ The early examples focused primarily on reactions of C–H bonds at activated positions, such as allylic, benzylic, or α to oxygen or nitrogen.^24^ Later, the work progressed to catalyst-controlled reaction at the most accessible unactivated 3°, 2°, or 1° C–H bonds by alternating the steric environment of the catalyst.^25^ Our latest strategy uses high symmetry bowl-shaped dirhodium catalysts in which the site selectivity is controlled by distal noncovalent secondary interactions between the approaching substrate and the bowl wall (Figure 1D). The proof-of-concept experiments to demonstrate the feasibility of this approach involved site-selective functionalization of substituted cyclohexanes, in which either C3 or C4 functionalization can occur on account of steric interference^26^ or positive nonbonding interactions^27^ with the catalyst wall. The Rh_2_(*S*-TPPTTL)_4_-catalyzed reaction of *tert-*butylcyclohexane **12** resulted in a selective functionalization at C3 to form **13**.^26,28^ The steric interference between the substituent on **12** and the catalyst guides the substrate to react at the C3 position. In contrast, the Rh_2_(*S*-*tetra*-MeOC_6_H_4_NTTL)_4_-catalyzed reaction of arylcyclohexane **14** resulted in clean formation of the C4-substituted product **15**, taking advantage of favorable π/π and C–H/π interactions between the approaching arylcyclohexane and the catalyst wall.^27^ In this paper, we describe a conceptually new approach to control site selectivity using a D_4_-symmetric catalyst with four well-defined grooves in the catalyst bowl (Figure 1E). In this system, the aryl group of the carbene is locked into one of the grooves, and the spiro[3.3]­heptane substrate approaches the carbene in an adjacent groove, resulting in a highly diastereoselective and enantioselective reaction even though the differentiating substituent on the spiro[3.3]­heptane is far removed from the site of C–H functionalization.

## Results and Discussion

### Reaction Optimization

On the basis of these background studies, we decided to commence our study by examining the C–H functionalization of 2-aryl spiro[3.3]­heptanes **16** with the aryldiazoacetate **17**. The initial evaluation was carried out using Rh_2_(*S*-TPPTTL)_4_ and Rh_2_(*S*-*tetra*-MeOC_6_H_4_NTTL)_4_ (Figure 2, entries 1 and 2). Unfortunately, the reaction was not selective, resulting in the formation of a mixture of products due to competitive C–H functionalization at the benzylic site (**18**) as well as the distal C6 methylene site (**19**). Furthermore, the reaction at C6 was not diastereoselective, resulting in a 1:1 mixture of diastereomers. In the case of the arylcyclohexane derivative **14**, the benzylic C–H bond is located at a sterically unfavored axial position and Rh_2_(*S*-*tetra*-MeOC_6_H_4_NTTL)_4_-catalyzed reaction gave trace benzylic C–H functionalization. Presumably, the steric demand for the electronically favored benzylic position in the spiro[3.3]­heptane system (**16**) is not as pronounced as that with arylcyclohexane **14**. Previously, we have shown that it is possible to avoid attack at the electronically favored positions by using a bulky catalyst such as Rh_2_(*S*-2Cl5BrTPCP)_4_.^29^ Although the catalyst was able to favor reaction at the desired distal methylene site at C6 (**19**), the diastereoselectivity of the reaction is low, indicating insufficient catalyst recognition for the two hydrogens at the distal methylene position in 2-aryl spiro[3.3]­heptane **16** (Figure 2, entry 3).

**Figure 2:**
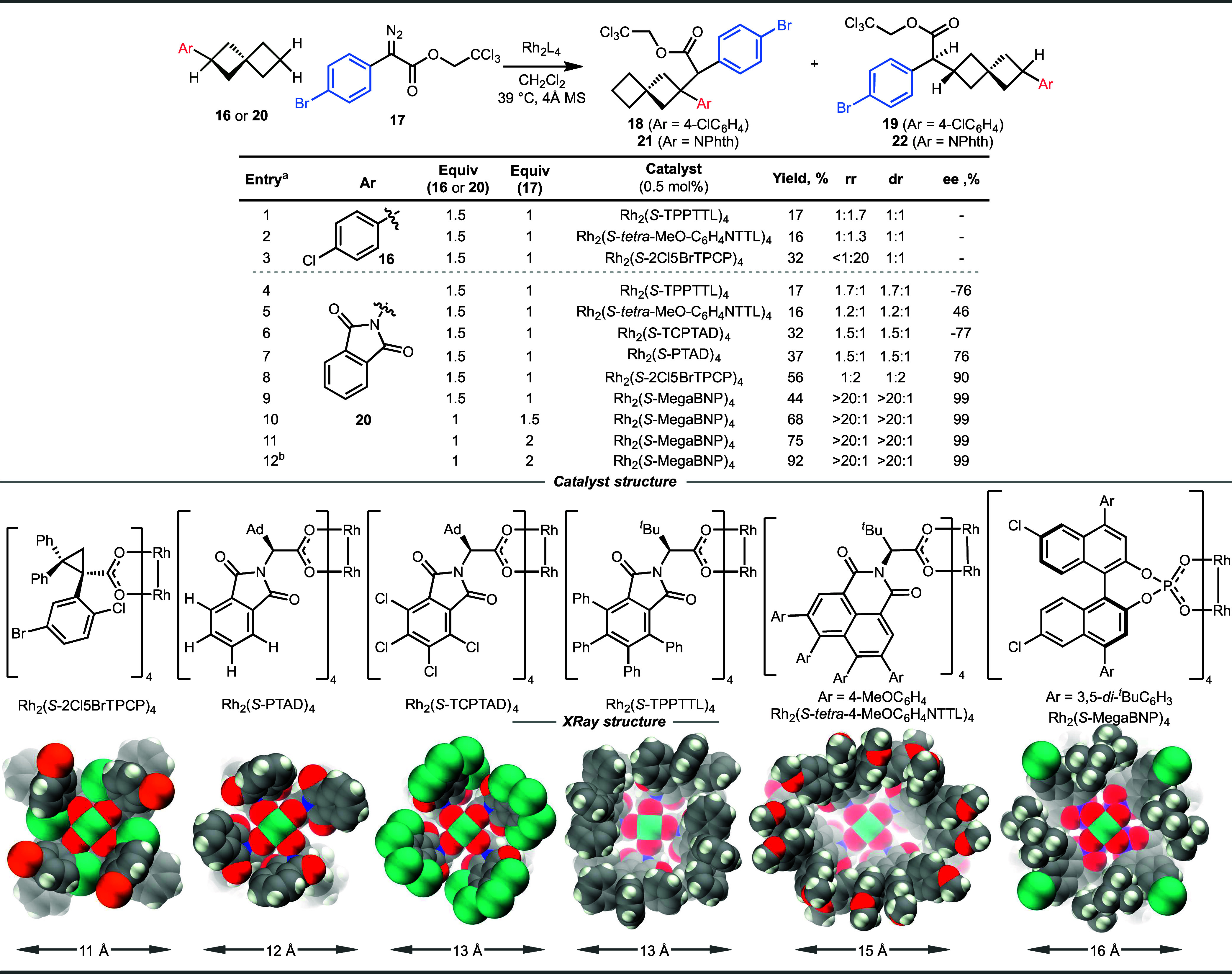
Catalyst optimization. ^a^Reaction conditions: 16 or 20, 17, 4 Å MS, and Rh_2_L_4_ (0.5 mol %) in CH_2_Cl_2_ at 39 °C. Yields are isolated yields. Regioisomeric (rr) and diastereomeric (dr) ratios were determined by ^1^H NMR. The regioisomeric ratio (rr) is >20:1 in all cases. ^b^1,1,1,3,3,3-Hexafluoro-2-propanol (HFIP) (0.25 equiv) was added. The structures of rhodium catalysts are reproduced from refs 27,41 . Copyright 2026 American Chemical Society.

Having determined that an aryl substituent would not work as a suitable group to influence the diastereoselectivity at the distal position of the spiro[3.3]­heptane, an alternative group that can constrain the conformational flexibility was explored. It was hypothesized that the substituent would need to have an aryl functionality for effective interactions with the catalyst wall, but would need to avoid activating C–H bonds adjacent to it. Hence, we decided to explore the reaction with the *N*-phthalimido group because it has been shown to block functionalization of C–H bonds at the *α* position.^30^ Furthermore, the *N*-phthalimido group would offer synthetic versatility, as it can be readily converted to the free amine. The optimization studies on C–H functionalization with 2-*N*-phthalimido spiro­[3.3]­heptane **20** as the substrate and 2,2,2-trichloroethyl 2-(4-bromophenyl)-2-diazoacetate **17** as the carbene source are shown in Figure 2. Initially, we explored a series of dirhodium tetracarboxylate catalysts. Our newly developed bowl-shaped catalysts, Rh_2_(*S*-TPPTTL)_4_ and Rh_2_(*S*-*tetra*-MeOC_6_H_4_NTTL)_4_, did result in distal C–H functionalization (**22**), but the yields were low (16–17%) with no observation of other regioisomers (Figure 2, entries 4 and 5). The asymmetric induction at the carbene site was moderately high, but the diastereoselectivity was low (<2:1 d.r.), indicating that a combination of the distal *N*-phthalimido substituent and regular bowl-shaped dirhodium tetracarboxylate catalysts had limited effect to desymmetrize **20**. A sampling of some of our other chiral dirhodium tetracarboxylate catalysts with different shapes was conducted. Although they resulted in better yields and reasonably high levels of enantioselectivity (76–90% ee), the diastereoselectivity with all of these catalysts was poor (<2:1 d.r.) (Figure 2, entries 6–8). The low yield and poor diastereoselectivity indicate that the substrate is failing to fit effectively and in a defined way into the catalyst pocket, suggesting that a different type of dirhodium catalyst is needed.

A range of bowl-shaped catalysts have been generated by us^26,27,31–34^ and others^35–40^ (Figure 2), many of which have been impactful in the enantioselective reactions with donor/acceptor carbenes. The size of the bowl varies from 11 to 15 Å depending on the specific carboxylate ligands, and this greatly influences the site selectivity exhibited by these catalysts. Having limited success with the dirhodium tetracarboxylate catalysts, we switched to a recently reported binaphthylphosphonate-derived catalyst, Rh_2_(*S*-MegaBNP)_4_.^34^ This catalyst has a larger bowl structure than the dirhodium tetracarboxylates and is an exceptional catalyst for the reactions of donor/acceptor carbenes, being the best catalyst to date for selective C–H functionalization of unactivated tertiary C–H bonds^34^ and bicyclohexanes.^41^ When Rh_2_(*S*-MegaBNP)_4_ was applied to the spiro[3.3]­heptane challenge, we were delighted to observe that it performed extremely well in this system. Under the test conditions, **22** was formed in 44% yield with excellent control of diastereoselectivity (>20:1 dr) and enantioselectivity (99% ee) (Figure 2, entry 9) with no trace of regioisomers. Further optimization by changing the stoichiometry resulted in considerable improvement in the yield (Figure 2, entries 10 and 11). Particularly, when the spiro[3.3]­heptane **20** is the limiting agent and 2 equiv of aryldiazoacetate **17** was used, **22** is formed in 75% yield (entry 11). Previously, we have shown that adding small quantities of 1,1,1,3,3,3-hexafluoro-2-propanol (HFIP) can enhance the C–H functionalization reaction^42,43^ and when HFIP (0.25 equiv) was added, the yield of **22** improved to 92%, presumably by helping the catalyst last longer (Figure 2, entry 12). It is also worth noting that the addition of 4 Å molecule sieves (4 Å MS) in this system helps the reaction to be more robust by preventing the interference of adventitious moisture.^44^

### Reaction Scope

We then studied the scope of the current desymmetrization C–H functionalization using the optimized conditions (Figure 3A). Generally, the reaction was applicable to a wide range of aryldiazoacetates, proceeding with high levels of stereoselectivity. For example, para-iodo derivative **23** was produced in 93% yield, 29:1 dr, and 99% ee. The reaction also performed extremely well with electron-withdrawing groups, such as −CF_3_, −CO_2_Me, −NO_2_, −BPin, −OTf, and −Ph (**24**-**29**). A diazo-bearing heterocycle also performed very well under slightly modified optimized conditions to give the pyridine derivative **30** in good yield and selectivity. Moving the para-substituent to the meta-position significantly affected the stereoselectivity of the reaction, as the meta-Br (**31**) and meta-Me (**32**) products are formed only in 5:1 dr and 7:1 dr but good asymmetric induction with 88% and 97% ee, respectively. The reaction completely lost the distal selectivity if the substituent is at the ortho-position (**33**), resulting in a 1:1 dr and 72% ee. This effect of meta- and ortho-substituents was observed before for Rh_2_(MegaBNP)_4_ in C–H functionalization with cyclohexane and the para-substituent is important to obtain a high level of enantioselectivity.^34^ Indeed, installing the para-substituent to meta- or ortho-substituted aryldiazoacetates fully or partially recovered the stereoselectivity as observed with **34** (21:1 dr, 98% ee) and **35** (4:1 dr, 85% ee). The effect of para-substituents on diastereoselectivity indicates that they are important to sustain a well-defined catalyst pocket.

**Figure 3:**
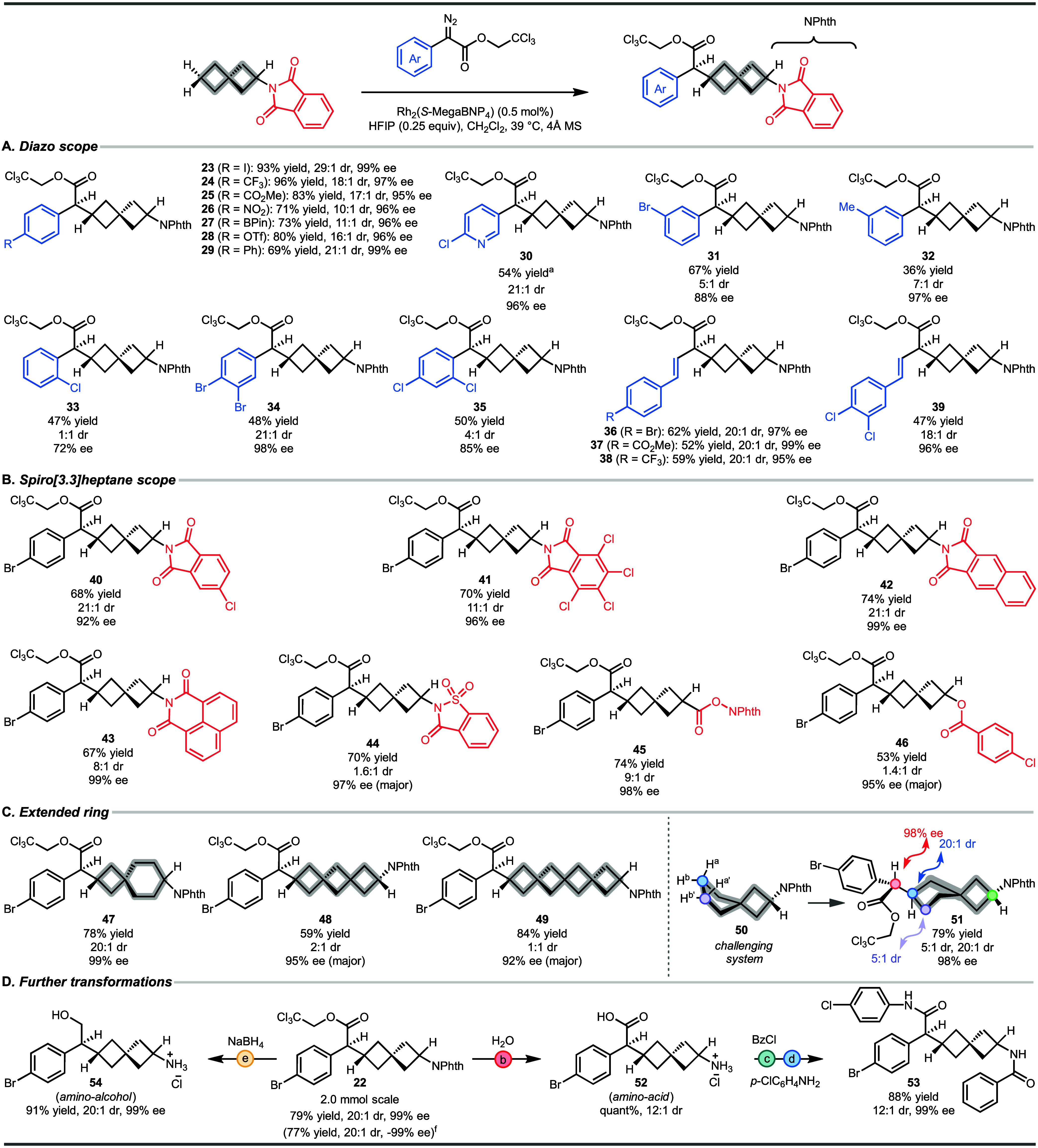
Reaction scope and further transformations. Reaction conditions: substrate (0.2 mmol), diazo (2.0 equiv), 4 Å MS, HFIP (0.25 equiv), and Rh_2_(*S*-MegaBNP)_4_ (0.5 mol %) in CH_2_Cl_2_ at 39 °C. (a) Diazo (1.0 equiv) and substrate (1.5 equiv); (b) acetic acid, HCl (5N, aq), reflux; (c) BzCl (2 equiv), Na_2_CO_3_ (4 equiv), THF/H_2_O (1:1); (d) *p*-ClC_6_H_4_NH_2_ (1.5 equiv), EDCI (1.7 equiv), DMAP (2.0 equiv), HOBt (1.2 equiv) in CH_2_Cl_2_; (e) NaBH_4_ (6 equiv), *i*-PrOH/H_2_O/THF (6:1:1, 2 mL), then HCl (4M, dioxane). (f) Rh_2_(*R*-MegaBNP)_4_ was used.

Styryldiazoacetates are another important class of donor–acceptor carbene precursors, but studies on their regio- and stereoselectivity in C–H functionalization of unactivated C–H bonds are limited.^45^ One of the most challenging aspects of vinyldiazoacetates is their tendency to isomerize to pyrazoles which can then poison the catalyst.^46^ Therefore, the catalysts need to be active, generating carbene before undesirable pyrazole formation can occur. We were pleased to see that Rh_2_(*S*-MegaBNP)_4_ performed well with styryldiazoacetates to give **36**–**39** in good yields and high levels of stereoselectivity (20:1 dr, >95% ee), albeit lower yields than the model study. We were able to unambiguously determine the relative and absolute stereocenters of **37** via single-crystal X-ray determination. The absolute configuration of the other products is tentatively assigned by analogy. The reaction is not compatible with donating groups at the para-position with either the aryldiazoacetates or the styryldiazoacetates. With these carbene precursors, we observed significant carbene dimerization and only a trace of the desired C–H functionalization products. Presumably, these carbenes are not sufficiently electrophilic to undergo effective C–H functionalization with **20**.

The *N*-phthalimido group is assumed to play a significant role in controlling the selectivity of the reaction, and in order to confirm that this is indeed the case, we studied related functionality to the *N*-phthalimido group (Figure 3B). The monochloro *N*-phthalimide (**40**) performed smoothly under the optimized conditions, retaining a high level of stereoselectivity (21:1 dr, 92% ee), but the tetrachloro derivative (**41**) resulted in a drop in the diastereoselectivity to 11:1 dr, implying the steric environment around the *N*-phthalimido group influences the selectivity of the reaction. The extended *N*-naphthalimide was also effective, but the linear *N*-naphthalimide gave higher diastereoselectivity (**42**, 21:1 dr, 99% ee) than the 1,8-substituted *N*-naphthalimide (**43**, 8:1 dr, 99% ee). When the *N*-phthalimido group was moved two atoms further away, it was still reasonably effective, generating **45** in 74% yield, 9:1 dr, and 98% ee. In contrast, changing the *N*-phthalimido group to a saccharine derivative resulted in the formation of **44** in only 1.5:1 dr but with 97% ee. The diastereoselectivity was also seriously compromised when an aryl ester group was used, leading to the formation of **46** with no diastereocontrol in 1.4:1 dr. These studies support the concept that the *N*-phthalimido group plays a highly significant role in controlling the diastereoselectivity of the reaction. Groups similar to the *N*-phthalimido group also give good diastereocontrol, but other types of groups give very low diastereoselectivity. The diastereoselectivity is high throughout except when the aryl group of the carbene has ortho or meta substituents and is lacking a para-substituent, while the enantioselectivity is generally high throughout the study.

Highly stereoselective reactions can also be obtained with other spirocyclic systems (Figure 3C). The reaction to form the spiro[3.5]­nonane (**47**) is extremely effective, resulting in high levels of enantioselectivity (98% ee) and diastereoselectivity (20:1 dr). These results intrigued us to explore whether *N*-phthalimide can still be recognized by the catalyst pocket when it is further away. Therefore, we decided to extend the spiro[3.3]­heptane to more elaborate systems, such as dispiro­[3.1.3^6.14^]­decane (**48**) and trispiro­[3.1.1.3^8.16.14^]­tridecane (**49**). The C–H functionalization was still effectively achieved at the distal methylene site (>20:1 rr, >90% ee), but no control of diastereoselectivity was observed (1:1 dr). The poor diastereoselectivity in the formation of **48** and **49** suggests that the *N*-phthalimido group is now too far away to cause significant influence through interactions with the catalyst wall.

Building on the successful desymmetrization of spiro[3.3]­heptane (**20**) and spiro[3.5]­nonane (**47**), we decided to explore an even more challenging system, spiro[3.4]­octane (**50**) (Figure 3C). During the formation of **51**, three sets of diastereomers could be generated in which an additional diastereomer is generated due to the asymmetry introduced in the cyclopentane ring of the product. Despite the complexity, the reaction proceeded smoothly, affording product **51** in 79% yield and 98% ee with only two observable diastereomers in a ratio of 5:1 dr. Asymmetric induction at the carbene center (red) is governed by the chiral catalyst, and as has been typically observed in this current study, the enantioselectivity is very high. The diastereoselectivity at the C–H functionalization site, which represents desymmetrization of the cyclopentane, depends on the selective activation of H^a^ versus H^b^ (or H^a′^ versus H^b′^). Detailed NMR analysis indicated that the desymmetrization was highly selective (>20:1 dr), favoring reaction at H^a^ and H^b′^. The diastereoselectivity in relationship to the *N*-phthalimido group (green) is controlled by selective C–H functionalization at H^a^ (blue) versus H^b′^ (purple), and reaction at H^a^ is favored by a factor of 5:1. The decreased influence of the *N*-phthalimido group on the diastereoselectivity at the second site compared with the spiro[3.3]­heptanes is presumably caused by the greater conformational mobility of a cyclopentane ring compared with a cyclobutane ring.

The reaction can easily be scaled up to a 2 mmol scale with a similar yield and stereoselectivity. The opposite enantiomer can easily be accessed by simply using the (*R*)-enantiomer of Rh_2_(MegaBNP)_4_ (Figure 3D). The *N*-phthalimido and trichloroethyl groups in the product can be easily removed by hydrolysis under acidic conditions to give chiral 1,6-amino acid **52**, but the product experienced slight epimerization under the reaction conditions (from 20:1 dr to 12:1 dr). 1,6-Amino acid **52** could be a novel linker for medicinal chemistry. For example, compound **53**—a derivative of the indoleamine 2,3-dioxygenase inhibitor **6**—can be easily prepared from **52** via 2 consecutive amide coupling reactions. Additionally, **22** can be transformed into a chiral 1,6-aminoalcohol **54** under reductive conditions, and this reaction proceeded with no epimerization.

### Computational Studies

The selective C–H functionalization of 2-substituted spiro[3.3]­heptane **20** described herein is intriguing because the controlling element is remote from the reactive center in the substrate. Therefore, the calculations for this study focused on how the spiro[3.3]­heptane substrate would react with the rhodium-bound carbene intermediate. We first analyzed the preferred conformations of spiro[3.3]­heptane **20** (Figure 4A). The *N*-phthalimido group preferentially occupies the pseudoequatorial position (**20a** or **20b**) over the pseudoaxial position **20c** by 3.0 kcal/mol. Also, the *N*-phthalimido in **20a** (or **20b**) aligns in an eclipsed orientation to the first cyclobutane ring, likely stabilized by a favorable CO/CH interaction (see Figure S13). Ring flipping (inversion) of the second cyclobutane ring generates enantiomeric conformers **20a** and **20b**, which interconvert rapidly due to a low energy barrier of 1.5 kcal/mol. Given the predominant conformation, we propose that C–H functionalization would occur on **20a** (or **20b**) rather than **20c**. Previous studies on the C–H functionalization of cyclohexanes and cyclobutanes with aryldiazoacetates have shown that the (pseudo)­equatorial C–H bonds are approximately 140 times more reactive than the (pseudo)­axial ones.^26,29^ As a result, we propose that only the purple pseudoequatorial C–H bond in **20a** (or **20b**) will be involved in the C–H insertion reaction. Consequently, the diastereoselectivity of the C–H functionalization would depend on dynamic kinetic resolution, favoring the reaction of the carbene to one of the enantiomeric conformers over the other.

**Figure 4:**
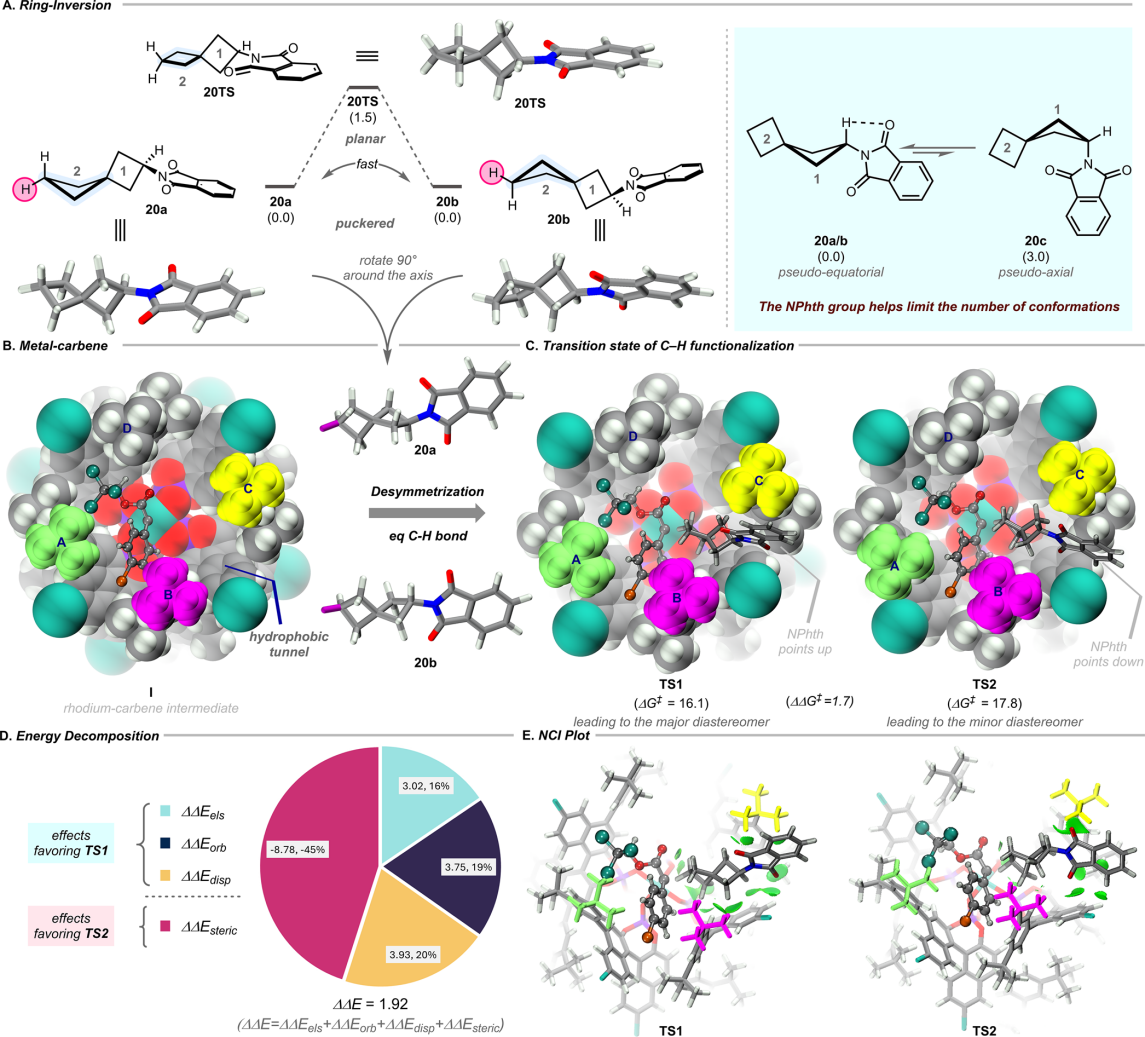
Computational study. (A) Conformational analysis of substrate **20** at the M06/6–31G­(d,p) level with the relative Gibbs free energies in kcal/mol. (B) Optimized structure of rhodium–carbene complex I with the hydrophobic groove is highlighted between two ^*t*^Bu groups (purple (B) and yellow (C)). (C) Transition states **TS1** and **TS2** of the desymmetrization process, leading to the major and minor diastereomers, respectively; reported energies are the Gibbs free energies in kcal/mol at the ONIOM (M06/[Lanl2dz + 6–31G­(d,p)]:UFF) level. (D) Energy decomposition analysis of **TS2** and **TS1** with relative energies for electronic energy (*Δ*Δ*E*), and electrostatic (*Δ*Δ*E*_els_), orbital (*Δ*Δ*E*_orb_), dispersive (*Δ*Δ*E*_disp_), and steric (*Δ*Δ*E*_steric_) interactions: all reported energies are relative energies of **TS2** to **TS1** and in kcal/mol. (E) Noncovalent interaction (NCI) map via IGMH method,^56^ the green surface represents noncovalent interaction (0.007 au), using the Multiwfn program.^57^ All of the figures were rendered by the VMD program.^58^ The structure of rhodium–carbene complex I is reproduced from ref 41. Copyright 2026 American Chemical Society.

Having established the key structural features of the substrate, we then investigated its interaction with the rhodium carbene complex. Due to the large size of the Rh_2_(*S*-MegaBNP)_4_, we employed a 2-layer ONIOM (QM:MM) approach^47–49^ in which 16 *t*-butyl groups of Rh_2_(*S*-MegaBNP)_4_ are included in the second layer and treated at molecular mechanic (UFF^50^) level, while the rest of the system (including carbene, substrate, and catalyst) used as a model system (i.e., the first layer) and treated by the density functional (M06)^51^ method (see Figure S8). Detailed information about the structure of the Rh_2_(*S*-MegaBNP)_4_ catalyst and its carbene-bound complex has been previously obtained from a combination of X-ray crystallographic and computational studies.^34,41^ These studies showed that Rh_2_(*S*-MegaBNP)_4_ exhibits a D_4_-symmetric architecture, with four of the 16 *tert*-butyl (^*t*^Bu) groups forming hydrophobic grooves on each face of the dirhodium complex (Figure 2).^34^ Owing to this high symmetry, its rhodium–carbene complex adopts the energetically most favorable isomer **I** (Figure 4B), where the bowl-shaped framework of the catalyst remains largely unaltered. In intermediate **I**, the aryl and trichloroethyl substituents of the carbene are confined within two hydrophobic grooves formed between the ^*t*^Bu groups (^*t*^Bu-A, B, and D).^41^ Notably, the aryl group positioned between the green (A) and purple (B) ^*t*^Bu groups is tilted significantly out of the rhodium–carbene plane with a dihedral angle of 16° (see Figure S14). This contrasts with the typical close-to-0° angle observed in rhodium–carbene complexes derived from tetracarboxylate-based dirhodium catalysts.^40,52–54^ This favorable tilting of the aryl group to one side results in an open pocket on the opposite side of the catalyst, which presumably accounts for the high enantioselectivity observed in the C–H functionalization reactions.

With two corners blocked by the carbene fragment, as described above, substrate **20** can approach the highly electrophilic carbene center only from the remaining two sites. Because of the well-defined trajectory of the C–H insertion step,^52,55^ this approach occurs specifically through the hydrophobic groove formed by the purple (B) and yellow (C) ^*t*^Bu groups (Figure 4B,C). Examining the approach of the two predominant conformers of **20**, we were able to locate the two corresponding transition states (**TS1** and **TS2**), each accommodating the different enantiomers **20a** and **20b**, respectively. The desymmetrization process requires the catalyst to distinguish subtle conformational differences within a chiral environment. Indeed, the computational studies predict **TS1**, which leads to the major diastereomer, to be 1.7 kcal/mol lower in free energy than **TS2**, consistent with the experimentally observed diastereomeric ratio (20:1 dr).

To shed light on factors contributing to the energy difference between **TS1** and **TS2**, the energy decomposition analysis^59,60^ was performed. This analysis breaks down the 1.9 kcal/mol electronic energy difference (*Δ*Δ*E*) between **TS2** and **TS1** into electrostatic (*Δ*Δ*E*_els_), orbital (*Δ*Δ*E*_orb_), dispersive (*Δ*Δ*E*_disp_), and steric (*Δ*Δ*E*_steric_) interaction terms (Figure 4D and see the Supporting Information). The results show that **TS1** is favored by electrostatic, orbital, and dispersive interactions, whereas **TS2** benefits from reduced steric repulsion. However, the combined stabilizing effects of the electrostatic, orbital, and dispersive components outweigh the steric penalty, making **TS1** the overall lower-energy pathway. The favorable orbital interaction in **TS1** likely arises from the stabilizing secondary interaction between substrate and catalyst environment, facilitating better orbital overlap between the σ­(C–H) bond and the π* orbital of the rhodium carbene (see Figure S11). Dispersive interactions between the substrate and the catalyst pocket are also more pronounced in **TS1**, as visualized by the IGMH map^56,57^ (Figure 4E), which highlights stronger noncovalent contacts—including CH/π, CH/CO, and CH/CH— compared with **TS2**. This computational study highlights the importance of the *N*-phthalimido group, which not only helps limit the conformation of the substrate but also forms a favorable interaction with the catalyst pocket via the aromatic system and the carbonyl group.

## Conclusion

We have demonstrated that donor–acceptor carbenes, in combination with dirhodium catalysts, can effectively desymmetrize spiro[3.3]­heptane derivatives, a transformation previously restricted to enzymatic catalysis. The optimal functionality on the spiro[3.3]­heptane is the *N*-phthalimido group, which is ideally suited for further derivatization to a range of amine and amide derivatives. The Rh_2_(*S*-MegaBNP)_4_-catalyzed C–H functionalization exhibits exceptional site-, diastereo-, and enantioselectivity, underscoring the unique capabilities of bowl-shaped dirhodium catalysts, especially where enzymes remain limited in site selectivity. This methodology is extended to other spirocyclic systems with similarly high selectivity. Due to the D_4_-symmetric nature of the catalyst, there is a single preferred orientation of the bound carbene. Computational analysis reveals that the catalyst achieves a dynamic kinetic resolution (conformation sorting) of two enantiomeric conformers via a combination of electronic, orbital, and dispersive interactions within its hydrophobic pocket. The catalyst wall locks the carbene in a specific position and guides substrate orientation to distinguish subtle conformational differences, enabling selective recognition. This mechanistic insight provides a foundation for expanding dirhodium-catalyzed C–H functionalization to even more challenging substrates, leveraging the distal recognition strategy traditionally beyond the scope of small-molecule catalysis.

## Methods

### General Procedure for C–H Functionalization of Spiro[3.3]­heptane

In an oven-dried 4 mL vial, a spiro[3.3]­heptane derivative (0.20 mmol, 1.0 equiv), Rh_2_(*S*-MegaBNP)_4_ (3.4 mg, 0.0001 mmol, 0.005 equiv, 0.5 mol %), 4 Å molecular sieves (100 wt %), and 1,1,1,3,3,3-hexafluoroisopropanol (HFIP) (5 μL, 8.40 mg, 0.05 mmol, 0.25 equiv) were added to CH_2_Cl_2_ (0.5 mL). The reaction mixture was stirred at 39 °C using a heating block. In a separate oven-dried 4 mL vial, the diazo compound (0.40 mmol, 2.0 equiv) was dissolved in CH_2_Cl_2_ (2.0 mL). This solution was loaded into a 3 mL syringe and added dropwise to the initial reaction mixture over the course of 3 h using a syringe pump. Upon completion of the addition, the reaction was stirred for an additional 1–2 h until complete consumption of the diazo compound. The mixture was then filtered through Celite to remove molecular sieve particulates and washed with CH_2_Cl_2_. The solvent was removed under reduced pressure, and the crude product was purified by flash chromatography (SiO_2_, ether, or ethyl acetate in hexane) to afford the desired C–H functionalization product.

## Supplementary Material



## Notes

Materials and methods, substrate and reagent details, computational studies, crystal structures, and spectroscopic data (PDF)
