# Supplementary material for: Enantioselective Synthesis of Axially Chiral Spiro[3.3]heptanes by Site-Selective C–H Functionalization
Source: ACS Catal. 2026 Mar 4;16(6):6057–66. doi: 10.1021/acscatal.6c00560 (PMC13010244; doi:10.1021/acscatal.6c00560)
Supplement: Supplementary file 1 [file cs6c00560_si_002.pdf]

Enantioselective Synthesis of Axially Chiral Spiro[3.3]heptanes by Site-Selective C–H Functionalization

Duc Ly[1], Ziyi Chen[1], Djamaladdin G. Musaev[1,2*], and Huw M. L. Davies[1*]

[1]Department of Chemistry, Emory University, 1515 Dickey Drive, Atlanta, Georgia 30322, United States

[2]Cherry L. Emerson Center for Scientific Computation, Emory University, Atlanta, Georgia 30322, United States

Corresponding author: hmdavie@emory.edu and dmusaev@emory.edu

Supporting Information

Complete experimental procedures, materials, computational details, and compound characterizations

1. **General Information**........S2
2. **Reaction optimization**........S3
3. **A General Procedure for C-H functionalization**........S13
4. **Determination of diastereomeric ratios**........S14
5. **Substrate synthesis**........S43
6. **Product characterization**........S76
7. **Product transformation**........S169
8. **Xray Crystallography**........S179
9. **Computational study**........S190
10. **References**........S208

1. General Information

All reagents and solvents were used as purchased from commercial sources (Sigma, Ambeed, Oakwood) for substrate synthesis unless otherwise noted. Dichloromethane used in C–H insertion reactions was prepared from solvent purification system. 4 Å molecular sieves were activated at 220 °C for 4 hours under vacuum and stored in an oven over 100 °C. All column chromatography was performed on silica gel (SiliaFlash® P60, 40-63 μm). Thin layer chromatographic (TLC) analysis was performed with aluminum-sheet silica gel plates. ^{1}H, ^{13}C and ^{19}F NMR spectra were recorded at 800MHz or 600 MHz on Bruker-800 spectrometer, Bruker-600 spectrometer or Varian IVONA-600 spectrometer (^{13}C at 151 MHz), 500 MHz on Varian INOVA-500 spectrometer, or 400 MHz (^{13}C at 101 MHz, ^{19}F at 376 MHz) on Bruker-400 spectrometer and all were reported in parts per million (ppm). Unless otherwise noted, ^{1}H, ^{13}C and ^{19}F NMR spectra were performed in solutions of deuterated chloroform ($CDCl_3$) with the residue chloroform set as an internal standard (7.26 ppm for 1 H, and 77.16 ppm for ^{13}C). Abbreviations for signal multiplicity are as follows: br = broad, s = singlet, d = doublet, t = triplet, q = quartet, m = multiplet, dd = doublet of doublet, tt = triplet of triplet, qt = quartet of triplet, dtd = doublet of triplet of doublet. Coupling constants (J values) were calculated directly from the spectra. Mass spectra were taken on a Thermo Finnigan LTQ-FTMS spectrometer with APCI, ESI or NSI. The enantiomeric excess (ee) was determined by High Performance Liquid Chromatography analysis was performed on either Agilent 1100 Technologies HPLC or Super Critical Fluid Chromatography using Water Acquity UPC^2 SFC system and the data outlined below varies in presentation based on the software used for each system. HPLC/SFC traces are reported based on the racemic retention times. The HPLC instruments used isopropanol/hexane gradient and commercial ChiralPak/ChiralCel columns from Daicel Chemical Industries, notably ChiralPak AD-H (5 μm particle size, 4.6 mm vs. 250 mm), ChiralCel OZ-H (5 μm particle size, 4.6 mm vs. 250 mm), and ChiralCel OD-H (5 μm particle size, 4.6 mm vs. 250 mm), ChiralCel AS-H (5 μm particle size, 4.6 mm vs. 250 mm), ChiralCel OJ-H (5 μm particle size, 4.6 mm vs. 250 mm), and Regis (S,S) Whelk-O1 5/100 Kromasil. The SFC system utilized supercritical fluid CO_2 with cosolvents of 1:1 MeOH:IPA with 0.2% formic acid with SFC columns: Trefoil AMY1 Column (2.5 μm, 3.0 mm X 150 mm), Trefoil CEL1 Column (2.5 μm, 3.0 mm X 150 mm), Trefoil CEL2 Column (2.5 μm, 3.0 mm X 150 mm), Regis (S,S) Whelk-O 1 Kromasil (3.5 μm, 3.0 mm X 150 mm), ChiralPak AD-3 (3.0 μm, 3.0 mm X 150 mm SFC), ChiralCel OZ-3 (3.0 μm, 3.0 mm X 150 mm), ChiralCel OD-3 (3.0 μm, 3.0 mm X 150 mm SFC), ChiralCel OX-3 (3.0 μm, 3.0 mm X 150 mm SFC); ChiralCel OJ-3 (3.0 μm, 3.0 mm X 150 mm SFC); ChiralPak AS-3 (3.0 μm, 3.0 mm X 150 mm SFC). The racemic samples were prepared by using either $Rh_2(esp)_2$ or Rh_2(*R/S*-MegaBNP)$_4$ (a mixture of 1:1 Rh_2(*S*-MegaBNP)$_4$ and Rh_2(*R*-MegaBNP)$_4$) as a catalyst.

2. Reaction optimization

a. Reaction with Aryl spiro[3.3]heptane

Table S1. Reaction with spiro[3.3]heptane 16[a]

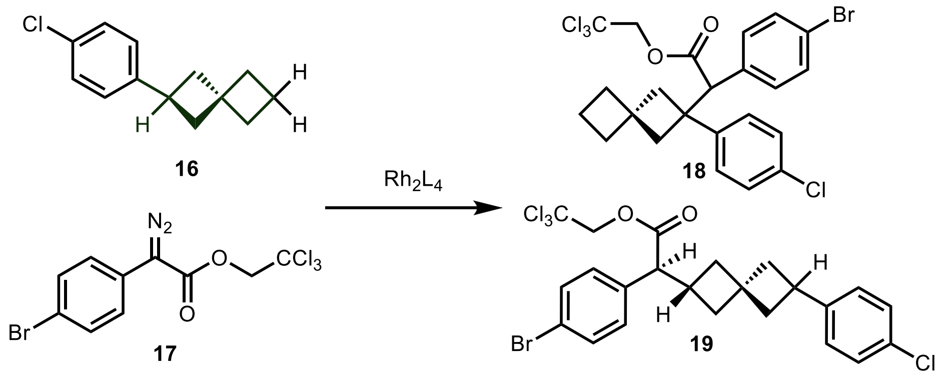

Entry	*Catalyst* *(1.0 mol%)*	*Yield, %*	rr (18:19)	*dr*
1	Rh_2(*S*-TPPTTL)$_4$	56	1:1.7	1:1
2	Rh_2(*S*-*tetra*-MeO-C_6H_4NTTL)$_4$	55	1:1.3	1:1
3	Rh_2(*S*-2Cl5BrTPCP)$_4$	55	<1:20	1:1

[a]Reaction conditions: **17** (0.05 mmol), **16** (1.5 equiv), Rh_2L_4 (1.0 mol%) in CH_2Cl_2 at 39 °C. Yields are isolated yield. Regioisomeric ratio (rr) was determined by ^{1}H-NMR. Diastereomeric ratio (dr) was determined based on isolated material

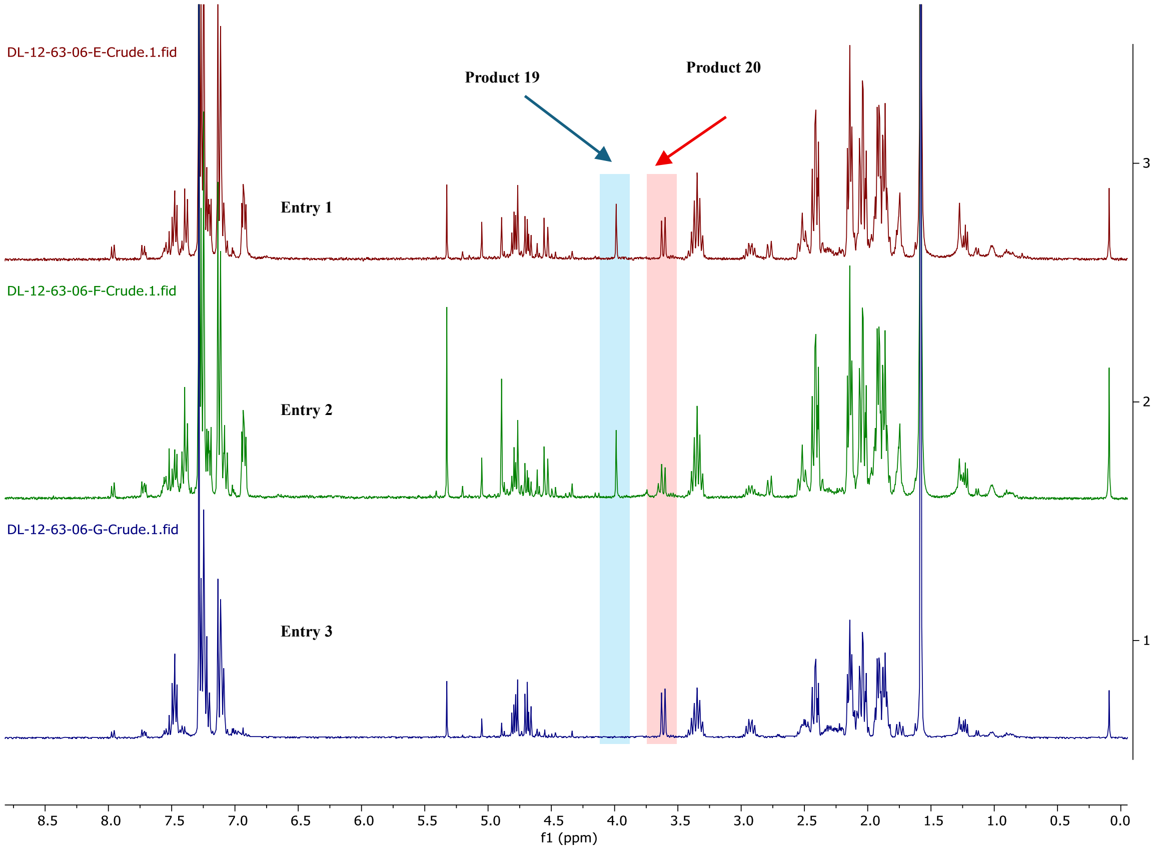

Figure S1. Crude ^{1}H-NMR for C-H functionalization reactions studied in Table S1

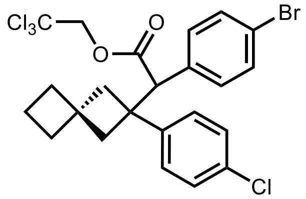

2,2,2-trichloroethyl 2-(4-bromophenyl)-2-(2-(4-chlorophenyl)spiro[3.3]heptan-2-yl)acetate (Compound 18)

This compound was synthesized via the C-H functionalization reaction studied in **Table S1**. The product was purified from the crude reaction mixture by flash chromatography (SiO_2, 0-10% diethyl ether in hexane) to give **compound 18** as a clear oil.

$\mathbf{R_f}$ (9Hex/1Et_2O) = 0.80 (CAM)

^{1}H NMR (600 MHz, $CDCl_3$) δ 7.36 (d, *J* = 8.4 Hz, 2H), 7.18 (d, *J* = 8.4 Hz, 2H), 6.91 (dd, *J* = 8.3, 5.4 Hz, 4H), 4.75 (d, *J* = 12.0 Hz, 1H), 4.52 (d, *J* = 11.9 Hz, 1H), 3.96 (s, 1H), 2.78 – 2.71 (m, 1H), 2.50 (ddd, *J* = 17.0, 11.6, 2.0 Hz, 2H), 2.36 (dd, *J* = 12.3, 2.5 Hz, 1H), 2.01 – 1.88 (m, 2H), 1.73 (m, 4H).

^{13}C NMR (151 MHz, $CDCl_3$) δ 170.2, 144.1, 133.7, 132.2, 131.9, 131.1, 129.8, 127.6, 122.1, 94.7, 74.4, 60.4, 45.32, 45.03, 44.97, 38.1, 36.1, 35.5, 16.7.

HRMS (-p APCI) calcd. for [$C_{23}H_{20}O_2{}^{79}Br^{35}Cl_4$] ($[M-H]^-$) 546.9406 found 546.9420.

DL-12-63-05-H-F1-Clean.10.fid

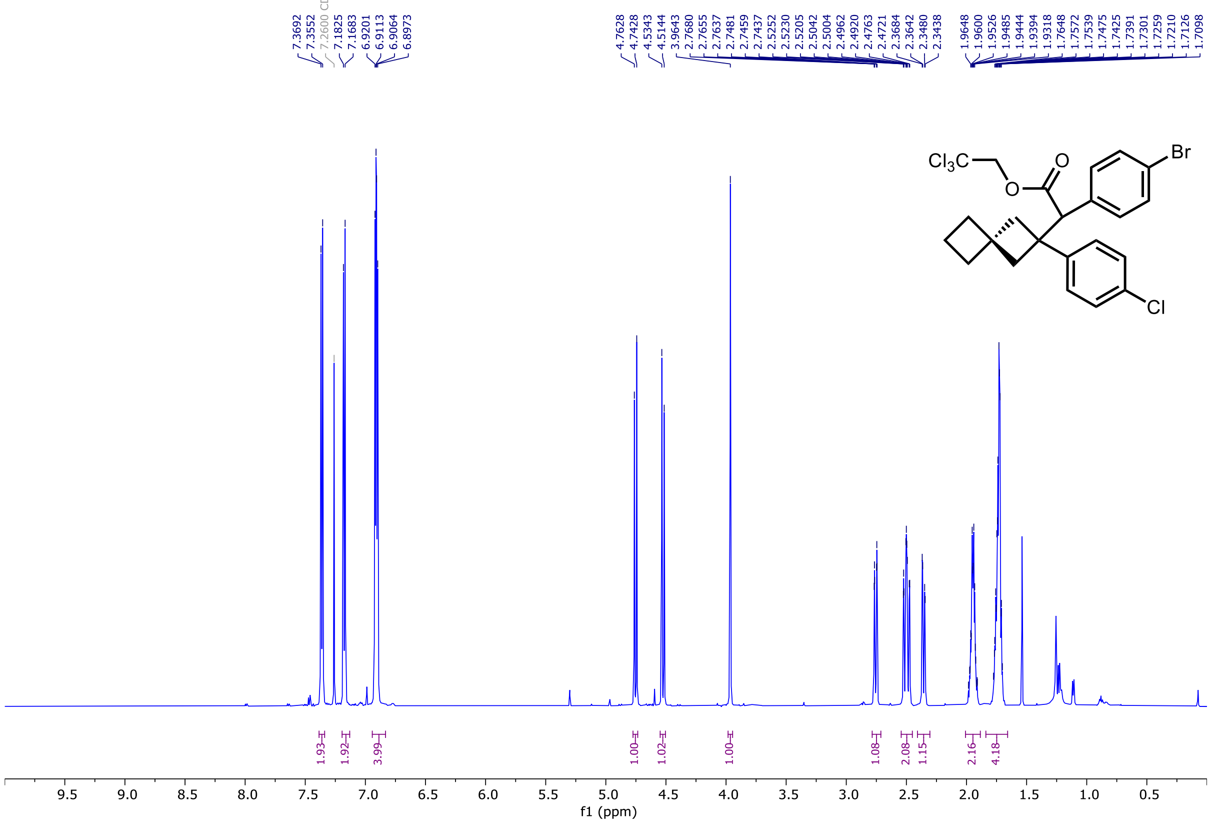

DL-12-63-05-H-F1-Clean.11.fid

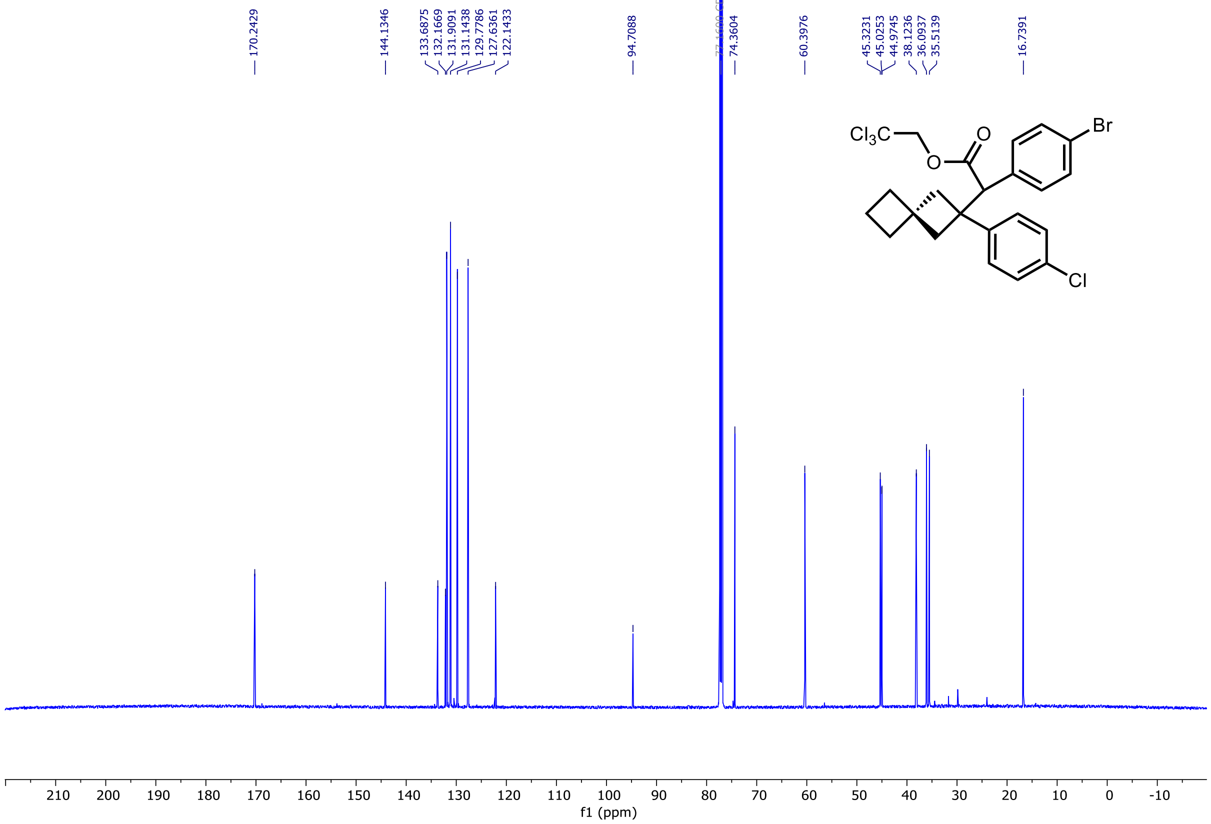

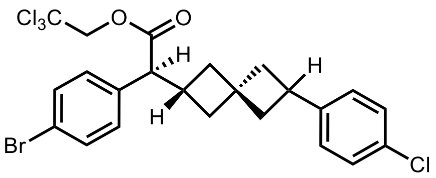

2,2,2-trichloroethyl (2S)-2-(4-bromophenyl)-2-(6-(4-chlorophenyl)spiro[3.3]heptan-2-yl)acetate (Compound 19)

This compound was synthesized via the C-H functionalization reaction studied in **Table S1**. The product was purified from the crude reaction mixture by flash chromatography (SiO_2, 0-10% diethyl ether in hexane) to give **compound 19** as a clear oil.

R_f (9Hex/1Et_2O) = 0.40 (CAM)

^{1}H NMR (600 MHz, $CDCl_3$) δ 7.49 – 7.42 (m, 4H), 7.25 – 7.16 (m, 8H), 7.10 – 7.05 (m, 4H), 4.76 (dd, *J* = 12.0, 9.3 Hz, 2H), 4.70 – 4.63 (m, 2H), 3.59 (d, *J* = 11.0 Hz, 2H), 3.34 (dp, *J* = 37.5, 8.8 Hz, 2H), 2.91 (dp, *J* = 11.0, 8.2 Hz, 2H), 2.54 – 2.42 (m, 3H), 2.35 – 2.24 (m, 2H), 2.20 (ddd, *J* = 11.8, 7.8, 4.2 Hz, 1H), 2.15 – 1.97 (m, 6H), 1.91 – 1.81 (m, 2H), 1.73 (dd, *J* = 11.3, 8.6 Hz, 1H), 1.58 (dd, *J* = 11.8, 8.7 Hz, 1H). (Analyzed as a 1:1 mixture of 2 diastereomers)

^{13}C NMR (151 MHz, $CDCl_3$) δ 171.25, 171.23, 144.14, 144.09, 135.8, 135.7, 131.91, 131.90, 131.52, 131.51, 130.11, 130.08, 128.4, 127.9, 127.8, 121.8, 94.94, 94.91, 74.2, 57.7, 57.6, 42.7, 42.6, 42.1, 42.0, 40.5, 39.5, 39.4, 38.4, 35.6, 34.2, 33.1, 33.0.

HRMS (+p APCI) calcd. for [$C_{23}H_{22}O_2{}^{79}Br^{35}Cl_4$] ([M+H]$^+$) 548.9563 found 548.9565.

DL-12-63-05-G-F2-Clean.10.fid

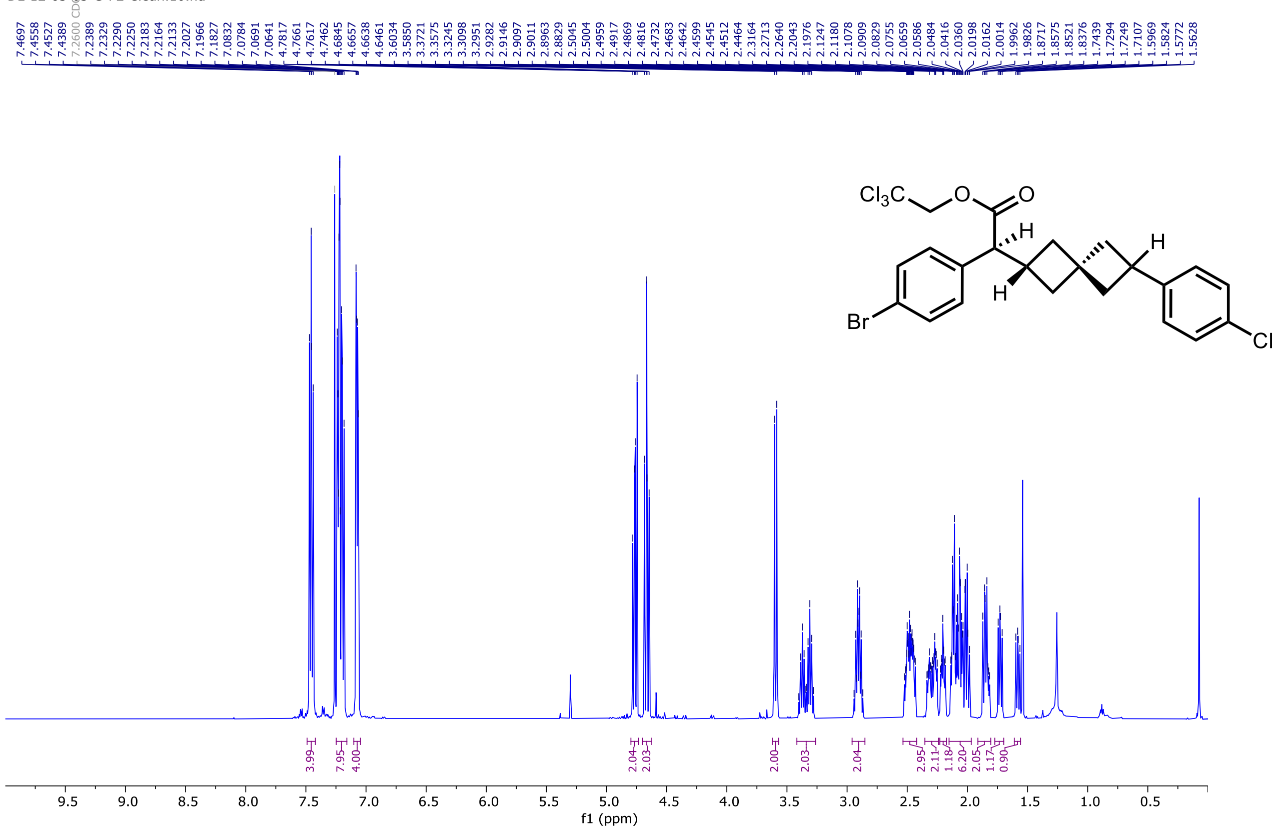

DL-12-63-05-G-F2-Clean.11.fid

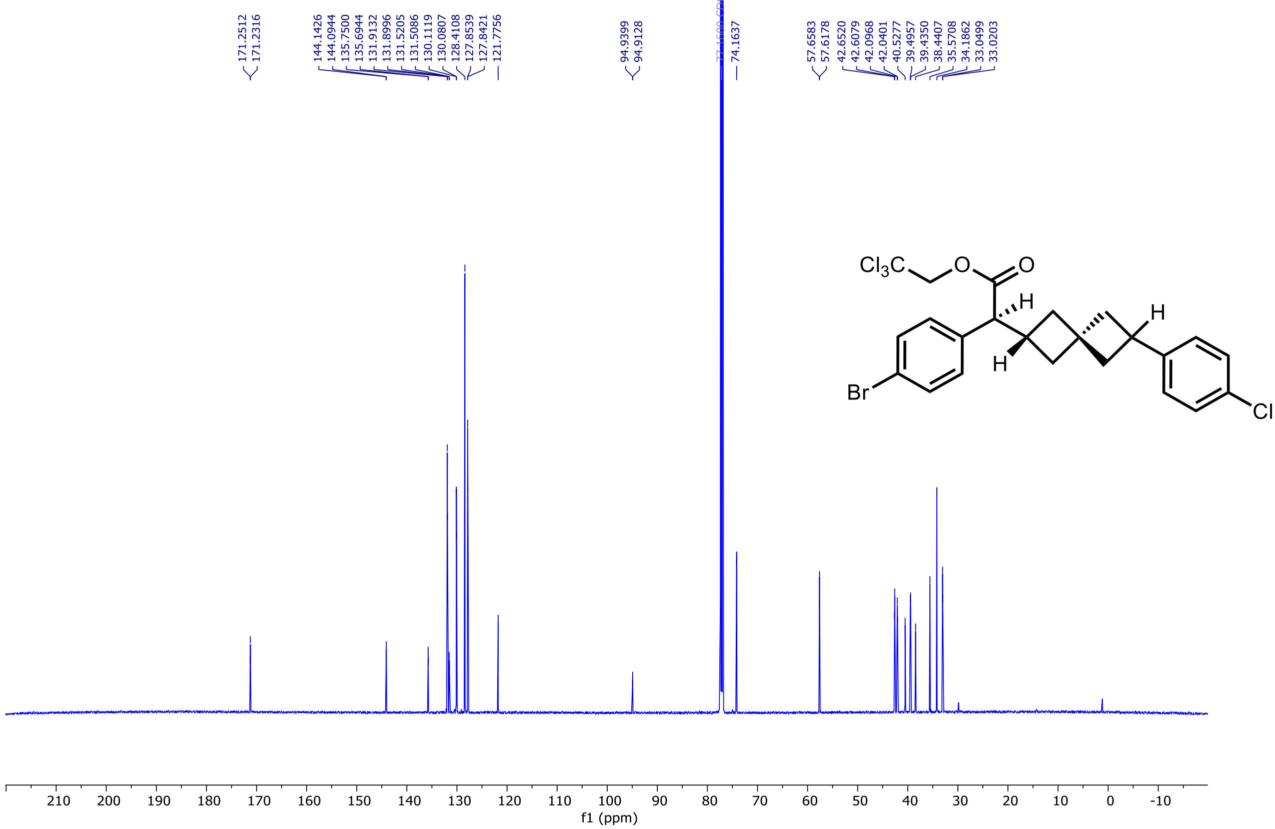

b. Reaction with *N*-phthalimido spiro[3.3]heptane

Table S2. Reaction with spiro[3.3]heptane 20[a]

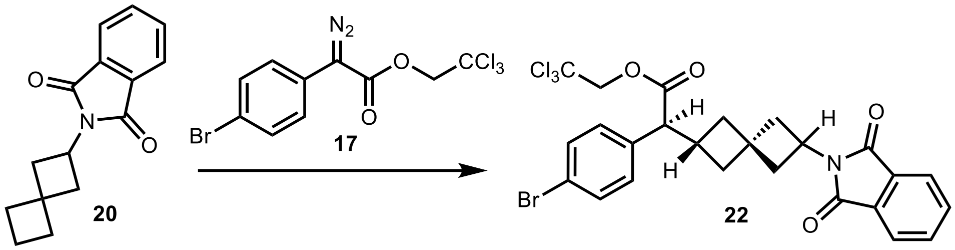

Entry	20	17	*Catalyst (0.5 mol%)*	*Yield, %*	*dr*	*ee ,%*
1	1.5	1	Rh_2(*S*-TPPTTL)$_4$	17	1.7:1	-76
2	1.5	1	Rh_2(*S*-*tetra*-MeO-C_6H_4NTTL)$_4$	16	1.2:1	46
3	1.5	1	Rh_2(*S*-TCPTAD)$_4$	32	1.5:1	-77
4	1.5	1	Rh_2(*S*-PTAD)$_4$	37	1.5:1	76
5	1.5	1	Rh_2(*S*-2Cl5BrTPCP)$_4$	56	1:2	90
6	1.5	1	Rh_2(*S*-MegaBNP)$_4$	44	>20:1	99
7	1	1.5	Rh_2(*S*-MegaBNP)$_4$	68	>20:1	99
8	1	2	Rh_2(*S*-MegaBNP)$_4$	75	>20:1	99
9[b]	1	2	Rh_2(*S*-MegaBNP)$_4$	92	>20:1	99

[a]Reaction conditions: **20** (0.10 mmol), **17**, Rh_2L_4 (1.0 mol%) in CH_2Cl_2 at 39 °C. Yields are isolated yields. Regioisomeric (rr) and diastereomeric ratios (dr) were determined by ^{1}H-NMR. The regioisomeric ratio (rr) is >20:1 in all cases. [b]HFIP (0.20 equiv) was added.

Because of the low resolution of 2 diastereomer on ^{1}H-NMR, the crude NMR was taken on 800 MHz ^{1}H-NMR for analyzing the diastereoselectivity. The diastereoselectivity was further confirmed by analyzing the purified product. It is worth noting that the 2 diastereomers are inseparable by flash chromatography (SiO_2), and the ratio was confirmed to be not changed after purification (**Figure S4** and **Figure S5**). This ensures the reliability of the analysis of diastereoselectivity based on purified products. As a result, the diastereoselectivity of the reaction scope was based on the purified products.

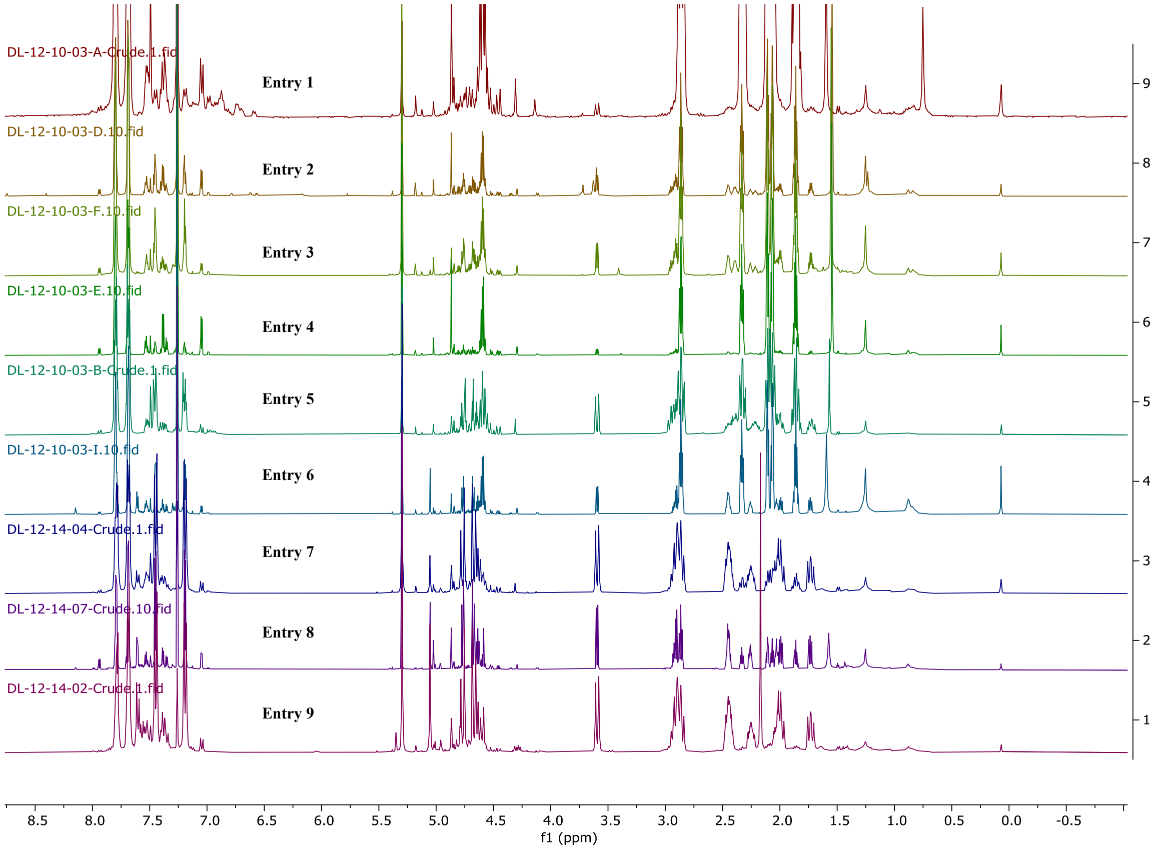

Figure S2. Crude ^{1}H-NMR for C-H functionalization reactions studied in Table S2

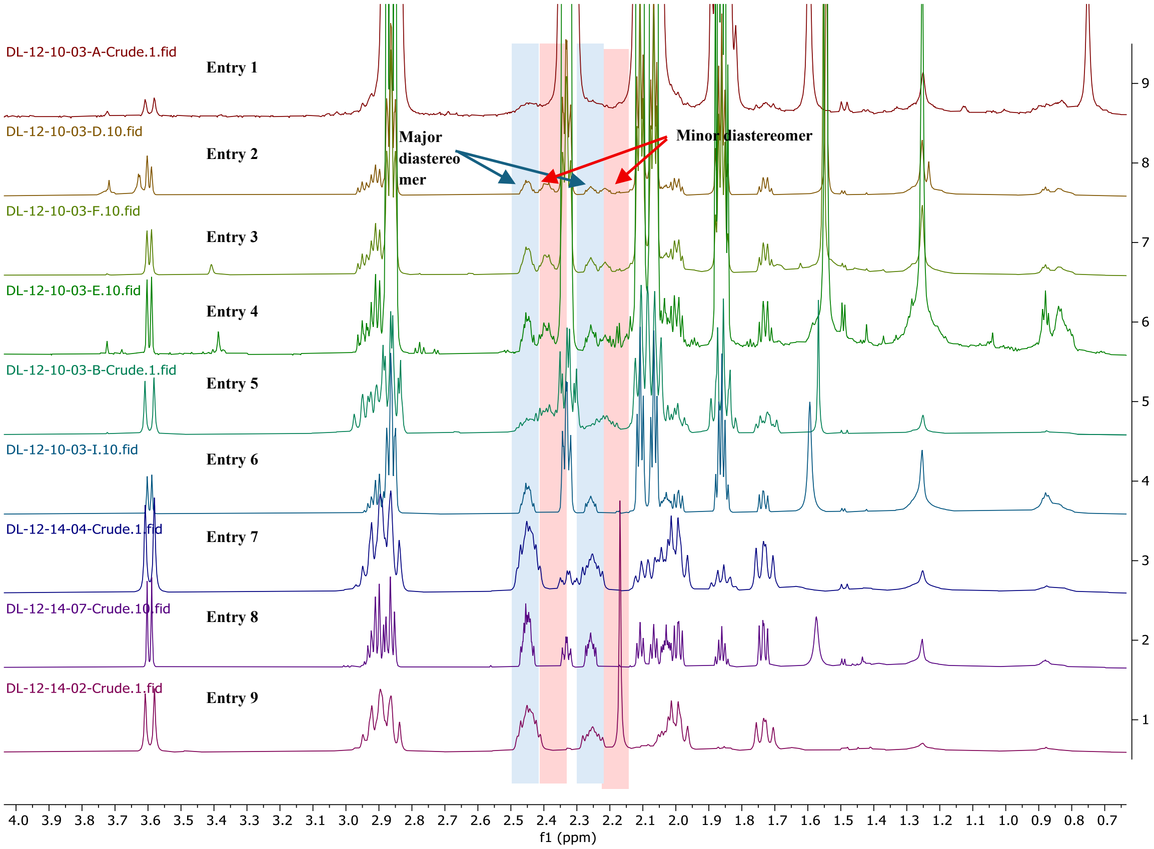

Figure S3. Crude ^{1}H-NMR for C-H functionalization reactions studied in Table S2, zoom in area of interest

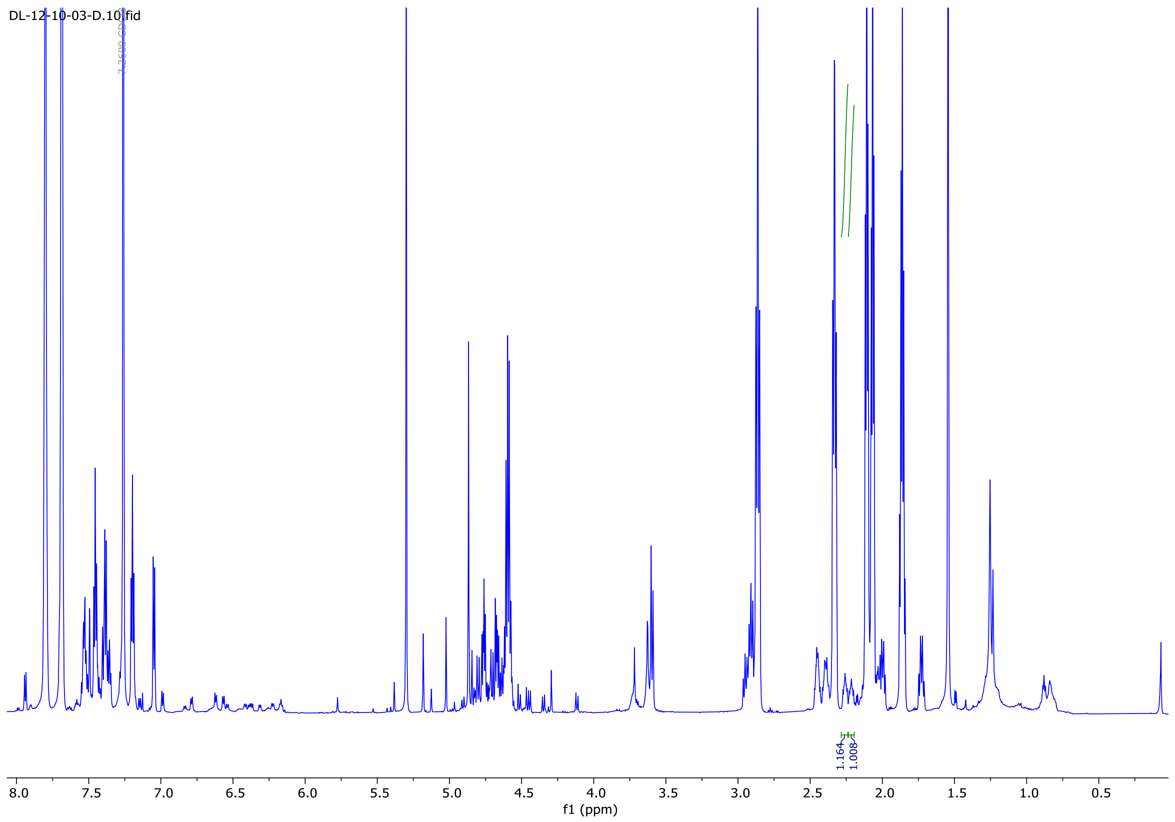

Figure S4. Crude ^{1}H-NMR for C-H functionalization reaction (Table S2, entry 2)

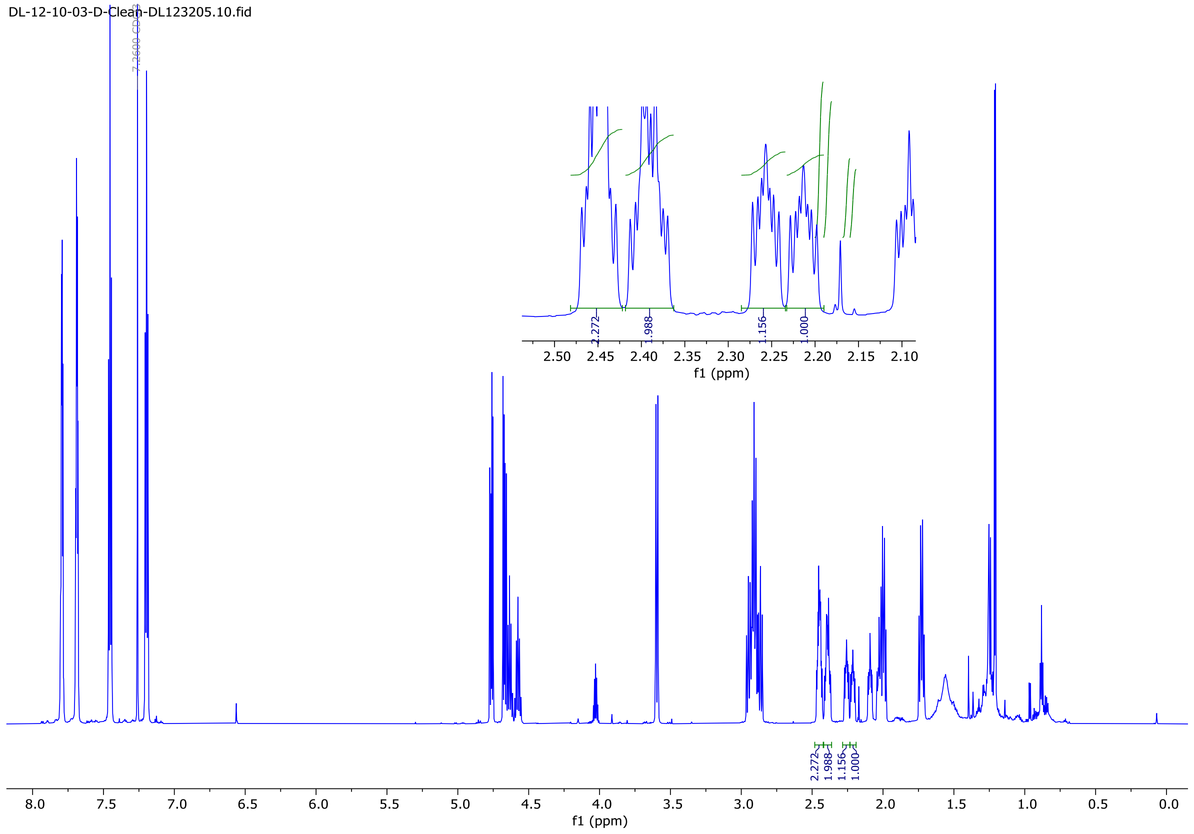

Figure S5. Purified ^{1}H-NMR for C-H functionalization reaction (Table S2, entry 2)

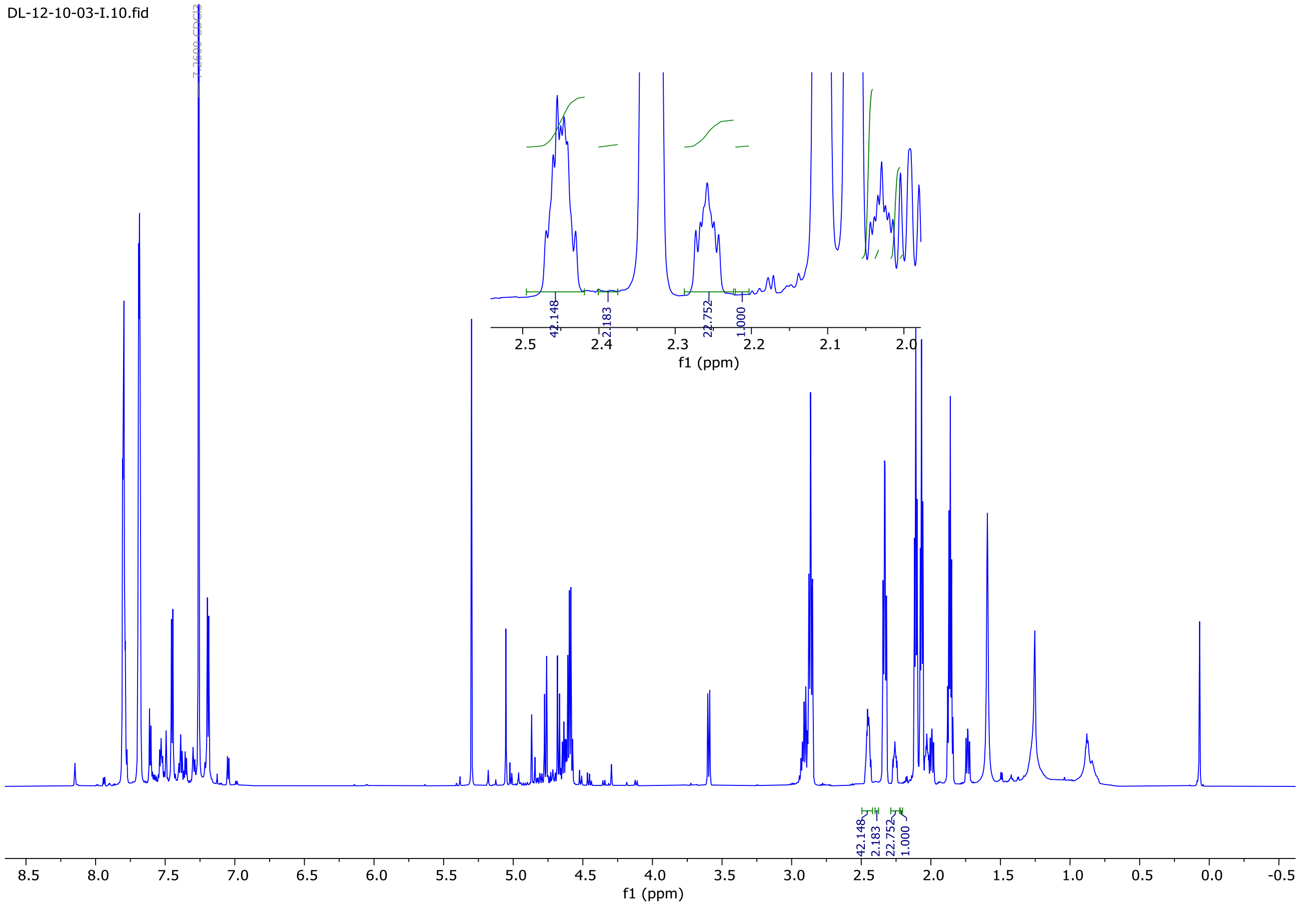

Figure S6. Crude ^{1}H-NMR for C-H functionalization reaction (Table S2, entry 6)

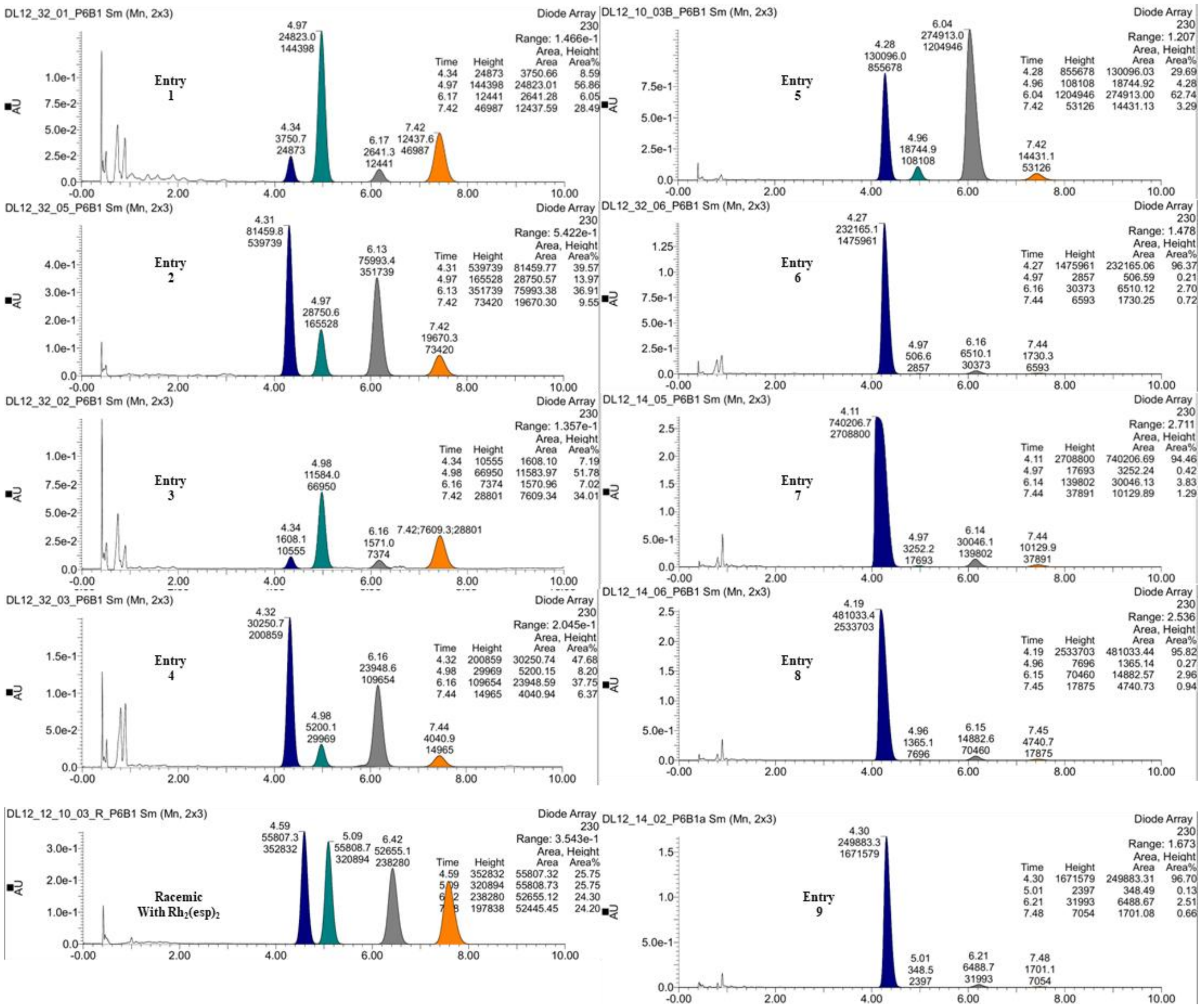

Figure S7. SFC analysis for purified products obtained from reaction studied in Tables S2. Diastereomer 1: 4.30 min and 5.01 min; Diastereomer 2: 6.21 min and 7.48 min.

3. A General Procedure for C-H functionalization

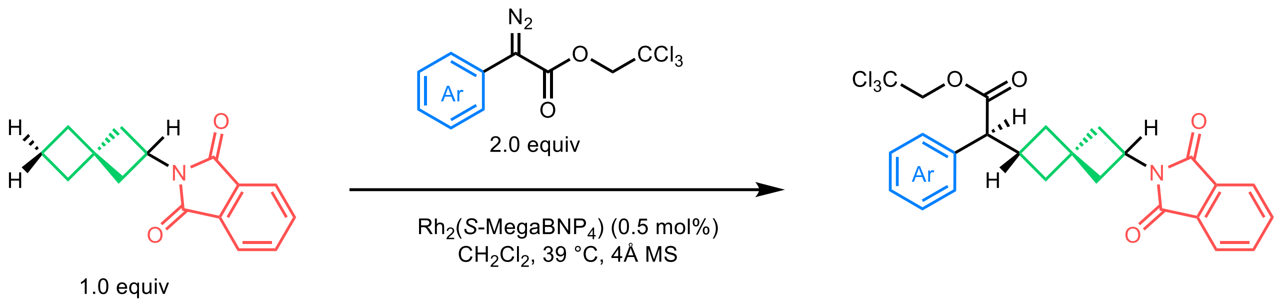

In an oven-dried 4 mL vial, spiro[3.3]heptane derivative (0.20 mmol, 1.0 equiv), Rh_2(S-MegaBNP)$_4$ (3.4 mg, 0.0001 mmol, 0.005 equiv, 0.5 mol%), 4 Å molecular sieves (100 wt%), and 1,1,1,3,3,3-hexafluoroisopropanol (HFIP) (5 µL, 8.40 mg, 0.05 mmol, 0.25 equiv) were added to 0.5 mL of CH_2Cl_2. The reaction mixture was stirred at 39 °C using a heating block. In a separate oven-dried 4 mL vial, the diazo compound (0.40 mmol, 2.0 equiv) was dissolved in 2.0 mL of CH_2Cl_2. This solution was loaded into a 3 mL syringe and added dropwise to the initial reaction mixture over the course of 3 hours using a syringe pump. Upon completion of the addition, the reaction was stirred for an additional 1–2 hours until complete consumption of the diazo compound. The mixture was then filtered through Celite to remove molecular sieve particulates and washed with CH_2Cl_2. The solvent was removed under reduced pressure, and the crude product was purified by flash chromatography (SiO_2, ether or ethyl acetate in hexane) to afford the desired C–H functionalization product.

Note: Because of the low resolution between 2 diastereomers in crude ^{1}H-NMR and the fact that we did not observe the diastereoenrichment during the purification for the model study, the diastereoselectivity for substrate scope study was determined on the purified product.

4. Determination of diastereomeric ratios

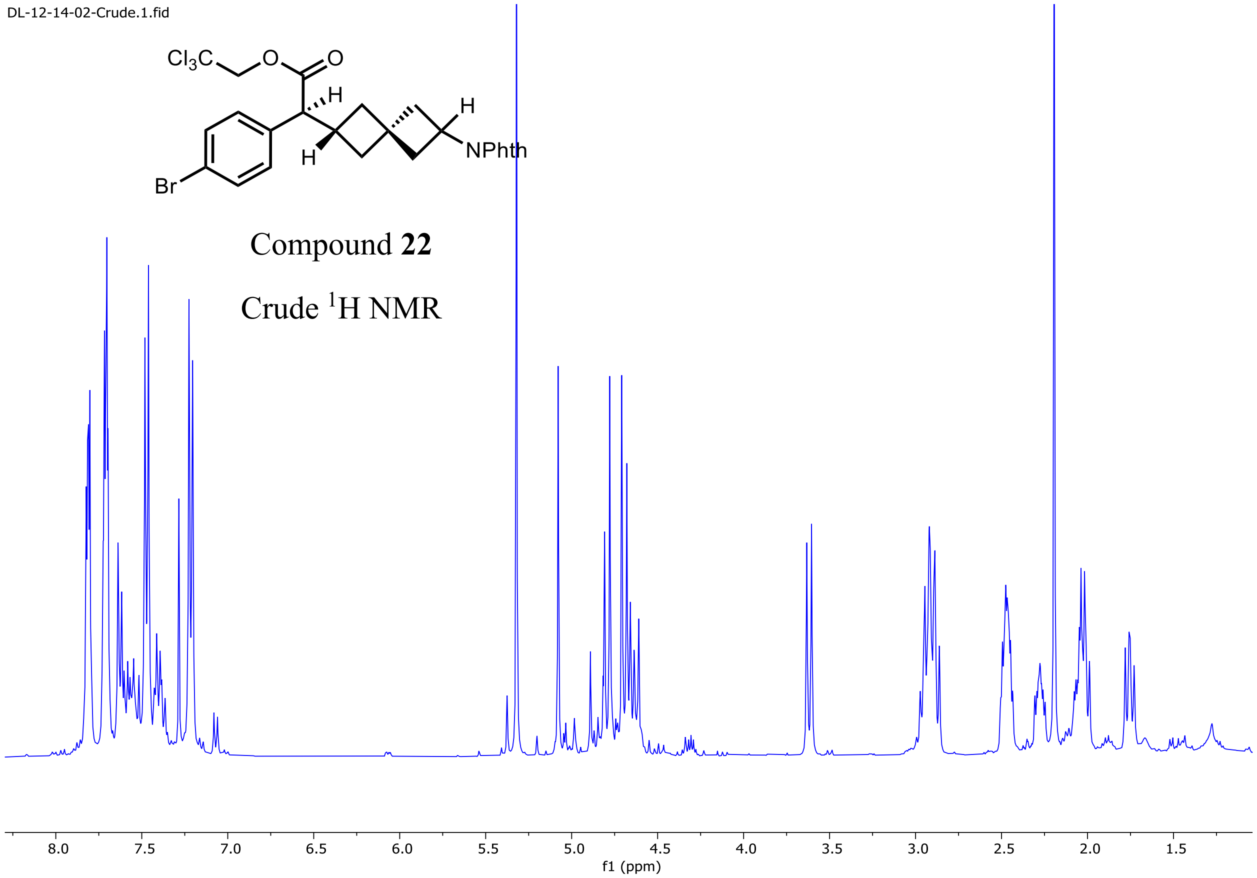

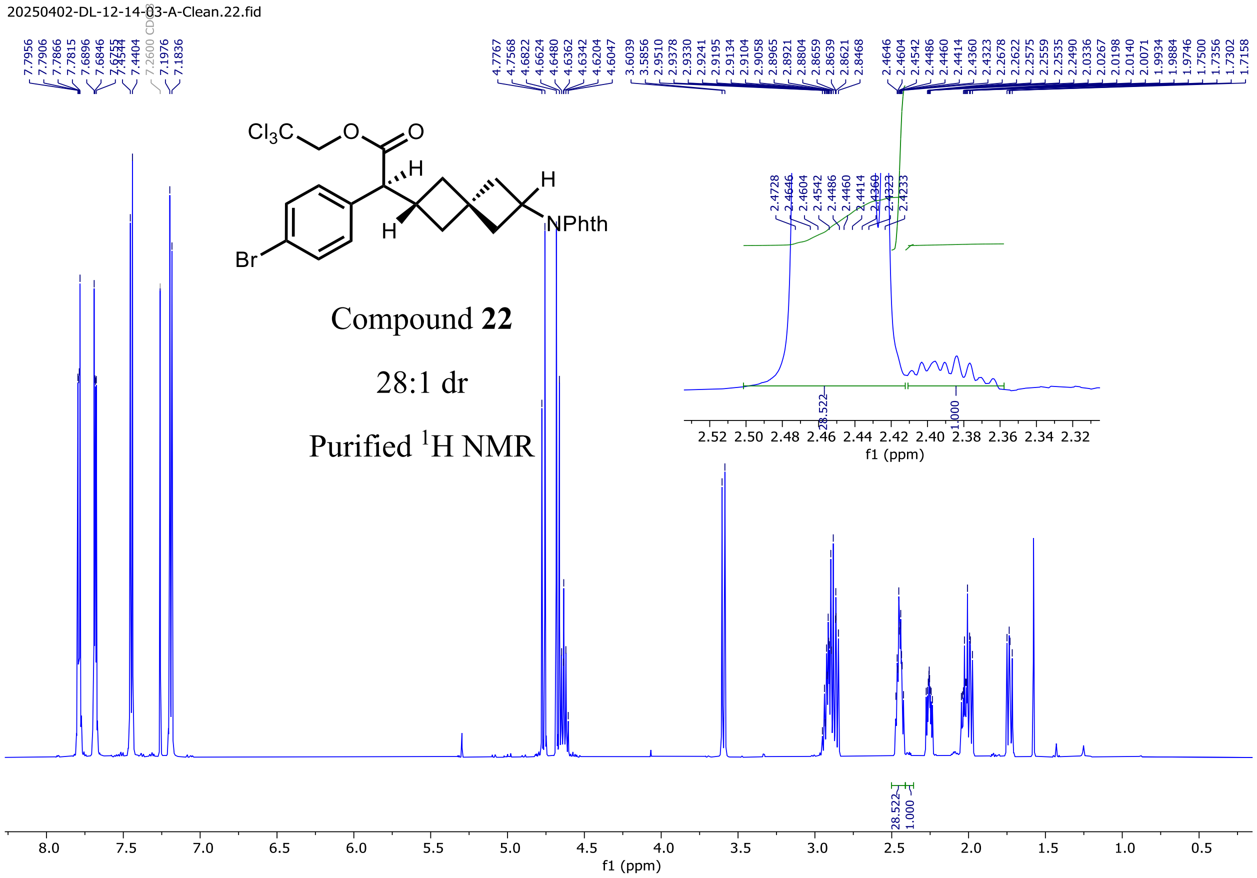

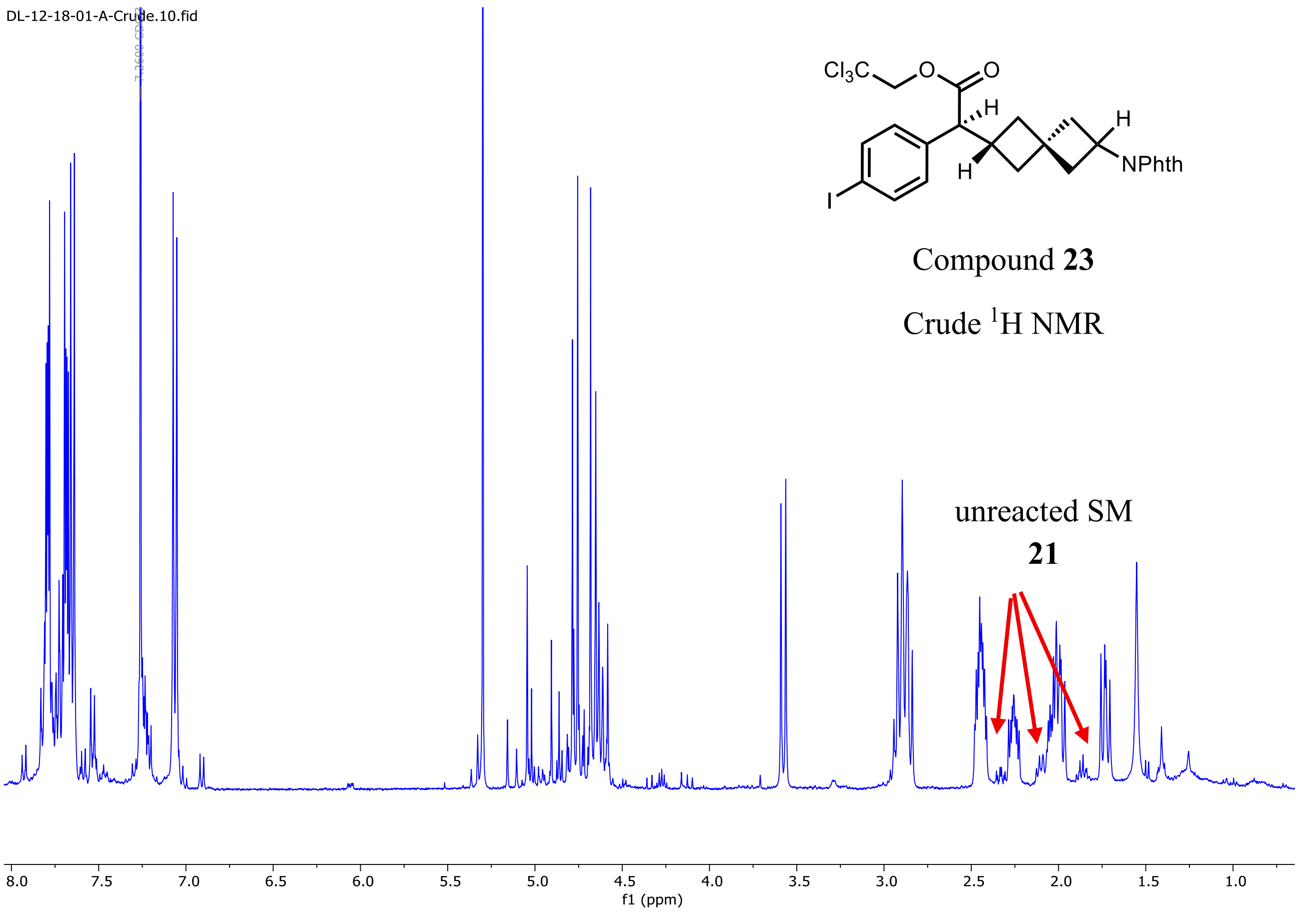
DL-12-18-01-A-Crude.10.fid
Compound **23**
Crude ¹H NMR
unreacted SM
21
f1 (ppm)

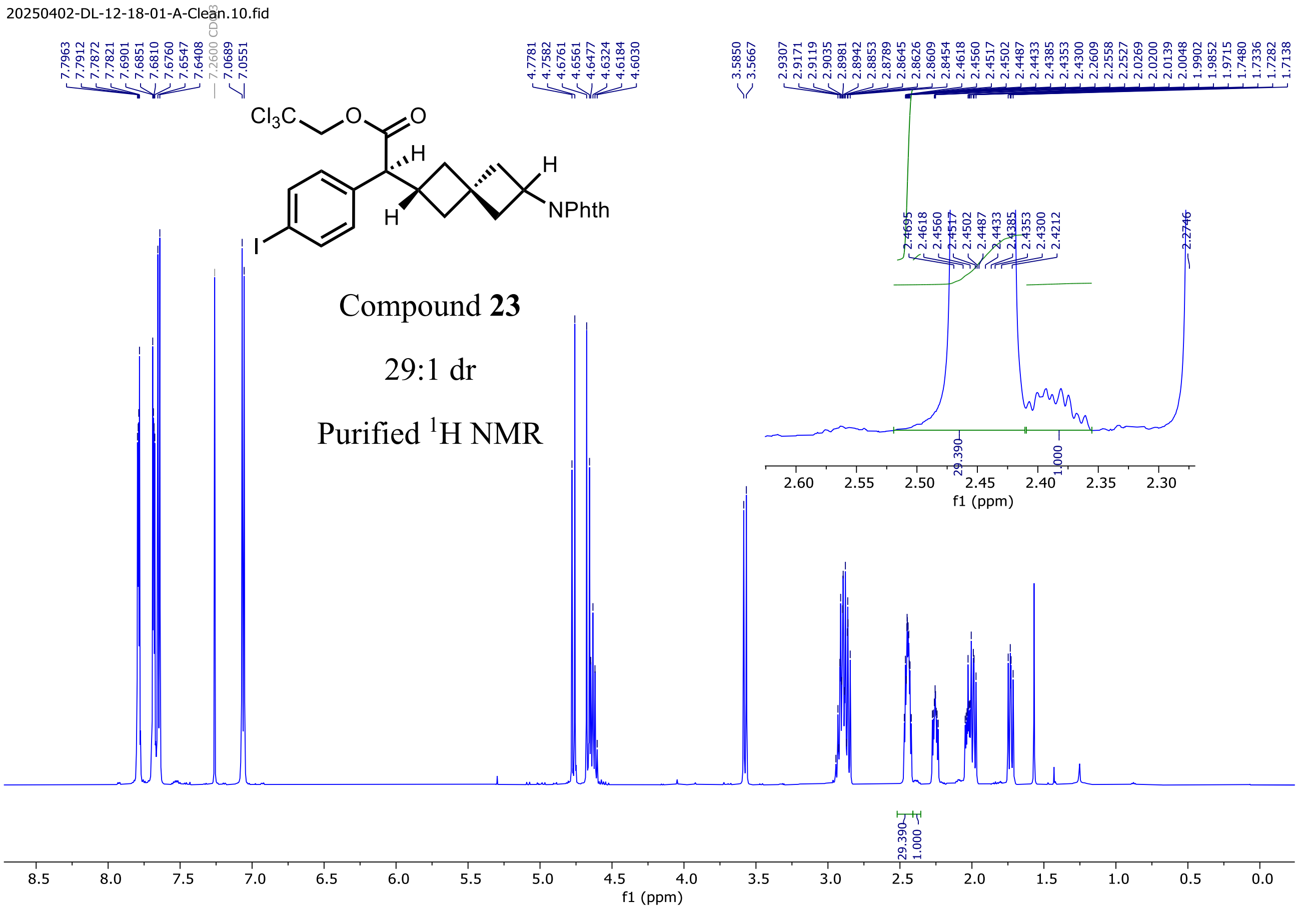
20250402-DL-12-18-01-A-Clean.10.fid
7.7963 7.7912 7.7872 7.7821 7.6901 7.6851 7.6810 7.6760 7.6547 7.6408 7.2600 CDCl3 7.0689 7.0551
4.7781 4.7582 4.6761 4.6561 4.6477 4.6324 4.6184 4.6030
3.5850 3.5667
2.9307 2.9171 2.9119 2.9035 2.8981 2.8942 2.8853 2.8789 2.8645 2.8626 2.8609 2.8454 2.4618 2.4560 2.4517 2.4502 2.4487 2.4433 2.4385 2.4353 2.4300 2.2609 2.2558 2.2527 2.0269 2.0200 2.0139 2.0048 1.9902 1.9852 1.9715 1.7480 1.7336 1.7282 1.7138
Compound **23**
29:1 dr
Purified ¹H NMR
2.4695 2.4618 2.4560 2.4517 2.4502 2.4487 2.4433 2.4385 2.4353 2.4300 2.4212
2.2746
29.390
1.000
f1 (ppm)

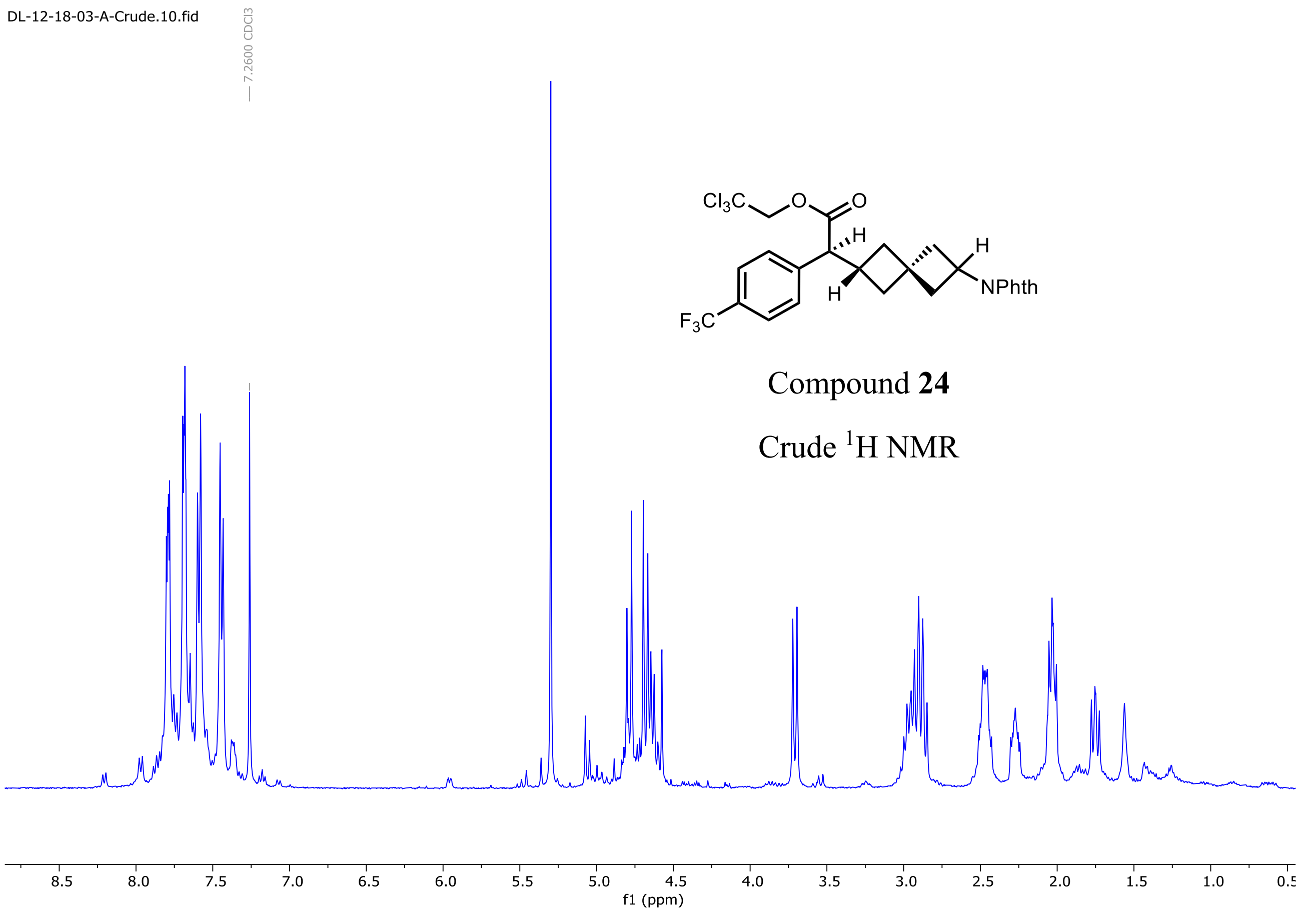
DL-12-18-03-A-Crude.10.fid
7.2600 CDCl3
Cl3C
O
O
H
H
H
NPhth
F3C
Compound 24
Crude 1H NMR
f1 (ppm)

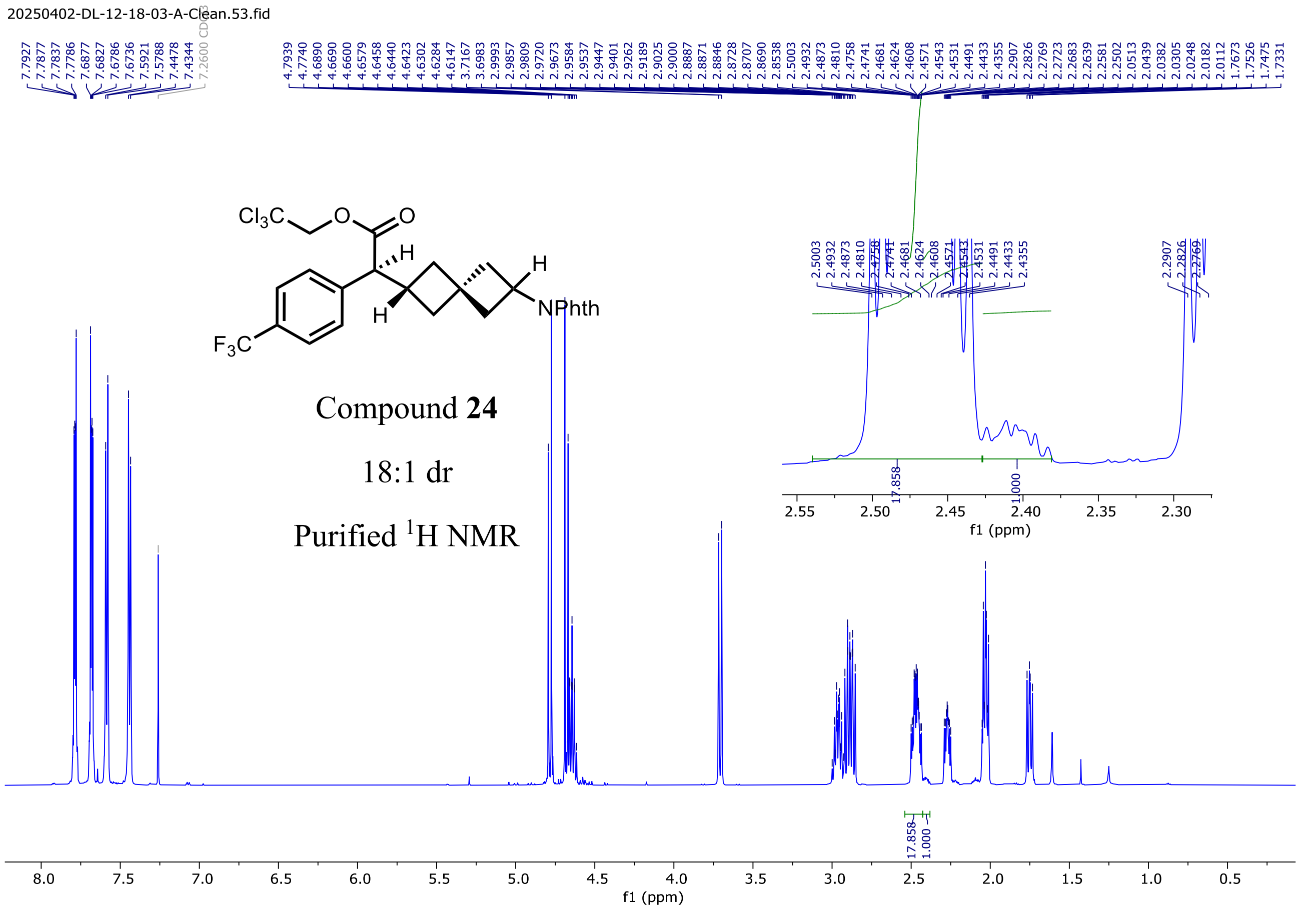
20250402-DL-12-18-03-A-Clean.53.fid
Cl3C
O
O
H
H
H
NPhth
F3C
Compound 24
18:1 dr
Purified 1H NMR
17.858
1.000
f1 (ppm)

DL-12-18-06-A-Crude.1.fid

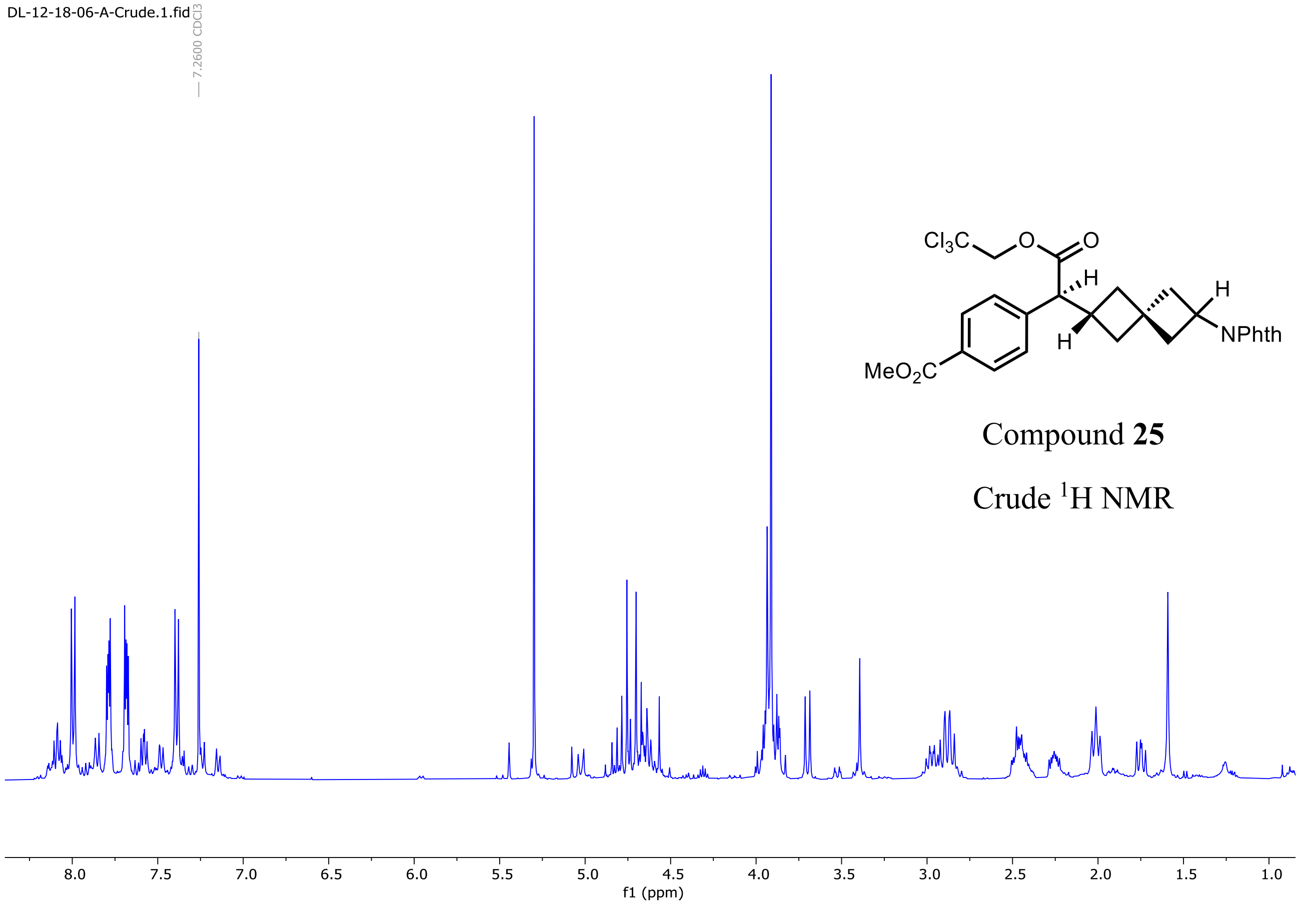

Compound **25**

Crude ^{1}H NMR

20250803-DL-12-18-06-A-Clean.10.fid

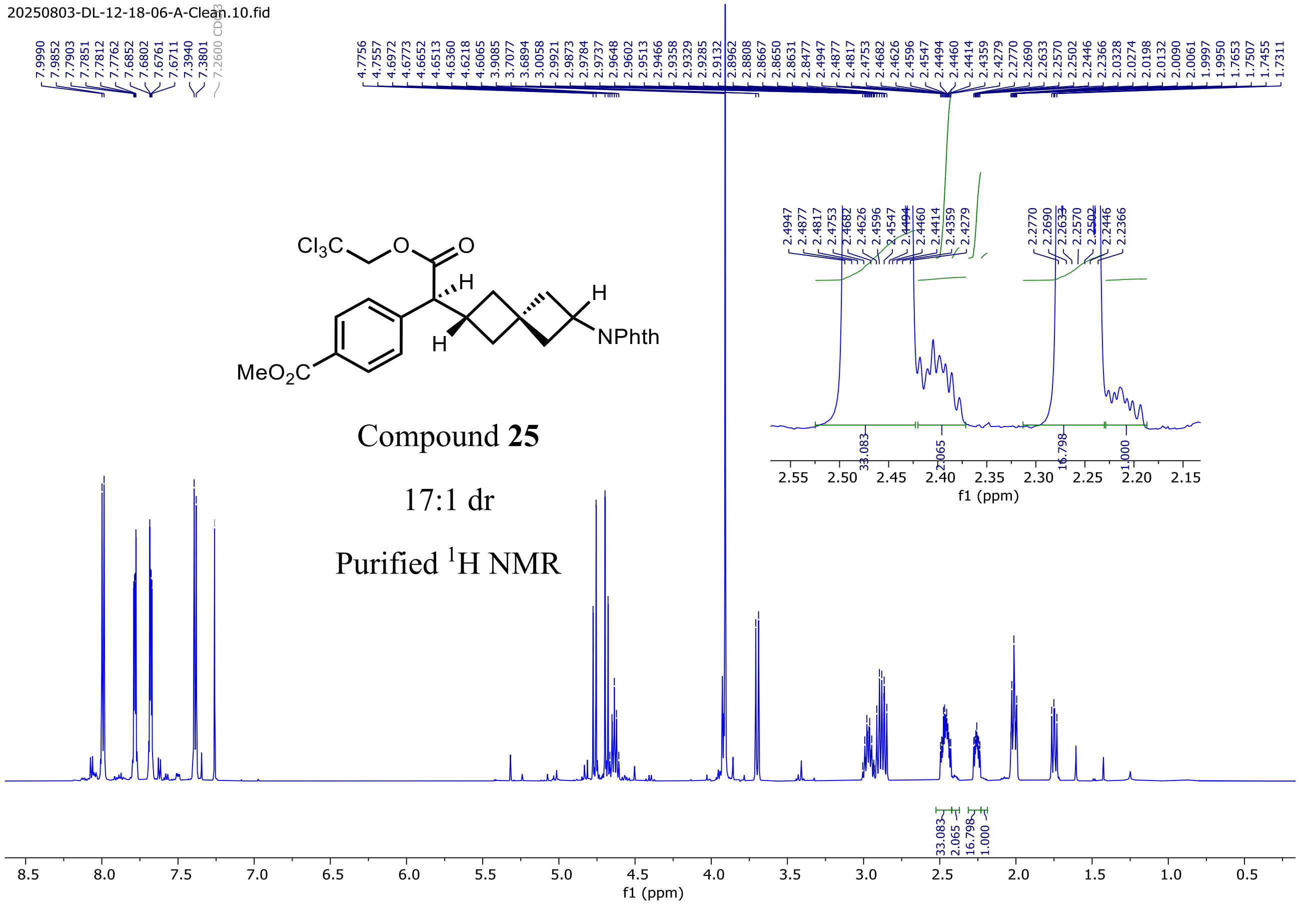

Compound **25**

17:1 dr

Purified ^{1}H NMR

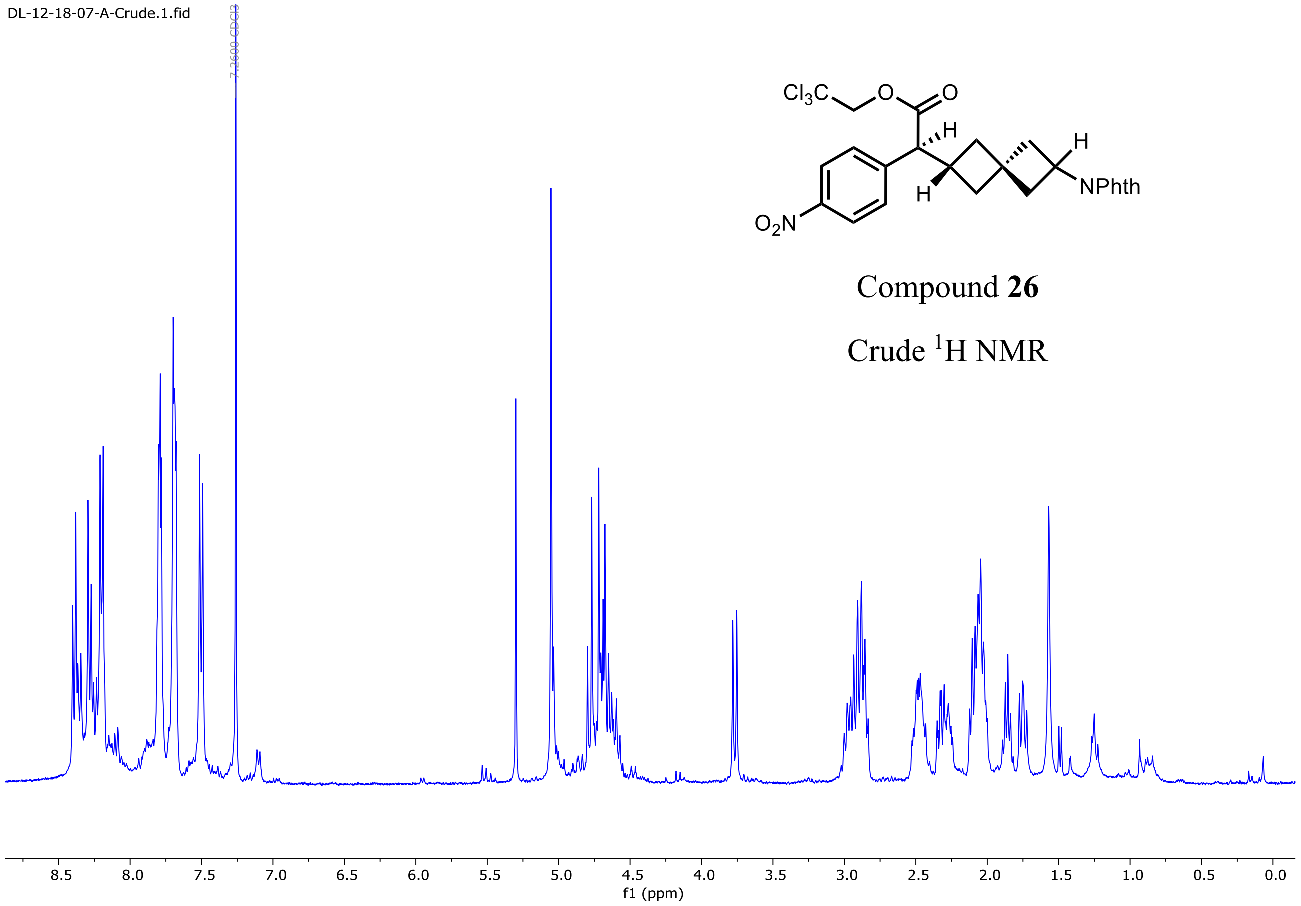
DL-12-18-07-A-Crude.1.fid
7.2600 CDCl3
Cl_3C
O
O
H
H
H
NPhth
O_2N
Compound **26**
Crude ^{1}H NMR
f1 (ppm)

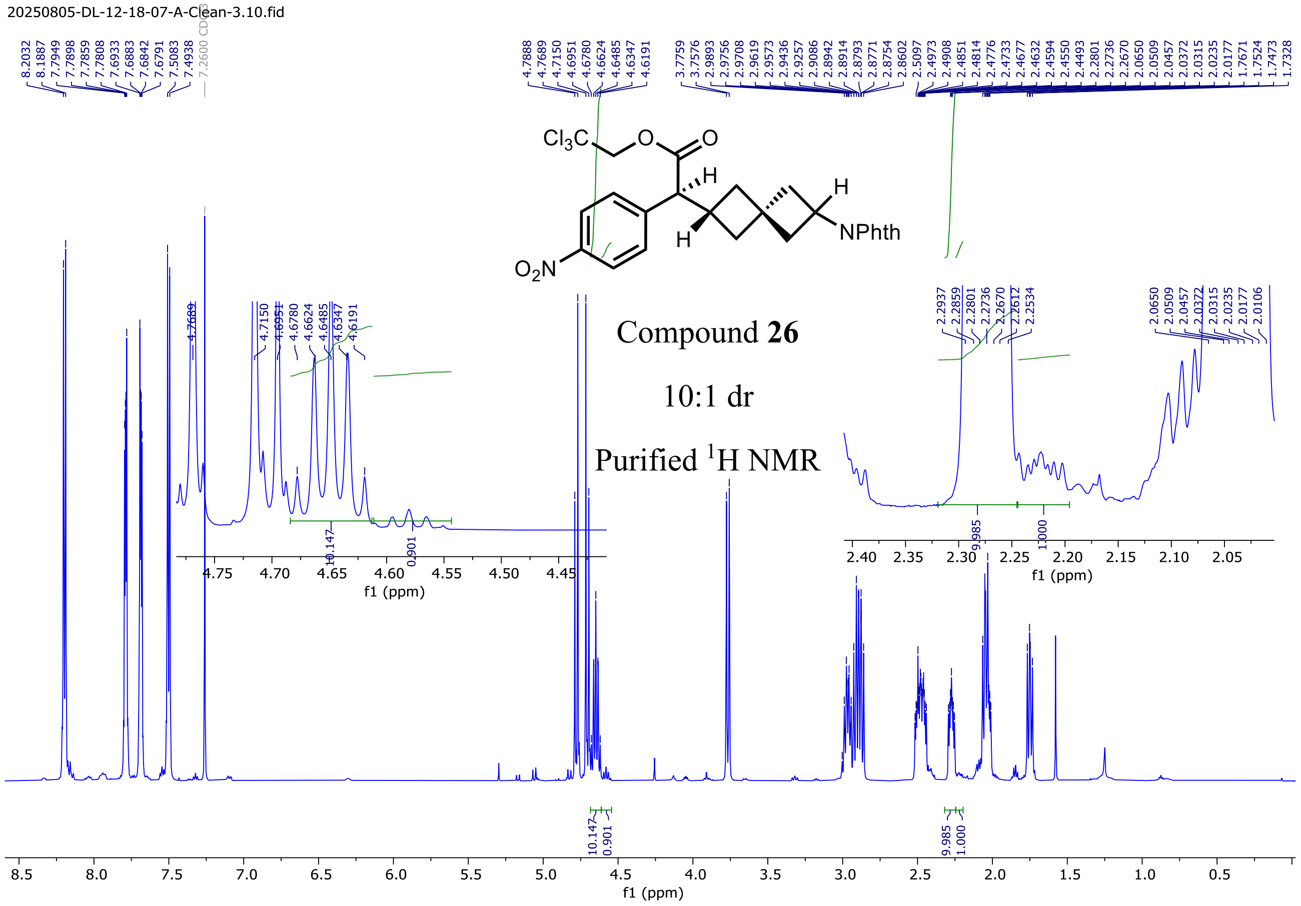
20250805-DL-12-18-07-A-Clean-3.10.fid
8.2032
8.1887
7.7949
7.7898
7.7859
7.7808
7.6933
7.6883
7.6842
7.6791
7.5083
7.4938
7.2600 CDCl3
4.7888
4.7689
4.7150
4.6951
4.6780
4.6624
4.6485
4.6347
4.6191
3.7759
3.7576
2.9893
2.9756
2.9708
2.9619
2.9573
2.9436
2.9257
2.9086
2.8942
2.8914
2.8793
2.8771
2.8754
2.8602
2.5097
2.4973
2.4908
2.4851
2.4814
2.4776
2.4733
2.4677
2.4632
2.4594
2.4550
2.4493
2.2801
2.2736
2.2670
2.0650
2.0509
2.0457
2.0372
2.0315
2.0235
2.0177
1.7671
1.7524
1.7473
1.7328
Cl_3C
O
O
H
H
H
NPhth
O_2N
Compound **26**
10:1 dr
Purified ^{1}H NMR
4.7689
4.7150
4.6951
4.6780
4.6624
4.6485
4.6347
4.6191
10.147
0.901
f1 (ppm)
2.2937
2.2859
2.2801
2.2736
2.2670
2.2612
2.2534
2.0650
2.0509
2.0457
2.0372
2.0315
2.0235
2.0177
2.0106
9.985
1.000
f1 (ppm)
10.147
0.901
9.985
1.000
f1 (ppm)

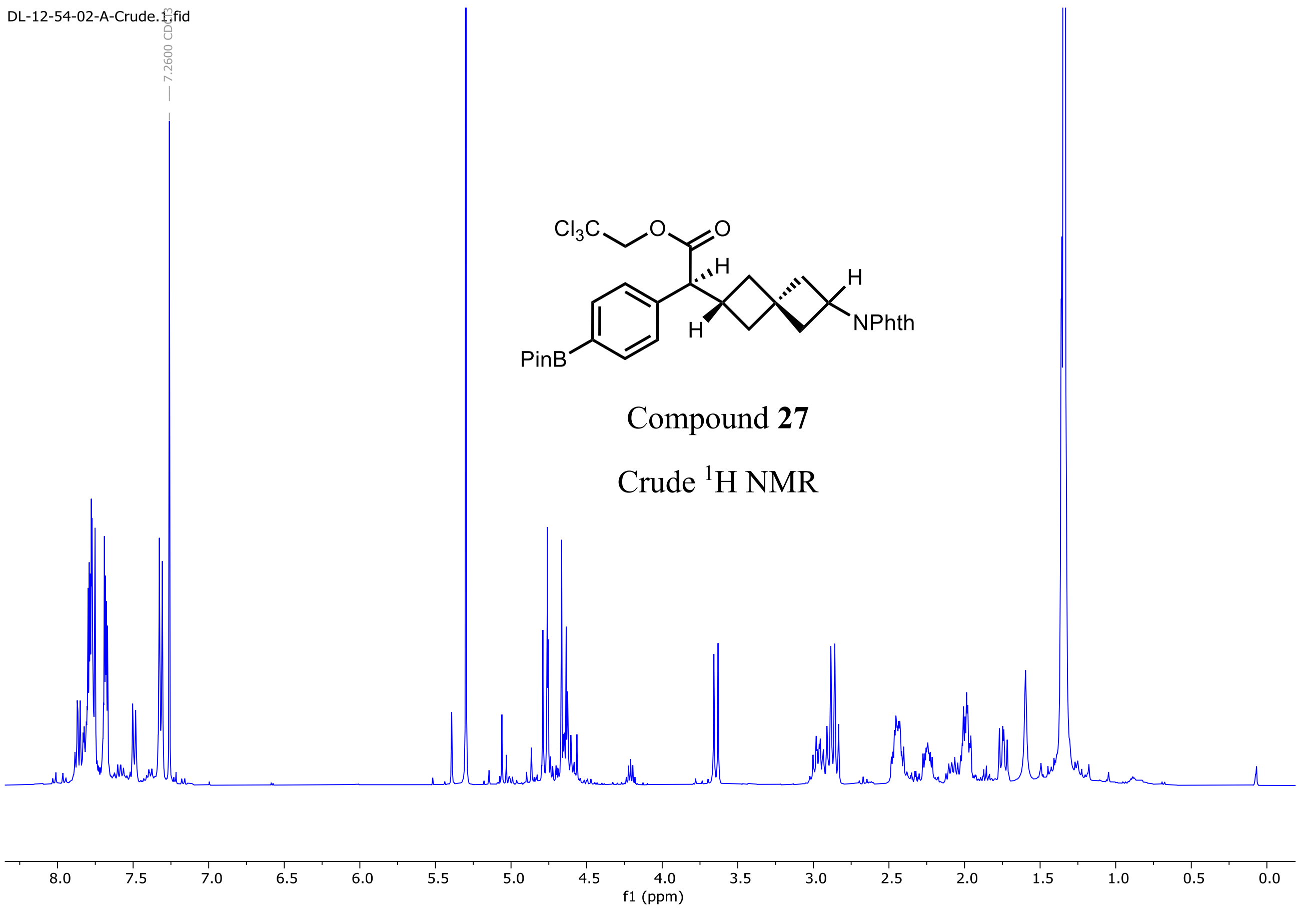
DL-12-54-02-A-Crude.1.fid
7.2600 CDCl3
Cl_3C
O
O
H
H
H
NPhth
PinB
Compound **27**
Crude ^{1}H NMR
f1 (ppm)

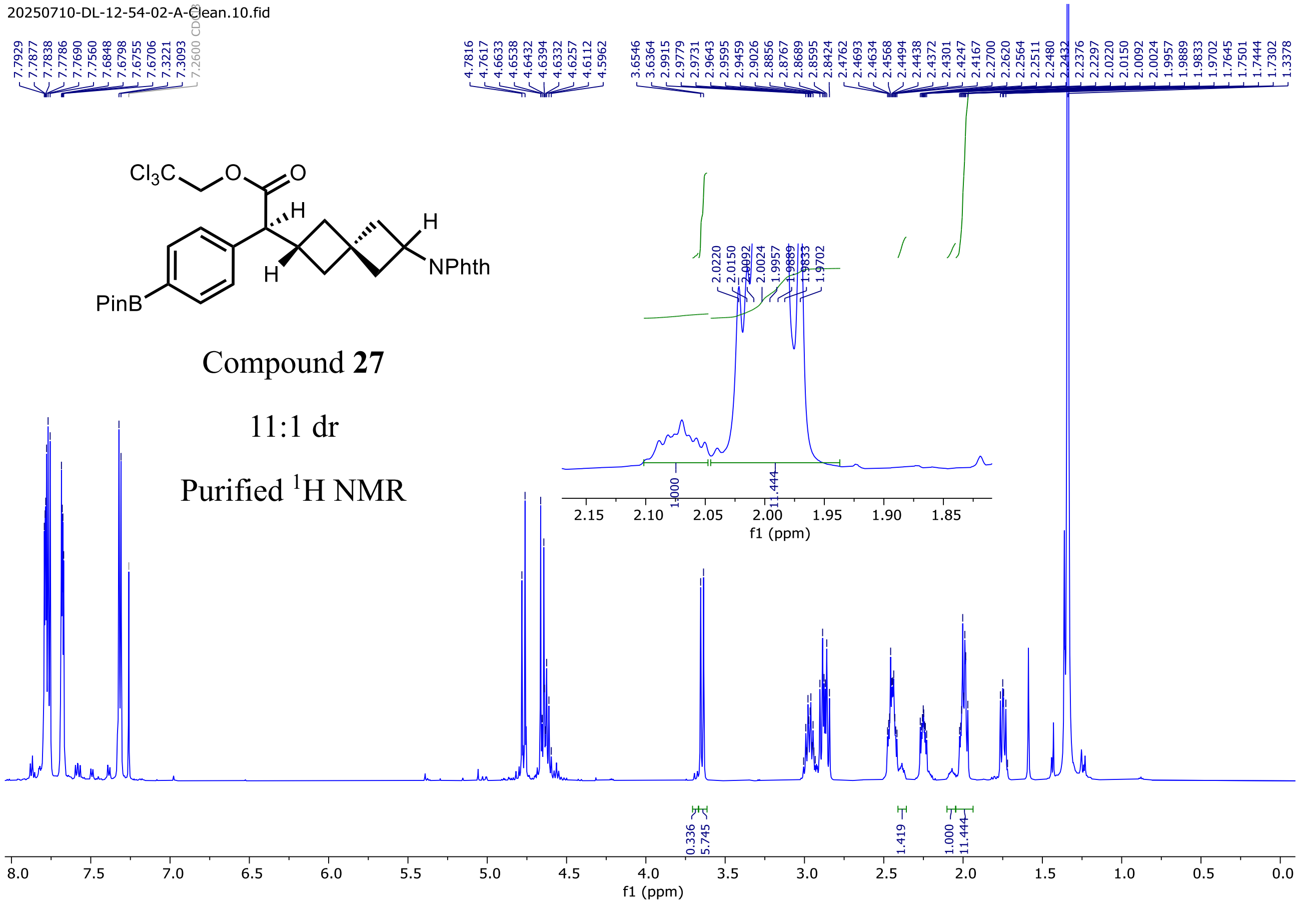
20250710-DL-12-54-02-A-Clean.10.fid
Cl_3C
O
O
H
H
H
NPhth
PinB
Compound **27**
11:1 dr
Purified ^{1}H NMR
1.000
11.444
0.336
5.745
1.419
f1 (ppm)

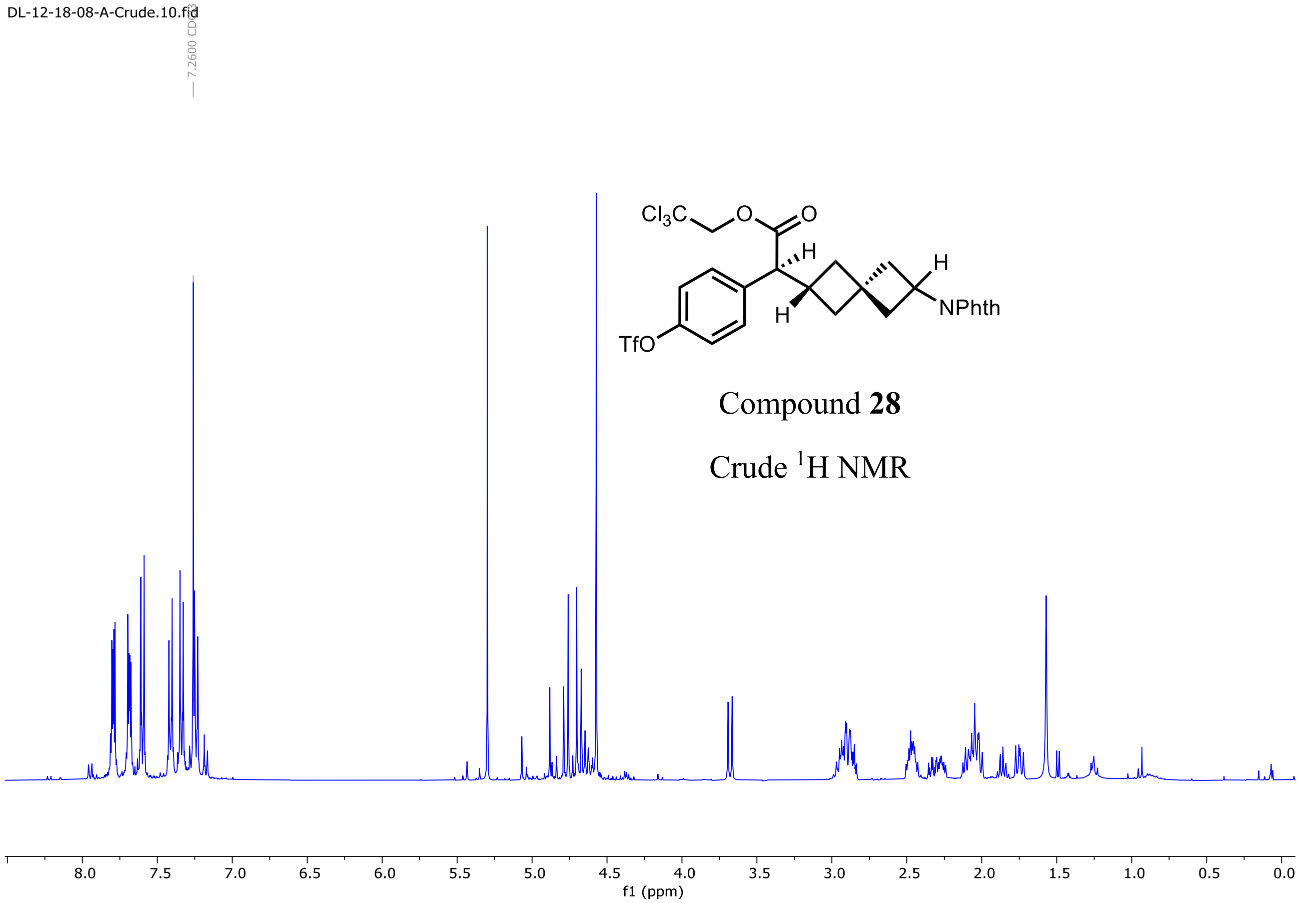

DL-12-18-08-A-Crude.10.fid
7.2600 CDCl3
Cl3C O O H H NPhth H TfO
Compound 28
Crude 1H NMR
f1 (ppm)

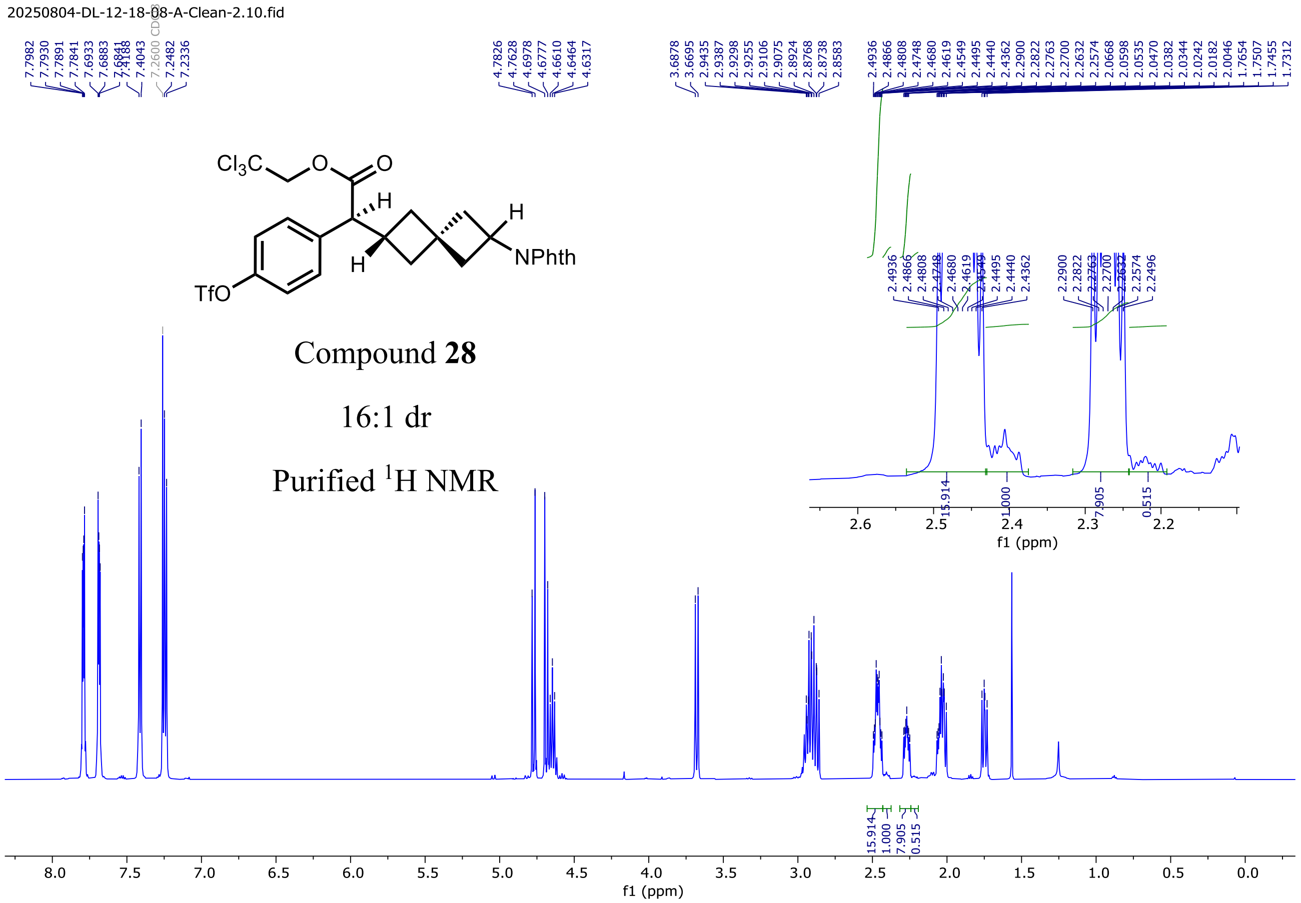

20250804-DL-12-18-08-A-Clean-2.10.fid
7.7982 7.7930 7.7891 7.7841 7.6933 7.6883 7.6841 7.4188 7.4043 7.2600 CDCl3 7.2482 7.2336
4.7826 4.7628 4.6978 4.6777 4.6610 4.6464 4.6317
3.6878 3.6695 2.9435 2.9387 2.9298 2.9255 2.9106 2.9075 2.8924 2.8768 2.8738 2.8583
2.4936 2.4866 2.4808 2.4748 2.4680 2.4619 2.4549 2.4495 2.4440 2.4362 2.2900 2.2822 2.2763 2.2700 2.2632 2.2574 2.0668 2.0598 2.0535 2.0470 2.0382 2.0344 2.0242 2.0182 2.0046 1.7654 1.7507 1.7455 1.7312
Cl3C O O H H NPhth H TfO
Compound 28
16:1 dr
Purified 1H NMR
15.914 1.000 7.905 0.515
f1 (ppm)

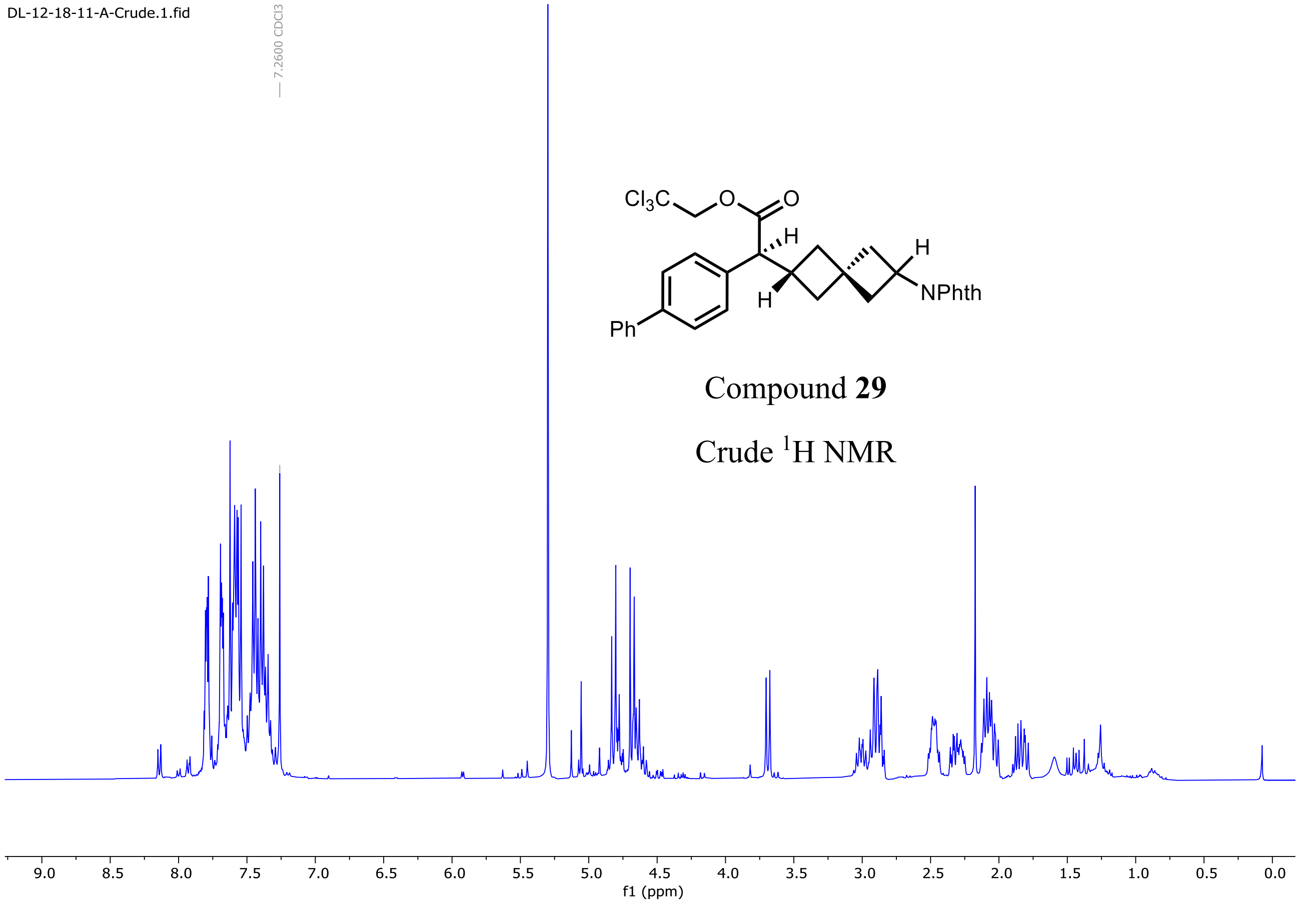
DL-12-18-11-A-Crude.1.fid
7.2600 CDCl3
Cl3C
O
O
H
H
H
NPhth
Ph
Compound 29
Crude 1H NMR
f1 (ppm)

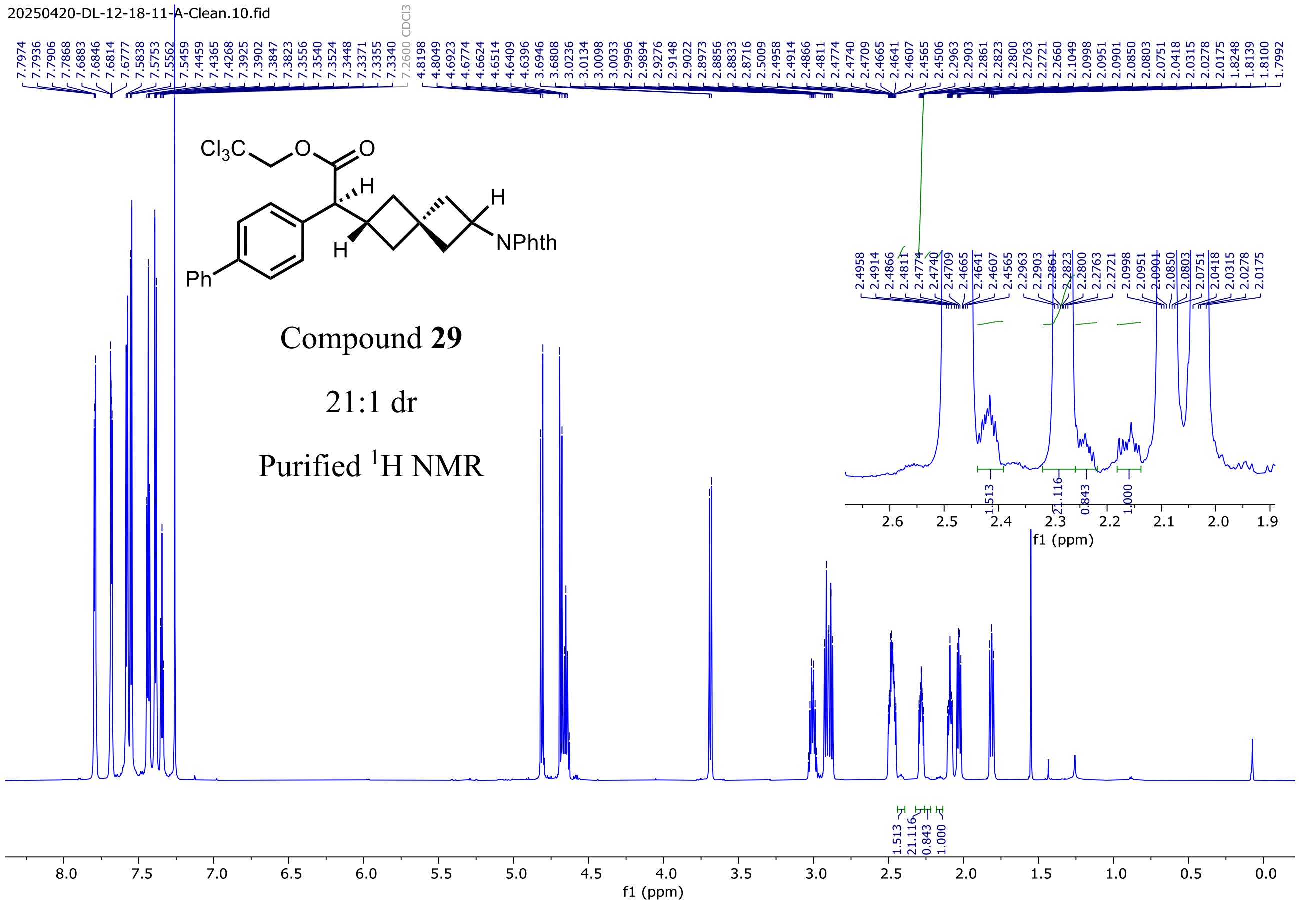
20250420-DL-12-18-11-A-Clean.10.fid
7.2600 CDCl3
Cl3C
O
O
H
H
H
NPhth
Ph
Compound 29
21:1 dr
Purified 1H NMR
1.513
21.116
0.843
1.000
f1 (ppm)

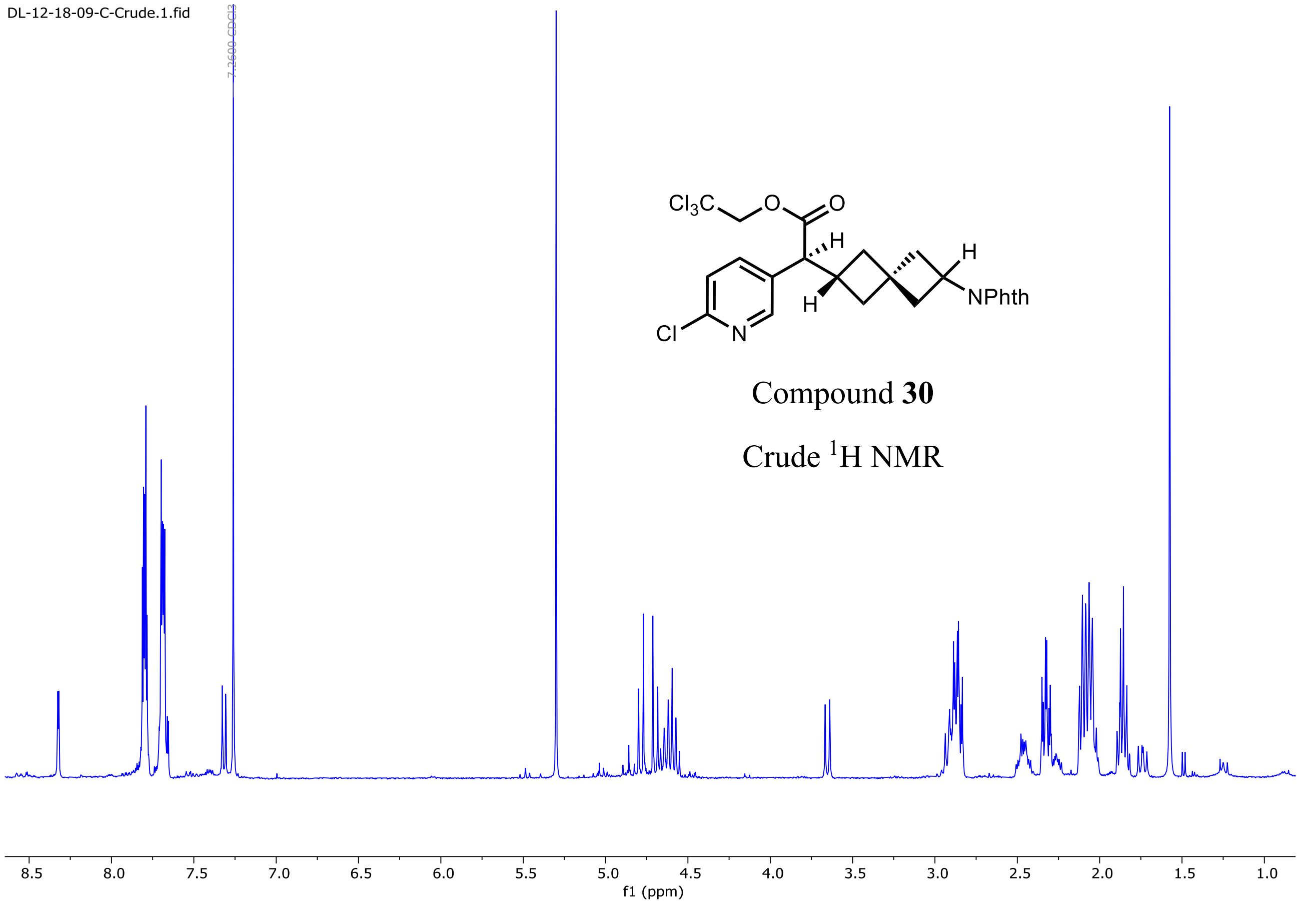
DL-12-18-09-C-Crude.1.fid
Compound **30**
Crude ¹H NMR
f1 (ppm)

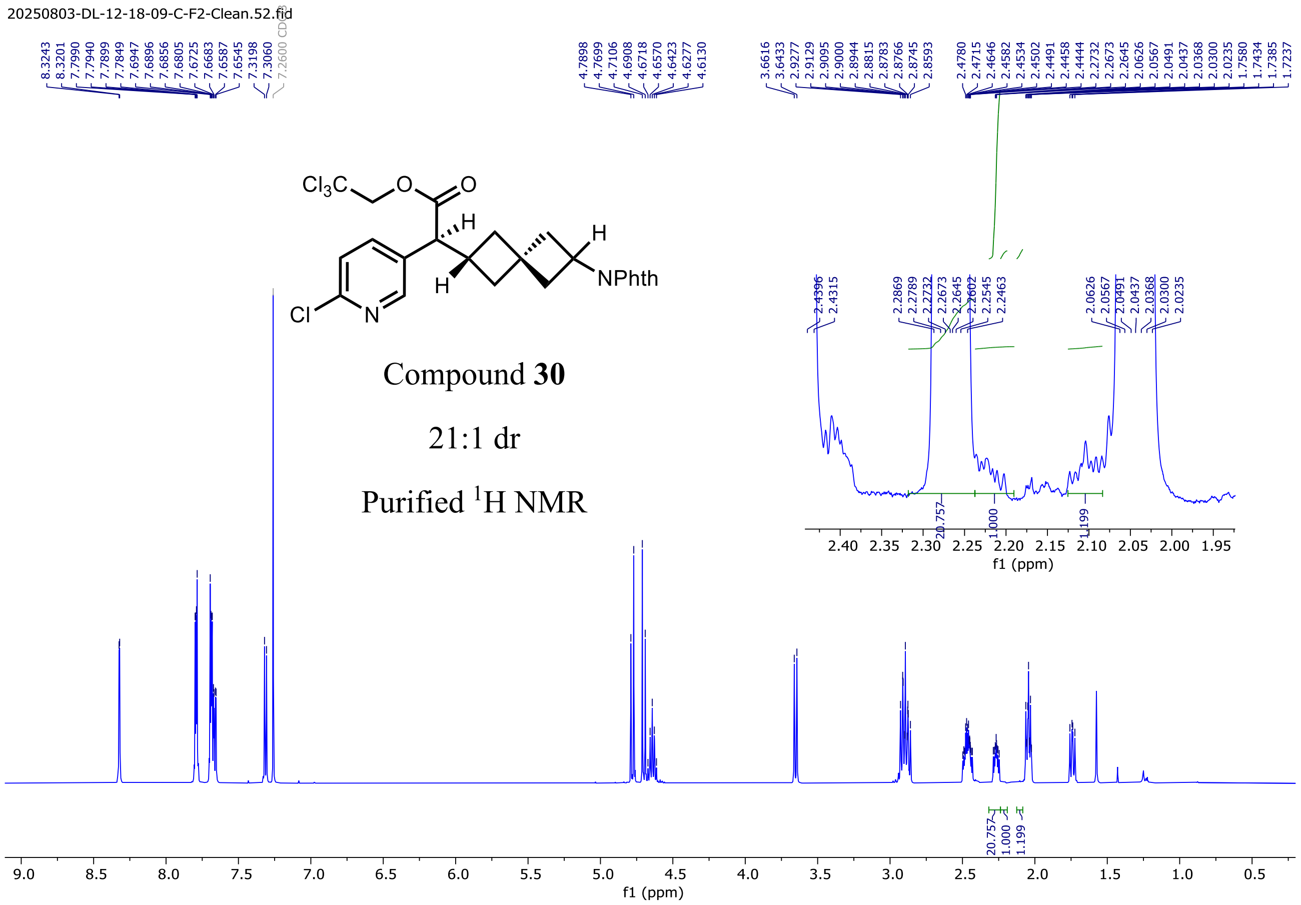
20250803-DL-12-18-09-C-F2-Clean.52.fid
Compound **30**
21:1 dr
Purified ¹H NMR
f1 (ppm)

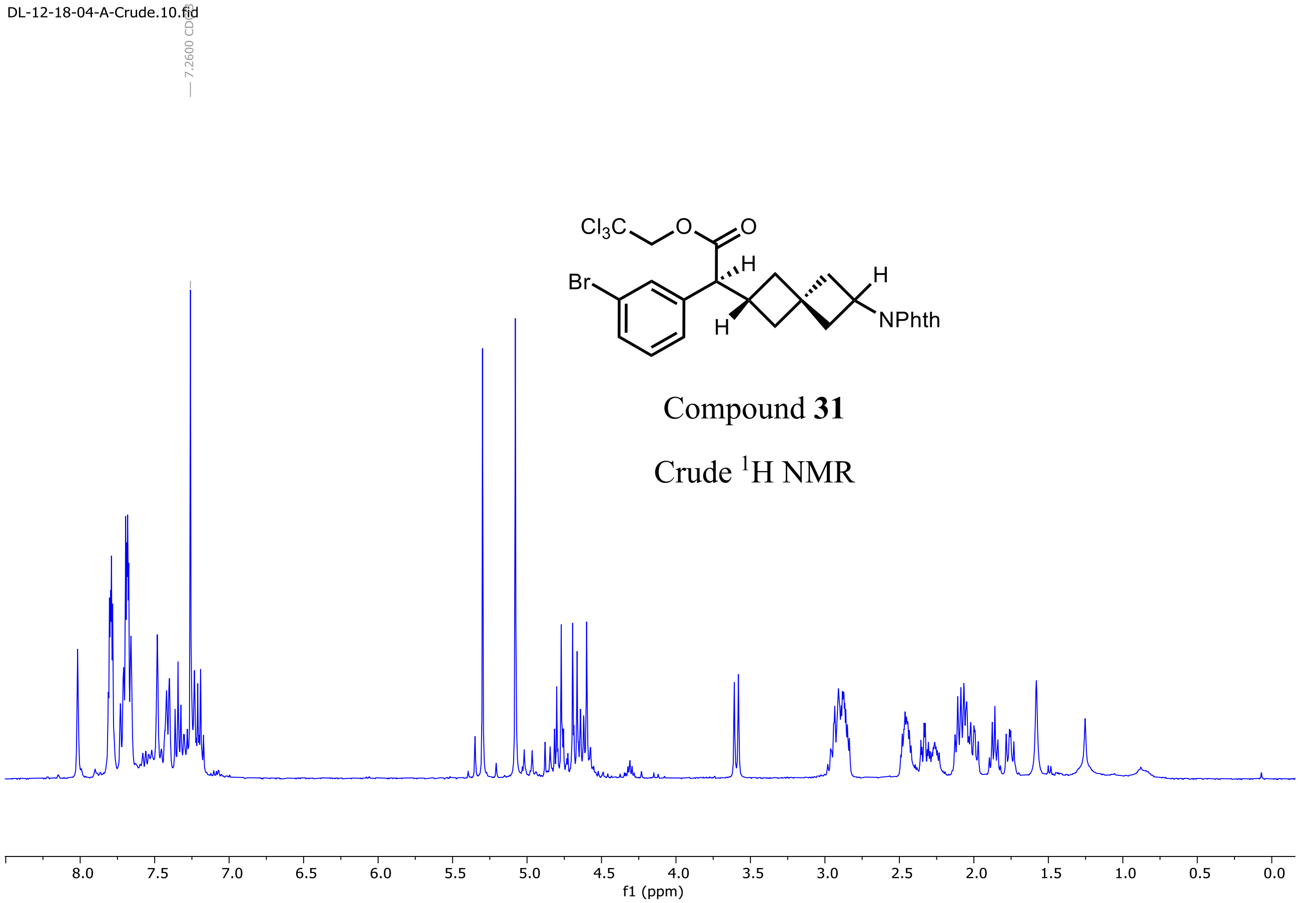
DL-12-18-04-A-Crude.10.fid
7.2600 CDCl3
Compound 31
Crude 1H NMR
f1 (ppm)

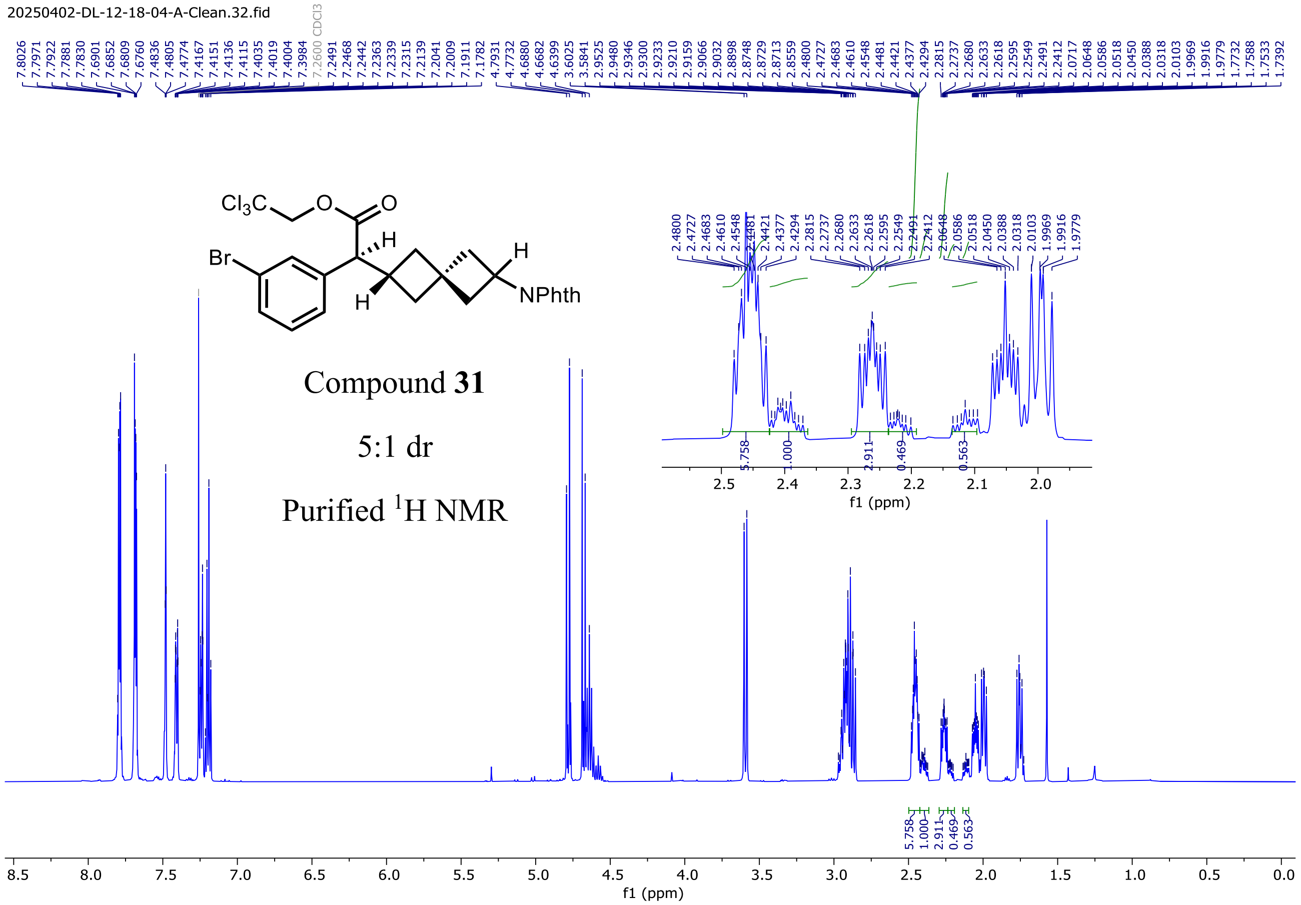
20250402-DL-12-18-04-A-Clean.32.fid
Compound 31
5:1 dr
Purified 1H NMR
f1 (ppm)

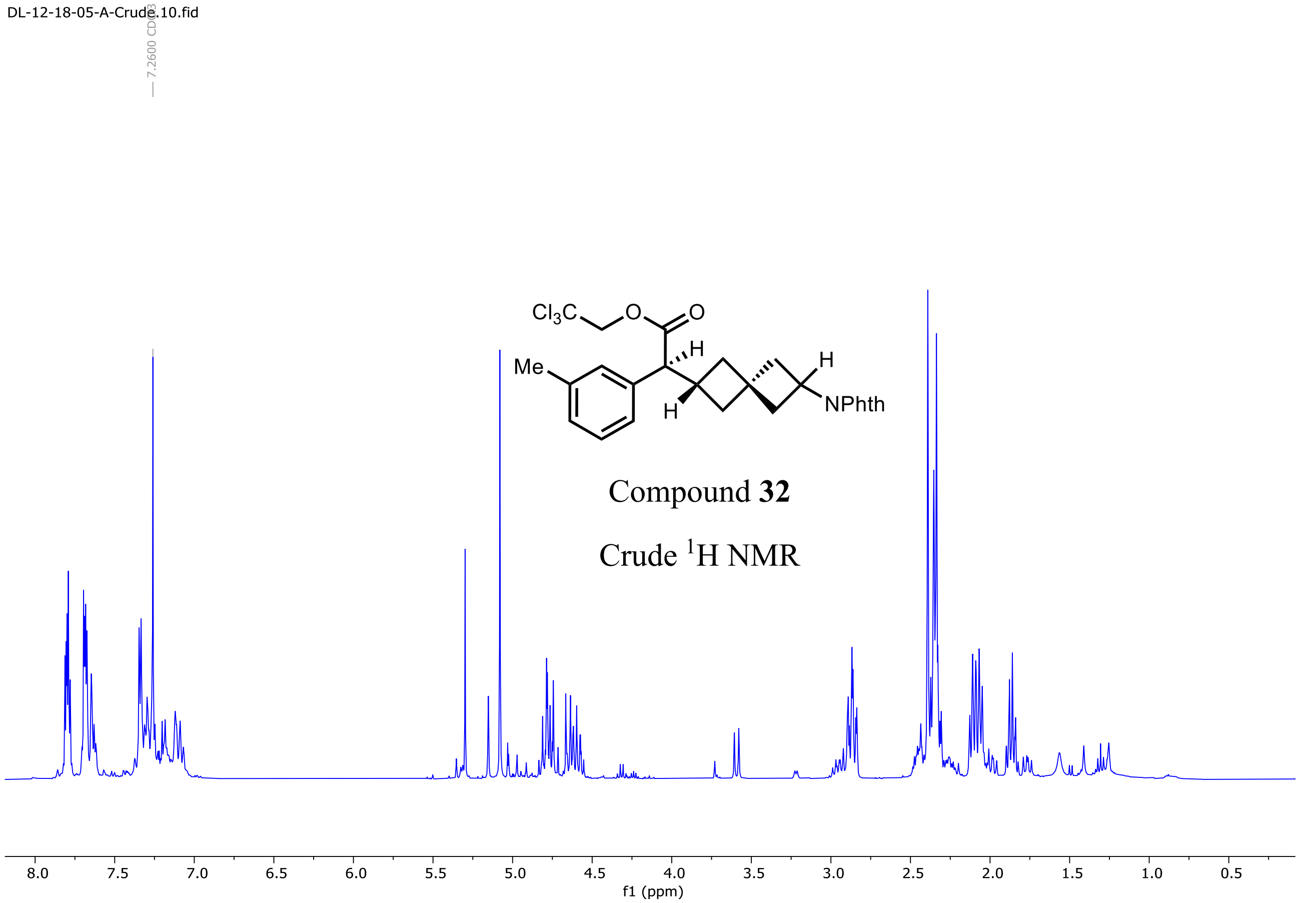

Compound **32**

Crude ^{1}H NMR

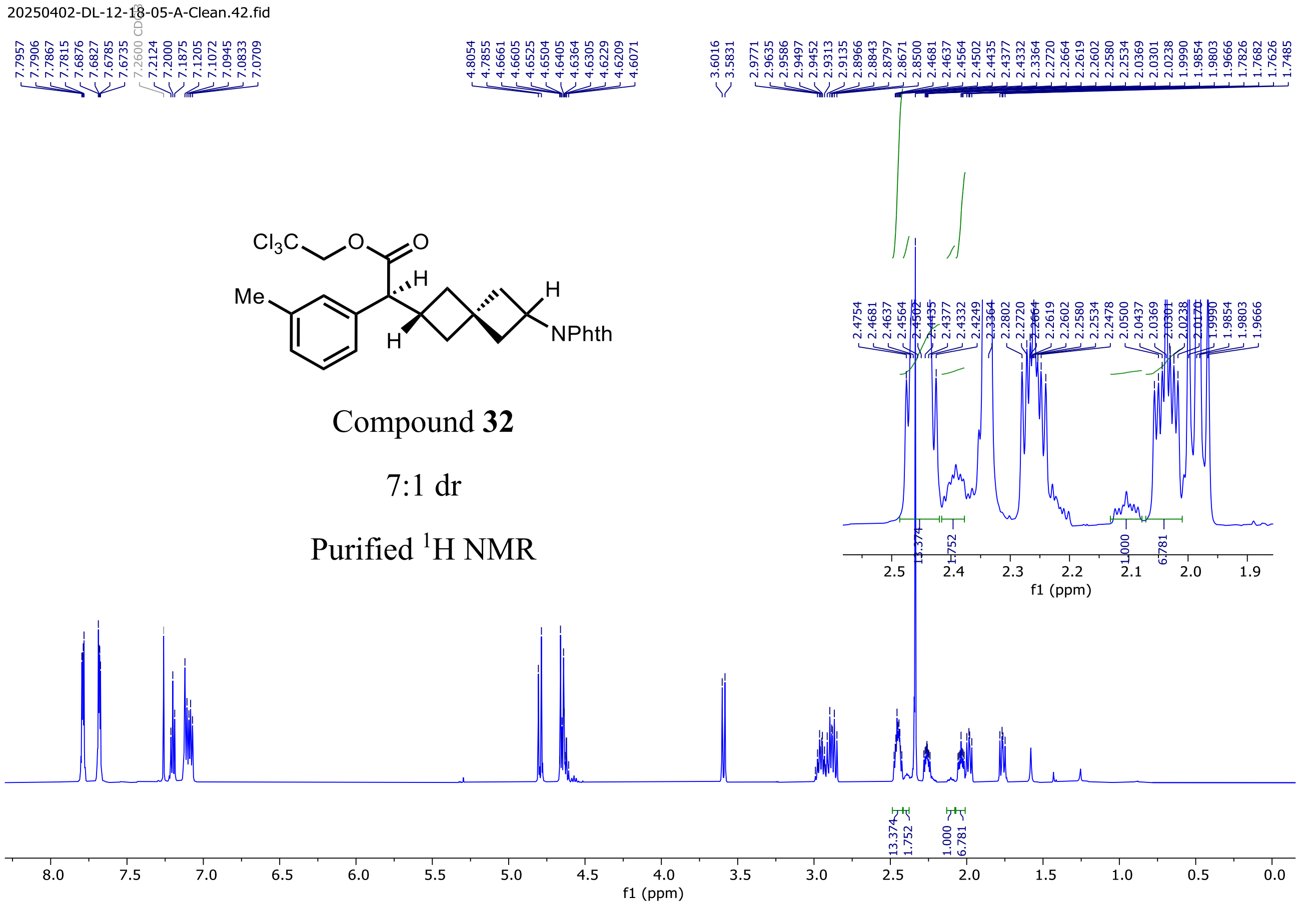

Compound **32**

7:1 dr

Purified ^{1}H NMR

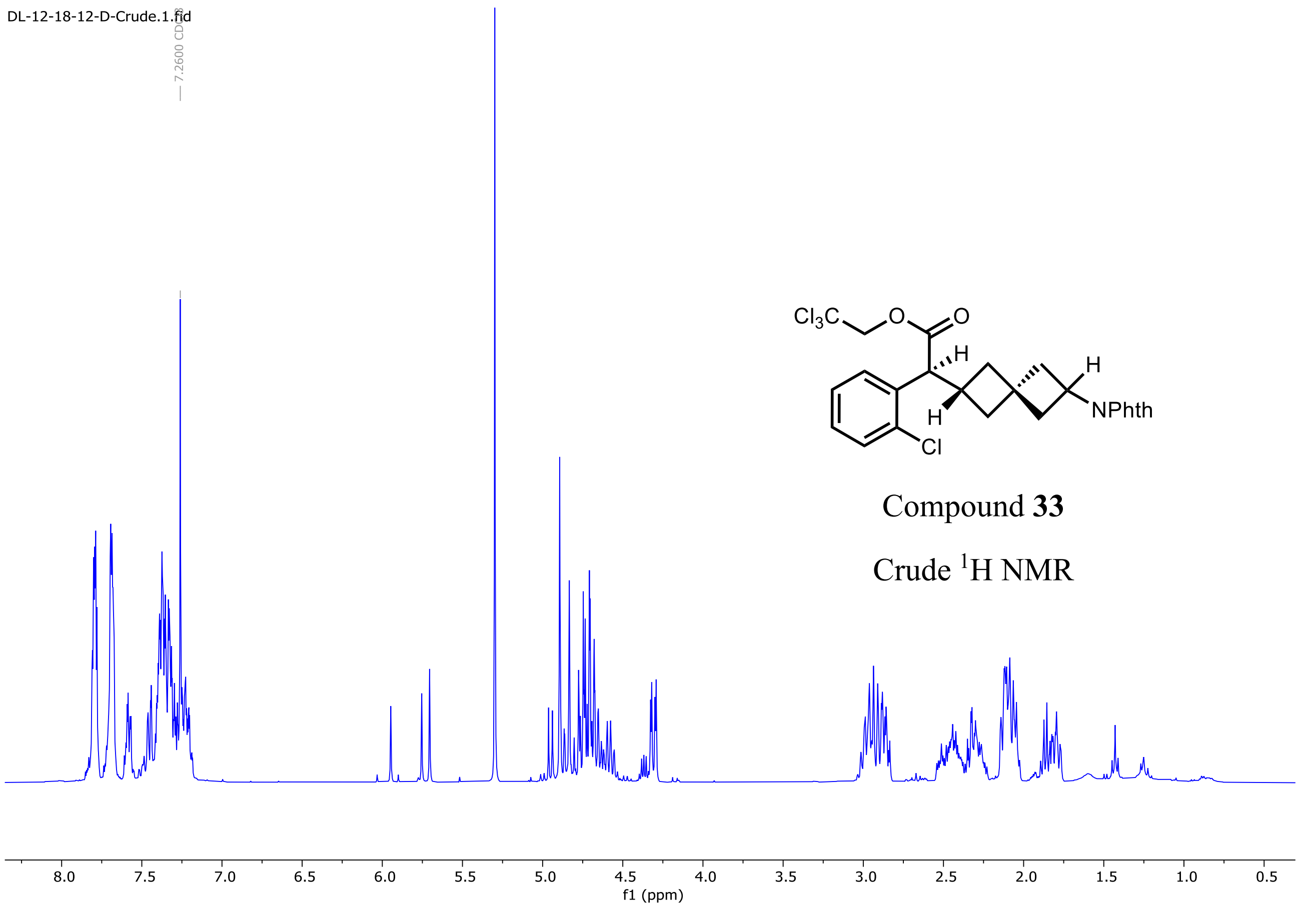
DL-12-18-12-D-Crude.1.fid
Compound **33**
Crude ^{1}H NMR
NPhth
Cl
f1 (ppm)

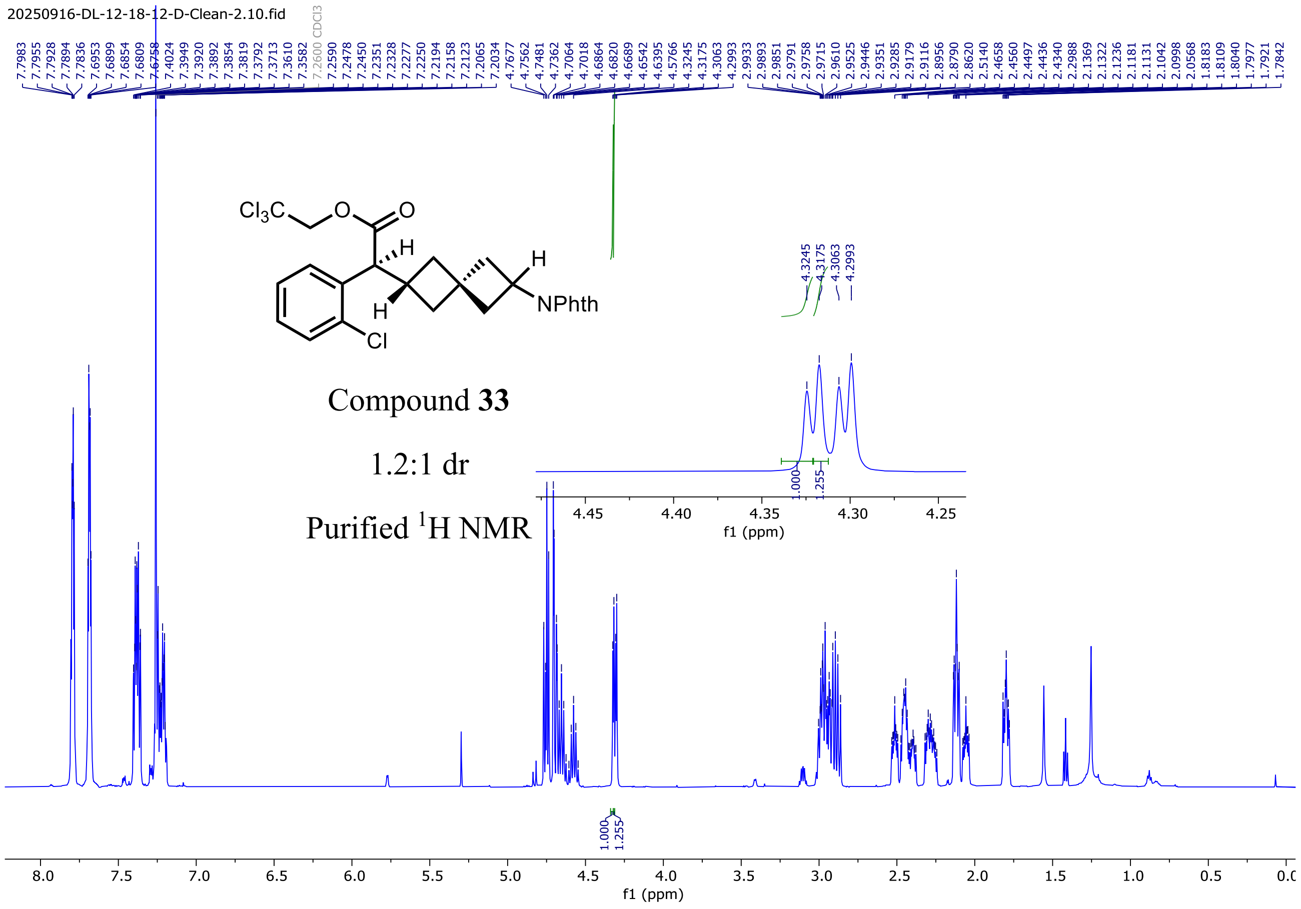
20250916-DL-12-18-12-D-Clean-2.10.fid
Compound **33**
1.2:1 dr
Purified ^{1}H NMR
NPhth
Cl
1.000
1.255
f1 (ppm)

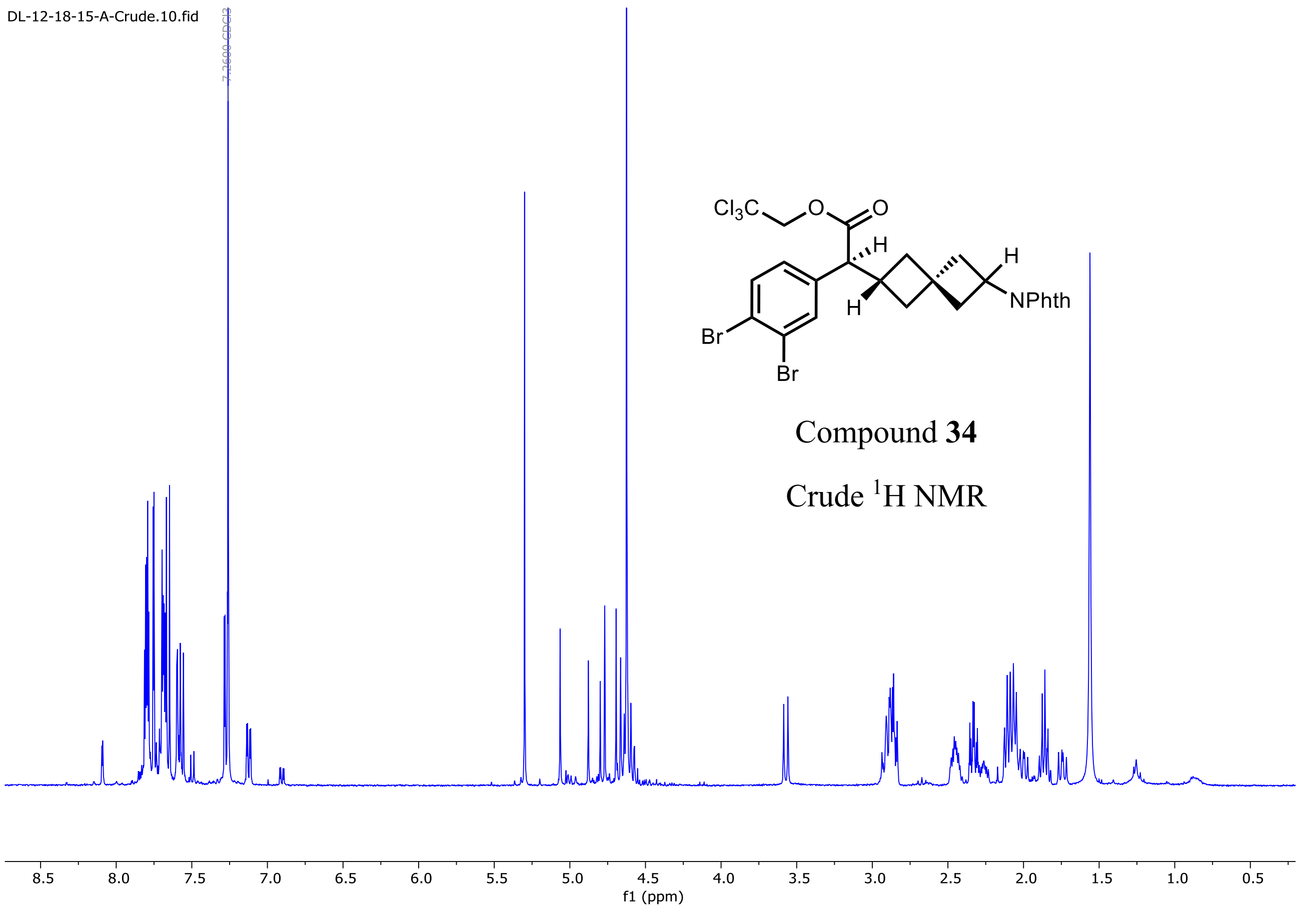
DL-12-18-15-A-Crude.10.fid
Compound 34
Crude ^{1}H NMR
NPhth
f1 (ppm)

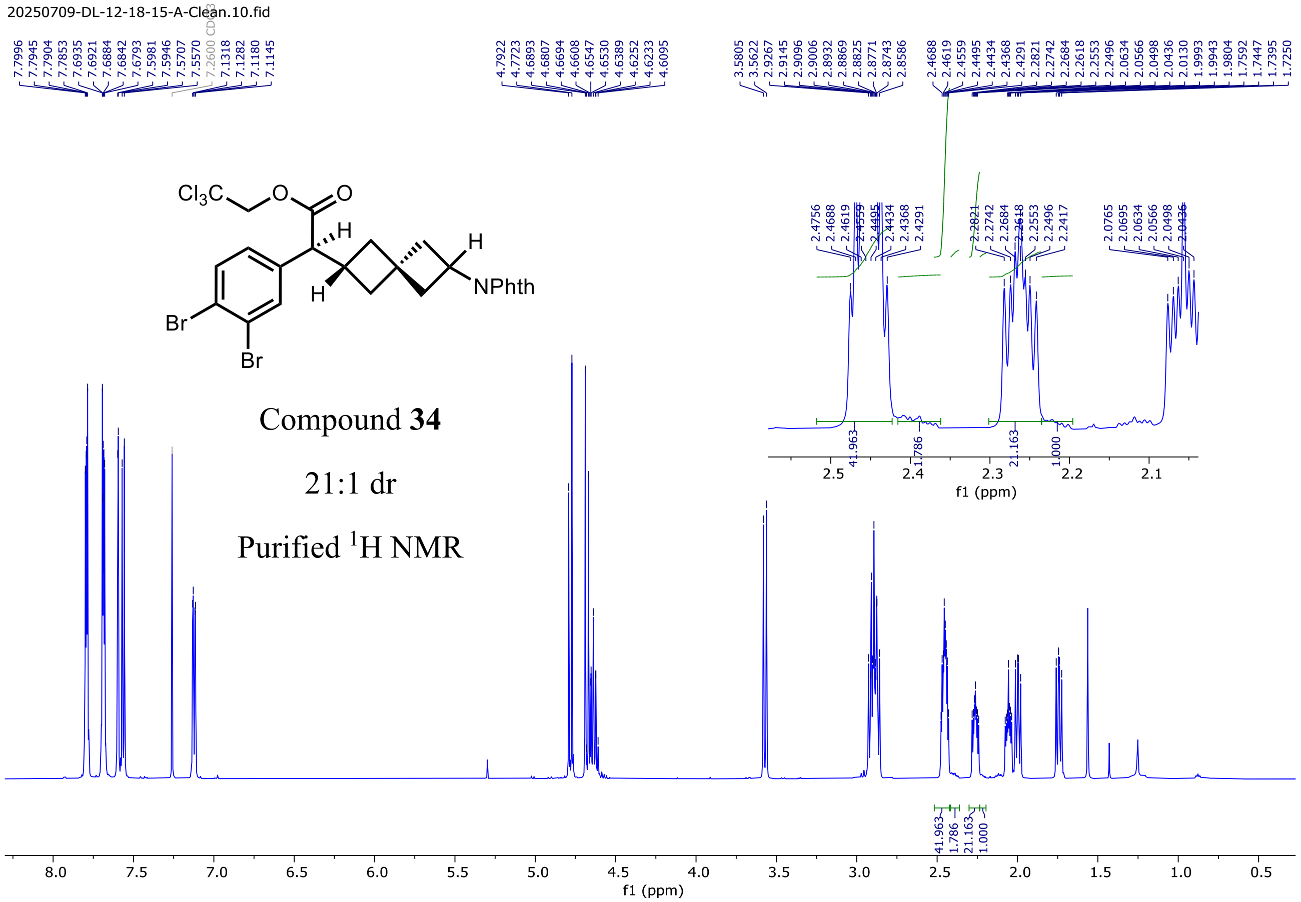
20250709-DL-12-18-15-A-Clean.10.fid
Compound 34
21:1 dr
Purified ^{1}H NMR
NPhth
f1 (ppm)

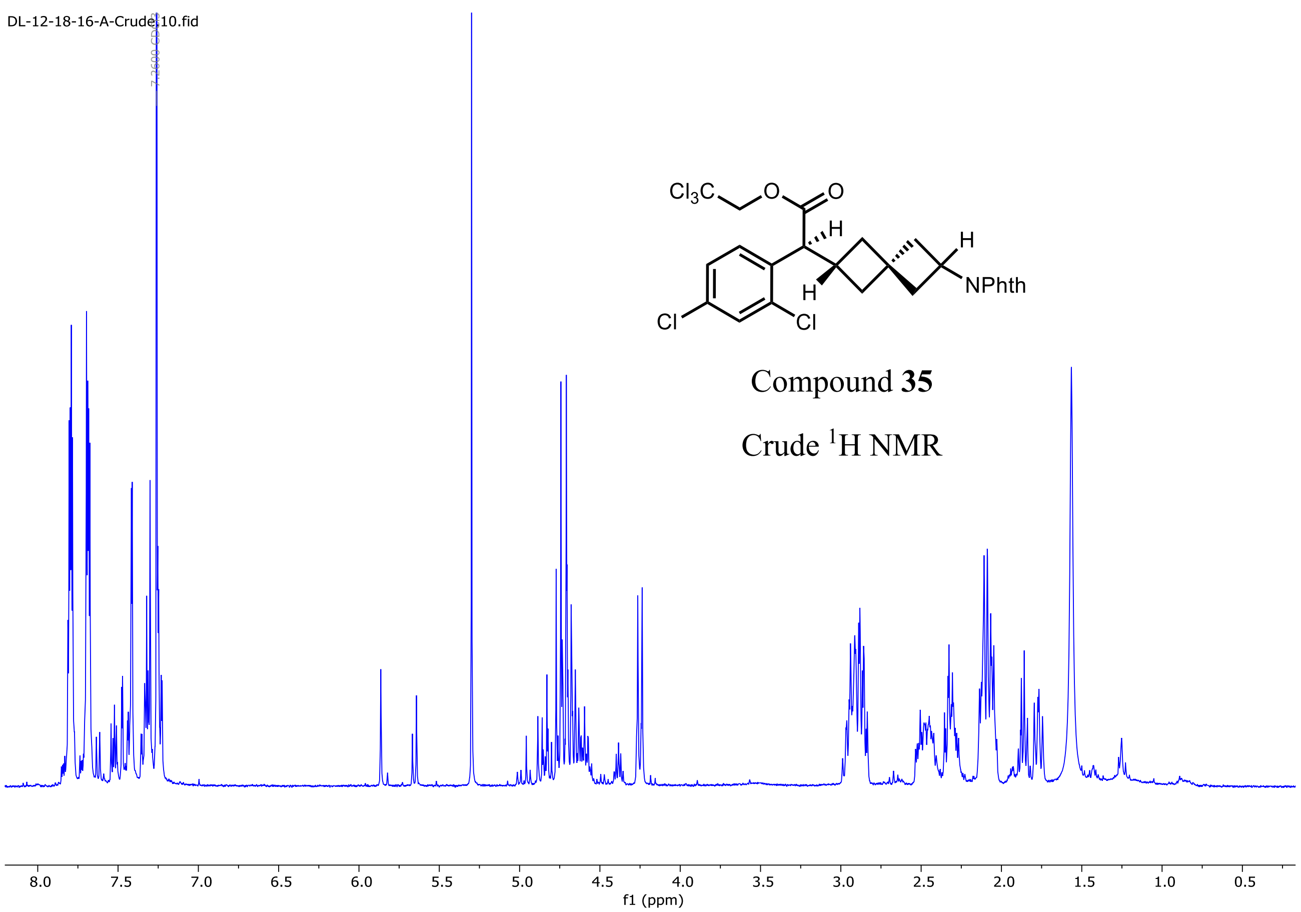
DL-12-18-16-A-Crude.10.fid
Cl3C
O
O
H
H
H
NPhth
Cl
Cl
Compound 35
Crude 1H NMR
f1 (ppm)

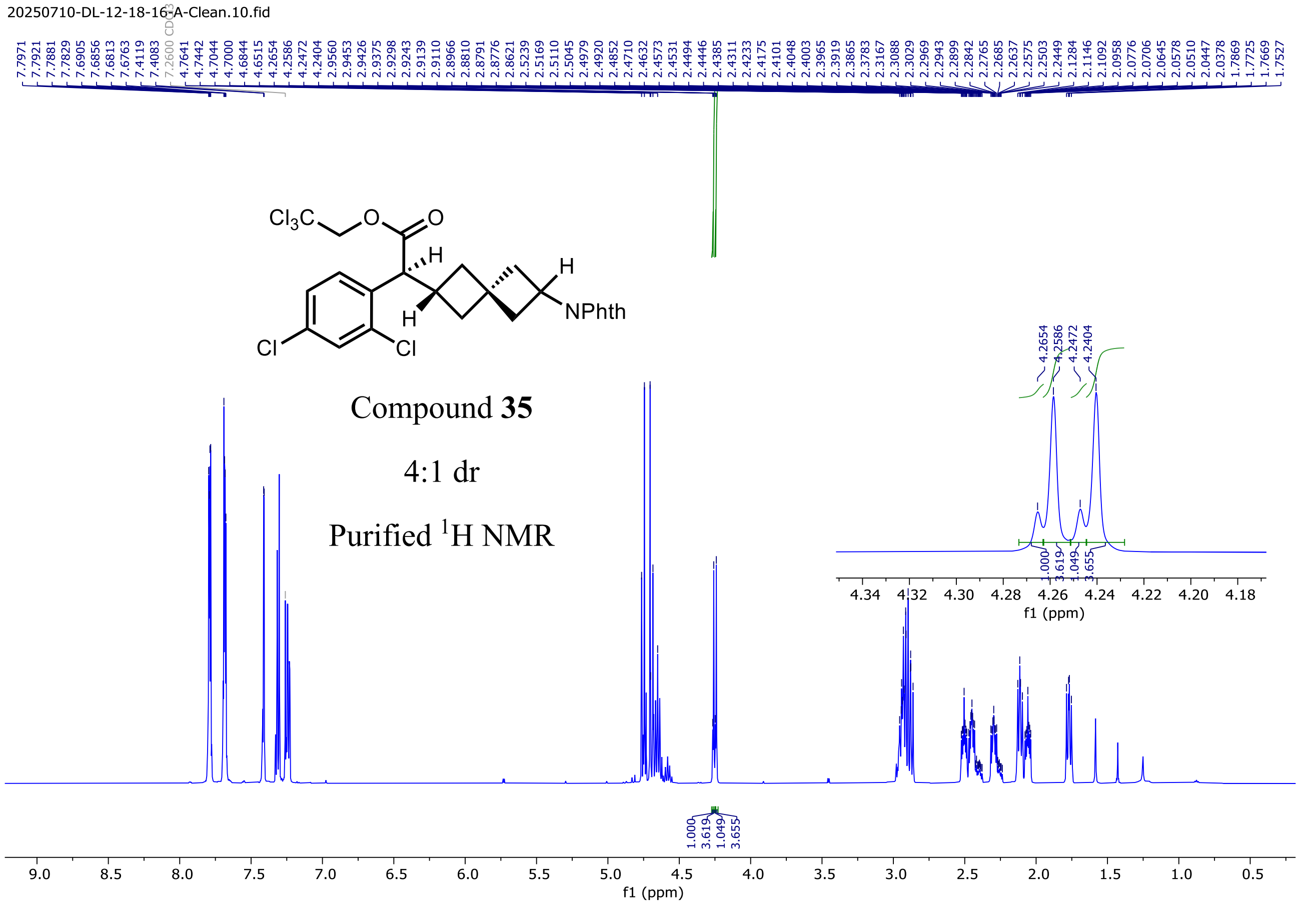
20250710-DL-12-18-16-A-Clean.10.fid
Cl3C
O
O
H
H
H
NPhth
Cl
Cl
Compound 35
4:1 dr
Purified 1H NMR
4.2654
4.2586
4.2472
4.2404
1.000
3.619
1.049
3.655
f1 (ppm)

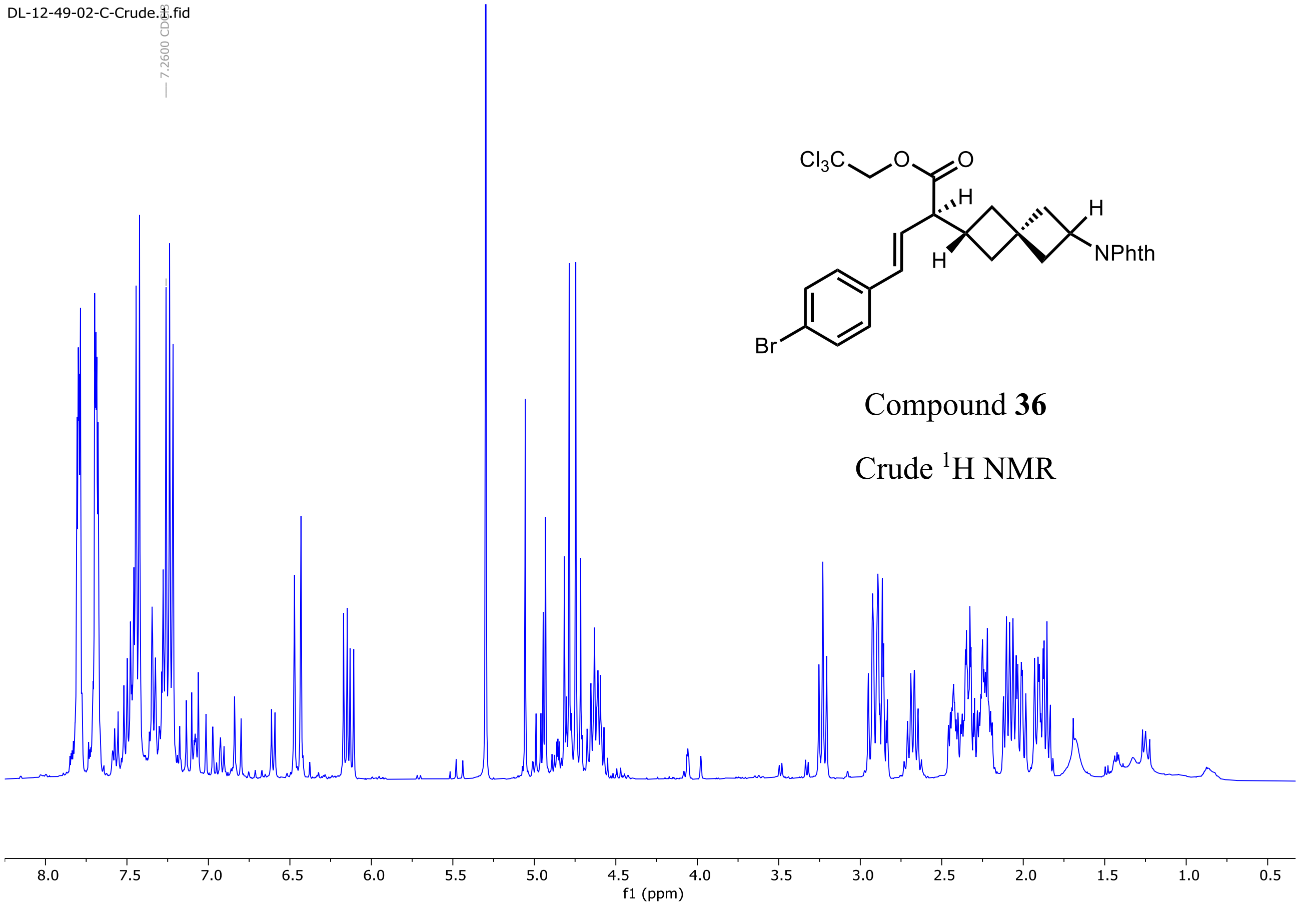
DL-12-49-02-C-Crude.1.fid
7.2600 CDCl3
Cl_3C
O
O
H
H
H
NPhth
Br
Compound **36**
Crude ^{1}H NMR
f1 (ppm)
8.0 7.5 7.0 6.5 6.0 5.5 5.0 4.5 4.0 3.5 3.0 2.5 2.0 1.5 1.0 0.5

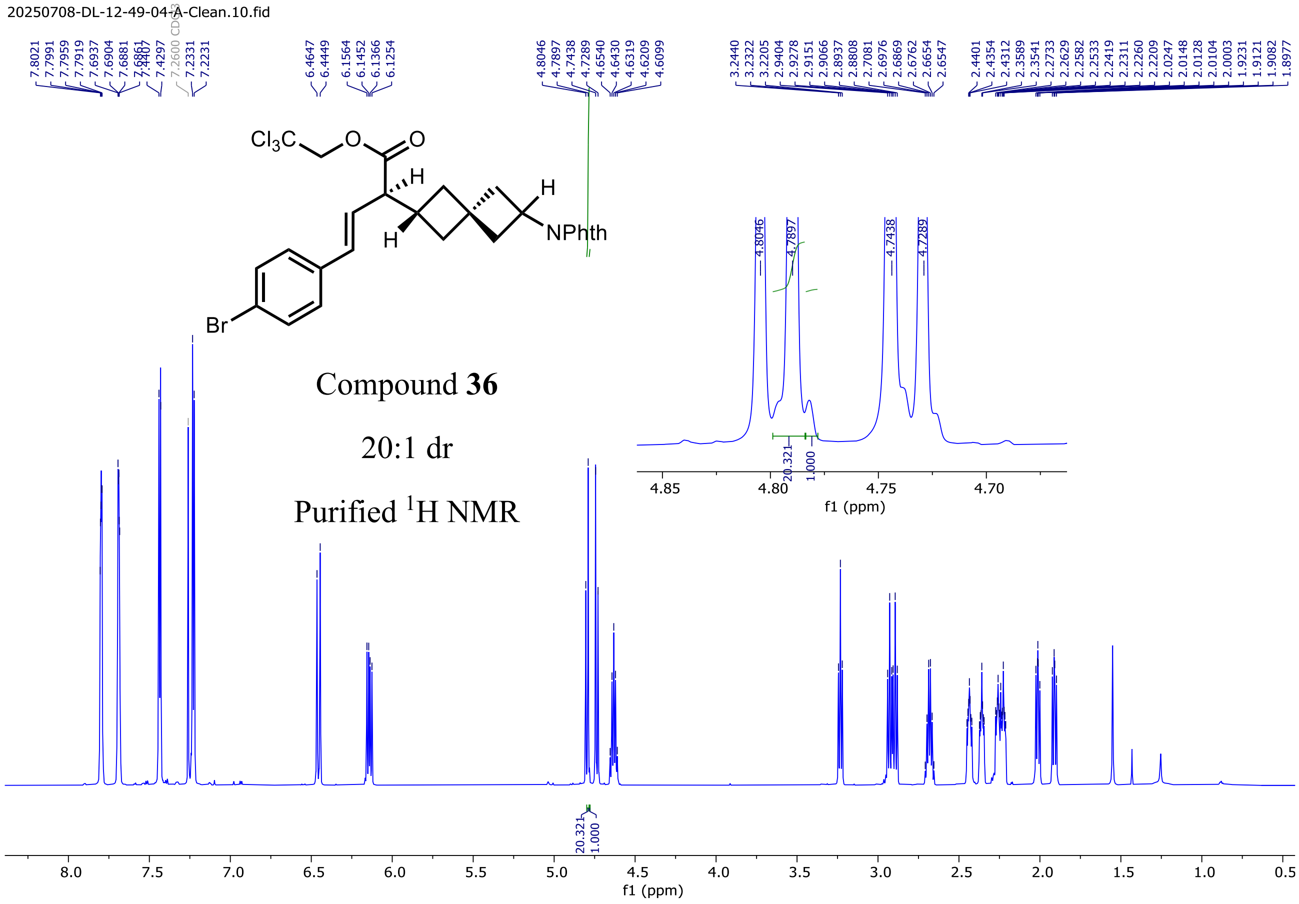
20250708-DL-12-49-04-A-Clean.10.fid
7.8021 7.7991 7.7959 7.7919 7.6937 7.6904 7.6881 7.6861 7.4407 7.4297 7.2600 CDCl3 7.2331 7.2231
6.4647 6.4449 6.1564 6.1452 6.1366 6.1254
4.8046 4.7897 4.7438 4.7289 4.6540 4.6430 4.6319 4.6209 4.6099
3.2440 3.2322 3.2205 2.9404 2.9278 2.9151 2.9066 2.8937 2.8808 2.7081 2.6976 2.6869 2.6762 2.6654 2.6547
2.4401 2.4354 2.4312 2.3589 2.3541 2.2733 2.2629 2.2582 2.2533 2.2419 2.2311 2.2260 2.2209 2.0247 2.0148 2.0128 2.0104 2.0003 1.9231 1.9121 1.9082 1.8977
Cl_3C
O
O
H
H
H
NPhth
Br
Compound **36**
20:1 dr
Purified ^{1}H NMR
4.8046 4.7897 4.7438 4.7289
20.321 1.000
4.85 4.80 4.75 4.70
f1 (ppm)
20.321 1.000
8.0 7.5 7.0 6.5 6.0 5.5 5.0 4.5 4.0 3.5 3.0 2.5 2.0 1.5 1.0 0.5
f1 (ppm)

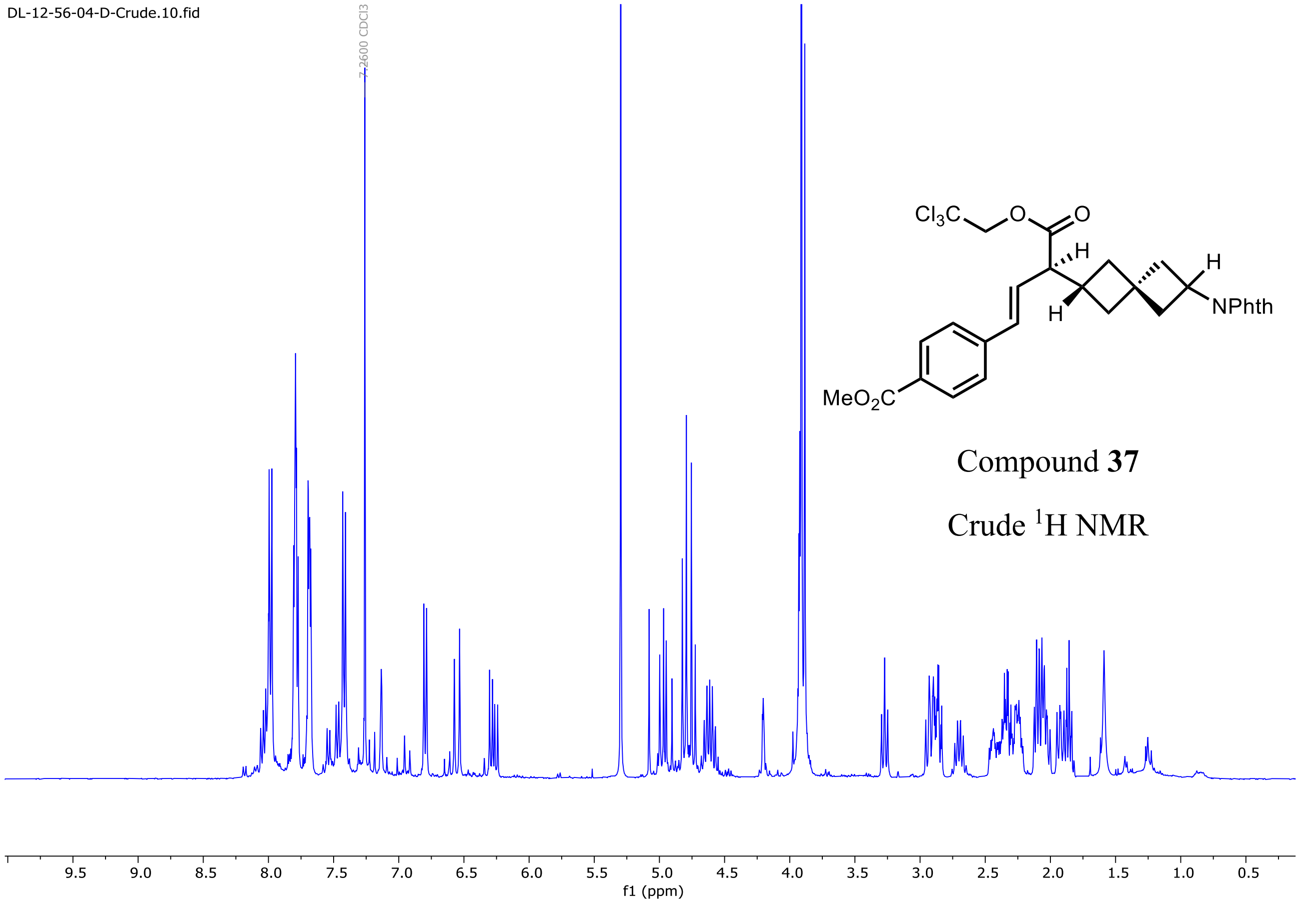

Compound **37**

Crude ^{1}H NMR

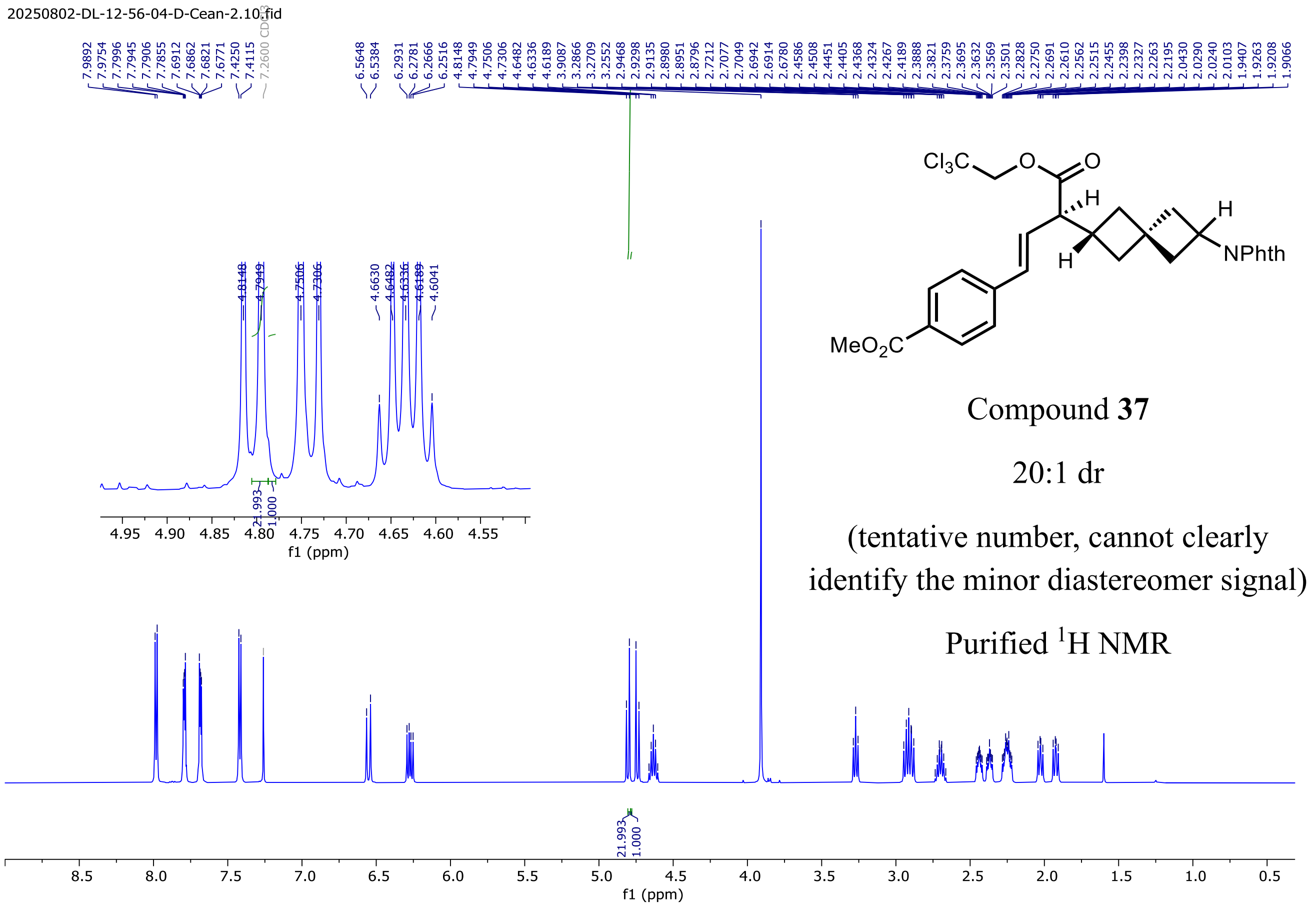

Compound **37**

20:1 dr

(tentative number, cannot clearly identify the minor diastereomer signal)

Purified ^{1}H NMR

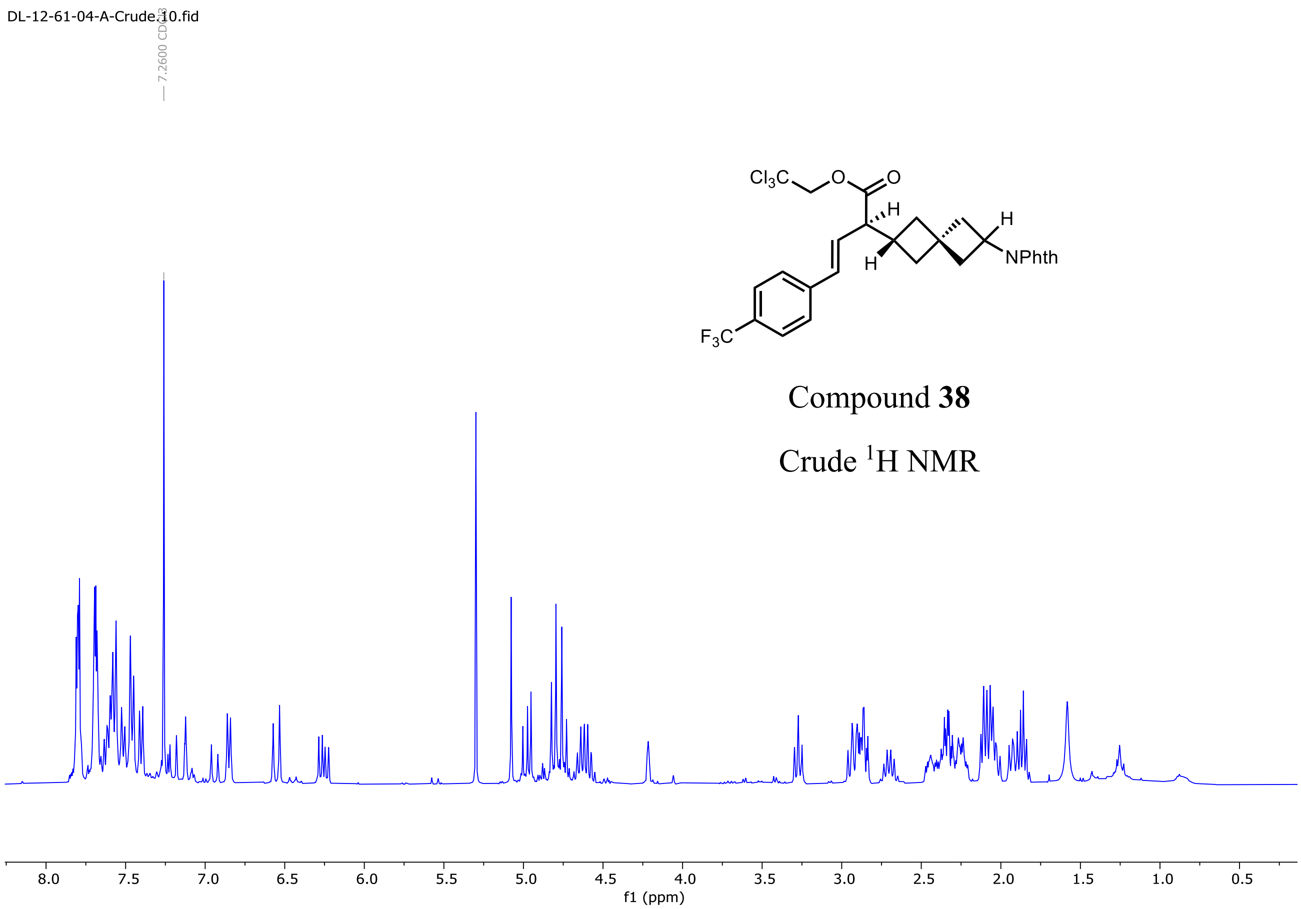

Compound **38**

Crude ^{1}H NMR

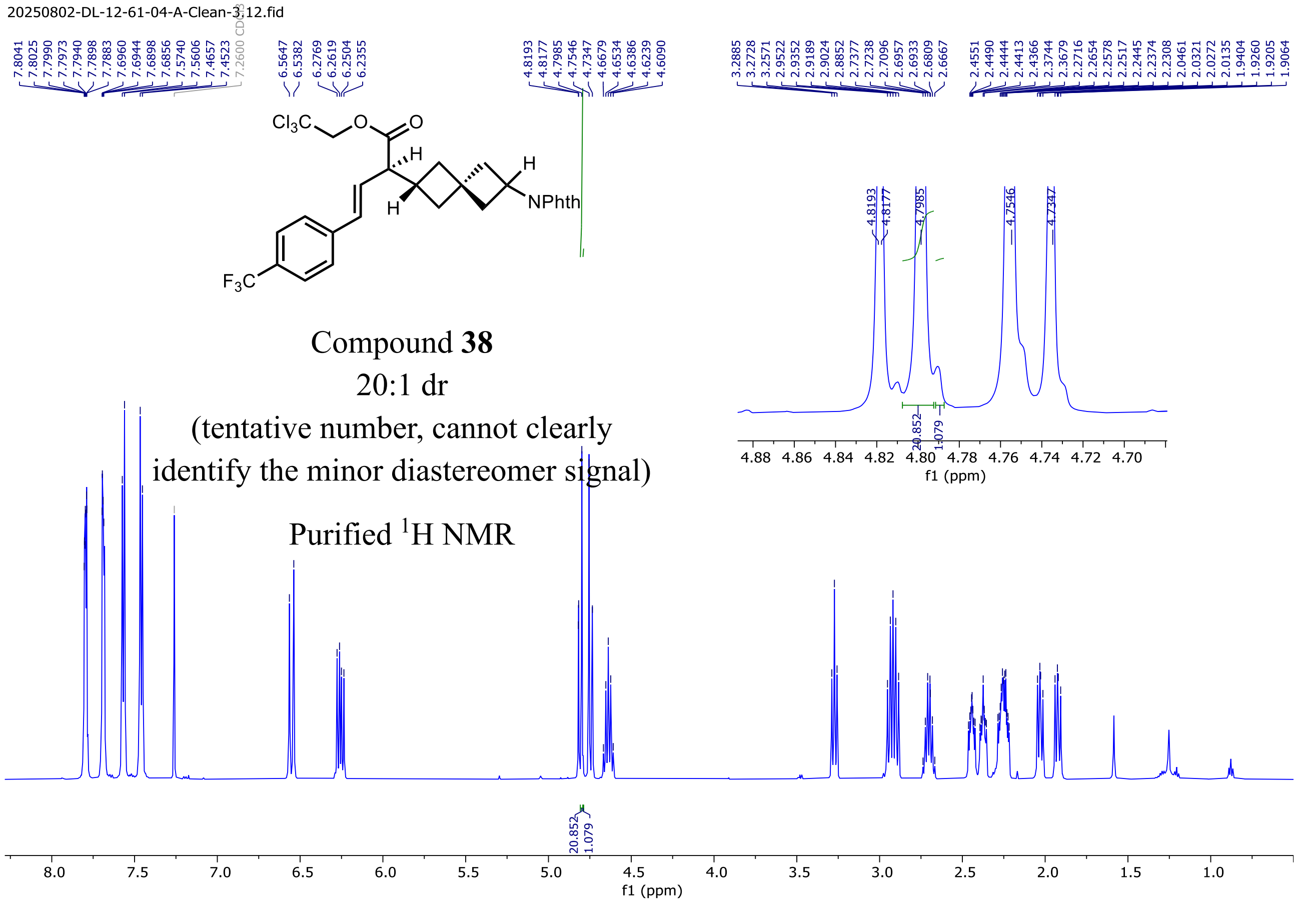

Compound **38**

20:1 dr

(tentative number, cannot clearly identify the minor diastereomer signal)

Purified ^{1}H NMR

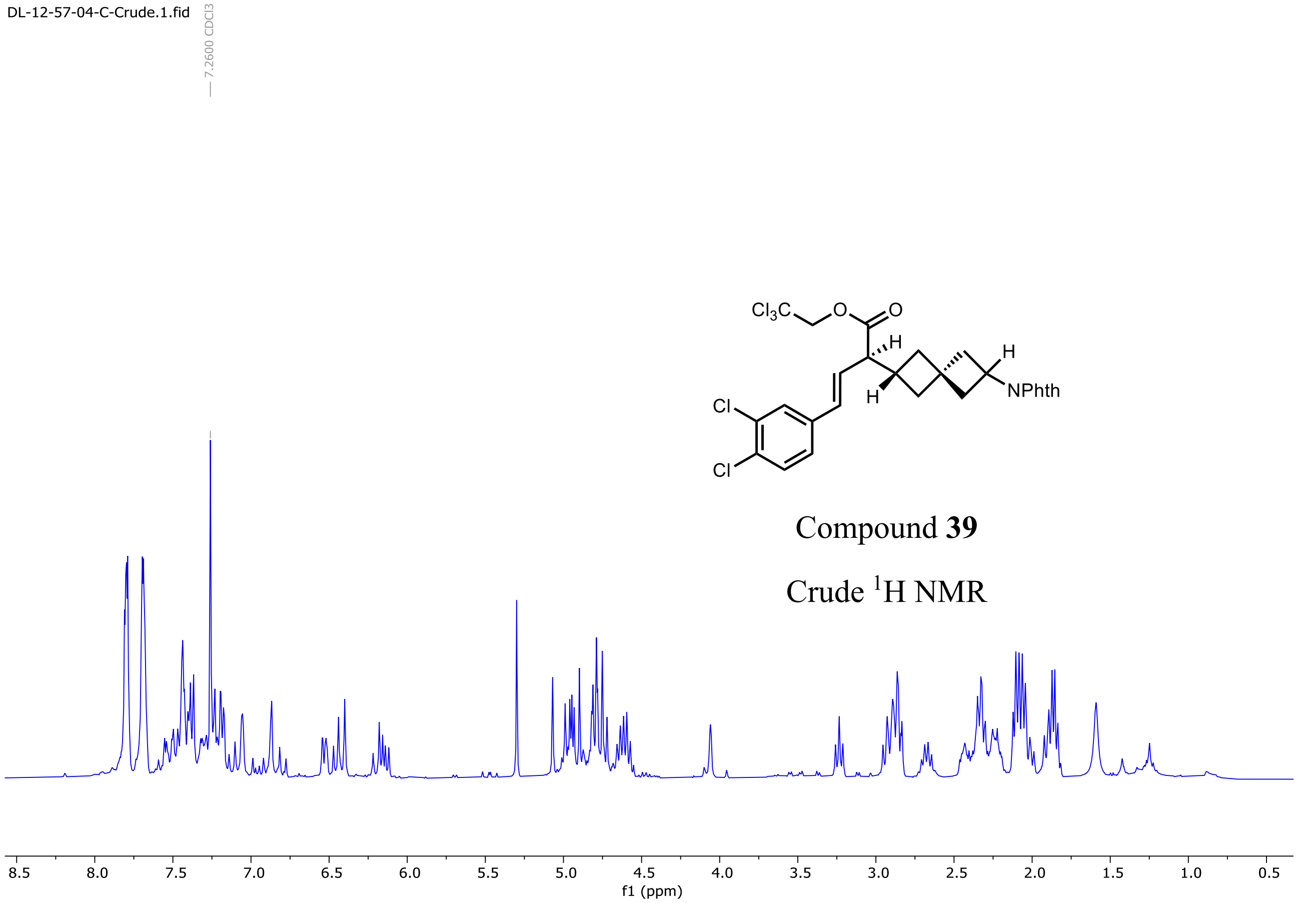
DL-12-57-04-C-Crude.1.fid
7.2600 CDCl3
Cl3C
O
O
H
H
H
NPhth
Cl
Cl
Compound **39**
Crude ^{1}H NMR
f1 (ppm)

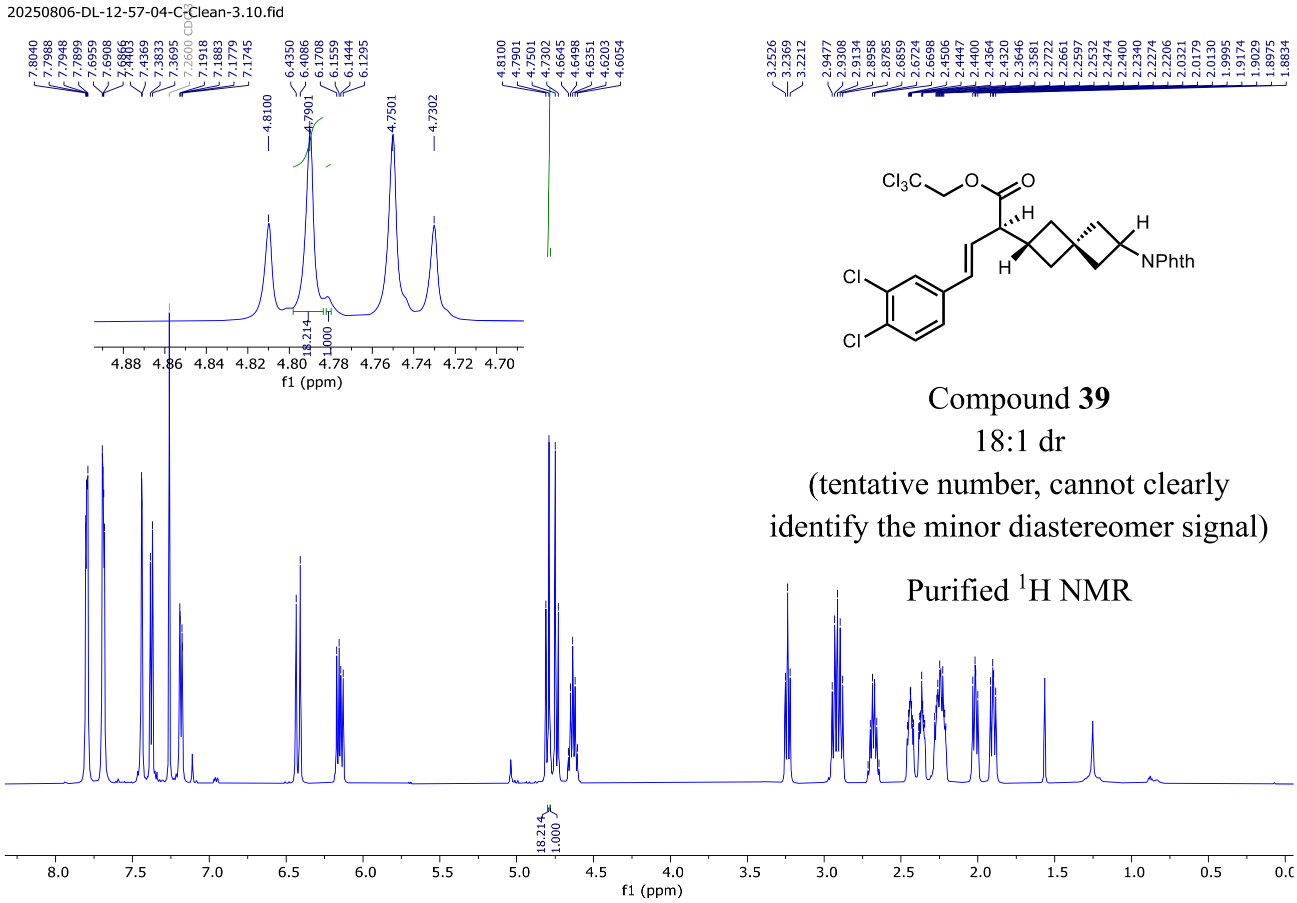
20250806-DL-12-57-04-C-Clean-3.10.fid
4.8100
4.7901
4.7501
4.7302
18.214
1.000
f1 (ppm)
Cl3C
O
O
H
H
H
NPhth
Cl
Cl
Compound **39**
18:1 dr
(tentative number, cannot clearly identify the minor diastereomer signal)
Purified ^{1}H NMR
f1 (ppm)

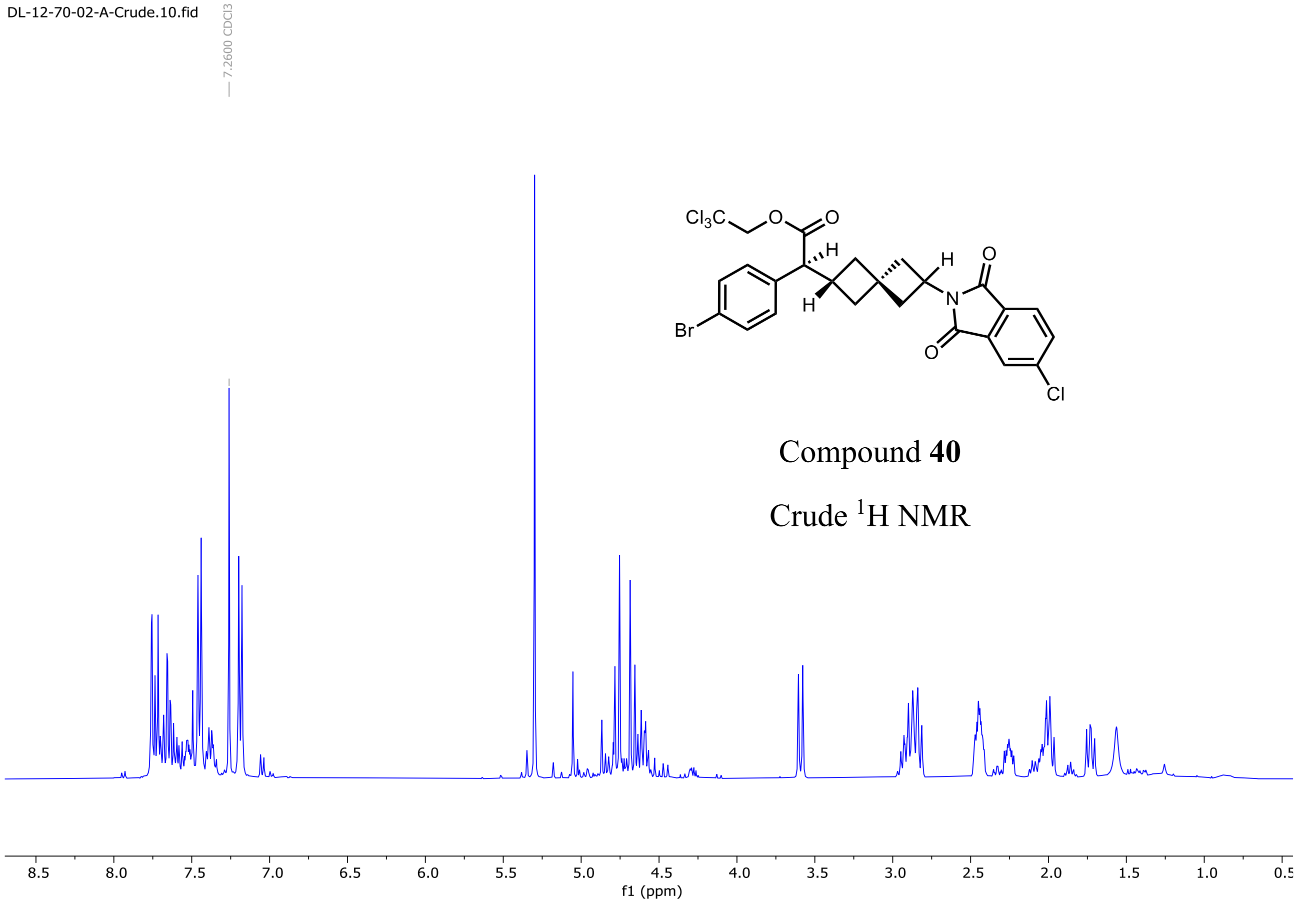
DL-12-70-02-A-Crude.10.fid
7.2600 CDCl3
Compound 40
Crude 1H NMR
f1 (ppm)

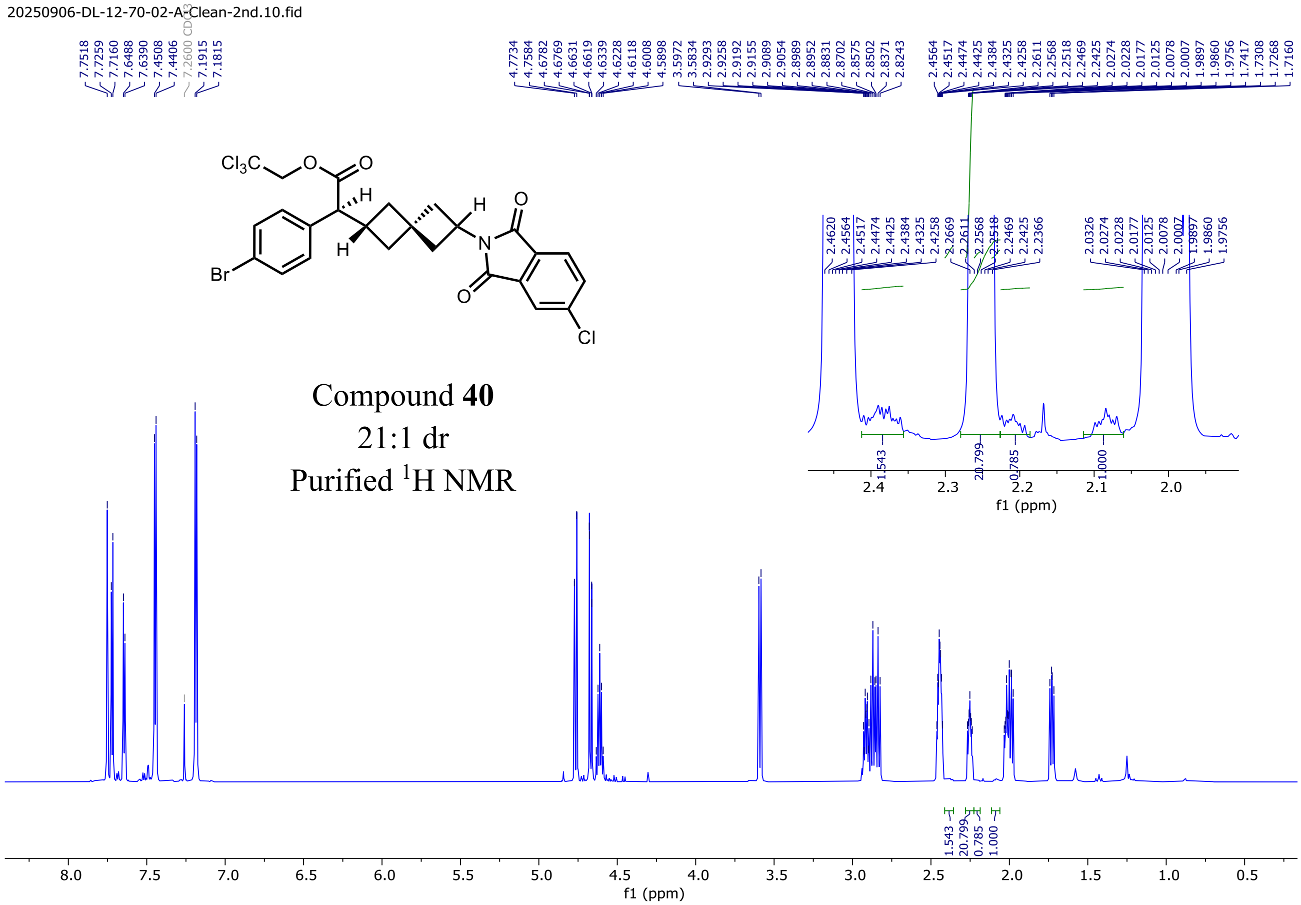
20250906-DL-12-70-02-A-Clean-2nd.10.fid
Compound 40
21:1 dr
Purified 1H NMR
1.543
20.799
0.785
1.000
f1 (ppm)

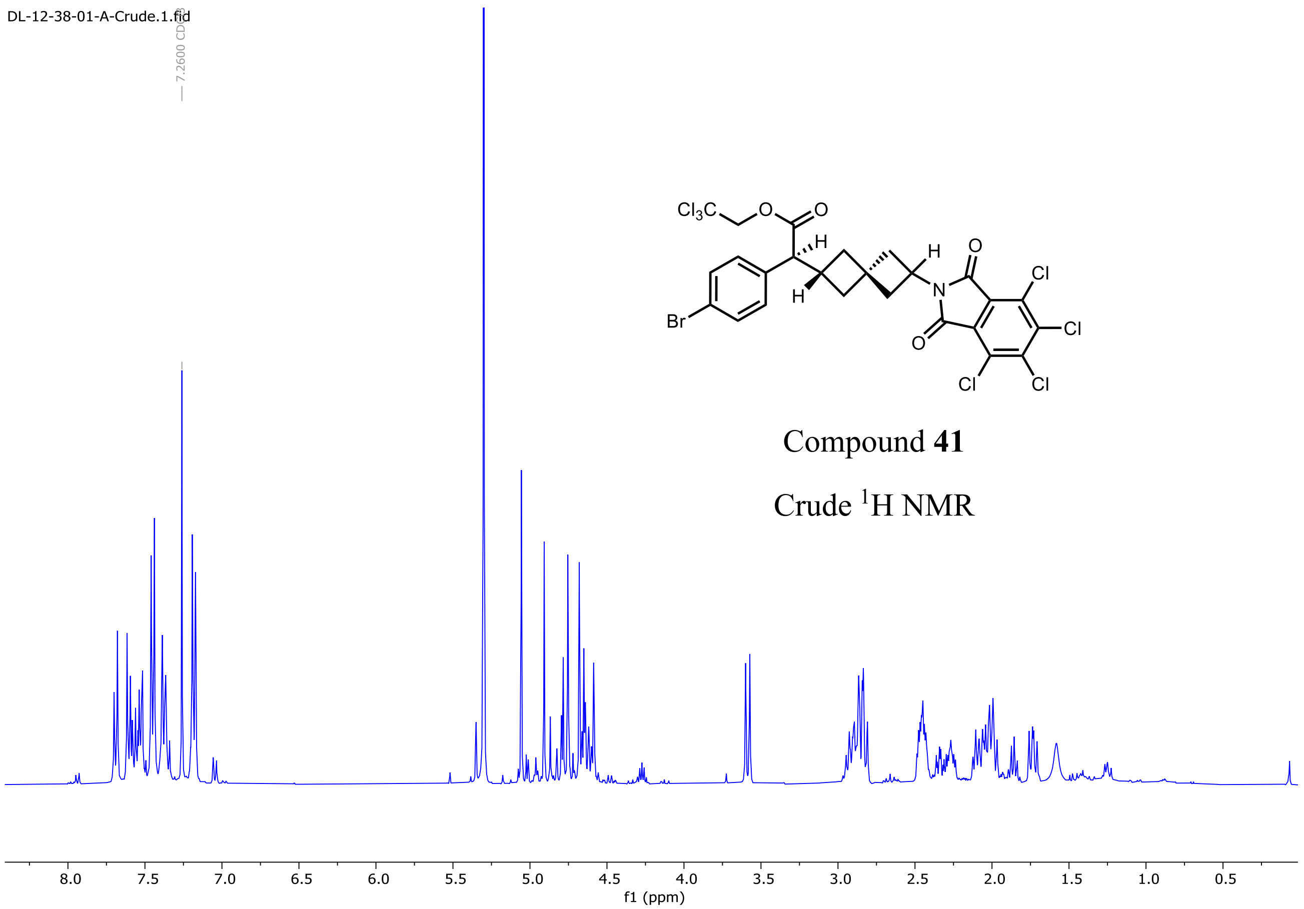

DL-12-38-01-A-Crude.1.fid
7.2600 CDCl3
Cl3C
O
O
H
H
Br
H
N
O
O
Cl
Cl
Cl
Cl
Compound 41
Crude 1H NMR
8.0 7.5 7.0 6.5 6.0 5.5 5.0 4.5 4.0 3.5 3.0 2.5 2.0 1.5 1.0 0.5
f1 (ppm)

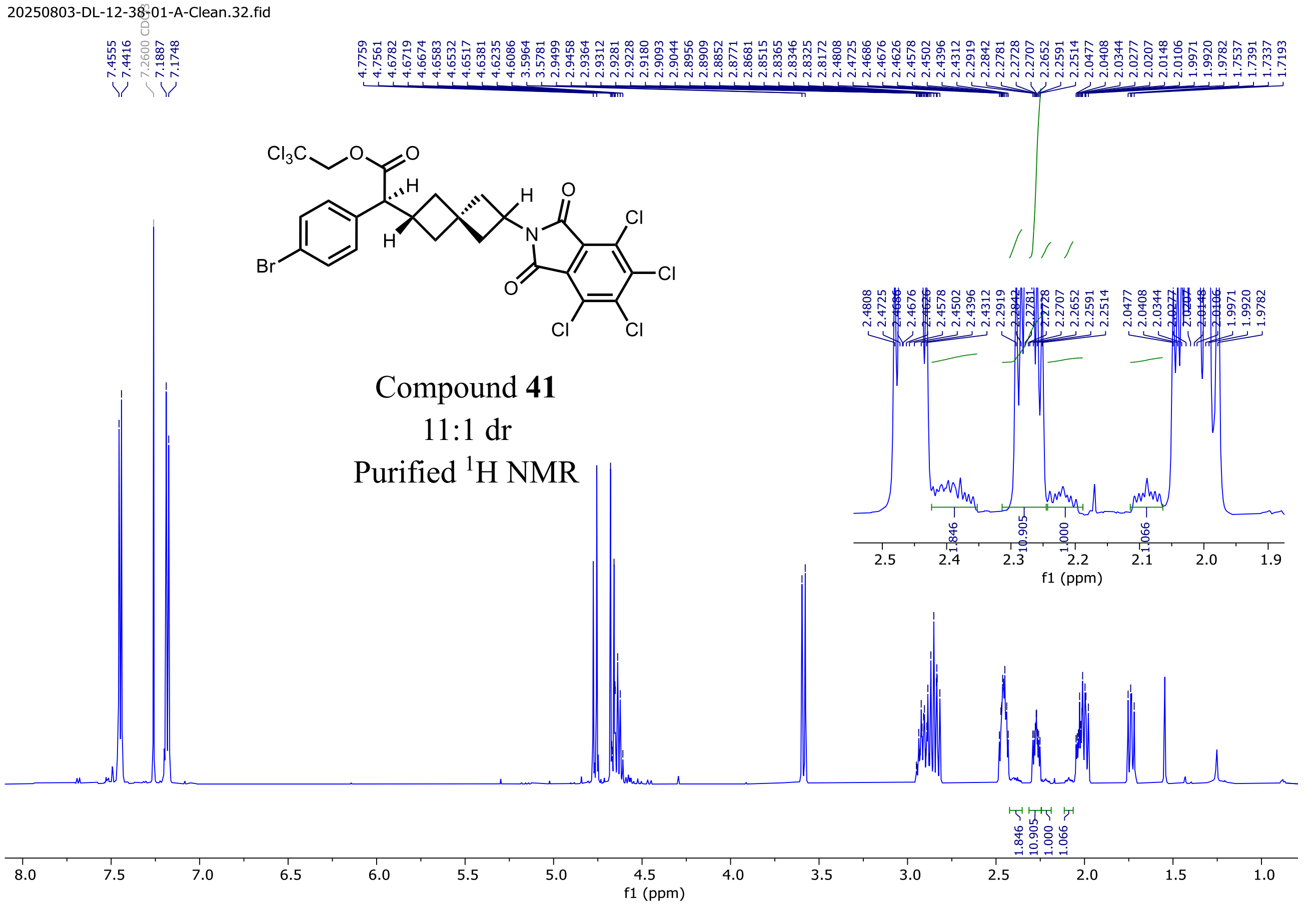

20250803-DL-12-38-01-A-Clean.32.fid
7.4555
7.4416
7.2600 CDCl3
7.1887
7.1748
4.7759 4.7561 4.6782 4.6719 4.6674 4.6583 4.6532 4.6517 4.6381 4.6235 4.6086 3.5964 3.5781 2.9499 2.9458 2.9364 2.9312 2.9281 2.9228 2.9180 2.9093 2.9044 2.8956 2.8909 2.8852 2.8771 2.8681 2.8515 2.8365 2.8346 2.8325 2.8172 2.4808 2.4725 2.4686 2.4676 2.4626 2.4578 2.4502 2.4396 2.4312 2.2919 2.2842 2.2781 2.2728 2.2707 2.2652 2.2591 2.2514 2.0477 2.0408 2.0344 2.0277 2.0207 2.0148 2.0106 1.9971 1.9920 1.9782 1.7537 1.7391 1.7337 1.7193
Cl3C
O
O
H
H
Br
H
N
O
O
Cl
Cl
Cl
Cl
Compound 41
11:1 dr
Purified 1H NMR
1.846
10.905
1.000
1.066
2.5 2.4 2.3 2.2 2.1 2.0 1.9
f1 (ppm)
8.0 7.5 7.0 6.5 6.0 5.5 5.0 4.5 4.0 3.5 3.0 2.5 2.0 1.5 1.0
f1 (ppm)

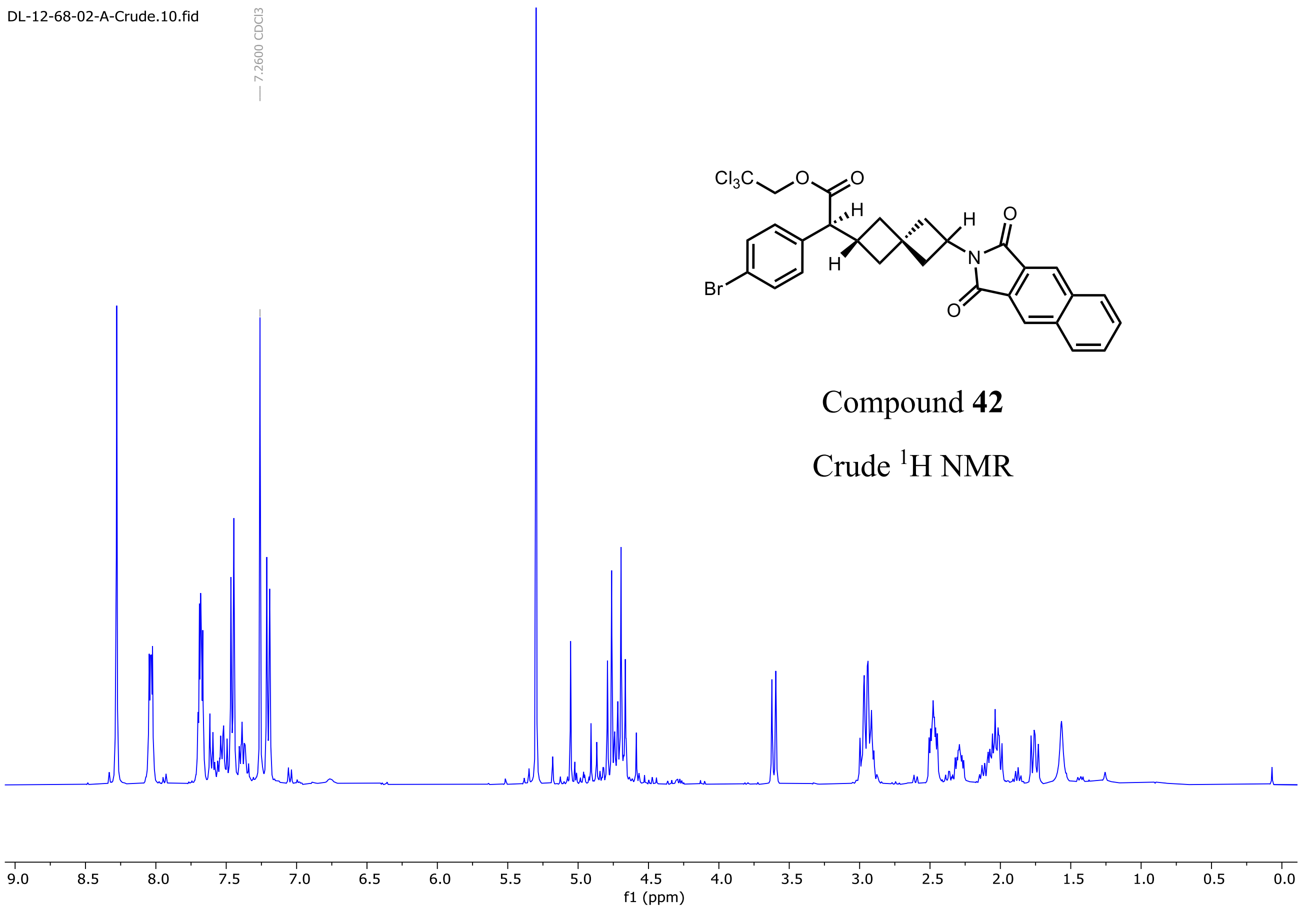
DL-12-68-02-A-Crude.10.fid
7.2600 CDCl3
Cl3C
O
O
H
H
H
N
O
O
Br
Compound 42
Crude 1H NMR
f1 (ppm)

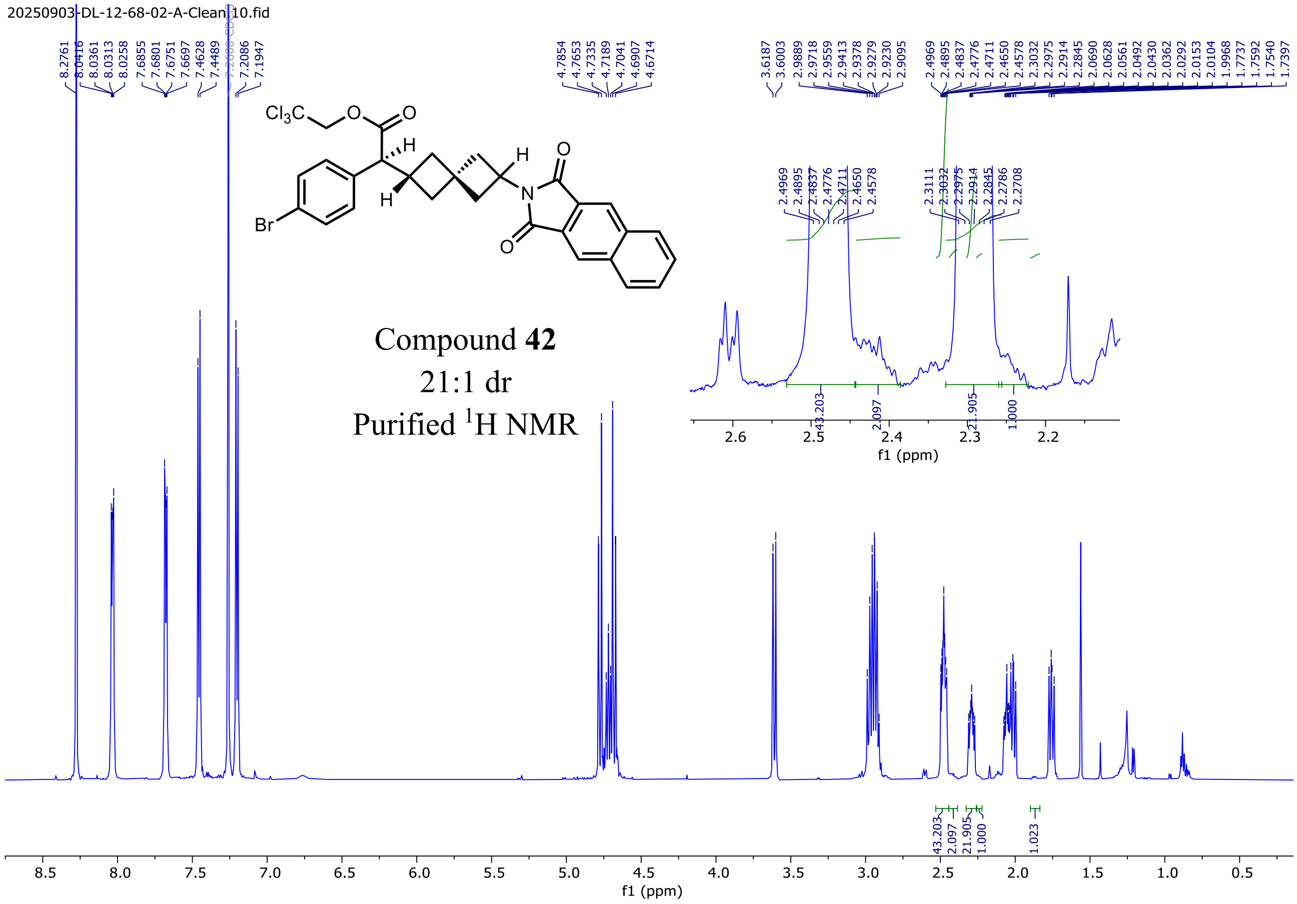
20250903-DL-12-68-02-A-Clean.10.fid
8.2761
8.0416
8.0361
8.0313
8.0258
7.6855
7.6801
7.6751
7.6697
7.4628
7.4489
7.2086
7.1947
4.7854
4.7653
4.7335
4.7189
4.7041
4.6907
4.6714
3.6187
3.6003
2.9889
2.9718
2.9559
2.9413
2.9378
2.9279
2.9230
2.9095
2.4969
2.4895
2.4837
2.4776
2.4711
2.4650
2.4578
2.3032
2.2975
2.2914
2.2845
2.0690
2.0628
2.0561
2.0492
2.0430
2.0362
2.0292
2.0153
2.0104
1.9968
1.7737
1.7592
1.7540
1.7397
Cl3C
O
O
H
H
H
N
O
O
Br
Compound 42
21:1 dr
Purified 1H NMR
2.3111
2.2786
2.2708
43.203
2.097
21.905
1.000
1.023
f1 (ppm)

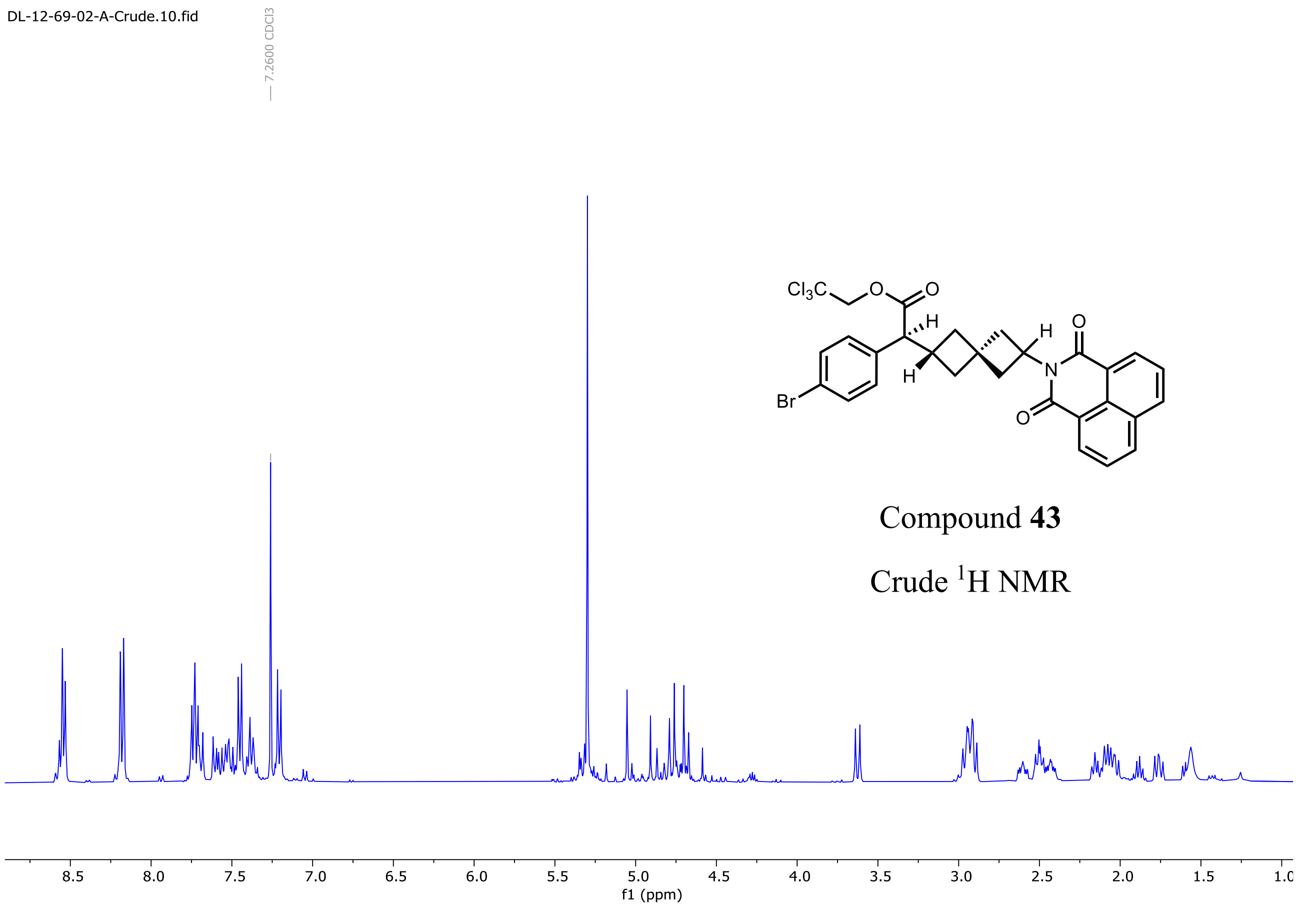
DL-12-69-02-A-Crude.10.fid
7.2600 CDCl3
Compound 43
Crude 1H NMR
f1 (ppm)

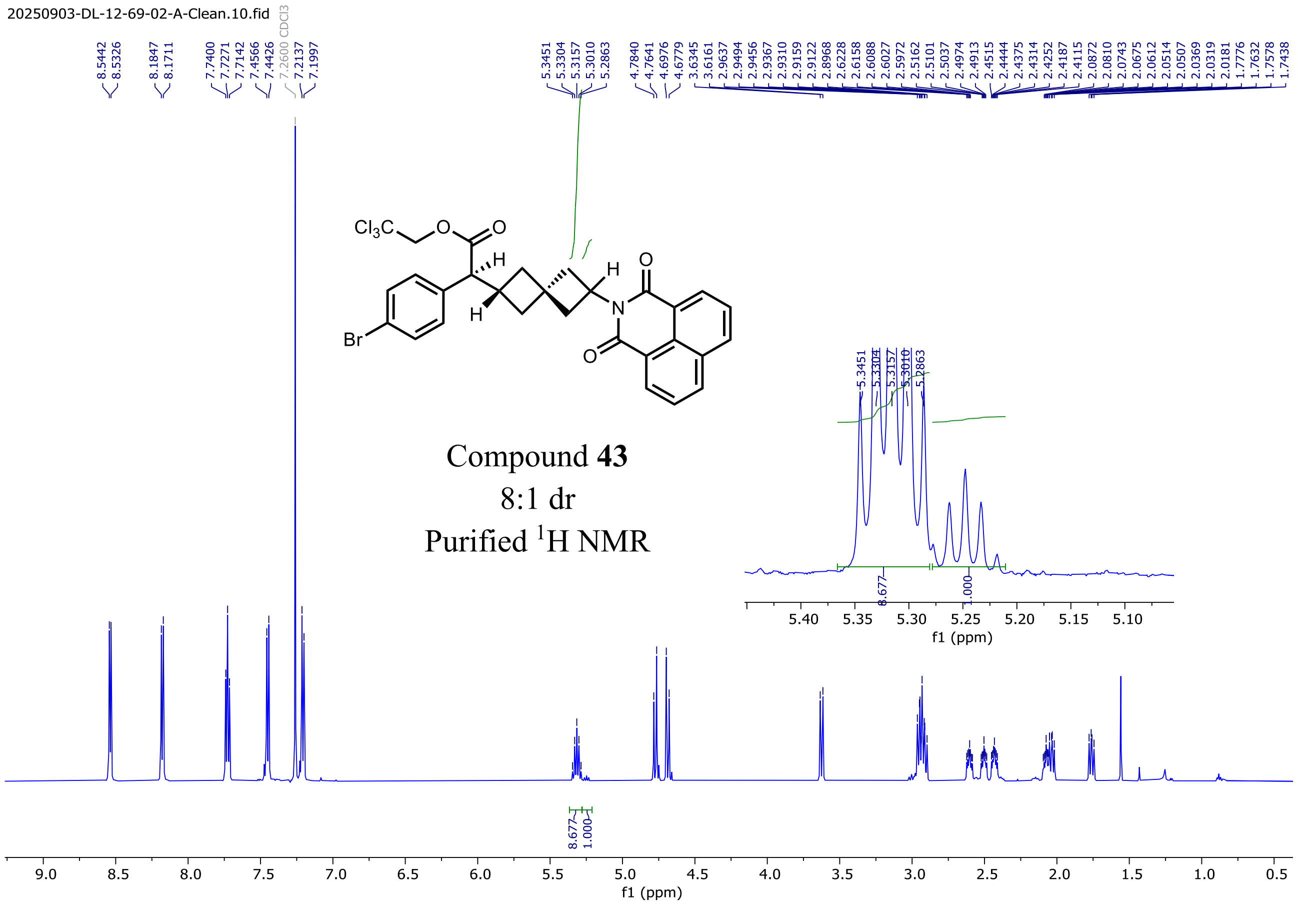
20250903-DL-12-69-02-A-Clean.10.fid
Compound 43
8:1 dr
Purified 1H NMR
8.677
1.000
f1 (ppm)

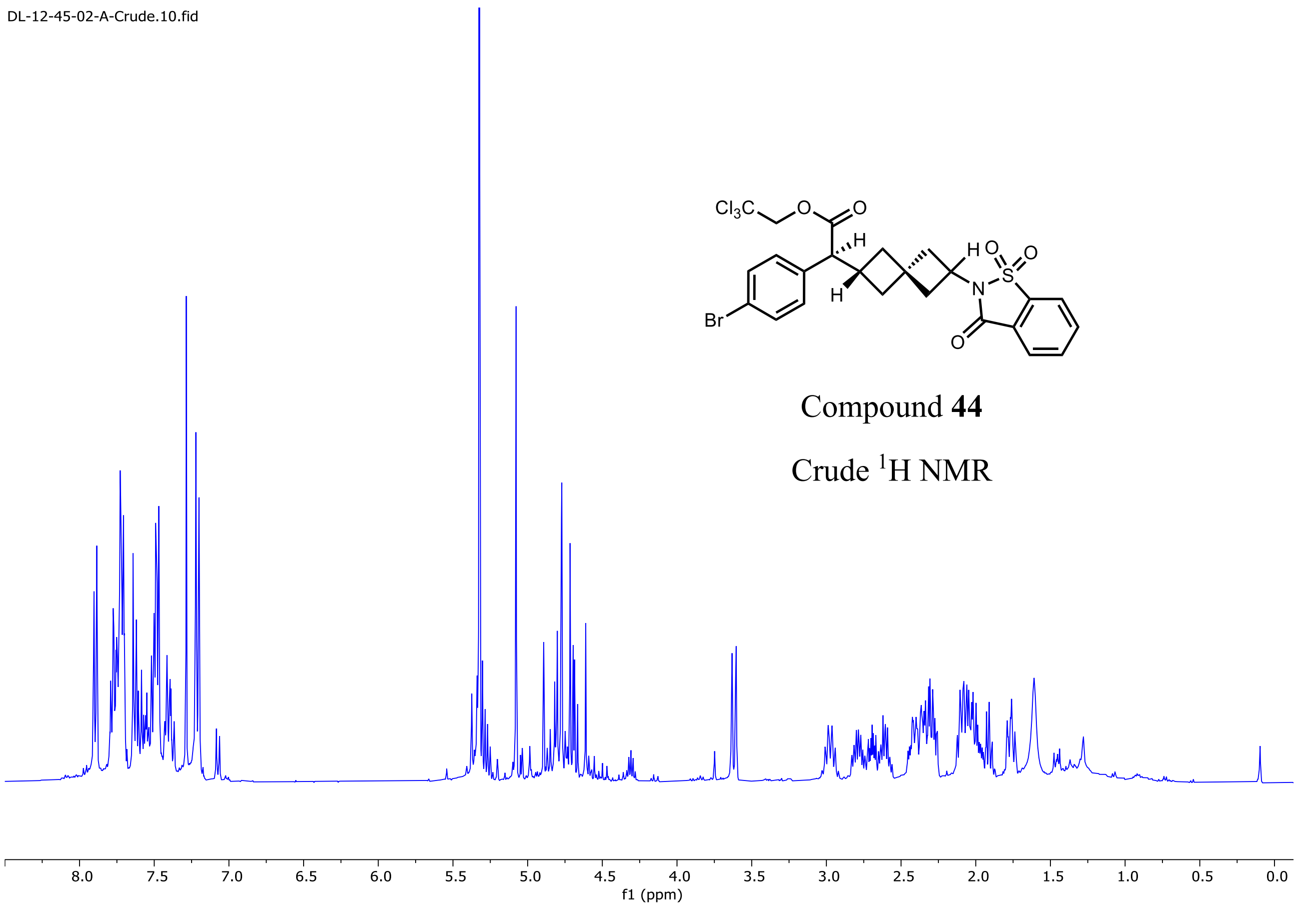

DL-12-45-02-A-Crude.10.fid
Cl3C O O H H O O H N S O Br
Compound 44
Crude 1H NMR
8.0 7.5 7.0 6.5 6.0 5.5 5.0 4.5 4.0 3.5 3.0 2.5 2.0 1.5 1.0 0.5 0.0
f1 (ppm)

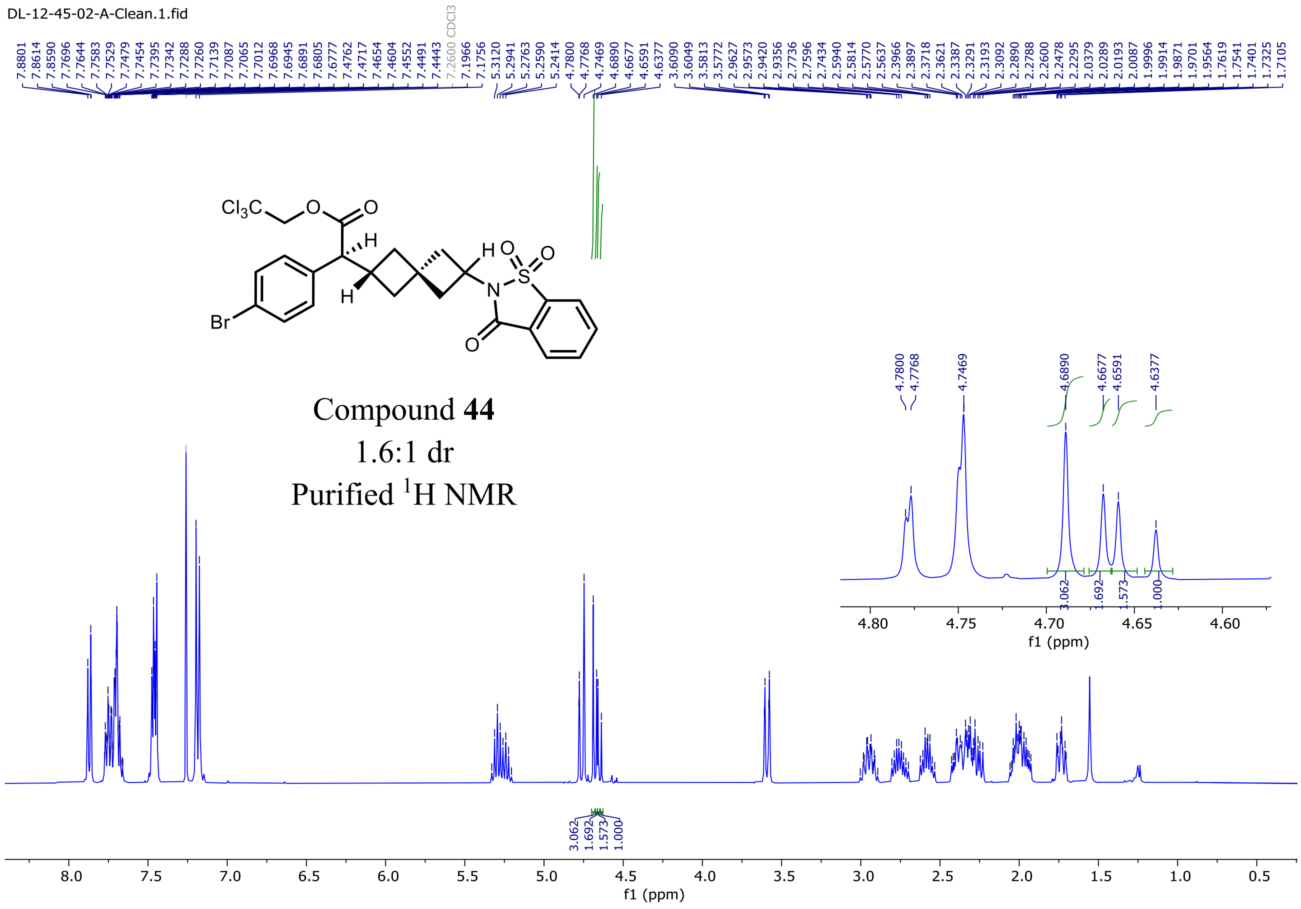

DL-12-45-02-A-Clean.1.fid
7.8801 7.8614 7.8590 7.7696 7.7644 7.7583 7.7529 7.7479 7.7454 7.7395 7.7342 7.7288 7.7260 7.7139 7.7087 7.7065 7.7012 7.6968 7.6945 7.6891 7.6805 7.6777 7.4762 7.4717 7.4654 7.4604 7.4552 7.4491 7.4443 7.2600 CDCl3 7.1966 7.1756 5.3120 5.2941 5.2763 5.2590 5.2414 4.7800 4.7768 4.7469 4.6890 4.6677 4.6591 4.6377 3.6090 3.6049 3.5813 3.5772 2.9627 2.9573 2.9420 2.9356 2.7736 2.7596 2.7434 2.5940 2.5814 2.5770 2.5637 2.3966 2.3897 2.3718 2.3621 2.3387 2.3291 2.3193 2.3092 2.2890 2.2788 2.2600 2.2478 2.2295 2.0379 2.0289 2.0193 2.0087 1.9996 1.9914 1.9871 1.9701 1.9564 1.7619 1.7541 1.7401 1.7325 1.7105
Cl3C O O H H O O H N S O Br
Compound 44
1.6:1 dr
Purified 1H NMR
4.7800 4.7768 4.7469 4.6890 4.6677 4.6591 4.6377
3.062 1.692 1.573 1.000
4.80 4.75 4.70 4.65 4.60
f1 (ppm)
3.062 1.692 1.573 1.000
8.0 7.5 7.0 6.5 6.0 5.5 5.0 4.5 4.0 3.5 3.0 2.5 2.0 1.5 1.0 0.5
f1 (ppm)

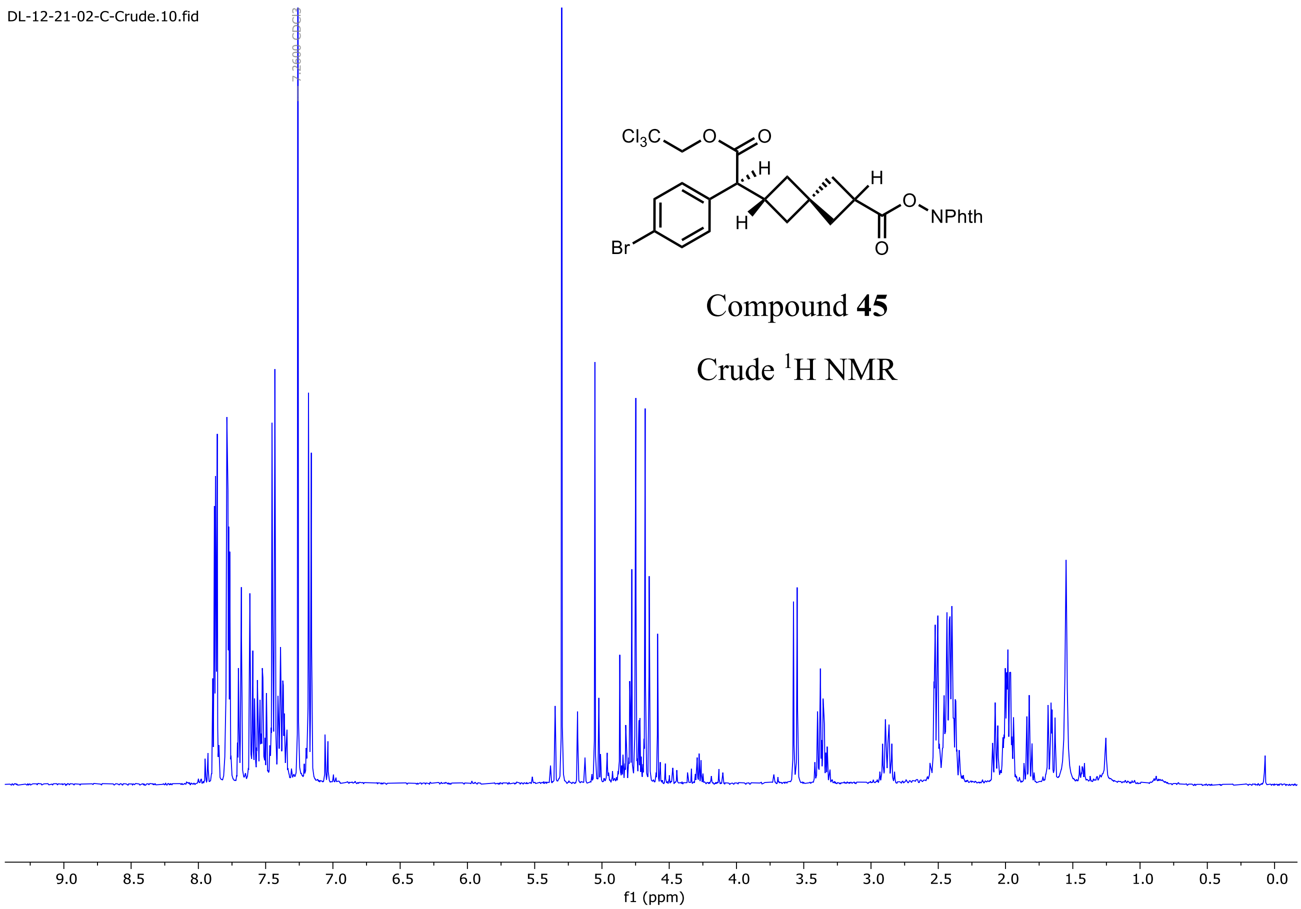
DL-12-21-02-C-Crude.10.fid
7.2600 CDCl3
Cl3C
O
O
H
H
H
O
NPhth
O
Br
Compound 45
Crude 1H NMR
9.0
8.5
8.0
7.5
7.0
6.5
6.0
5.5
5.0
4.5
4.0
3.5
3.0
2.5
2.0
1.5
1.0
0.5
0.0
f1 (ppm)

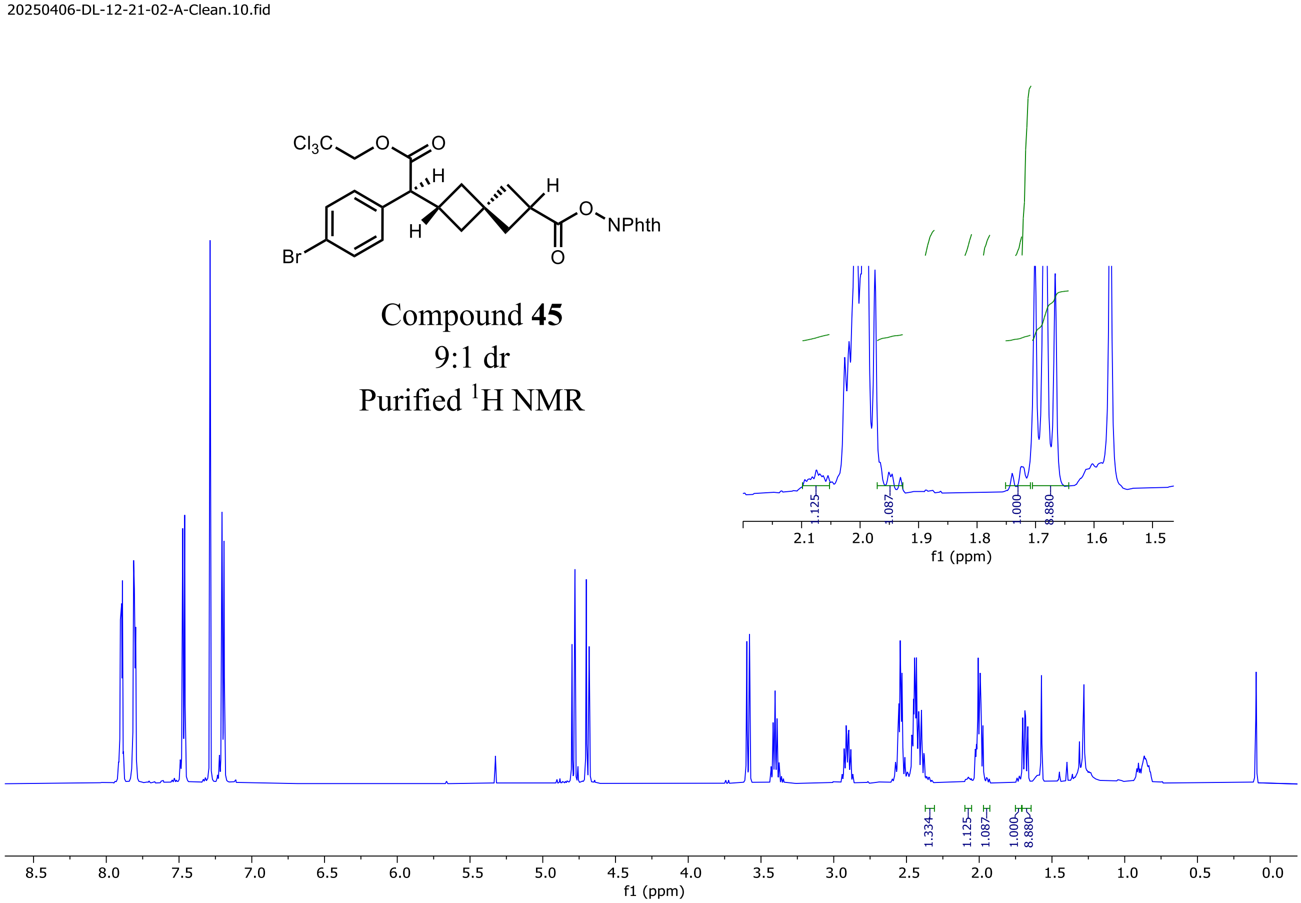
20250406-DL-12-21-02-A-Clean.10.fid
Cl3C
O
O
H
H
H
O
NPhth
O
Br
Compound 45
9:1 dr
Purified 1H NMR
1.125
1.087
1.000
8.880
2.1
2.0
1.9
1.8
1.7
1.6
1.5
f1 (ppm)
1.334
1.125
1.087
1.000
8.880
8.5
8.0
7.5
7.0
6.5
6.0
5.5
5.0
4.5
4.0
3.5
3.0
2.5
2.0
1.5
1.0
0.5
0.0
f1 (ppm)

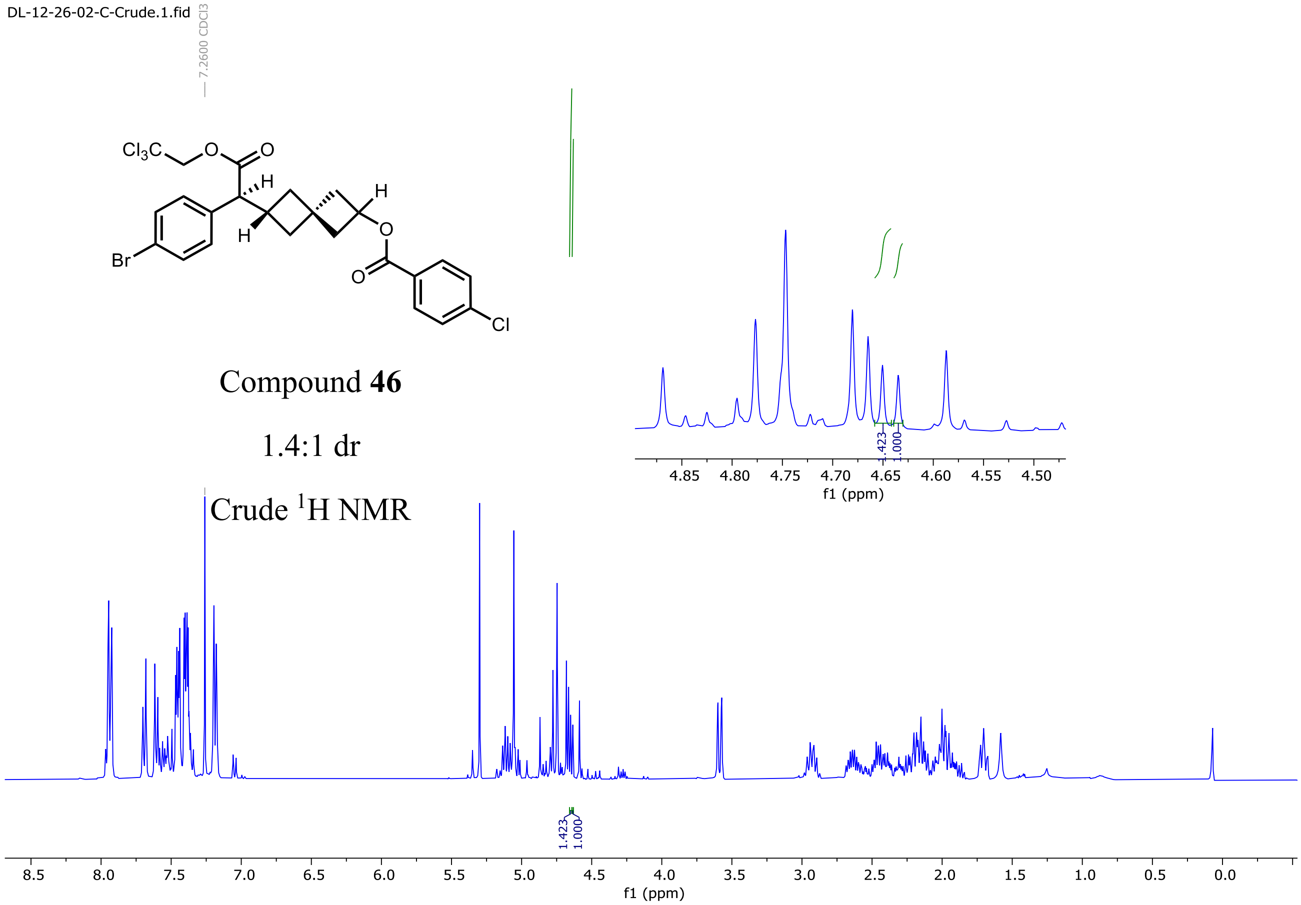
DL-12-26-02-C-Crude.1.fid
7.2600 CDCl3
Compound **46**
1.4:1 dr
Crude ^{1}H NMR
1.423
1.000
f1 (ppm)

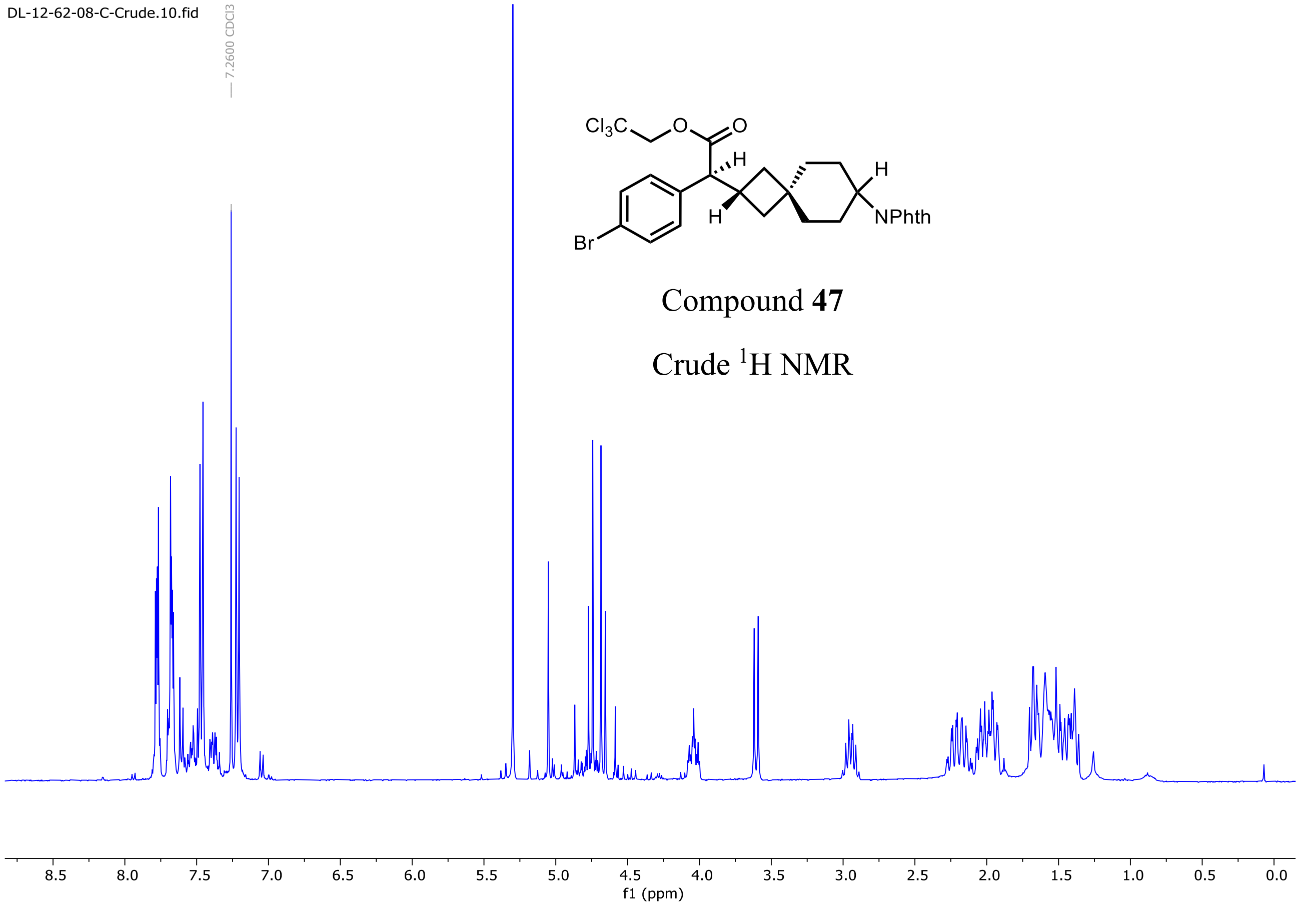
DL-12-62-08-C-Crude.10.fid
7.2600 CDCl3
Cl3C
O
O
H
H
NPhth
H
Br
Compound 47
Crude 1H NMR
f1 (ppm)

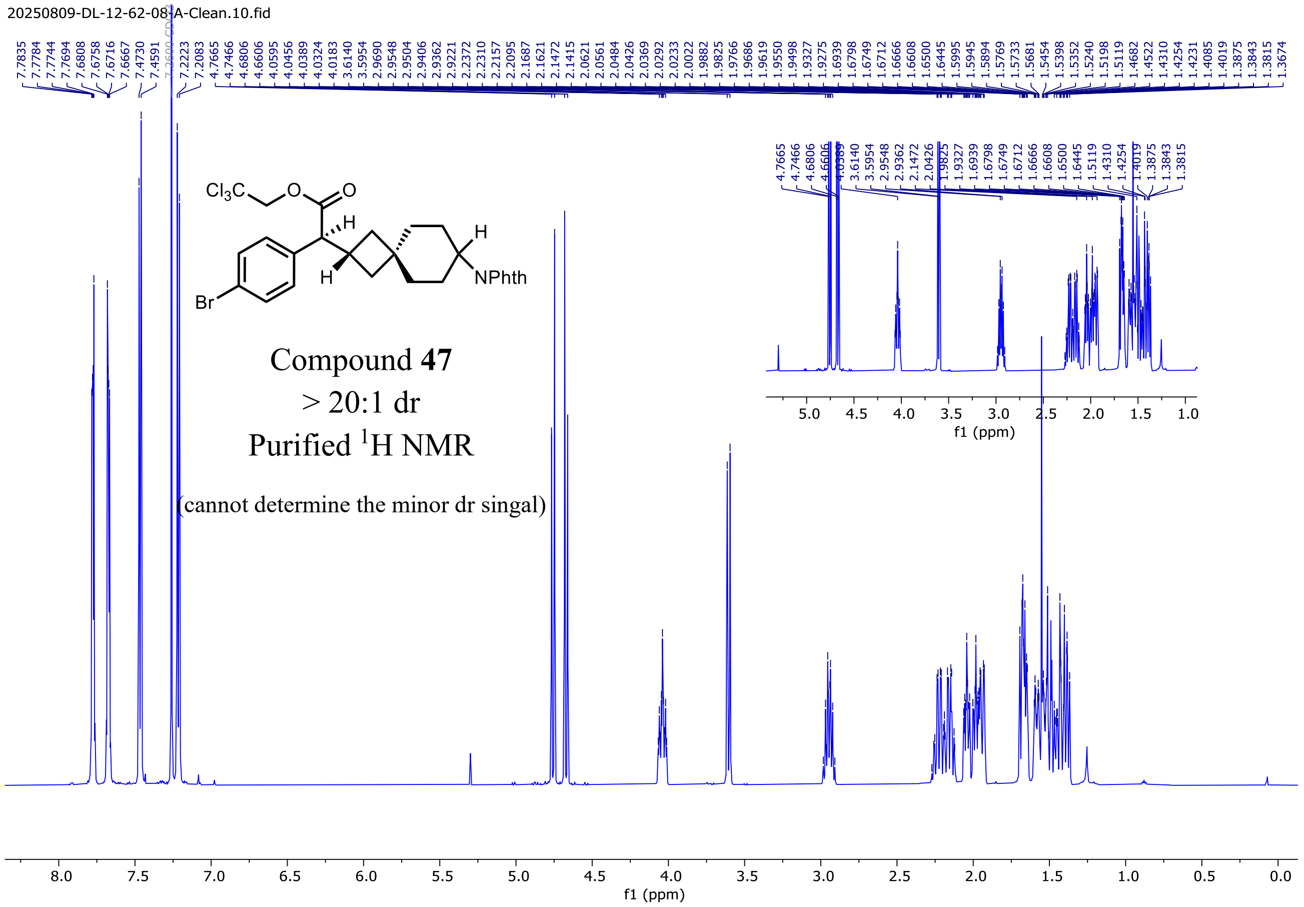
20250809-DL-12-62-08-A-Clean.10.fid
Cl3C
O
O
H
H
NPhth
H
Br
Compound 47
> 20:1 dr
Purified 1H NMR
(cannot determine the minor dr singal)
f1 (ppm)

DL-12-71-06-A-Crude.1.fid

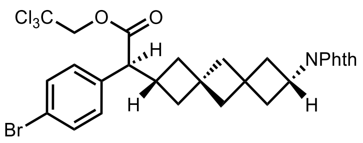

Compound **48**

2:1 dr

Crude ^{1}H NMR

The dr was determined by ^{13}C-NMR due to low resolution on ^{1}H-NMR

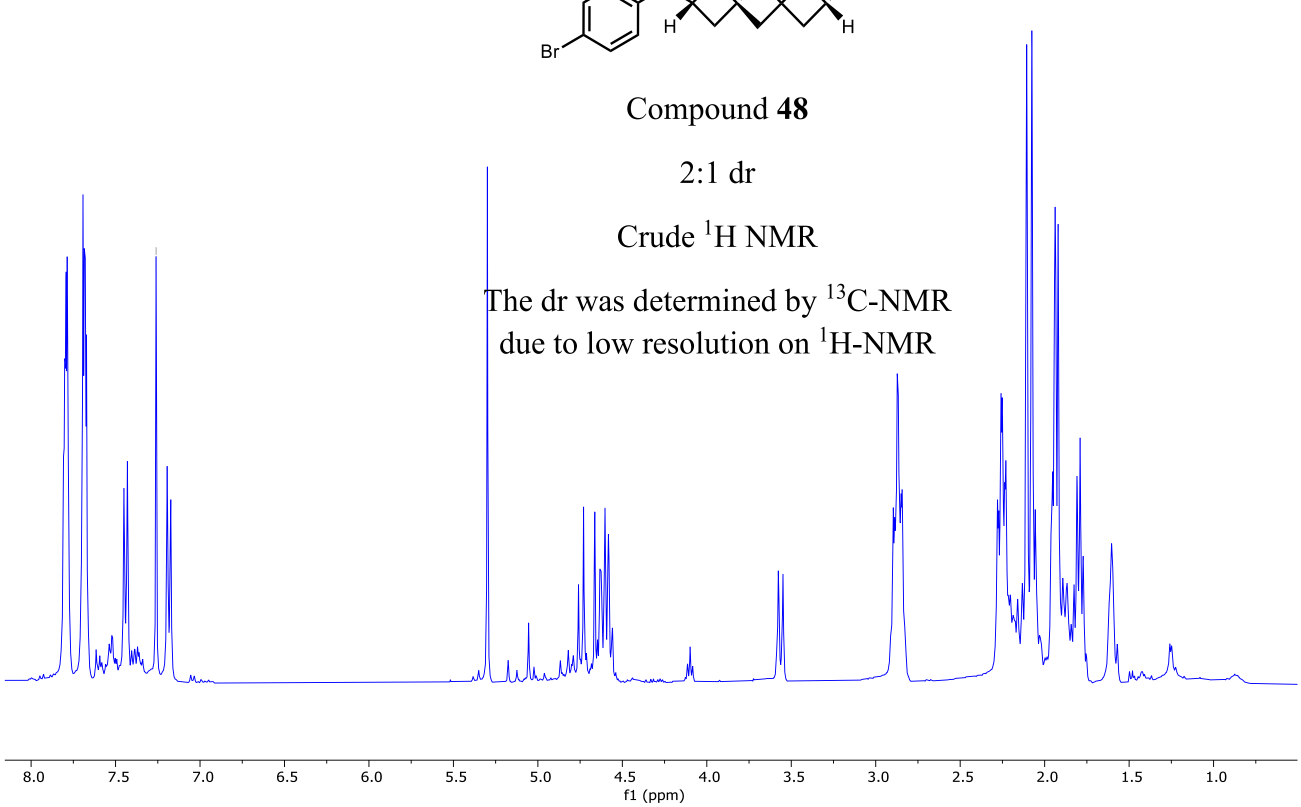

20250904-DL-12-71-06-C-Clean.11.fid

Compound **48**

2:1 dr

Purified ^{13}C NMR

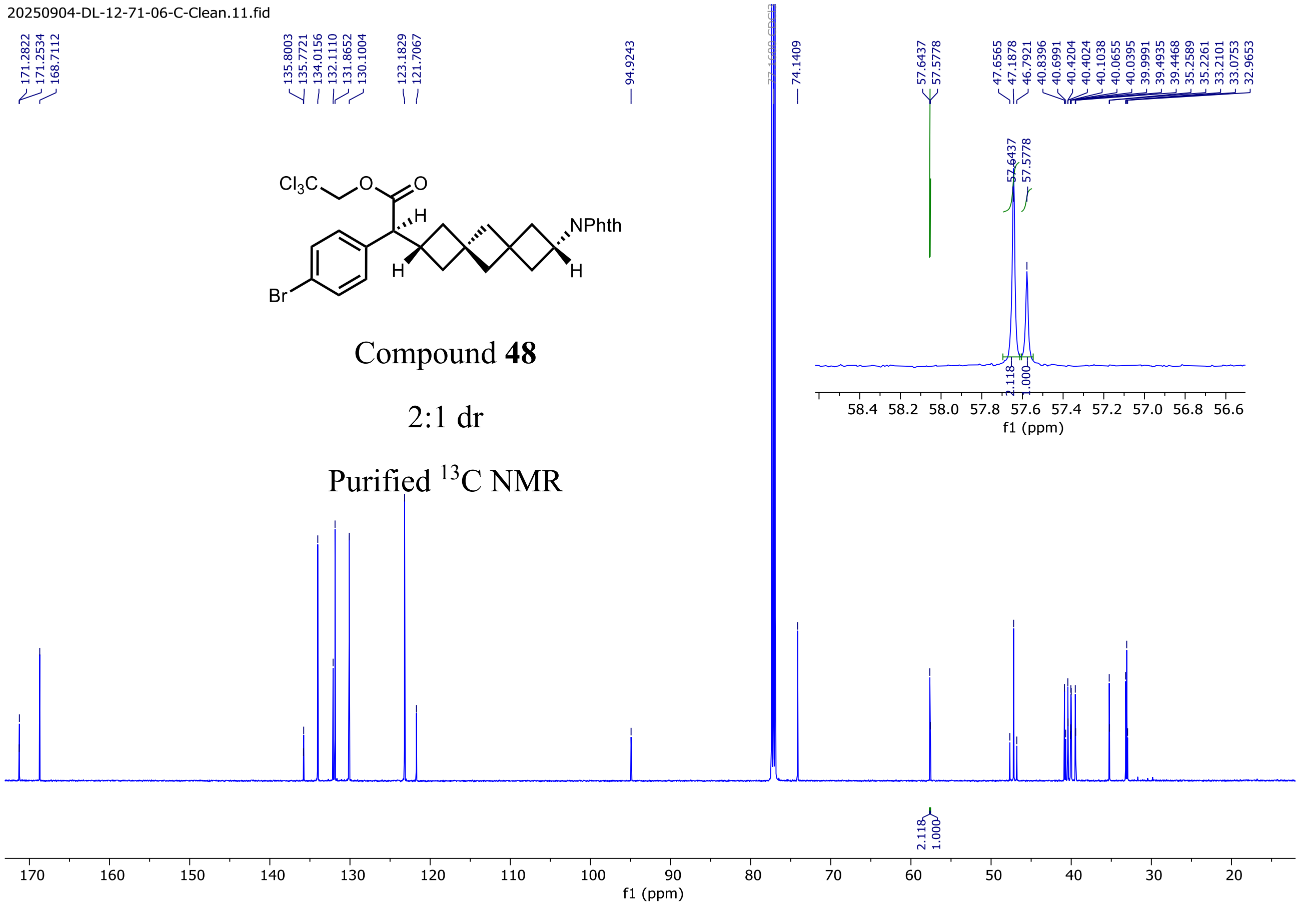

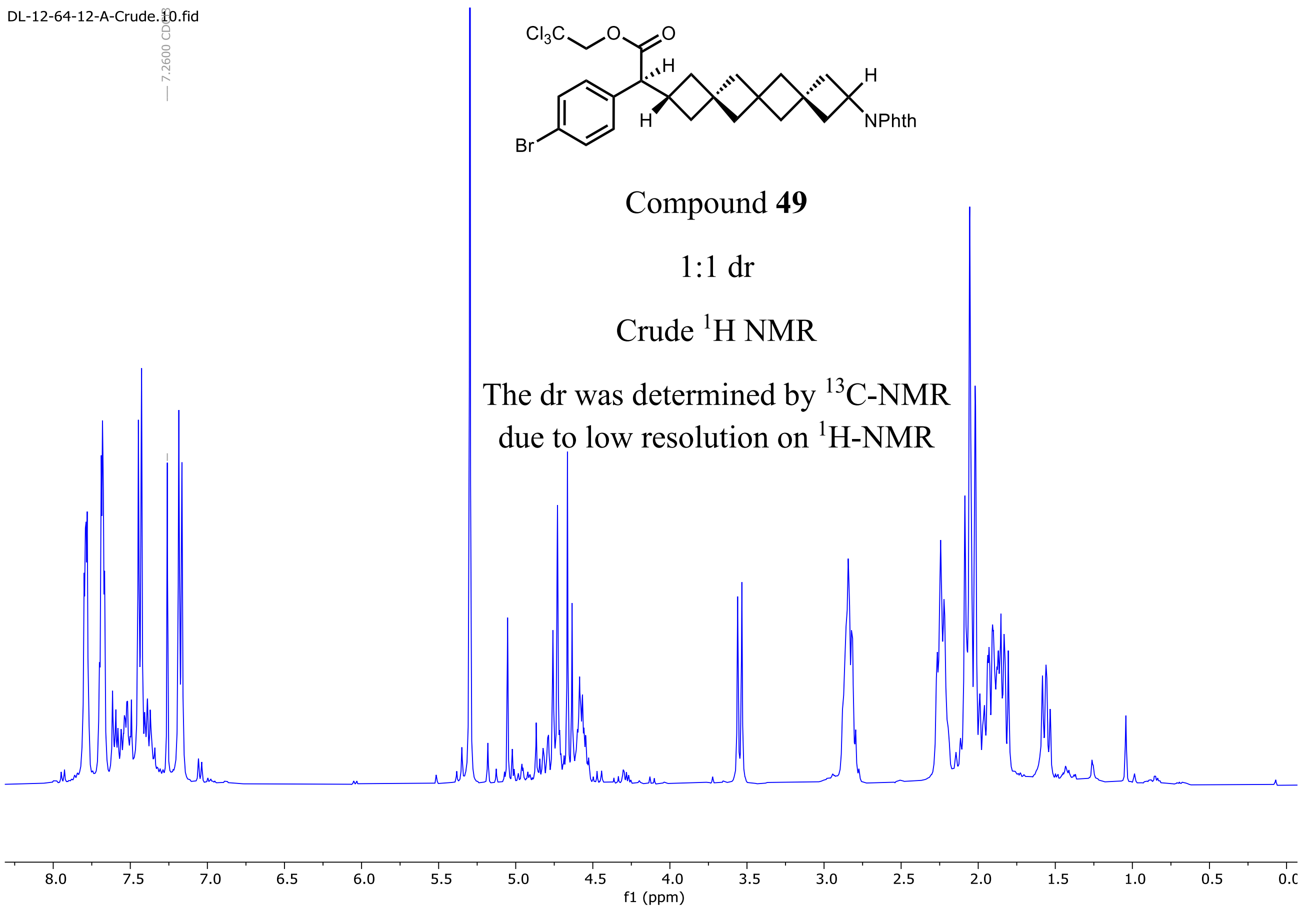
DL-12-64-12-A-Crude.10.fid
7.2600 CDCl3
Cl3C
O
O
H
H
H
Br
NPhth
Compound 49
1:1 dr
Crude 1H NMR
The dr was determined by 13C-NMR
due to low resolution on 1H-NMR
8.0 7.5 7.0 6.5 6.0 5.5 5.0 4.5 4.0 3.5 3.0 2.5 2.0 1.5 1.0 0.5
f1 (ppm)

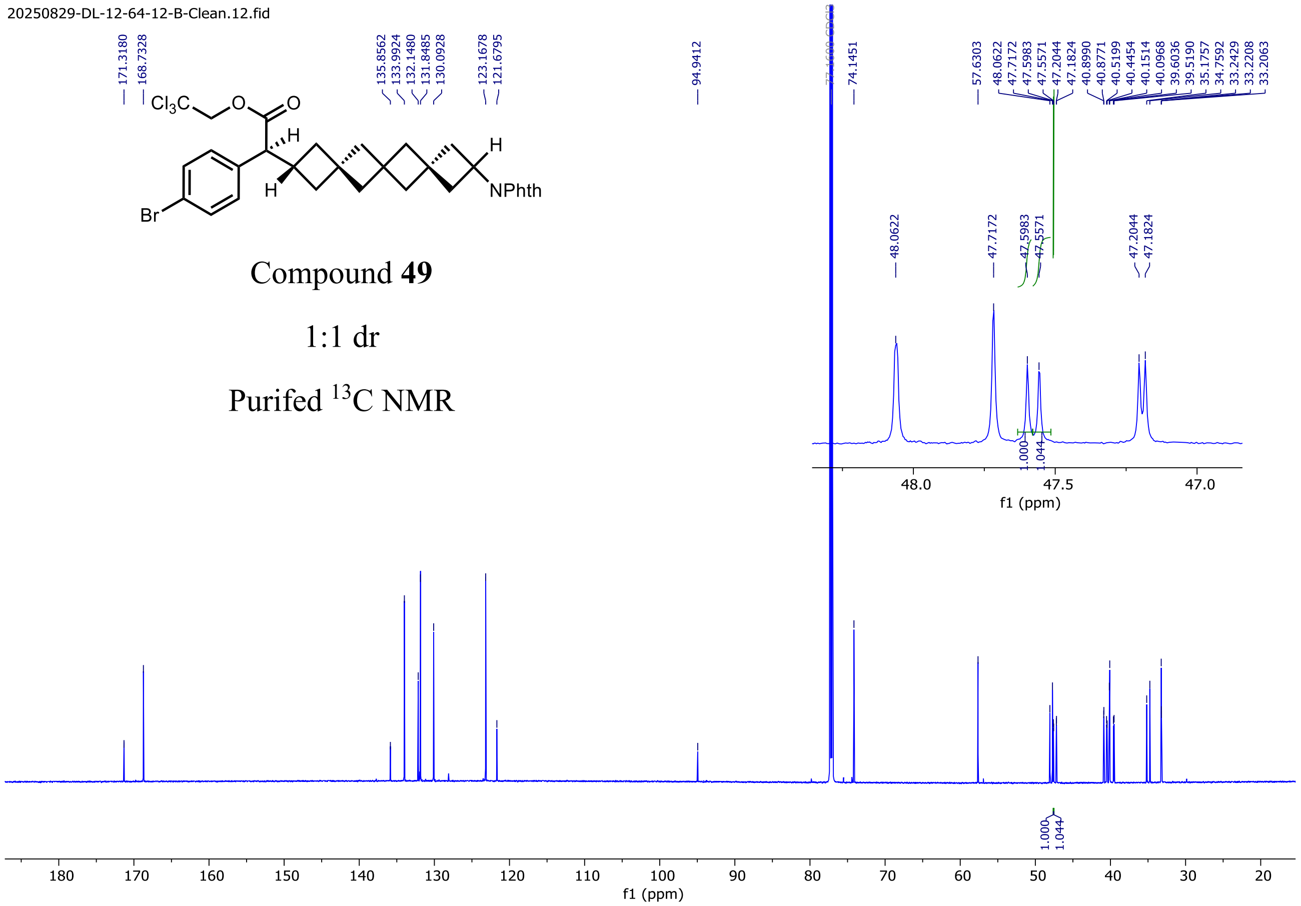
20250829-DL-12-64-12-B-Clean.12.fid
171.3180
168.7328
135.8562
133.9924
132.1480
131.8485
130.0928
123.1678
121.6795
94.9412
74.1451
57.6303
48.0622
47.7172
47.5983
47.5571
47.2044
47.1824
40.8990
40.8771
40.5199
40.4454
40.1514
40.0968
39.6036
39.5190
35.1757
34.7592
33.2429
33.2208
33.2063
Cl3C
O
O
H
H
H
Br
NPhth
Compound 49
1:1 dr
Purifed 13C NMR
1.000
1.044
48.0 47.5 47.0
f1 (ppm)
180 170 160 150 140 130 120 110 100 90 80 70 60 50 40 30 20
f1 (ppm)

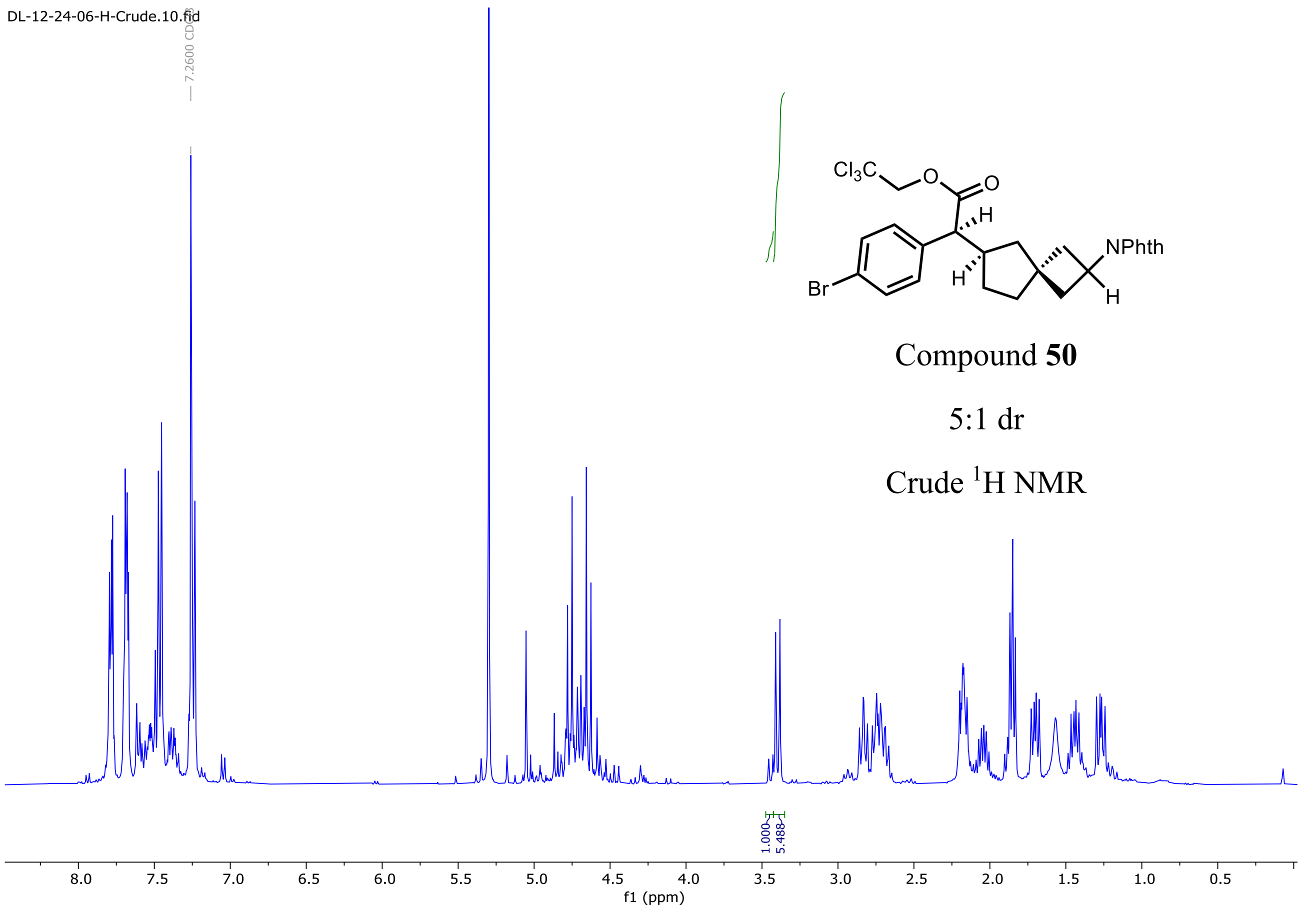

Compound **50**

5:1 dr

Crude ^{1}H NMR

5. Substrate synthesis

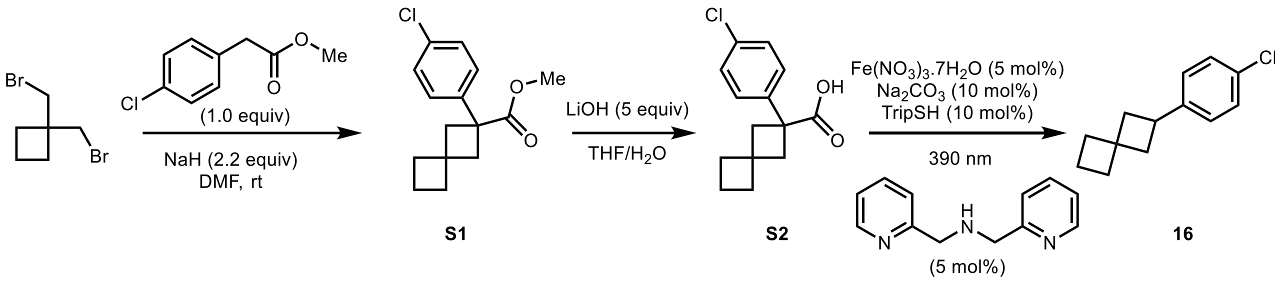

To a solution of methyl 2-(4-chlorophenyl)acetate (2.50 g, 13.5 mmol, 1.0 equiv) in DMF (22.6 mL) was added NaH (1.19 g, 60% Wt, 29.8 mmol, 2.2 equiv) one portions at 0 ºC. After stirring 15 min, 1,1-bis(bromomethyl)cyclobutane (3.60 g, 91% wt, 1 equiv, 13.5 mmol) in DMF (4.51 mL) was added dropwise at 0. The mixture was stirred at room temperature overnight. The reaction mixture was poured into aq. H_2O and extracted with ethyl acetate. The combined organic layers were dried over Na_2SO_4, and concentrated. The crude was purified by flash chromatography (SiO_2, 0-5% Et_2O in hexane) to give the desired product – **compound S1** – as clear oil (1.1 g, 31% yield).

To a solution of methyl 2-(4-chlorophenyl)spiro[3.3]heptane-2-carboxylate (1.1 g, 4.2 mmol, 1.0 equiv) in THF:H_2O (20 ml, 1:1) was added LiOH.H_2O (888 mg, 21.2 mmol, 5 equiv). The mixture was stirred at room temperature overnight. The reaction is then quenched with HCl 2.0N and extracted with ethyl acetate. The combined organic layers were dried over Na_2SO_4, and concentrated. The crude can be used for the next step without purification. The pure product could be obtained by flash chromatography (SiO_2, 0-20% Ethyl acetate in hexane) to give the desired product – **compound S2** – as a white solid (quant.).

The decarboxylation was adapted from the procedure reported by Weix group.[1] 1-(4-chlorophenyl)cyclobutane-1-carboxylic acid (210.66 mg, 1.0 mmol, 1 equiv), Fe(NO3)3.9H_2O (20.2 mg, 0.05 mmol, 5 mol%), di(2-picolyl)amine (9.96 mg, 0.05 mmol, 5 mol%) TRIP sulfide (23.54 mg, 0.05 mmol, 5 mol%) and Na_2CO_3 (10.59 mg, 0.1, 10 mol%) were loaded to a 20 mL vial; then evacuated and charged with N_2 twice. DCE (8 mL) and H_2O (8 mL) were subsequently added to the mixture, sparged with nitrogen balloon for 5 min and sealed the vial with parafilm. The reaction was stirred vigorously for extra 5 min before shining the light with 390 nm LED Kessil lamp. The crude mixture was extracted with DCM. The organic layers were combined and dried over Na_2SO_4. The solvent was removed under vacuum, and the crude mixture was purified by flash chromatography (SiO_2, 100% pentane) to give the product – **compound 16** – as a clear oil (120 mg, 36% yield).

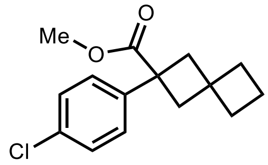

methyl 2-(4-chlorophenyl)spiro[3.3]heptane-2-carboxylate (Compound S1)

R_f (9Hex/1Et2O) = 0.50 (UV 254 nm)

^{1}H NMR (600 MHz, $CDCl_3$) δ 7.29 (m, 2H), 7.26 – 7.21 (m, 2H), 3.64 (s, 3H), 2.93 (d, *J* = 11.6 Hz, 2H), 2.47 (d, *J* = 11.6 Hz, 2H), 2.07 (t, *J* = 7.0 Hz, 2H), 1.91 – 1.85 (m, 2H), 1.85 – 1.78 (m, 2H).

^{13}C NMR (151 MHz, $CDCl_3$) δ 176.2, 142.4, 132.5, 128.5, 128.1, 52.6, 47.7, 45.1, 39.0, 35.3, 35.0, 16.5.

HRMS (+p APCI) calcd. for [$C_{15}H_{18}O_2{}^{35}Cl$] ([M+H]$^+$) 265.1001 found 265.0992.

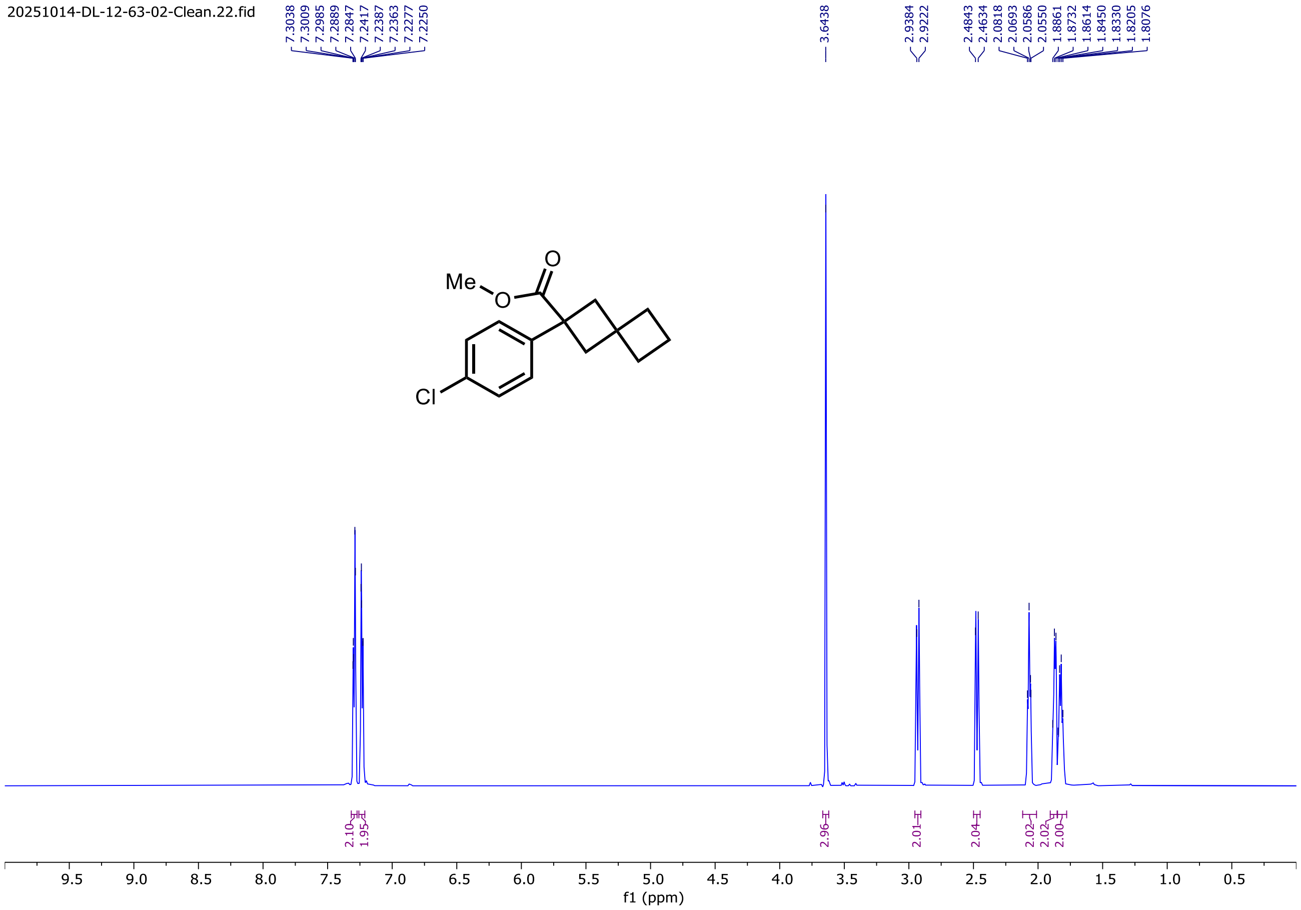
20251014-DL-12-63-02-Clean.22.fid
7.3038
7.3009
7.2985
7.2889
7.2847
7.2417
7.2387
7.2363
7.2277
7.2250
3.6438
2.9384
2.9222
2.4843
2.4634
2.0818
2.0693
2.0586
2.0550
1.8861
1.8732
1.8614
1.8450
1.8330
1.8205
1.8076
Me
O
O
Cl
2.10
1.95
2.96
2.01
2.04
2.02
2.02
2.00
9.5
9.0
8.5
8.0
7.5
7.0
6.5
6.0
5.5
5.0
4.5
4.0
3.5
3.0
2.5
2.0
1.5
1.0
0.5
f1 (ppm)

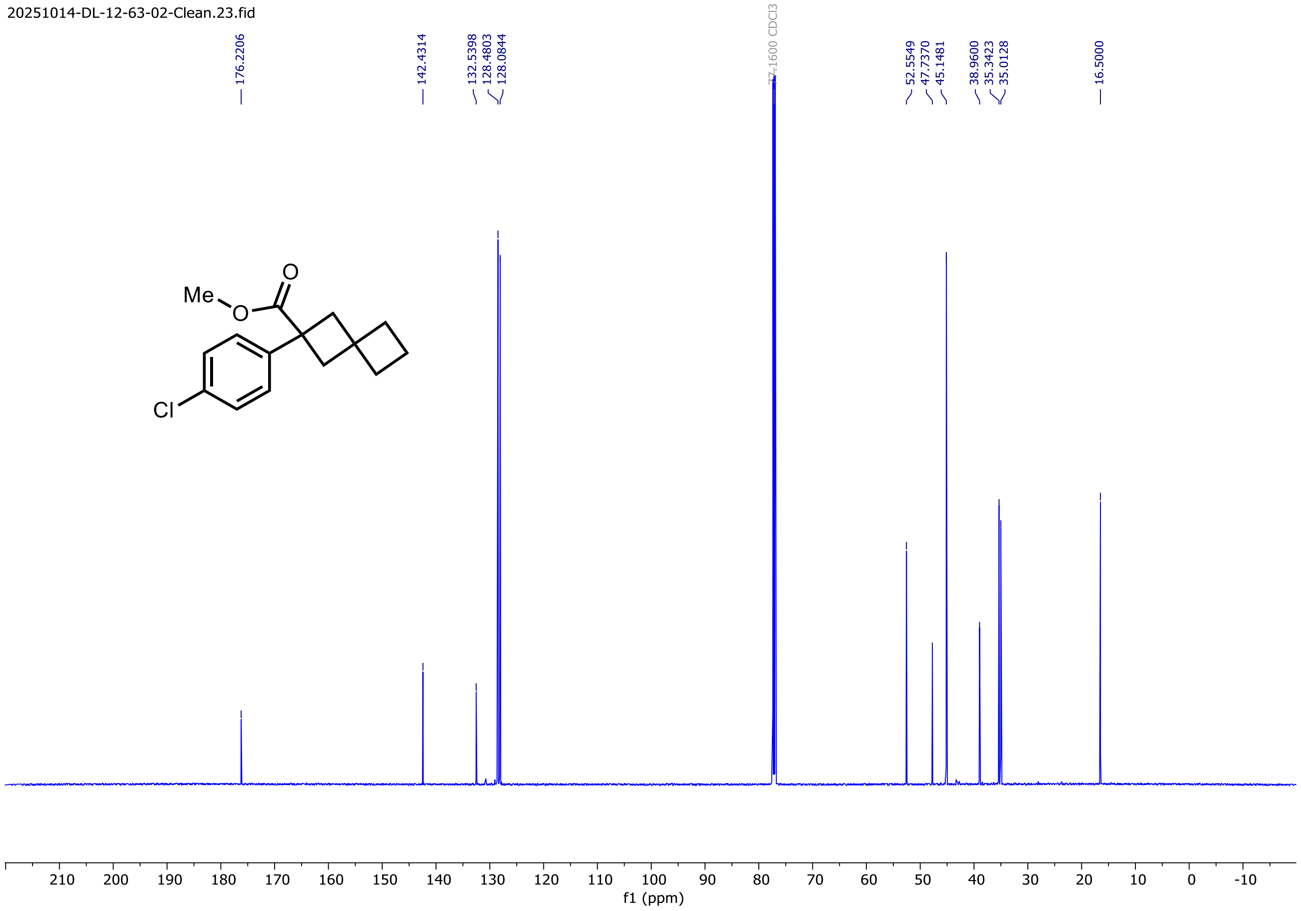
20251014-DL-12-63-02-Clean.23.fid
176.2206
142.4314
132.5398
128.4803
128.0844
77.1600 CDCl3
52.5549
47.7370
45.1481
38.9600
35.3423
35.0128
16.5000
Me
O
O
Cl
210
200
190
180
170
160
150
140
130
120
110
100
90
80
70
60
50
40
30
20
10
0
-10
f1 (ppm)

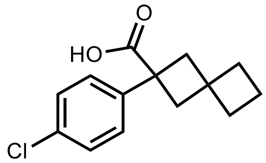

2-(4-chlorophenyl)spiro[3.3]heptane-2-carboxylic acid (Compound S2)

R_f (1Hex/1Et2O) = 0.20 (UV 254 nm)

^{1}H NMR (600 MHz, $CDCl_3$) δ 11.84 (s, 1H), 7.30 (d, *J* = 8.0 Hz, 2H), 7.24 (d, *J* = 8.0 Hz, 2H), 2.94 (d, *J* = 12.4 Hz, 2H), 2.50 (d, *J* = 12.4 Hz, 2H), 2.09 (t, *J* = 7.4 Hz, 2H), 1.90 – 1.85 (m, 2H), 1.85 – 1.78 (m, 2H).

^{13}C NMR (151 MHz, $CDCl_3$) δ 182.2, 141.8, 132.9, 128.5, 128.3, 47.5, 45.0, 38.9, 35.4, 35.1, 16.4.

HRMS (+p APCI) calcd. for [$C_{14}H_{15}O_2{}^{35}Cl$] ([M+H]$^+$) 250.0766 found 250.07571.

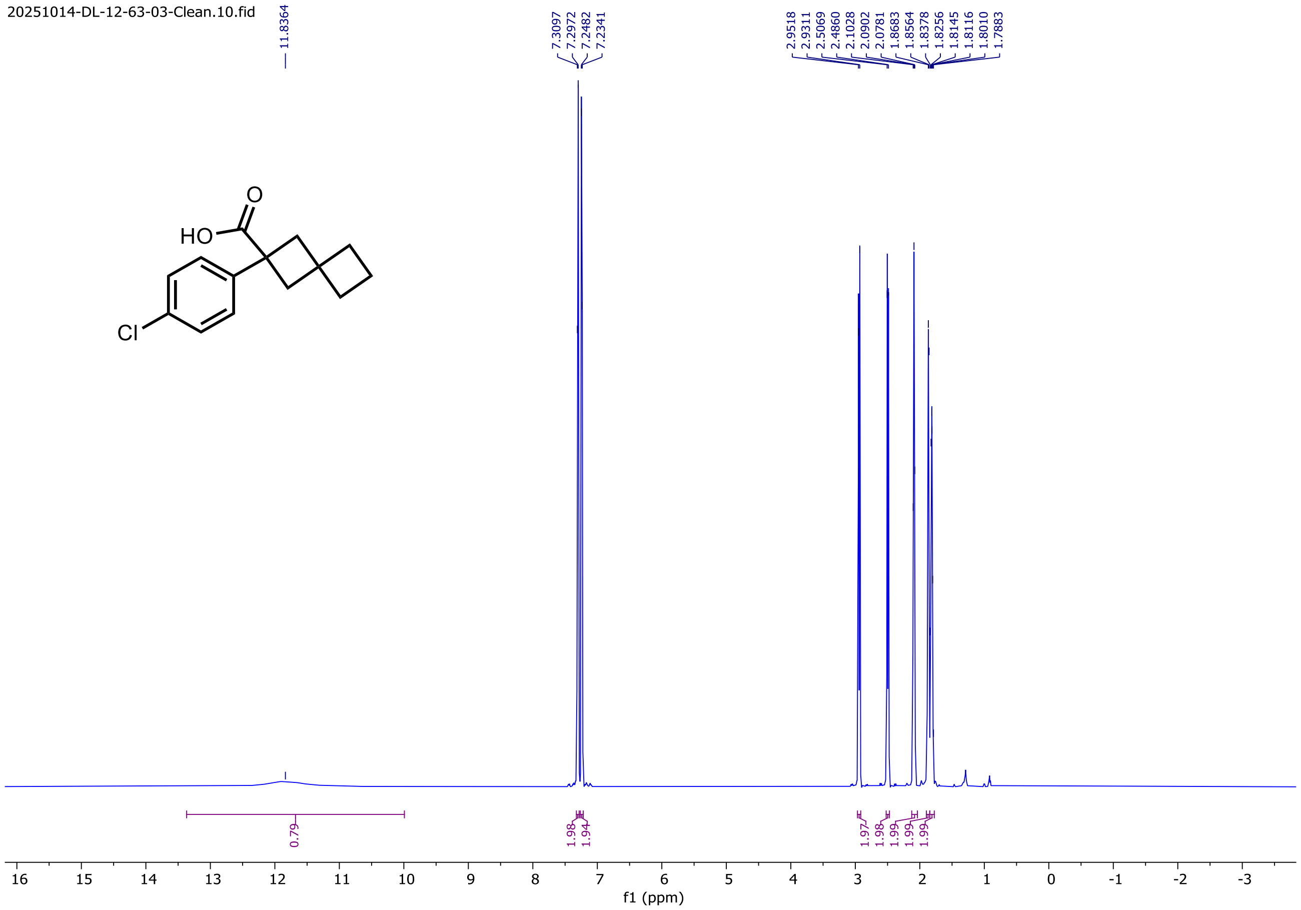
20251014-DL-12-63-03-Clean.10.fid
11.8364
7.3097
7.2972
7.2482
7.2341
2.9518
2.9311
2.5069
2.4860
2.1028
2.0902
2.0781
1.8683
1.8564
1.8378
1.8256
1.8145
1.8116
1.8010
1.7883
HO
O
Cl
0.79
1.98
1.94
1.97
1.98
1.99
1.99
1.99
f1 (ppm)

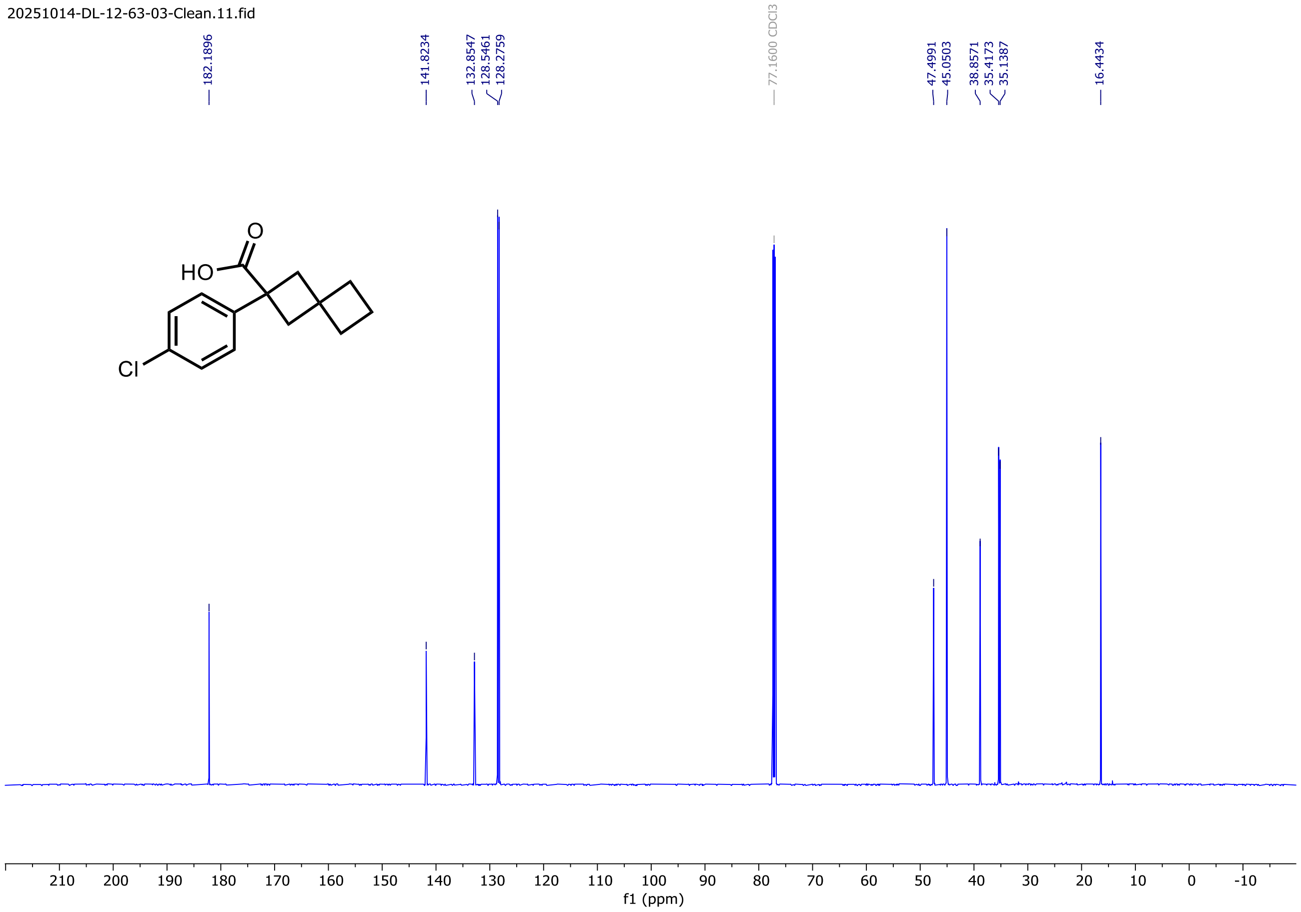
20251014-DL-12-63-03-Clean.11.fid
182.1896
141.8234
132.8547
128.5461
128.2759
77.1600 CDCl3
47.4991
45.0503
38.8571
35.4173
35.1387
16.4434
HO
O
Cl
f1 (ppm)

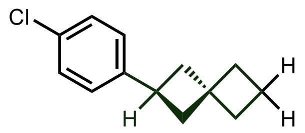

2-(4-chlorophenyl)spiro[3.3]heptane (Compound 16)

R_f (hexane) = 0.50 (UV 254 nm)

^{1}H NMR (600 MHz, $CDCl_3$) δ 7.24 (d, *J* = 8.4 Hz, 2H), 7.10 (d, *J* = 8.4 Hz, 2H), 3.33 (p, *J* = 8.4 Hz, 1H), 2.40 (td, *J* = 8.4, 2.8 Hz, 2H), 2.13 (t, *J* = 8.4 Hz, 2H), 2.02 (td, *J* = 9.3, 2.8 Hz, 2H), 1.93 – 1.89 (m, 2H), 1.88 – 1.80 (m, 2H).

^{13}C NMR (151 MHz, $CDCl_3$) δ 144.7, 131.3, 128.3, 127.9, 42.4, 39.8, 35.4, 34.3, 34.1, 16.7.

HRMS (+p APCI) calcd. for [$C_{13}H_{15}{}^{35}Cl$] ([M+H]$^+$) 206.0857 found 206.0855.

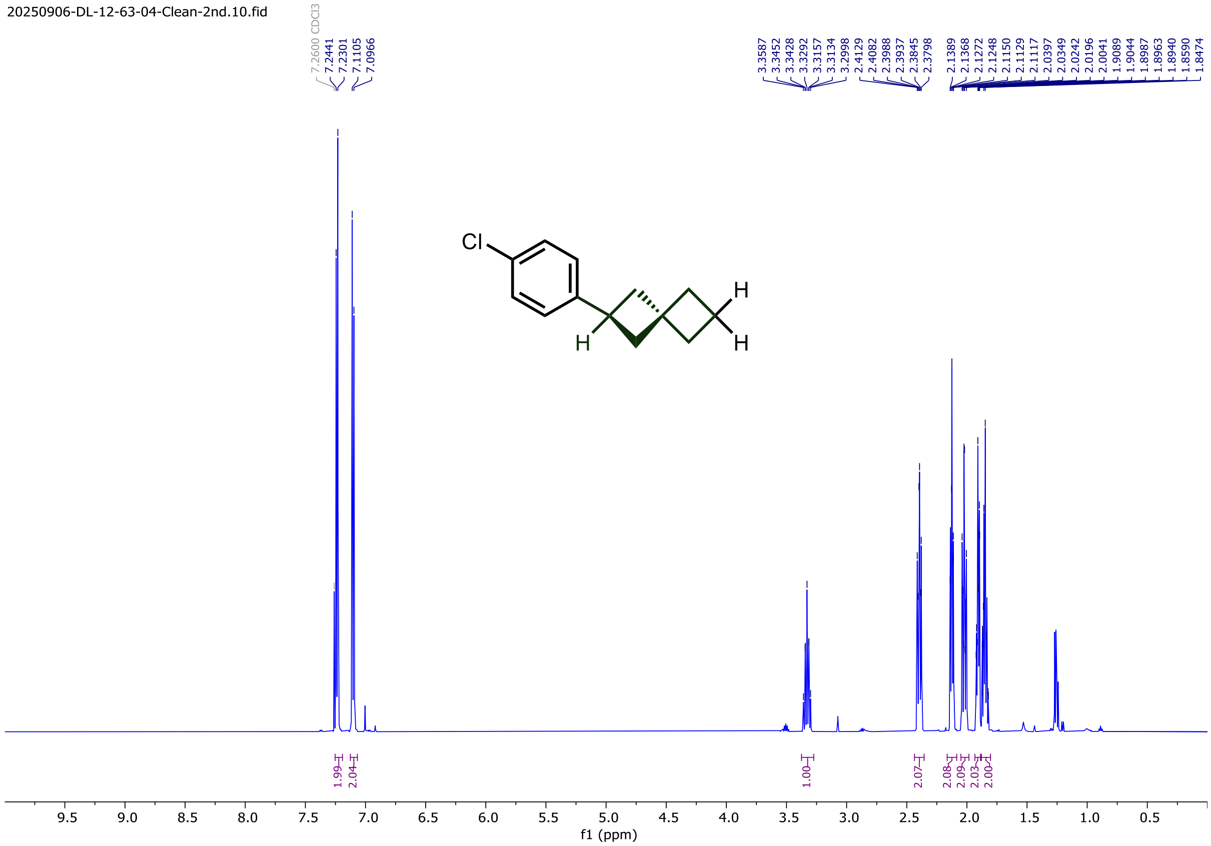
20250906-DL-12-63-04-Clean-2nd.10.fid
7.2600 CDCl3
7.2441
7.2301
7.1105
7.0966
3.3587
3.3452
3.3428
3.3292
3.3157
3.3134
3.2998
2.4129
2.4082
2.3988
2.3937
2.3845
2.3798
2.1389
2.1368
2.1272
2.1248
2.1150
2.1129
2.1117
2.0397
2.0349
2.0242
2.0196
2.0041
1.9089
1.9044
1.8987
1.8963
1.8940
1.8590
1.8474
Cl
H
H
H
1.99
2.04
1.00
2.07
2.08
2.09
2.03
2.00
f1 (ppm)

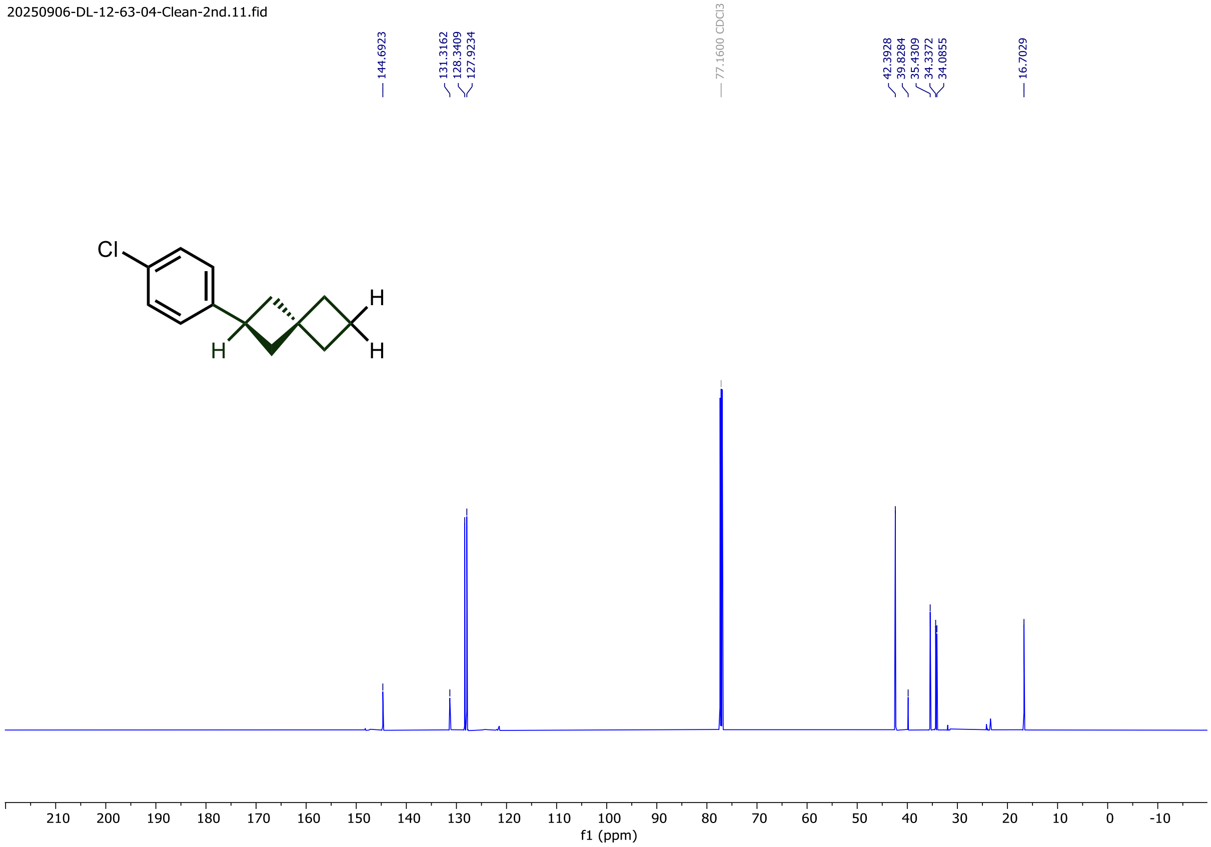
20250906-DL-12-63-04-Clean-2nd.11.fid
144.6923
131.3162
128.3409
127.9234
77.1600 CDCl3
42.3928
39.8284
35.4309
34.3372
34.0855
16.7029
Cl
H
H
H
f1 (ppm)

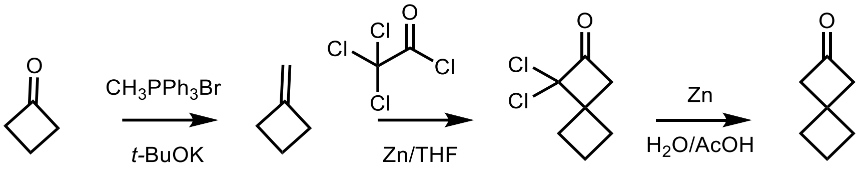

Step 1. To oven dried 250 ml round bottom flask was added methyltriphenylphosphonium bromide (38.2 g, 107 mmol, 1.5 equiv) and dry THF (140 ml, 0.5 M). The suspension was cool down to 0 ºC by an ice-bath followed by an addition of potassium tert-butoxide (12.0 g, 107 mmol, 1.5 eq). The mixture was kept stirring at 0 ºC for 2 hours before a solution of cyclobutanone (5.0 g, 71.3 mmol, 1.0 equiv) in THF (10 ml) was added. The reaction mixture warmed up to room temperature and kept stirring overnight. A clear solution of methylenecyclobutane in THF was obtained by simple distillation of the crude mixture at 90 ºC under ambient pressure.

Step 2. Zinc powder (6.53 g, 100 mmol, 1.4 equiv) was added to the solution of methylenecyclobutane in THF obtained above. The mixture was cooled to 0 ºC in an ice bath, before trichloroacetic acid chloride (10.4 ml, 16.9 g, 92.7 mmol, 1.3 equiv) was added dropwise to the reaction mixture. The reaction was kept stirred in an ice bath for 1 hour before warming up to room temperature. After 5 hours, the crude mixture was filtered through celite and washed by 20 ml of diethyl ether. The solvent was removed under vacuum to provide a dark yellowish oil crude.

Step 3. To the above crude mixture 144 ml of acetic acid and 213 ml of water was added. The mixture was cooled in an ice-bath followed by an addition of zinc powder (13.1 g, 200 mmol, 2.8 equiv). The mixture was let stirred overnight. Upon completion, the mixture was extract with pentane (200ml×2). The pentane layer was then washed with water then $NaHCO_3$(aq, sat), and brine. The pentane layer was then dried over Na_2SO_4. The solvent was removed under vacuum to provide spiro[3.3]heptan-2-one (6.1 g, 78% yield over 3 steps) as a slight yellowish clear oil. The product was used for the subsequent steps without further purification.

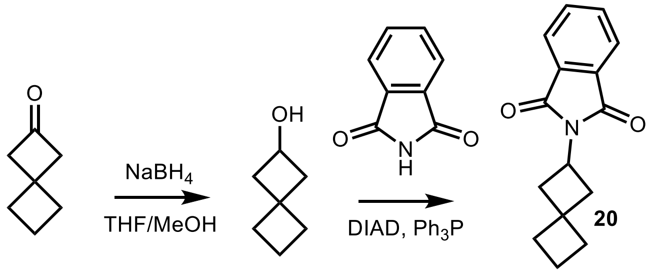

20

Step 1. Spiro[3.3]heptan-2-one (3.10 g, 28.1 mmol, 1.0 equiv) was added in methanol (50 mL), cooled with ice-bath, sodium borohydride (1.17 g, 31 mmol, 1.1 equiv) was slowly added in batches. The mixture was stirred at 0 ºC for 1 hour then at room temperature for another hour, then heated to 25°C and reacted for 2h. After completion of the reaction, the reaction was quenched by adding water and

extract with ethyl acetate. The organic layer was then washed sequentially with water (30 mL) and saturated brine (30 mL) followed by drying over anhydrous sodium sulfate. The solvent was removed under vacuum to provide a colorless oil product.

Step 2. To the above crude mixture was added phthalimide (4.13 g, 28.1 mmol, 1 equiv) and triphenylphosphine (14.7 g, 56.2 mmol, 2 equiv) were dissolved in dry tetrahydrofuran (100 mL). DIAD (11.1 ml, 11.3 g, 56.2 mmol, 2 equiv) was added dropwise under ice-water bath. After addition, the reaction was warmed up to 25°C and reacted for 4h, ethyl acetate (150 mL) was added, washed with saturated brine (100 mL), the organic phase was dried over anhydrous sodium sulfate, concentrated in vacuum. The crude product was purified by flash chromatography (SiO_2, 0-15% diethyl ether in hexane) to obtain 2-(spiro[3.3]heptan-2-yl)isoindoline-1,3-dione **20** (**compound 20**)(2.50 g, 37% yield) as a white solid.

R_f (1Hex/2Et_2O) = 0.60 (UV)

^{1}H NMR (400 MHz, $CDCl_3$) δ 7.80 (dd, J = 5.5, 3.0 Hz, 2H), 7.68 (dd, J = 5.4, 3.0 Hz, 2H), 4.59 (tt, J = 9.6, 8.2 Hz, 1H), 2.86 (td, J = 9.3, 2.9 Hz, 2H), 2.33 (td, J = 8.4, 2.8 Hz, 2H), 2.15 – 2.03 (m, 4H), 1.92 – 1.79 (m, 2H). The ^{1}H-NMR matched the reported literature.[2]

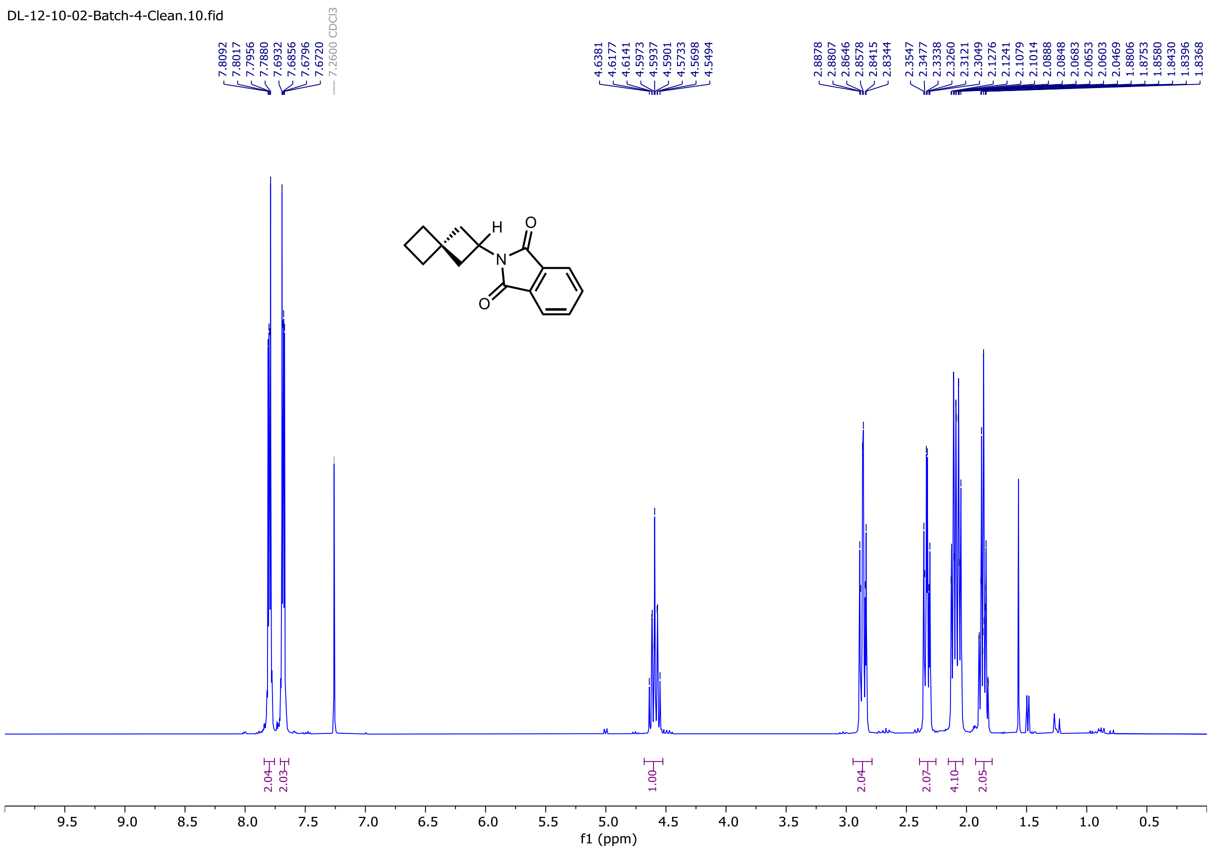

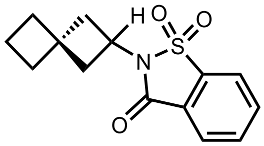

2-(spiro[3.3]heptan-2-yl)benzo[d]isothiazol-3(2H)-one 1,1-dioxide (Compound S4)

This compound was synthesized according to the above procedure using benzo[d]isothiazol-3(2H)-one 1,1-dioxide. The product was purified by flash chromatography (SiO_2, 0-30% ethyl acetate in hexane) and obtained **compound S4** (239.0 mg, 43%, 2.0 mmol scale) as a white solid.

R_f (2Hex/1EA) = 0.40 (UV 254 nm)

^{1}H NMR (600 MHz, $CDCl_3$) δ 7.87 (d, *J* = 7.5 Hz, 1H), 7.77 – 7.71 (m, 2H), 7.71 – 7.66 (m, 1H), 5.27 (p, *J* = 7.3 Hz, 1H), 2.66 (ddd, *J* = 10.0, 7.1, 3.0 Hz, 2H), 2.26 (ddd, *J* = 10.2, 7.4, 3.0 Hz, 2H), 2.13 – 2.00 (m, 4H), 1.92 – 1.83 (m, 2H).

^{13}C NMR (151 MHz, $CDCl_3$) δ 168.3, 143.6, 134.1, 133.5, 127.3, 123.4, 122.0, 73.1, 42.6, 36.3, 34.6, 34.6, 17.1.

HRMS (+p APCI) calcd. for [$C_{14}H_{16}O_3N^{32}S$] ([M+H]$^+$) 278.0845 found 278.0847.

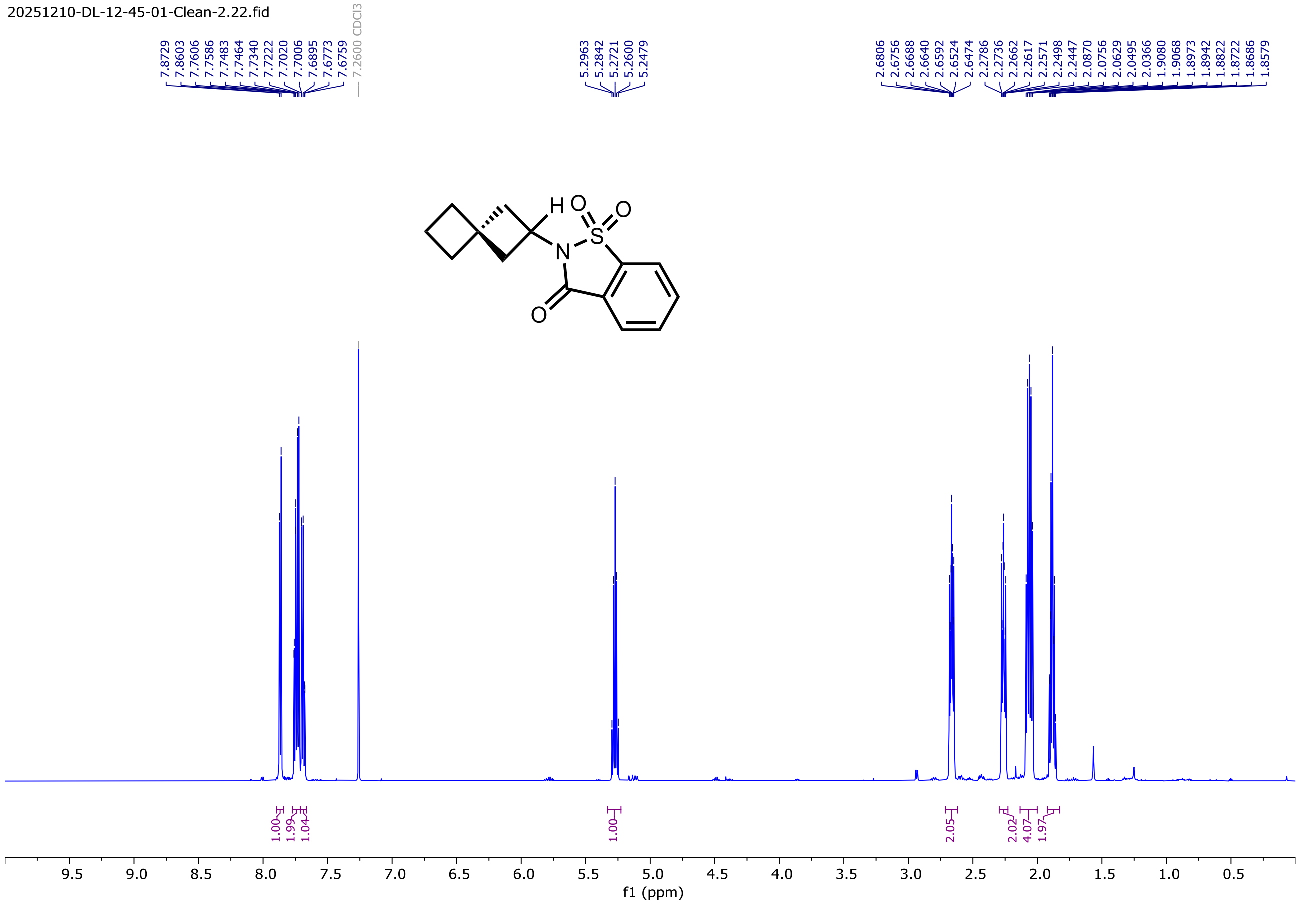
20251210-DL-12-45-01-Clean-2.22.fid
7.8729
7.8603
7.7606
7.7586
7.7483
7.7464
7.7340
7.7222
7.7020
7.7006
7.6895
7.6773
7.6759
7.2600 CDCl3
5.2963
5.2842
5.2721
5.2600
5.2479
2.6806
2.6756
2.6688
2.6640
2.6592
2.6524
2.6474
2.2786
2.2736
2.2662
2.2617
2.2571
2.2498
2.2447
2.0870
2.0756
2.0629
2.0495
2.0366
1.9080
1.9068
1.8973
1.8942
1.8822
1.8722
1.8686
1.8579
H
O
O
S
N
O
1.00
1.99
1.04
1.00
2.05
2.02
4.07
1.97
9.5
9.0
8.5
8.0
7.5
7.0
6.5
6.0
5.5
5.0
4.5
4.0
3.5
3.0
2.5
2.0
1.5
1.0
0.5
f1 (ppm)

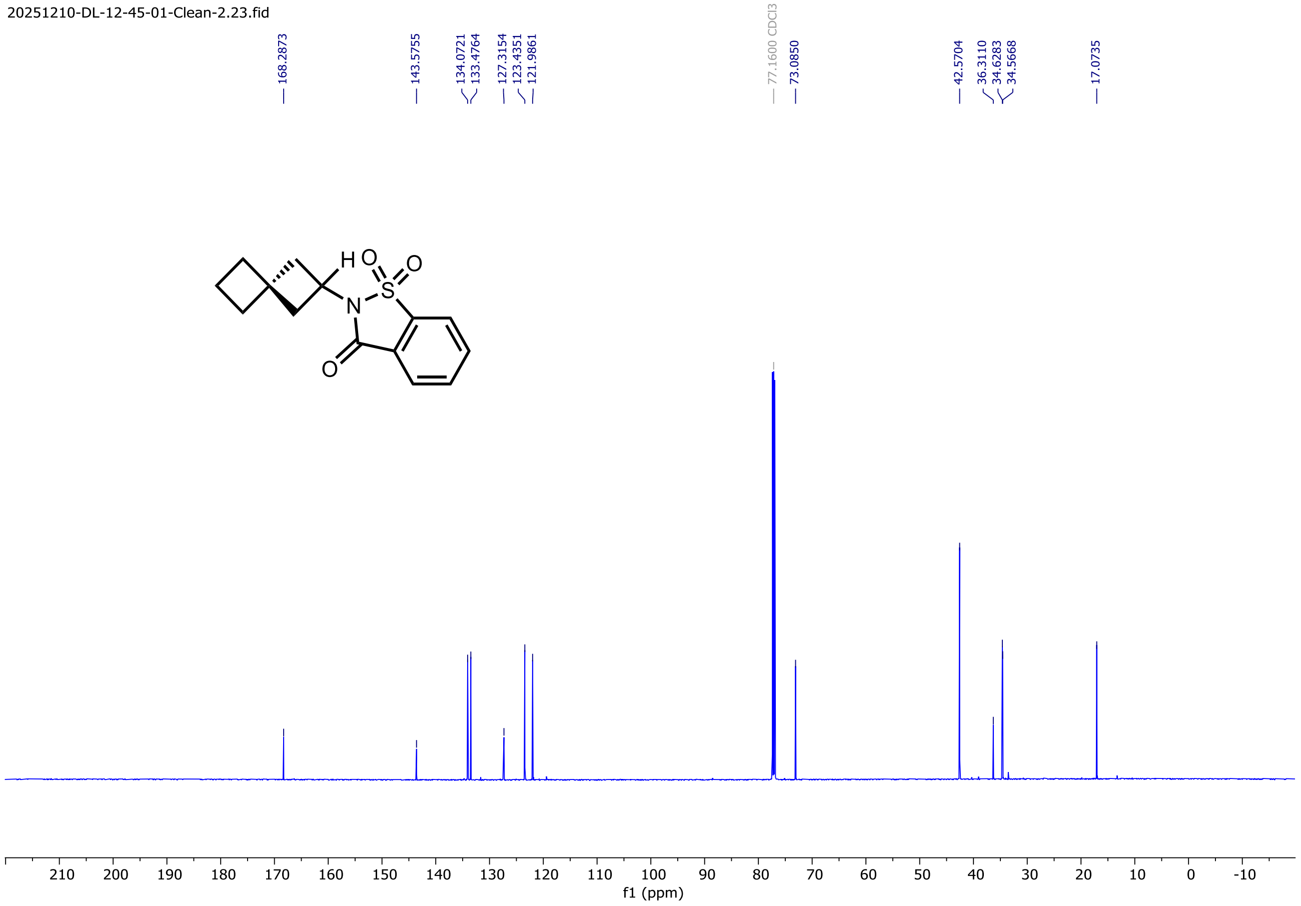
20251210-DL-12-45-01-Clean-2.23.fid
168.2873
143.5755
134.0721
133.4764
127.3154
123.4351
121.9861
77.1600 CDCl3
73.0850
42.5704
36.3110
34.6283
34.5668
17.0735
H
O
O
S
N
O
210
200
190
180
170
160
150
140
130
120
110
100
90
80
70
60
50
40
30
20
10
0
-10
f1 (ppm)

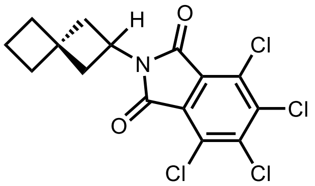

4,5,6,7-tetrachloro-2-(spiro[3.3]heptan-2-yl)isoindoline-1,3-dione (Compound S5)

This compound was synthesized according to the above procedure using 4,5,6,7-tetrachloroisoindoline-1,3-dione. The product was purified by flash chromatography (SiO_2, 0-20% diethyl ether in hexane) and obtained **compound S5** (510.0 mg, 45%, 3.0 mmol scale) as a white solid.

R_f (1Hex/1Et2O) = 0.60 (UV 254 nm)

^{1}H NMR (600 MHz, $CDCl_3$) δ 4.67 – 4.58 (m, 1H), 2.86 (td, *J* = 9.4, 2.8 Hz, 2H), 2.37 (td, *J* = 8.5, 2.8 Hz, 2H), 2.13 (t, *J* = 7.6 Hz, 2H), 2.09 (t, *J* = 7.4 Hz, 2H), 1.92 – 1.83 (m, 2H).

^{13}C NMR (151 MHz, $CDCl_3$) δ 163.9, 140.1, 129.7, 127.6, 41.5, 39.6, 37.7, 35.0, 34.5, 16.9.

HRMS (+p APCI) calcd. for [$C_{15}H_{12}O_2N^{35}Cl_4$] ($[M+H]^+$) 377.9617 found 377.9621.

20251210-DL-12-34-06-Clean-2.10.fid

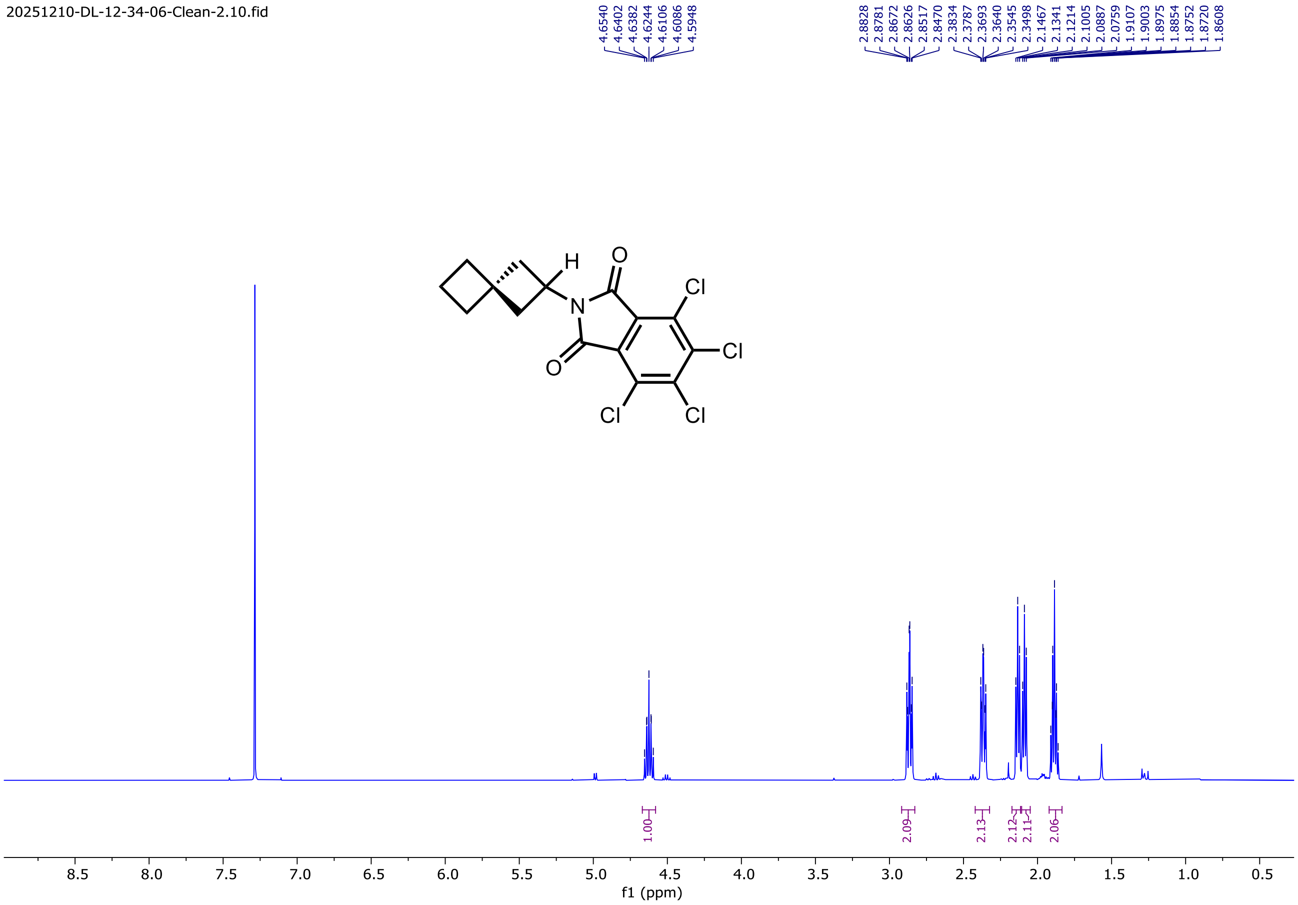

20251210-DL-12-34-06-Clean-2.11.fid

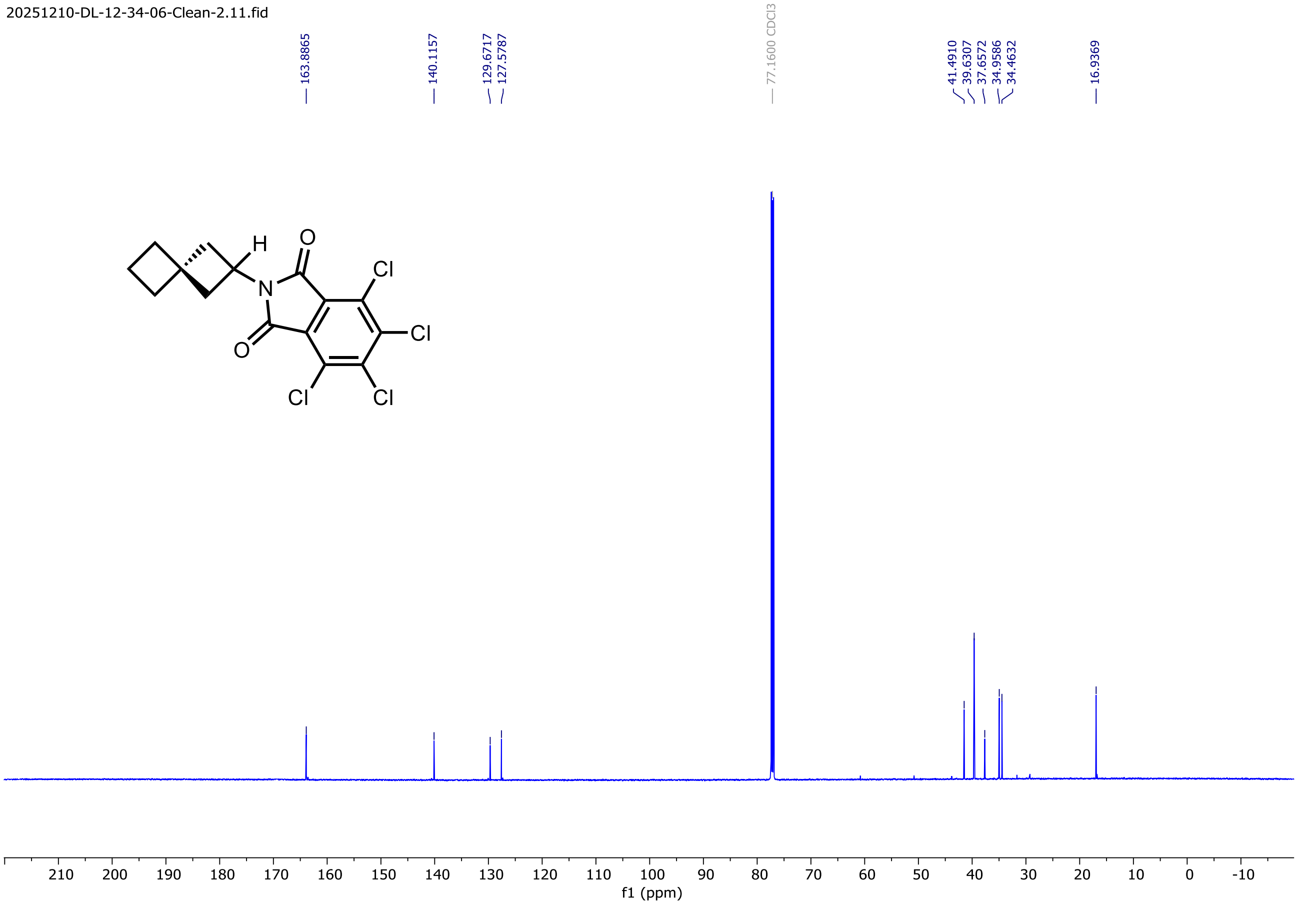

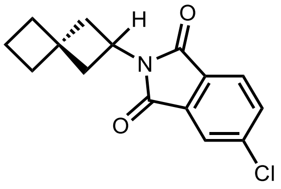

5-chloro-2-(spiro[3.3]heptan-2-yl)isoindoline-1,3-dione (Compound S6)

This compound was synthesized according to the above procedure using 5-chloroisoindoline-1,3-dione. The product was purified by flash chromatography (SiO_2, 0-20% diethyl ether in hexane) and obtained **compound S6** (100 mg, 20% yield, 1.7 mmol scale) as a white solid.

R_f (1Hex/1Et2O) = 0.60 (UV 254 nm)

^{1}H NMR (600 MHz, $CDCl_3$) δ 7.76 (d, J = 1.8 Hz, 1H), 7.73 (d, J = 7.9 Hz, 1H), 7.65 (dd, J = 7.9, 1.8 Hz, 1H), 4.57 (tt, J = 9.4, 8.2 Hz, 1H), 2.83 (td, J = 9.4, 2.9 Hz, 2H), 2.33 (td, J = 8.2, 2.9 Hz, 2H), 2.10 (t, J = 7.8 Hz, 2H), 2.06 (t, J = 7.8 Hz, 2H), 1.86 (p, J = 7.8 Hz, 2H).

^{13}C NMR (151 MHz, $CDCl_3$) δ 167.76, 167.42, 140.66, 134.04, 133.83, 130.21, 124.43, 123.64, 40.95, 39.84, 37.70, 34.98, 34.35, 16.97.

HRMS (+p APCI) calcd. for [$C_{15}H_{15}O_2N^{35}Cl$] ($[M+H]^+$) 276.0786 found 276.0789.

20250919-DL-12-70-01-Clean.52.fid

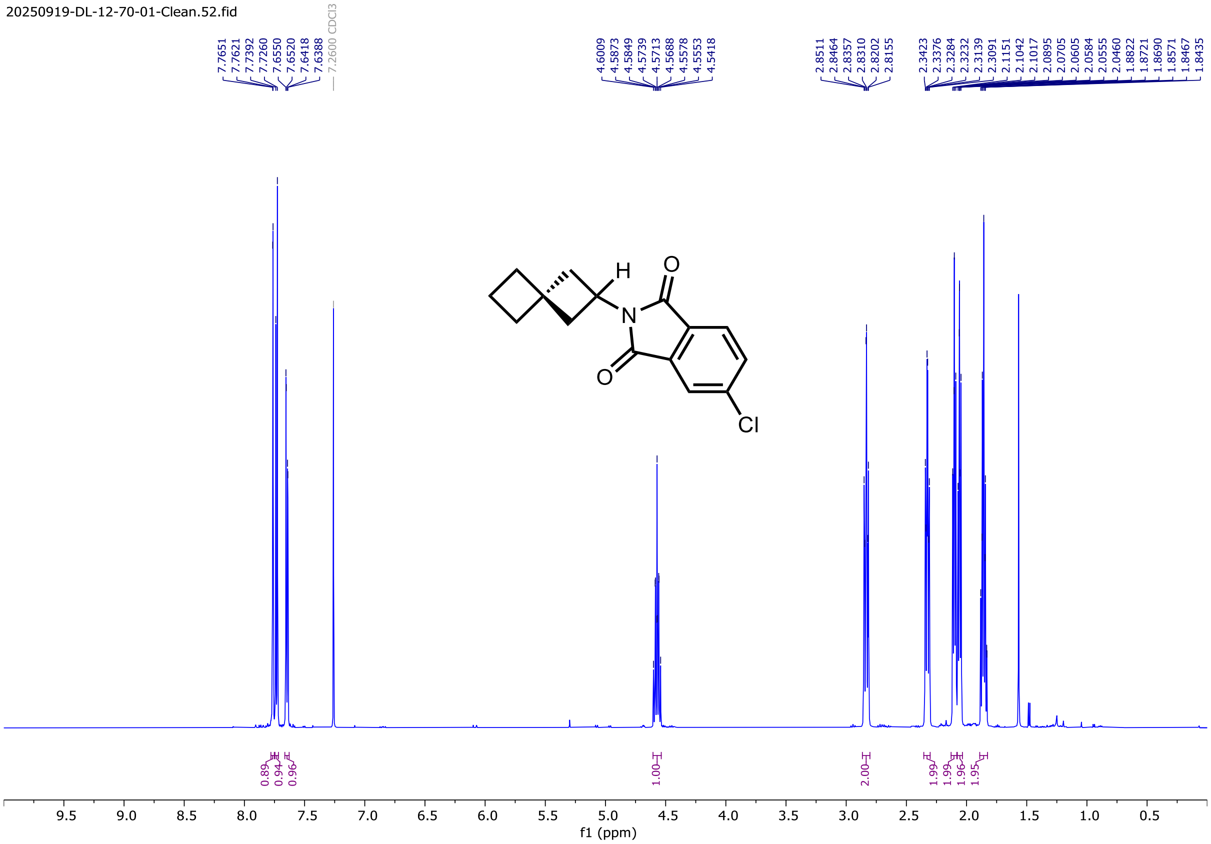

20250919-DL-12-70-01-Clean.53.fid

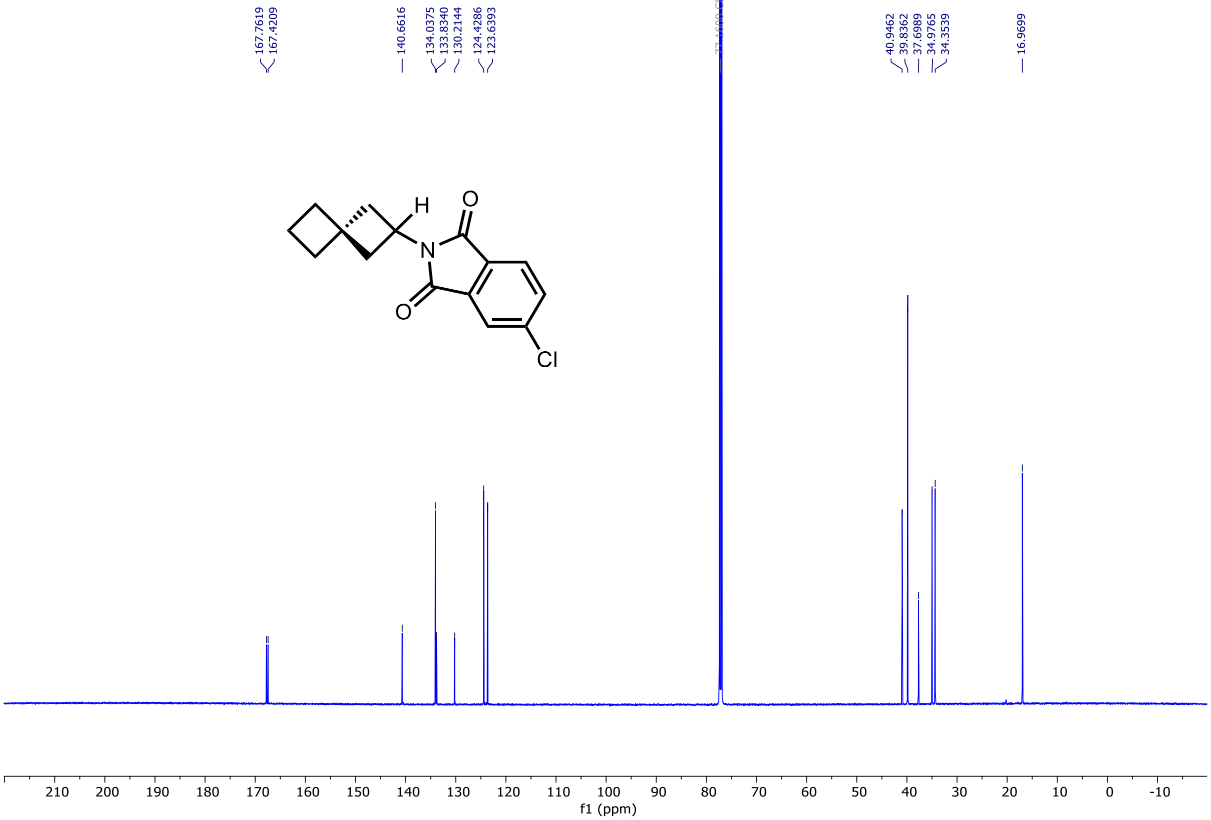

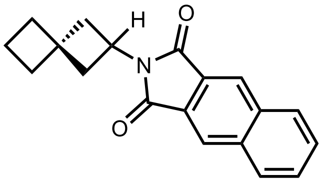

2-(spiro[3.3]heptan-2-yl)-1H-benzo[f]isoindole-1,3(2H)-dione (Compound S7)

This compound was synthesized according to the above procedure using 1H-benzo[f]isoindole-1,3(2H)-dione. The product was purified by flash chromatography (SiO_2, 0-20% diethyl ether in hexane) and obtained **compound S7** (174 mg, 34% yield, 1.7 mmol scale) as a white solid.

R$_f$ (1Hex/1Et2O) = 0.60 (UV 254 nm)

^{1}H NMR (600 MHz, CDCl$_3$) δ 8.28 (s, 2H), 8.03 (dd, *J* = 6.2, 3.2 Hz, 2H), 7.67 (dd, *J* = 6.2, 3.2 Hz, 2H), 4.68 (tt, *J* = 9.4, 8.3 Hz, 1H), 2.93 (td, *J* = 9.4, 2.8 Hz, 2H), 2.36 (td, *J* = 8.3, 2.8 Hz, 2H), 2.13 (t, *J* = 7.5 Hz, 2H), 2.09 (t, *J* = 7.5 Hz, 2H), 1.91 – 1.83 (m, 2H).

^{13}C NMR (151 MHz, CDCl$_3$) δ 168.5, 135.6, 130.4, 129.2, 128.0, 124.5, 41.0, 39.8, 37.8, 35.1, 34.4, 17.0.

HRMS (+p APCI) calcd. for [$C_{19}H_{18}O_2N$] ([M+H]$^+$) 292.1332 found 292.1335.

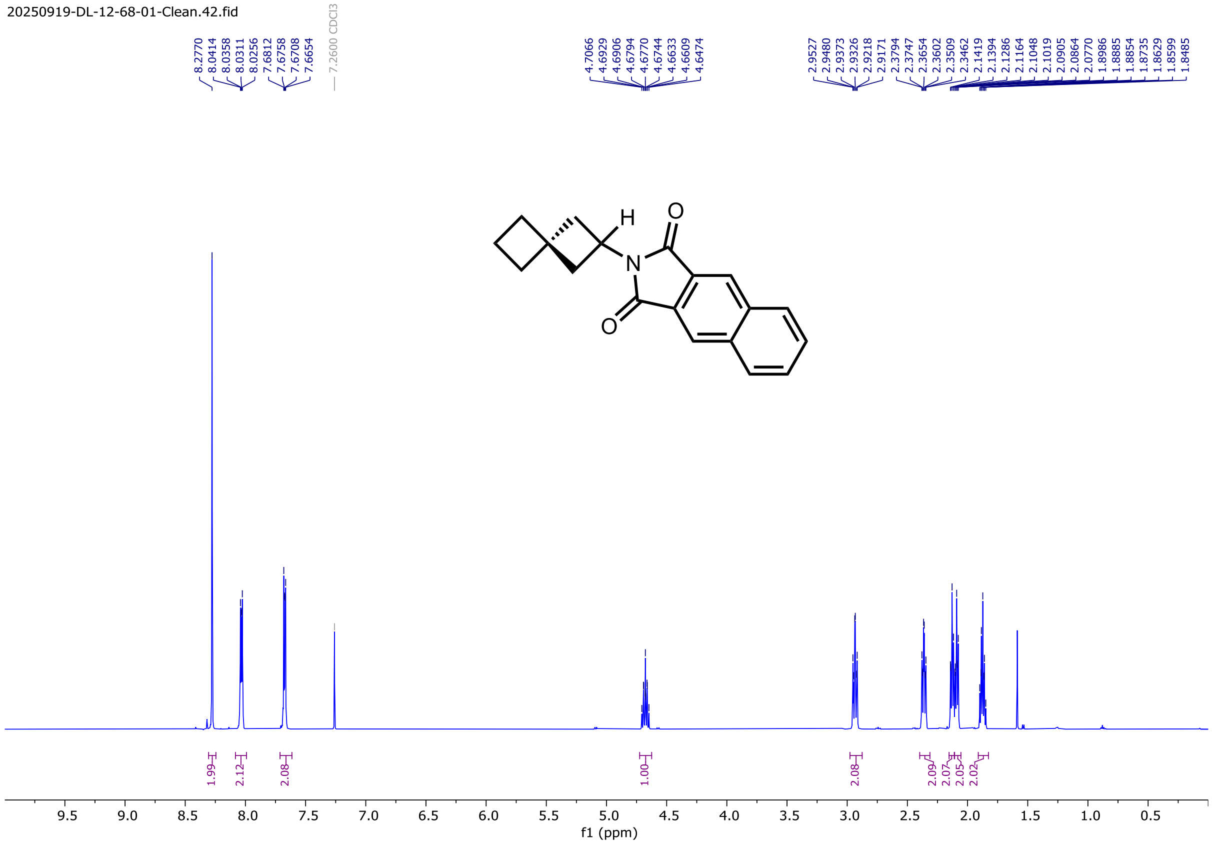
20250919-DL-12-68-01-Clean.42.fid
8.2770
8.0414
8.0358
8.0311
8.0256
7.6812
7.6758
7.6708
7.6654
7.2600 CDCl3
4.7066
4.6929
4.6906
4.6794
4.6770
4.6744
4.6633
4.6609
4.6474
2.9527
2.9480
2.9373
2.9326
2.9218
2.9171
2.3794
2.3747
2.3654
2.3602
2.3509
2.3462
2.1419
2.1394
2.1286
2.1164
2.1048
2.1019
2.0905
2.0864
2.0770
1.8986
1.8885
1.8854
1.8735
1.8629
1.8599
1.8485
H
O
N
O
1.99
2.12
2.08
1.00
2.08
2.09
2.07
2.05
2.02
9.5
9.0
8.5
8.0
7.5
7.0
6.5
6.0
5.5
5.0
4.5
4.0
3.5
3.0
2.5
2.0
1.5
1.0
0.5
f1 (ppm)

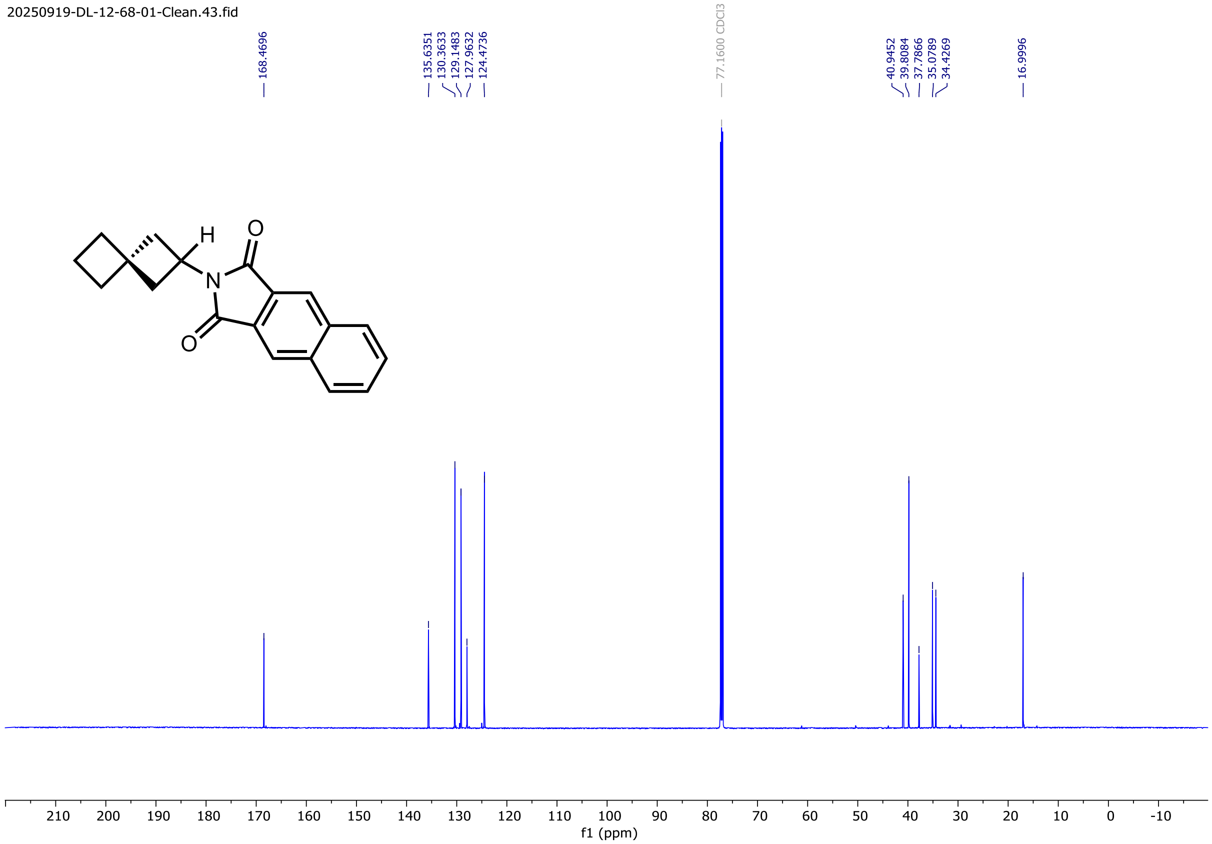
20250919-DL-12-68-01-Clean.43.fid
168.4696
135.6351
130.3633
129.1483
127.9632
124.4736
77.1600 CDCl3
40.9452
39.8084
37.7866
35.0789
34.4269
16.9996
H
O
N
O
210
200
190
180
170
160
150
140
130
120
110
100
90
80
70
60
50
40
30
20
10
0
-10
f1 (ppm)

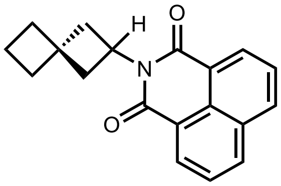

2-(spiro[3.3]heptan-2-yl)-1H-benzo[de]isoquinoline-1,3(2H)-dione (Compound S8)

This compound was synthesized according to the above procedure using 1H,3H-benzo[de]isochromene-1,3-dione. The product was purified by flash chromatography (SiO_2, 0-20% diethyl ether in hexane) and obtained **compound S8** (320.0 mg, 62% yield, 1.7 mmol scale) as a white solid.

R_f (1Hex/1Et2O) = 0.60 (UV 254 nm)

^{1}H NMR (600 MHz, $CDCl_3$) δ 8.56 (d, J = 7.3 Hz, 2H), 8.18 (d, J = 7.7 Hz, 2H), 7.77 – 7.69 (m, 2H), 5.30 – 5.21 (m, 1H), 2.91 (td, J = 9.4, 2.7 Hz, 2H), 2.50 (td, J = 8.6, 2.7 Hz, 2H), 2.16 (t, J = 7.5 Hz, 2H), 2.10 (t, J = 7.5 Hz, 2H), 1.88 (p, J = 7.4 Hz, 2H).

^{13}C NMR (151 MHz, $CDCl_3$) δ 164.9, 133.7, 131.6, 131.1, 128.3, 127.1, 123.4, 43.9, 40.8, 37.9, 35.5, 35.0, 17.1.

HRMS (+p APCI) calcd. for [$C_{19}H_{18}O_2N$] ($[M+H]^+$) 292.1332 found 292.1336.

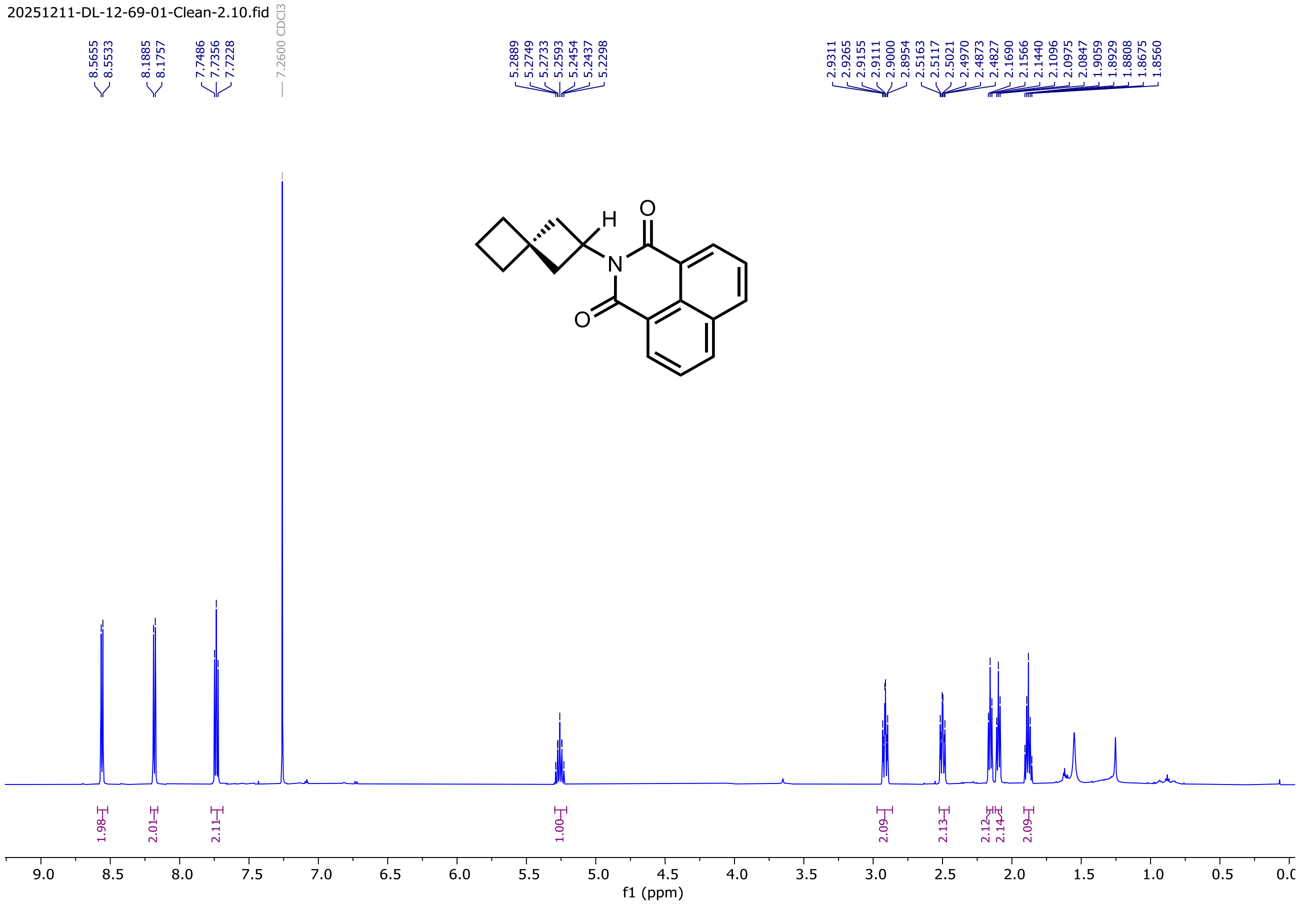
20251211-DL-12-69-01-Clean-2.10.fid
8.5655
8.5533
8.1885
8.1757
7.7486
7.7356
7.7228
7.2600 CDCl3
5.2889
5.2749
5.2733
5.2593
5.2454
5.2437
5.2298
2.9311
2.9265
2.9155
2.9111
2.9000
2.8954
2.5163
2.5117
2.5021
2.4970
2.4873
2.4827
2.1690
2.1566
2.1440
2.1096
2.0975
2.0847
1.9059
1.8929
1.8808
1.8675
1.8560
H
O
N
O
1.98
2.01
2.11
1.00
2.09
2.13
2.12
2.14
2.09
f1 (ppm)

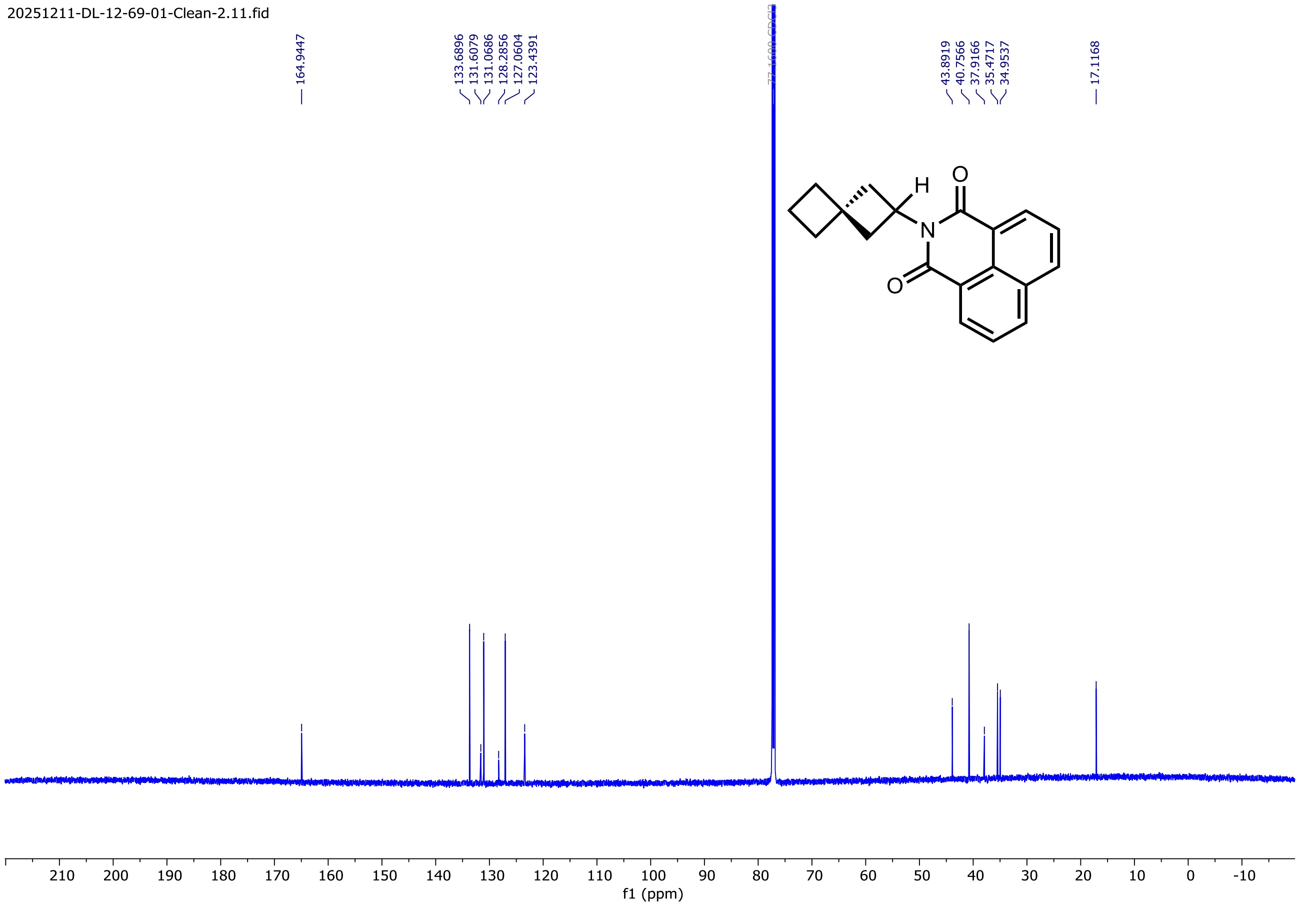
20251211-DL-12-69-01-Clean-2.11.fid
164.9447
133.6896
131.6079
131.0686
128.2856
127.0604
123.4391
43.8919
40.7566
37.9166
35.4717
34.9537
17.1168
H
O
N
O
f1 (ppm)

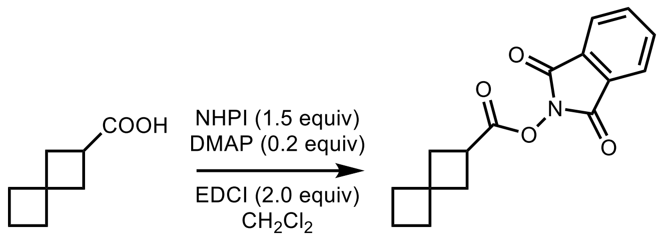

1,3-dioxoisoindolin-2-yl spiro[3.3]heptane-2-carboxylate (Compound S9)

spiro[3.3]heptane-2-carboxylic acid (421 mg, 3.0 mmol, 1.0 equiv), N-hydroxyphthalimide (734 mg, 4.5 mmol, 1.5 equiv) and DMAP (73.3 mg, 0.60 mmol, 0.2 equiv) were added to a 40 mL glass vial equipped with a stir bar. Anhydrous CH_2Cl_2 (20 mL) was then added to the vial followed by EDCI (1.15 g, 6.0 mmol, 2 equiv) at room temperature. The mixture was stirred for 12 hours at room temperature. The solvent was removed under reduced pressure. Purification using flash chromatography (SiO_2, 0-25% diethyl ether in hexane) afforded **compound S9** (1.02 g, 77% yield) as a white solid.

$\mathbf{R_f}$ (1Hex/1Et2O) = 0.50 (UV 254 nm)

^{1}H NMR (600 MHz, $CDCl_3$) δ 7.87 (dd, J = 5.5, 3.1 Hz, 2H), 7.78 (dd, J = 5.5, 3.1 Hz, 2H), 3.34 (p, J = 8.4 Hz ,1H), 2.50 – 2.33 (m, 4H), 2.07 (t, J = 7.8 Hz, 2H), 1.99 (t, J = 7.5 Hz, 2H), 1.87 – 1.76 (m, 2H).

^{13}C NMR (151 MHz, $CDCl_3$) δ 171.8, 162.2, 134.8, 129.1, 124.0, 40.9, 38.1, 35.1, 34.7, 30.4, 16.2.

HRMS (+p APCI) calcd. for [$C_{16}H_{16}O_4N$] ([M+H]$^+$) 286.1074 found 286.1062.

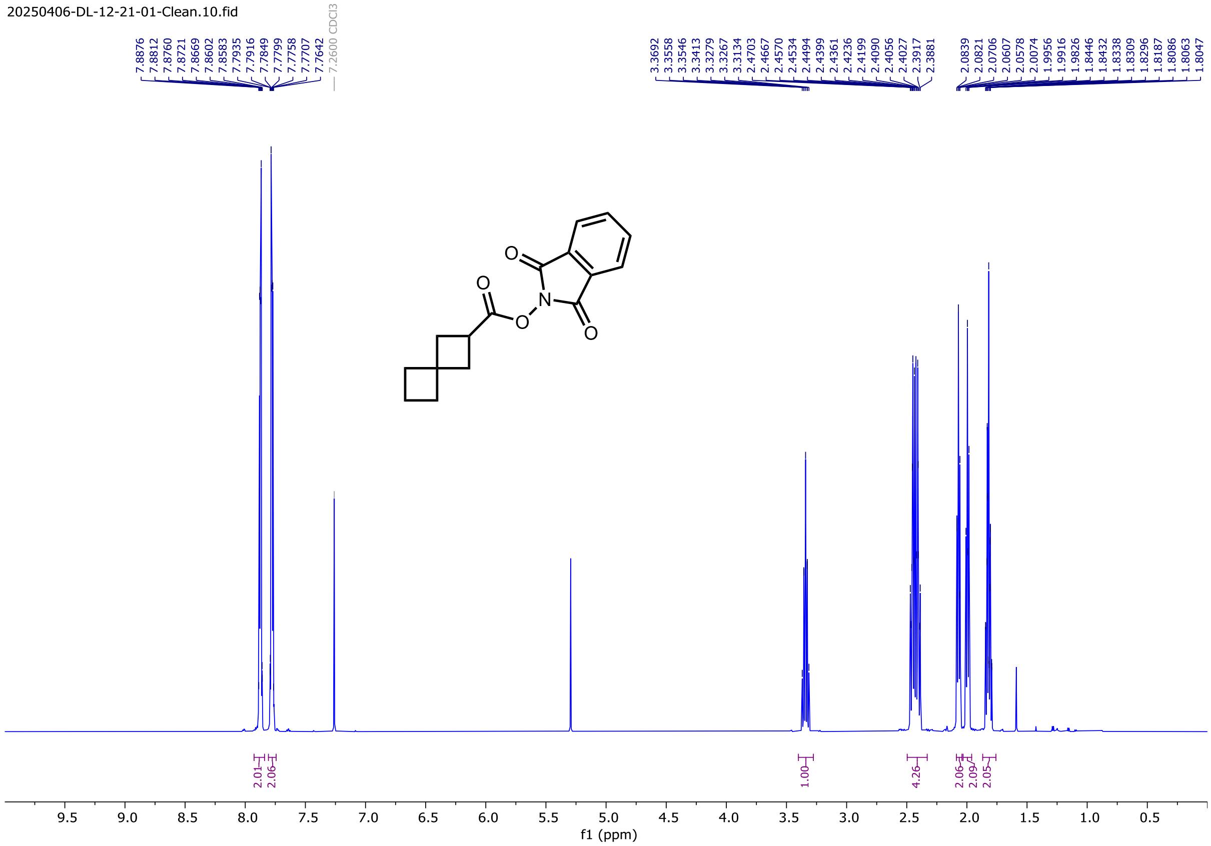
20250406-DL-12-21-01-Clean.10.fid
7.8876
7.8812
7.8760
7.8721
7.8669
7.8602
7.8583
7.7935
7.7916
7.7849
7.7799
7.7758
7.7707
7.7642
7.2600 CDCl3
3.3692
3.3558
3.3546
3.3413
3.3279
3.3267
3.3134
2.4703
2.4667
2.4570
2.4534
2.4494
2.4399
2.4361
2.4236
2.4199
2.4090
2.4056
2.4027
2.3917
2.3881
2.0839
2.0821
2.0706
2.0607
2.0578
2.0074
1.9956
1.9916
1.9826
1.8446
1.8432
1.8338
1.8309
1.8296
1.8187
1.8086
1.8063
1.8047
O
O
N
O
O
2.01
2.06
1.00
4.26
2.06
2.09
2.05
f1 (ppm)

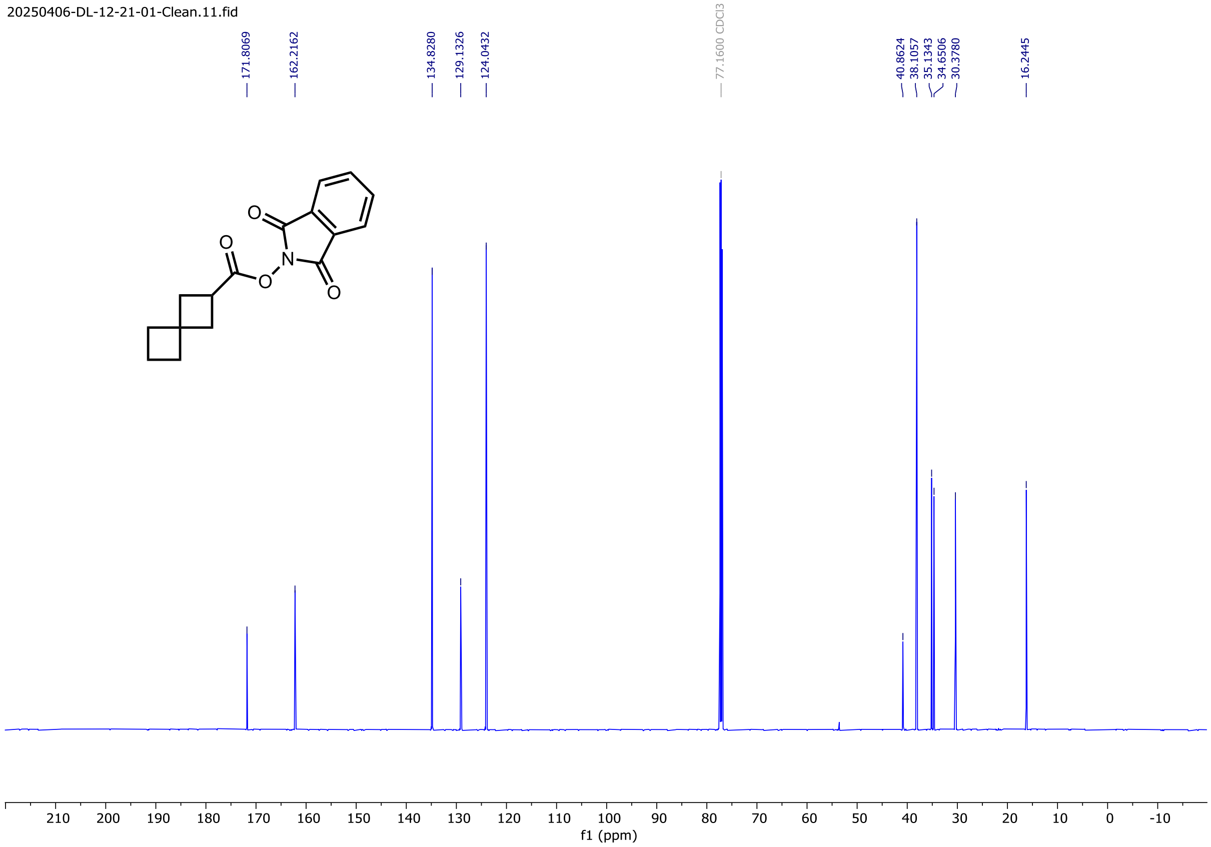
20250406-DL-12-21-01-Clean.11.fid
171.8069
162.2162
134.8280
129.1326
124.0432
77.1600 CDCl3
40.8624
38.1057
35.1343
34.6506
30.3780
16.2445
O
O
N
O
O
f1 (ppm)

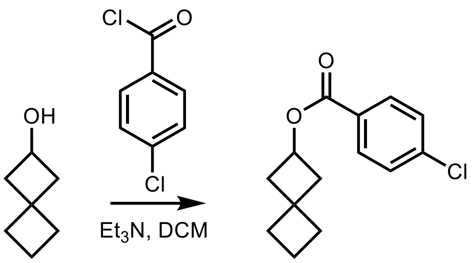

spiro[3.3]heptan-2-yl 4-chlorobenzoate (Compound S10)

To a 20 ml vial, was added spiro[3.3]heptan-2-ol (561 mg, 5.0 mmol, 1.0 equiv), DMAP (916 mg, 7.5 mmol, 1.5 equiv), Et_3N (0.70 ml, 506 mg, 5.0 mmol, 1.0 equiv), and CH_2Cl_2 (10 ml, 0.5 M). The mixture was cool to 0 °C by an ice bath. 4-chlorobenzoyl chloride (0.7 ml, 963 mg, 5.5 mmol, 1.1 equiv) was added to the above mixture. The mixture was stirred overnight. The mixture was then wash with 1.0N HCl (aq) and brine. The crude was purified by flash chromatography (SiO_2, 0-5% diethyl ether in hexane) to give **compound S10** (646 mg, 52% yield) as a clear oil.

R_f (9Hex/1Et2O) = 0.40 (UV 254 nm)

^{1}H NMR (600 MHz, $CDCl_3$) δ 7.95 (d, J = 8.5 Hz, 2H), 7.39 (d, J = 8.5 Hz, 2H), 5.10 (p, J = 7.3 Hz, 1H), 2.58 – 2.51 (m, 2H), 2.17 – 2.10 (m, 2H), 2.06 (t, J = 7.5 Hz, 2H), 2.01 (t, J = 7.4 Hz, 2H), 1.91 – 1.83 (m, 2H).

^{13}C NMR (151 MHz, $CDCl_3$) δ 165.4, 139.4, 131.1, 129.0, 128.8, 66.4, 42.7, 36.7, 34.9, 34.6, 17.1.

HRMS (+p APCI) calcd. for [$C_{14}H_{16}O_2{}^{35}Cl$] ($[M+H]^+$) 251.0833 found 251.0831.

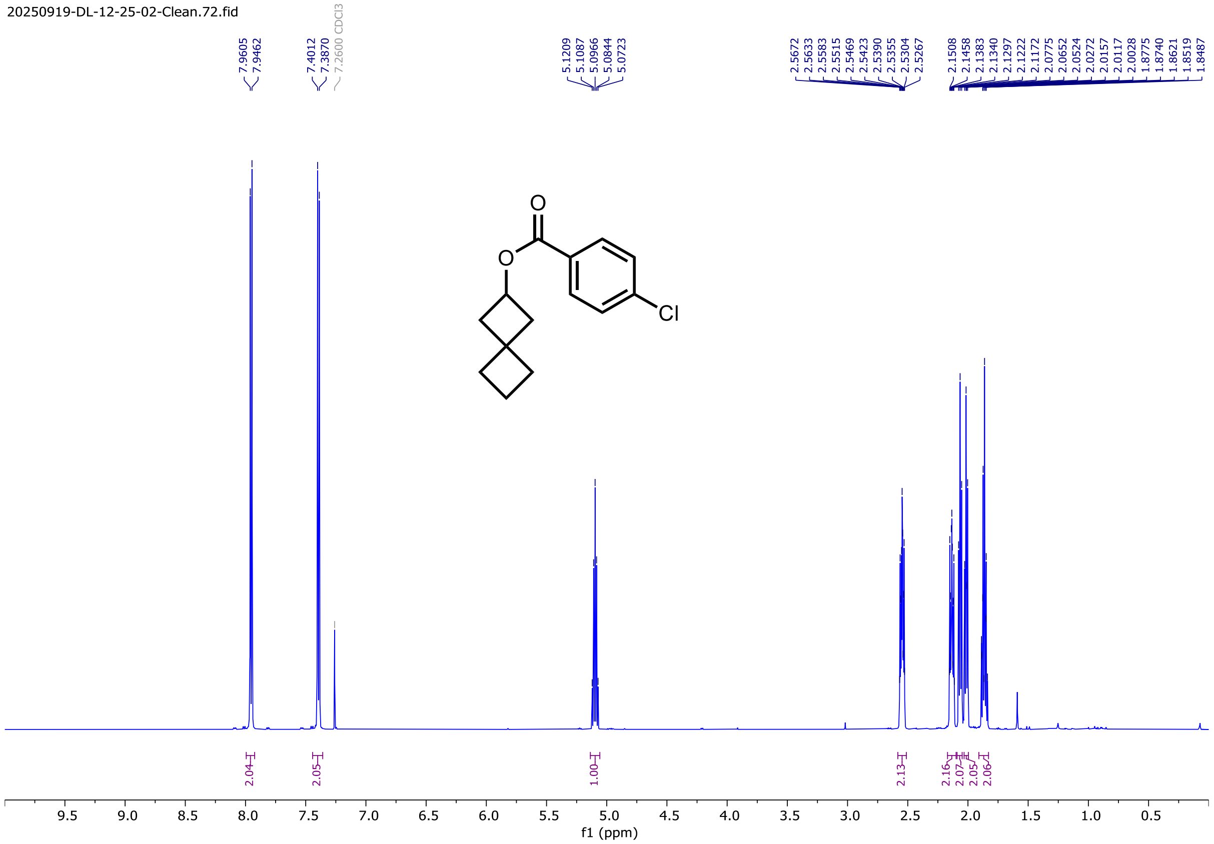
20250919-DL-12-25-02-Clean.72.fid
7.9605
7.9462
7.4012
7.3870
7.2600 CDCl3
5.1209
5.1087
5.0966
5.0844
5.0723
2.5672
2.5633
2.5583
2.5515
2.5469
2.5423
2.5390
2.5355
2.5304
2.5267
2.1508
2.1458
2.1383
2.1340
2.1297
2.1222
2.1172
2.0775
2.0652
2.0524
2.0272
2.0157
2.0117
2.0028
1.8775
1.8740
1.8621
1.8519
1.8487
O
O
Cl
2.04
2.05
1.00
2.13
2.16
2.07
2.05
2.06
9.5 9.0 8.5 8.0 7.5 7.0 6.5 6.0 5.5 5.0 4.5 4.0 3.5 3.0 2.5 2.0 1.5 1.0 0.5
f1 (ppm)

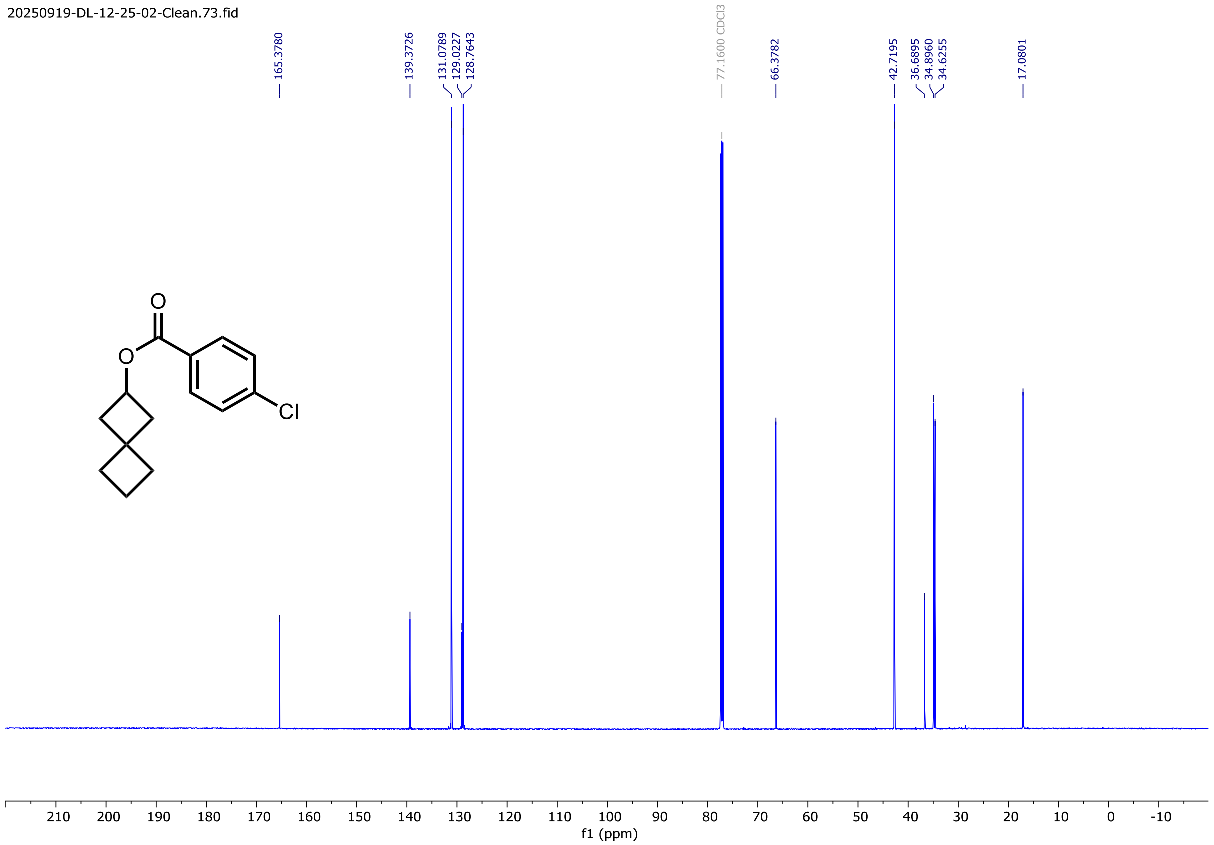
20250919-DL-12-25-02-Clean.73.fid
165.3780
139.3726
131.0789
129.0227
128.7643
77.1600 CDCl3
66.3782
42.7195
36.6895
34.8960
34.6255
17.0801
O
O
Cl
210 200 190 180 170 160 150 140 130 120 110 100 90 80 70 60 50 40 30 20 10 0 -10
f1 (ppm)

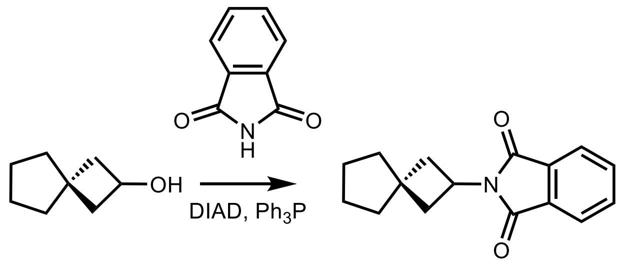

2-(spiro[3.4]octan-2-yl)isoindoline-1,3-dione (compound 50)

To a mixture of spiro[3.4]octan-2-ol[3] (2.03 g, 16.1 mmol, 1.0 equiv), phthalimide (2.37 g, 16.1 mmol, 1 equiv) and triphenylphosphine (8.45 g, 32.2 mmol, 2 equiv) were dissolved in dry tetrahydrofuran (100 mL). DIAD (6.35 ml, 6.51 g, 32.2 mmol, 2 equiv) was added dropwise under ice-water bath. After addition, the reaction was warmed up to 25°C and reacted for 4h, ethyl acetate was added, washed with saturated brine, the organic phase was dried over anhydrous sodium sulfate, concentrated in vacuum. The crude product was purified by flash chromatography (SiO_2, 0-15% diethyl ether in hexane) to obtain **compound 50** (900 mg, 22% yield) as a white solid.

R_f (1Hex/1Et2O) = 0.40 (UV 254 nm)

^{1}H NMR (600 MHz, $CDCl_3$) δ 7.80 (dd, *J* = 5.5, 3.0 Hz, 2H), 7.69 (dd, *J* = 5.5, 3.0 Hz, 2H), 4.71 (p, *J* = 9.0 Hz, 1H), 2.83 (td, *J* = 9.3, 2.8 Hz, 2H), 2.16 (td, *J* = 8.7, 2.8 Hz, 2H), 1.73 (t, *J* = 7.3 Hz, 2H), 1.70 (t, *J* = 7.1 Hz, 2H), 1.65 – 1.52 (m, 4H).

^{13}C NMR (151 MHz, $CDCl_3$) δ 168.8, 134.0, 132.2, 123.1, 40.7, 40.5, 40.3, 38.7, 38.7, 24.5, 23.9.

HRMS (+p APCI) calcd. for $[C_{16}H_{18}O_2N]$ ($[M+H]^+$) 256.1332 found 256.1335.

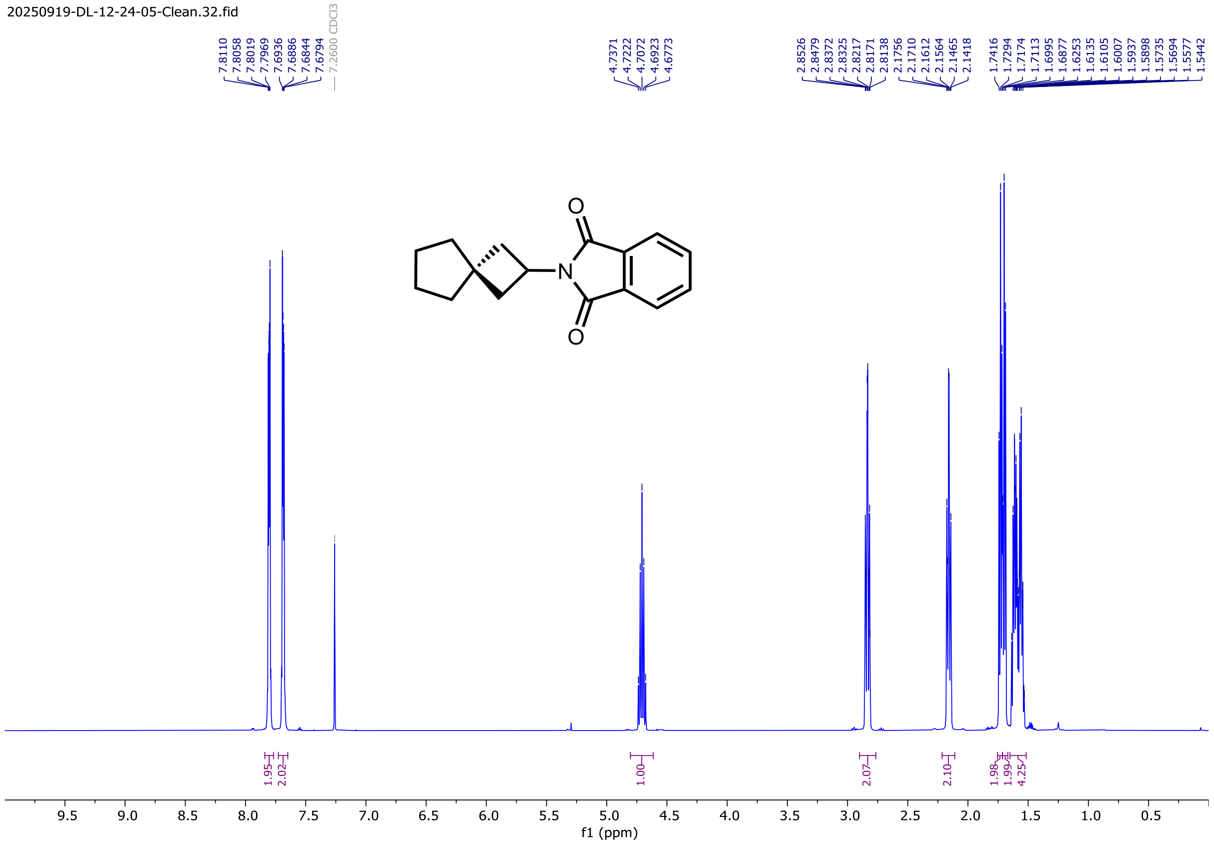
20250919-DL-12-24-05-Clean.32.fid
7.8110
7.8058
7.8019
7.7969
7.6936
7.6886
7.6844
7.6794
7.2600 CDCl3
4.7371
4.7222
4.7072
4.6923
4.6773
2.8526
2.8479
2.8372
2.8325
2.8217
2.8171
2.8138
2.1756
2.1710
2.1612
2.1564
2.1465
2.1418
1.7416
1.7294
1.7174
1.7113
1.6995
1.6877
1.6253
1.6135
1.6105
1.6007
1.5937
1.5898
1.5735
1.5694
1.5577
1.5442
O
N
O
1.95
2.02
1.00
2.07
2.10
1.98
1.99
4.25
9.5
9.0
8.5
8.0
7.5
7.0
6.5
6.0
5.5
5.0
4.5
4.0
3.5
3.0
2.5
2.0
1.5
1.0
0.5
f1 (ppm)

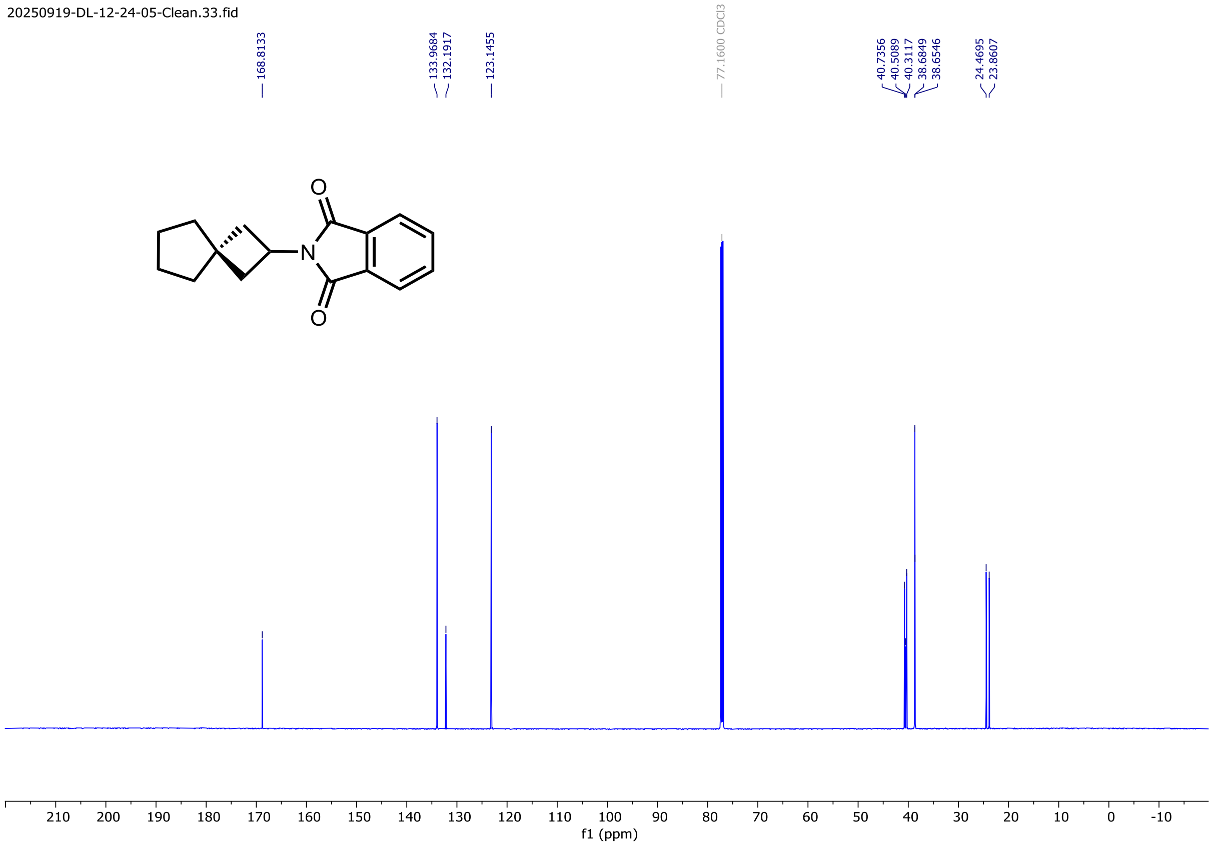
20250919-DL-12-24-05-Clean.33.fid
168.8133
133.9684
132.1917
123.1455
77.1600 CDCl3
40.7356
40.5089
40.3117
38.6849
38.6546
24.4695
23.8607
O
N
O
210
200
190
180
170
160
150
140
130
120
110
100
90
80
70
60
50
40
30
20
10
0
-10
f1 (ppm)

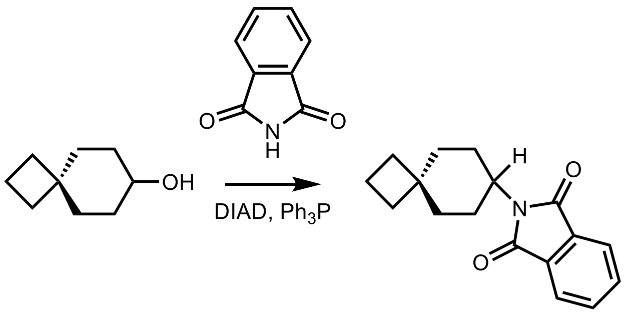

2-(spiro[3.5]nonan-7-yl)isoindoline-1,3-dione (Compound S12)

To a mixture of spiro[3.5]nonan-7-ol[3] (654.0 mg, 4.7 mmol, 1.0 equiv), phthalimide (686.2 g, 4.7 mmol, 1 equiv) and triphenylphosphine (1.84 g, 7.0 mmol, 1.5 equiv) were dissolved in dry tetrahydrofuran (50 mL). DIAD (1.36 ml, 1.42 g, 7.0 mmol, 1.5 equiv) was added dropwise under ice-water bath. After addition, the reaction was warmed up to 25°C and reacted for 4h, ethyl acetate was added, washed with saturated brine, the organic phase was dried over anhydrous sodium sulfate, concentrated in vacuum. The crude product was purified by flash chromatography (SiO_2, 0-15% diethyl ether in hexane) to obtain **compound S12** (450 mg, 36% yield) as a white solid.

R_f (1Hex/1Et2O) = 0.40 (UV 254 nm)

^{1}H NMR (400 MHz, $CDCl_3$) δ 7.80 (dd, J = 5.5, 3.1 Hz, 2H), 7.69 (dd, J = 5.5, 3.1 Hz, 2H), 4.05 (tt, J = 12.4, 3.9 Hz, 1H), 2.26 (qd, J = 12.9, 3.5 Hz, 2H), 1.89 – 1.83 (m, 5H), 1.79 – 1.67 (m, 2H), 1.58 – 1.50 (m, 2H), 1.37 (td, J = 13.4, 3.6 Hz, 2H).

^{13}C NMR (101 MHz, $CDCl_3$) δ 168.6, 133.9, 132.2, 123.1, 50.7, 38.7, 37.5, 33.3, 30.7, 29.9, 26.1, 15.3.

HRMS (+p APCI) calcd. for [$C_{17}H_{20}O_2N$] ($[M+H]^+$) 270.1489 found 270.1490.

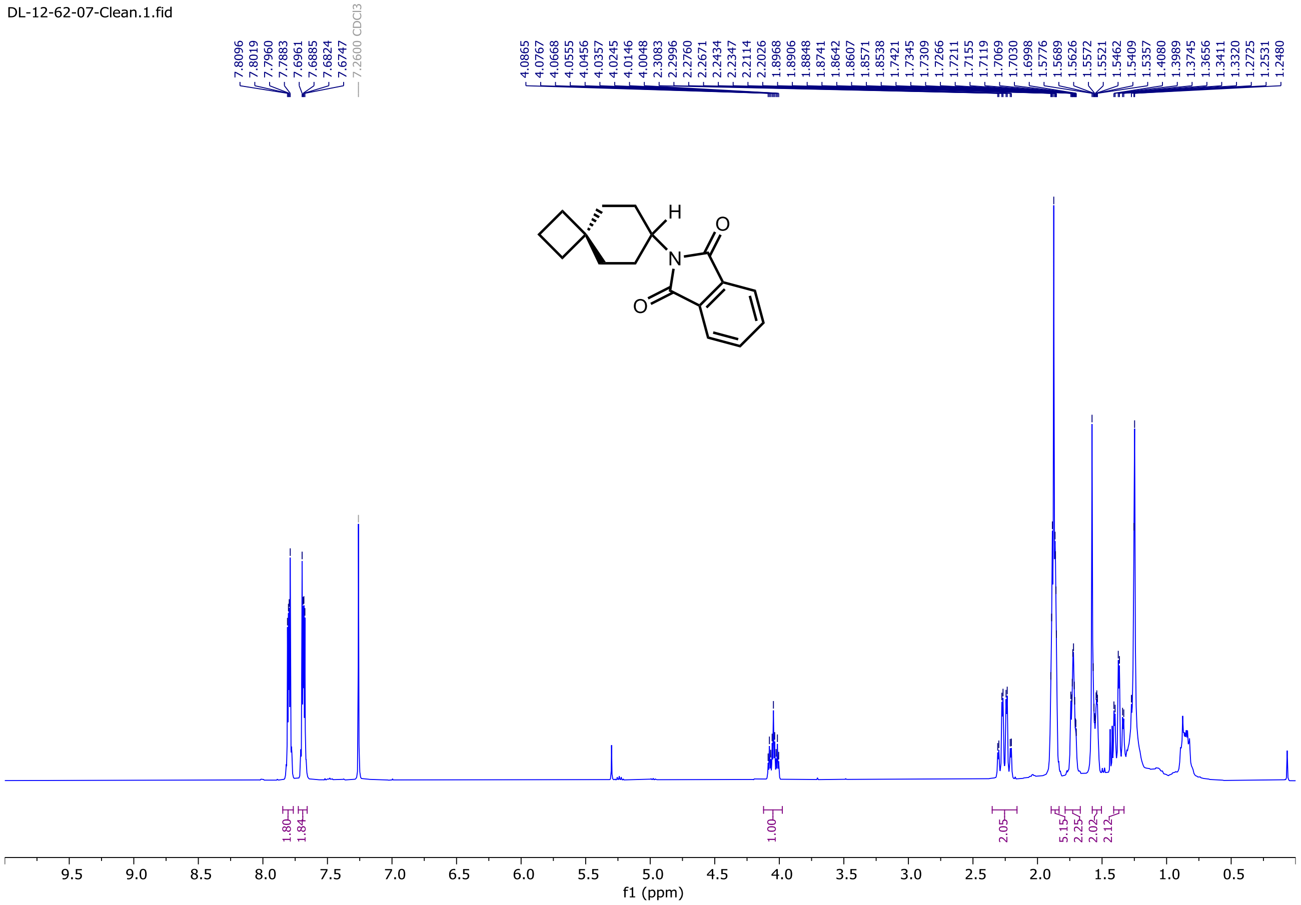
DL-12-62-07-Clean.1.fid
7.8096
7.8019
7.7960
7.7883
7.6961
7.6885
7.6824
7.6747
7.2600 CDCl3
4.0865
4.0767
4.0668
4.0555
4.0456
4.0357
4.0245
4.0146
4.0048
2.3083
2.2996
2.2760
2.2671
2.2434
2.2347
2.2114
2.2026
1.8968
1.8906
1.8848
1.8741
1.8642
1.8607
1.8571
1.8538
1.7421
1.7345
1.7309
1.7266
1.7211
1.7155
1.7119
1.7069
1.7030
1.6998
1.5776
1.5689
1.5626
1.5572
1.5521
1.5462
1.5409
1.5357
1.4080
1.3989
1.3745
1.3656
1.3411
1.3320
1.2725
1.2531
1.2480
H
N
O
O
1.80
1.84
1.00
2.05
5.15
2.25
2.02
2.12
9.5
9.0
8.5
8.0
7.5
7.0
6.5
6.0
5.5
5.0
4.5
4.0
3.5
3.0
2.5
2.0
1.5
1.0
0.5
f1 (ppm)

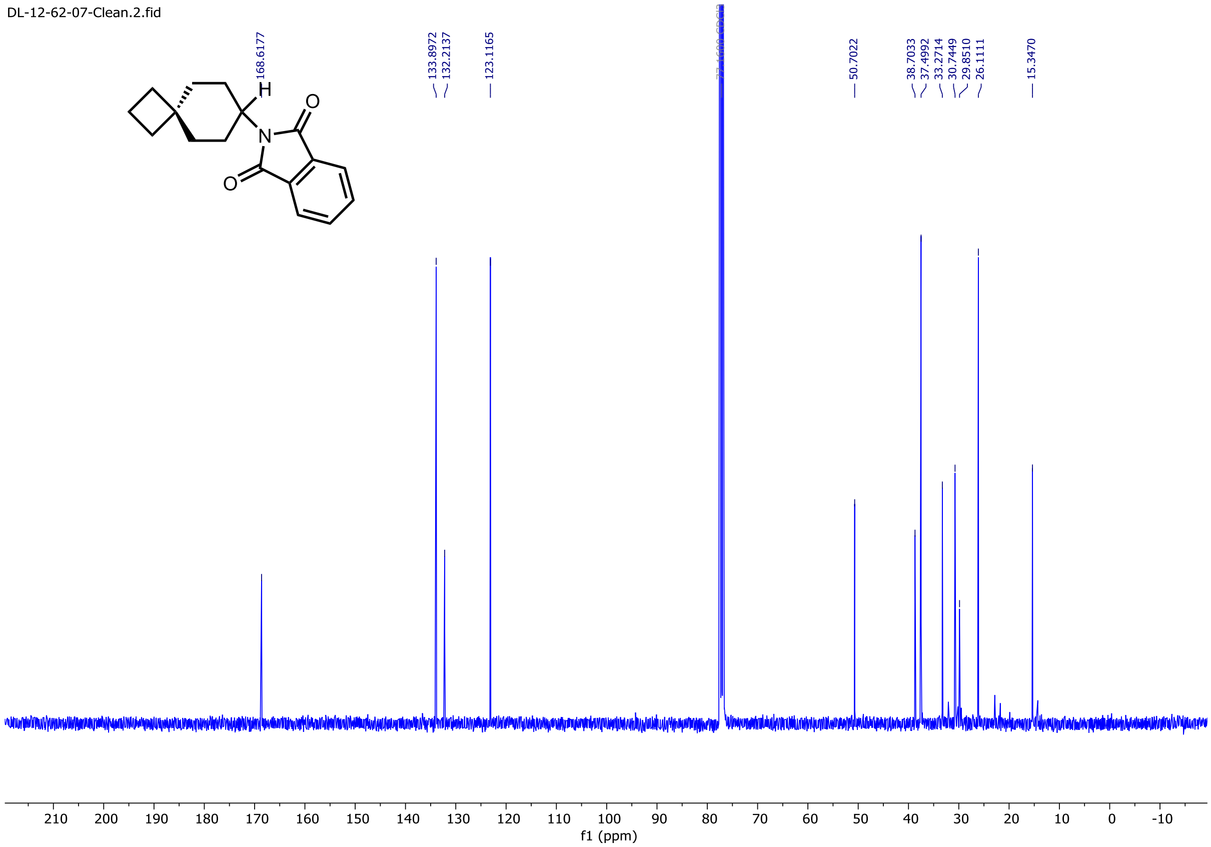
DL-12-62-07-Clean.2.fid
168.6177
133.8972
132.2137
123.1165
50.7022
38.7033
37.4992
33.2714
30.7449
29.8510
26.1111
15.3470
H
N
O
O
210
200
190
180
170
160
150
140
130
120
110
100
90
80
70
60
50
40
30
20
10
0
-10
f1 (ppm)

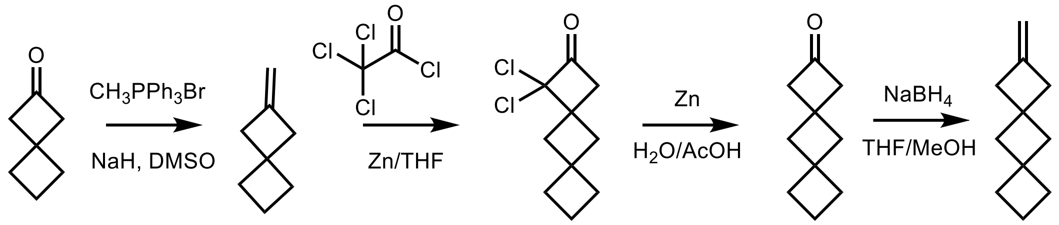

Step 1. To a flamed dried 250 ml round bottom flask, NaH (60 % dispersion in mineral oil, 1.82 g, 45.5 mmol, 1.5 equiv) was added and dissolved carefully in anhydrous DMSO (65 mL) at room temperature and heated up to 60 °C for 20 min. The resulting solution was cooled to room temperature and Ph_3PMeI (18.6 g, 52.0 mmol, 1.6 equiv) was added under argon atmosphere, and the mixture was allowed to react for 60 min. spiro[3.3]heptan-2-one (3.58 g, 32.5 mmol) was added dropwise at rt, and the resulting solution was stirred for 3 h at rt. The alkene formed was removed from the reaction mixture by Kugerol distillation under reduced pressure and condensed in a liquid nitrogen cooled vacuum trap. 2-methylenespiro[3.3]heptane retrieved from the vacuum trap was obtained as clear oil (2.50 g, 71% yield) and was used for subsequent steps without further purification.

Step 2. Zinc powder (2.12 g, 32.4 mmol, 1.4 equiv) was added to the solution of 2-methylenespiro[3.3]heptane (2.50 g, 23.1 mmol, 1.0 equiv) in THF (30.0 ml). The mixture was cooled to 0 °C in an ice bath, before trichloroacetic acid chloride (3.36 ml, 5.46 g, 30.0 mmol, 1.3 equiv) was added dropwise to the reaction mixture. The reaction was kept stirred in an ice bath for 1 hour before warming up to room temperature. After stirring overnight, the crude mixture was filtered through celite and washed by 20 ml of diethyl ether. The solvent was removed under vacuum to provide a dark yellowish oil crude.

Step 3. To the above crude mixture 47 ml of acetic acid and 69 ml of water were added. The mixture was cooled in an ice-bath followed by an addition of zinc powder (4.23 g, 64.7 mmol, 2.8 equiv). The mixture was let stirred overnight. Upon completion, the mixture was extract with pentane (200ml×2). The pentane layer was then washed with water then $NaHCO_3$(aq, sat), and brine. The pentane layer was then dried over Na_2SO_4. The solvent was removed under vacuum to provide dispiro[3.1.36.14]decan-2-one (1.77 g, 51% yield over 2 steps) as a slight yellowish clear oil. The product was used for the subsequent steps without further purification.

Step 4. dispiro[3.1.36.14]decan-2-one (901 mg, 6.0 mmol, 1.0 equiv) was added in methanol (17 mL), cooled with ice-bath, sodium borohydride (250 mg, 6.6 mmol, 1.1 equiv) was slowly added in batches. The mixture was stirred at 0 °C for 1 hour then at room temperature for another hour, then heated to 25°C and reacted for 2h. After completion of the reaction, the reaction was quenched by adding water and extract with ethyl acetate. The organic layer was then washed sequentially with water (30 mL)

and saturated brine (30 mL) followed by drying over anhydrous sodium sulfate. The solvent was removed under vacuum to provide dispiro[3.1.36.14]decan-2-ol as a colorless oil product, the product was used for the next step without further purification.

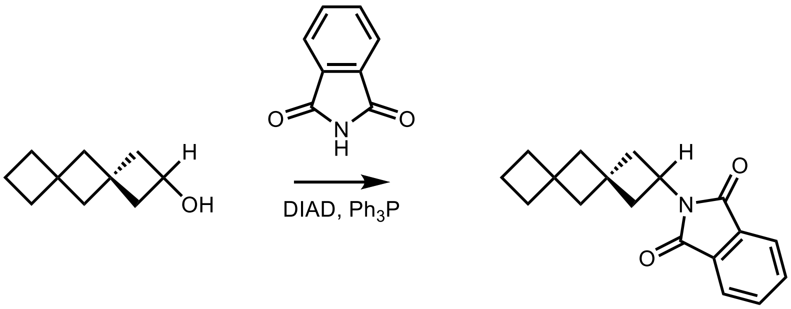

2-(dispiro[3.1.36.14]decan-2-yl)isoindoline-1,3-dione (Compound S13)

To a mixture of dispiro[3.1.36.14]decan-2-ol (913 mg, 6.0 mmol, 1.0 equiv) (from the above crude mixture), phthalimide (883 mg, 6.0 mmol, 1 equiv) and triphenylphosphine (3.15 g, 12.0 mmol, 2 equiv) were dissolved in dry tetrahydrofuran (50 mL). DIAD (2.37 ml, 2.43 g, 12 mmol, 2 equiv) was added dropwise under ice-water bath. After addition, the reaction was warmed up to 25°C and reacted for 4h, ethyl acetate was added, washed with saturated brine, the organic phase was dried over anhydrous sodium sulfate, concentrated in vacuum. The crude product was purified by flash chromatography (SiO_2, 0-15% diethyl ether in hexane) to obtain **compound S13** (296 mg, 18% yield) as a white solid.

R_f (1Hex/1Et2O) = 0.40 (UV 254 nm)

^{1}H NMR (400 MHz, $CDCl_3$) δ 7.80 (dd, J = 5.5, 3.0 Hz, 2H), 7.68 (dd, J = 5.5, 3.0 Hz, 2H), 4.60 (tt, J = 9.4, 8.2 Hz, 1H), 2.87 (td, J = 9.4, 2.9 Hz, 2H), 2.26 (td, J = 8.2, 2.9 Hz, 2H), 2.11 (d, J = 1.5 Hz, 2H), 2.08 (d, J = 1.5 Hz, 2H), 2.00 – 1.88 (m, 4H), 1.85 – 1.75 (m, 2H).

^{13}C NMR (101 MHz, $CDCl_3$) δ 168.8, 134.0, 132.1, 123.2, 47.4, 46.9, 40.9, 40.2, 39.6, 35.4, 32.8, 16.8.

HRMS (+p APCI) calcd. for [$C_{18}H_{20}O_2N$] ([M+H]$^+$) 282.1489 found 282.1492.

DL-12-29-05-Clean.1.fid

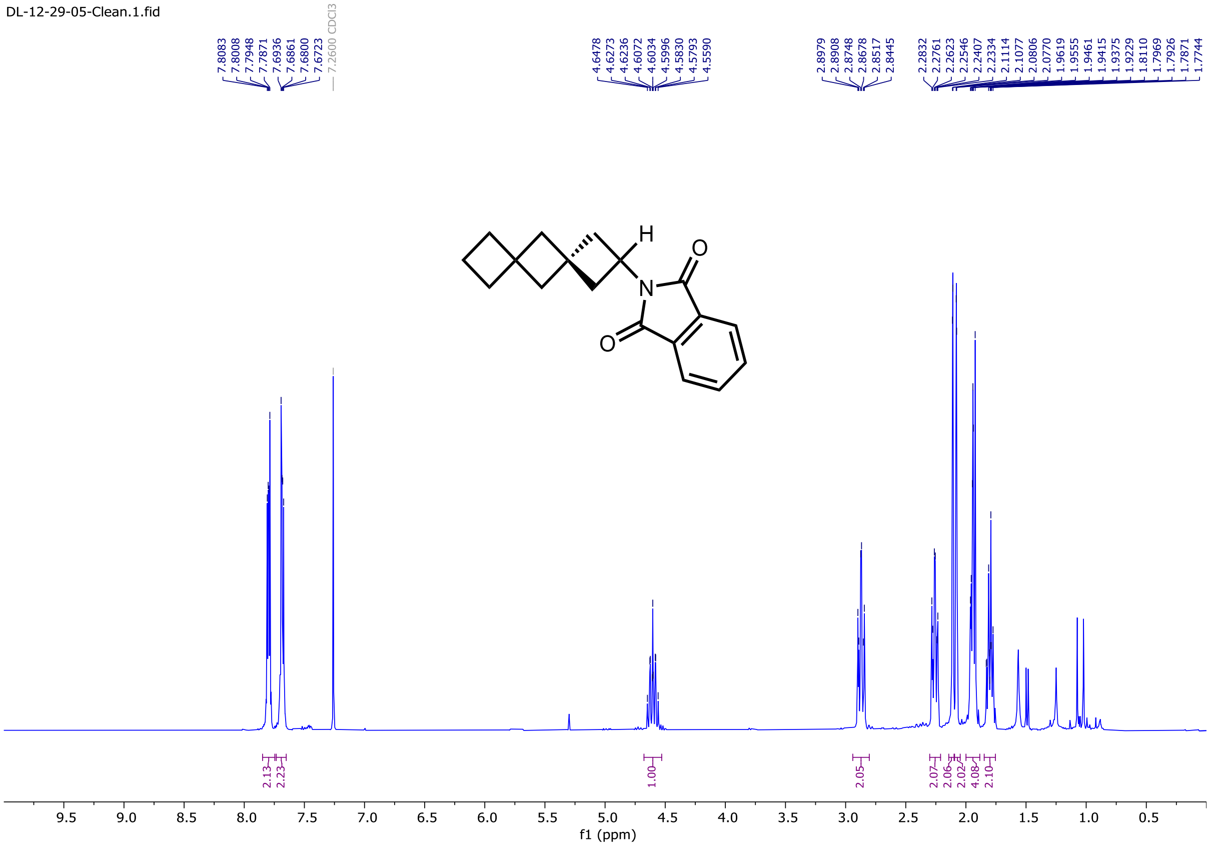

DL-12-29-05-Clean.2.fid

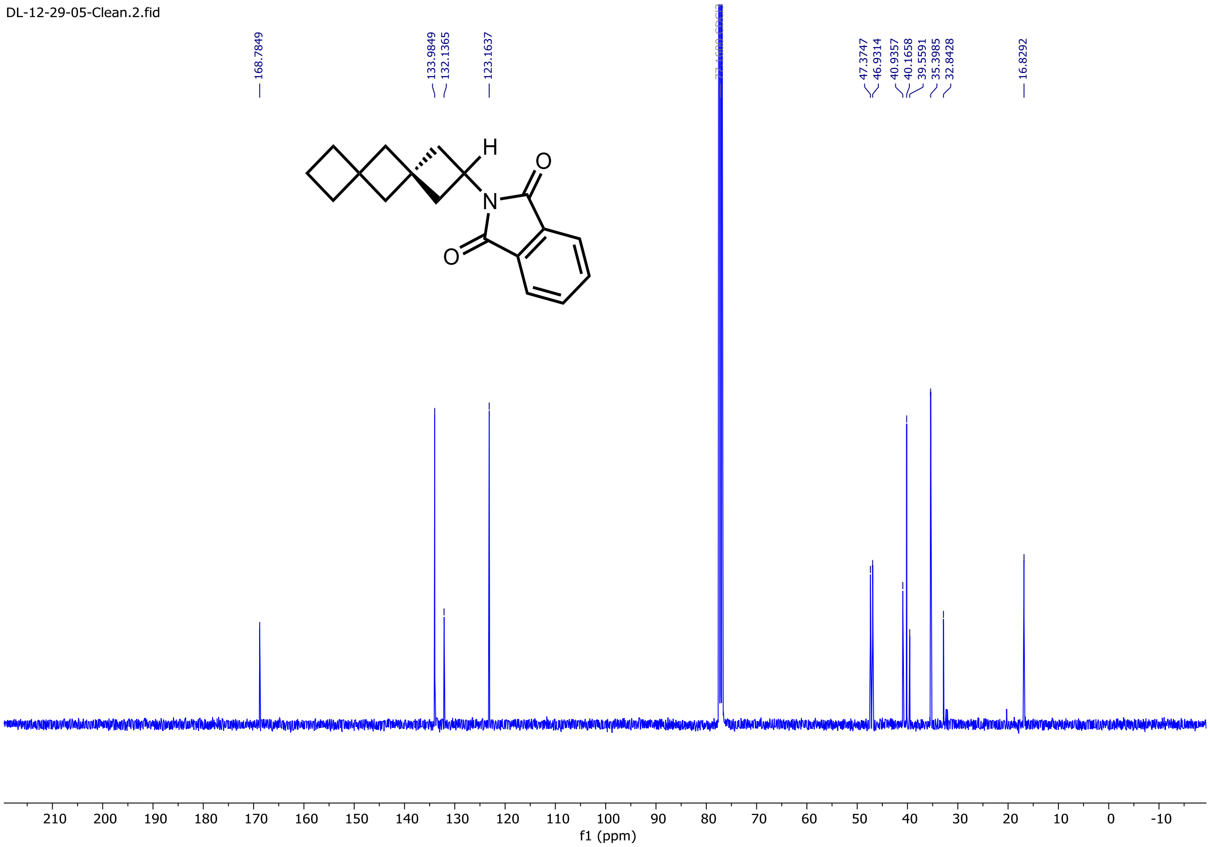

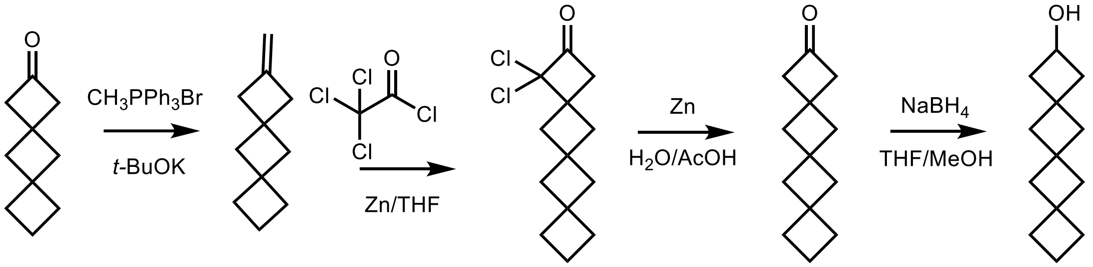

Step 1. To oven dried 250 ml round bottom flask was added methyltriphenylphosphonium bromide (6.31 g, 17.7 mmol, 1.5 equiv) and dry Et_2O (40 ml). The suspension was cool down to 0 ºC by an ice-bath followed by an addition of potassium tert-butoxide (1.98 g, 17.7 mmol, 1.5 eq). The mixture was kept stirring at 0 ºC for 2 hours before a solution of dispiro[3.1.36.14]decan-2-one (1.77 g, 11.8 mmol, 1.0 equiv) in Et_2O (10 ml) was added. The reaction mixture warmed up to room temperature and kept stirring overnight. The reaction is then quenched by the addition of H_2O (50 ml). The reaction mixture is then extracted by pentane (3 × 30 ml). The combined organic layer is dried over Na_2SO_4 and solvent was then removed under vacuum. The crude was then dissolved in pentane, and filtered through a silica plug, using pentane to wash. The combined organic is concentrated under vacuum to provide 2-methylenedispiro[3.1.36.14]decane as clear oil.

Step 2. Zinc powder (1.08 g, 16.5 mmol, 1.4 equiv) was added to the solution 2-methylenedispiro[3.1.36.14]decane (1.75 g, 17.7 mmol, 1.0 equiv) in THF (30.0 ml). The mixture was cooled to 0 ºC in an ice bath, before trichloroacetic acid chloride (1.72 ml, 2.79 g, 15.3 mmol, 1.3 equiv) was added dropwise to the reaction mixture. The reaction was kept stirred in an ice bath for 1 hour before warming up to room temperature. After stirring overnight, the crude mixture was filtered through celite and washed by 20 ml of diethyl ether. The solvent was removed under vacuum to provide a dark yellowish oil crude.

Step 3. To the above crude mixture 24 ml of acetic acid and 35 ml of water were added. The mixture was cooled in an ice-bath followed by an addition of zinc powder (2.16 g, 33.0 mmol, 2.8 equiv). The mixture was let stirred overnight. Upon completion, the mixture was extract with pentane (200ml×2). The pentane layer was then washed with water then $NaHCO_3$(aq, sat), and brine. The pentane layer was then dried over Na_2SO_4. The solvent was removed under vacuum to provide dispiro[3.1.36.14]decan-2-one (1.20 g, 53% yield over 2 steps) as a slight yellowish clear oil. The product was used for the subsequent steps without further purification.

Step 4. dispiro[3.1.36.14]decan-2-one (1.00 g, 5.3 mmol, 1.0 equiv) was added in methanol (20 mL), cooled with ice-bath, sodium borohydride (219 mg, 5.8 mmol, 1.1 equiv) was slowly added in batches. The mixture was stirred at 0 ºC for 1 hour then at room temperature for another hour, then heated to 25°C and reacted for 2h. After completion of the reaction, the reaction was quenched by adding water

and extract with ethyl acetate. The organic layer was then washed sequentially with water (30 mL) and saturated brine (30 mL) followed by drying over anhydrous sodium sulfate. The solvent was removed under vacuum to provide trispiro[3.1.1.38.16.14]tridecan-2-ol as a colorless oil product, the product was used for the next step without further purification.

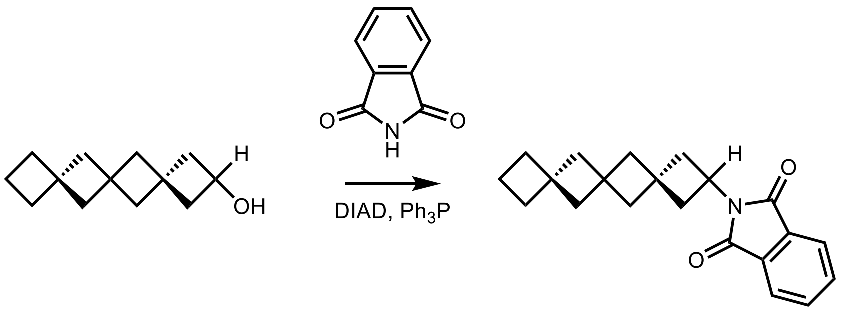

2-(trispiro[3.1.1.38.16.14]tridecan-2-yl)isoindoline-1,3-dione (Compound S14)

To a mixture of trispiro[3.1.1.38.16.14]tridecan-2-ol (1.01 g, 5.26 mmol, 1.0 equiv) (from the above crude product) and phthalimide (774 mg, 5.26 mmol, 1 equiv) and triphenylphosphine (2.76 g, 10.5 mmol, 2 equiv) were dissolved in dry tetrahydrofuran (100 mL). DIAD (2.08 ml, 2.13 g, 10.5 mmol, 2 equiv) was added dropwise under ice-water bath. After addition, the reaction was warmed up to 25°C and reacted for 4h, ethyl acetate was added, washed with saturated brine, the organic phase was dried over anhydrous sodium sulfate, concentrated in vacuum. The crude product was purified by flash chromatography (SiO_2, 0-15% diethyl ether in hexane) to obtain **compound S14** (807 mg, 48% yield) as a white solid.

R_f (1Hex/1Et2O) = 0.40 (UV 254 nm)

^{1}H NMR (600 MHz, $CDCl_3$) δ 7.79 (dd, J = 5.5, 3.0 Hz, 2H), 7.68 (dd, J = 5.5, 3.0 Hz, 2H), 4.59 (tt, J = 9.4, 8.3 Hz, 1H), 2.86 (td, J = 9.4, 2.8 Hz, 2H), 2.26 (td, J = 8.3, 2.8 Hz, 2H), 2.09 (s, 2H), 2.06 (s, 2H), 1.96 (s, 4H), 1.90 (t, J = 7.1 Hz, 4H), 1.80 – 1.74 (m, 2H).

^{13}C NMR (151 MHz, $CDCl_3$) δ 168.8, 134.0, 132.2, 123.2, 47.9, 47.7, 47.3, 41.0, 40.2, 39.5, 35.5, 35.5, 34.6, 33.2, 16.9.

HRMS (+p APCI) calcd. for [$C_{21}H_{24}O_2N$] ([M+H]$^+$) 322.1802 found 322.1806.

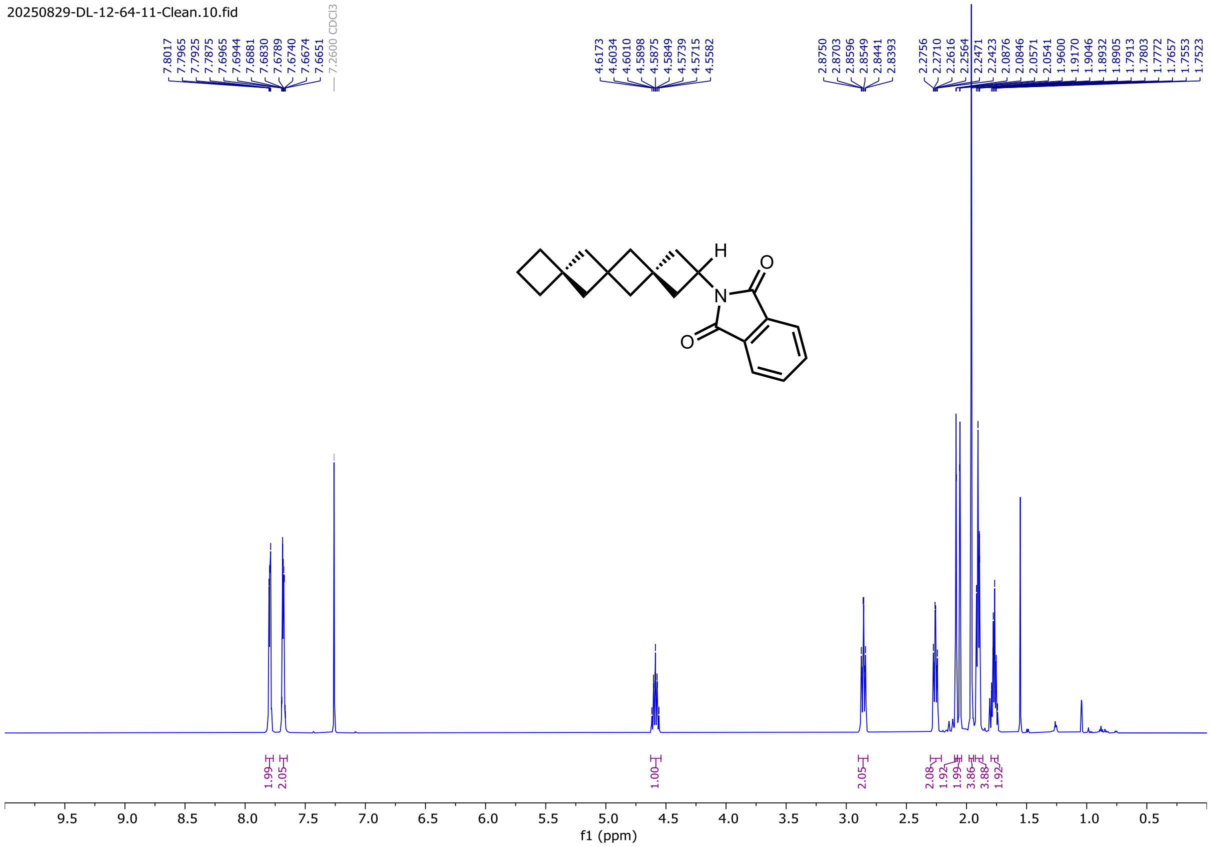

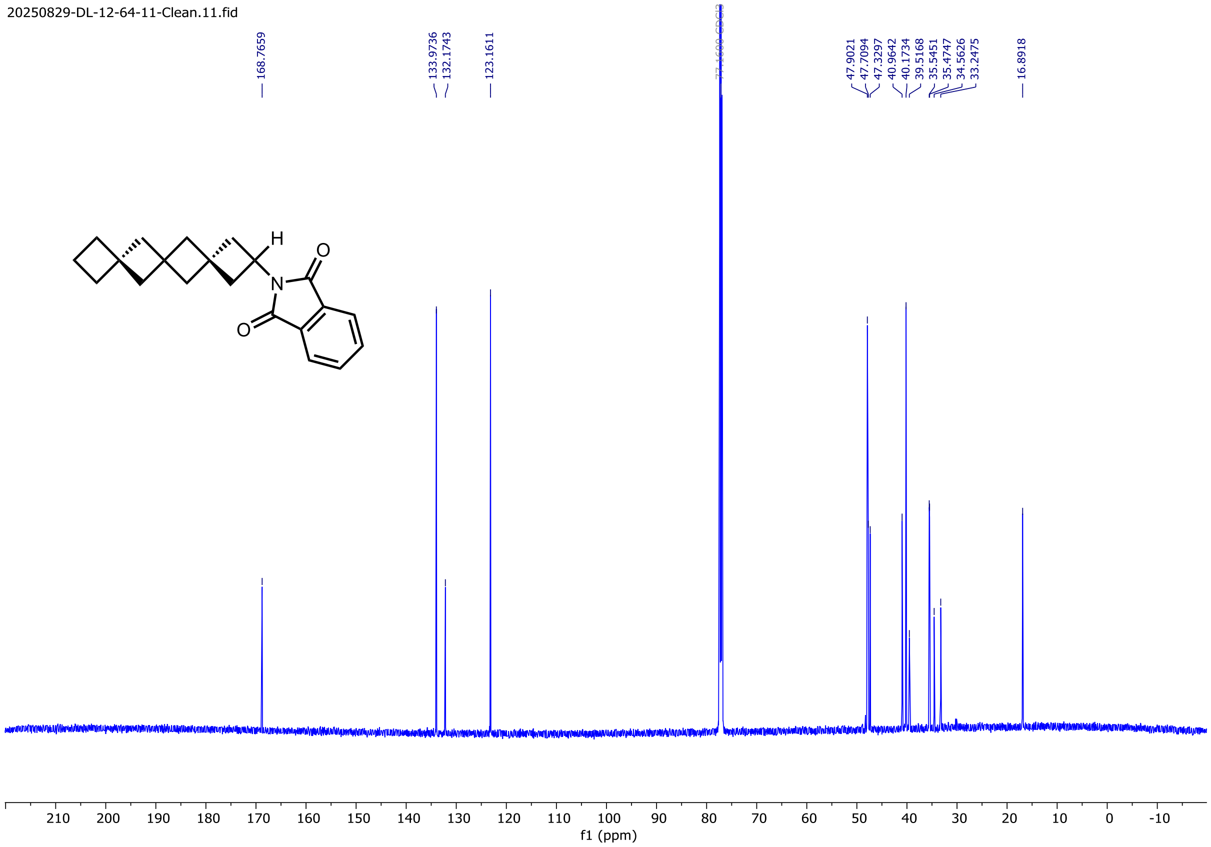

6. Product characterization

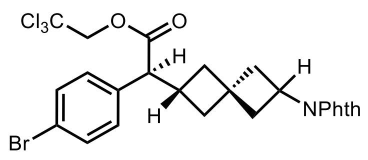

2,2,2-trichloroethyl (2S)-2-(4-bromophenyl)-2-(6-(1,3-dioxoisoindolin-2-yl)spiro[3.3]heptan-2-yl)acetate (Compound 22)

Prepared according to general procedure for C-H functionalization, 2-(spiro[3.3]heptan-2-yl)isoindoline-1,3-dione (48.3 mg, 0.2 mmol, 1.0 equiv), Rh_2(*S*-MegaBNP)$_4$ (3.4 mg, 0.0001 mmol, 0.005 equiv), molecular sieve 4Å (100 wt%) and 2,2,2- HFIP (5 µL, 8.40 mg, 0.05 mmol, 0.25 equiv) in 0.5 ml CH_2Cl_2 were added a solution of trichloroethyl 2-(4-bromophenyl)-2-diazoacetate (149.0 mg, 0.4 mmol, 2.0 equiv) in 2.0 ml CH_2Cl_2 at 39℃ in 3 hours. The crude mixture was purified by flash chromatography (SiO_2, gradient 0%-20% Et_2O in hexane) afforded **compound 22** as a white amorphous solid (107.4 mg, 92% yield, 99% ee, >20:1 dr). Note. **Compound 22** was obtained with (919 mg, 79% yield, 99% ee, >20:1 dr) at 2.0 mmol scale. The opposite enantiomer of **compound 22** was also obtained with (900 mg, 77% yield, 99% ee, >20:1 dr) at 2.0 mmol scale using Rh_2(*R*-MegaBNP)$_4$.

R_f (1Hex/2Et_2O) = 0.50 (CAM)

$[\alpha]^{20}_D$: 22.2° (c = 2.57 g/100 ml, $CHCl_3$, 99% ee)

^{1}H NMR (600 MHz, $CDCl_3$) δ 7.79 (dd, *J* = 5.4, 3.0 Hz, 2H), 7.68 (dd, *J* = 5.4, 3.0 Hz, 2H), 7.45 (d, *J* = 8.4 Hz, 2H), 7.19 (d, *J* = 8.4 Hz, 2H), 4.77 (d, *J* = 11.9 Hz, 1H), 4.67 (d, *J* = 11.9 Hz, 1H), 4.66 (p, *J* = 8.3 Hz, 1H), 3.59 (d, *J* = 11.0 Hz, 1H), 2.97 – 2.81 (m, 3H), 2.52 – 2.39 (m, 2H), 2.26 (ddd, *J* = 12.3, 8.2, 4.8 Hz, 1H), 2.06 – 1.95 (m, 2H), 1.73 (dd, *J* = 11.9, 8.7 Hz, 1H).

^{13}C NMR (151 MHz, $CDCl_3$) δ 171.2, 168.7, 135.6, 134.1, 132.1, 131.9, 130.1, 123.2, 121.8, 94.9, 74.2, 57.5, 40.5, 40.2, 40.2, 39.7, 38.6, 33.5, 33.2.

HRMS (+p APCI) calcd. for [$C_{25}H_{22}O_4N^{79}Br^{35}Cl_3$] ([M+H]$^+$) 583.9792 found 583.9806.

SFC (AMY1, 30% (50% methanol in isopropanol with 0.2% Formic Acid) in CO_2, 2.5 mL/min, 1.0 mg/ml, UV 230 nm) retention times of 4.27 min (major) and 5.02 min (minor), 99% ee. (For major diastereomer)

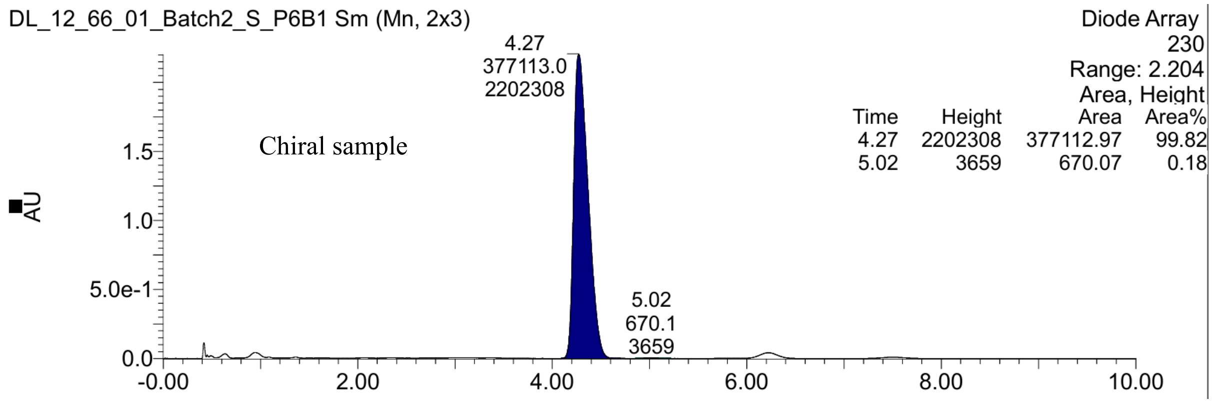
DL_12_66_01_Batch2_S_P6B1 Sm (Mn, 2x3)
Diode Array
230
Range: 2.204
Area, Height
Time Height Area Area%
4.27 2202308 377112.97 99.82
5.02 3659 670.07 0.18
Chiral sample
4.27
377113.0
2202308
5.02
670.1
3659
AU

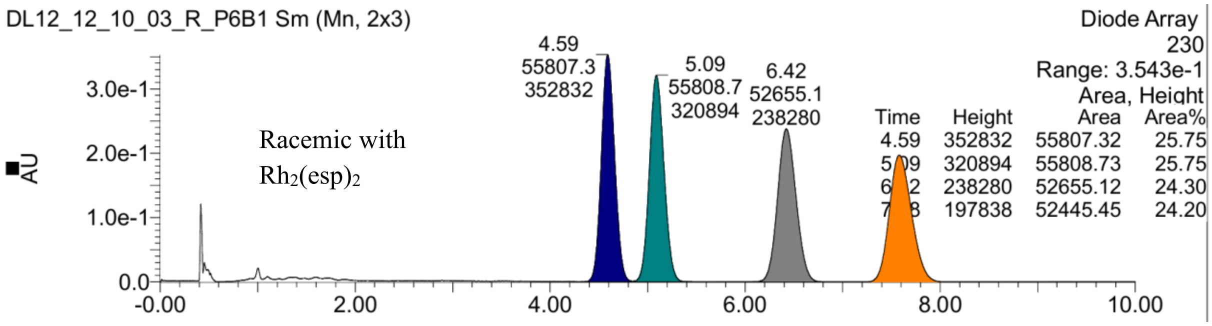
DL12_12_10_03_R_P6B1 Sm (Mn, 2x3)
Diode Array
230
Range: 3.543e-1
Area, Height
Time Height Area Area%
4.59 352832 55807.32 25.75
320894 55808.73 25.75
238280 52655.12 24.30
197838 52445.45 24.20
Racemic with
Rh2(esp)2
4.59
55807.3
352832
5.09
55808.7
320894
6.42
52655.1
238280
AU

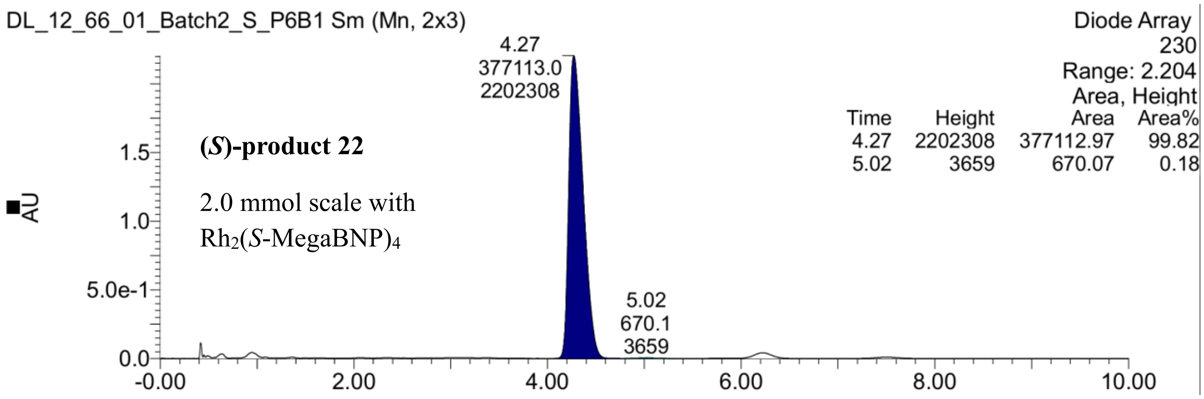
DL_12_66_01_Batch2_S_P6B1 Sm (Mn, 2x3)
Diode Array
230
Range: 2.204
Area, Height
Time Height Area Area%
4.27 2202308 377112.97 99.82
5.02 3659 670.07 0.18
(S)-product 22
2.0 mmol scale with
Rh2(S-MegaBNP)4
4.27
377113.0
2202308
5.02
670.1
3659
AU

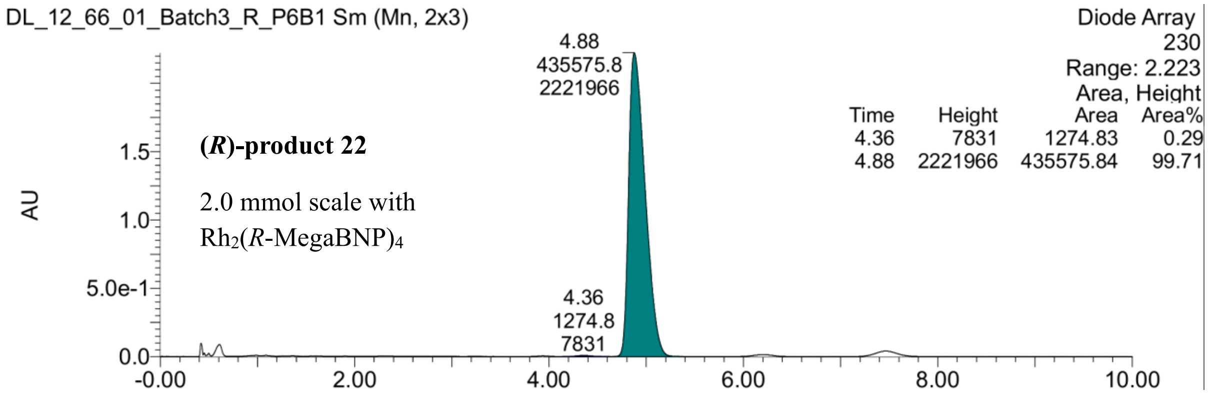
DL_12_66_01_Batch3_R_P6B1 Sm (Mn, 2x3)
Diode Array
230
Range: 2.223
Area, Height
Time Height Area Area%
4.36 7831 1274.83 0.29
4.88 2221966 435575.84 99.71
(R)-product 22
2.0 mmol scale with
Rh2(R-MegaBNP)4
4.88
435575.8
2221966
4.36
1274.8
7831
AU

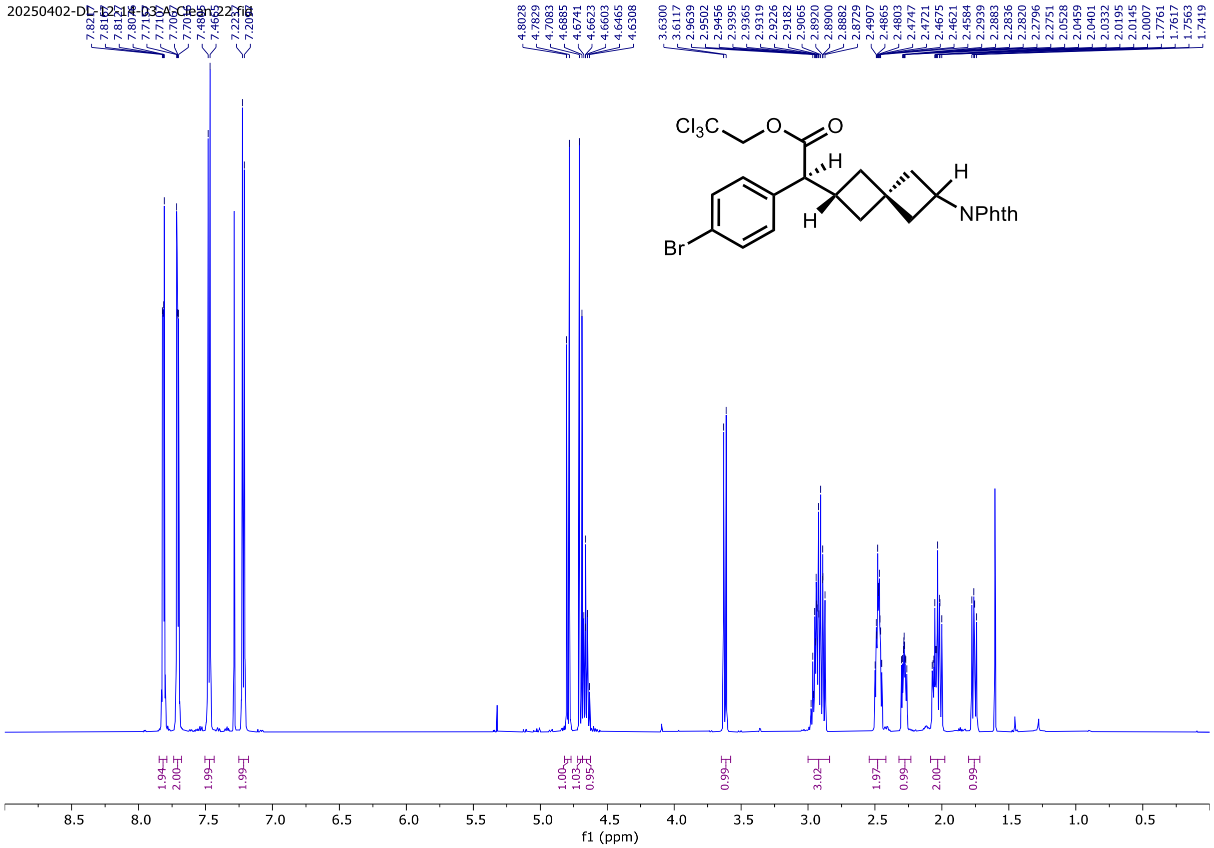

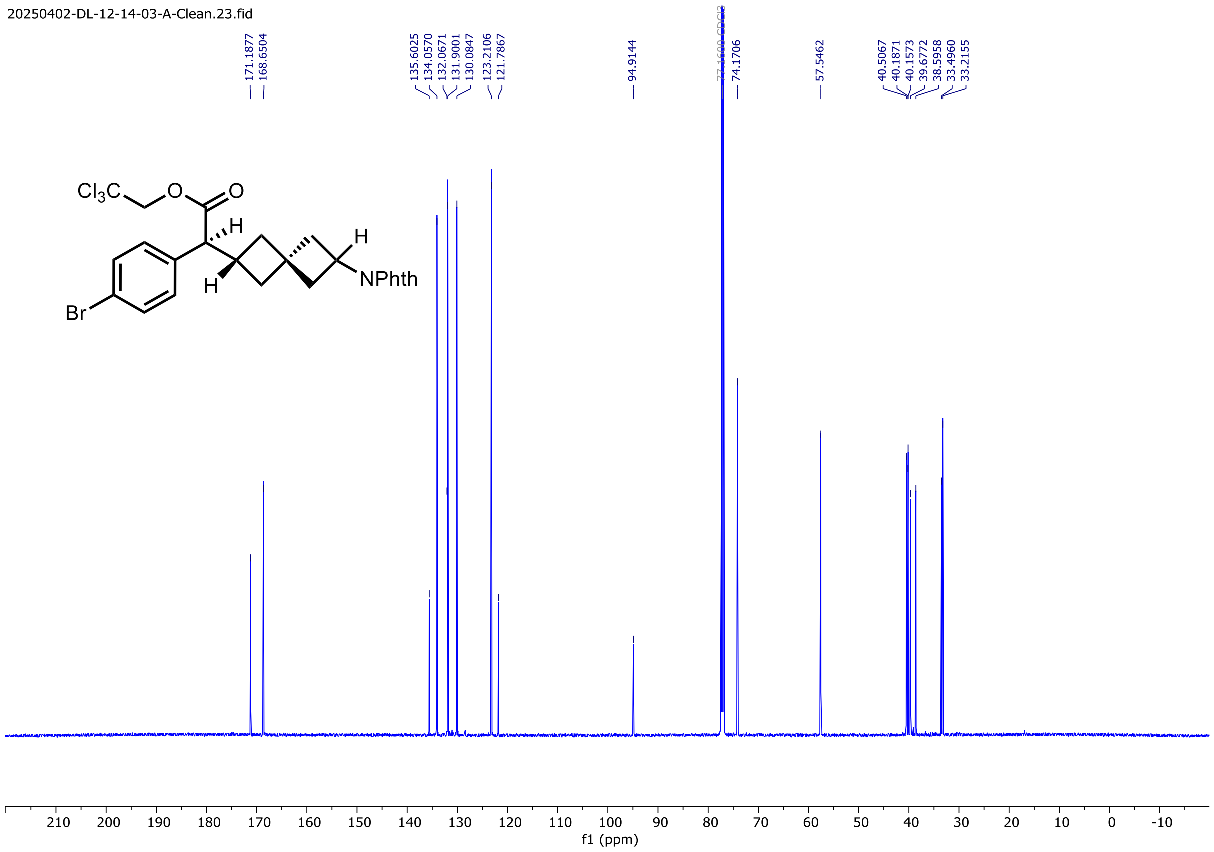

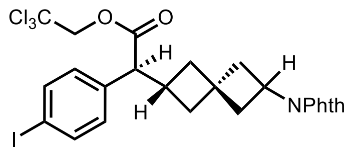

2,2,2-trichloroethyl (2S)-2-(6-(1,3-dioxoisoindolin-2-yl)spiro[3.3]heptan-2-yl)-2-(4-iodophenyl)acetate (Compound 23)

Prepared according to general procedure for C-H functionalization, 2-(spiro[3.3]heptan-2-yl)isoindoline-1,3-dione (48.3 mg, 0.2 mmol, 1.0 equiv), Rh_2(*S*-MegaBNP)$_4$ (3.4 mg, 0.0001 mmol, 0.005 equiv), molecular sieve 4Å (100 wt%) and 2,2,2- HFIP (5 μL, 8.40 mg, 0.05 mmol, 0.25 equiv) in 0.5 ml CH_2Cl_2 were added a solution of trichloroethyl 2-(4-iodophenyl)-2-diazoacetate (168.0 mg, 0.4 mmol, 2.0 equiv) in 2.0 ml CH_2Cl_2 at 39℃ in 3 hours. The crude mixture was purified by flash chromatography (SiO_2, gradient 0%-25% Et_2O in hexane) afforded **compound 23** as a white amorphous solid (117.5 mg, 93% yield, 99% ee, 29:1 dr).

R_f (1H/2Et_2O) = 0.50 (CAM, UV 254 nm)

$[\alpha]^{20}_D$: 23.9° (c = 1.5 g/100 ml, $CHCl_3$, 99% ee)

^{1}H NMR (600 MHz, $CDCl_3$) δ 7.79 (dd, *J* = 5.5, 3.0 Hz, 2H), 7.68 (dd, *J* = 5.5, 3.0 Hz, 2H), 7.65 (d, *J* = 8.3 Hz, 2H), 7.06 (d, *J* = 8.3 Hz, 2H), 4.77 (d, *J* = 11.9 Hz, 1H), 4.67 (d, *J* = 11.9 Hz, 1H), 4.66 – 4.60 (m, 1H), 3.58 (d, *J* = 11.0 Hz, 1H), 2.96 – 2.83 (m, 3H), 2.49 – 2.40 (m, 2H), 2.25 (ddd, *J* = 11.0, 8.2, 4.7 Hz, 1H), 2.07 – 1.96 (m, 2H), 1.73 (dd, *J* = 11.9, 8.7 Hz, 1H).

^{13}C NMR (151 MHz, $CDCl_3$) δ 171.2, 168.7, 137.9, 136.3, 134.1, 132.1, 130.3, 123.2, 94.9, 93.4, 74.2, 57.7, 40.5, 40.2, 40.2, 39.7, 38.6, 33.5, 33.2.

HRMS (+p APCI) calcd. for [$C_{25}H_{22}O_4N^{35}Cl_3{}^{127}I$] ($[M+H]^+$) 631.9654 found 631.9673.

HPLC (Chiralpak ADH column, 5% isopropanol in hexane, 1.0 $mLmin^{-1}$, 1.0 $mgmL^{-1}$, 60 min, UV 230 nm) retention times of 22.5 min (major) and 28.6 min (major), 99% ee.

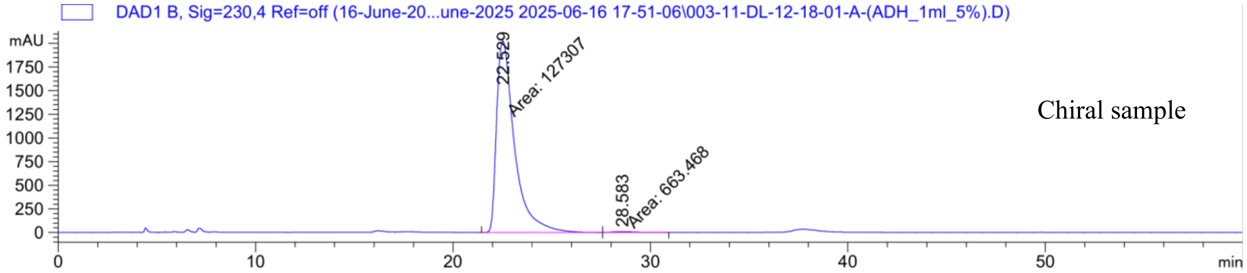


```
Signal 2: DAD1 B, Sig=230,4 Ref=off

Peak RetTime Type  Width     Area      Height     Area
  #   [min]        [min]   [mAU*s]     [mAU]       %
----|-------|----|-------|----------|----------|--------|
   1  22.529 MF     1.0471 1.27307e5  2026.37622  99.4815
   2  28.583 FM     1.3551  663.46814    8.16010   0.5185

Totals :                   1.27971e5  2034.53632
```

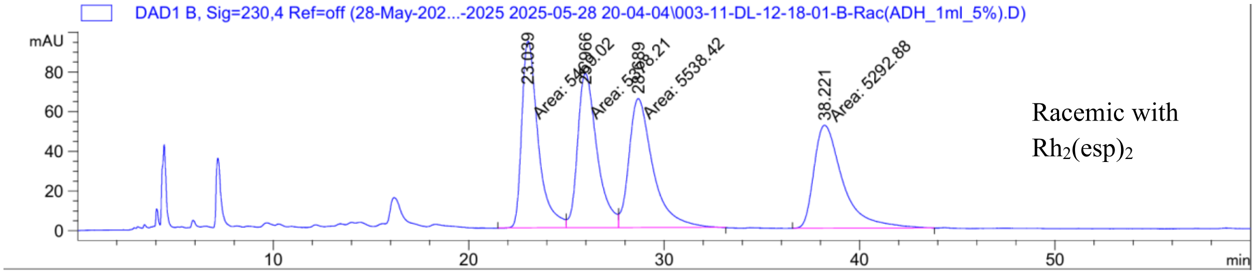


```
Signal 2: DAD1 B, Sig=230,4 Ref=off

Peak RetTime Type  Width     Area      Height     Area
  #   [min]        [min]   [mAU*s]     [mAU]       %
----|-------|----|-------|----------|----------|--------|
   1  23.039 MF     0.9706 5469.02441   93.91392  25.2278
   2  25.966 MF     1.1538 5378.20654   77.68995  24.8089
   3  28.689 FM     1.4210 5538.42334   64.96030  25.5480
   4  38.221 MM     1.7030 5292.87646   51.79987  24.4153

Totals :                   2.16785e4   288.36404
```

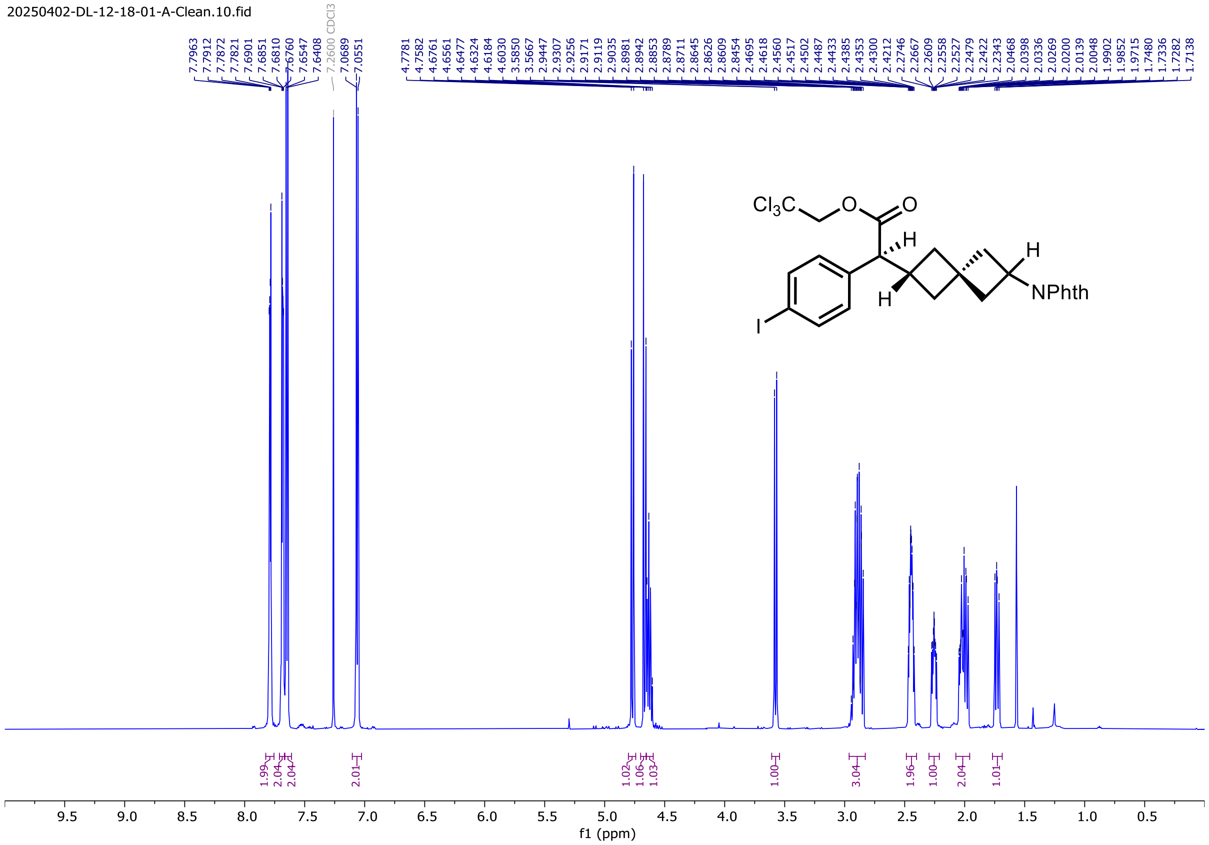
20250402-DL-12-18-01-A-Clean.10.fid
f1 (ppm)
NPhth

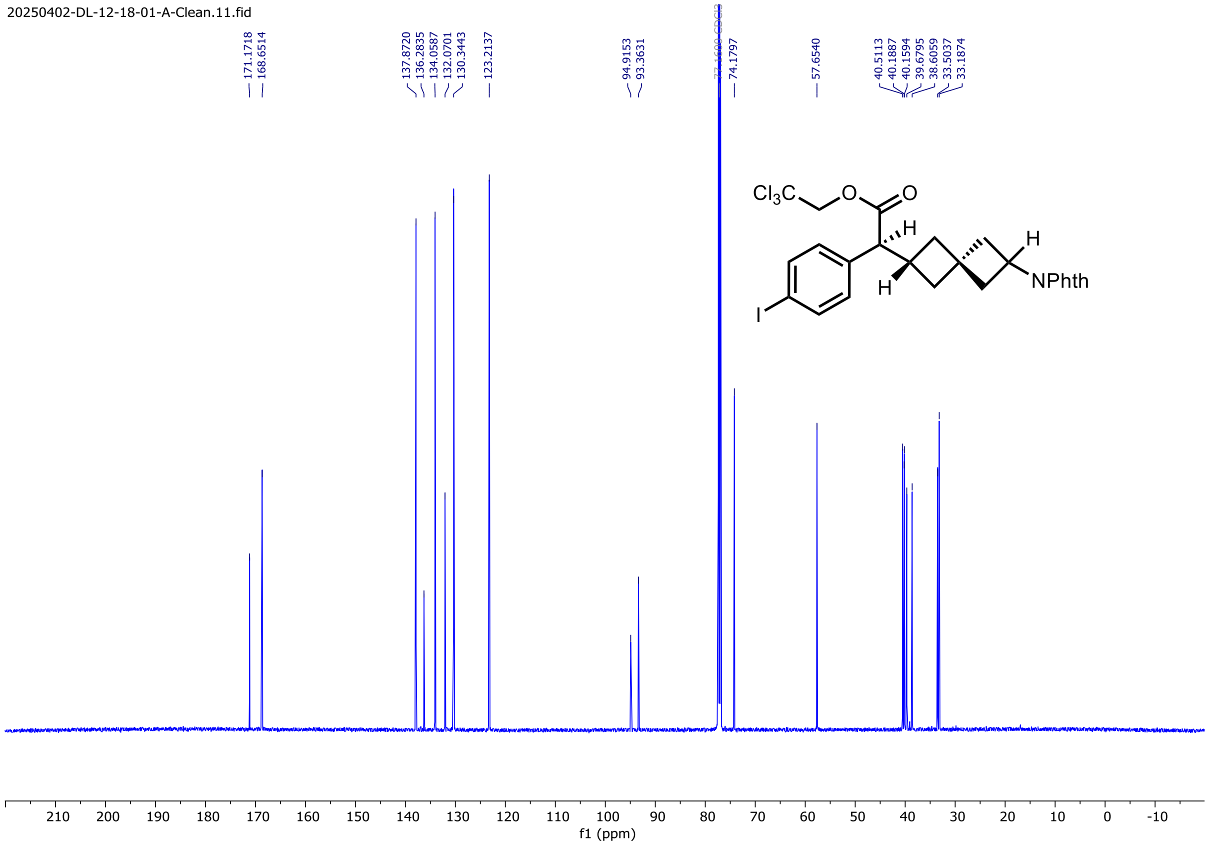
20250402-DL-12-18-01-A-Clean.11.fid
171.1718
168.6514
137.8720
136.2835
134.0587
132.0701
130.3443
123.2137
94.9153
93.3631
74.1797
57.6540
40.5113
40.1887
40.1594
39.6795
38.6059
33.5037
33.1874
f1 (ppm)
NPhth

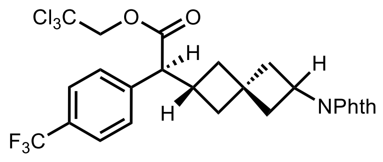

2,2,2-trichloroethyl (2S)-2-(6-(1,3-dioxoisoindolin-2-yl)spiro[3.3]heptan-2-yl)-2-(4-(trifluoromethyl)phenyl)acetate (Compound 24)

Prepared according to general procedure for C-H functionalization, 2-(spiro[3.3]heptan-2-yl)isoindoline-1,3-dione (48.3 mg, 0.2 mmol, 1.0 equiv), Rh_2(*S*-MegaBNP)$_4$ (3.4 mg, 0.0001 mmol, 0.005 equiv), molecular sieve 4Å (100 wt%) and 2,2,2- HFIP (5 µL, 8.40 mg, 0.05 mmol, 0.25 equiv) in 0.5 ml CH_2Cl_2 were added a solution of trichloroethyl 2-(4-(trifluoromethyl)phenyl)-2-diazoacetate (145.0 mg, 0.4 mmol, 2.0 equiv) in 2.0 ml CH_2Cl_2 at 39℃ in 3 hours. The crude mixture was purified by flash chromatography (SiO_2, gradient 0%-25% Et_2O in hexane) afforded **compound 24** as a clear oil (110.6 mg, 96% yield, 97% ee, 18:1 dr).

R_f (1Hex/2Et_2O) = 0.50 (CAM, UV 254 nm)

$[\alpha]^{20}_D$: 17.6° (c = 1.35 g/100 ml, $CHCl_3$, 97% ee)

^{1}H NMR (600 MHz, $CDCl_3$) δ 7.79 (dd, *J* = 5.5, 3.0 Hz, 2H), 7.68 (dd, *J* = 5.5, 3.0 Hz, 2H), 7.59 (d, *J* = 8.0 Hz, 2H), 7.44 (d, *J* = 8.0 Hz, 2H), 4.78 (d, *J* = 11.9 Hz, 1H), 4.68 (d, *J* = 11.9 Hz, 1H), 4.66 – 4.60 (m, 1H), 3.71 (d, *J* = 11.0 Hz, 1H), 2.96 (dp, *J* = 11.1, 8.3 Hz, 1H), 2.92 – 2.84 (m, 2H), 2.52 – 2.43 (m, 2H), 2.27 (ddd, *J* = 10.8, 8.2, 4.8 Hz, 1H), 2.07 – 1.99 (m, 2H), 1.75 (dd, *J* = 11.8, 8.7 Hz, 1H).

^{13}C NMR (151 MHz, $CDCl_3$) δ 170.9, 170.9, 168.6, 140.6, 134.1, 132.1, 130.1 (q, *J* = 32.5 Hz), 128.8, 125.7 (q, *J* = 3.8 Hz), 124.2 (q, *J* = 272.0 Hz), 123.2, 94.8, 74.2, 57.9, 40.5, 40.2, 40.2, 39.6, 38.6, 33.5, 33.3.

^{19}F NMR (565 MHz, $CDCl_3$) δ -62.56.

HRMS (+p APCI) calcd. for $[C_{26}H_{22}O_4N^{35}Cl_3F_3]$ ($[M+H]^+$) 574.0561 found 574.0574.

SFC (CEL1, 10% (50% methanol in isopropanol with 0.2% Formic Acid) in CO_2, 1.5 mL/min, 1.0 mg/ml, UV 230 nm) retention times of 9.38 min (major) and 10.07 min (minor) 97% ee.

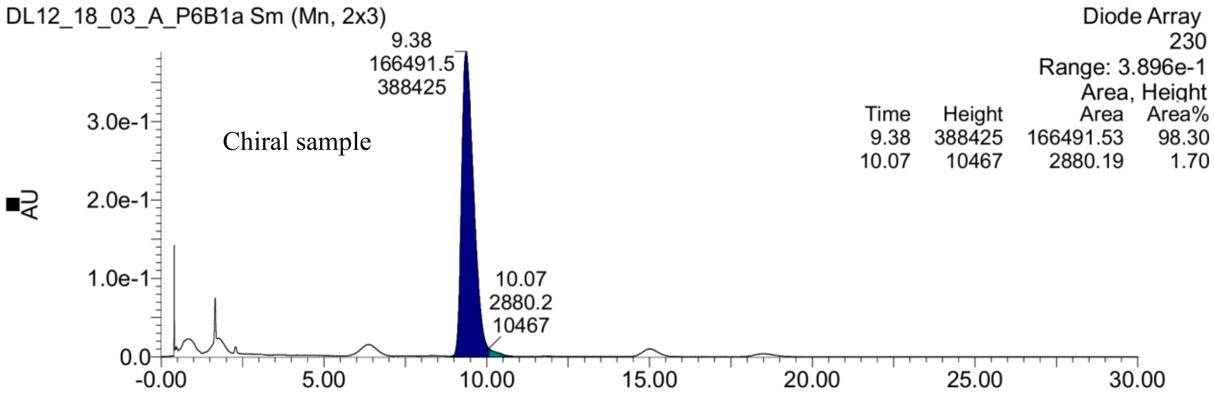

DL12_18_03_A_P6B1a Sm (Mn, 2x3)
Diode Array
230
Range: 3.896e-1
Area, Height
Time Height Area Area%
9.38 388425 166491.53 98.30
10.07 10467 2880.19 1.70
Chiral sample
9.38
166491.5
388425
10.07
2880.2
10467
AU

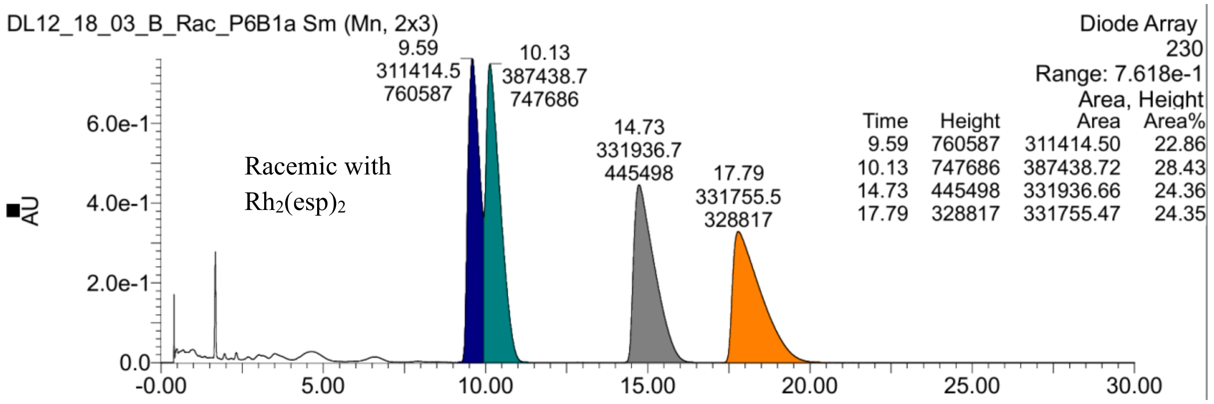

DL12_18_03_B_Rac_P6B1a Sm (Mn, 2x3)
Diode Array
230
Range: 7.618e-1
Area, Height
Time Height Area Area%
9.59 760587 311414.50 22.86
10.13 747686 387438.72 28.43
14.73 445498 331936.66 24.36
17.79 328817 331755.47 24.35
Racemic with
Rh2(esp)2
9.59
311414.5
760587
10.13
387438.7
747686
14.73
331936.7
445498
17.79
331755.5
328817
AU

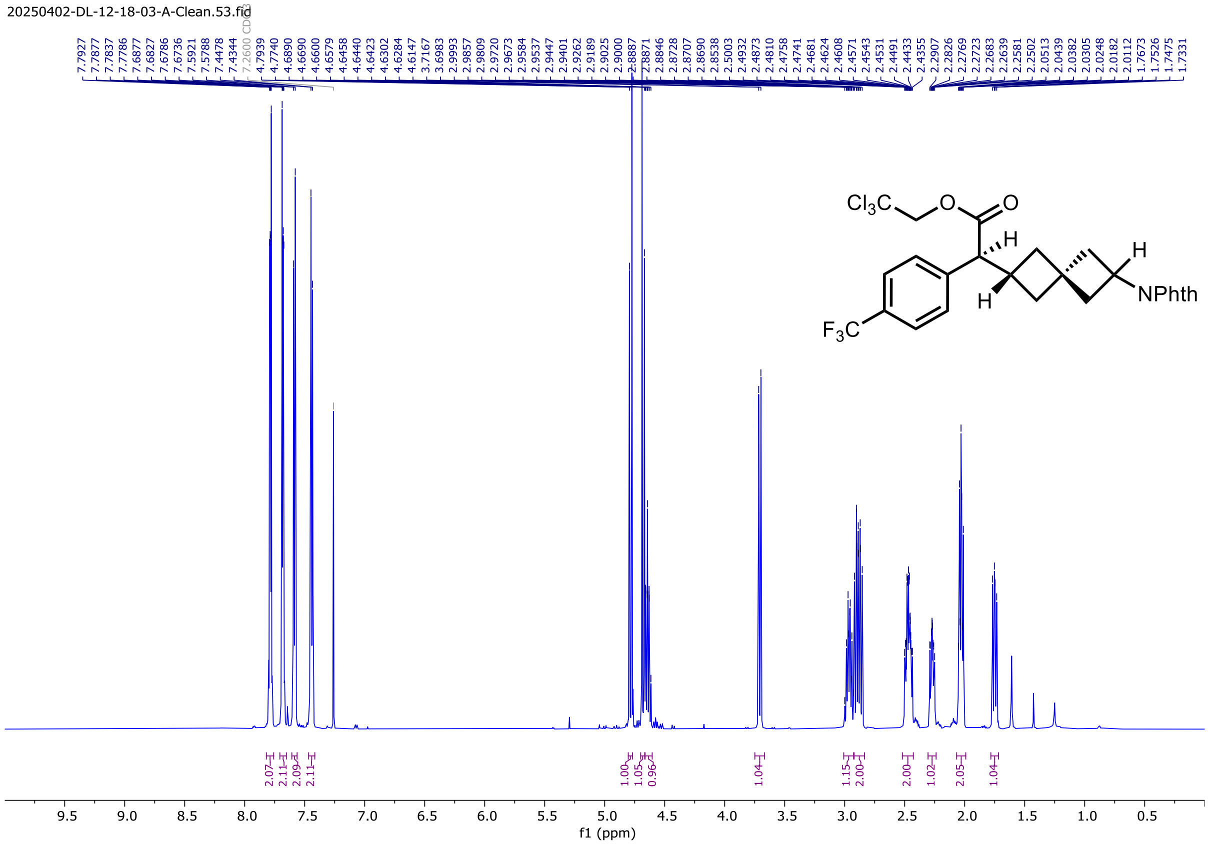

20250402-DL-12-18-03-A-Clean.53.fid
Cl3C
O
O
H
H
H
NPhth
F3C
f1 (ppm)

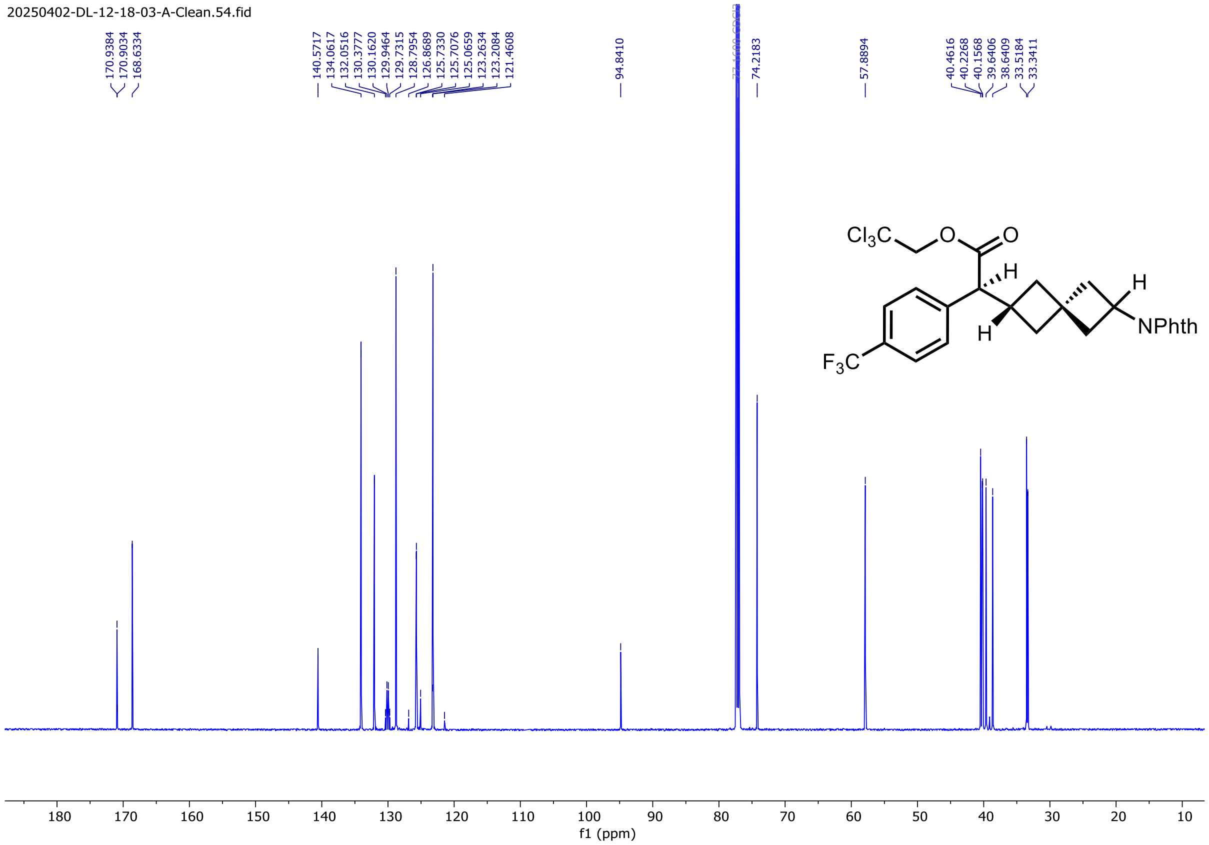
20250402-DL-12-18-03-A-Clean.54.fid
170.9384
170.9034
168.6334
140.5717
134.0617
132.0516
130.3777
130.1620
129.9464
129.7315
128.7954
126.8689
125.7330
125.7076
125.0659
123.2634
123.2084
121.4608
94.8410
74.2183
57.8894
40.4616
40.2268
40.1568
39.6406
38.6409
33.5184
33.3411
Cl_3C
O
O
H
H
H
NPhth
F_3C
f1 (ppm)

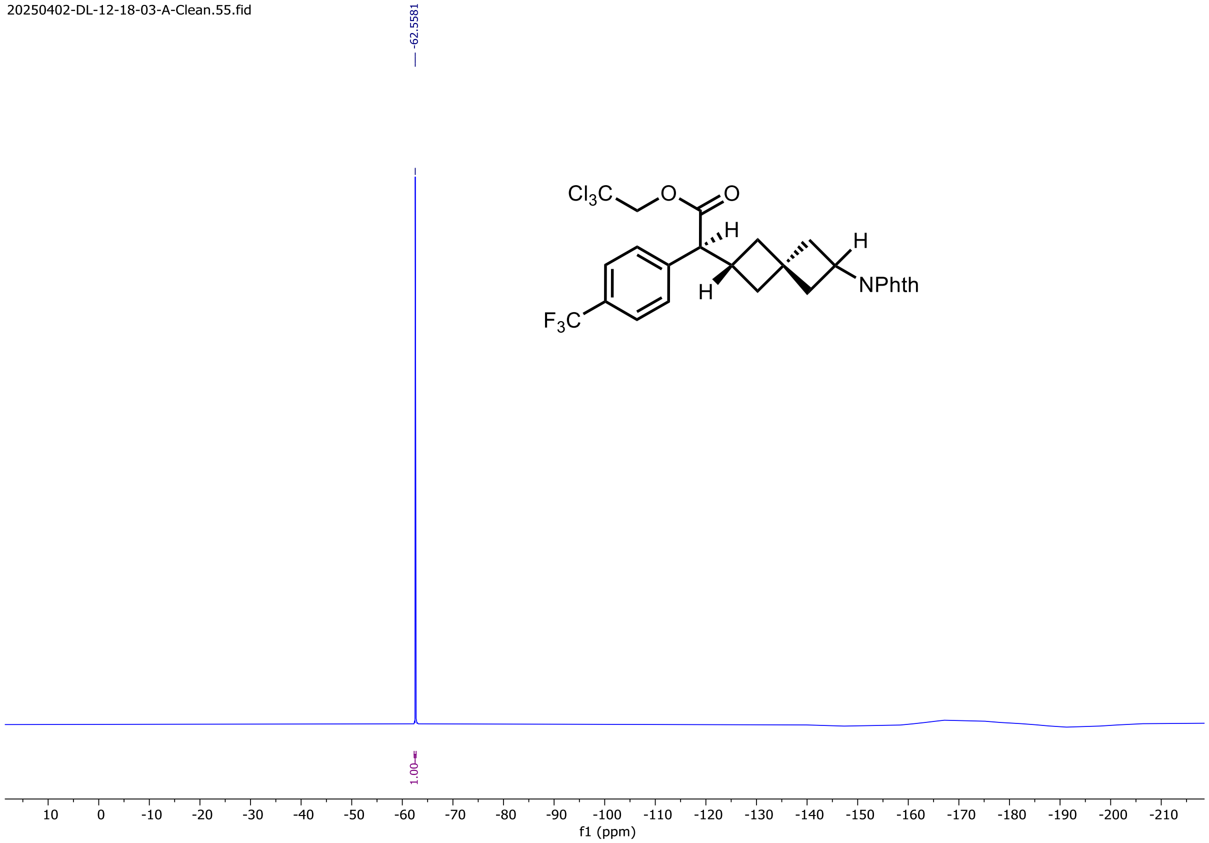
20250402-DL-12-18-03-A-Clean.55.fid
-62.5581
Cl_3C
O
O
H
H
H
NPhth
F_3C
1.00
f1 (ppm)

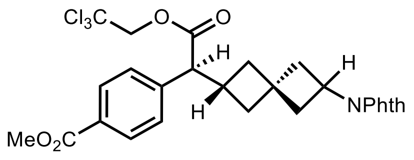

methyl 4-((1S)-1-(6-(1,3-dioxoisoindolin-2-yl)spiro[3.3]heptan-2-yl)-2-oxo-2-(2,2,2-trichloroethoxy)ethyl)benzoate (Compound 25)

Prepared according to general procedure for C-H functionalization, 2-(spiro[3.3]heptan-2-yl)isoindoline-1,3-dione (48.3 mg, 0.2 mmol, 1.0 equiv), Rh_2(*S*-MegaBNP)$_4$ (3.4 mg, 0.0001 mmol, 0.005 equiv), molecular sieve 4Å (100 wt%) and 2,2,2- HFIP (5 µL, 8.40 mg, 0.05 mmol, 0.25 equiv) in 0.5 ml CH_2Cl_2 were added a solution of methyl 4-(1-diazo-2-oxo-2-(2,2,2-trichloroethoxy)ethyl)benzoate (141.0 mg, 0.4 mmol, 2.0 equiv) in 2.0 ml CH_2Cl_2 at 39℃ in 3 hours. The crude mixture was purified by flash chromatography (SiO_2, gradient 0%-25% Et_2O in hexane) afforded **compound 25** as a white amorphous solid (93.5 mg, 83% yield, 95% ee, 17:1 dr).

R_f (1Hex/2Et_2O) = 0.50 (CAM, UV 254 nm)

$[\alpha]^{20}_D$: 20.3° (c = 1.94 g/100 ml, $CHCl_3$, 95% ee)

^{1}H NMR (600 MHz, $CDCl_3$) δ 7.99 (d, *J* = 8.2 Hz, 2H), 7.79 (dd, *J* = 5.4, 3.1 Hz, 2H), 7.68 (dd, *J* = 5.4, 3.0 Hz, 2H), 7.39 (d, *J* = 8.2 Hz, 2H), 4.77 (d, *J* = 12.0 Hz, 1H), 4.69 (d, *J* = 12.0 Hz, 1H), 4.64 (p, *J* = 9.0 Hz, 1H), 3.91 (s, 3H), 3.70 (d, *J* = 11.0 Hz, 1H), 3.05 – 2.93 (m, 1H), 2.93 – 2.80 (m, 2H), 2.56 – 2.41 (m, 2H), 2.26 (ddd, *J* = 12.2, 8.3, 4.9 Hz, 1H), 2.01 (t, *J* = 9.6 Hz, 2H), 1.75 (dd, *J* = 11.6, 8.9 Hz, 1H).

^{13}C NMR (151 MHz, $CDCl_3$) δ 171.0, 168.6, 166.9, 141.7, 134.1, 132.1, 130.1, 129.7, 128.5, 123.2, 94.9, 74.2, 58.1, 52.3, 40.5, 40.3, 40.2, 39.7, 38.6, 33.5, 33.3.

HRMS (+p APCI) calcd. for $[C_{27}H_{25}O_6N^{35}Cl_3]$ ($[M+H]^+$) 564.0742 found 564.0754.

HPLC (Chiralpak ADH column, 10% isopropanol in hexane, 1.0 $mLmin^{-1}$, 1.0 $mgmL^{-1}$, 60 min, UV 230 nm) retention times of 33.1 min (minor) and 40.3 min (major), 95% ee.

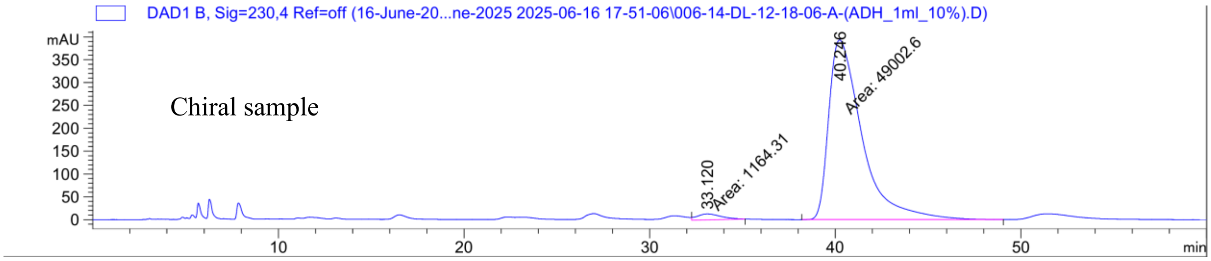

Signal 2: DAD1 B, Sig=230,4 Ref=off

Peak #	RetTime [min]	Type	Width [min]	Area [mAU*s]	Height [mAU]	Area %
1	33.120	MM	1.5004	1164.31433	12.93374	2.3209
2	40.246	MM	2.0775	4.90026e4	393.12720	97.6791
Totals :				5.01669e4	406.06093	

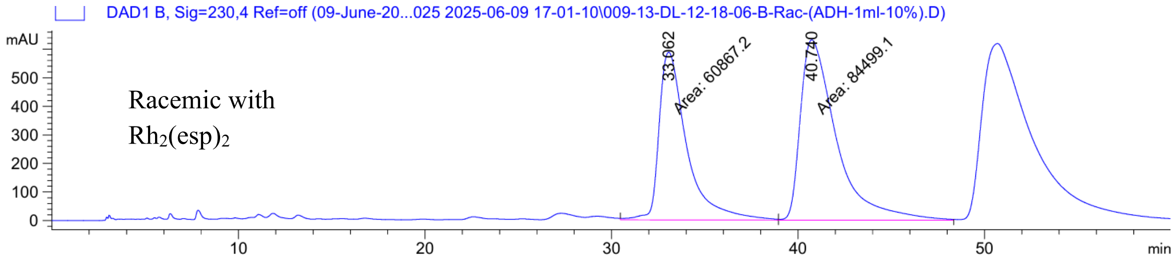

Signal 2: DAD1 B, Sig=230,4 Ref=off

Peak #	RetTime [min]	Type	Width [min]	Area [mAU*s]	Height [mAU]	Area %
1	33.062	FM	1.7280	6.08672e4	587.05896	41.8716
2	40.740	FM	2.2313	8.44991e4	631.15753	58.1284
Totals :				1.45366e5	1218.21649	

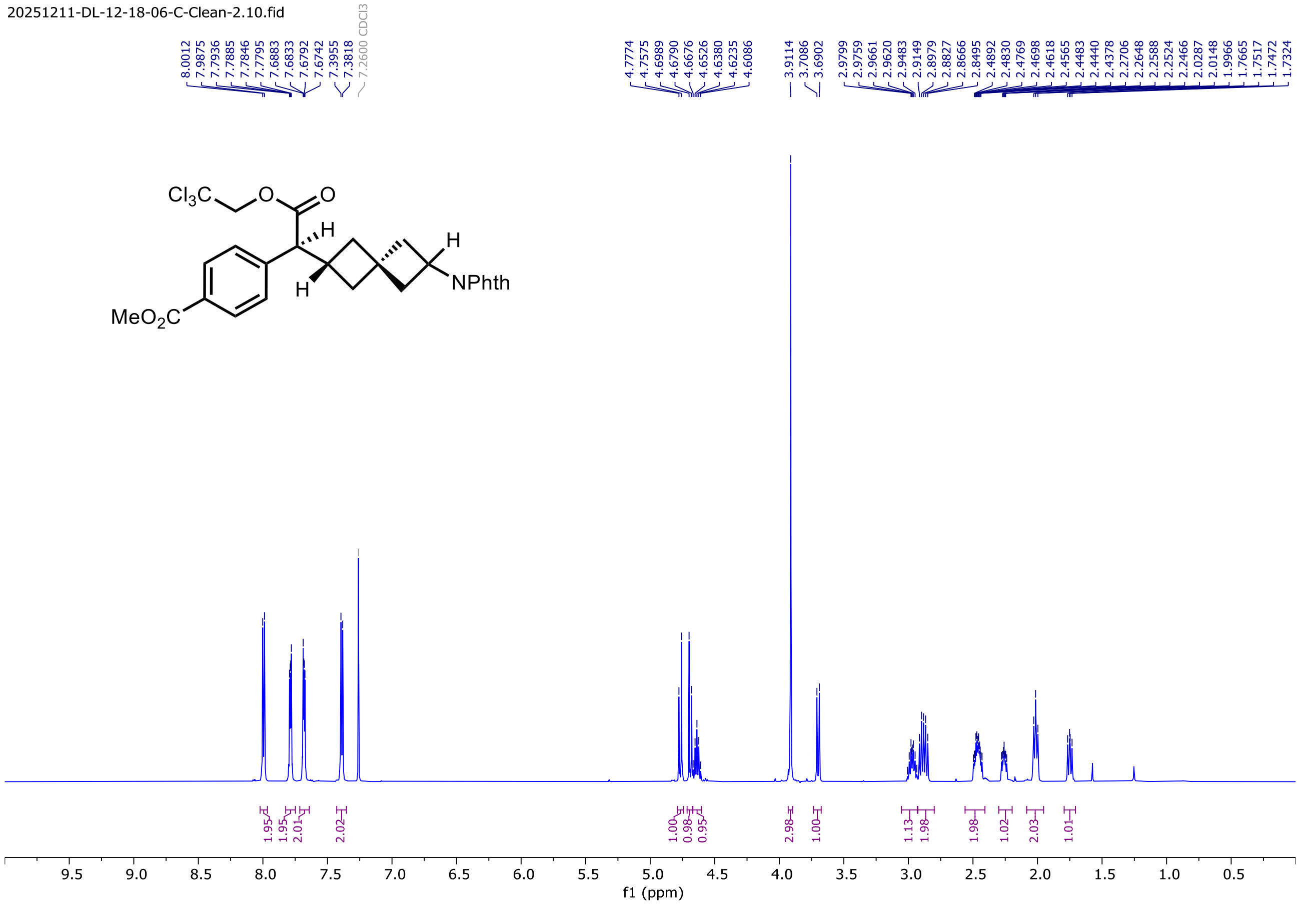

20251211-DL-12-18-06-C-Clean-2.10.fid
8.0012 7.9875 7.7936 7.7885 7.7846 7.7795 7.6883 7.6833 7.6792 7.6742 7.3955 7.3818 7.2600 CDCl3
4.7774 4.7575 4.6989 4.6790 4.6676 4.6526 4.6380 4.6235 4.6086
3.9114 3.7086 3.6902
2.9799 2.9759 2.9661 2.9620 2.9483 2.9149 2.8979 2.8827 2.8666 2.8495 2.4892 2.4830 2.4769 2.4698 2.4618 2.4565 2.4483 2.4440 2.4378 2.2706 2.2648 2.2588 2.2524 2.2466 2.0287 2.0148 1.9966 1.7665 1.7517 1.7472 1.7324
Cl3C
O
O
H
H
H
NPhth
MeO2C
1.95 1.95 2.01 2.02 1.00 0.98 0.95 2.98 1.00 1.13 1.98 1.98 1.02 2.03 1.01
9.5 9.0 8.5 8.0 7.5 7.0 6.5 6.0 5.5 5.0 4.5 4.0 3.5 3.0 2.5 2.0 1.5 1.0 0.5
f1 (ppm)

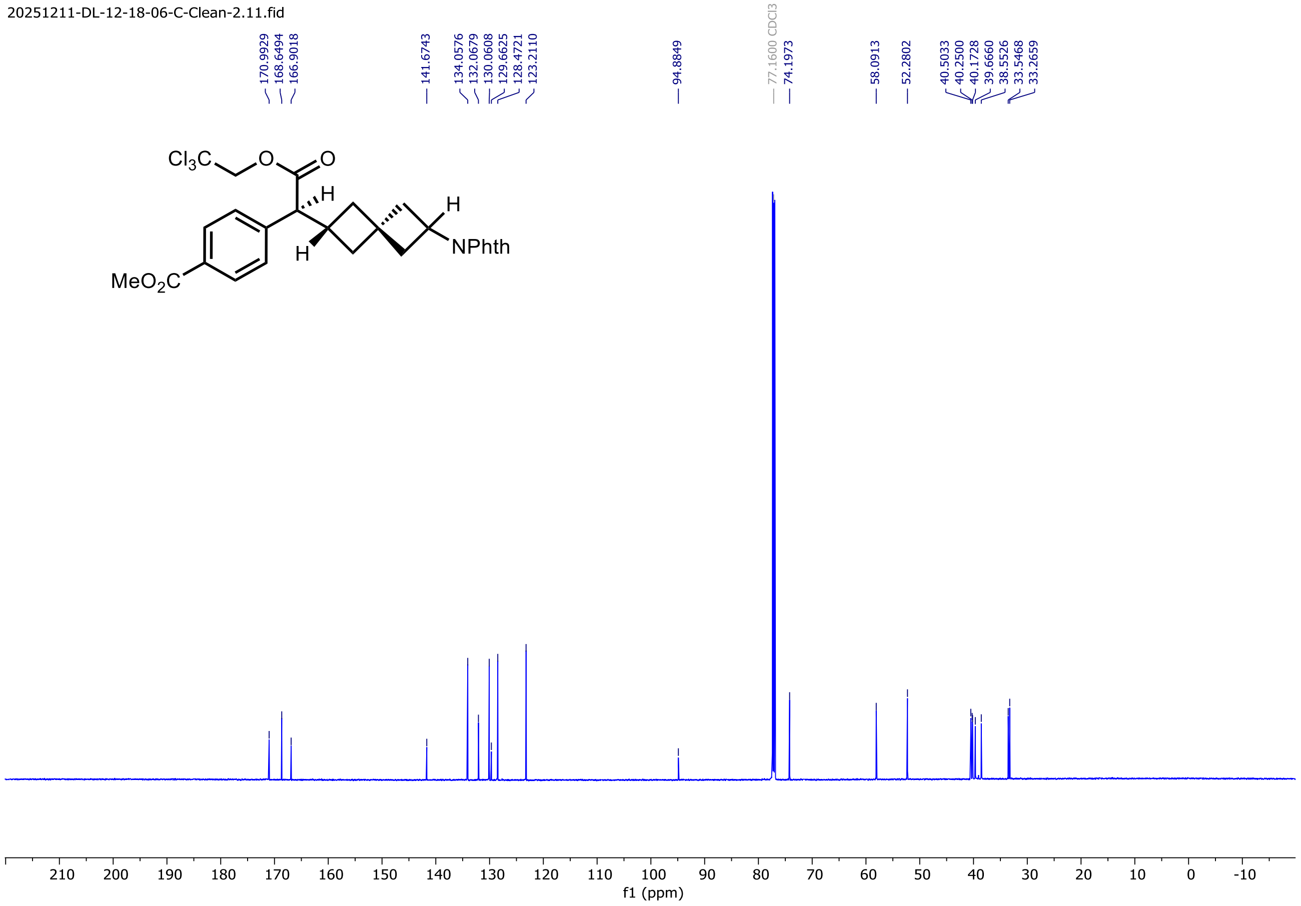

20251211-DL-12-18-06-C-Clean-2.11.fid
170.9929 168.6494 166.9018
141.6743
134.0576 132.0679 130.0608 129.6625 128.4721 123.2110
94.8849
77.1600 CDCl3
74.1973
58.0913
52.2802
40.5033 40.2500 40.1728 39.6660 38.5526 33.5468 33.2659
Cl3C
O
O
H
H
H
NPhth
MeO2C
210 200 190 180 170 160 150 140 130 120 110 100 90 80 70 60 50 40 30 20 10 0 -10
f1 (ppm)

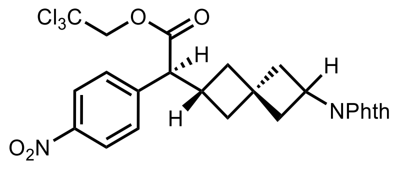

2,2,2-trichloroethyl (2S)-2-(6-(1,3-dioxoisoindolin-2-yl)spiro[3.3]heptan-2-yl)-2-(4-nitrophenyl)acetate (Compound 26)

Prepared according to general procedure for C-H functionalization, 2-(spiro[3.3]heptan-2-yl)isoindoline-1,3-dione (48.3 mg, 0.2 mmol, 1.0 equiv), Rh_2(*S*-MegaBNP)$_4$ (3.4 mg, 0.0001 mmol, 0.005 equiv), molecular sieve 4Å (100 wt%) and 2,2,2- HFIP (5 µL, 8.40 mg, 0.05 mmol, 0.25 equiv) in 0.5 ml CH_2Cl_2 were added a solution of trichloroethyl 2-(4-nitrophenyl)-2-diazoacetate (135.0 mg, 0.4 mmol, 2.0 equiv) in 2.0 ml CH_2Cl_2 at 39℃ in 3 hours. The crude mixture was purified by flash chromatography (SiO_2, gradient 0%-30% Et_2O in hexane) afforded **compound 26** as a yellow oil which turned into a yellow amorphous solid upon vaccum (79.5 mg, 71% yield, 99% ee, 10:1 dr).

R$_f$ (1Hex/2Et_2O) = 0.20 (CAM, UV 254 nm)

$[\alpha]^{20}_D$: 19.7° (c = 1.36 g/100 ml, $CHCl_3$, 99% ee)

^{1}H NMR (600 MHz, $CDCl_3$) δ 8.20 (d, *J* = 8.7 Hz, 2H), 7.79 (dd, *J* = 5.4, 3.1 Hz, 2H), 7.69 (dd, *J* = 5.5, 3.0 Hz, 2H), 7.50 (d, *J* = 8.7 Hz, 2H), 4.78 (d, *J* = 11.9 Hz, 1H), 4.71 (d, *J* = 12.0 Hz, 1H), 4.65 (p, *J* = 8.9 Hz, 1H), 3.77 (d, *J* = 11.0 Hz, 1H), 2.97 (dt, *J* = 10.7, 8.2 Hz, 1H), 2.94 – 2.85 (m, 2H), 2.54 – 2.42 (m, 2H), 2.28 (ddd, *J* = 12.3, 8.3, 4.8 Hz, 1H), 2.08 – 2.01 (m, 2H), 1.75 (dd, *J* = 11.7, 8.9 Hz, 1H).

^{13}C NMR (151 MHz, $CDCl_3$) δ 170.4, 168.6, 147.6, 143.9, 134.1, 132.0, 129.4, 124.0, 123.2, 94.7, 74.3, 57.8, 40.4, 40.3, 40.1, 39.6, 38.6, 33.6, 33.4.

HRMS (+p APCI) calcd. for [$C_{25}H_{22}O_6N_2{}^{35}Cl_3$] ($[M+H]^+$) 551.0538 found 551.0551.

HPLC (Chiralpak ADH column, 10% isopropanol in hexane, 1.0 $mLmin^{-1}$, 1.0 $mgmL^{-1}$, 90 min, UV 230 nm) retention times of 38.5 min (major) and 47.9 min (minor), 99% ee.

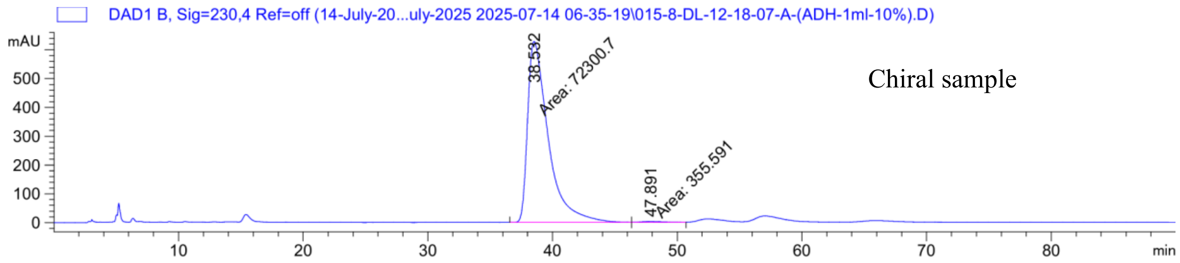

Chiral sample

Signal 2: DAD1 B, Sig=230,4 Ref=off

Peak #	RetTime [min]	Type	Width [min]	Area [mAU*s]	Height [mAU]	Area %
1	38.532	MF	1.9167	7.23007e4	628.70618	99.5106
2	47.891	FM	1.9257	355.59109	3.07754	0.4894
Totals :				7.26563e4	631.78372	

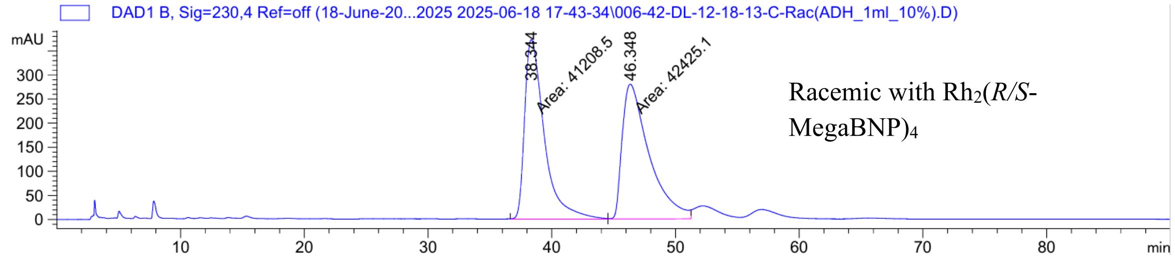

Racemic with Rh_2(*R/S*-MegaBNP)$_4$

Signal 2: DAD1 B, Sig=230,4 Ref=off

Peak #	RetTime [min]	Type	Width [min]	Area [mAU*s]	Height [mAU]	Area %
1	38.344	MF	1.8431	4.12085e4	372.63660	49.2727
2	46.348	FM	2.5246	4.24251e4	280.07928	50.7273
Totals :				8.36336e4	652.71588	

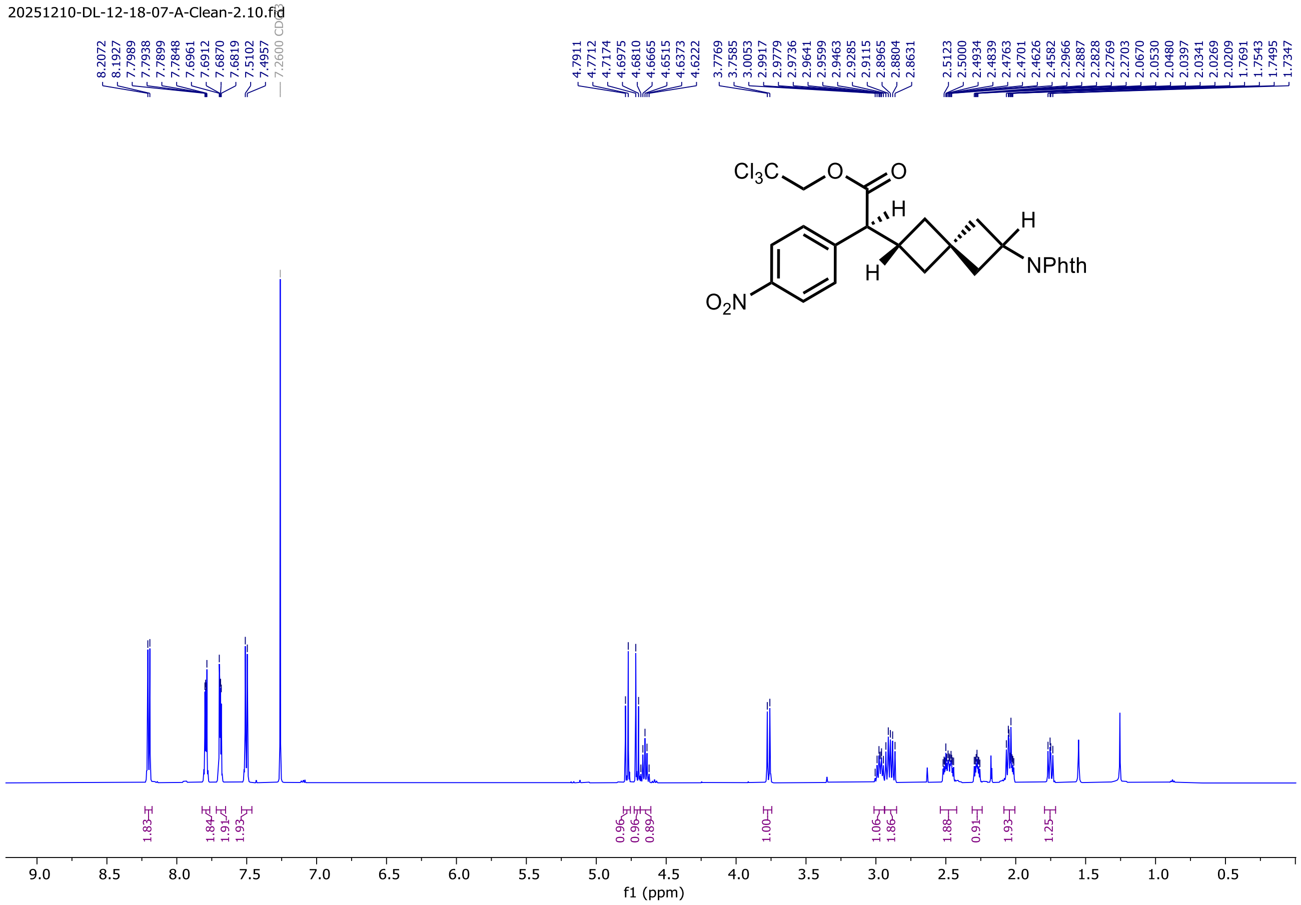

20251210-DL-12-18-07-A-Clean-2.10.fid
8.2072
8.1927
7.7989
7.7938
7.7899
7.7848
7.6961
7.6912
7.6870
7.6819
7.5102
7.4957
7.2600 CDCl3
4.7911
4.7712
4.7174
4.6975
4.6810
4.6665
4.6515
4.6373
4.6222
3.7769
3.7585
3.0053
2.9917
2.9779
2.9736
2.9641
2.9599
2.9463
2.9285
2.9115
2.8965
2.8804
2.8631
2.5123
2.5000
2.4934
2.4839
2.4763
2.4701
2.4626
2.4582
2.2966
2.2887
2.2828
2.2769
2.2703
2.0670
2.0530
2.0480
2.0397
2.0341
2.0269
2.0209
1.7691
1.7543
1.7495
1.7347
Cl3C
O
O
H
H
H
NPhth
O2N
1.83
1.84
1.91
1.93
0.96
0.96
0.89
1.00
1.06
1.86
1.88
0.91
1.93
1.25
f1 (ppm)

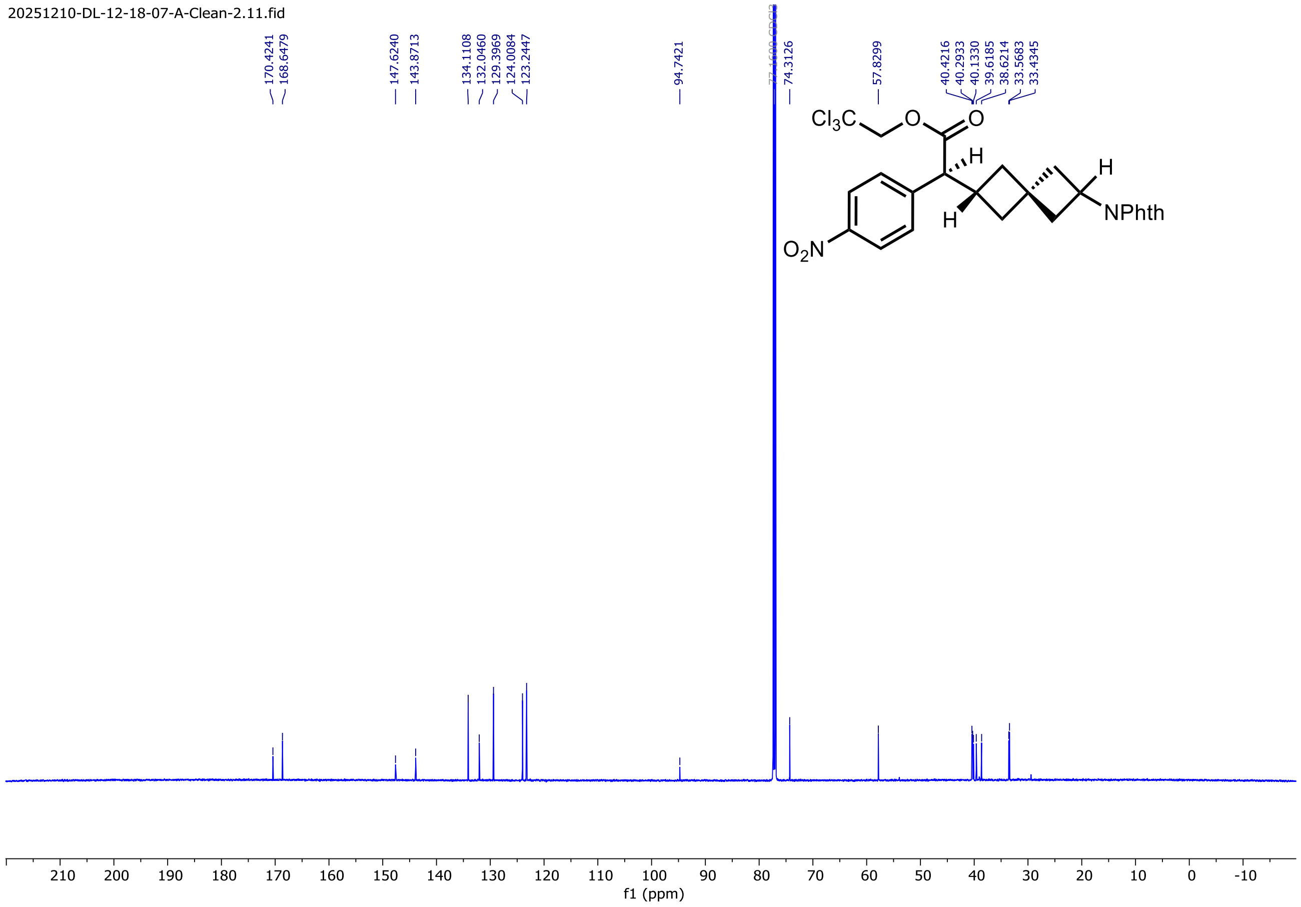

20251210-DL-12-18-07-A-Clean-2.11.fid
170.4241
168.6479
147.6240
143.8713
134.1108
132.0460
129.3969
124.0084
123.2447
94.7421
74.3126
57.8299
40.4216
40.2933
40.1330
39.6185
38.6214
33.5683
33.4345
Cl3C
O
O
H
H
H
NPhth
O2N
f1 (ppm)

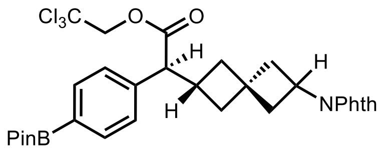

2,2,2-trichloroethyl (2S)-2-(6-(1,3-dioxoisoindolin-2-yl)spiro[3.3]heptan-2-yl)-2-(4-(4,4,5,5-tetramethyl-1,3,2-dioxaborolan-2-yl)phenyl)acetate (Compound 27)

Prepared according to general procedure for C-H functionalization, 2-(spiro[3.3]heptan-2-yl)isoindoline-1,3-dione (48.3 mg, 0.2 mmol, 1.0 equiv), Rh_2(*S*-MegaBNP)$_4$ (3.4 mg, 0.0001 mmol, 0.005 equiv), molecular sieve 4Å (100 wt%) and 2,2,2- HFIP (5 µL, 8.40 mg, 0.05 mmol, 0.25 equiv) in 0.5 ml CH_2Cl_2 were added a solution of 2,2,2-trichloroethyl 2-diazo-2-(4-(4,4,5,5-tetramethyl-1,3,2-dioxaborolan-2-yl)phenyl)acetate (168.0 mg, 0.4 mmol, 2.0 equiv) in 2.0 ml CH_2Cl_2 at 39℃ in 3 hours. The crude mixture was purified by flash chromatography (SiO_2, gradient 0%-25% Et_2O in hexane) afforded **compound 27** as a white amorphous solid (92.4 mg, 73% yield, 96% ee, 11:1 dr).

R_f (1Hex/2Et_2O) = 0.50 (CAM, UV 254 nm)

$[\alpha]^{20}_D$: 21.3° (c = 0.79 g/100 ml, $CHCl_3$, 96% ee)

^{1}H NMR (600 MHz, $CDCl_3$) δ 7.79 (dd, *J* = 5.4, 3.1 Hz, 2H), 7.76 (d, *J* = 8.1 Hz, 2H), 7.68 (dd, *J* = 5.5, 3.0 Hz, 2H), 7.32 (d, *J* = 8.1 Hz, 2H), 4.77 (d, *J* = 12.0 Hz, 1H), 4.69 – 4.59 (m, 2H), 3.65 (d, *J* = 11.0 Hz, 1H), 2.97 (dt, *J* = 10.8, 8.2 Hz, 1H), 2.91 – 2.83 (m, 2H), 2.52 – 2.39 (m, 2H), 2.25 (ddd, *J* = 12.7, 8.2, 4.9 Hz, 1H), 2.06 – 1.92 (m, 2H), 1.75 (dd, *J* = 11.9, 8.7 Hz, 1H), 1.34 (s, 12H).

^{13}C NMR (151 MHz, $CDCl_3$) δ 171.4, 168.7, 139.7, 135.2, 134.0, 132.1, 127.8, 123.2, 95.0, 84.0, 74.1, 58.4, 40.6, 40.2, 39.7, 38.5, 33.6, 33.2, 25.0.

HRMS (+p APCI) calcd. for [$C_{31}H_{34}O_6N^{10}B^{35}Cl_3$] ($[M+H]^+$) 631.1576 found 631.1592.

HPLC (Chiralpak ADH column, 5% isopropanol in hexane, 1.0 mLmin^{-1}, 1.0 mgmL^{-1}, 60 min, UV 230 nm) retention times of 16.9 min (major) and 46.9 min (minor), 96% ee.

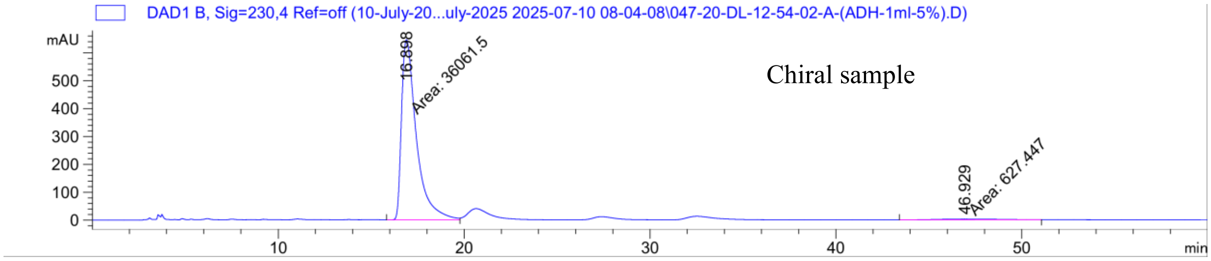

Signal 2: DAD1 B, Sig=230,4 Ref=off

Peak #	RetTime [min]	Type	Width [min]	Area [mAU*s]	Height [mAU]	Area %
1	16.898	MF	0.9300	3.60615e4	646.25623	98.2898
2	46.929	MM	3.7173	627.44678	2.81319	1.7102

Totals : 3.66890e4 649.06941

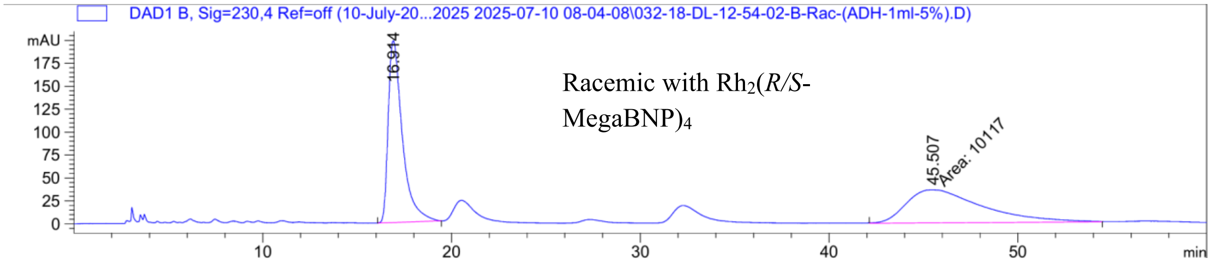

Signal 2: DAD1 B, Sig=230,4 Ref=off

Peak #	RetTime [min]	Type	Width [min]	Area [mAU*s]	Height [mAU]	Area %
1	16.914	BB	0.6158	1.03258e4	198.20340	50.5108
2	45.507	MM	4.6570	1.01170e4	36.20674	49.4892

Totals : 2.04428e4 234.41014

20251210-DL-12-54-02-A-Clean-2.10.fid

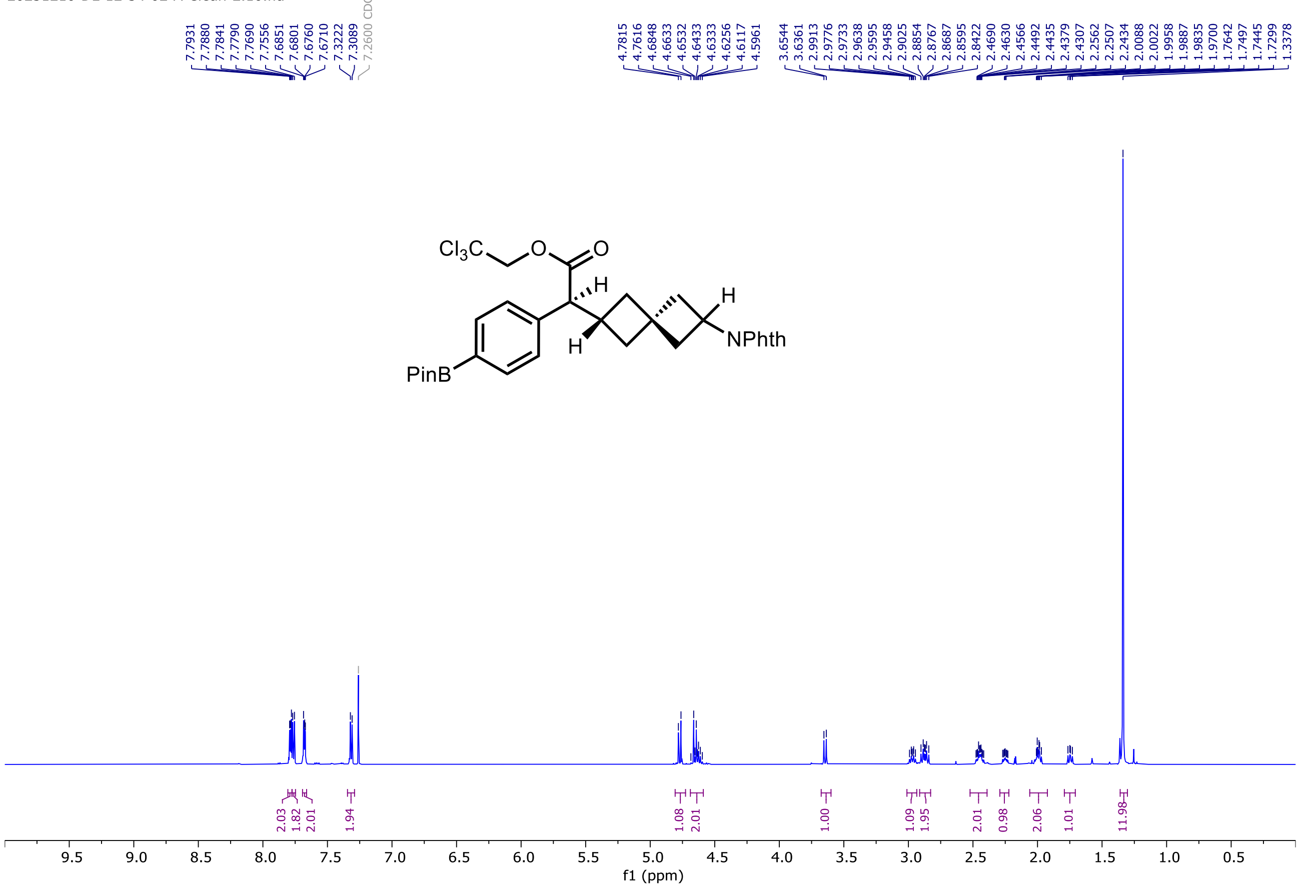

20251210-DL-12-54-02-A-Clean-2.11.fid

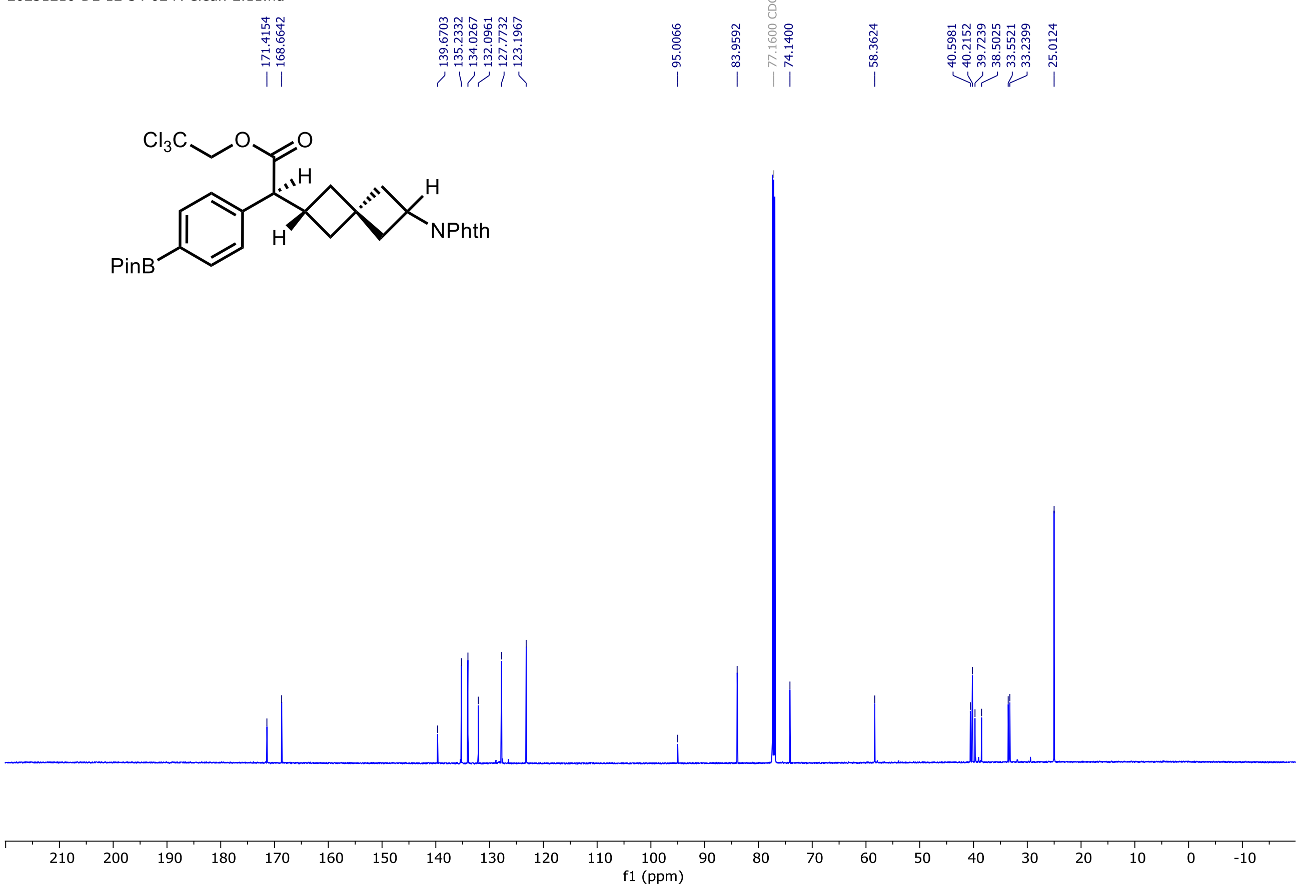

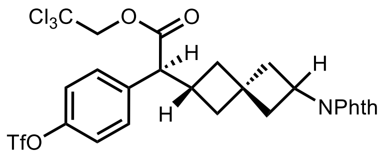

2,2,2-trichloroethyl (2S)-2-(6-(1,3-dioxoisoindolin-2-yl)spiro[3.3]heptan-2-yl)-2-(4-(((trifluoromethyl)sulfonyl)oxy)phenyl)acetate (Compound 28)

Prepared according to general procedure for C-H functionalization, 2-(spiro[3.3]heptan-2-yl)isoindoline-1,3-dione (48.3 mg, 0.2 mmol, 1.0 equiv), Rh_2(*S*-MegaBNP)$_4$ (3.4 mg, 0.0001 mmol, 0.005 equiv), molecular sieve 4Å (100 wt%) and 2,2,2- HFIP (5 μL, 8.40 mg, 0.05 mmol, 0.25 equiv) in 0.5 ml CH_2Cl_2 were added a solution of 2,2,2-trichloroethyl 2-diazo-2-(4-(((trifluoromethyl)sulfonyl)oxy)phenyl)acetate (177.0 mg, 0.4 mmol, 2.0 equiv) in 2.0 ml CH_2Cl_2 at 39℃ in 3 hours. The crude mixture was purified by flash chromatography (SiO_2, gradient 0%-25% Et_2O in hexane) afforded **compound 28** as a white amorphous solid (105.1 mg, 80% yield, 99% ee, 16:1 dr).

R_f (1Hex/2Et_2O) = 0.50 (CAM, UV 254 nm)

$[\alpha]^{20}_D$: 15.3° (c = 1.77 g/100 ml, $CHCl_3$, 99% ee)

^{1}H NMR (600 MHz, $CDCl_3$) δ 7.79 (dd, *J* = 5.5, 3.0 Hz, 2H), 7.69 (dd, *J* = 5.5, 3.0 Hz, 2H), 7.41 (d, *J* = 8.7 Hz, 2H), 7.24 (d, *J* = 8.7 Hz, 2H), 4.77 (d, *J* = 11.9 Hz, 1H), 4.72 – 4.61 (m, 2H), 3.68 (d, *J* = 11.0 Hz, 1H), 2.99 – 2.81 (m, 3H), 2.51 – 2.43 (m, 2H), 2.27 (ddd, *J* = 12.2, 8.2, 4.7 Hz, 1H), 2.09 – 1.98 (m, 2H), 1.75 (dd, *J* = 11.8, 8.7 Hz, 1H).

^{13}C NMR (151 MHz, $CDCl_3$) δ 170.9, 168.6, 149.1, 137.2, 134.1, 132.1, 130.3, 123.2, 121.7, 118.9 (q, *J* = 320.8 Hz), 94.8, 74.2, 57.4, 53.6, 40.4, 40.2, 40.2, 39.6, 38.7, 33.5, 33.3.

^{19}F NMR (565 MHz, $CDCl_3$) δ -72.78.

HRMS (+p APCI) calcd. for $[C_{26}H_{22}O_7N^{35}Cl_3F_3{}^{32}S]$ ($[M+H]^+$) 654.0129 found 654.0145.

SFC (AMY1, 10% (50% methanol in isopropanol with 0.2% Formic Acid) in CO_2, 2.5 mL/min, 1.0 mg/ml, UV 230 nm) retention times of 3.67 min (major) and 5.05 min (minor), 99% ee.

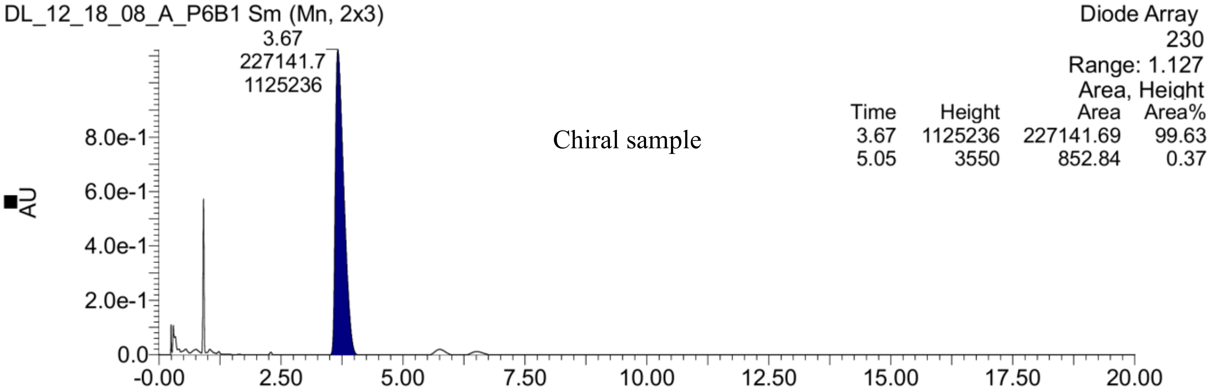

DL_12_18_08_A_P6B1 Sm (Mn, 2x3)
Diode Array
230
Range: 1.127
Area, Height
3.67
227141.7
1125236
Chiral sample
Time Height Area Area%
3.67 1125236 227141.69 99.63
5.05 3550 852.84 0.37
AU

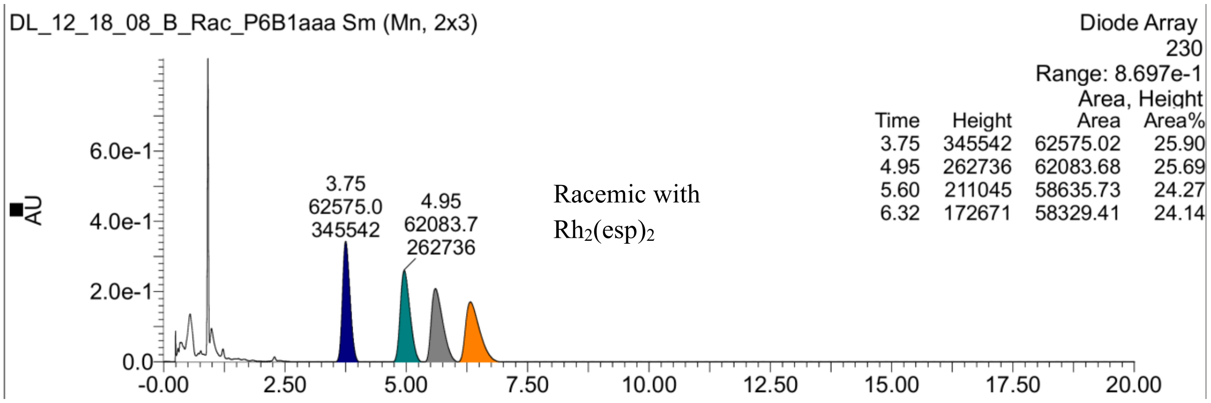

DL_12_18_08_B_Rac_P6B1aaa Sm (Mn, 2x3)
Diode Array
230
Range: 8.697e-1
Area, Height
3.75
62575.0
345542
4.95
62083.7
262736
Racemic with
Rh2(esp)2
Time Height Area Area%
3.75 345542 62575.02 25.90
4.95 262736 62083.68 25.69
5.60 211045 58635.73 24.27
6.32 172671 58329.41 24.14
AU

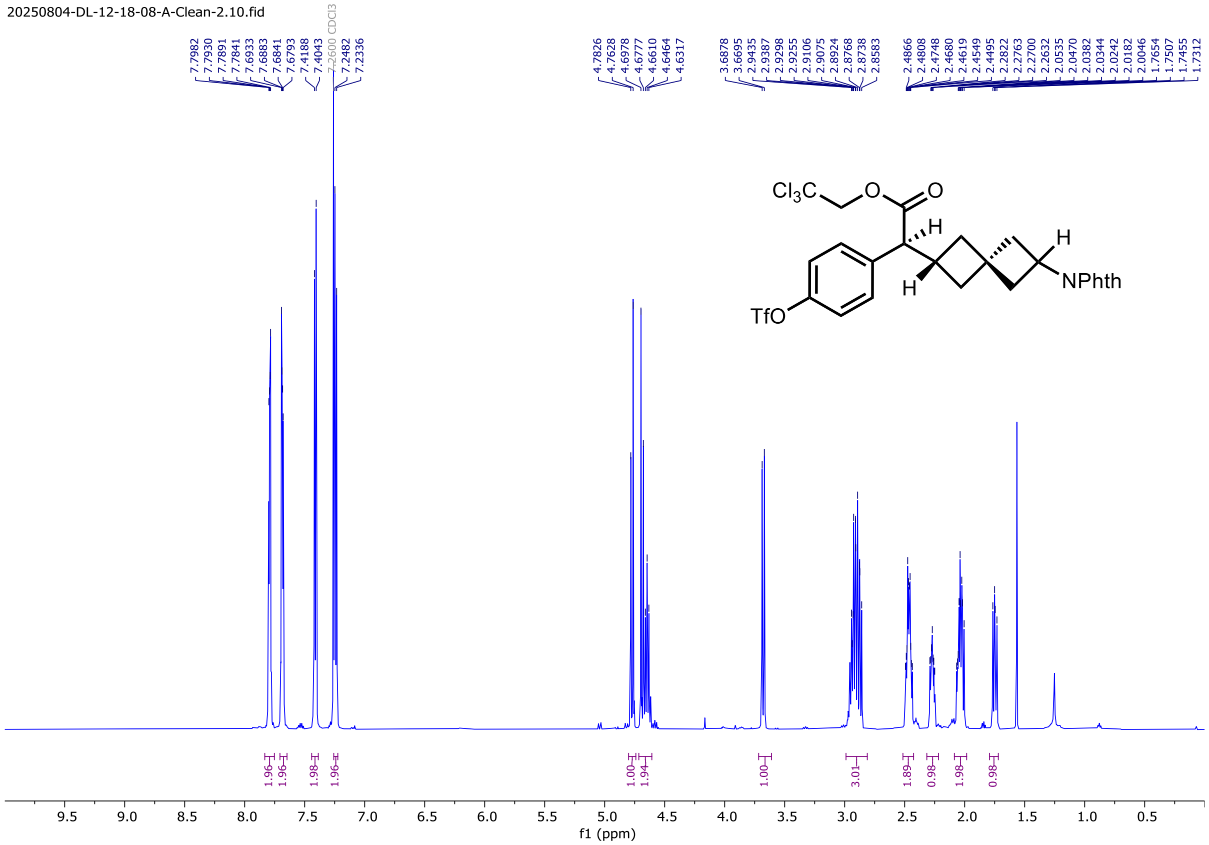

20250804-DL-12-18-08-A-Clean-2.10.fid
Cl3C
O
O
H
H
H
NPhth
TfO
f1 (ppm)

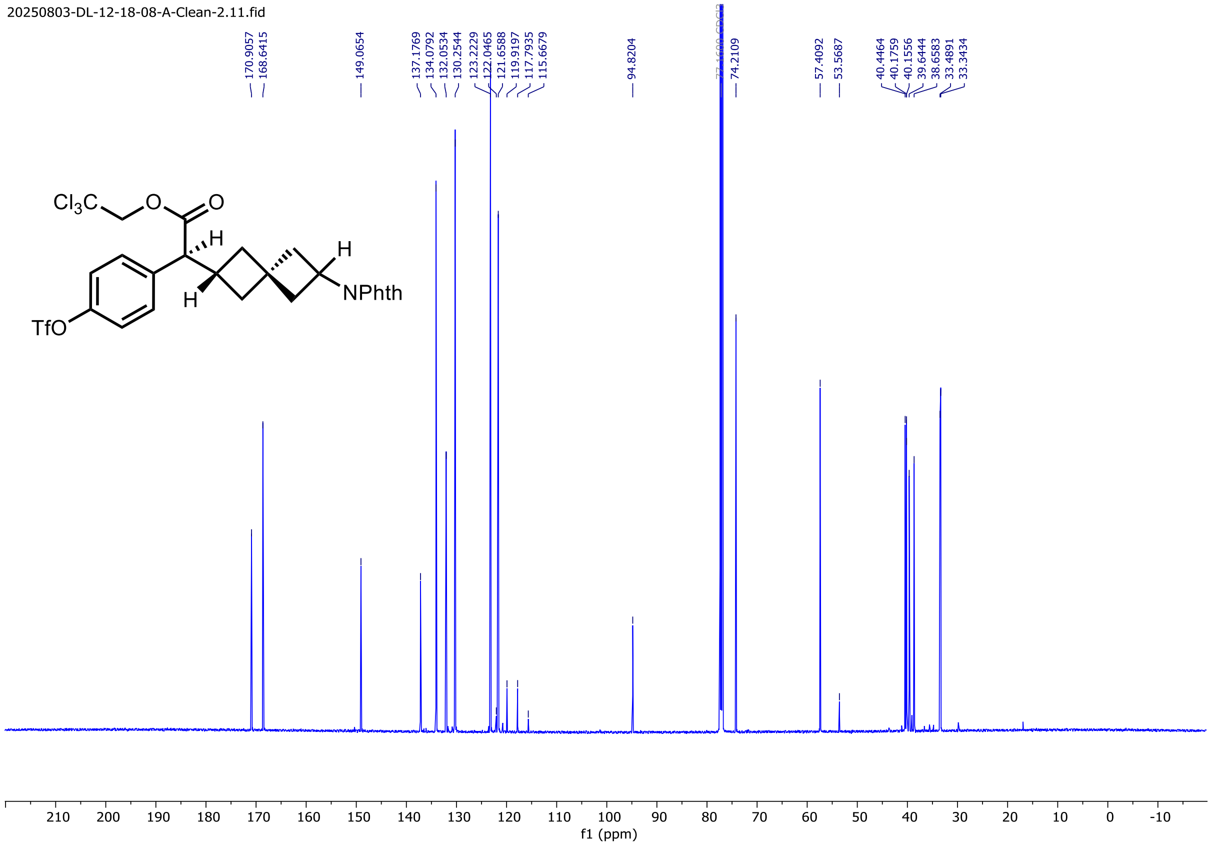
20250803-DL-12-18-08-A-Clean-2.11.fid
170.9057
168.6415
149.0654
137.1769
134.0792
132.0534
130.2544
123.2229
122.0465
121.6588
119.9197
117.7935
115.6679
94.8204
74.2109
57.4092
53.5687
40.4464
40.1759
40.1556
39.6444
38.6583
33.4891
33.3434
Cl3C
O
O
H
H
H
NPhth
TfO
210 200 190 180 170 160 150 140 130 120 110 100 90 80 70 60 50 40 30 20 10 0 -10
f1 (ppm)

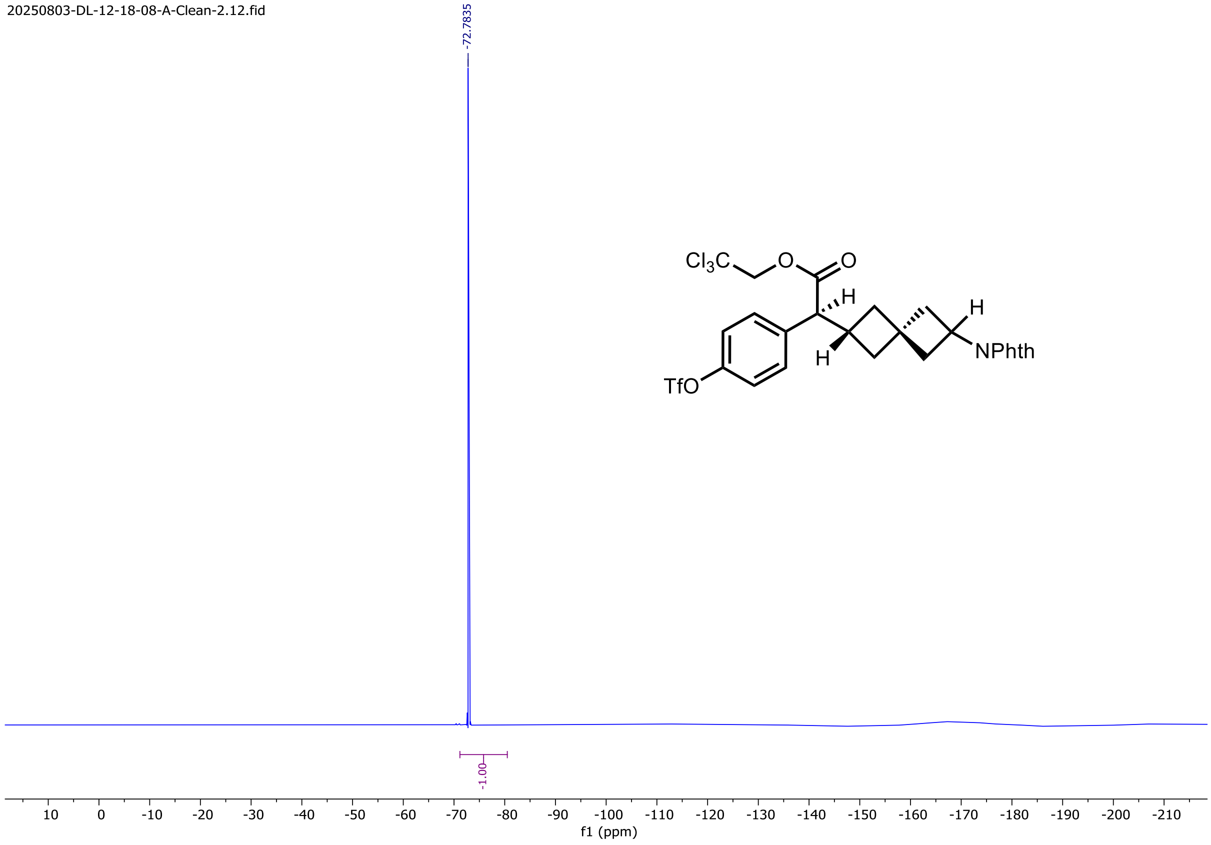
20250803-DL-12-18-08-A-Clean-2.12.fid
-72.7835
Cl3C
O
O
H
H
H
NPhth
TfO
-1.00
10 0 -10 -20 -30 -40 -50 -60 -70 -80 -90 -100 -110 -120 -130 -140 -150 -160 -170 -180 -190 -200 -210
f1 (ppm)

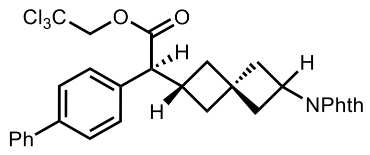

2,2,2-trichloroethyl (2S)-2-([1,1'-biphenyl]-4-yl)-2-(6-(1,3-dioxoisoindolin-2-yl)spiro[3.3]heptan-2-yl)acetate (Compound 29)

Prepared according to general procedure for C-H functionalization, 2-(spiro[3.3]heptan-2-yl)isoindoline-1,3-dione (48.3 mg, 0.2 mmol, 1.0 equiv), Rh_2(*S*-MegaBNP)$_4$ (3.4 mg, 0.0001 mmol, 0.005 equiv), molecular sieve 4Å (100 wt%) and 2,2,2- HFIP (5 µL, 8.40 mg, 0.05 mmol, 0.25 equiv) in 0.5 ml CH_2Cl_2 were added a solution of 2,2,2-trichloroethyl 2-([1,1'-biphenyl]-4-yl)-2-diazoacetate (148.0 mg, 0.4 mmol, 2.0 equiv) in 2.0 ml CH_2Cl_2 at 39℃ in 3 hours. The crude mixture was purified by flash chromatography (SiO_2, gradient 0%-25% Et_2O in hexane) afforded **compound 29** as a white amorphous solid (79.8 mg, 69% yield, 99% ee, 21:1 dr).

R_f (1Hex/2Et_2O) = 0.50 (CAM, UV 254 nm)

$[\alpha]^{20}_D$: 29.1° (c = 0.38 g/100 ml, $CHCl_3$, 99% ee)

^1H NMR (600 MHz, $CDCl_3$) δ 7.79 (dd, *J* = 5.5, 3.0 Hz, 2H), 7.68 (dd, *J* = 5.5, 3.0 Hz, 2H), 7.58 (d, *J* = 7.5 Hz, 2H), 7.55 (d, *J* = 8.2 Hz, 2H), 7.44 (t, *J* = 7.5 Hz, 2H), 7.39 (d, *J* = 8.2 Hz, 2H), 7.34 (t, *J* = 7.5 Hz, 1H), 4.81 (d, *J* = 11.9 Hz, 1H), 4.70 – 4.62 (m, 2H), 3.69 (d, *J* = 11.0 Hz, 1H), 3.01 (dp, *J* = 11.1, 8.2 Hz, 1H), 2.93 – 2.84 (m, 2H), 2.51 – 2.43 (m, 2H), 2.28 (ddd, *J* = 11.2, 8.2, 4.8 Hz, 1H), 2.09 (ddd, *J* = 12.0, 7.9, 4.1 Hz, 1H), 2.03 (dd, *J* = 11.2, 8.2 Hz, 1H), 1.81 (dd, *J* = 11.8, 8.7 Hz, 1H).

^{13}C NMR (151 MHz, $CDCl_3$) δ 171.7, 168.7, 140.8, 140.7, 135.7, 134.0, 132.1, 128.9, 128.8, 127.5, 127.2, 123.2, 95.0, 74.2, 57.9, 40.6, 40.3, 40.2, 39.7, 38.7, 33.6, 33.3. (*missing 1 carbon*)

HRMS (+p APCI) calcd. for [$C_{31}H_{27}O_4N^{35}Cl_3$] ($[M+H]^+$) 582.1000 found 582.1014.

HPLC (Chiralpak ADH column, 10% isopropanol in hexane, 1.0 $mLmin^{-1}$, 1.0 $mgmL^{-1}$, 60 min, UV 230 nm) retention times of 18.9 min (major) and 22.9 min (minor), 99% ee.

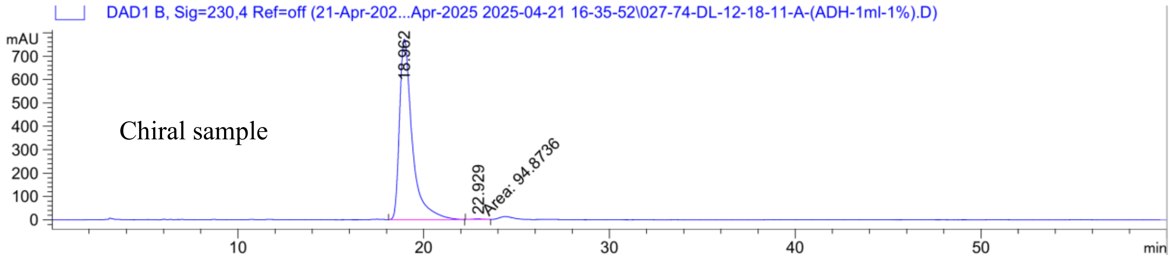

Signal 2: DAD1 B, Sig=230,4 Ref=off

Peak #	RetTime [min]	Type	Width [min]	Area [mAU*s]	Height [mAU]	Area %
1	18.962	BB	0.5629	3.67521e4	771.34351	99.7425
2	22.929	MM	0.6723	94.87361	2.35197	0.2575
Totals :				3.68470e4	773.69548	

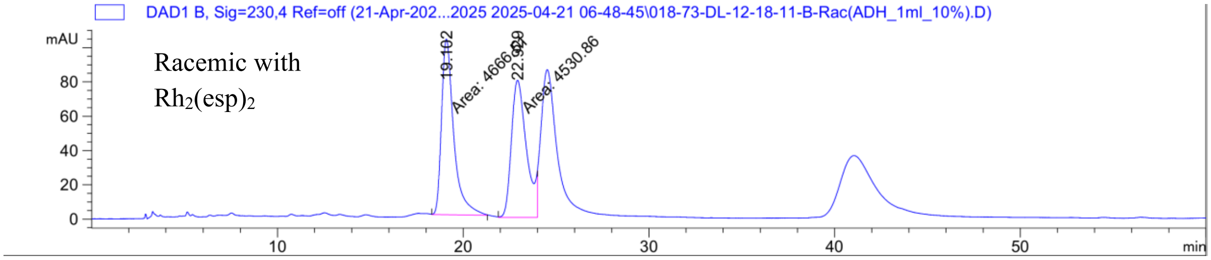

Signal 2: DAD1 B, Sig=230,4 Ref=off

Peak #	RetTime [min]	Type	Width [min]	Area [mAU*s]	Height [mAU]	Area %
1	19.102	MM	0.7593	4666.50781	102.42846	50.7374
2	22.929	MM	0.9433	4530.86279	80.05630	49.2626
Totals :				9197.37061	182.48476	

20250420-DL-12-18-11-A-Clean.10.fid

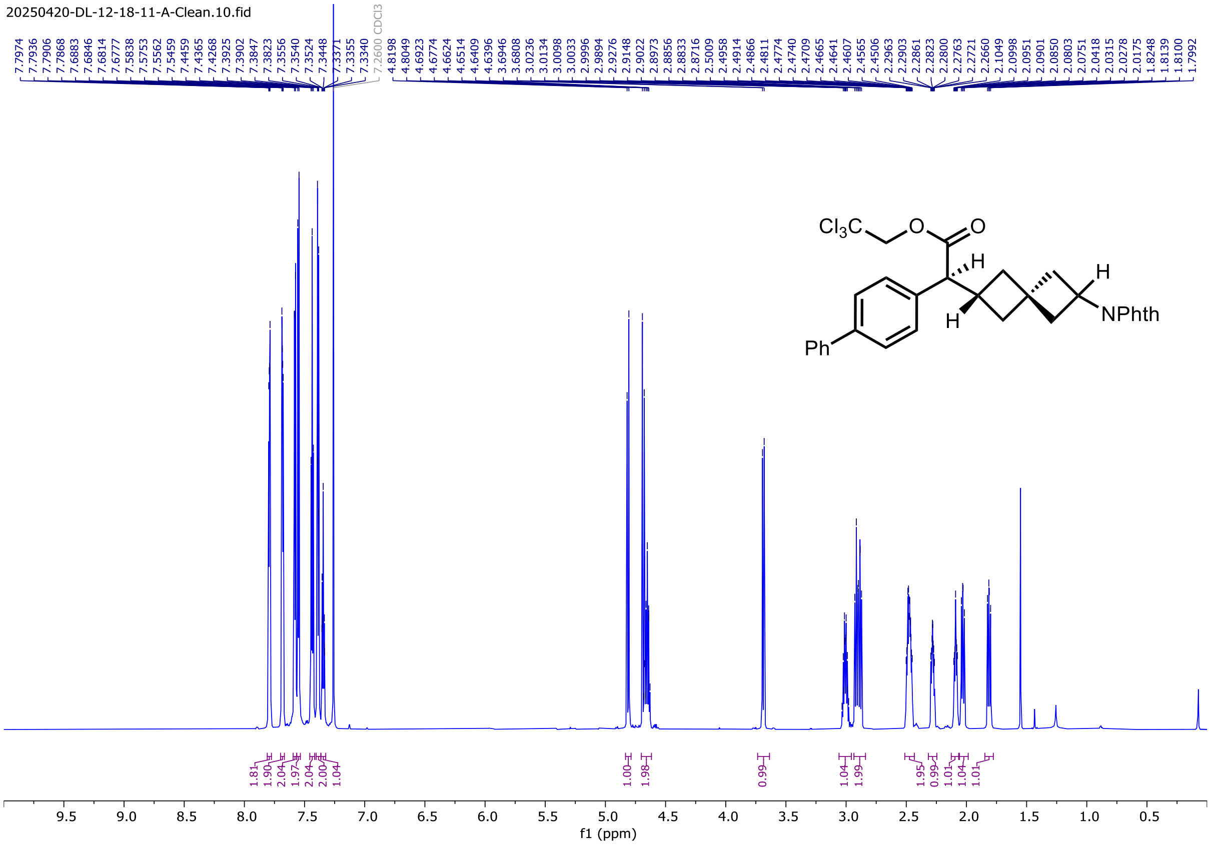

20250420-DL-12-18-11-A-Clean.11.fid

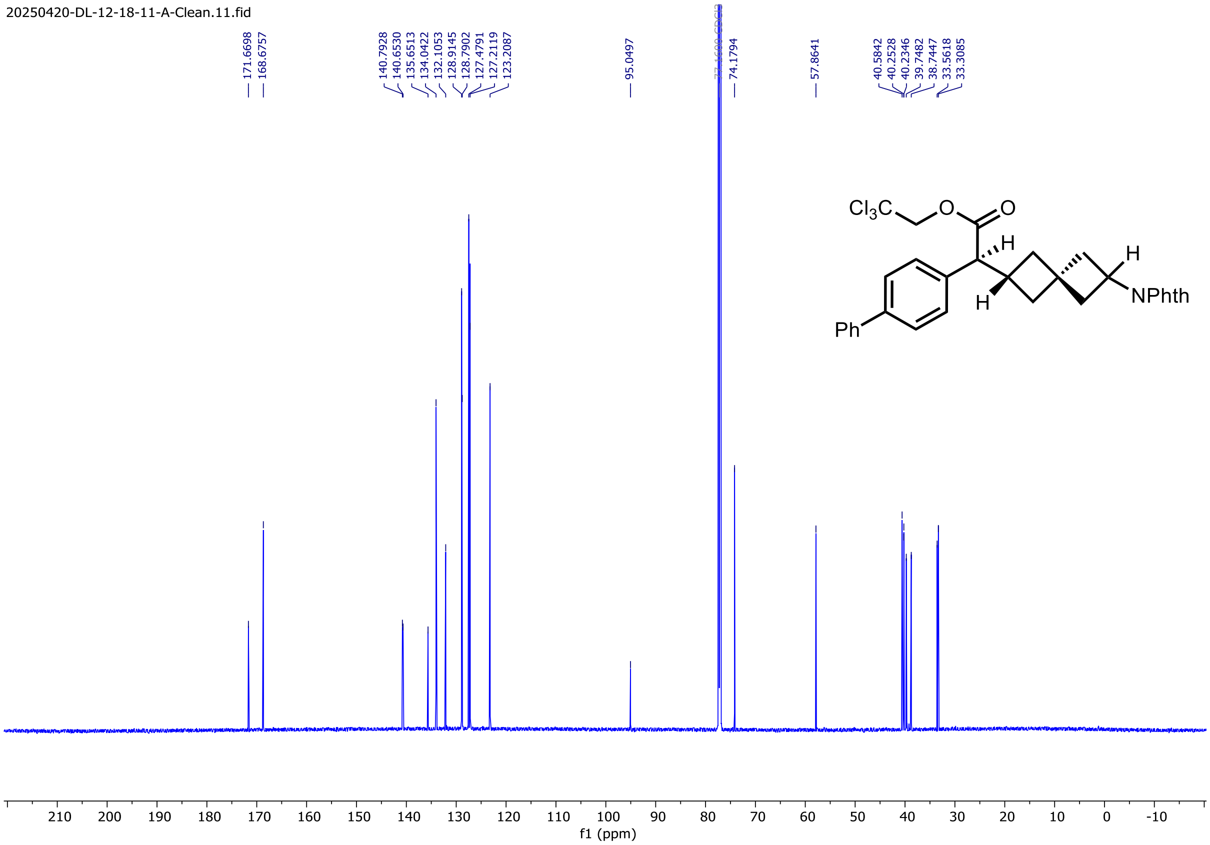

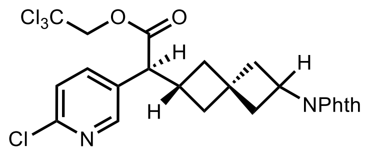

2,2,2-trichloroethyl (2S)-2-(6-chloropyridin-3-yl)-2-(6-(1,3-dioxoisoindolin-2-yl)spiro[3.3]heptan-2-yl)acetate (Compound 30)

Prepared according to general procedure for C-H functionalization, 2-(spiro[3.3]heptan-2-yl)isoindoline-1,3-dione (72.4 mg, 0.3 mmol, 1.0 equiv), Rh_2(*S*-MegaBNP)$_4$ (3.4 mg, 0.0001 mmol, 0.005 equiv), molecular sieve 4Å (100 wt%) and 2,2,2- HFIP (5 μL, 8.40 mg, 0.05 mmol, 0.25 equiv) in 0.5 ml CH_2Cl_2 were added a solution of 2,2,2-trichloroethyl 2-(6-chloropyridin-3-yl)-2-diazoacetate (65.8 mg, 0.2 mmol, 1.0 equiv) in 2.0 ml CH_2Cl_2 at 39℃ in 3 hours. The crude mixture was purified by flash chromatography (SiO_2, gradient 0%-50% Et_2O in hexane) afforded **compound 30** as a clear oil (58.0 mg, 54% yield, 96% ee, 21:1 dr).

R_f (1Hex/3Et_2O) = 0.30 (CAM, UV 254 nm)

$[\alpha]^{20}_D$: 14.3° (c = 1.25 g/100 ml, $CHCl_3$, 96% ee)

^{1}H NMR (600 MHz, $CDCl_3$) δ 8.32 (d, *J* = 2.5 Hz, 1H), 7.79 (dd, *J* = 5.5, 3.0 Hz, 2H), 7.69 (dd, *J* = 5.5, 3.0 Hz, 2H), 7.66 (dd, *J* = 8.3, 2.5 Hz, 1H), 7.31 (d, *J* = 8.3 Hz, 1H), 4.78 (d, *J* = 11.9 Hz, 1H), 4.70 (d, *J* = 11.9 Hz, 1H), 4.64 (p, *J* = 8.8 Hz, 1H), 3.65 (d, *J* = 11.0 Hz, 1H), 2.96 – 2.83 (m, 3H), 2.53 – 2.42 (m, 2H), 2.27 (ddd, *J* = 10.9, 8.3, 4.8 Hz, 1H), 2.08 – 2.00 (m, 2H), 1.74 (dd, *J* = 11.8, 8.8 Hz, 1H).

^{13}C NMR (151 MHz, $CDCl_3$) δ 170.5, 168.6, 151.1, 149.7, 138.5, 134.1, 132.0, 131.3, 124.5, 123.2, 94.7, 74.3, 54.7, 40.4, 40.2, 40.1, 39.6, 38.6, 33.5, 33.3.

HRMS (+p APCI) calcd. for [$C_{24}H_{21}O_4N_2{}^{35}Cl_4$] ($[M+H]^+$) 541.0250 found 541.0264.

HPLC (Chiralpak ADH column, 10% isopropanol in hexane, 1.0 mLmin^{-1}, 1.0 mgmL^{-1}, 90 min, UV 230 nm) retention times of 33.0 min (major) and 51.6 min (minor), 96% ee

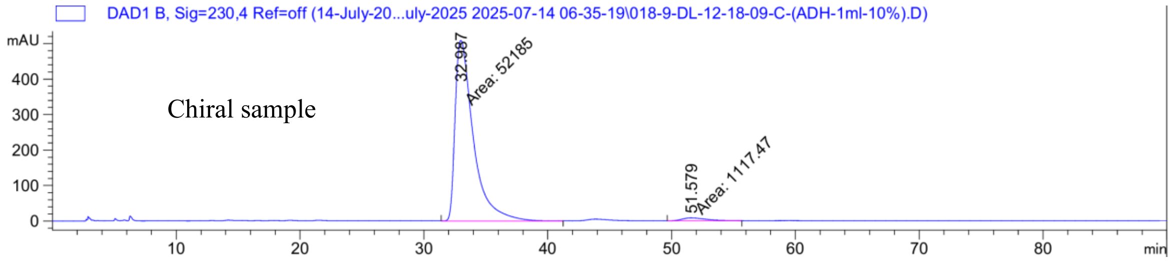

Signal 2: DAD1 B, Sig=230,4 Ref=off

Peak #	RetTime [min]	Type	Width [min]	Area [mAU*s]	Height [mAU]	Area %
1	32.987	MM	1.7097	5.21850e4	508.70755	97.9035
2	51.579	MM	2.3009	1117.46899	8.09459	2.0965
Totals :				5.33025e4	516.80214	

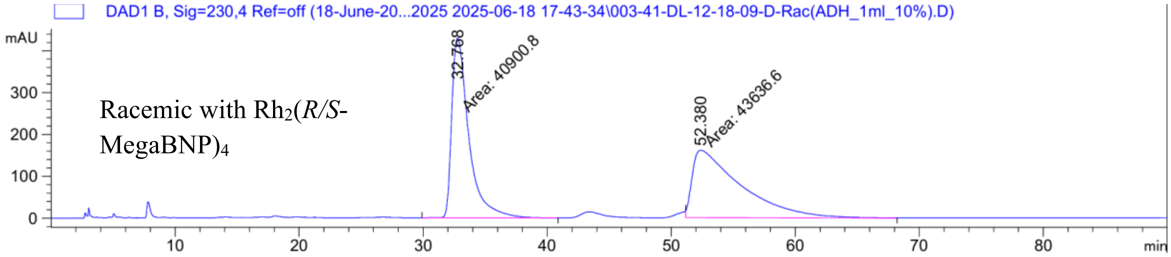

Signal 2: DAD1 B, Sig=230,4 Ref=off

Peak #	RetTime [min]	Type	Width [min]	Area [mAU*s]	Height [mAU]	Area %
1	32.768	MM	1.5892	4.09008e4	428.94687	48.3819
2	52.380	FM	4.5166	4.36366e4	161.02191	51.6181
Totals :				8.45373e4	589.96878	

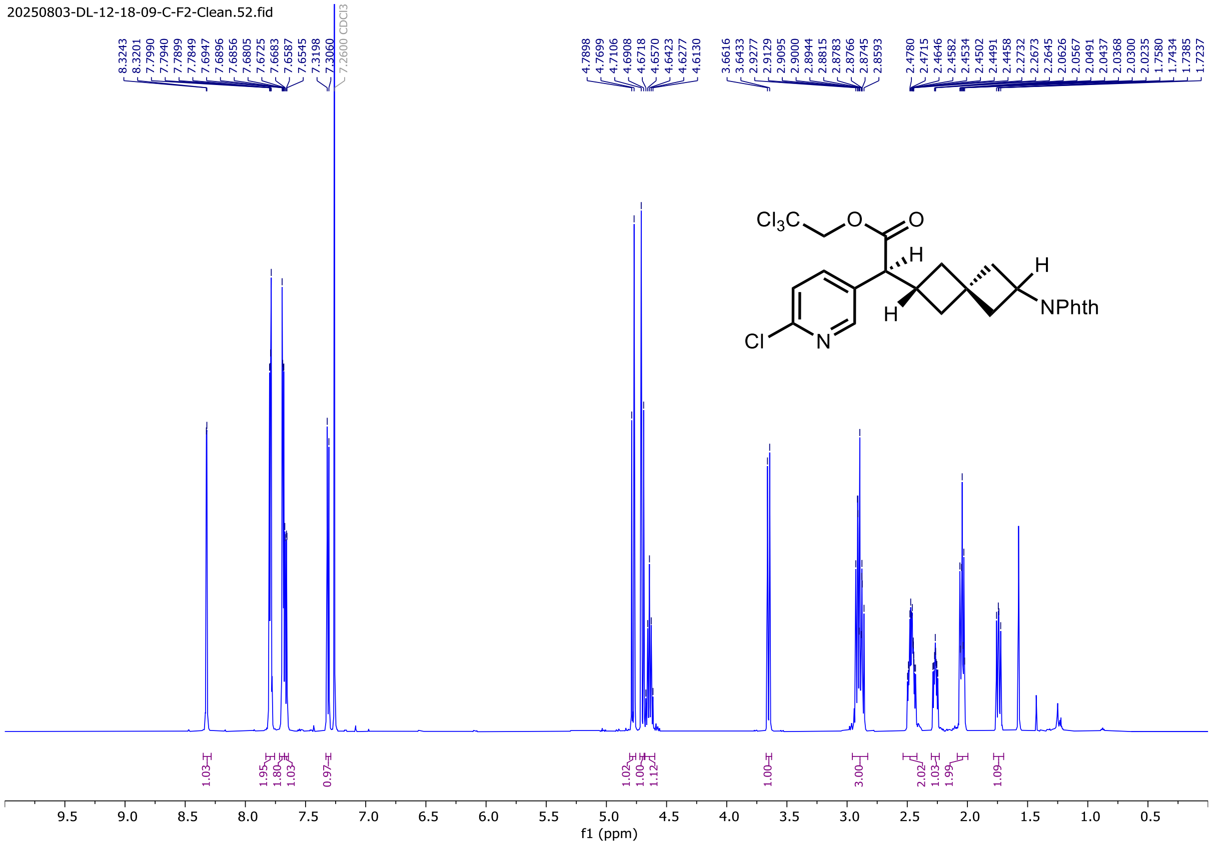
20250803-DL-12-18-09-C-F2-Clean.52.fid
Cl3C
O
O
H
H
H
NPhth
Cl
N
f1 (ppm)

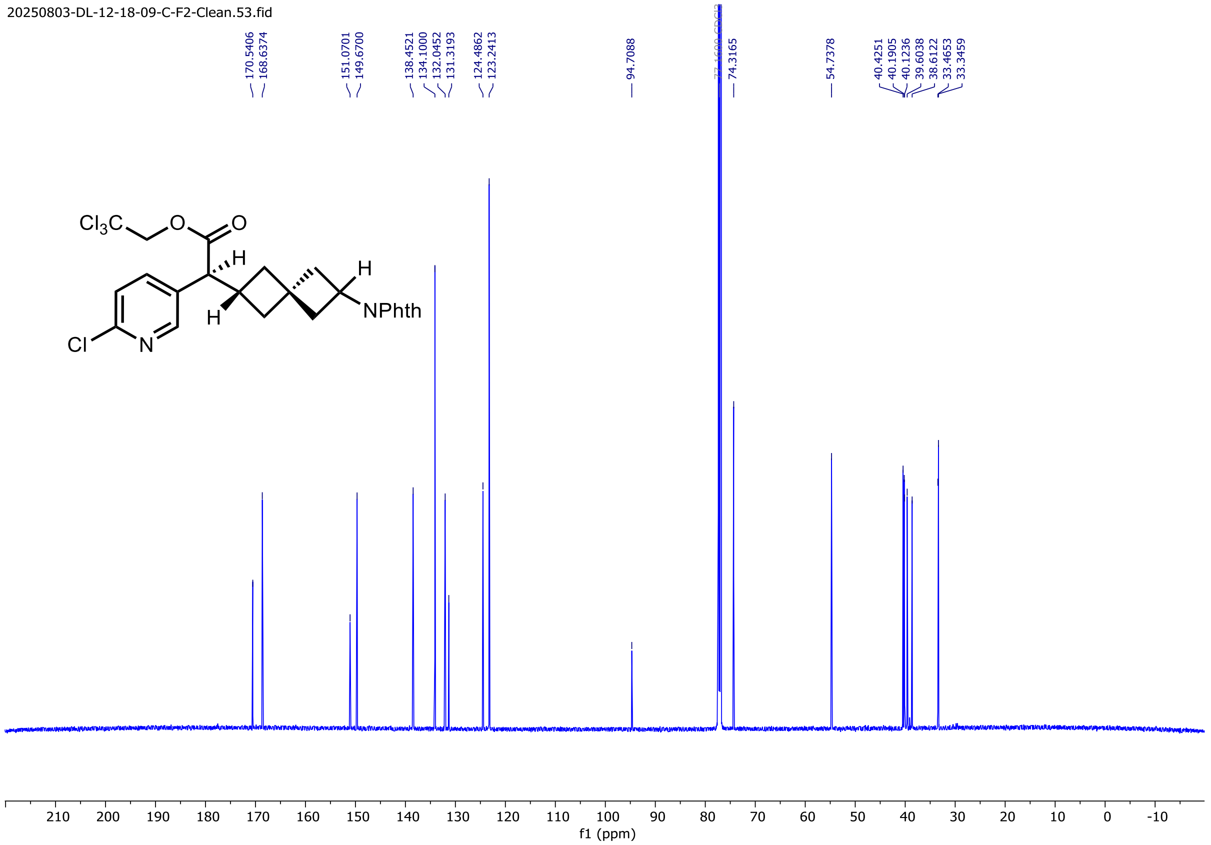
20250803-DL-12-18-09-C-F2-Clean.53.fid
Cl3C
O
O
H
H
H
NPhth
Cl
N
f1 (ppm)

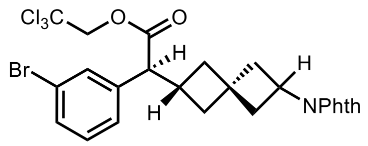

2,2,2-trichloroethyl (2S)-2-(3-bromophenyl)-2-(6-(1,3-dioxoisoindolin-2-yl)spiro[3.3]heptan-2-yl)acetate (Compound 31)

Prepared according to general procedure for C-H functionalization, 2-(spiro[3.3]heptan-2-yl)isoindoline-1,3-dione (48.3 mg, 0.2 mmol, 1.0 equiv), Rh_2(*S*-MegaBNP)$_4$ (3.4 mg, 0.0001 mmol, 0.005 equiv), molecular sieve 4Å (100 wt%) and 2,2,2- HFIP (5 µL, 8.40 mg, 0.05 mmol, 0.25 equiv) in 0.5 ml CH_2Cl_2 were added a solution of trichloroethyl 2-(3-bromophenyl)-2-diazoacetate (149.0 mg, 0.4 mmol, 2.0 equiv) in 2.0 ml CH_2Cl_2 at 39℃ in 3 hours. The crude mixture was purified by flash chromatography (SiO_2, gradient 0%-25% Et_2O in hexane) afforded **compound 31** as a white amorphous solid (78.7 mg, 67% yield, 88% ee, 5:1 dr).

R_f (1Hex/2Et_2O) = 0.50 (CAM, UV 254 nm)

$[\alpha]^{20}_D$: 15.3° (c = 1.27 g/100 ml, $CHCl_3$, 88% ee)

^{1}H NMR (600 MHz, $CDCl_3$) δ 7.79 (dd, *J* = 5.5, 3.0 Hz, 2H), 7.68 (dd, *J* = 5.5, 3.0 Hz, 2H), 7.48 (t, *J* = 1.9 Hz, 1H), 7.41 (d, *J* = 7.8 Hz, 1H), 7.24 (d, *J* = 7.8 Hz, 1H), 7.19 (t, *J* = 7.8 Hz, 1H), 4.78 (d, *J* = 11.9 Hz, 1H), 4.70 – 4.56 (m, 2H), 3.59 (d, *J* = 11.1 Hz, 1H), 2.99 – 2.82 (m, 3H), 2.51 – 2.34 (m, 2H), 2.30 – 2.18 (m, 1H), 2.15 – 2.03 (m, 1H), 1.99 (dd, *J* = 11.3, 8.2 Hz, 1H), 1.76 (dd, *J* = 11.9, 8.6 Hz, 1H). (Note: analyzed as a mixture of 2 diastereomers)

^{13}C NMR (151 MHz, $CDCl_3$) δ 171.0, 168.7, 138.8, 134.1, 132.1, 131.4, 130.9, 130.9, 130.3, 127.1, 123.2, 122.8, 94.9, 74.2, 57.8, 40.5, 40.2, 39.7, 38.6, 33.5, 33.2. (only the major diastereomer is reported)

HRMS (+p APCI) calcd. for $[C_{25}H_{22}O_4N^{79}Br^{35}Cl_3]$ ($[M+H]^+$) 583.9792 found 583.9806.

HPLC (OD column, 5% isopropanol in hexane, 1.0 mLmin^{-1}, 1.0 mgmL^{-1}, 60 min, UV 230 nm) retention times of 18.4 min (major) and 22.5 min (minor), 88% ee.

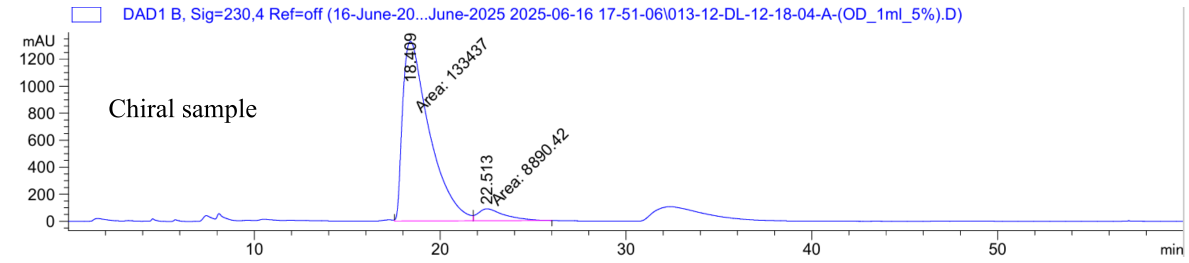

Signal 2: DAD1 B, Sig=230,4 Ref=off

Peak #	RetTime [min]	Type	Width [min]	Area [mAU*s]	Height [mAU]	Area %
1	18.409	MF	1.6712	1.33437e5	1330.74133	93.7535
2	22.513	FM	1.6743	8890.41895	88.49674	6.2465

Totals : 1.42327e5 1419.23808

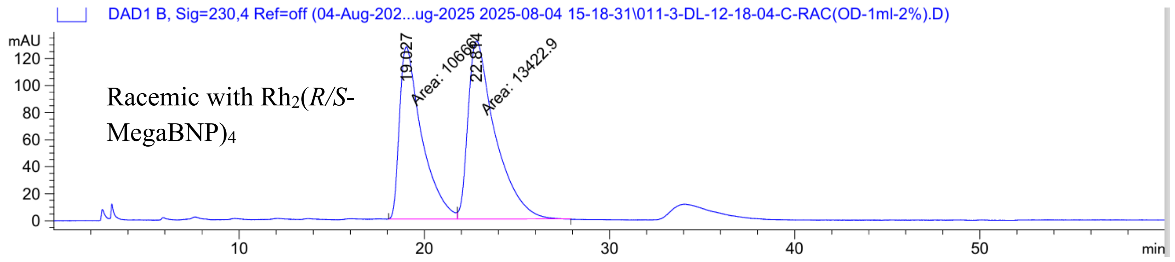

Signal 2: DAD1 B, Sig=230,4 Ref=off

Peak #	RetTime [min]	Type	Width [min]	Area [mAU*s]	Height [mAU]	Area %
1	19.027	MF	1.3946	1.06660e4	127.46774	44.2775
2	22.814	FM	1.6996	1.34229e4	131.63161	55.7225

Totals : 2.40889e4 259.09935

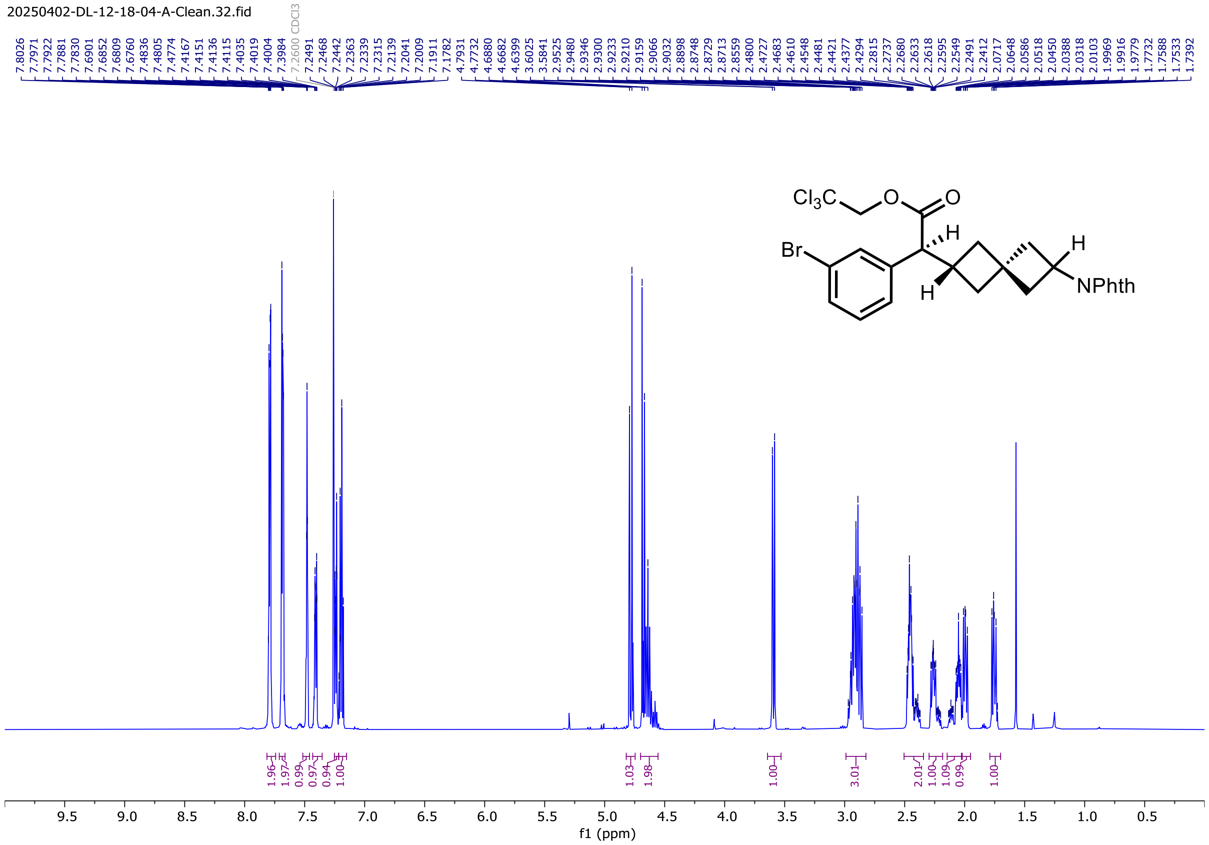
20250402-DL-12-18-04-A-Clean.32.fid
Cl3C
O
O
H
Br
H
H
NPhth
f1 (ppm)

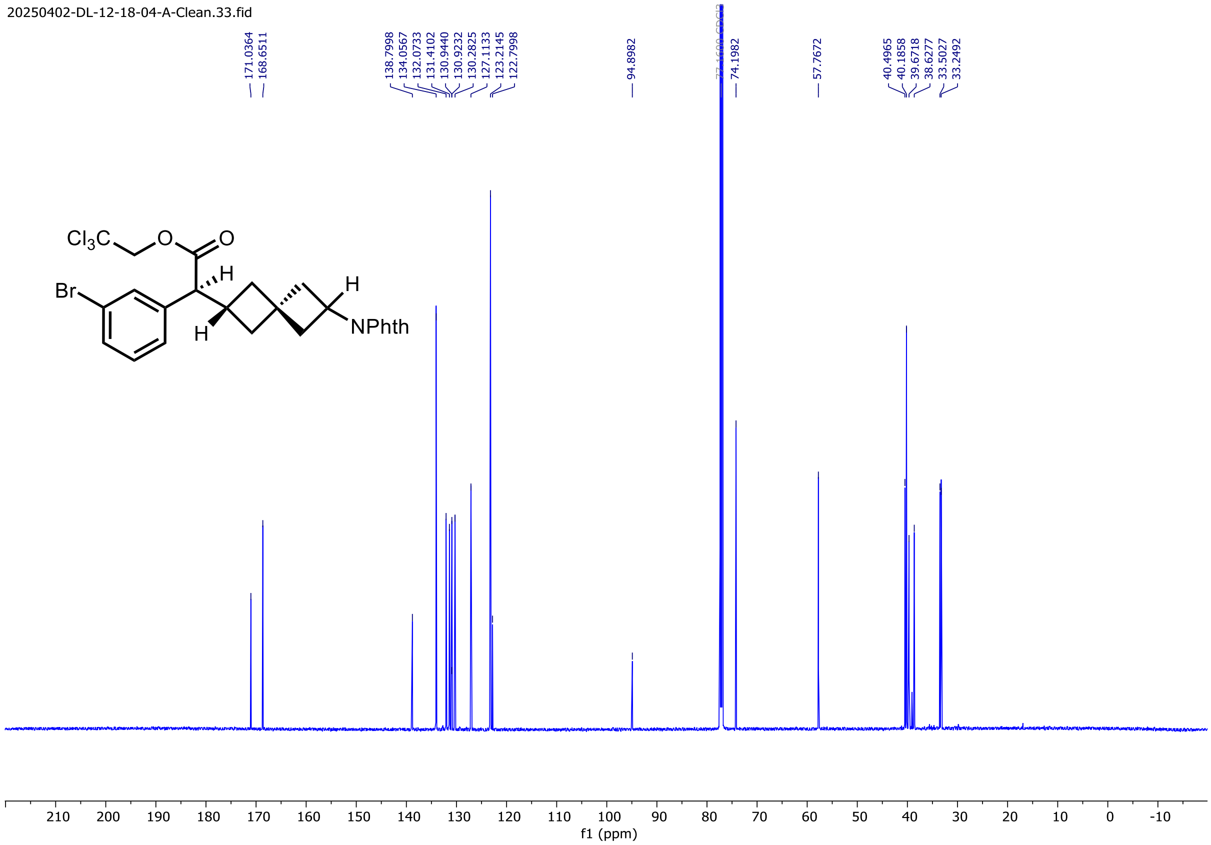
20250402-DL-12-18-04-A-Clean.33.fid
Cl3C
O
O
H
Br
H
H
NPhth
f1 (ppm)

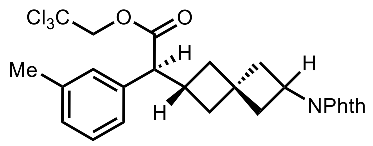

2,2,2-trichloroethyl (2S)-2-(6-(1,3-dioxoisoindolin-2-yl)spiro[3.3]heptan-2-yl)-2-(m-tolyl)acetate (Compound 32)

Prepared according to general procedure for C-H functionalization, 2-(spiro[3.3]heptan-2-yl)isoindoline-1,3-dione (48.3 mg, 0.2 mmol, 1.0 equiv), Rh_2(*S*-MegaBNP)$_4$ (3.4 mg, 0.0001 mmol, 0.005 equiv), molecular sieve 4Å (100 wt%) and 2,2,2- HFIP (5 µL, 8.40 mg, 0.05 mmol, 0.25 equiv) in 0.5 ml CH_2Cl_2 were added a solution of trichloroethyl 2-(3-methylphenyl)-2-diazoacetate (123.0 mg, 0.4 mmol, 2.0 equiv) in 2.0 ml CH_2Cl_2 at 39℃ in 3 hours. The crude mixture was purified by flash chromatography (SiO_2, gradient 0%-25% Et_2O in hexane) afforded **compound 32** as a white amorphous solid (37.0 mg, 36% yield, 97% ee, 7:1 dr).

R_f (1Hex/2Et_2O) = 0.50 (CAM, UV 254 nm)

$[\alpha]^{20}_D$: 22.3° (c = 0.48 g/100 ml, $CHCl_3$, 97% ee)

^{1}H NMR (600 MHz, $CDCl_3$) δ 7.79 (dd, *J* = 5.5, 3.0 Hz, 2H), 7.68 (dd, *J* = 5.5, 3.0 Hz, 2H), 7.20 (t, *J* = 7.5 Hz, 1H), 7.16 – 7.05 (m, 3H), 4.80 (d, *J* = 11.9 Hz, 1H), 4.68 – 4.59 (m, 2H), 3.59 (d, *J* = 11.1 Hz, 1H), 3.00 – 2.92 (m, 1H), 2.92 – 2.84 (m, 2H), 2.50 – 2.41 (m, 2H), 2.34 (s, 3H), 2.26 (ddd, *J* = 10.8, 8.1, 4.8 Hz, 1H), 2.04 (ddd, *J* = 12.0, 7.9, 4.1 Hz, 1H), 1.98 (dd, *J* = 11.2, 8.2 Hz, 1H), 1.77 (dd, *J* = 11.9, 8.5 Hz, 1H).

^{13}C NMR (151 MHz, $CDCl_3$) δ 171.8, 168.7, 138.4, 136.5, 134.0, 132.1, 129.1, 128.6, 128.5, 125.4, 123.2, 95.1, 74.1, 58.2, 40.6, 40.2, 40.2, 39.7, 38.7, 33.5, 33.2, 21.6.

HRMS (+p APCI) calcd. for [$C_{26}H_{25}O_4N^{35}Cl_3$] ($[M+H]^+$) 520.0844 found 520.0858.

HPLC (ASH column, 2% isopropanol in hexane, 1.0 mLmin^{-1}, 1.0 mgmL^{-1}, 60 min, UV 230 nm) retention times of 16.0 min (minor) and 21.9 min (major), 97% ee.

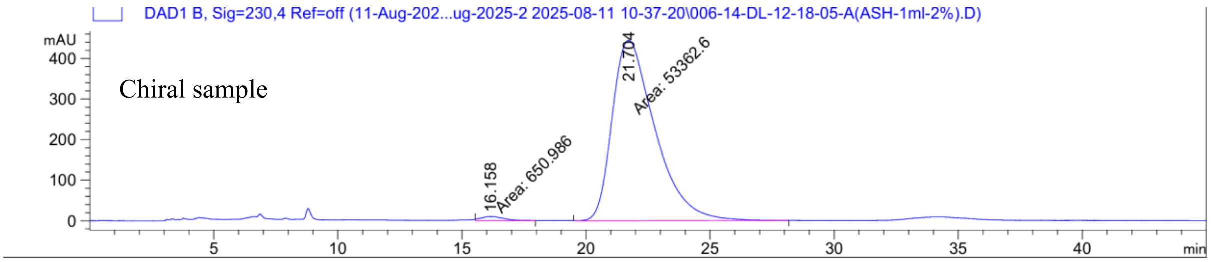

Signal 2: DAD1 B, Sig=230,4 Ref=off

Peak #	RetTime [min]	Type	Width [min]	Area [mAU*s]	Height [mAU]	Area %
1	16.158	MM	1.0804	650.98627	10.04201	1.2052
2	21.704	MM	2.0005	5.33626e4	444.58667	98.7948

Totals : 5.40135e4 454.62868

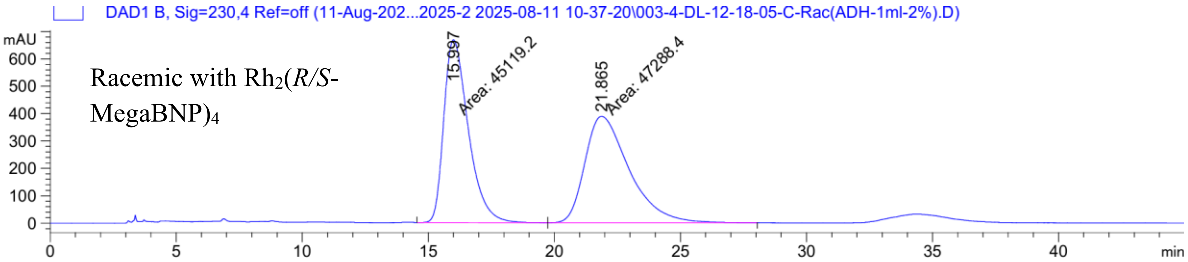

Signal 2: DAD1 B, Sig=230,4 Ref=off

Peak #	RetTime [min]	Type	Width [min]	Area [mAU*s]	Height [mAU]	Area %
1	15.997	MF	1.1264	4.51192e4	667.59064	48.8263
2	21.865	FM	2.0281	4.72884e4	388.60437	51.1737

Totals : 9.24076e4 1056.19501

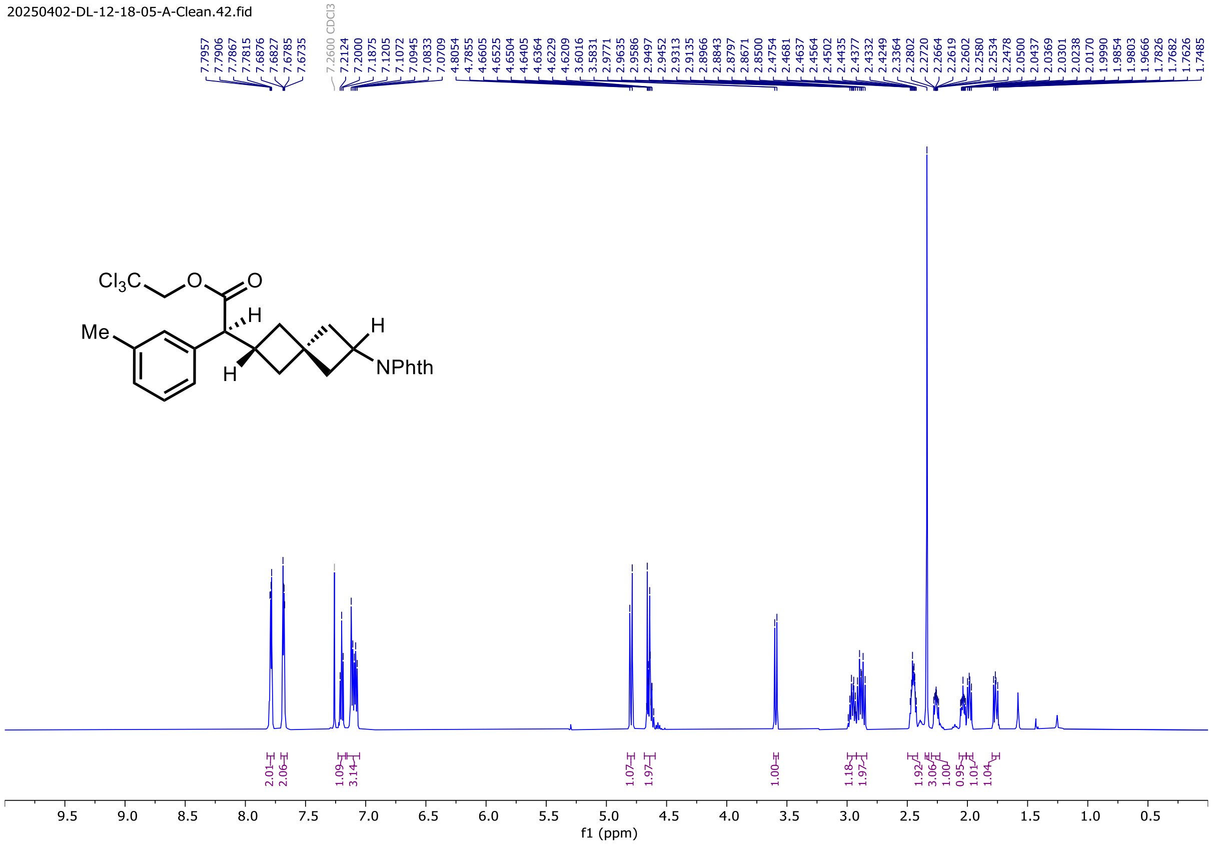
20250402-DL-12-18-05-A-Clean.42.fid
Cl3C
O
O
H
Me
H
H
NPhth
f1 (ppm)

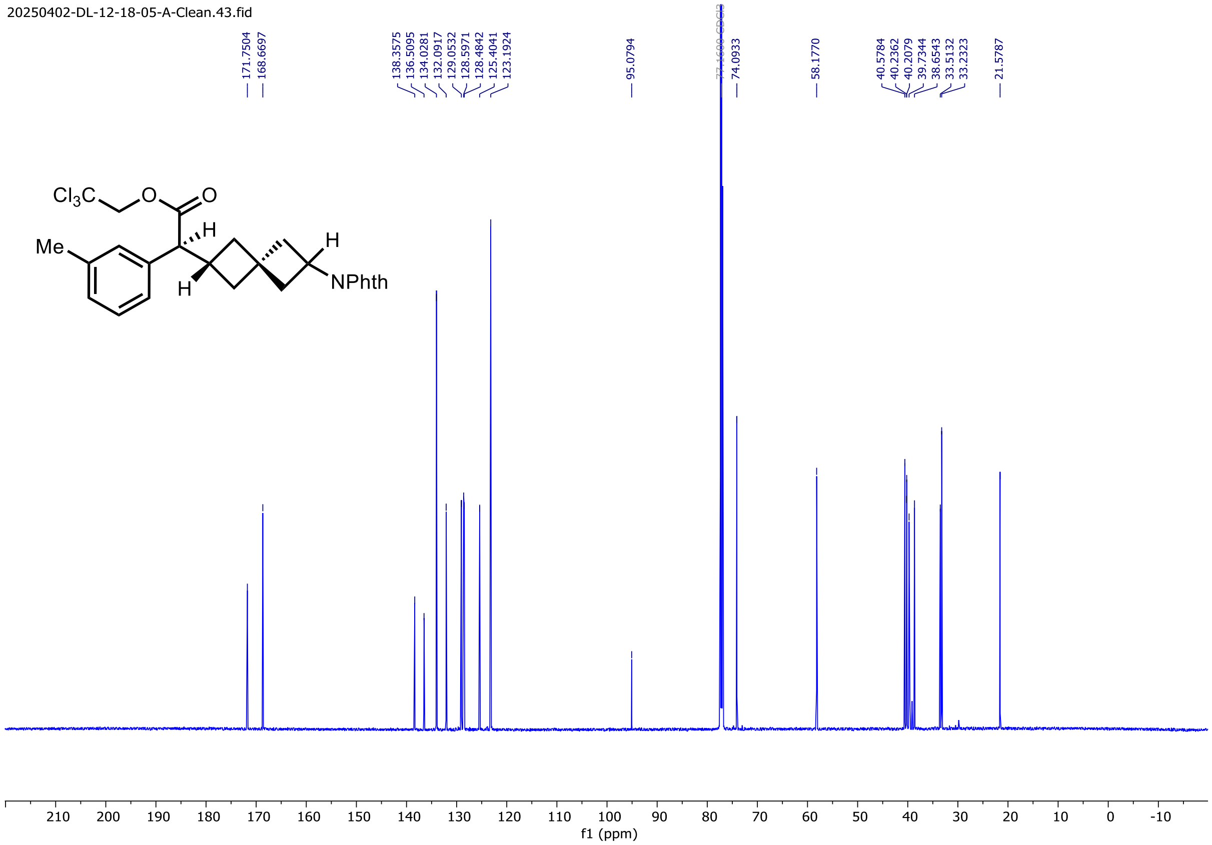
20250402-DL-12-18-05-A-Clean.43.fid
171.7504
168.6697
138.3575
136.5095
134.0281
132.0917
129.0532
128.5971
128.4842
125.4041
123.1924
95.0794
74.0933
58.1770
40.5784
40.2362
40.2079
39.7344
38.6543
33.5132
33.2323
21.5787
Cl3C
O
O
H
Me
H
H
NPhth
f1 (ppm)

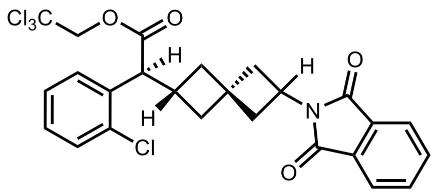

2,2,2-trichloroethyl (2S)-2-(2-chlorophenyl)-2-(6-(1,3-dioxoisoindolin-2-yl)spiro[3.3]heptan-2-yl)acetate (Compound 33)

Prepared according to general procedure for C-H functionalization, 2-(spiro[3.3]heptan-2-yl)isoindoline-1,3-dione (48.3 mg, 0.2 mmol, 1.0 equiv), Rh_2(*S*-MegaBNP)$_4$ (3.4 mg, 0.0001 mmol, 0.005 equiv), molecular sieve 4Å (100 wt%) and 2,2,2- HFIP (5 μL, 8.40 mg, 0.05 mmol, 0.25 equiv) in 0.5 ml CH_2Cl_2 were added a solution of 2,2,2-trichloroethyl 2-(2-chlorophenyl)-2-diazoacetate (131.0 mg, 0.4 mmol, 2.0 equiv) in 2.0 ml CH_2Cl_2 at 39℃ in 3 hours. The crude mixture was purified by flash chromatography (SiO_2, gradient 0%-25% Et_2O in hexane) afforded **compound 33** as a white amorphous solid (51.3 mg, 47% yield, 72% ee, 1:1 dr).

R_f (1Hex/2Et_2O) = 0.50 (CAM, UV 254 nm)

$[\alpha]^{20}_D$: 7.54° (c = 0.83 g/100 ml, $CHCl_3$, 72% ee)

^{1}H NMR (600 MHz, $CDCl_3$) δ 7.83 – 7.76 (m, 2H), 7.72 – 7.65 (m, 2H), 7.43 – 7.34 (m, 2H), 7.26 – 7.17 (m, 2H), 4.79 – 4.73 (m, 1H), 4.72 – 4.54 (m, 2H), 4.31 (dd, *J* = 10.9, 4.2 Hz, 1H), 3.02 – 2.85 (m, 3H), 2.55 – 2.37 (m, 2H), 2.34 – 2.24 (m, 1H), 2.15 – 2.03 (m, 2H), 1.84 – 1.76 (m, 1H). (Note: the spectrum was analyzed as a mixture of 2 diastereomers)

^{13}C NMR (151 MHz, $CDCl_3$) δ 171.12, 171.08, 168.69, 168.67, 134.75, 134.69, 134.61, 134.05, 132.10, 129.83, 129.81, 129.31, 129.30, 128.81, 128.79, 127.22, 127.20, 123.21, 94.94, 74.24, 53.31, 53.25, 40.57, 40.54, 40.16, 40.14, 39.85, 39.72, 38.85, 38.47, 33.68, 33.66, 32.92, 32.89. (Note: the spectrum was analyzed as a mixture of 2 diastereomers)

HRMS (+p APCI) calcd. for [$C_{25}H_{22}O_4N^{35}Cl_4$] ($[M+H]^+$) 540.0298 found 540.0313.

HPLC (Chiralpak ADH column, 3% isopropanol in hexane, 1.0 mLmin^{-1}, 1.0 mgmL^{-1}, 60 min, UV 230 nm) retention times of 17.6 min (major) and 18.3 min (minor), 72% ee for the first diastereomer and retention times of 20.3 min (major) and 21.4 (minor), 83% ee for the second diastereomer.

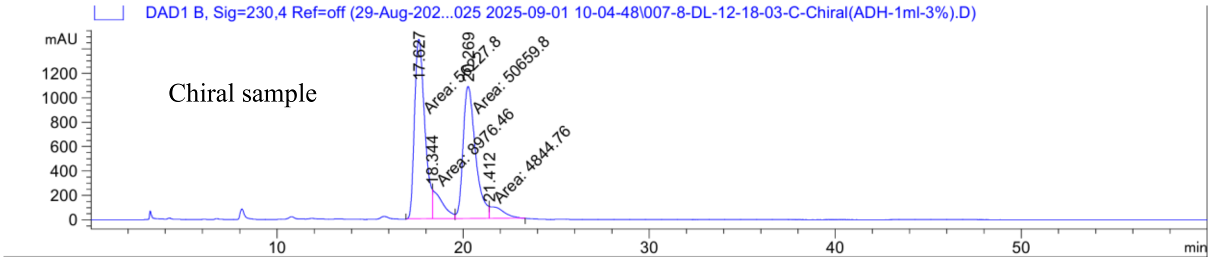

Signal 2: DAD1 B, Sig=230,4 Ref=off

Peak #	RetTime [min]	Type	Width [min]	Area [mAU*s]	Height [mAU]	Area %
1	17.627	MF	0.6374	5.62278e4	1470.31641	46.5813
2	18.344	MF	0.6220	8976.46289	240.53792	7.4365
3	20.269	FM	0.7802	5.06598e4	1082.20557	41.9686
4	21.412	FM	0.8420	4844.75977	95.89917	4.0136
Totals :				1.20709e5	2888.95906	

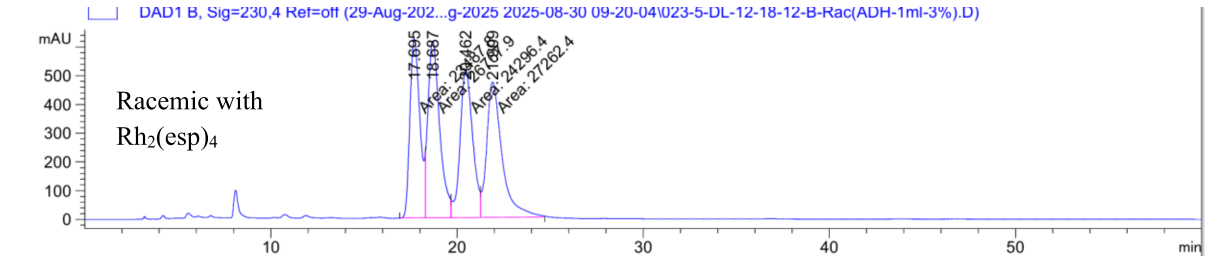

Signal 2: DAD1 B, Sig=230,4 Ref=off

Peak #	RetTime [min]	Type	Width [min]	Area [mAU*s]	Height [mAU]	Area %
1	17.695	MF	0.6285	2.34878e4	622.88928	23.0692
2	18.687	MF	0.7262	2.67679e4	614.32001	26.2908
3	20.462	MF	0.7917	2.42964e4	511.49091	23.8634
4	21.909	FM	0.9649	2.72624e4	470.89456	26.7766
Totals :				1.01815e5	2219.59476	

20250916-DL-12-18-12-D-Clean-2.10.fid

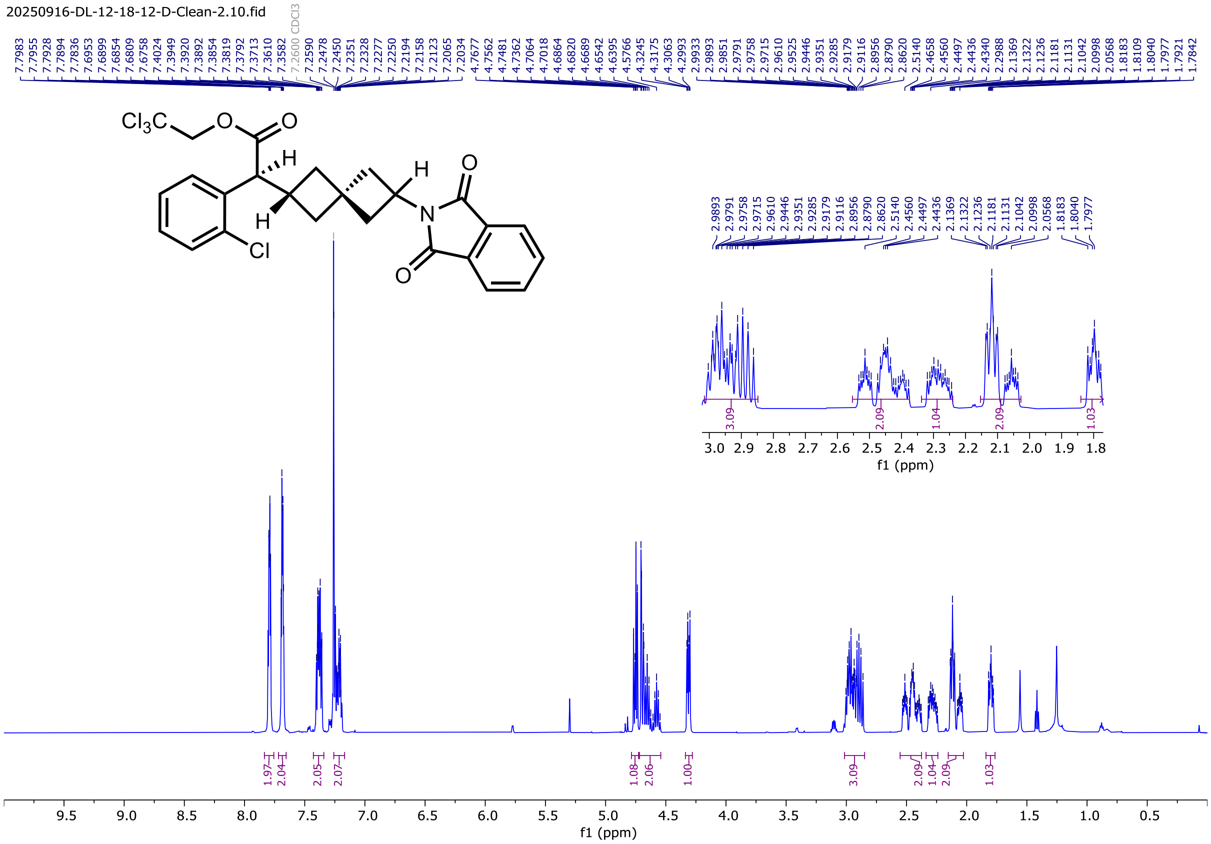

20250916-DL-12-18-12-D-Clean-2.11.fid

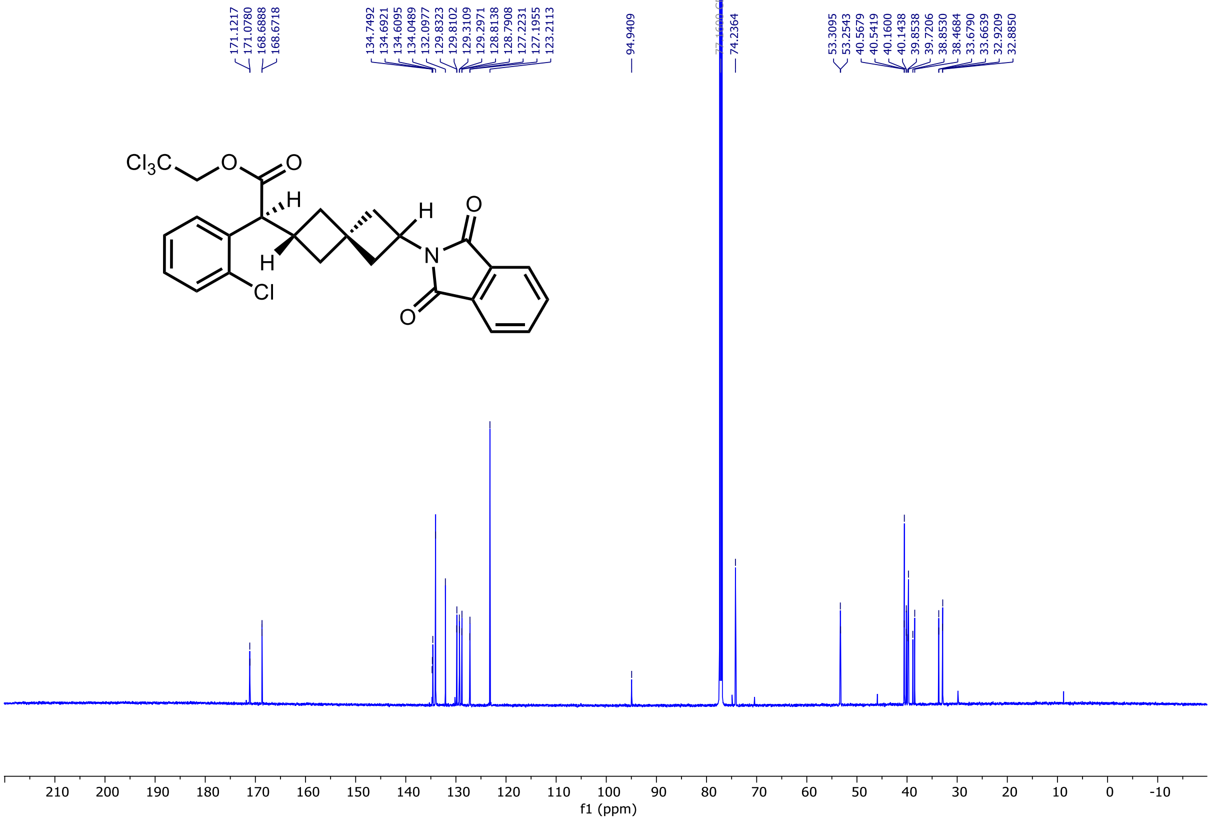

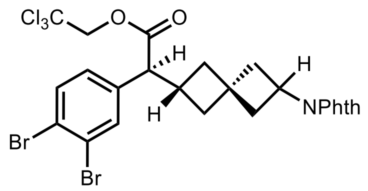

2,2,2-trichloroethyl (2S)-2-(3,4-dibromophenyl)-2-(6-(1,3-dioxoisoindolin-2-yl)spiro[3.3]heptan-2-yl)acetate (Compound 34)

Prepared according to general procedure for C-H functionalization, 2-(spiro[3.3]heptan-2-yl)isoindoline-1,3-dione (48.3 mg, 0.2 mmol, 1.0 equiv), Rh_2(*S*-MegaBNP)$_4$ (3.4 mg, 0.0001 mmol, 0.005 equiv), molecular sieve 4Å (100 wt%) and 2,2,2- HFIP (5 μL, 8.40 mg, 0.05 mmol, 0.25 equiv) in 0.5 ml CH_2Cl_2 were added a solution of 2,2,2-trichloroethyl 2-diazo-2-(3,4-dibromophenyl)acetate (181.0 mg, 0.4 mmol, 2.0 equiv) in 2.0 ml CH_2Cl_2 at 39℃ in 3 hours. The crude mixture was purified by flash chromatography (SiO_2, gradient 0%-25% Et_2O in hexane) afforded **compound 34** as a white amorphous solid (64.0 mg, 48% yield, 98% ee, 21:1 dr).

R_f (1Hex/2Et_2O) = 0.50 (CAM, UV 254 nm)

$[\alpha]^{20}_D$: 18.9° (c = 1.29 g/100 ml, $CHCl_3$, 98% ee)

^{1}H NMR (600 MHz, $CDCl_3$) δ 7.79 (dd, *J* = 5.5, 3.1 Hz, 2H), 7.69 (dd, *J* = 5.5, 3.0 Hz, 2H), 7.60 (d, *J* = 2.1 Hz, 1H), 7.56 (d, *J* = 8.3 Hz, 1H), 7.12 (dd, *J* = 8.3, 2.1 Hz, 1H), 4.78 (d, *J* = 11.9 Hz, 1H), 4.70 – 4.60 (m, 2H), 3.57 (d, *J* = 11.0 Hz, 1H), 2.95 – 2.83 (m, 3H), 2.51 – 2.41 (m, 2H), 2.26 (ddd, *J* = 12.0, 8.2, 4.7 Hz, 1H), 2.06 (ddd, *J* = 12.0, 7.9, 4.2 Hz, 1H), 2.00 (dd, *J* = 11.3, 8.2 Hz, 1H), 1.74 (dd, *J* = 11.8, 8.7 Hz, 1H).

^{13}C NMR (151 MHz, $CDCl_3$) δ 170.7, 168.6, 137.5, 134.1, 133.9, 133.6, 132.1, 128.6, 125.2, 124.1, 123.2, 94.8, 74.3, 57.2, 40.5, 40.2, 40.2, 39.7, 38.6, 33.5, 33.2.

HRMS (+p APCI) calcd. for [$C_{25}H_{21}O_4N^{79}Br_2{}^{35}Cl_3$] ($[M+H]^+$) 661.8897 found 661.8911.

SFC (OZ3, 7% (50% methanol in isopropanol with 0.2% Formic Acid) in CO_2, 2.5 mL/min, 1.0 mg/ml, UV 230 nm) retention times of 10.3 min (major) and 12.8 min (minor), 98% ee.

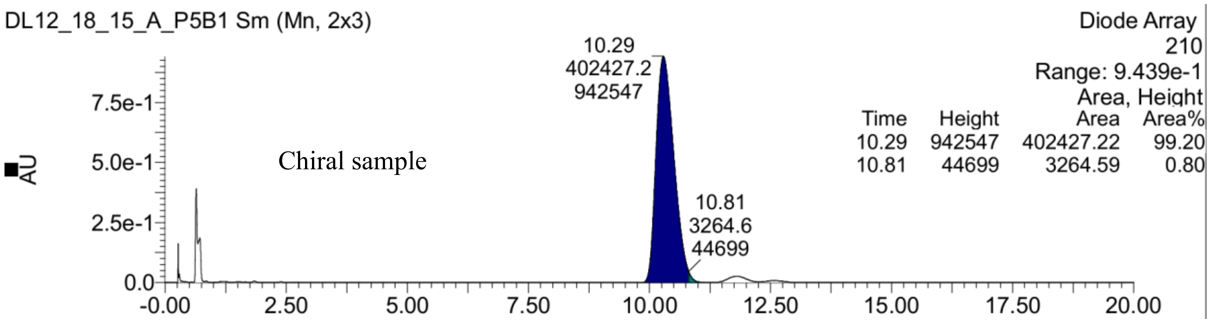
DL12_18_15_A_P5B1 Sm (Mn, 2x3)
Diode Array
210
Range: 9.439e-1
Area, Height
Time Height Area Area%
10.29 942547 402427.22 99.20
10.81 44699 3264.59 0.80
Chiral sample
10.29
402427.2
942547
10.81
3264.6
44699
AU

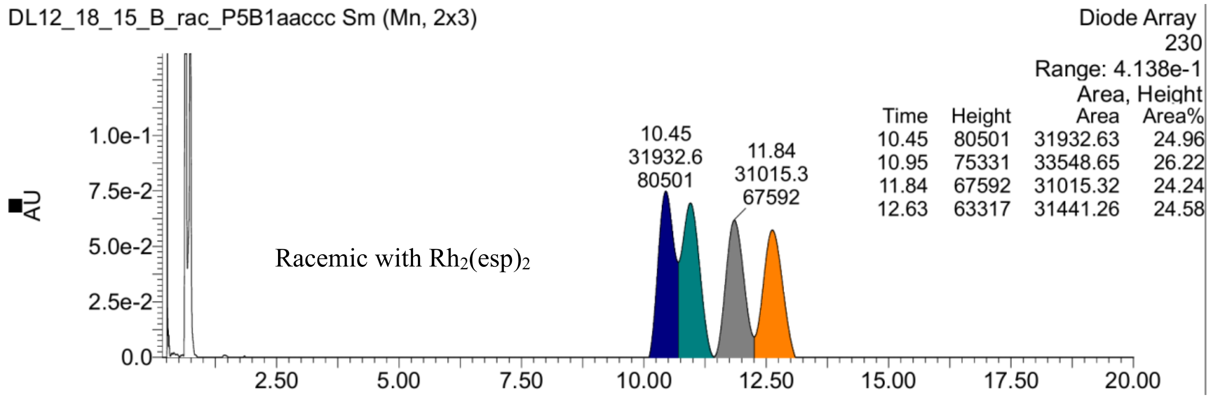
DL12_18_15_B_rac_P5B1aaccc Sm (Mn, 2x3)
Diode Array
230
Range: 4.138e-1
Area, Height
Time Height Area Area%
10.45 80501 31932.63 24.96
10.95 75331 33548.65 26.22
11.84 67592 31015.32 24.24
12.63 63317 31441.26 24.58
Racemic with Rh2(esp)2
10.45
31932.6
80501
11.84
31015.3
67592
AU

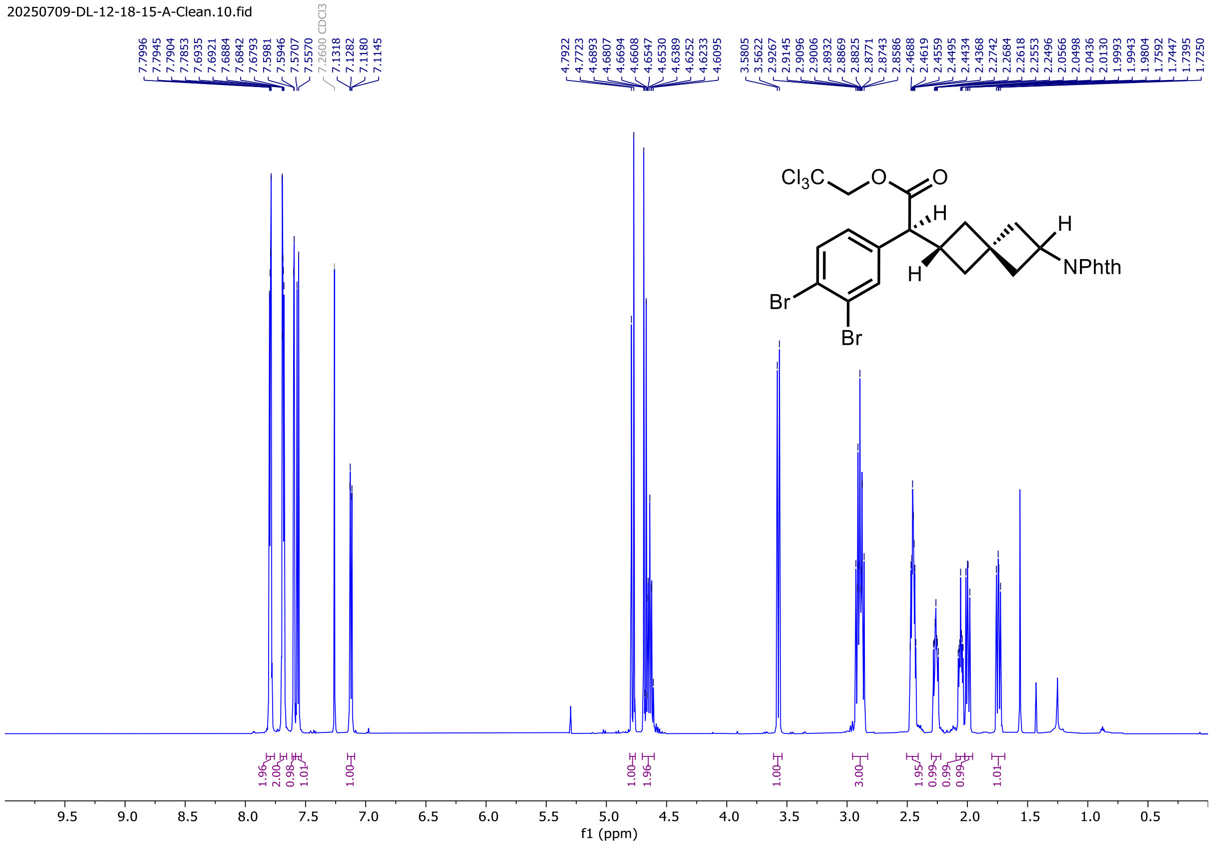
20250709-DL-12-18-15-A-Clean.10.fid
7.7996
7.7945
7.7904
7.7853
7.6935
7.6921
7.6884
7.6842
7.6793
7.5981
7.5946
7.5707
7.5570
7.2600 CDCl3
7.1318
7.1282
7.1180
7.1145
4.7922
4.7723
4.6893
4.6807
4.6694
4.6608
4.6547
4.6530
4.6389
4.6252
4.6233
4.6095
3.5805
3.5622
2.9267
2.9145
2.9096
2.9006
2.8932
2.8869
2.8825
2.8771
2.8743
2.8586
2.4688
2.4619
2.4559
2.4495
2.4434
2.4368
2.2742
2.2684
2.2618
2.2553
2.2496
2.0566
2.0498
2.0436
2.0130
1.9993
1.9943
1.9804
1.7592
1.7447
1.7395
1.7250
Cl3C
O
O
H
H
H
NPhth
Br
Br
1.96
2.00
0.98
1.01
1.00
1.00
1.96
1.00
3.00
1.95
0.99
0.99
0.99
1.01
9.5
9.0
8.5
8.0
7.5
7.0
6.5
6.0
5.5
5.0
4.5
4.0
3.5
3.0
2.5
2.0
1.5
1.0
0.5
f1 (ppm)

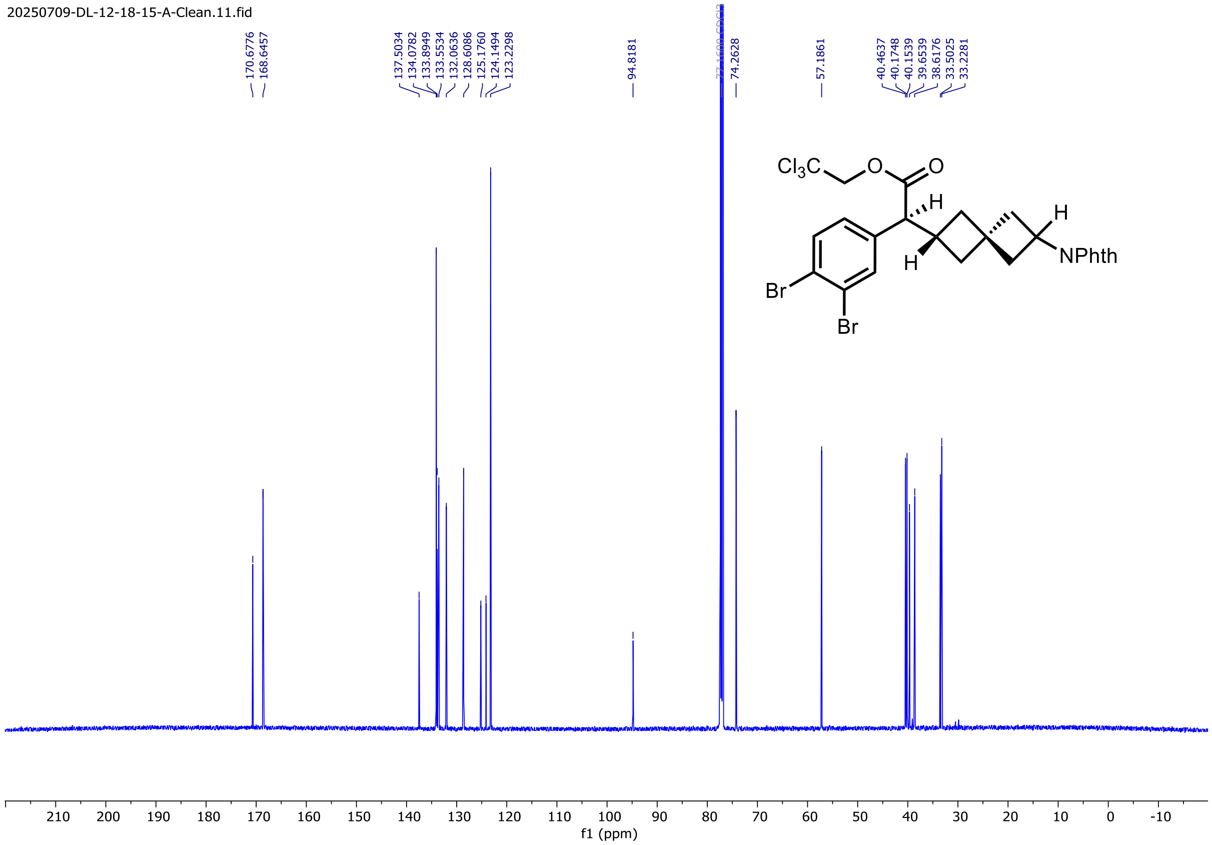
20250709-DL-12-18-15-A-Clean.11.fid
170.6776
168.6457
137.5034
134.0782
133.8949
133.5534
132.0636
128.6086
125.1760
124.1494
123.2298
94.8181
74.2628
57.1861
40.4637
40.1748
40.1539
39.6539
38.6176
33.5025
33.2281
Cl3C
O
O
H
H
H
NPhth
Br
Br
210
200
190
180
170
160
150
140
130
120
110
100
90
80
70
60
50
40
30
20
10
0
-10
f1 (ppm)

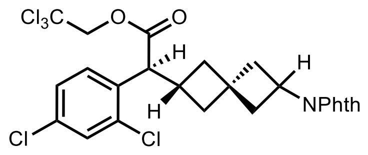

2,2,2-trichloroethyl (2S)-2-(2,4-dichlorophenyl)-2-(6-(1,3-dioxoisoindolin-2-yl)spiro[3.3]heptan-2-yl)acetate (Compound 35)

Prepared according to general procedure for C-H functionalization, 2-(spiro[3.3]heptan-2-yl)isoindoline-1,3-dione (48.3 mg, 0.2 mmol, 1.0 equiv), Rh_2(*S*-MegaBNP)$_4$ (3.4 mg, 0.0001 mmol, 0.005 equiv), molecular sieve 4Å (100 wt%) and 2,2,2- HFIP (5 µL, 8.40 mg, 0.05 mmol, 0.25 equiv) in 0.5 ml CH_2Cl_2 were added a solution of 2,2,2-trichloroethyl 2-diazo-2-(2,4-dichlorophenyl)acetate (145.0 mg, 0.4 mmol, 2.0 equiv) in 2.0 ml CH_2Cl_2 at 39℃ in 3 hours. The crude mixture was purified by flash chromatography (SiO_2, gradient 0%-25% Et_2O in hexane) afforded **compound 35** as a white amorphous solid (66.6 mg, 50% yield, 85% ee, 4:1 dr).

R_f (1Hex/2Et_2O) = 0.50 (CAM, UV 254 nm)

$[\alpha]^{20}_D$: 12.6° (c = 1.14 g/100 ml, $CHCl_3$, 85% ee)

^{1}H NMR (600 MHz, $CDCl_3$) δ 7.79 (dd, *J* = 5.4, 3.0 Hz, 2H), 7.68 (dd, *J* = 5.4, 3.0 Hz, 2H), 7.41 (d, *J* = 2.2 Hz, 1H), 7.31 (d, *J* = 8.3 Hz, 1H), 7.24 (dd, *J* = 8.3, 2.2 Hz, 1H), 4.75 (d, *J* = 11.9 Hz, 1H), 4.72 – 4.55 (m, 2H), 4.25 (dd, *J* = 10.9, 4.1 Hz, 1H), 3.00 – 2.83 (m, 3H), 2.50 (ddd, *J* = 11.6, 7.7, 4.1 Hz, 1H), 2.48 – 2.36 (m, 1H), 2.33 – 2.22 (m, 1H), 2.11 (dd, *J* = 11.4, 8.2 Hz, 1H), 2.06 (ddd, *J* = 12.0, 7.9, 4.2 Hz, 1H), 1.77 (dd, *J* = 11.9, 8.6 Hz, 1H). (analyzed as a mixture of 2 diastereomer)

^{13}C NMR (151 MHz, $CDCl_3$) δ 170.7, 168.6, 135.3, 134.1, 134.0, 133.3, 132.1, 130.2, 129.6, 127.6, 127.6, 123.2, 94.8, 74.3, 52.8, 40.5, 40.1, 39.7, 38.4, 33.7, 32.9. (only major diastereomer is reported due to low resolution)

HRMS (+p APCI) calcd. for $[C_{25}H_{21}O_4N^{35}Cl_5]$ ($[M+H]^+$) 573.9908 found 573.9921.

HPLC (Chiralpak ADH column, 3% isopropanol in hexane, 1.0 mLmin^{-1}, 1.0 mgmL^{-1}, 60 min, UV 230 nm) retention times of 21.9 min (minor) and 23.3 min (major), 85% ee.

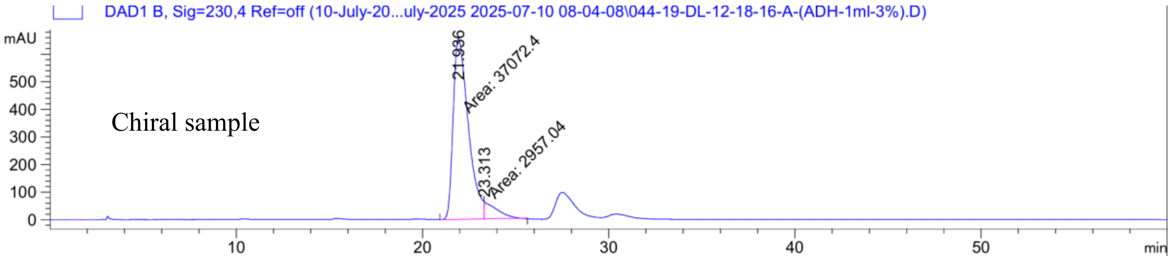

Signal 2: DAD1 B, Sig=230,4 Ref=off

Peak #	RetTime [min]	Type	Width [min]	Area [mAU*s]	Height [mAU]	Area %
1	21.936	MF	0.9434	3.70724e4	654.95178	92.6129
2	23.313	FM	0.7902	2957.03638	62.36946	7.3871
Totals :				4.00295e4	717.32124	

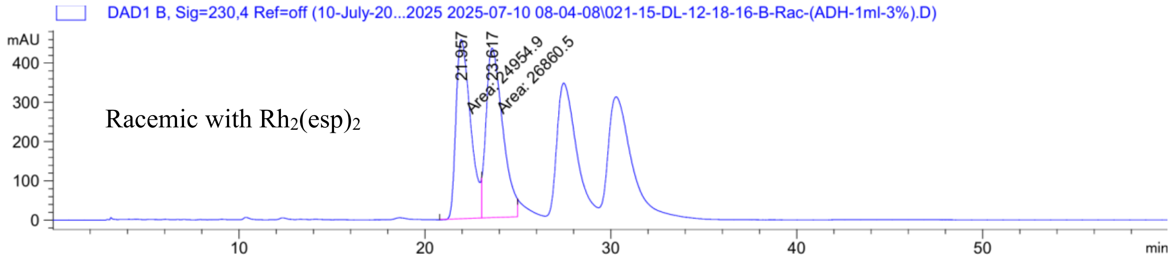

Signal 2: DAD1 B, Sig=230,4 Ref=off

Peak #	RetTime [min]	Type	Width [min]	Area [mAU*s]	Height [mAU]	Area %
1	21.957	MF	0.9112	2.49549e4	456.43918	48.1612
2	23.617	MF	1.0362	2.68605e4	432.02609	51.8388
Totals :				5.18154e4	888.46527	

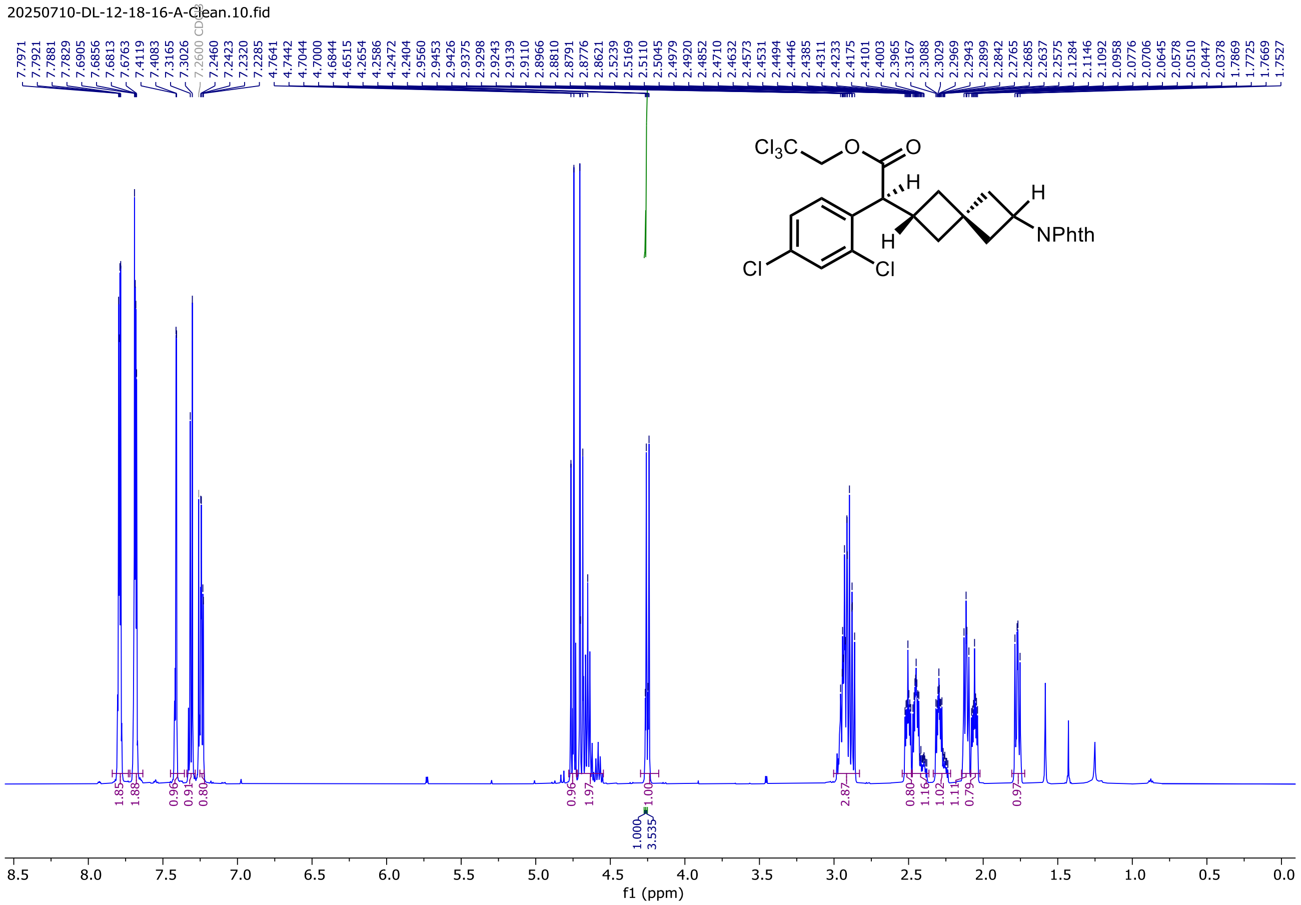

20250710-DL-12-18-16-A-Clean.10.fid
Cl3C
O
O
H
H
H
NPhth
Cl
Cl
f1 (ppm)

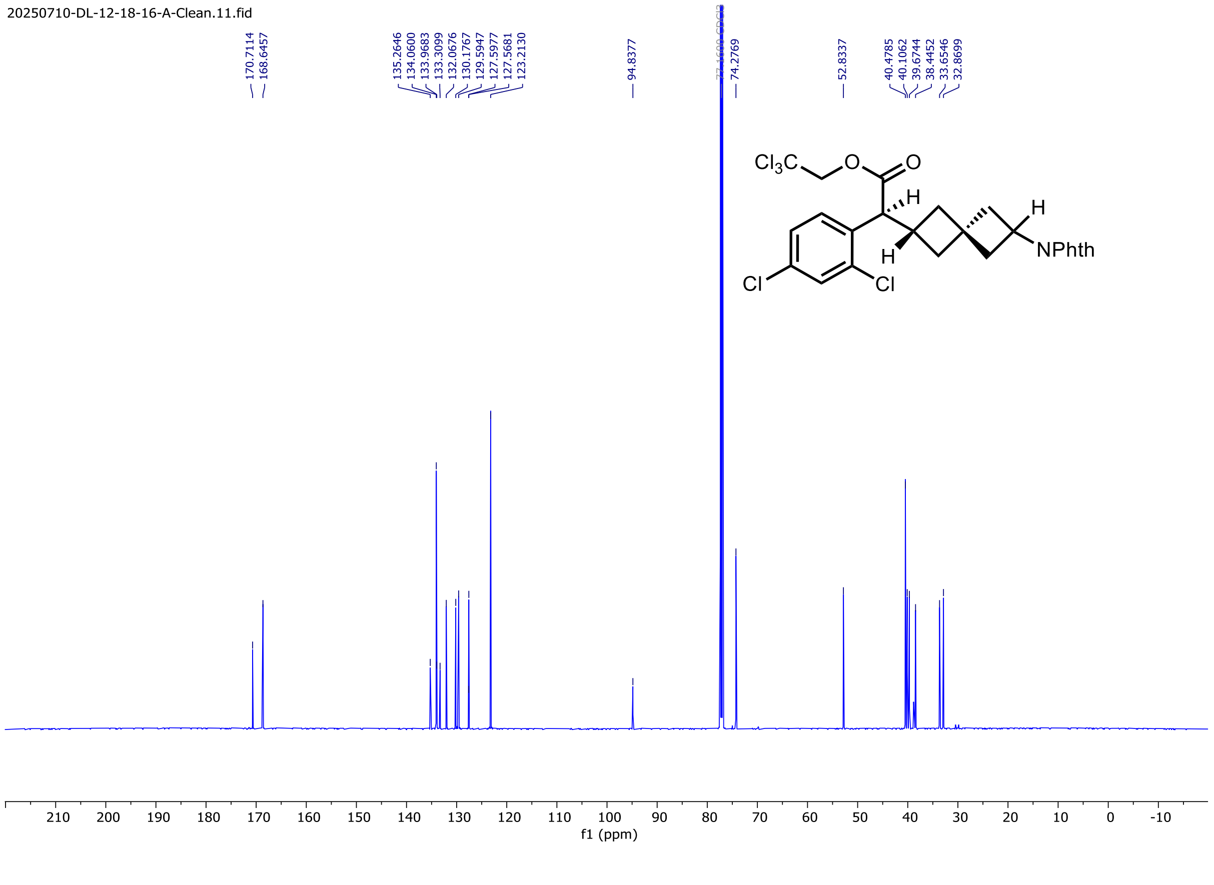

20250710-DL-12-18-16-A-Clean.11.fid
170.7114
168.6457
135.2646
134.0600
133.9683
133.3099
132.0676
130.1767
129.5947
127.5977
127.5681
123.2130
94.8377
74.2769
52.8337
40.4785
40.1062
39.6744
38.4452
33.6546
32.8699
Cl3C
O
O
H
H
H
NPhth
Cl
Cl
f1 (ppm)

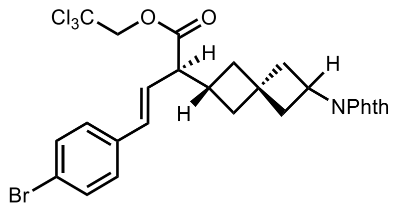

2,2,2-trichloroethyl (2S,E)-4-(4-bromophenyl)-2-(6-(1,3-dioxoisoindolin-2-yl)spiro[3.3]heptan-2-yl)but-3-enoate (Compound 36)

Prepared according to general procedure for C-H functionalization, 2-(spiro[3.3]heptan-2-yl)isoindoline-1,3-dione (48.3 mg, 0.2 mmol, 1.0 equiv), Rh_2(*S*-MegaBNP)$_4$ (3.4 mg, 0.0001 mmol, 0.005 equiv), molecular sieve 4Å (100 wt%) and 2,2,2- HFIP (5 µL, 8.40 mg, 0.05 mmol, 0.25 equiv) in 0.5 ml CH_2Cl_2 were added a solution of 2,2,2-trichloroethyl (E)-4-(4-bromophenyl)-2-diazobut-3-enoate (159.0 mg, 0.4 mmol, 2.0 equiv) in 2.0 ml CH_2Cl_2 at 39℃ in 3 hours. The crude mixture was purified by flash chromatography (SiO_2, gradient 0%-25% Et_2O in hexane) afforded **compound 36** as a white amorphous solid (75.5 mg, 62% yield, 97% ee, 20:1 dr).

R_f (1Hex/2Et_2O) = 0.50 (CAM, UV 254 nm)

$[\alpha]^{20}_D$: 45.1° (c = 0.47 g/100 ml, $CHCl_3$, 97% ee)

^{1}H NMR (600 MHz, $CDCl_3$) δ 7.82 – 7.78 (m, 2H), 7.70 – 7.67 (m, 2H), 7.44 (d, *J* = 8.4 Hz, 2H), 7.23 (d, *J* = 8.4 Hz, 2H), 6.45 (d, *J* = 15.8 Hz, 1H), 6.14 (dd, *J* = 15.8, 9.0 Hz, 1H), 4.80 (d, *J* = 11.9 Hz, 1H), 4.74 (d, *J* = 11.9 Hz, 1H), 4.63 (p, *J* = 8.8 Hz, 1H), 3.23 (t, *J* = 9.4 Hz, 1H), 2.93 (t, *J* = 10.1 Hz, 1H), 2.89 (t, *J* = 10.3 Hz, 1H), 2.68 (h, *J* = 8.5 Hz, 1H), 2.44 (ddd, *J* = 12.0, 8.5, 4.7 Hz, 1H), 2.36 (ddd, *J* = 11.6, 7.8, 4.0 Hz, 1H), 2.26 (ddd, *J* = 13.0, 8.7, 5.1 Hz, 1H), 2.22 (td, *J* = 7.8, 3.8 Hz, 1H), 2.04 – 1.98 (m, 1H), 1.91 (dd, *J* = 11.8, 8.6 Hz, 1H).

^{13}C NMR (151 MHz, $CDCl_3$) δ 171.4, 168.7, 135.6, 134.1, 132.4, 132.1, 131.9, 128.1, 125.5, 123.2, 121.7, 95.0, 74.1, 55.5, 40.5, 40.2, 39.7, 39.4, 38.6, 33.6, 32.6.

HRMS (+p APCI) calcd. for $[C_{27}H_{24}O_4N^{79}Br^{35}Cl_3]$ ($[M+H]^+$) 609.9949 found 609.9964.

HPLC (Chiralpak ADH column, 5% isopropanol in hexane, 1.0 $mLmin^{-1}$, 1.0 $mgmL^{-1}$, 60 min, UV 230 nm) retention times of 29.9 min (major) and 35.2 min (minor), 97% ee.

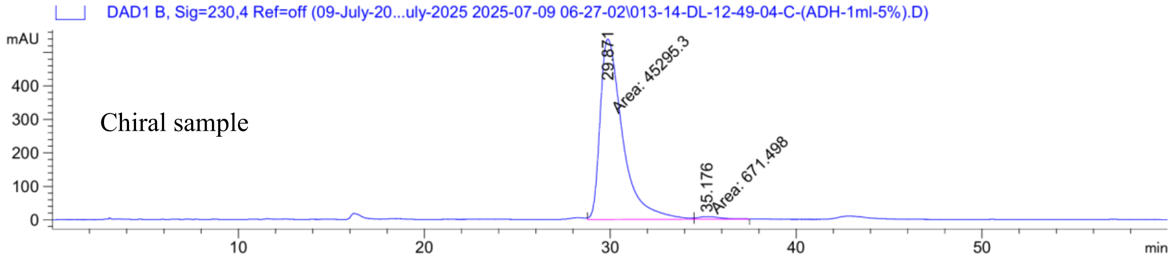

Signal 2: DAD1 B, Sig=230,4 Ref=off

Peak #	RetTime [min]	Type	Width [min]	Area [mAU*s]	Height [mAU]	Area %
1	29.871	FM	1.4021	4.52953e4	538.43164	98.5392
2	35.176	FM	1.4878	671.49756	7.52238	1.4608

Totals : 4.59668e4 545.95402

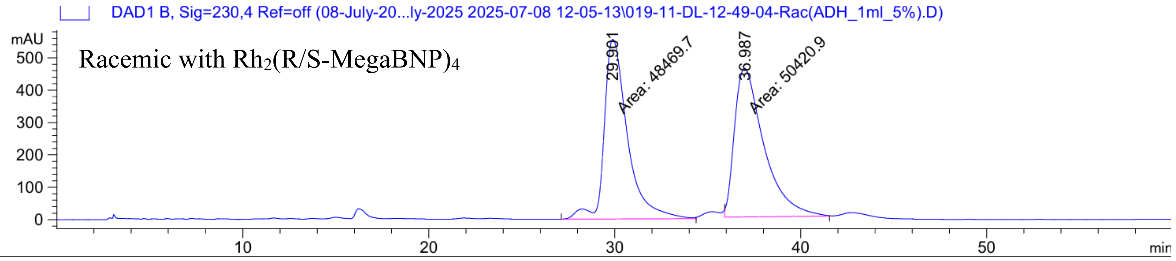

Signal 2: DAD1 B, Sig=230,4 Ref=off

Peak #	RetTime [min]	Type	Width [min]	Area [mAU*s]	Height [mAU]	Area %
1	29.901	MM	1.4569	4.84697e4	554.47235	49.0134
2	36.987	MM	1.8296	5.04209e4	459.31573	50.9866

Totals : 9.88906e4 1013.78809

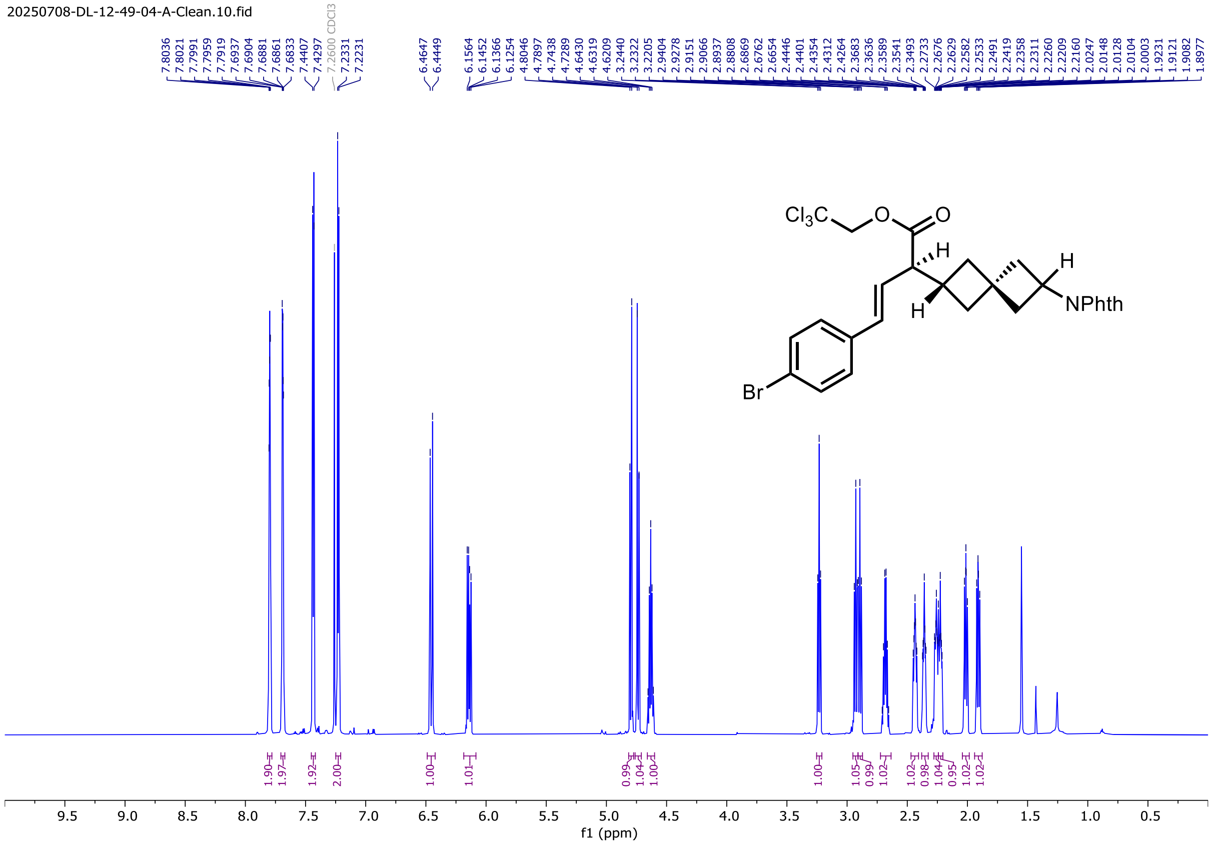
20250708-DL-12-49-04-A-Clean.10.fid
Cl3C
O
O
H
H
H
NPhth
Br
f1 (ppm)

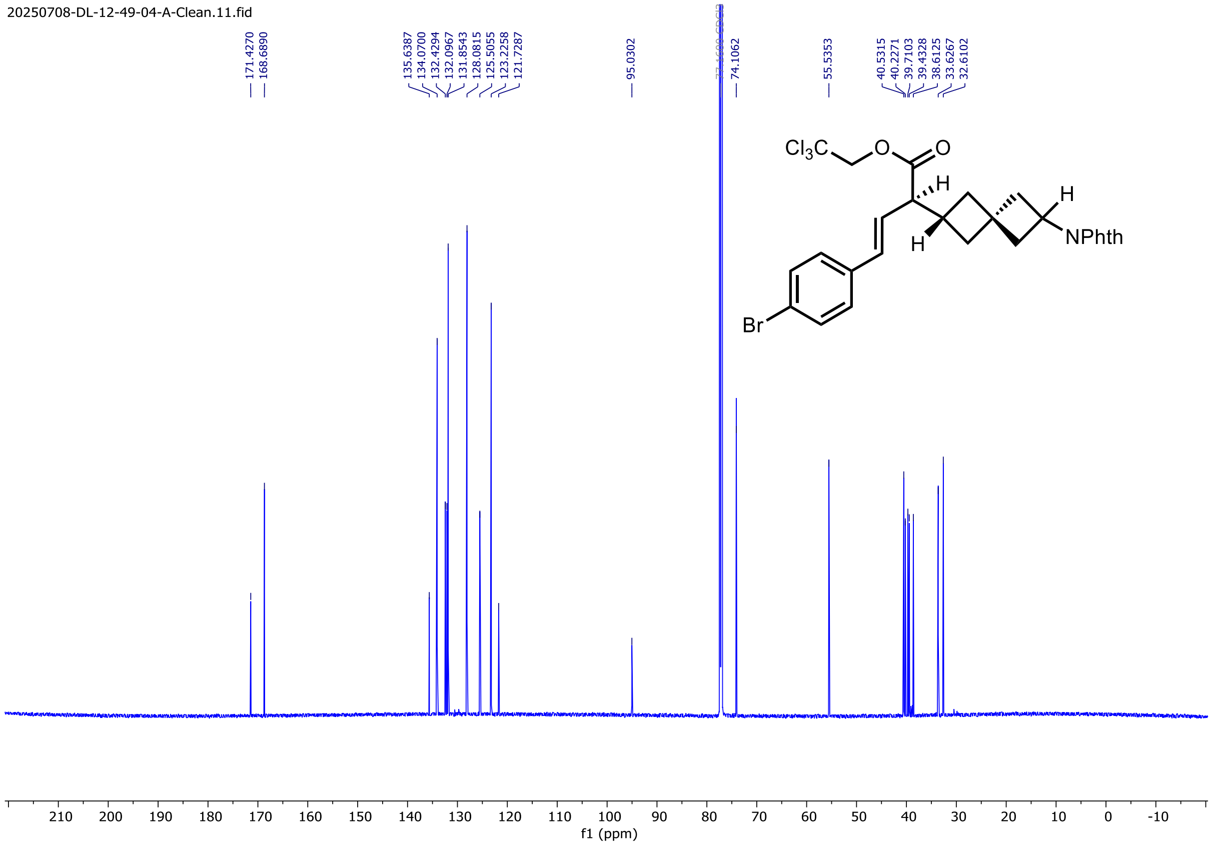
20250708-DL-12-49-04-A-Clean.11.fid
171.4270
168.6890
135.6387
134.0700
132.4294
132.0967
131.8543
128.0815
125.5055
123.2258
121.7287
95.0302
74.1062
55.5353
40.5315
40.2271
39.7103
39.4328
38.6125
33.6267
32.6102
Cl3C
O
O
H
H
H
NPhth
Br
f1 (ppm)

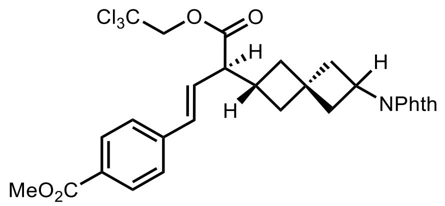

methyl 4-((3S,E)-3-(6-(1,3-dioxoisoindolin-2-yl)spiro[3.3]heptan-2-yl)-4-oxo-4-(2,2,2-trichloroethoxy)but-1-en-1-yl)benzoate (Compound 37)

Prepared according to general procedure for C-H functionalization, 2-(spiro[3.3]heptan-2-yl)isoindoline-1,3-dione (48.3 mg, 0.2 mmol, 1.0 equiv), Rh_2(*S*-MegaBNP)$_4$ (3.4 mg, 0.0001 mmol, 0.005 equiv), molecular sieve 4Å (100 wt%) and 2,2,2- HFIP (5 µL, 8.40 mg, 0.05 mmol, 0.25 equiv) in 0.5 ml CH_2Cl_2 were added a solution of methyl (E)-4-(3-diazo-4-oxo-4-(2,2,2-trichloroethoxy)but-1-en-1-yl)benzoate (151.0 mg, 0.4 mmol, 2.0 equiv) in 2.0 ml CH_2Cl_2 at 39℃ in 3 hours. The crude mixture was purified by flash chromatography (SiO_2, gradient 0%-25% Et_2O in hexane) afforded **compound 37** as a white amorphous solid (61.6 mg, 52% yield, 99% ee, 20:1 dr).

R_f (1Hex/2Et_2O) = 0.50 (CAM, UV 254 nm)

$[\alpha]^{20}_D$: 43.8° (c = 0.65 g/100 ml, $CHCl_3$, 99% ee)

^{1}H NMR (600 MHz, $CDCl_3$) δ 7.98 (d, *J* = 8.3 Hz, 2H), 7.79 (dd, *J* = 5.5, 3.0 Hz, 2H), 7.68 (dd, *J* = 5.5, 3.0 Hz, 2H), 7.42 (d, *J* = 8.3 Hz, 2H), 6.55 (d, *J* = 15.9 Hz, 1H), 6.27 (dd, *J* = 15.9, 9.0 Hz, 1H), 4.80 (d, *J* = 11.9 Hz, 1H), 4.74 (d, *J* = 11.9 Hz, 1H), 4.63 (p, *J* = 8.8 Hz, 1H), 3.91 (s, 3H), 3.27 (t, *J* = 9.4 Hz, 1H), 2.98 – 2.86 (m, 2H), 2.70 (dt, *J* = 9.7, 8.1 Hz, 1H), 2.44 (ddd, *J* = 10.8, 8.1, 4.7 Hz, 1H), 2.37 (ddd, *J* = 11.7, 7.8, 4.1 Hz, 1H), 2.30 – 2.20 (m, 2H), 2.03 (dd, *J* = 11.3, 8.3 Hz, 1H), 1.92 (dd, *J* = 11.9, 8.6 Hz, 1H).

^{13}C NMR (151 MHz, $CDCl_3$) δ 171.3, 168.7, 166.9, 141.1, 134.1, 132.7, 132.1, 130.1, 129.4, 127.4, 126.4, 123.2, 95.0, 74.1, 55.6, 52.2, 40.5, 40.2, 39.7, 39.5, 38.7, 33.6, 32.6.

HRMS (+p APCI) calcd. for [$C_{29}H_{27}O_6N^{35}Cl_3$] ([M+H]$^+$) 590.0899 found 590.0914.

HPLC (Chiralpak ADH column, 10% isopropanol in hexane, 1.0 mLmin^{-1}, 1.0 mgmL^{-1}, 60 min, UV 230 nm) retention times of 35.7 min (major) and 59.5 min (minor), 99% ee.

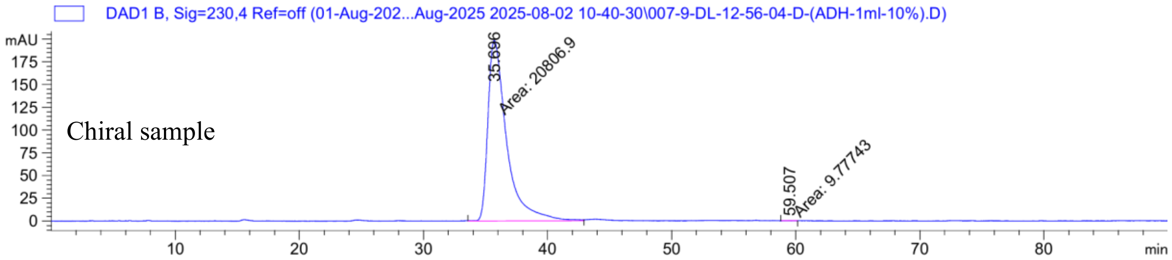

Signal 2: DAD1 B, Sig=230,4 Ref=off

Peak #	RetTime [min]	Type	Width [min]	Area [mAU*s]	Height [mAU]	Area %
1	35.696	MM	1.7497	2.08069e4	198.19003	99.9530
2	59.507	MM	0.5806	9.77743	2.80671e-1	0.0470

Totals : 2.08167e4 198.47070

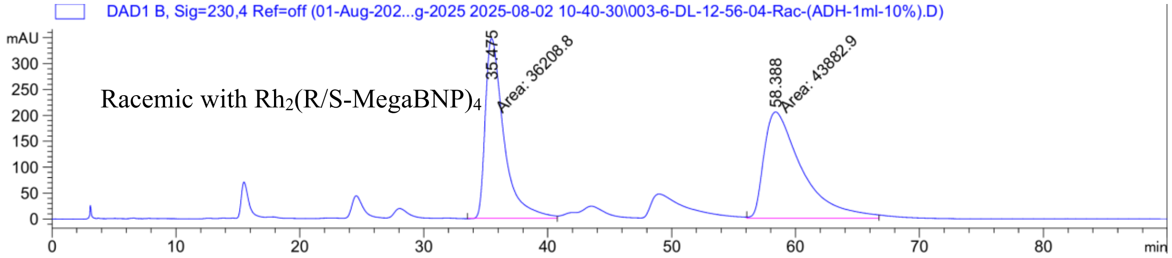

Signal 2: DAD1 B, Sig=230,4 Ref=off

Peak #	RetTime [min]	Type	Width [min]	Area [mAU*s]	Height [mAU]	Area %
1	35.475	MM	1.7383	3.62088e4	347.16547	45.2092
2	58.388	MM	3.5635	4.38829e4	205.24136	54.7908

Totals : 8.00918e4 552.40683

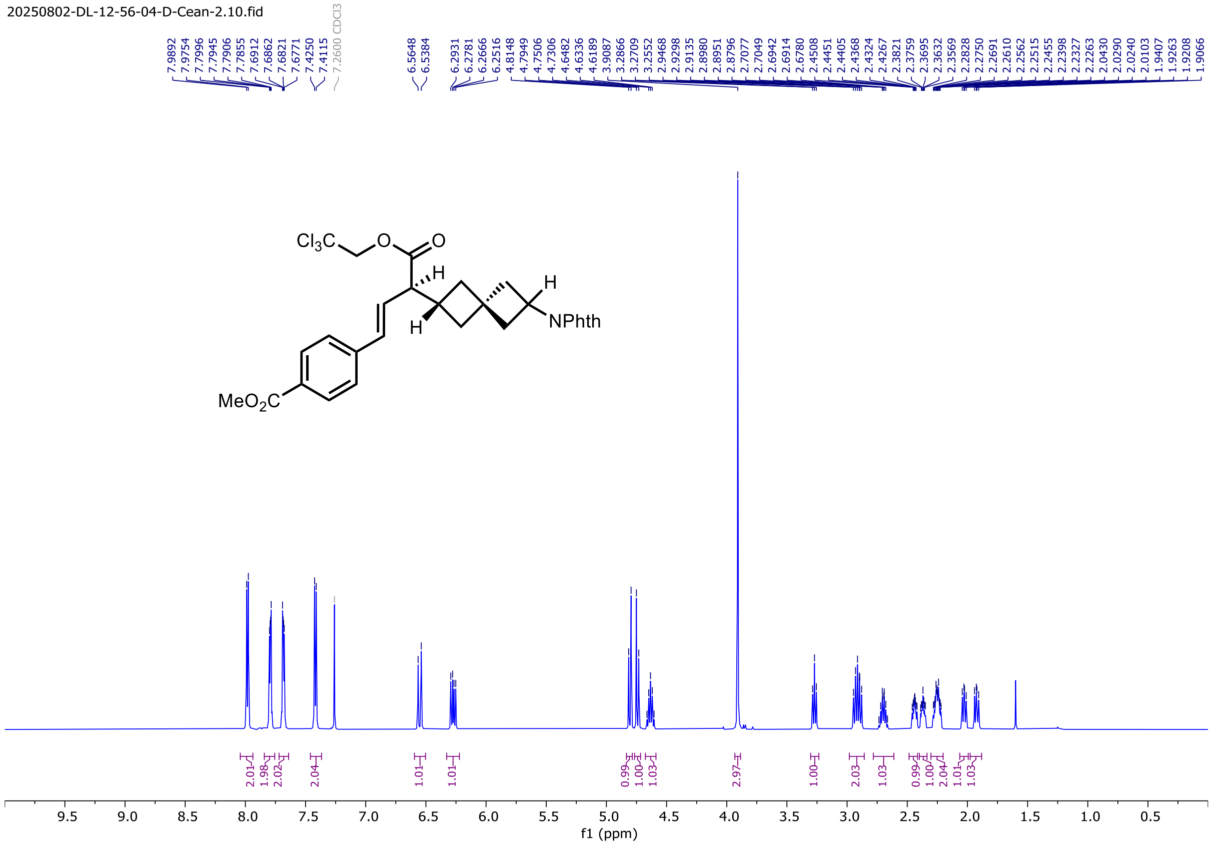
20250802-DL-12-56-04-D-Cean-2.10.fid
7.9892 7.9754 7.7996 7.7945 7.7906 7.7855 7.6912 7.6862 7.6821 7.6771 7.4250 7.4115 7.2600 CDCl3
6.5648 6.5384 6.2931 6.2781 6.2666 6.2516 4.8148 4.7949 4.7506 4.7306 4.6482 4.6336 4.6189 3.9087 3.2866 3.2709 3.2552 2.9468 2.9298 2.9135 2.8980 2.8951 2.8796 2.7077 2.7049 2.6942 2.6914 2.6780 2.4508 2.4451 2.4405 2.4368 2.4324 2.4267 2.3821 2.3759 2.3695 2.3632 2.3569 2.2828 2.2750 2.2691 2.2610 2.2562 2.2515 2.2455 2.2398 2.2327 2.2263 2.0430 2.0290 2.0240 2.0103 1.9407 1.9263 1.9208 1.9066
Cl3C
O
O
H
H
H
NPhth
MeO2C
2.01 1.98 2.02 2.04 1.01 1.01 0.99 1.00 1.03 2.97 1.00 2.03 1.03 0.99 1.00 2.04 1.01 1.03
f1 (ppm)

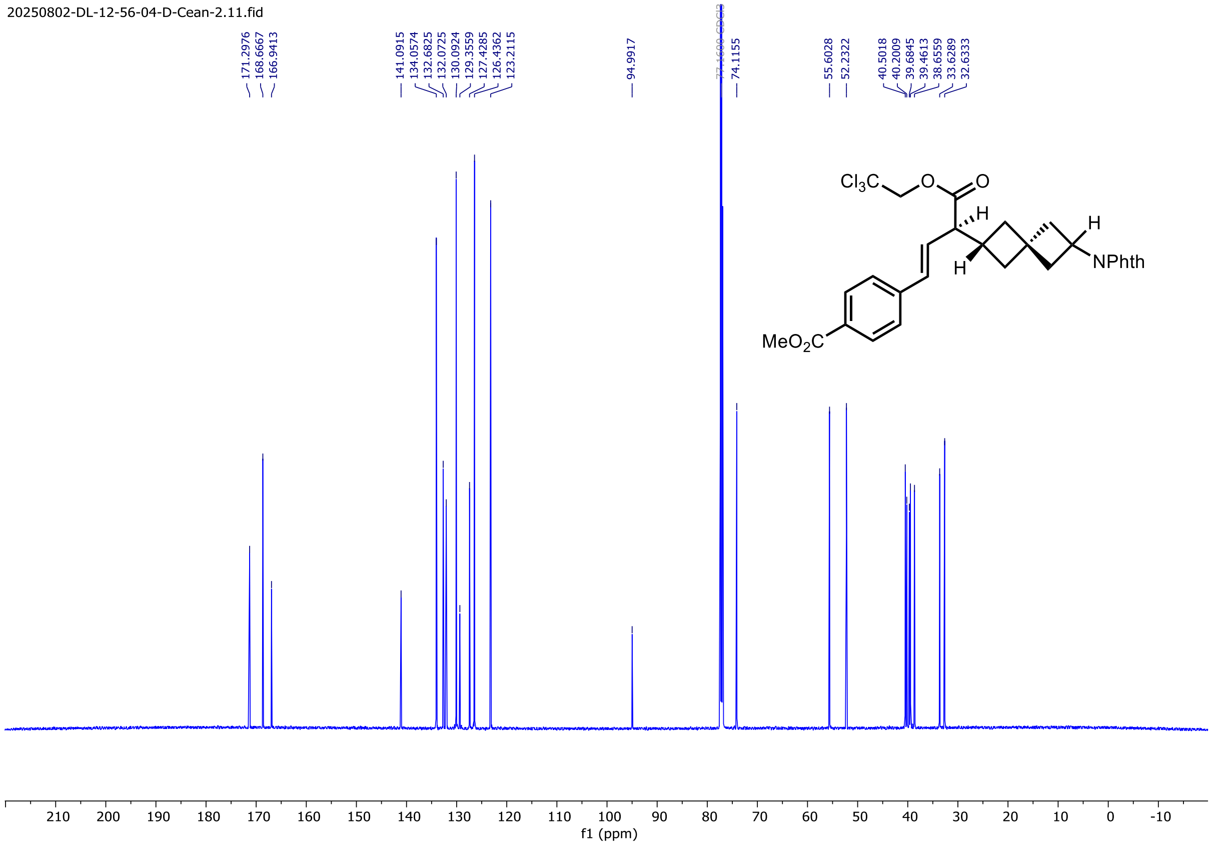
20250802-DL-12-56-04-D-Cean-2.11.fid
171.2976 168.6667 166.9413 141.0915 134.0574 132.6825 132.0725 130.0924 129.3559 127.4285 126.4362 123.2115 94.9917 77.1600 CDCl3 74.1155 55.6028 52.2322 40.5018 40.2009 39.6845 39.4613 38.6559 33.6289 32.6333
Cl3C
O
O
H
H
H
NPhth
MeO2C
f1 (ppm)

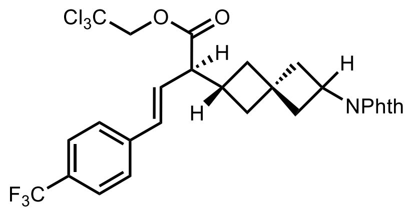

2,2,2-trichloroethyl (2S,E)-2-(6-(1,3-dioxoisoindolin-2-yl)spiro[3.3]heptan-2-yl)-4-(4-(trifluoromethyl)phenyl)but-3-enoate (Compound 38)

Prepared according to general procedure for C-H functionalization, 2-(spiro[3.3]heptan-2-yl)isoindoline-1,3-dione (48.3 mg, 0.2 mmol, 1.0 equiv), Rh_2(*S*-MegaBNP)$_4$ (3.4 mg, 0.0001 mmol, 0.005 equiv), molecular sieve 4Å (100 wt%) and 2,2,2- HFIP (5 μL, 8.40 mg, 0.05 mmol, 0.25 equiv) in 0.5 ml CH_2Cl_2 were added a solution of 2,2,2-trichloroethyl (E)-2-diazo-4-(4-(trifluoromethyl)phenyl)but-3-enoate (155.0 mg, 0.4 mmol, 2.0 equiv) in 2.0 ml CH_2Cl_2 at 39℃ in 3 hours. The crude mixture was purified by flash chromatography (SiO_2, gradient 0%-25% Et_2O in hexane) afforded **compound 38** as a white amorphous solid (71.3 mg, 59% yield, 95% ee, 20:1 dr).

R_f (1Hex/2Et_2O) = 0.50 (CAM, UV 254 nm)

$[\alpha]^{20}_D$: 31.9° (c = 0.76 g/100 ml, $CHCl_3$, 95% ee)

^{1}H NMR (600 MHz, $CDCl_3$) δ 7.82 – 7.78 (m, 2H), 7.69 (dd, *J* = 4.5, 1.7 Hz, 2H), 7.57 (d, *J* = 8.0 Hz, 2H), 7.46 (d, *J* = 8.0 Hz, 2H), 6.55 (d, *J* = 15.9 Hz, 1H), 6.26 (dd, *J* = 15.9, 9.0 Hz, 1H), 4.81 (d, *J* = 11.9 Hz, 1H), 4.75 (d, *J* = 11.9 Hz, 1H), 4.64 (p, *J* = 8.8 Hz, 1H), 3.27 (t, *J* = 9.4 Hz, 1H), 2.92 (dt, *J* = 20.3, 10.3 Hz, 2H), 2.76 – 2.66 (m, 1H), 2.44 (ddd, *J* = 10.9, 8.2, 4.7 Hz, 1H), 2.37 (ddd, *J* = 11.6, 7.8, 4.0 Hz, 1H), 2.30 – 2.20 (m, 2H), 2.03 (dd, *J* = 11.3, 8.3 Hz, 1H), 1.92 (dd, *J* = 11.8, 8.6 Hz, 1H).

^{13}C NMR (151 MHz, $CDCl_3$) δ 171.3, 168.7, 140.1, 134.1, 132.3, 132.1, 129.7 (q, *J* = 32.4 Hz), 127.4, 126.7, 125.7 (q, *J* = 3.8 Hz), 124.3 (q, *J* = 271.9 Hz), 123.2, 95.0, 74.1, 55.5, 40.5, 40.2, 39.7, 39.5, 38.6, 33.6, 32.6.

^{19}F NMR (565 MHz, $CDCl_3$) δ -62.52.

HRMS (+p APCI) calcd. for [$C_{28}H_{24}O_4N^{35}Cl_3F_3$] ($[M+H]^+$) 600.0718 found 600.0730.

HPLC (Chiralpak ADH column, 5% isopropanol in hexane, 1.0 mLmin^{-1}, 1.0 mgmL^{-1}, 60 min, UV 230 nm) retention times of 24.8 min (major) and 31.1 min (minor), 95% ee.

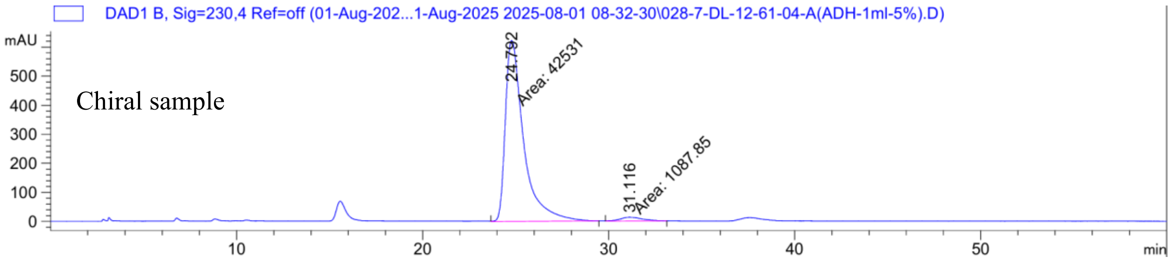

Signal 2: DAD1 B, Sig=230,4 Ref=off

Peak #	RetTime [min]	Type	Width [min]	Area [mAU*s]	Height [mAU]	Area %
1	24.792	MM	1.1382	4.25310e4	622.77869	97.5060
2	31.116	MM	1.4036	1087.84656	12.91776	2.4940

Totals : 4.36189e4 635.69645

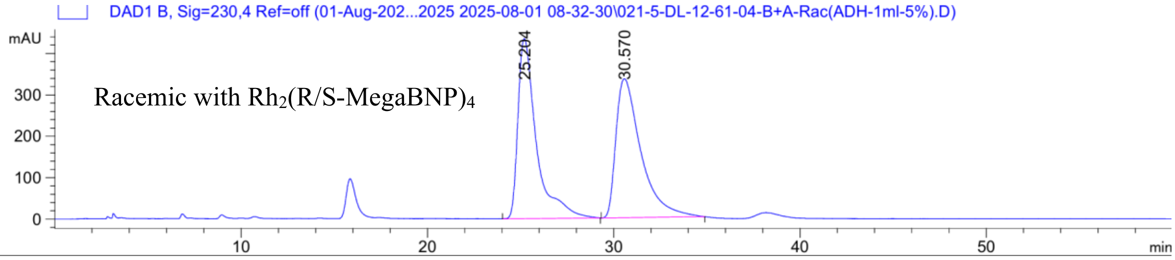

Signal 2: DAD1 B, Sig=230,4 Ref=off

Peak #	RetTime [min]	Type	Width [min]	Area [mAU*s]	Height [mAU]	Area %
1	25.204	VV R	0.8059	2.99561e4	434.48880	48.8817
2	30.570	VV R	1.0917	3.13268e4	335.70709	51.1183

Totals : 6.12829e4 770.19589

20250802-DL-12-61-04-A-Clean-3.12.fid

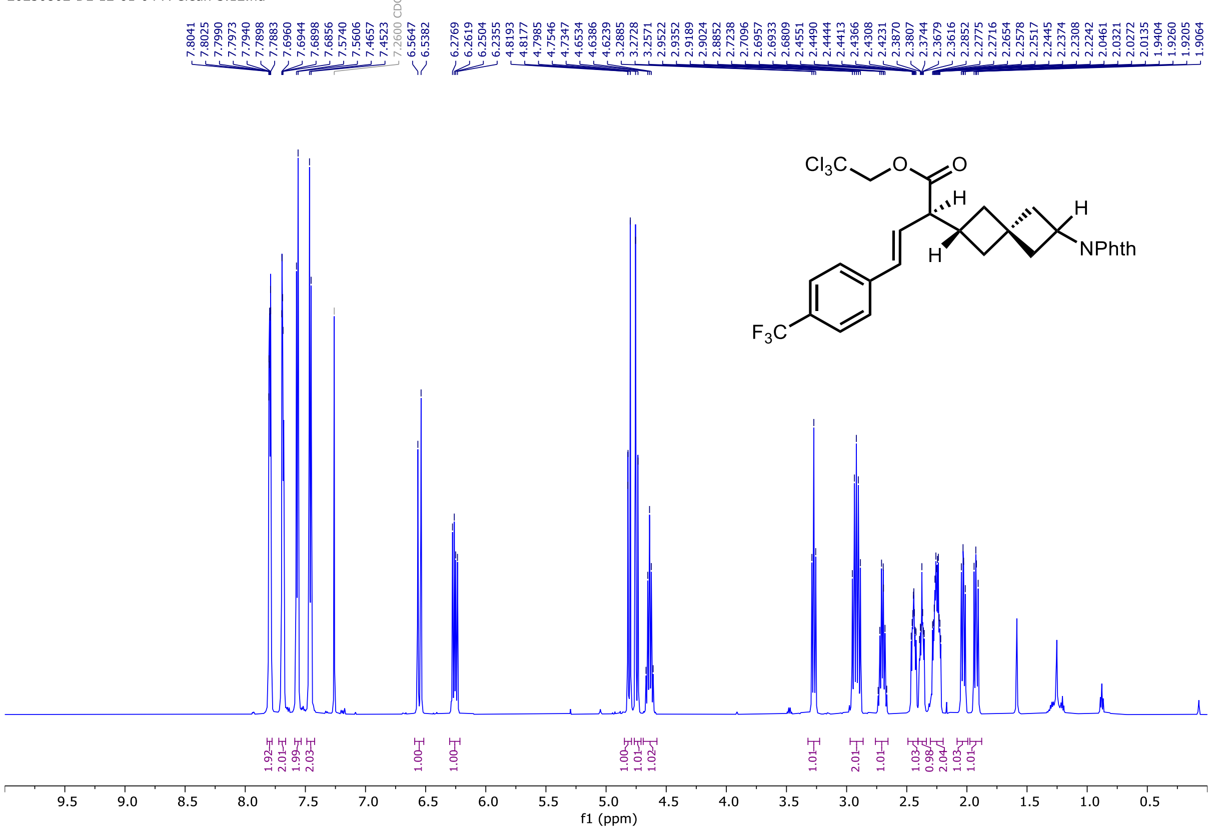

20250802-DL-12-61-04-A-Clean-3.13.fid

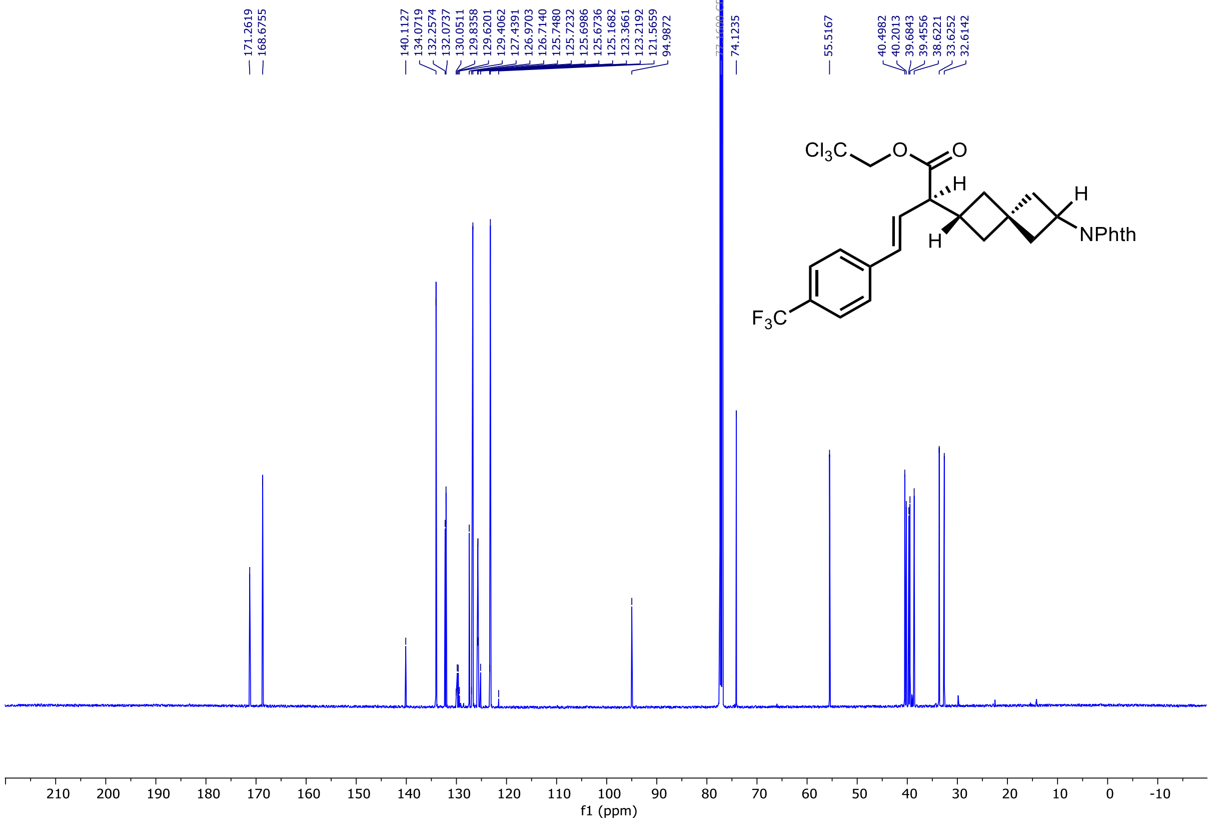

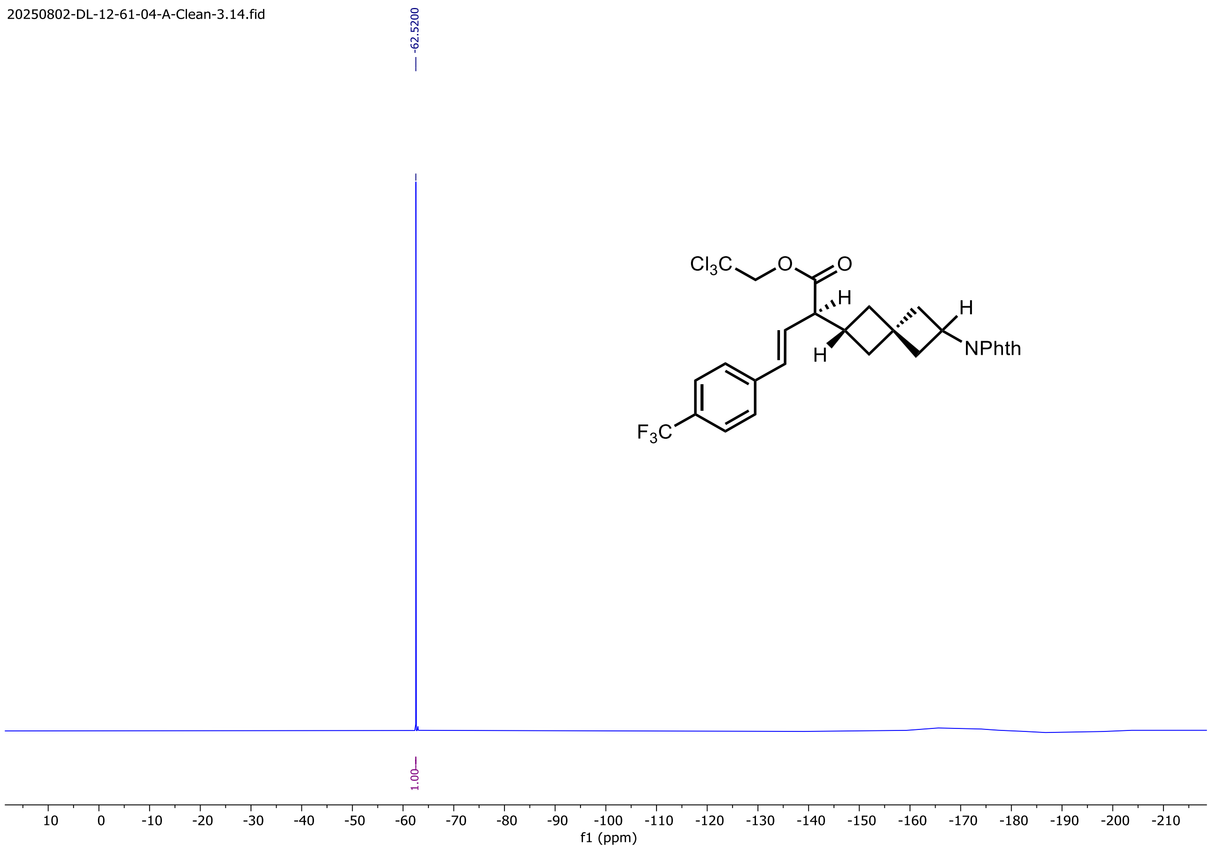
20250802-DL-12-61-04-A-Clean-3.14.fid
-62.5200
1.00
10 0 -10 -20 -30 -40 -50 -60 -70 -80 -90 -100 -110 -120 -130 -140 -150 -160 -170 -180 -190 -200 -210
f1 (ppm)
Cl3C
O
O
H
H
H
NPhth
F3C

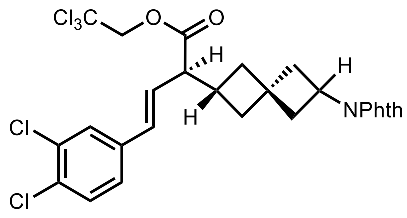

2,2,2-trichloroethyl (2S,E)-4-(3,4-dichlorophenyl)-2-(6-(1,3-dioxoisoindolin-2-yl)spiro[3.3]heptan-2-yl)but-3-enoate (Compound 39)

Prepared according to general procedure for C-H functionalization, 2-(spiro[3.3]heptan-2-yl)isoindoline-1,3-dione (48.3 mg, 0.2 mmol, 1.0 equiv), Rh_2(*S*-MegaBNP)$_4$ (3.4 mg, 0.0001 mmol, 0.005 equiv), molecular sieve 4Å (100 wt%) and 2,2,2- HFIP (5 µL, 8.40 mg, 0.05 mmol, 0.25 equiv) in 0.5 ml CH_2Cl_2 were added a solution of 2,2,2-trichloroethyl (E)-2-diazo-4-(3,4-dichlorophenyl)but-3-enoate (155.0 mg, 0.4 mmol, 2.0 equiv) in 2.0 ml CH_2Cl_2 at 39℃ in 3 hours. The crude mixture was purified by flash chromatography (SiO_2, gradient 0%-25% Et_2O in hexane) afforded **compound 39** as a white amorphous solid (56.4 mg, 47% yield, 96% ee, 18:1 dr).

R_f (1Hex/2Et_2O) = 0.50 (CAM, UV 254 nm)

$[\alpha]^{20}_D$: 34.8° (c = 0.30 g/100 ml, $CHCl_3$, 96% ee)

^{1}H NMR (600 MHz, $CDCl_3$) δ 7.80 (dd, *J* = 5.5, 3.0 Hz, 2H), 7.69 (dd, *J* = 5.5, 3.0 Hz, 2H), 7.44 (d, *J* = 2.1 Hz, 1H), 7.38 (d, *J* = 8.3 Hz, 1H), 7.18 (dd, *J* = 8.3, 2.1 Hz, 1H), 6.42 (d, *J* = 15.9 Hz, 1H), 6.15 (dd, *J* = 15.9, 8.9 Hz, 1H), 4.80 (d, *J* = 11.9 Hz, 1H), 4.74 (d, *J* = 11.9 Hz, 1H), 4.64 (p, *J* = 8.9 Hz, 1H), 3.24 (t, *J* = 9.4 Hz, 1H), 2.91 (dt, *J* = 20.9, 10.3 Hz, 2H), 2.77 – 2.60 (m, 1H), 2.44 (ddd, *J* = 10.7, 8.2, 4.7 Hz, 1H), 2.36 (ddd, *J* = 11.6, 7.8, 4.0 Hz, 1H), 2.24 (dddd, *J* = 19.5, 11.9, 8.2, 4.4 Hz, 2H), 2.02 (dd, *J* = 11.3, 8.3 Hz, 1H), 1.90 (dd, *J* = 11.8, 8.6 Hz, 1H).

^{13}C NMR (151 MHz, $CDCl_3$) δ 171.2, 168.7, 136.8, 134.1, 132.9, 132.1, 131.6, 131.3, 130.6, 128.3, 126.8, 125.7, 123.2, 95.0, 74.1, 55.4, 40.5, 40.2, 39.7, 39.5, 38.6, 33.6, 32.6.

HRMS (+p APCI) calcd. for [$C_{27}H_{23}O_4N^{35}Cl_5$] ([M+H]$^+$) 600.0064 found 600.0080.

HPLC (Chiralpak ADH column, 5% isopropanol in hexane, 1.0 mLmin^{-1}, 1.0 mgmL^{-1}, 60 min, UV 230 nm) retention times of 27.5 min (major) and 36.2 min (minor), 96% ee.

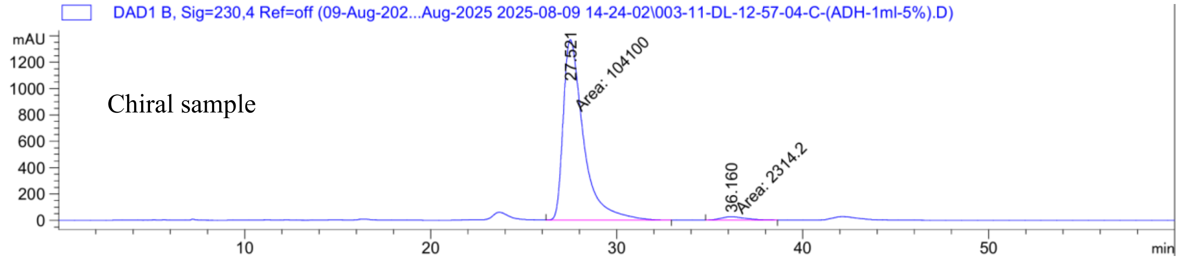

Signal 2: DAD1 B, Sig=230,4 Ref=off

Peak #	RetTime [min]	Type	Width [min]	Area [mAU*s]	Height [mAU]	Area %
1	27.521	MM	1.2667	1.04100e5	1369.65833	97.8253
2	36.160	MM	1.5712	2314.19556	24.54758	2.1747
Totals :				1.06414e5	1394.20591	

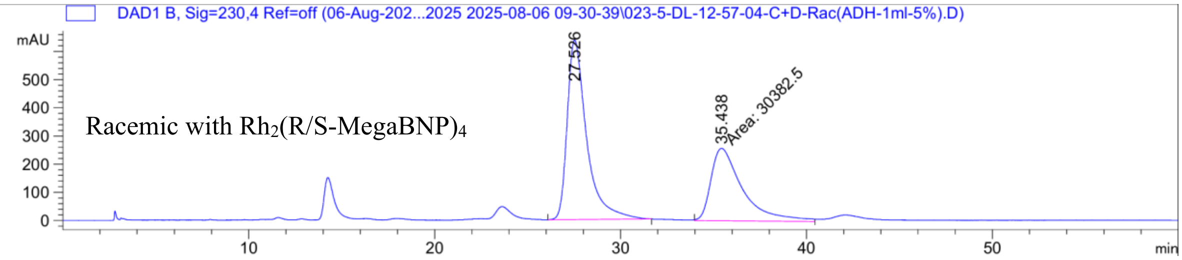

Signal 2: DAD1 B, Sig=230,4 Ref=off

Peak #	RetTime [min]	Type	Width [min]	Area [mAU*s]	Height [mAU]	Area %
1	27.526	VB R	0.8507	4.63862e4	636.86725	60.4233
2	35.438	MM	1.9679	3.03825e4	257.31543	39.5767
Totals :				7.67688e4	894.18268	

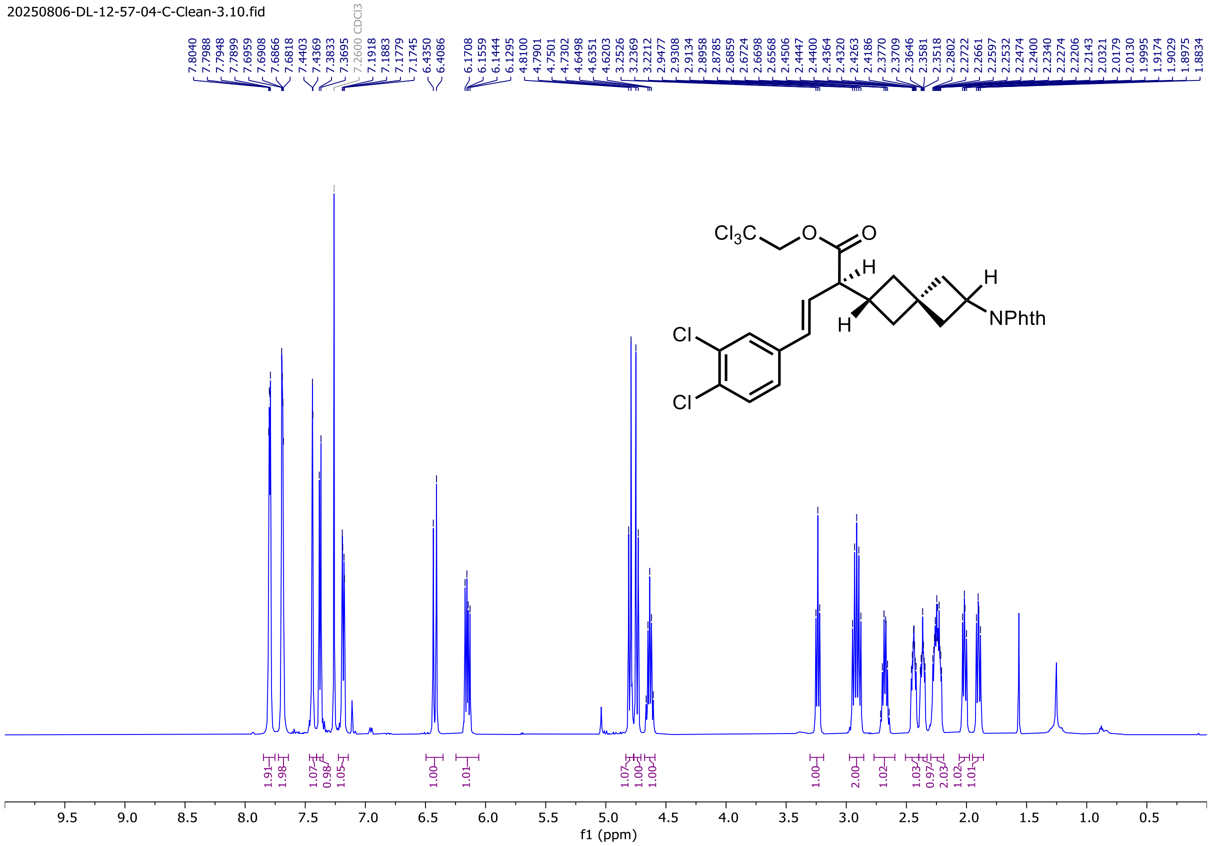

20250806-DL-12-57-04-C-Clean-3.10.fid
7.8040
7.7988
7.7948
7.7899
7.6959
7.6908
7.6866
7.6818
7.4403
7.4369
7.3833
7.3695
7.2600 CDCl3
7.1918
7.1883
7.1779
7.1745
6.4350
6.4086
6.1708
6.1559
6.1444
6.1295
4.8100
4.7901
4.7501
4.7302
4.6498
4.6351
4.6203
3.2526
3.2369
3.2212
2.9477
2.9308
2.9134
2.8958
2.8785
2.6859
2.6724
2.6698
2.6568
2.4506
2.4447
2.4400
2.4364
2.4320
2.4263
2.4186
2.3770
2.3709
2.3646
2.3581
2.3518
2.2802
2.2722
2.2661
2.2597
2.2532
2.2474
2.2400
2.2340
2.2274
2.2206
2.2143
2.0321
2.0179
2.0130
1.9995
1.9174
1.9029
1.8975
1.8834
Cl3C
O
O
H
H
H
NPhth
Cl
Cl
1.91
1.98
1.07
0.98
1.05
1.00
1.01
1.07
1.00
1.00
1.00
2.00
1.02
1.03
0.97
2.03
1.02
1.01
9.5
9.0
8.5
8.0
7.5
7.0
6.5
6.0
5.5
5.0
4.5
4.0
3.5
3.0
2.5
2.0
1.5
1.0
0.5
f1 (ppm)

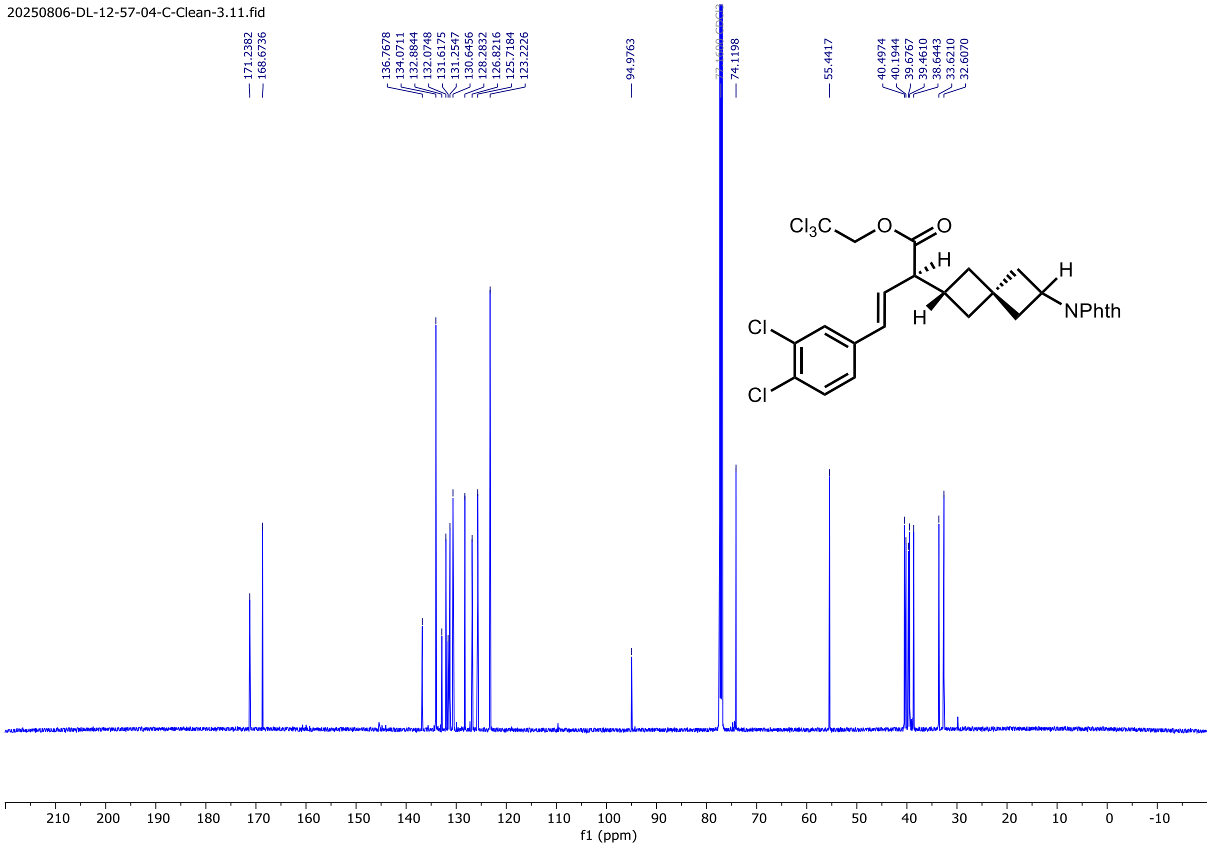

20250806-DL-12-57-04-C-Clean-3.11.fid
171.2382
168.6736
136.7678
134.0711
132.8844
132.0748
131.6175
131.2547
130.6456
128.2832
126.8216
125.7184
123.2226
94.9763
77.1600 CDCl3
74.1198
55.4417
40.4974
40.1944
39.6767
39.4610
38.6443
33.6210
32.6070
Cl3C
O
O
H
H
H
NPhth
Cl
Cl
210
200
190
180
170
160
150
140
130
120
110
100
90
80
70
60
50
40
30
20
10
0
-10
f1 (ppm)

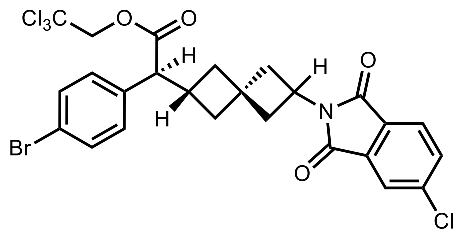

2,2,2-trichloroethyl (2S)-2-(4-bromophenyl)-2-(6-(5-chloro-1,3-dioxoisoindolin-2-yl)spiro[3.3]heptan-2-yl)acetate (Compound 40)

Prepared according to general procedure for C-H functionalization, 5-chloro-2-(spiro[3.3]heptan-2-yl)isoindoline-1,3-dione (55.1 mg, 0.2 mmol, 1.0 equiv), Rh_2(*S*-MegaBNP)$_4$ (3.4 mg, 0.0001 mmol, 0.005 equiv), molecular sieve 4Å (100 wt%) and 2,2,2- HFIP (5 µL, 8.40 mg, 0.05 mmol, 0.25 equiv) in 0.5 ml CH_2Cl_2 were added a solution of trichloroethyl 2-(4-bromophenyl)-2-diazoacetate (149.0 mg, 0.4 mmol, 2.0 equiv) in 2.0 ml CH_2Cl_2 at 39℃ in 3 hours. The crude mixture was purified by flash chromatography (SiO_2, gradient 0%-25% Et_2O in hexane) afforded **compound 40** as a white amorphous solid (84.0 mg, 68% yield, 92% ee, 21:1 dr).

R_f (1Hex/2Et_2O) = 0.50 (CAM, UV 254 nm)

$[\alpha]^{20}_D$: 25.6° (c = 0.76 g/100 ml, $CHCl_3$, 92% ee)

^1H NMR (800 MHz, $CDCl_3$) δ 7.75 (s, 1H), 7.72 (d, *J* = 7.9 Hz, 1H), 7.64 (d, *J* = 7.9 Hz, 1H), 7.45 (d, *J* = 8.1 Hz, 2H), 7.19 (d, *J* = 8.1 Hz, 2H), 4.77 (d, *J* = 12.0 Hz, 1H), 4.67 (d, *J* = 12.0 Hz, 1H), 4.61 (p, *J* = 8.8 Hz, 1H), 3.59 (d, *J* = 11.0 Hz, 1H), 2.91 (dt, *J* = 11.0, 8.2 Hz, 1H), 2.87 (t, *J* = 10.2 Hz, 1H), 2.84 (t, *J* = 10.4 Hz, 1H), 2.47 – 2.41 (m, 2H), 2.25 (ddd, *J* = 12.2, 8.2, 4.7 Hz, 1H), 2.02 (td, *J* = 7.9, 4.0 Hz, 1H), 1.99 (dd, *J* = 11.5, 8.5 Hz, 1H), 1.73 (dd, *J* = 11.8, 8.7 Hz, 1H).

^{13}C NMR (201 MHz, $CDCl_3$) δ 171.2, 167.6, 167.3, 140.7, 135.6, 134.1, 133.7, 131.9, 130.1, 130.1, 124.5, 123.7, 121.8, 94.9, 74.2, 57.5, 40.8, 40.1, 40.1, 39.6, 38.5, 33.5, 33.2.

HRMS (+p APCI) calcd. for [$C_{25}H_{21}O_4N^{79}Br^{35}Cl_4$] ($[M+H]^+$) 617.9403 found 617.9419.

HPLC (Chiralpak ADH column, 5% isopropanol in hexane, 1.0 mLmin^{-1}, 1.0 mgmL^{-1}, 90 min, UV 230 nm) retention times of 23.7 min (major) and 26.1 min (minor), 92% ee.

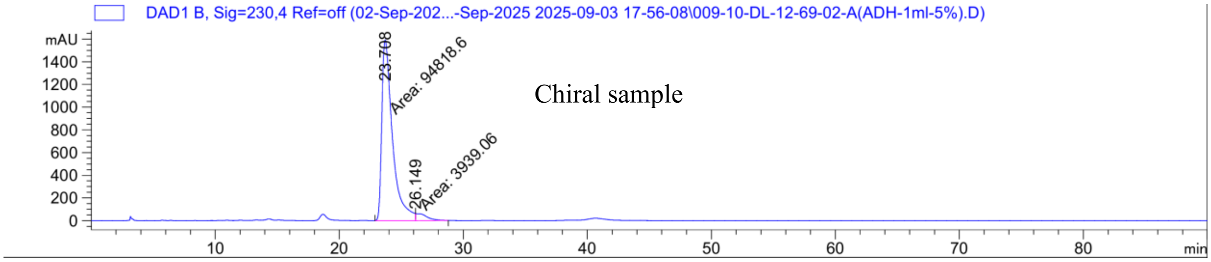

Signal 2: DAD1 B, Sig=230,4 Ref=off

Peak #	RetTime [min]	Type	Width [min]	Area [mAU*s]	Height [mAU]	Area %
1	23.708	MF	0.9923	9.48186e4	1592.53149	96.0114
2	26.149	FM	1.0508	3939.05615	62.47935	3.9886

Totals : 9.87577e4 1655.01085

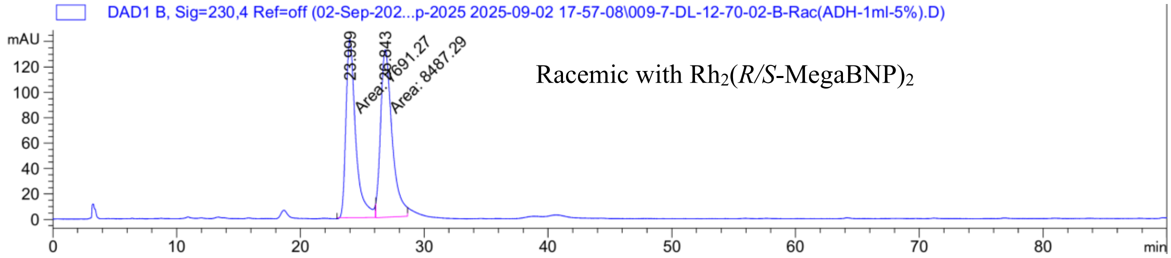

Signal 2: DAD1 B, Sig=230,4 Ref=off

Peak #	RetTime [min]	Type	Width [min]	Area [mAU*s]	Height [mAU]	Area %
1	23.999	MM	0.9123	7691.27441	140.51117	47.5399
2	26.843	MM	1.0721	8487.28906	131.93719	52.4601

Totals : 1.61786e4 272.44836

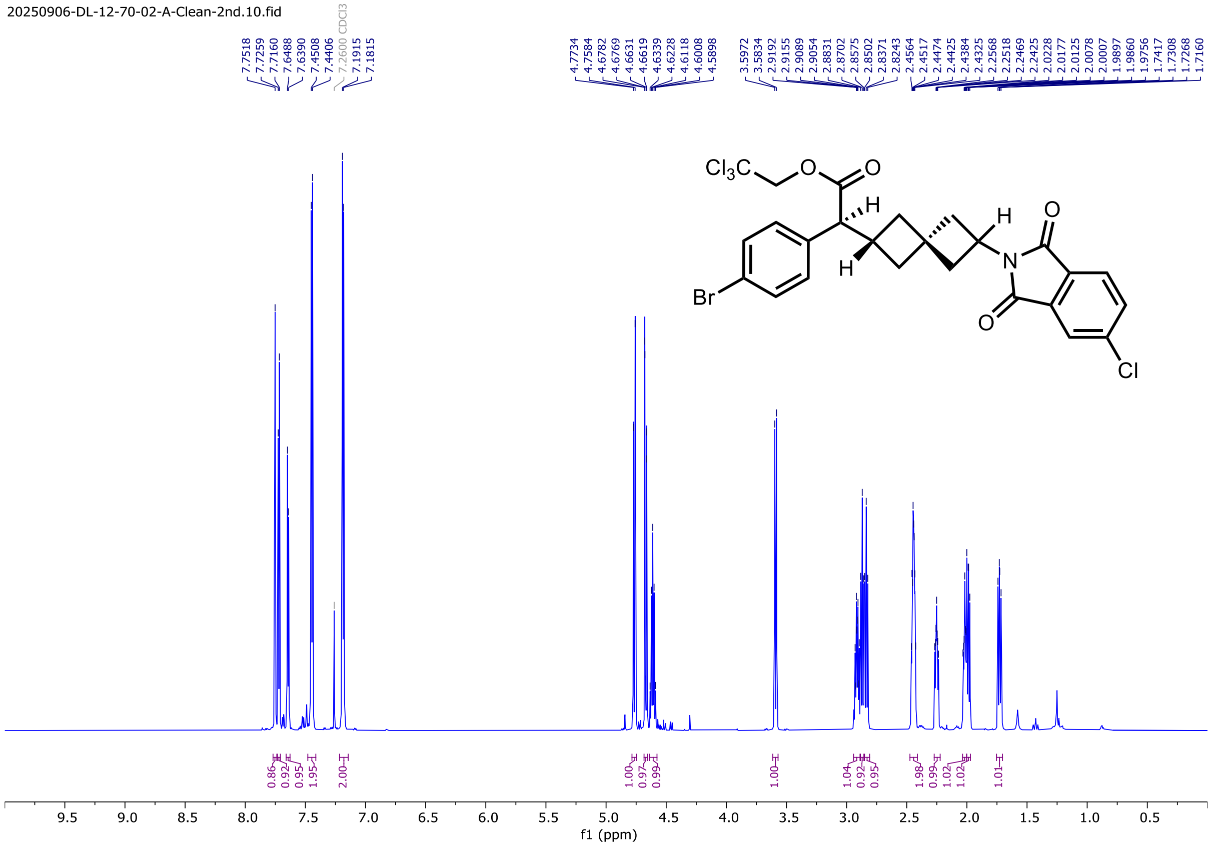
20250906-DL-12-70-02-A-Clean-2nd.10.fid
7.7518 7.7259 7.7160 7.6488 7.6390 7.4508 7.4406 7.2600 CDCl3 7.1915 7.1815
4.7734 4.7584 4.6782 4.6769 4.6631 4.6619 4.6339 4.6228 4.6118 4.6008 4.5898
3.5972 3.5834 2.9192 2.9155 2.9089 2.9054 2.8831 2.8702 2.8575 2.8502 2.8371 2.8243 2.4564 2.4517 2.4474 2.4425 2.4384 2.4325 2.2568 2.2518 2.2469 2.2425 2.0228 2.0177 2.0125 2.0078 2.0007 1.9897 1.9860 1.9756 1.7417 1.7308 1.7268 1.7160
0.86 0.92 0.95 1.95 2.00 1.00 0.97 0.99 1.00 1.04 0.92 0.95 1.98 0.99 1.02 1.02 1.01
Cl3C O O H H H N O O Br Cl
f1 (ppm)

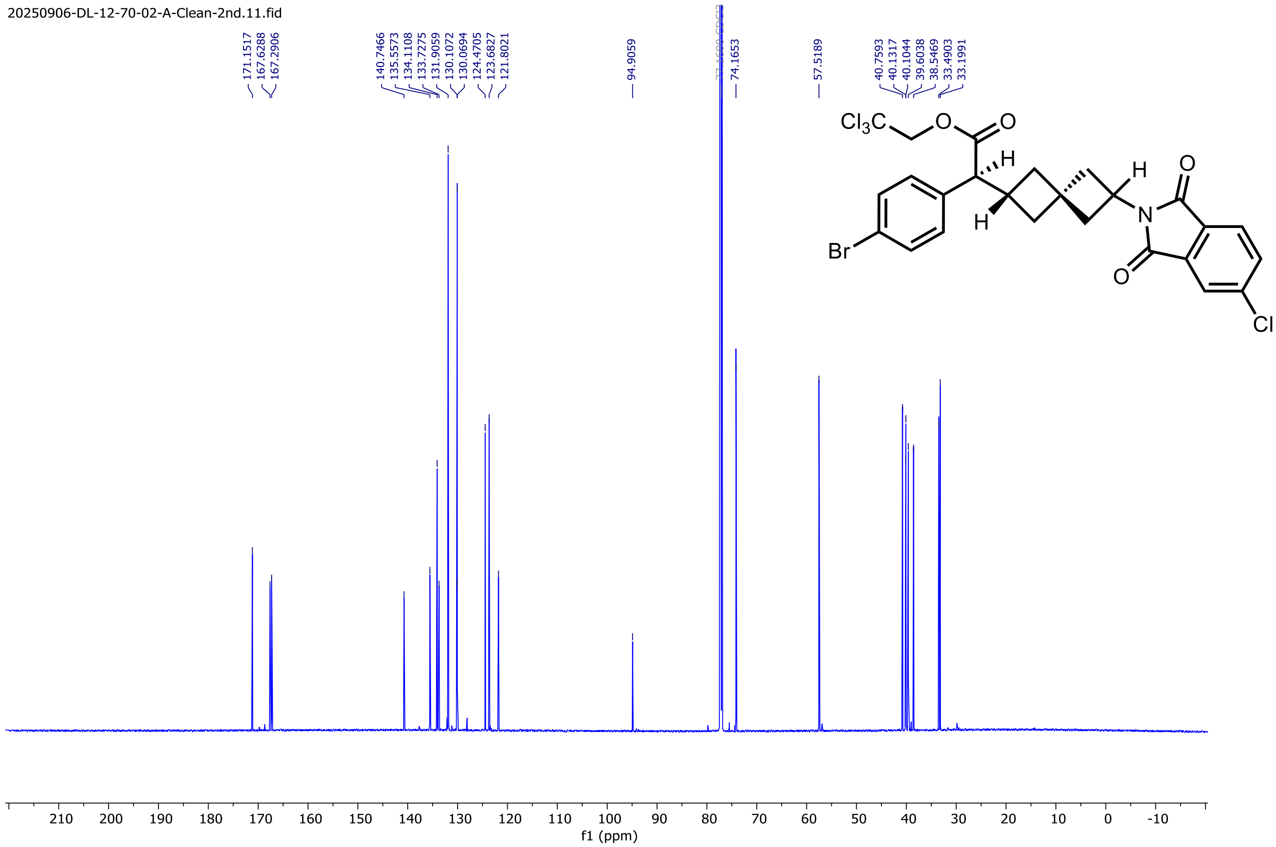
20250906-DL-12-70-02-A-Clean-2nd.11.fid
171.1517 167.6288 167.2906
140.7466 135.5573 134.1108 133.7275 131.9059 130.1072 130.0694 124.4705 123.6827 121.8021
94.9059
77.1600 CDCl3
74.1653
57.5189
40.7593 40.1317 40.1044 39.6038 38.5469 33.4903 33.1991
Cl3C O O H H H N O O Br Cl
f1 (ppm)

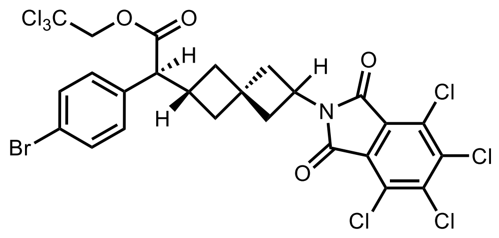

2,2,2-trichloroethyl (2S)-2-(4-bromophenyl)-2-(6-(4,5,6,7-tetrachloro-1,3-dioxoisoindolin-2-yl)spiro[3.3]heptan-2-yl)acetate (Compound 41)

Prepared according to general procedure for C-H functionalization, 4,5,6,7-tetrachloro-2-(spiro[3.3]heptan-2-yl)isoindoline-1,3-dione (75.8 mg, 0.2 mmol, 1.0 equiv), Rh_2(*S*-MegaBNP)$_4$ (3.4 mg, 0.0001 mmol, 0.005 equiv), molecular sieve 4Å (100 wt%) and 2,2,2- HFIP (5 µL, 8.40 mg, 0.05 mmol, 0.25 equiv) in 0.5 ml CH_2Cl_2 were added a solution of trichloroethyl 2-(4-bromophenyl)-2-diazoacetate (149.0 mg, 0.4 mmol, 2.0 equiv) in 2.0 ml CH_2Cl_2 at 39℃ in 3 hours. The crude mixture was purified by flash chromatography (SiO_2, gradient 0%-25% Et_2O in hexane) afforded **compound 41** as a white amorphous solid (101.3 mg, 70% yield, 96% ee, 10:1 dr).

R$_f$ (1Hex/2Et_2O) = 0.50 (CAM, UV 254 nm)

$[\alpha]^{20}_D$: 21.0° (c = 0.63 g/100 ml, $CHCl_3$, 96% ee)

^{1}H NMR (600 MHz, $CDCl_3$) δ 7.45 (d, *J* = 8.4 Hz, 2H), 7.18 (d, *J* = 8.4 Hz, 2H), 4.77 (d, *J* = 11.9 Hz, 1H), 4.70 – 4.60 (m, 2H), 3.59 (d, *J* = 11.0 Hz, 1H), 2.98 – 2.79 (m, 3H), 2.53 – 2.41 (m, 2H), 2.32 – 2.24 (m, 1H), 2.07 – 1.96 (m, 2H), 1.74 (dd, *J* = 12.0, 8.7 Hz, 1H).

^{13}C NMR (151 MHz, $CDCl_3$) δ 171.1, 163.8, 140.2, 135.5, 131.9, 130.1, 129.7, 127.5, 121.8, 94.9, 74.2, 57.5, 41.3, 40.1, 40.0, 39.4, 38.7, 33.5, 33.2.

HRMS (+p APCI) calcd. for [$C_{25}H_{18}O_4N^{79}Br^{35}Cl_7$] ([M+H]$^+$) 719.8233 found 719.8250.

SFC (OZ3, 25% (50% methanol in isopropanol with 0.2% Formic Acid) in CO_2, 2.5 mL/min, 1.0 mg/ml, UV 230 nm) retention times of 4.61 min (major) and 5.38 min (minor), 96% ee.

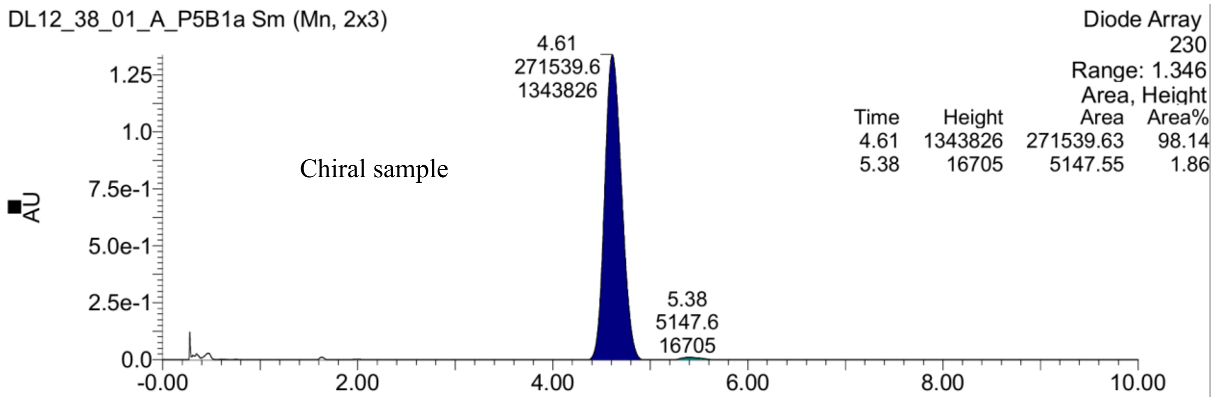
DL12_38_01_A_P5B1a Sm (Mn, 2x3)
Diode Array
230
Range: 1.346
Area, Height
Time Height Area Area%
4.61 1343826 271539.63 98.14
5.38 16705 5147.55 1.86
Chiral sample
4.61
271539.6
1343826
5.38
5147.6
16705
AU

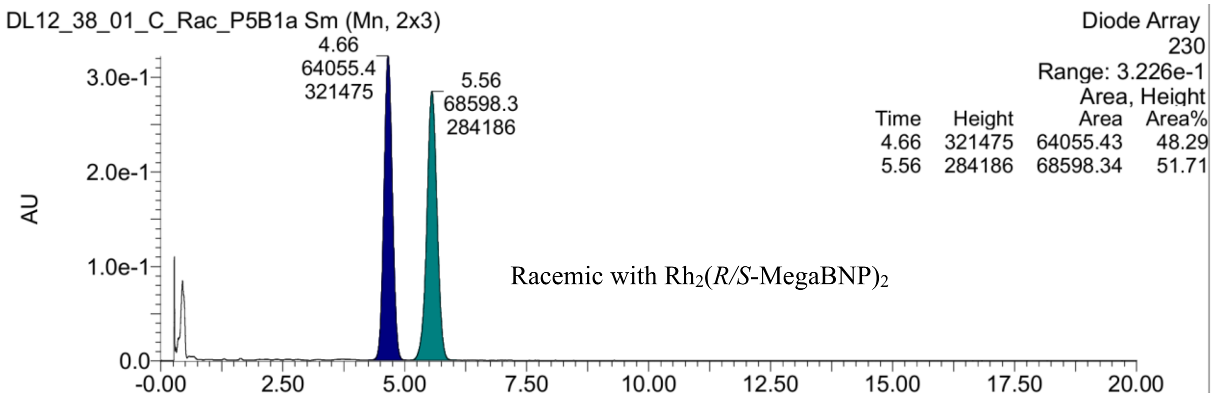
DL12_38_01_C_Rac_P5B1a Sm (Mn, 2x3)
Diode Array
230
Range: 3.226e-1
Area, Height
Time Height Area Area%
4.66 321475 64055.43 48.29
5.56 284186 68598.34 51.71
4.66
64055.4
321475
5.56
68598.3
284186
Racemic with Rh2(R/S-MegaBNP)2
AU

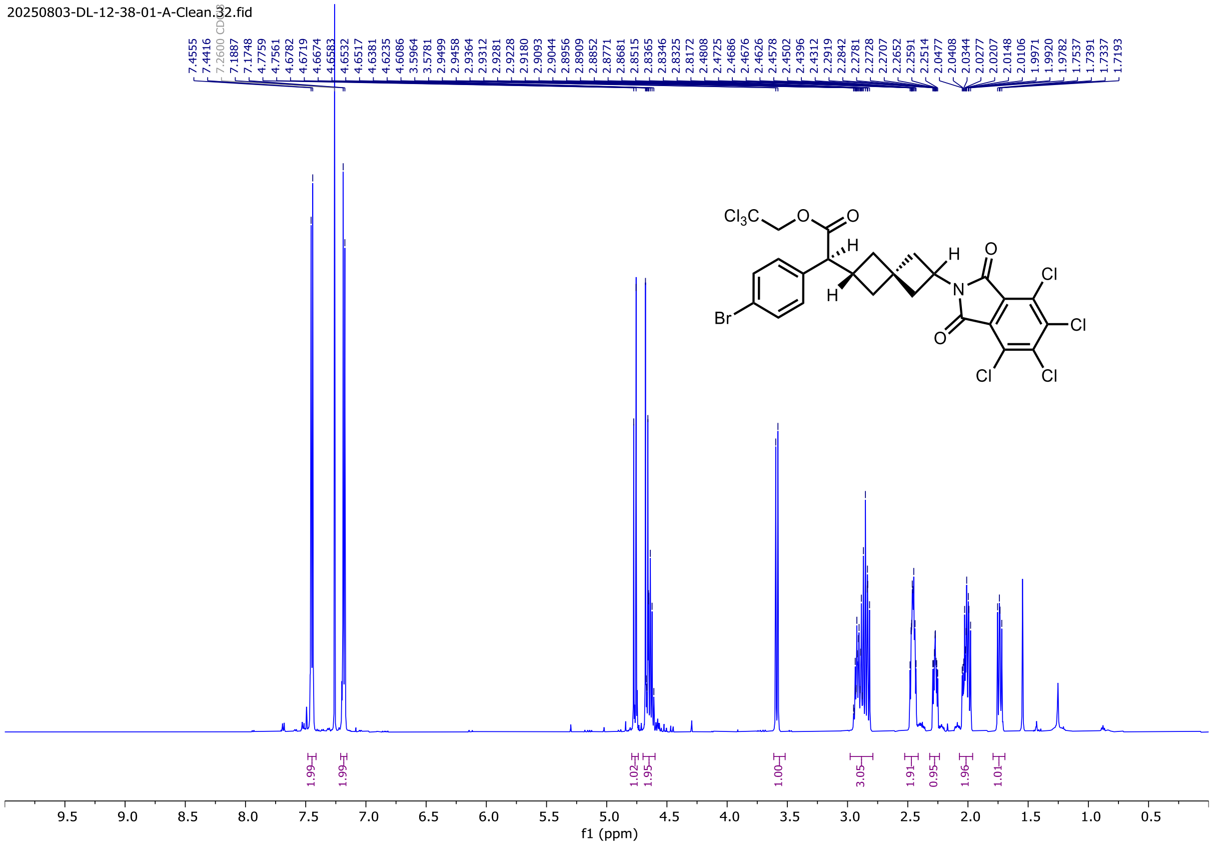
20250803-DL-12-38-01-A-Clean.32.fid
7.4555
7.4416
7.2600 CDCl3
7.1887
7.1748
4.7759
4.7561
4.6782
4.6719
4.6674
4.6583
4.6532
4.6517
4.6381
4.6235
4.6086
3.5964
3.5781
2.9499
2.9458
2.9364
2.9312
2.9281
2.9228
2.9180
2.9093
2.9044
2.8956
2.8909
2.8852
2.8771
2.8681
2.8515
2.8365
2.8346
2.8325
2.8172
2.4808
2.4725
2.4686
2.4676
2.4626
2.4578
2.4502
2.4396
2.4312
2.2919
2.2842
2.2781
2.2728
2.2707
2.2652
2.2591
2.2514
2.0477
2.0408
2.0344
2.0277
2.0207
2.0148
2.0106
1.9971
1.9920
1.9782
1.7537
1.7391
1.7337
1.7193
1.99
1.99
1.02
1.95
1.00
3.05
1.91
0.95
1.96
1.01
f1 (ppm)
Cl_3C
O
O
H
H
H
N
O
O
Cl
Cl
Cl
Cl
Br

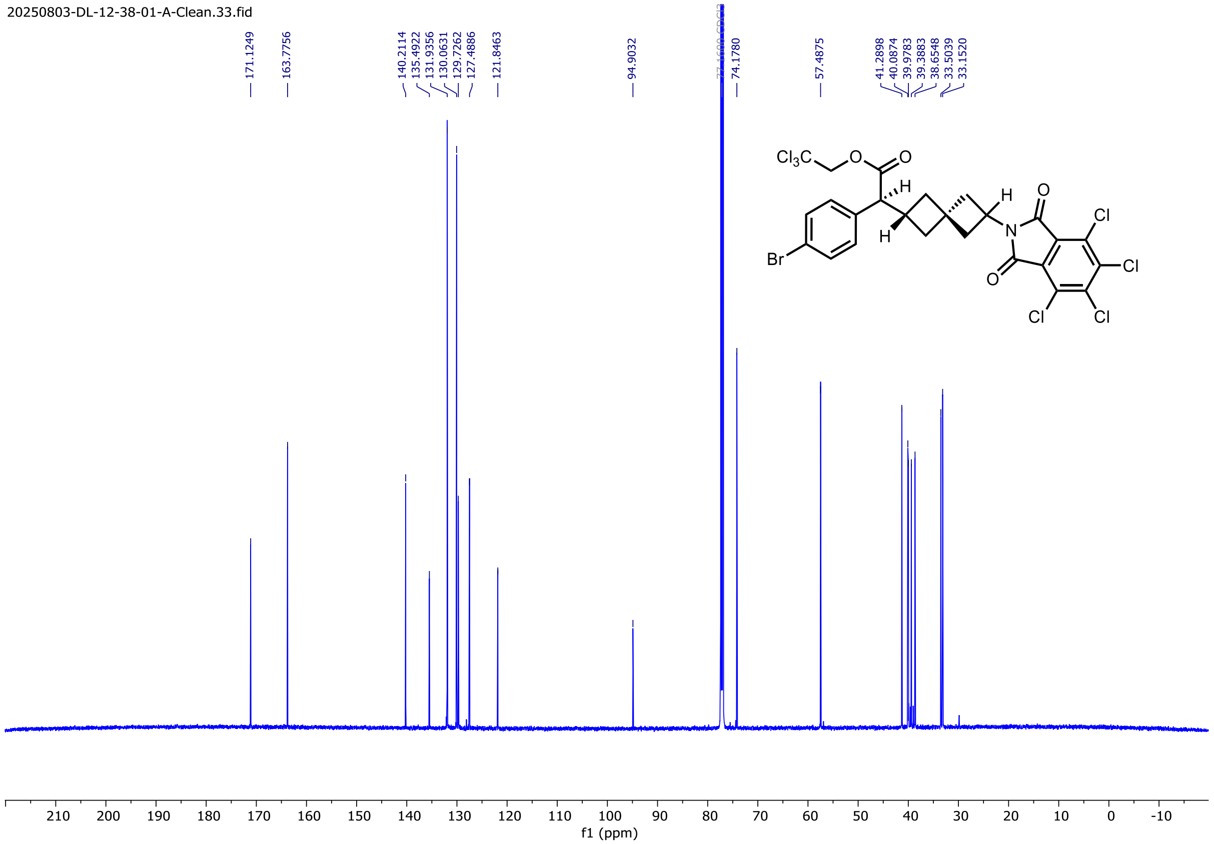
20250803-DL-12-38-01-A-Clean.33.fid
171.1249
163.7756
140.2114
135.4922
131.9356
130.0631
129.7262
127.4886
121.8463
94.9032
74.1780
57.4875
41.2898
40.0874
39.9783
39.3883
38.6548
33.5039
33.1520
f1 (ppm)
Cl_3C
O
O
H
H
H
N
O
O
Cl
Cl
Cl
Cl
Br

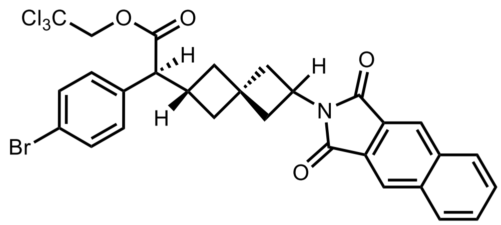

2,2,2-trichloroethyl (2S)-2-(4-bromophenyl)-2-(6-(1,3-dioxo-1,3-dihydro-2H-benzo[f]isoindol-2-yl)spiro[3.3]heptan-2-yl)acetate (Compound 42)

Prepared according to general procedure for C-H functionalization, 2-(spiro[3.3]heptan-2-yl)-1H-benzo[f]isoindole-1,3(2H)-dione (58.3 mg, 0.2 mmol, 1.0 equiv), Rh_2(*S*-MegaBNP)$_4$ (3.4 mg, 0.0001 mmol, 0.005 equiv), molecular sieve 4Å (100 wt%) and 2,2,2- HFIP (5 µL, 8.40 mg, 0.05 mmol, 0.25 equiv) in 0.5 ml CH_2Cl_2 were added a solution of trichloroethyl 2-(4-bromophenyl)-2-diazoacetate (149.0 mg, 0.4 mmol, 2.0 equiv) in 2.0 ml CH_2Cl_2 at 39℃ in 3 hours. The crude mixture was purified by flash chromatography (SiO_2, gradient 0%-25% Et_2O in hexane) afforded **compound 42** as a white amorphous solid (93.7 mg, 74% yield, 99% ee, 21:1 dr).

$\mathbf{R_f}$ (1Hex/2Et_2O) = 0.50 (CAM, UV 254 nm)

$\mathbf{[\alpha]^{20}_D}$**:** 20.7° (c = 0.64 g/100 ml, $CHCl_3$, 99% ee)

^{1}H NMR (600 MHz, $CDCl_3$) δ 8.28 (s, 2H), 8.03 (dd, *J* = 6.2, 3.3 Hz, 2H), 7.68 (dd, *J* = 6.2, 3.3 Hz, 2H), 7.46 (d, *J* = 8.3 Hz, 2H), 7.20 (d, *J* = 8.3 Hz, 2H), 4.78 (d, *J* = 12.0 Hz, 1H), 4.75 – 4.70 (m, 1H), 4.68 (d, *J* = 12.0 Hz, 1H), 3.61 (d, *J* = 11.0 Hz, 1H), 3.04 – 2.87 (m, 3H), 2.52 – 2.42 (m, 2H), 2.29 (ddd, *J* = 12.4, 8.2, 4.7 Hz, 1H), 2.11 – 1.97 (m, 2H), 1.76 (dd, *J* = 11.8, 8.7 Hz, 1H).

^{13}C NMR (151 MHz, $CDCl_3$) δ 171.2, 168.4, 135.6, 135.6, 131.9, 130.4, 130.1, 129.2, 127.8, 124.6, 121.8, 94.9, 74.2, 57.6, 40.8, 40.2, 40.1, 39.6, 38.6, 33.6, 33.2.

HRMS (+p APCI) calcd. for [$C_{29}H_{24}O_4N^{79}Br^{35}Cl_3$] ($[M+H]^+$) 633.9949 found 633.9967.

HPLC (Chiralpak ADH column, 5% isopropanol in hexane, 1.0 $mLmin^{-1}$, 1.0 $mgmL^{-1}$, 90 min, UV 230 nm) retention times of 58.7 min (minor) and 70.3 min (major), 99% ee.

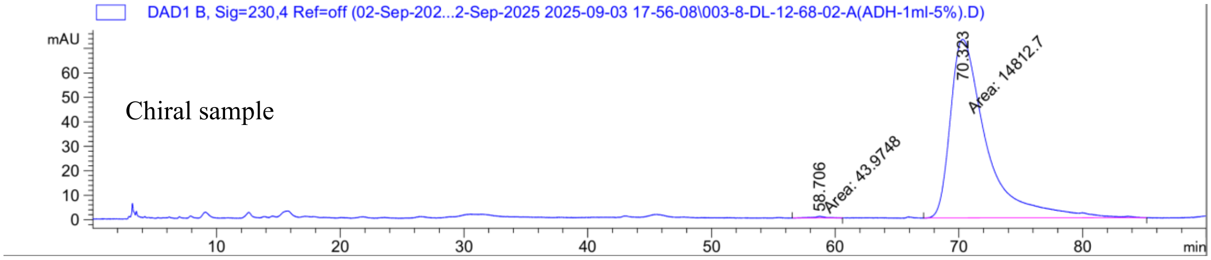

Signal 2: DAD1 B, Sig=230,4 Ref=off

Peak #	RetTime [min]	Type	Width [min]	Area [mAU*s]	Height [mAU]	Area %
1	58.706	MM	1.1117	43.97481	6.59257e-1	0.2960
2	70.323	MM	3.3796	1.48127e4	73.05009	99.7040

Totals : 1.48567e4 73.70935

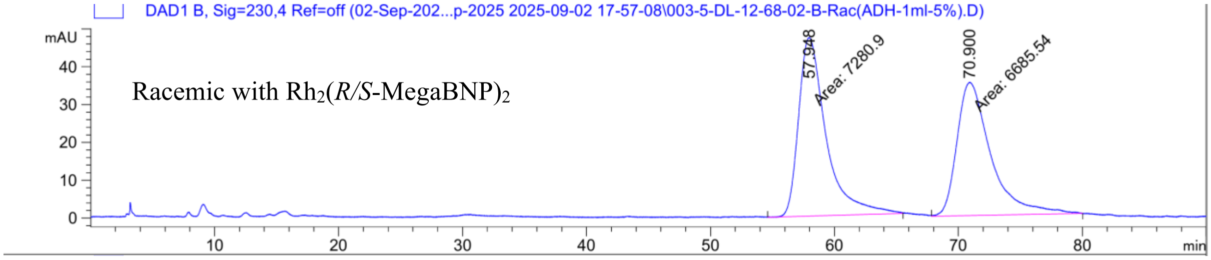

Signal 2: DAD1 B, Sig=230,4 Ref=off

Peak #	RetTime [min]	Type	Width [min]	Area [mAU*s]	Height [mAU]	Area %
1	57.948	MM	2.5708	7280.90479	47.20313	52.1314
2	70.900	MM	3.1606	6685.54150	35.25428	47.8686

Totals : 1.39664e4 82.45741

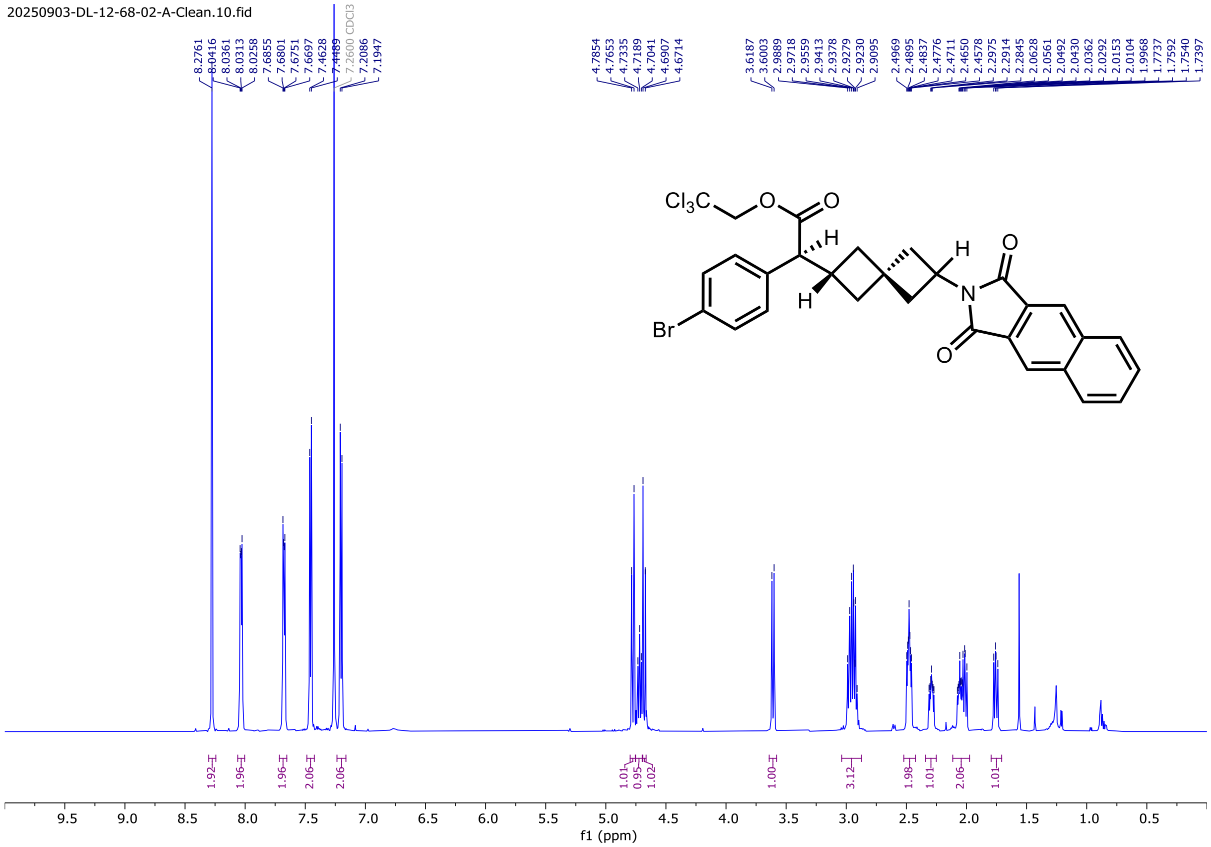
20250903-DL-12-68-02-A-Clean.10.fid
f1 (ppm)

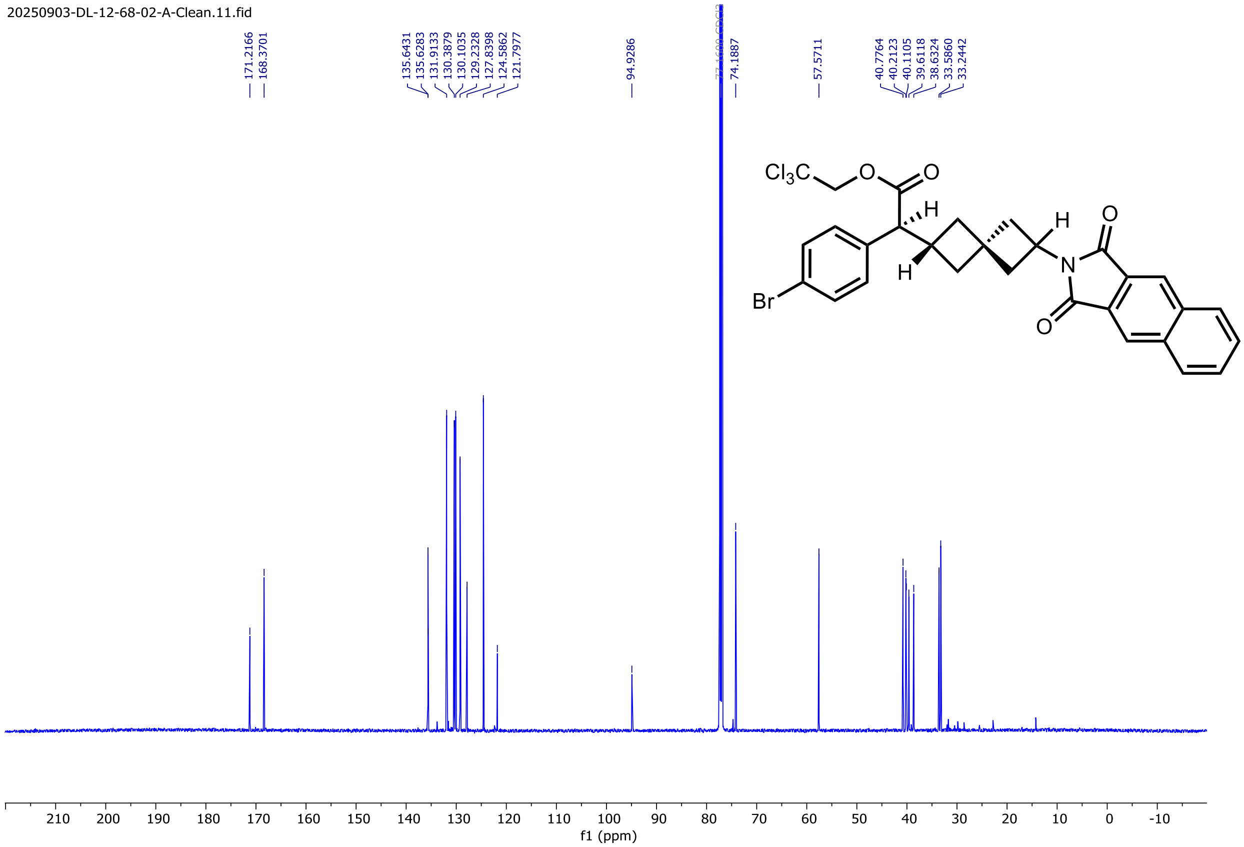
20250903-DL-12-68-02-A-Clean.11.fid
f1 (ppm)

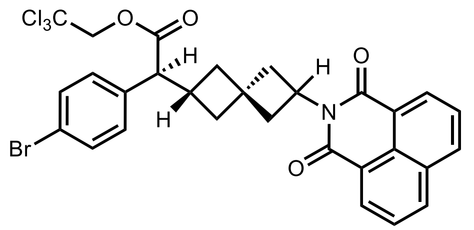

2,2,2-trichloroethyl (2S)-2-(4-bromophenyl)-2-(6-(1,3-dioxo-1H-benzo[de]isoquinolin-2(3H)-yl)spiro[3.3]heptan-2-yl)acetate (Compound 43)

Prepared according to general procedure for C-H functionalization, 2 2-(spiro[3.3]heptan-2-yl)-1H-benzo[de]isoquinoline-1,3(2H)-dione (58.3 mg, 0.2 mmol, 1.0 equiv), Rh_2(*S*-MegaBNP)$_4$ (3.4 mg, 0.0001 mmol, 0.005 equiv), molecular sieve 4Å (100 wt%) and 2,2,2- HFIP (5 µL, 8.40 mg, 0.05 mmol, 0.25 equiv) in 0.5 ml CH_2Cl_2 were added a solution of trichloroethyl 2-(4-bromophenyl)-2-diazoacetate (149.0 mg, 0.4 mmol, 2.0 equiv) in 2.0 ml CH_2Cl_2 at 39℃ in 3 hours. The crude mixture was purified by flash chromatography (SiO_2, gradient 0%-25% Et_2O in hexane) afforded **compound 43** as a white amorphous solid (85.7 mg, 67% yield, 99% ee, 8:1 dr).

R_f (1Hex/2Et_2O) = 0.50 (CAM, UV 254 nm)

$[\alpha]^{20}_D$: 20.9° (c = 1.11 g/100 ml, $CHCl_3$, 99% ee)

^{1}H NMR (600 MHz, $CDCl_3$) δ 8.54 (d, *J* = 6.9 Hz, 2H), 8.18 (d, *J* = 8.2 Hz, 2H), 7.73 (t, *J* = 7.7 Hz, 2H), 7.45 (d, *J* = 8.4 Hz, 2H), 7.21 (d, *J* = 8.4 Hz, 2H), 5.32 (p, *J* = 8.9 Hz, 1H), 4.77 (d, *J* = 11.9 Hz, 1H), 4.69 (d, *J* = 11.8 Hz, 1H), 3.63 (d, *J* = 11.1 Hz, 1H), 2.98 – 2.88 (m, 3H), 2.60 (ddd, *J* = 11.6, 8.3, 4.2 Hz, 1H), 2.50 (ddd, *J* = 11.5, 7.7, 4.1 Hz, 1H), 2.43 (ddd, *J* = 11.9, 8.3, 4.3 Hz, 1H), 2.08 (td, *J* = 7.8, 3.9 Hz, 1H), 2.03 (dd, *J* = 11.3, 8.3 Hz, 1H), 1.76 (dd, *J* = 11.7, 8.5 Hz, 1H).

^{13}C NMR (151 MHz, $CDCl_3$) δ 171.3, 164.9, 135.8, 133.8, 131.9, 131.6, 131.1, 130.1, 128.3, 127.1, 123.3, 121.7, 94.9, 74.2, 57.6, 43.6, 41.0, 40.6, 40.5, 39.2, 33.7, 33.4.

HRMS (+p APCI) calcd. for [$C_{29}H_{24}O_4N^{79}Br^{35}Cl_3$] ([M+H]$^+$) 633.9949 found 633.9964.

HPLC (Chiralpak ADH column, 10% isopropanol in hexane, 1.0 mLmin^{-1}, 1.0 mgmL^{-1}, 60 min, UV 230 nm) retention times of 36.9 min (major) and 45.6 min (minor), 99% ee.

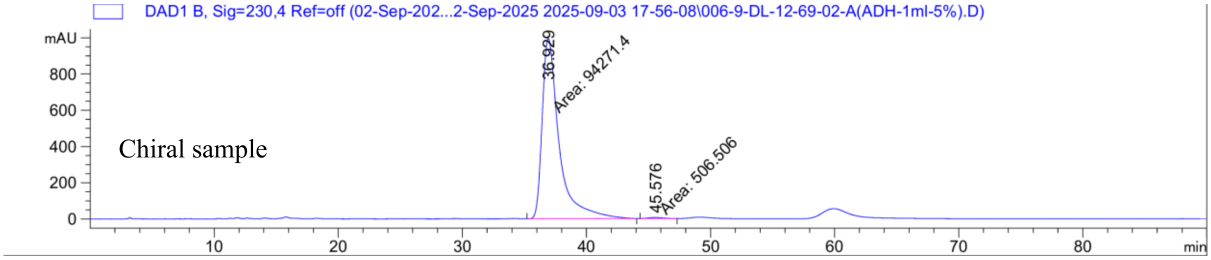

Signal 2: DAD1 B, Sig=230,4 Ref=off

Peak #	RetTime [min]	Type	Width [min]	Area [mAU*s]	Height [mAU]	Area %
1	36.929	MM	1.5791	9.42714e4	995.01147	99.4656
2	45.576	MM	1.4904	506.50562	5.66397	0.5344

Totals : 9.47779e4 1000.67544

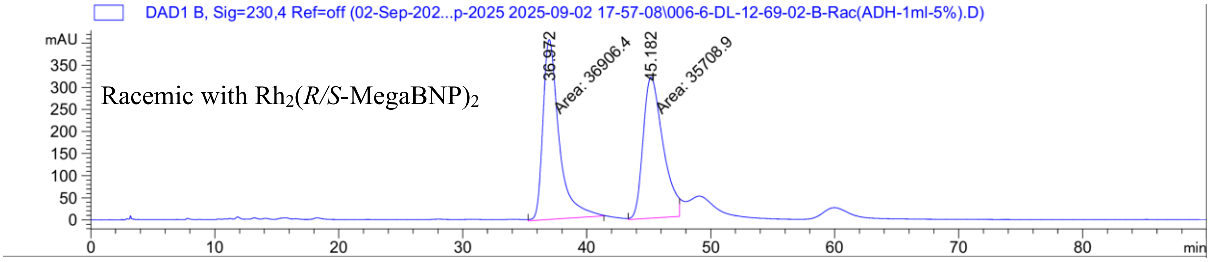

Signal 2: DAD1 B, Sig=230,4 Ref=off

Peak #	RetTime [min]	Type	Width [min]	Area [mAU*s]	Height [mAU]	Area %
1	36.972	MM	1.5104	3.69064e4	407.24084	50.8245
2	45.182	MM	1.8638	3.57089e4	319.32370	49.1755

Totals : 7.26153e4 726.56454

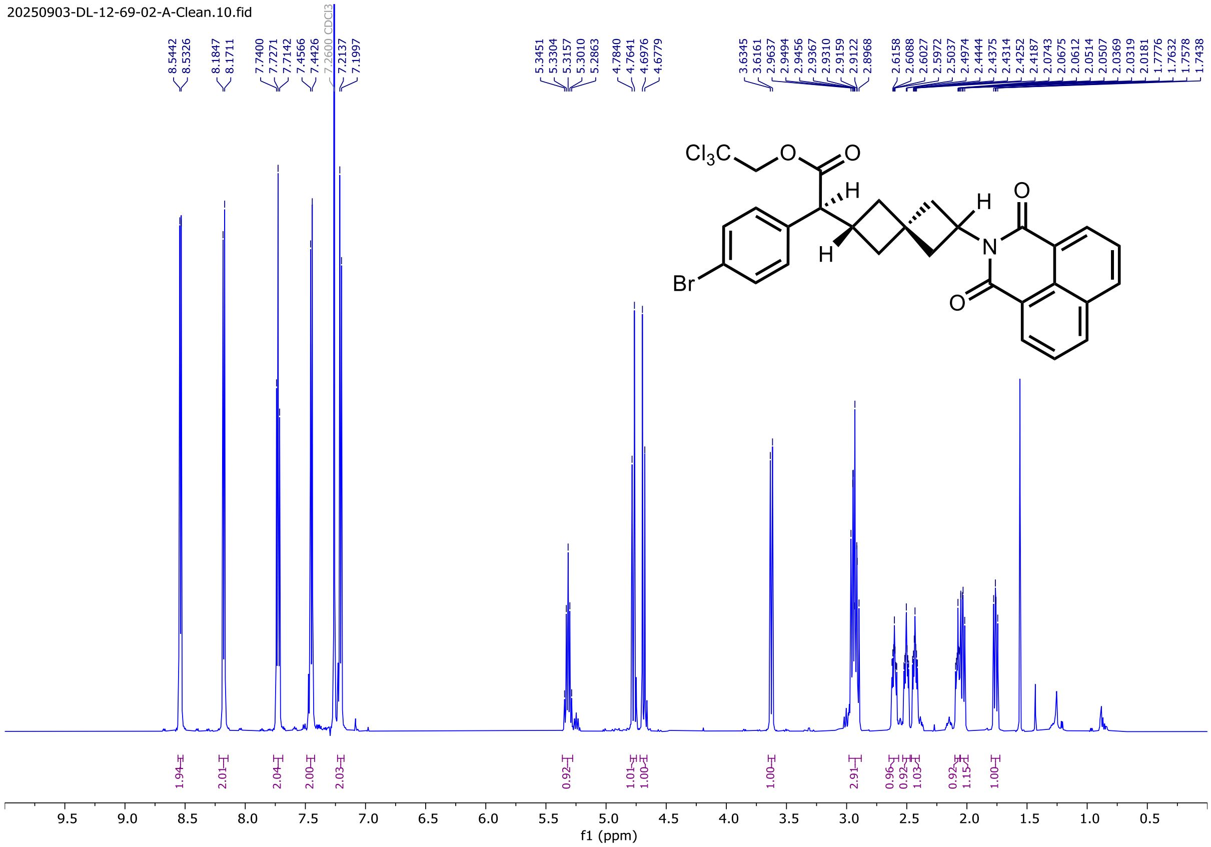
20250903-DL-12-69-02-A-Clean.10.fid
f1 (ppm)

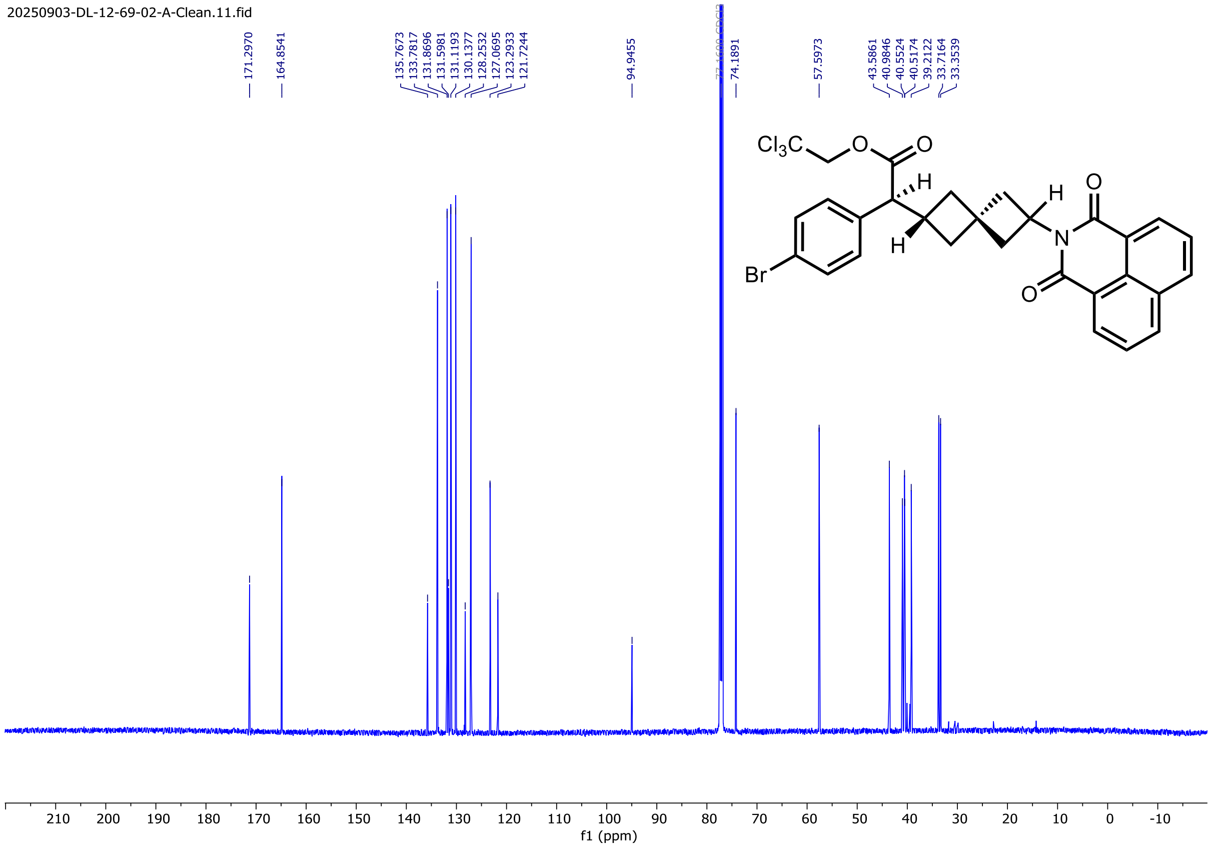
20250903-DL-12-69-02-A-Clean.11.fid
f1 (ppm)

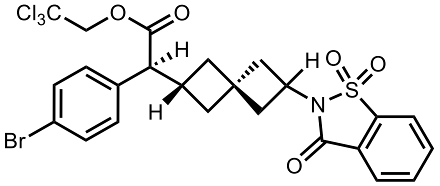

2,2,2-trichloroethyl (2S)-2-(4-bromophenyl)-2-(6-(1,1-dioxido-3-oxobenzo[d]isothiazol-2(3H)-yl)spiro[3.3]heptan-2-yl)acetate (Compound 44)

Prepared according to general procedure for C-H functionalization, 2-(spiro[3.3]heptan-2-yl)benzo[d]isothiazol-3(2H)-one 1,1-dioxide (55.5 mg, 0.2 mmol, 1.0 equiv), Rh_2(*S*-MegaBNP)$_4$ (3.4 mg, 0.0001 mmol, 0.005 equiv), molecular sieve 4Å (100 wt%) and 2,2,2- HFIP (5 µL, 8.40 mg, 0.05 mmol, 0.25 equiv) in 0.5 ml CH_2Cl_2 were added a solution of trichloroethyl 2-(4-bromophenyl)-2-diazoacetate (149.0 mg, 0.4 mmol, 2.0 equiv) in 2.0 ml CH_2Cl_2 at 39℃ in 3 hours. The crude mixture was purified by flash chromatography (SiO_2, gradient 0%-25% Et_2O in hexane) afforded **compound 44** as a white amorphous solid (87.3 mg, 70% yield, 97% ee (major) and 87% ee (minor), 1.6:1 dr).

R_f (1Hex/2Et_2O) = 0.50 (CAM, UV 254 nm)

$[\alpha]^{20}_D$: 28.1° (c = 0.39 g/100 ml, $CHCl_3$, 97% ee)

^{1}H NMR (400 MHz, $CDCl_3$) δ 7.87 (d, *J* = 7.5 Hz, 1H), 7.81 – 7.64 (m, 3H), 7.51 – 7.42 (m, 2H), 7.19 (d, *J* = 8.4 Hz, 2H), 5.35 – 5.19 (m, 1H), 4.80 – 4.73 (m, 1H), 4.71 – 4.63 (m, 1H), 3.59 (dd, *J* = 11.1, 1.7 Hz, 1H), 2.95 (dtd, *J* = 9.9, 8.2, 1.8 Hz, 1H), 2.84 – 2.67 (m, 1H), 2.65 – 2.51 (m, 1H), 2.45 – 2.21 (m, 3H), 2.09 – 1.90 (m, 2H), 1.73 (ddd, *J* = 11.6, 8.7, 3.0 Hz, 1H). (analyzed as a 1.5:1 mixture of 2 diastereomers)

^{13}C NMR (151 MHz, $CDCl_3$) δ 171.0, 171.0, 168.2, 143.6, 135.4, 135.3, 134.2, 133.5, 132.0, 132.0, 130.1, 130.0, 127.1, 123.4, 122.1, 122.0, 121.9, 94.9, 74.2, 74.2, 72.8, 72.7, 57.4, 57.4, 42.8, 42.8, 42.4, 42.3, 39.8, 39.7, 38.6, 38.6, 33.3, 33.2, 32.3, 32.3. (analyzed as a 1.5:1 mixture of 2 diastereomers)

HRMS (+p APCI) calcd. for [$C_{24}H_{22}O_5N^{79}Br^{35}Cl_3{}^{32}S$] ([M+H]$^+$) 619.9462 found 619.9477.

HPLC (Chiralpak ADH column, 10% isopropanol in hexane, 1.0 mLmin^{-1}, 1.0 mgmL^{-1}, 90 min, UV 230 nm). For major diastereomer retention times of 39.2 min (major) and 45.3 min (minor), 97% ee. For minor diastereomer retention times of 32.3 min (minor) and 63.9 min (major), 87% ee.

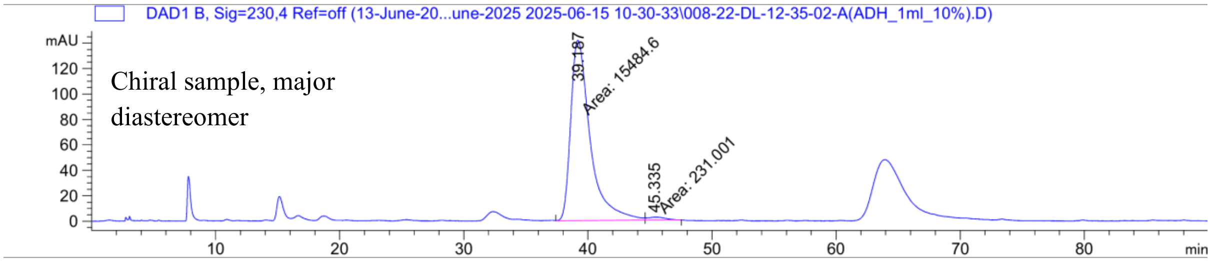

Signal 2: DAD1 B, Sig=230,4 Ref=off

Peak #	RetTime [min]	Type	Width [min]	Area [mAU*s]	Height [mAU]	Area %
1	39.187	MF	1.8209	1.54846e4	141.72704	98.5301
2	45.335	FM	1.6537	231.00150	2.32816	1.4699
Totals :				1.57156e4	144.05519	

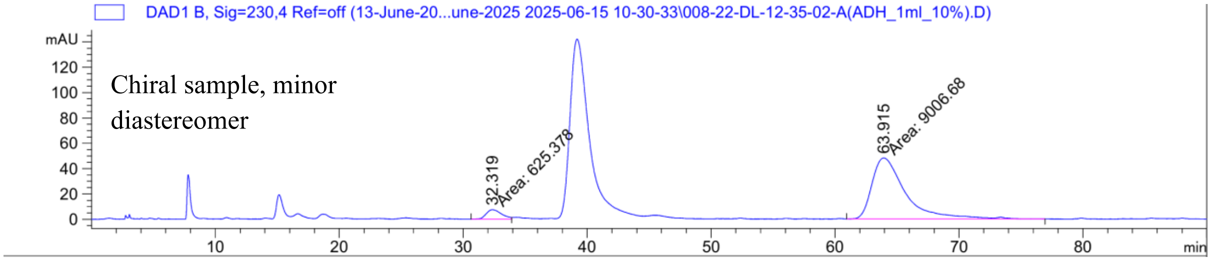

Signal 2: DAD1 B, Sig=230,4 Ref=off

Peak #	RetTime [min]	Type	Width [min]	Area [mAU*s]	Height [mAU]	Area %
1	32.319	MF	1.4165	625.37750	7.35813	6.4927
2	63.915	MM	3.1211	9006.68457	48.09571	93.5073
Totals :				9632.06207	55.45384	

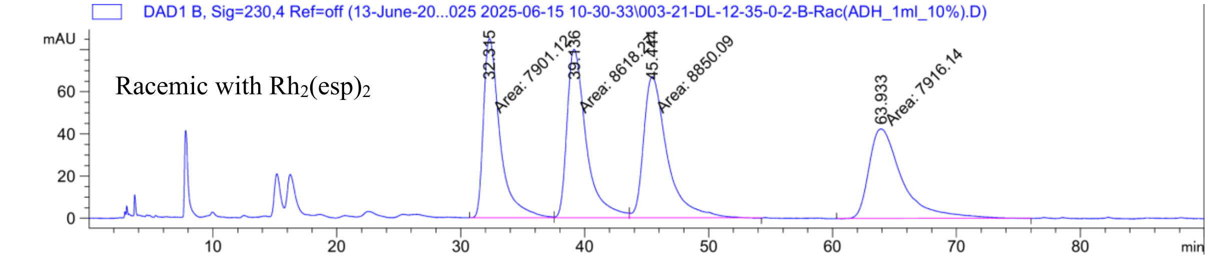

Signal 2: DAD1 B, Sig=230,4 Ref=off

Peak #	RetTime [min]	Type	Width [min]	Area [mAU*s]	Height [mAU]	Area %
1	32.315	MF	1.5510	7901.12158	84.90275	23.7373
2	39.136	FM	1.7978	8618.27344	79.89433	25.8919
3	45.444	FM	2.2044	8850.09180	66.91264	26.5883
4	63.933	MM	3.1078	7916.14111	42.45309	23.7825
Totals :				3.32856e4	274.16282	

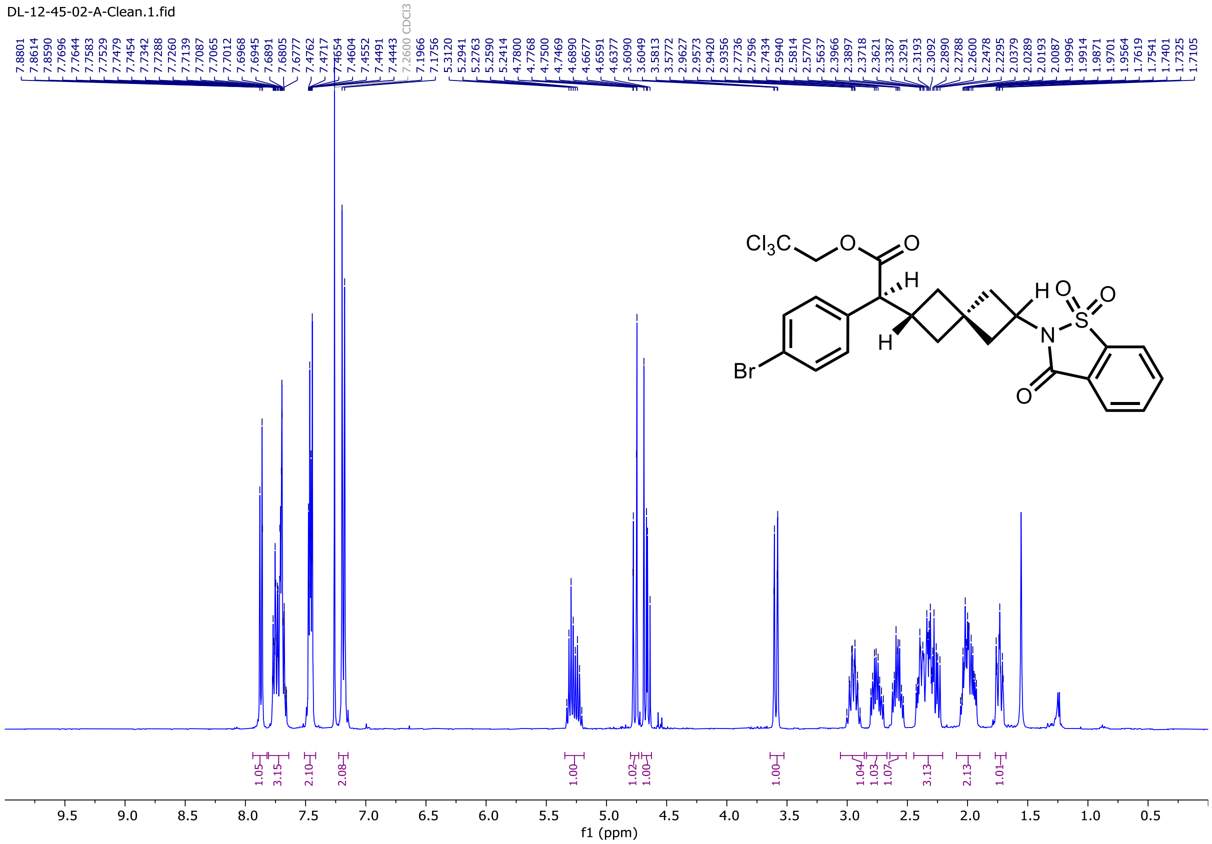
DL-12-45-02-A-Clean.1.fid
f1 (ppm)
Cl3C
O
H
Br
N
S

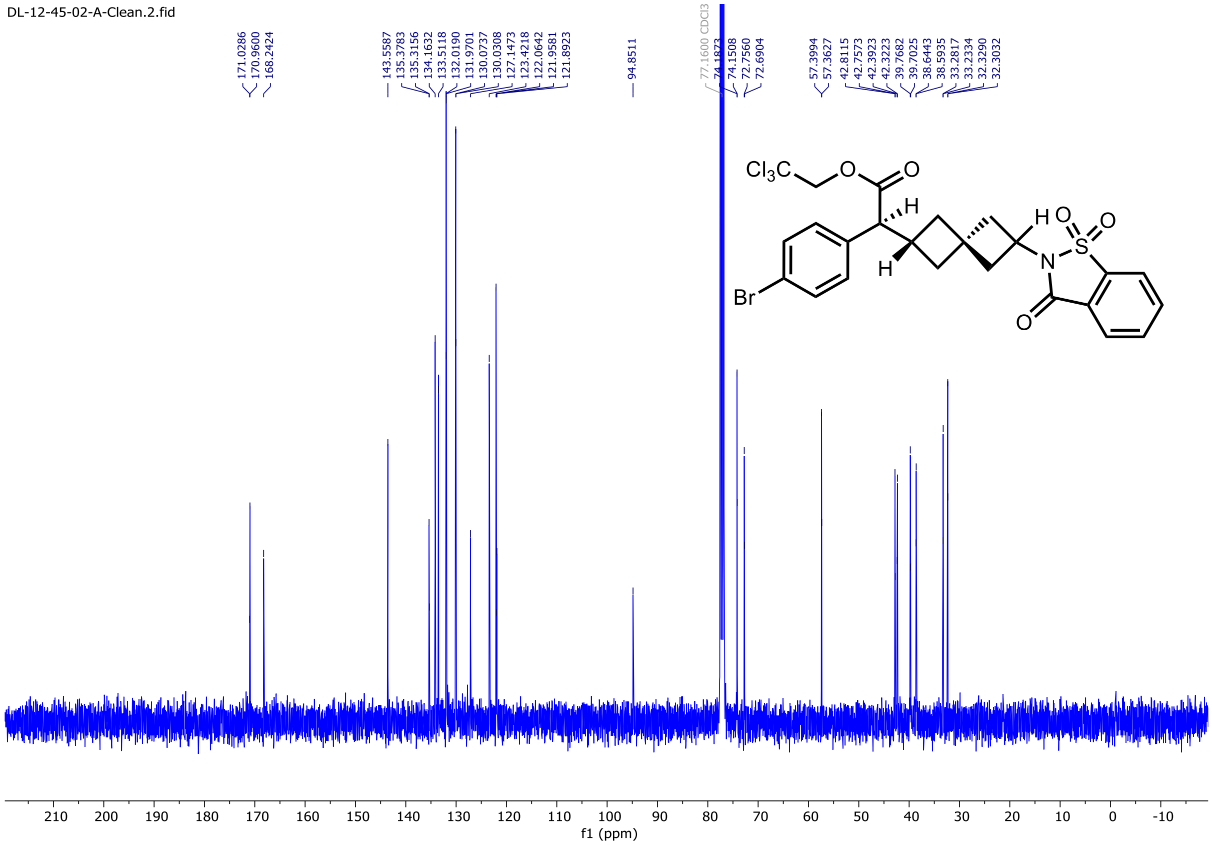
DL-12-45-02-A-Clean.2.fid
f1 (ppm)
Cl3C
O
H
Br
N
S

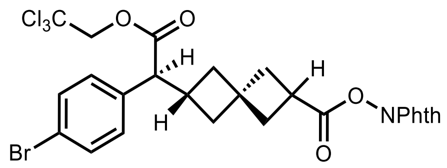

1,3-dioxoisoindolin-2-yl 6-((S)-1-(4-bromophenyl)-2-oxo-2-(2,2,2-trichloroethoxy)ethyl)spiro[3.3]heptane-2-carboxylate (Compound 45)

Prepared according to general procedure for C-H functionalization, 2-(spiro[3.3]heptan-2-yl)isoindoline-1,3-dione (48.3 mg, 0.2 mmol, 1.0 equiv), Rh_2(*S*-MegaBNP)$_4$ (3.4 mg, 0.0001 mmol, 0.005 equiv), molecular sieve 4Å (100 wt%) and 2,2,2- HFIP (5 μL, 8.40 mg, 0.05 mmol, 0.25 equiv) in 0.5 ml CH_2Cl_2 were added a solution of trichloroethyl 2-(4-bromophenyl)-2-diazoacetate (149.0 mg, 0.4 mmol, 2.0 equiv) in 2.0 ml CH_2Cl_2 at 39℃ in 3 hours. The crude mixture was purified by flash chromatography (SiO_2, gradient 0%-25% Et_2O in hexane) afforded **compound 45** as a white amorphous solid (92.9 mg, 74% yield, 98% ee, 9:1 dr).

R_f (1Hex/2Et_2O) = 0.50 (CAM, UV 254 nm)

$[\alpha]^{20}_D$: 16.8° (c = 0.86 g/100 ml, $CHCl_3$, 98% ee)

^{1}H NMR (600 MHz, $CDCl_3$) δ 7.87 (dd, *J* = 5.5, 3.1 Hz, 2H), 7.78 (dd, *J* = 5.5, 3.1 Hz, 2H), 7.44 (d, *J* = 8.3 Hz, 2H), 7.17 (d, *J* = 8.3 Hz, 2H), 4.76 (d, *J* = 12.0 Hz, 1H), 4.66 (d, *J* = 12.0 Hz, 1H), 3.56 (d, *J* = 11.0 Hz, 1H), 3.38 (p, *J* = 8.3 Hz, 1H), 2.88 (dp, *J* = 10.9, 8.2 Hz, 1H), 2.58 – 2.47 (m, 2H), 2.46 – 2.33 (m, 3H), 1.97 (td, *J* = 11.5, 6.1 Hz, 2H), 1.66 (dd, *J* = 12.0, 8.8 Hz, 1H).

^{13}C NMR (151 MHz, $CDCl_3$) δ 171.6, 171.1, 162.2, 135.4, 134.9, 132.0, 130.0, 129.1, 124.1, 121.9, 94.9, 74.2, 57.5, 40.3, 38.9, 38.4, 37.6, 36.7, 32.5, 30.6.

HRMS (+p APCI) calcd. for [$C_{26}H_{22}O_6N^{79}Br^{35}Cl_3$] ([M+H]$^+$) 627.9691 found 627.9706.

HPLC (Chiralpak ADH column, 5% isopropanol in hexane, 1.0 mLmin^{-1}, 1.0 mgmL^{-1}, 60 min, UV 230 nm) retention times of 36.0 min (minor) and 41.5 min (major), 98% ee.

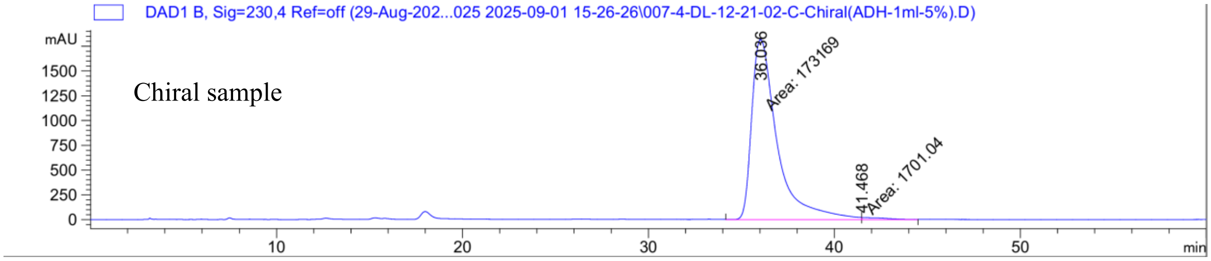

Signal 2: DAD1 B, Sig=230,4 Ref=off

Peak #	RetTime [min]	Type	Width [min]	Area [mAU*s]	Height [mAU]	Area %
1	36.036	MF	1.5871	1.73169e5	1818.55542	99.0273
2	41.468	FM	1.4418	1701.03906	19.66271	0.9727

Totals : 1.74870e5 1838.21813

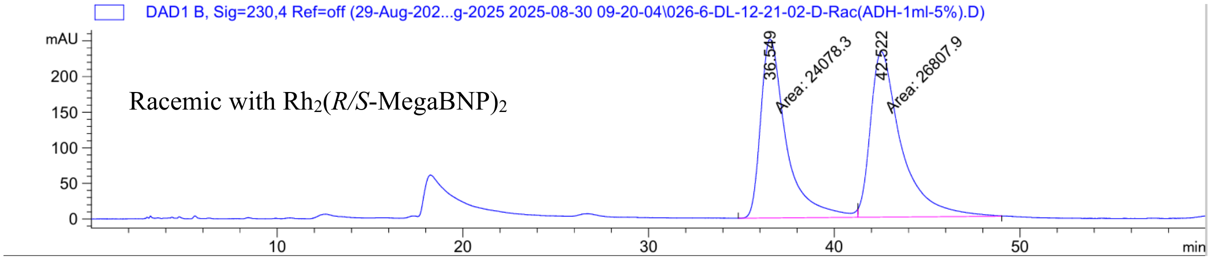

Signal 2: DAD1 B, Sig=230,4 Ref=off

Peak #	RetTime [min]	Type	Width [min]	Area [mAU*s]	Height [mAU]	Area %
1	36.549	MF	1.5994	2.40783e4	250.90726	47.3180
2	42.522	FM	1.9248	2.68079e4	232.13057	52.6820

Totals : 5.08862e4 483.03783

20250406-DL-12-21-02-A-Clean.10.fid

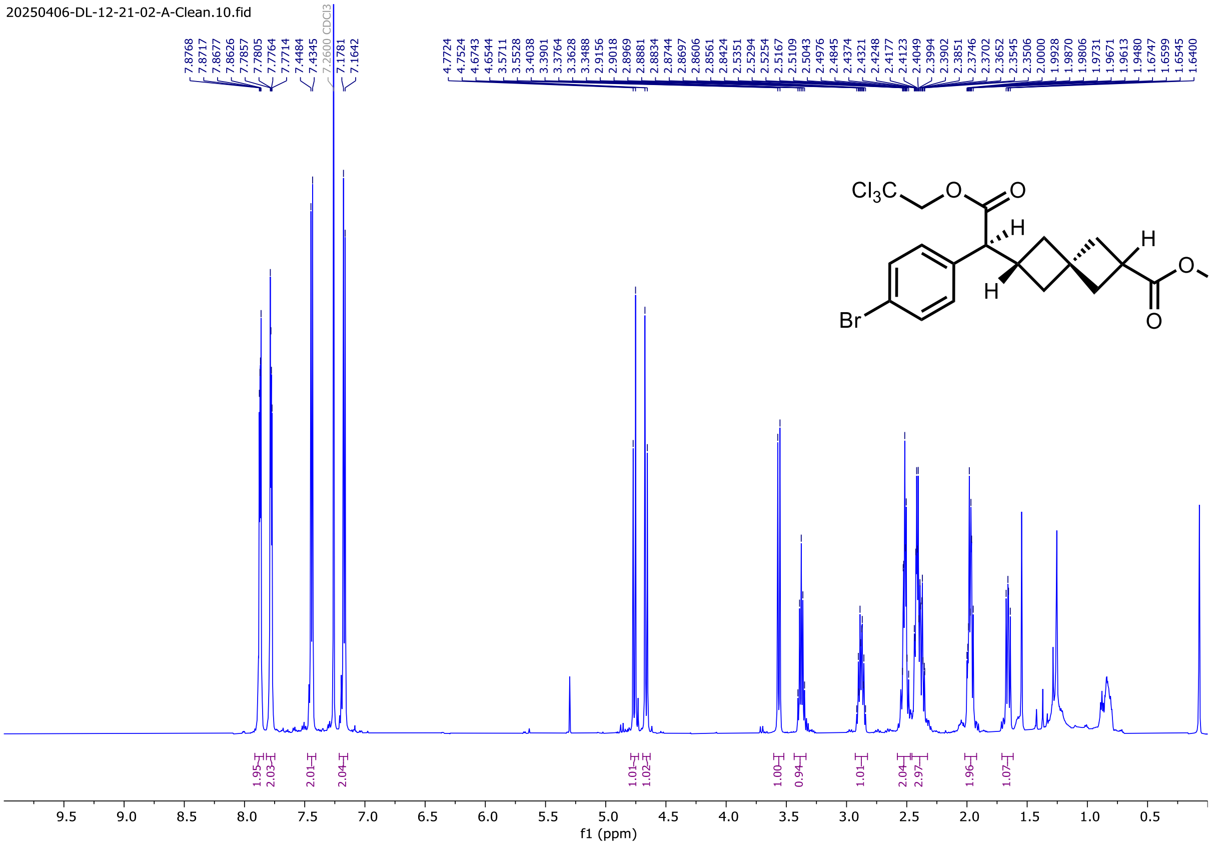

20250406-DL-12-21-02-A-Clean.12.fid

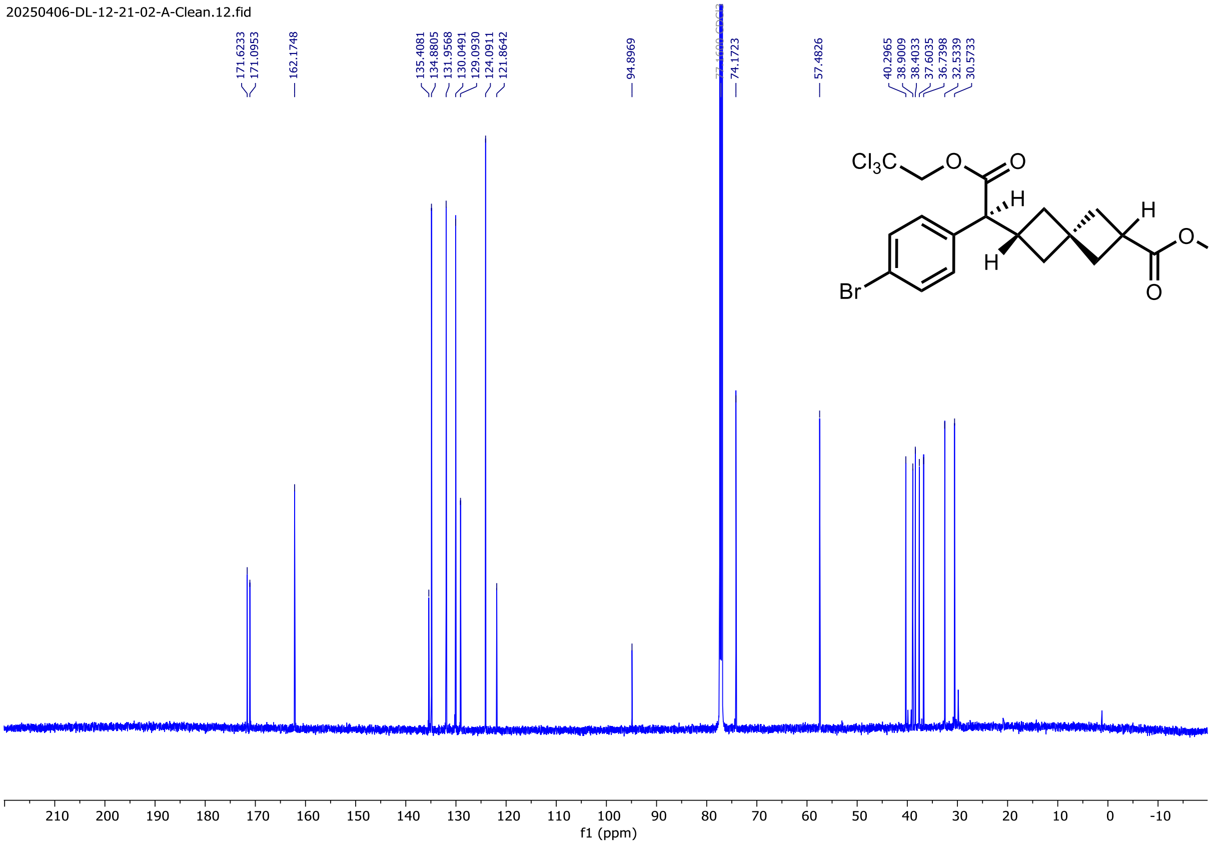

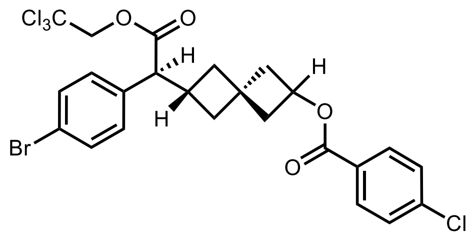

6-((S)-1-(4-bromophenyl)-2-oxo-2-(2,2,2-trichloroethoxy)ethyl)spiro[3.3]heptan-2-yl 4-chlorobenzoate (Compound 46)

Prepared according to general procedure for C-H functionalization, spiro[3.3]heptan-2-yl 4-chlorobenzoate (50.1 mg, 0.2 mmol, 1.0 equiv), Rh_2(*S*-MegaBNP)$_4$ (3.4 mg, 0.0001 mmol, 0.005 equiv), molecular sieve 4Å (100 wt%) and 2,2,2- HFIP (5 µL, 8.40 mg, 0.05 mmol, 0.25 equiv) in 0.5 ml CH_2Cl_2 were added a solution of trichloroethyl 2-(4-bromophenyl)-2-diazoacetate (149.0 mg, 0.4 mmol, 2.0 equiv) in 2.0 ml CH_2Cl_2 at 39℃ in 3 hours. The crude mixture was purified by flash chromatography (SiO_2, gradient 0%-8% Et_2O in hexane) afforded **compound 46** as white amorphous solids – major diastereomers (37.3 mg, 31% yield, 95% ee) and minor diastereomers (26.3 mg, 22% yield, 91% ee) (ratio of 2 diastereomer 1.4:1 dr)

Major diastereomer of compound 46

R_f (10Hex/1Et_2O) = 0.35 (CAM, UV 254 nm)

$[\alpha]^{20}_D$: 16.8° (c = 0.86 g/100 ml, $CHCl_3$, 95% ee)

^{1}H NMR (800 MHz, $CDCl_3$) δ 7.93 (d, *J* = 8.6 Hz, 2H), 7.45 (d, *J* = 8.4 Hz, 2H), 7.39 (d, *J* = 8.6 Hz, 2H), 7.18 (d, *J* = 8.4 Hz, 2H), 5.12 (p, *J* = 7.2 Hz, 1H), 4.76 (d, *J* = 12.0 Hz, 1H), 4.67 (d, *J* = 12.0 Hz, 1H), 3.59 (d, *J* = 11.0 Hz, 1H), 2.93 (dp, *J* = 11.1, 8.2 Hz, 1H), 2.65 (ddd, *J* = 11.9, 7.0, 5.0 Hz, 1H), 2.47 (ddd, *J* = 12.1, 7.1, 5.1 Hz, 1H), 2.39 (ddd, *J* = 11.8, 7.7, 4.2 Hz, 1H), 2.18 (dd, *J* = 11.6, 7.3 Hz, 1H), 2.13 (dd, *J* = 11.9, 7.4 Hz, 1H), 1.98 (dd, *J* = 11.5, 8.4 Hz, 1H), 1.93 (ddd, *J* = 12.0, 7.9, 4.2 Hz, 1H), 1.70 (dd, *J* = 11.8, 8.7 Hz, 1H).

^{13}C NMR (201 MHz, $CDCl_3$) δ 171.1, 165.3, 139.5, 135.5, 131.9, 131.1, 130.1, 128.8, 121.8, 94.9, 74.2, 66.2, 57.5, 43.0, 42.5, 40.0, 38.7, 33.3, 32.6.

HRMS (+p APCI) calcd. for [$C_{24}H_{20}O_4{}^{79}Br^{35}Cl_4$] ([M+H]$^+$) 590.9305 found 590.9295.

HPLC (Chiralpak ADH column, 2% isopropanol in hexane, 1.0 mLmin^{-1}, 1.0 mgmL^{-1}, 60 min, UV 230 nm) retention times of 16.7 min (major) and 19.8 min (minor), 95% ee.

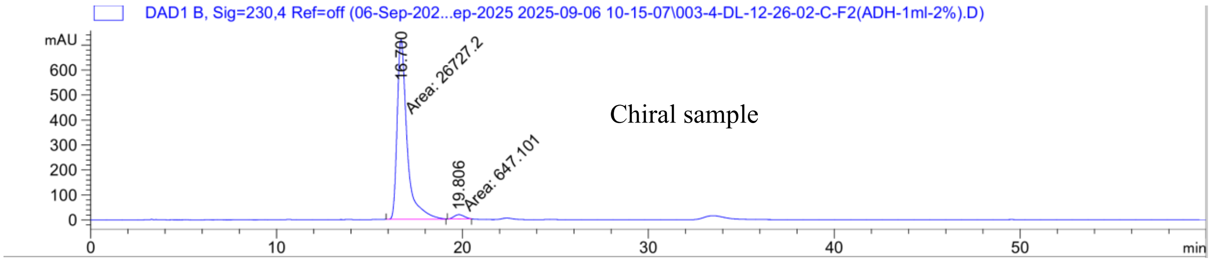

Chiral sample

Signal 2: DAD1 B, Sig=230,4 Ref=off

Peak #	RetTime [min]	Type	Width [min]	Area [mAU*s]	Height [mAU]	Area %
1	16.700	MM	0.6199	2.67272e4	718.56714	97.6361
2	19.806	MM	0.6261	647.10107	17.22665	2.3639
Totals :				2.73743e4	735.79379	

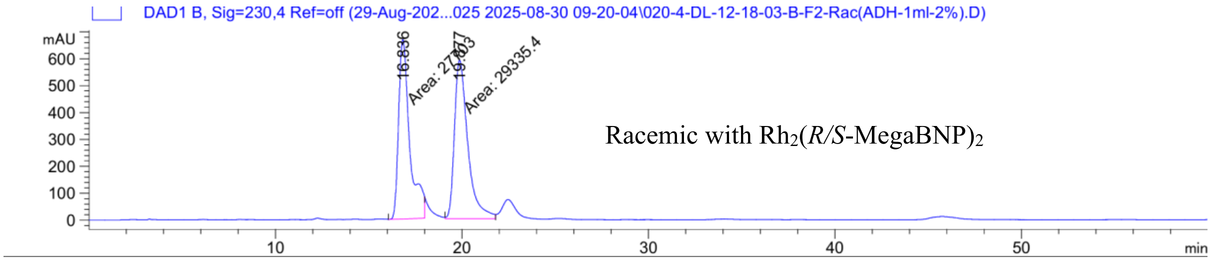

Racemic with Rh_2(*R/S*-MegaBNP)$_2$

Signal 2: DAD1 B, Sig=230,4 Ref=off

Peak #	RetTime [min]	Type	Width [min]	Area [mAU*s]	Height [mAU]	Area %
1	16.836	MM	0.6922	2.77030e4	667.01904	48.5691
2	19.877	FM	0.8296	2.93354e4	589.33508	51.4309
Totals :				5.70384e4	1256.35413	

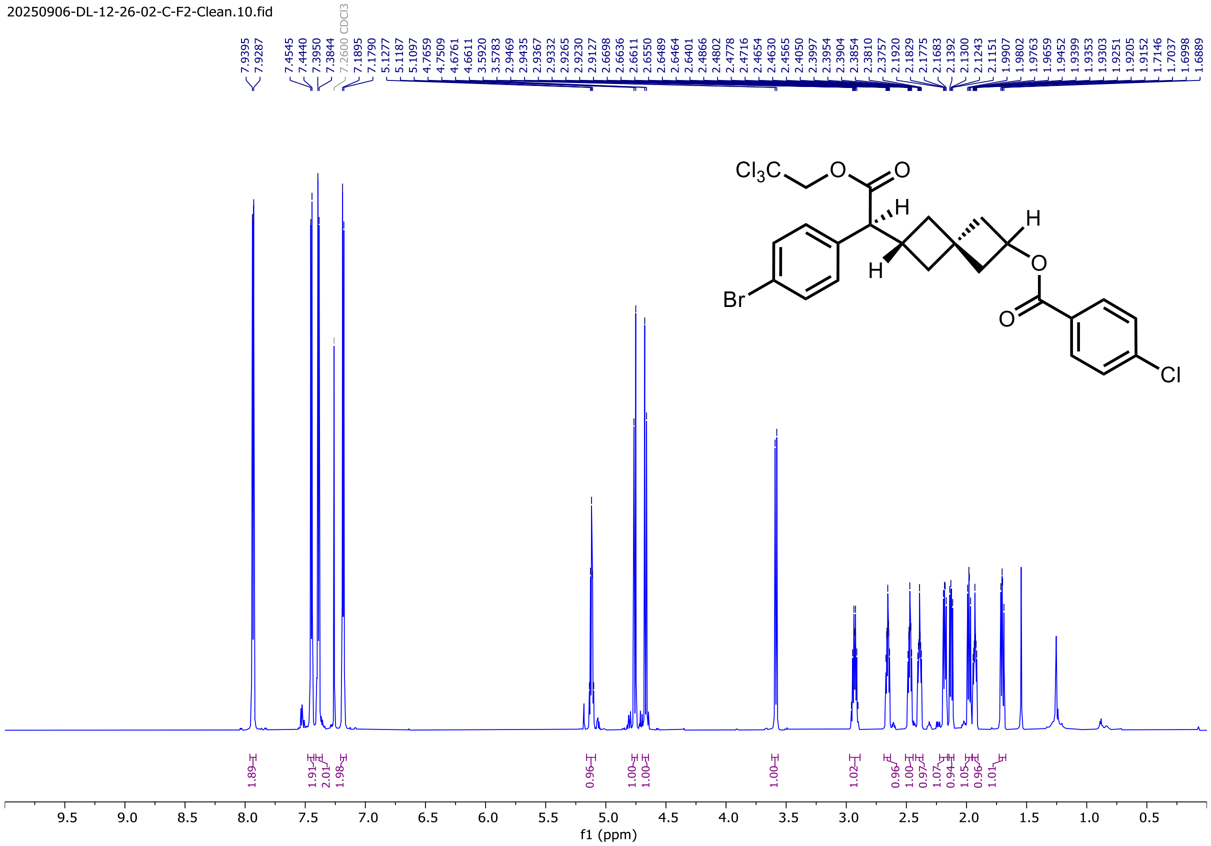

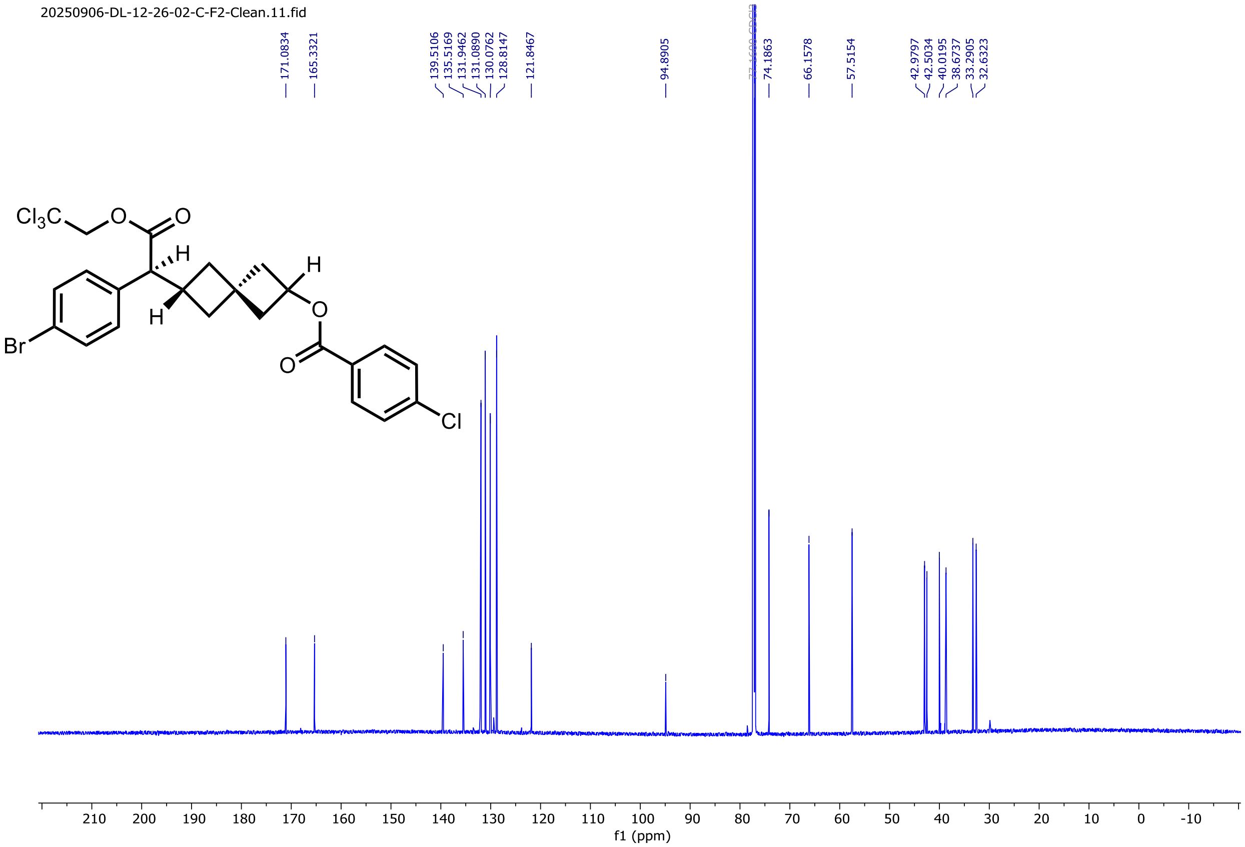

Minor diastereomer of compound 46

R$_f$ (10Hex/1Et_2O) = 0.40 (CAM, UV 254 nm)

[α]$^{20}_{D}$: 14.8° (c = 0.25 g/100 ml, $CHCl_3$, 91% ee)

^{1}H NMR (800 MHz, $CDCl_3$) δ 7.94 (d, J = 8.3 Hz, 2H), 7.46 (d, J = 8.1 Hz, 2H), 7.40 (d, J = 8.3 Hz, 2H), 7.19 (d, J = 8.1 Hz, 2H), 5.07 (p, J = 7.2 Hz, 1H), 4.76 (d, J = 12.0 Hz, 1H), 4.65 (d, J = 12.0 Hz, 1H), 3.59 (d, J = 11.0 Hz, 1H), 2.97 – 2.88 (m, 1H), 2.61 (dt, J = 12.0, 6.0 Hz, 1H), 2.44 (dt, J = 12.0, 6.0 Hz, 1H), 2.31 (ddd, J = 11.9, 7.7, 4.2 Hz, 1H), 2.24 (dd, J = 11.8, 7.4 Hz, 1H), 2.18 (dd, J = 12.0, 7.4 Hz, 1H), 2.02 (ddd, J = 11.8, 7.8, 4.1 Hz, 1H), 1.98 (dd, J = 11.8, 8.4 Hz, 1H), 1.71 (dd, J = 11.5, 8.6 Hz, 1H).

^{13}C NMR (201 MHz, $CDCl_3$) δ 171.1, 165.3, 139.5, 135.5, 132.0, 131.1, 130.1, 128.8, 121.9, 94.9, 74.2, 66.1, 57.5, 42.9, 42.6, 39.8, 38.9, 33.3, 32.6.

HRMS (+p APCI) calcd. for [$C_{24}H_{20}O_4{}^{79}Br^{35}Cl_4$] ([M+H]$^+$) 590.9305 found 590.9298.

HPLC (Chiralpak ADH column, 3% isopropanol in hexane, 1.0 mLmin^{-1}, 1.0 mgmL^{-1}, 60 min, UV 230 nm) retention times of 17.7 min (minor) and 24.0 min (major), 91% ee.

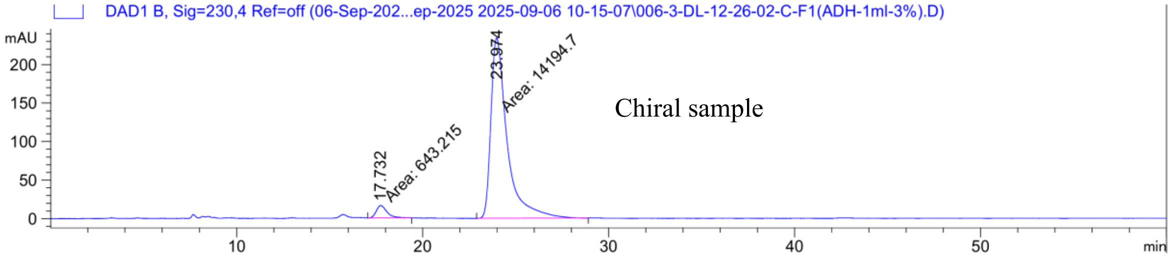

Signal 2: DAD1 B, Sig=230,4 Ref=off

Peak #	RetTime [min]	Type	Width [min]	Area [mAU*s]	Height [mAU]	Area %
1	17.732	MM	0.6628	643.21521	16.17501	4.3350
2	23.974	MM	1.0115	1.41947e4	233.88301	95.6650
Totals :				1.48379e4	250.05802	

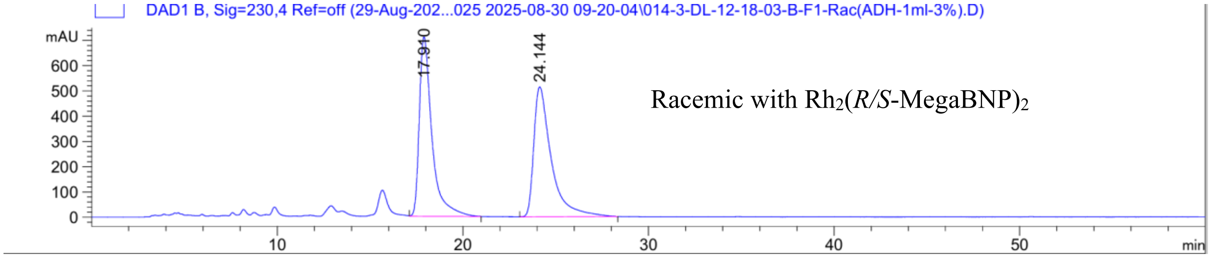

Signal 2: DAD1 B, Sig=230,4 Ref=off

Peak #	RetTime [min]	Type	Width [min]	Area [mAU*s]	Height [mAU]	Area %
1	17.910	VV R	0.5540	3.36021e4	710.24908	49.9746
2	24.144	VV R	0.7677	3.36363e4	514.49182	50.0254
Totals :				6.72384e4	1224.74091	

20250906-DL-12-26-02-C-F1-Clean.10.fid

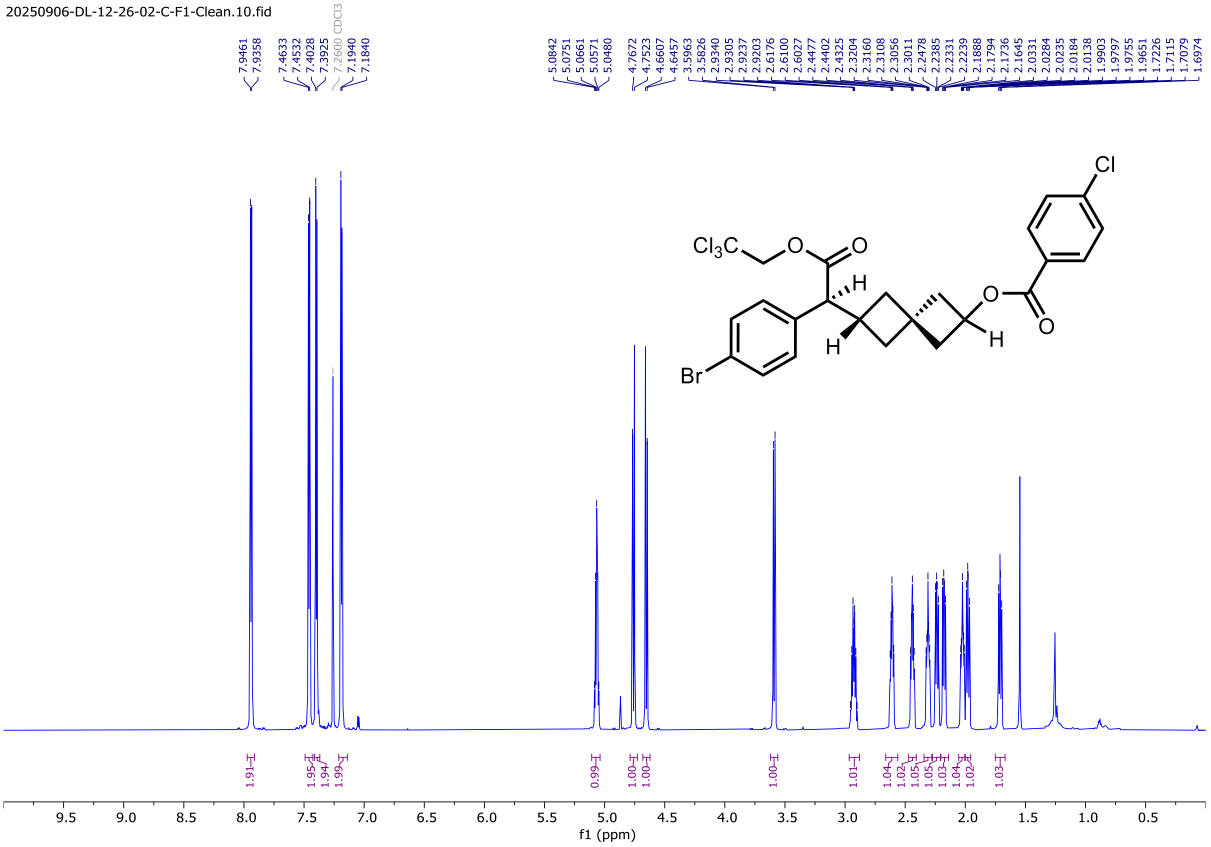

20250906-DL-12-26-02-C-F1-Clean.11.fid

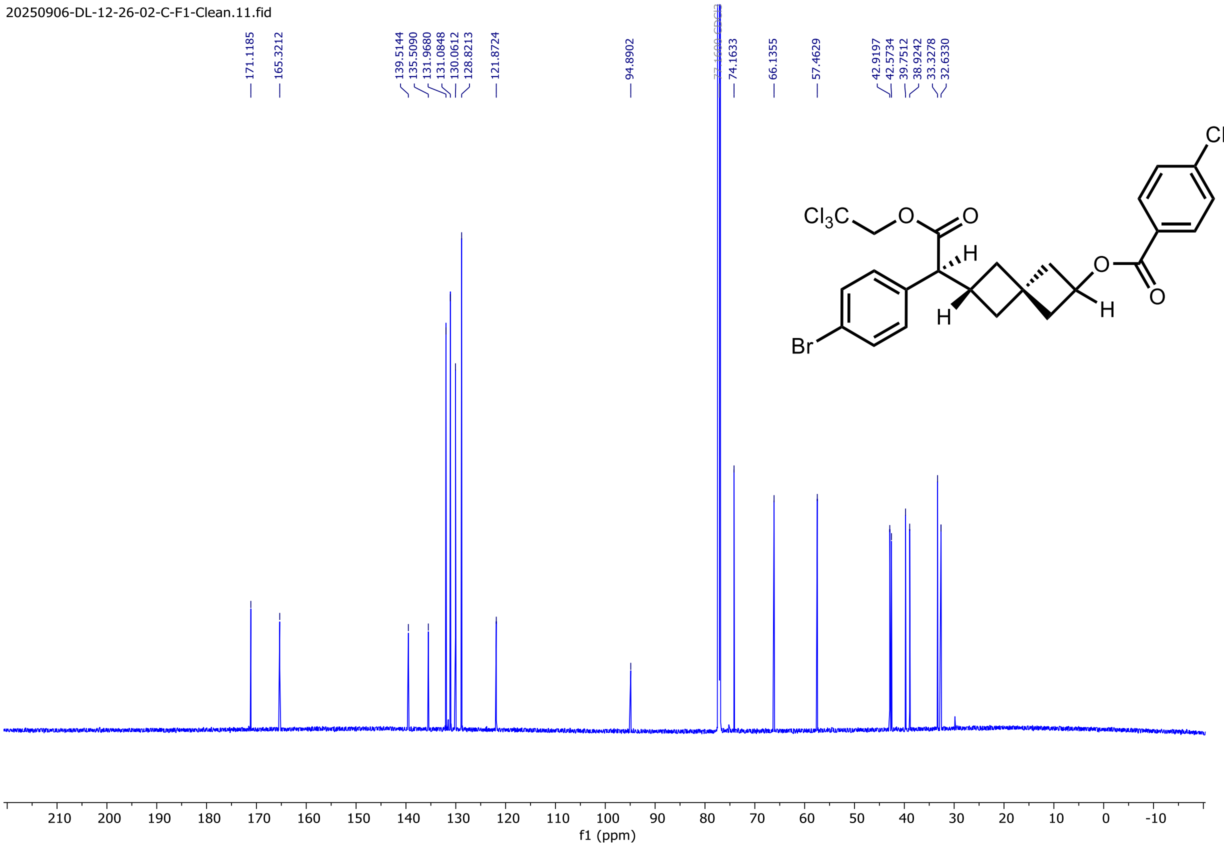

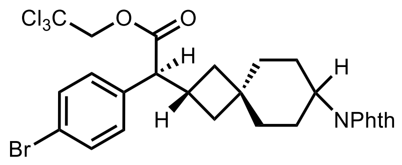

2,2,2-trichloroethyl (2S)-2-(4-bromophenyl)-2-(7-(1,3-dioxoisoindolin-2-yl)spiro[3.5]nonan-2-yl)acetate (Compound 47)

Prepared according to general procedure for C-H functionalization, 2-(spiro[3.5]nonan-7-yl)isoindoline-1,3-dione (53.9 mg, 0.2 mmol, 1.0 equiv), Rh_2(*S*-MegaBNP)$_4$ (3.4 mg, 0.0001 mmol, 0.005 equiv), molecular sieve 4Å (100 wt%) and 2,2,2- HFIP (5 µL, 8.40 mg, 0.05 mmol, 0.25 equiv) in 0.5 ml CH_2Cl_2 were added a solution of trichloroethyl 2-(4-bromophenyl)-2-diazoacetate (149.0 mg, 0.4 mmol, 2.0 equiv) in 2.0 ml CH_2Cl_2 at 39℃ in 3 hours. The crude mixture was purified by flash chromatography (SiO_2, gradient 0%-25% Et_2O in hexane) afforded **compound 47** as a white amorphous solid (96.3 mg, 78% yield, 99% ee, 20:1 dr).

R_f (1Hex/2Et_2O) = 0.50 (CAM, UV 254 nm)

$[\alpha]^{20}_D$: 25.5° (c = 0.55 g/100 ml, $CHCl_3$, 99% ee)

^{1}H NMR (600 MHz, $CDCl_3$) δ 7.78 (dd, *J* = 5.5, 3.0 Hz, 2H), 7.67 (dd, *J* = 5.5, 3.0 Hz, 2H), 7.47 (d, *J* = 8.4 Hz, 2H), 7.22 (d, *J* = 8.4 Hz, 2H), 4.76 (d, *J* = 12.0 Hz, 1H), 4.67 (d, *J* = 12.0 Hz, 1H), 4.04 (tt, *J* = 12.4, 4.0 Hz, 1H), 3.60 (d, *J* = 11.2 Hz, 1H), 2.95 (dp, *J* = 11.2, 8.5 Hz, 1H), 2.22 (qd, *J* = 12.6, 3.5 Hz, 1H), 2.19 – 2.10 (m, 1H), 2.04 (ddd, *J* = 11.6, 8.2, 3.6 Hz, 1H), 1.98 (ddd, *J* = 12.4, 8.6, 3.8 Hz, 1H), 1.94 (dd, *J* = 13.4, 3.1 Hz, 1H), 1.72 – 1.63 (m, 2H), 1.58 (dt, *J* = 12.8, 3.0 Hz, 1H), 1.55 – 1.46 (m, 2H), 1.46 – 1.36 (m, 2H).

^{13}C NMR (151 MHz, $CDCl_3$) δ 171.3, 168.5, 135.7, 133.9, 132.2, 131.9, 130.1, 123.1, 121.8, 94.9, 74.2, 58.4, 50.3, 39.8, 38.9, 36.0, 35.4, 34.6, 31.9, 26.0, 25.9.

HRMS (+p APCI) calcd. for $[C_{27}H_{26}O_4N^{79}Br^{35}Cl_3]$ ($[M+H]^+$) 612.0105 found 612.0122.

HPLC (ASH column, 5% isopropanol in hexane, 1.0 $mLmin^{-1}$, 1.0 $mgmL^{-1}$, 60 min, UV 230 nm) retention times of 13.3 min (minor) and 16.1 min (major), 99% ee.

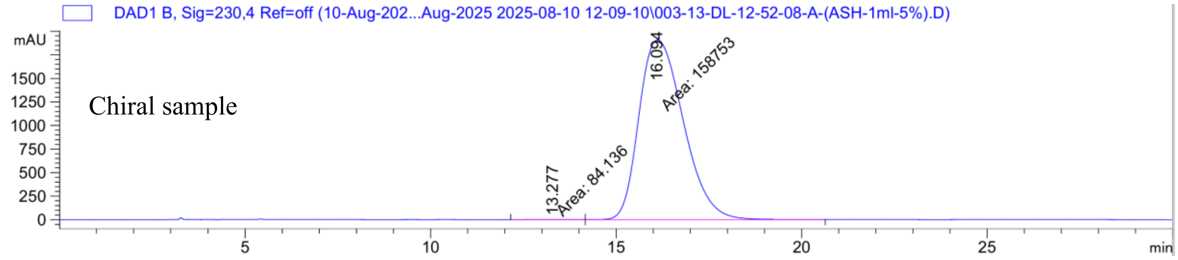

Signal 2: DAD1 B, Sig=230,4 Ref=off

Peak #	RetTime [min]	Type	Width [min]	Area [mAU*s]	Height [mAU]	Area %
1	13.277	MF	1.0061	84.13602	1.39372	0.0530
2	16.094	FM	1.3865	1.58753e5	1908.31396	99.9470

Totals : 1.58837e5 1909.70769

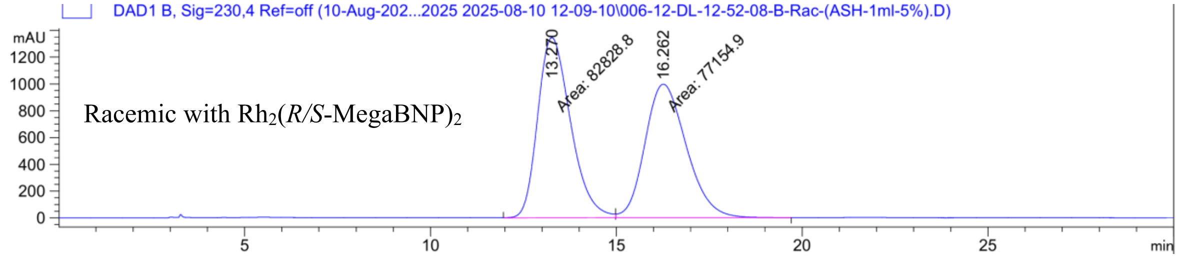

Signal 2: DAD1 B, Sig=230,4 Ref=off

Peak #	RetTime [min]	Type	Width [min]	Area [mAU*s]	Height [mAU]	Area %
1	13.270	MF	1.0259	8.28288e4	1345.68298	51.7733
2	16.262	FM	1.2889	7.71549e4	997.64624	48.2267

Totals : 1.59984e5 2343.32922

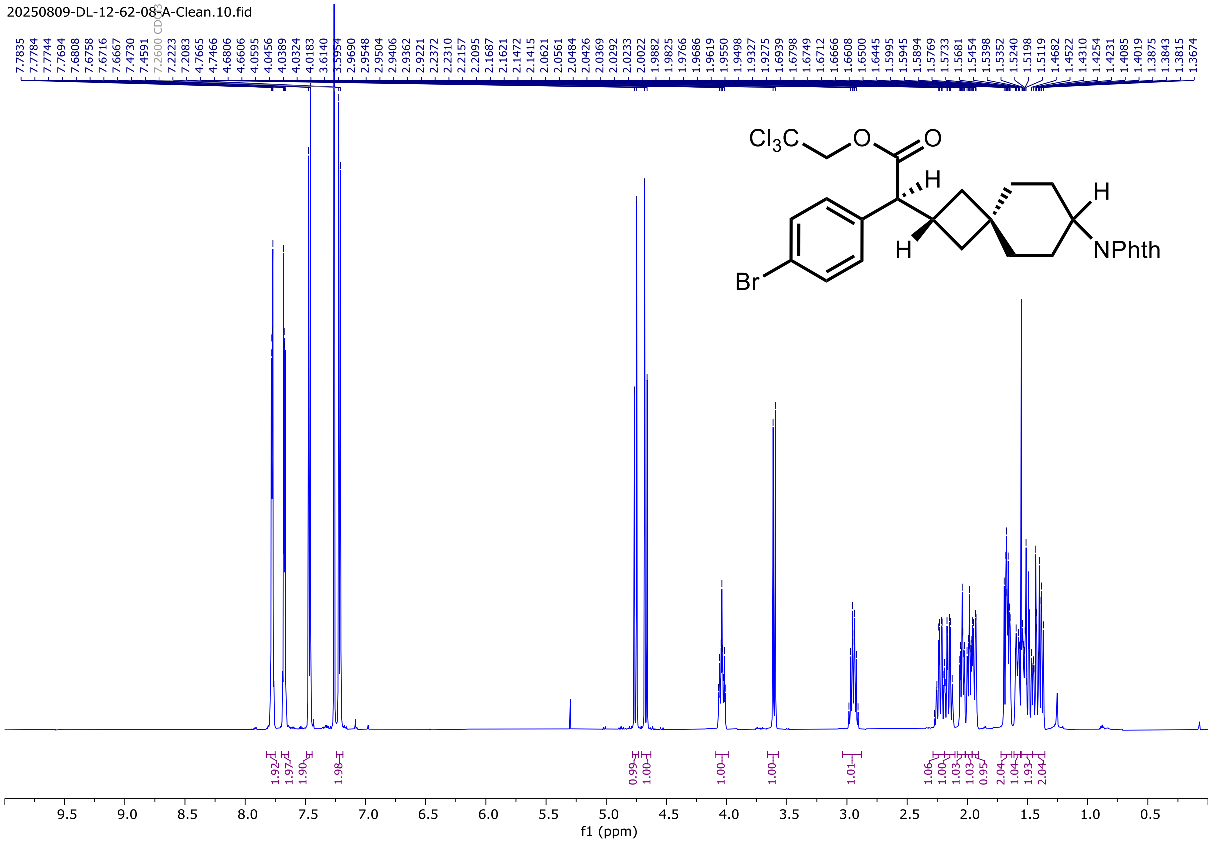
20250809-DL-12-62-08-A-Clean.10.fid
7.7835 7.7784 7.7744 7.7694 7.6808 7.6758 7.6716 7.6667 7.4730 7.4591 7.2600 CDCl3 7.2223 7.2083 4.7665 4.7466 4.6806 4.6606 4.0595 4.0456 4.0389 4.0324 4.0183 3.6140 3.5994 2.9690 2.9548 2.9504 2.9406 2.9362 2.9221 2.2372 2.2310 2.2157 2.2095 2.1687 2.1621 2.1472 2.1415 2.0621 2.0561 2.0484 2.0426 2.0369 2.0292 2.0233 2.0022 1.9882 1.9825 1.9766 1.9686 1.9619 1.9550 1.9498 1.9327 1.9275 1.6939 1.6798 1.6749 1.6712 1.6666 1.6608 1.6500 1.6445 1.5995 1.5945 1.5894 1.5769 1.5733 1.5681 1.5454 1.5398 1.5352 1.5240 1.5198 1.5119 1.4682 1.4522 1.4310 1.4254 1.4231 1.4085 1.4019 1.3875 1.3843 1.3815 1.3674
Cl3C
O
O
H
H
H
Br
NPhth
1.92 1.97 1.90 1.98 0.99 1.00 1.00 1.00 1.01 1.06 1.00 1.03 1.03 0.95 2.04 1.04 1.93 2.04
f1 (ppm)

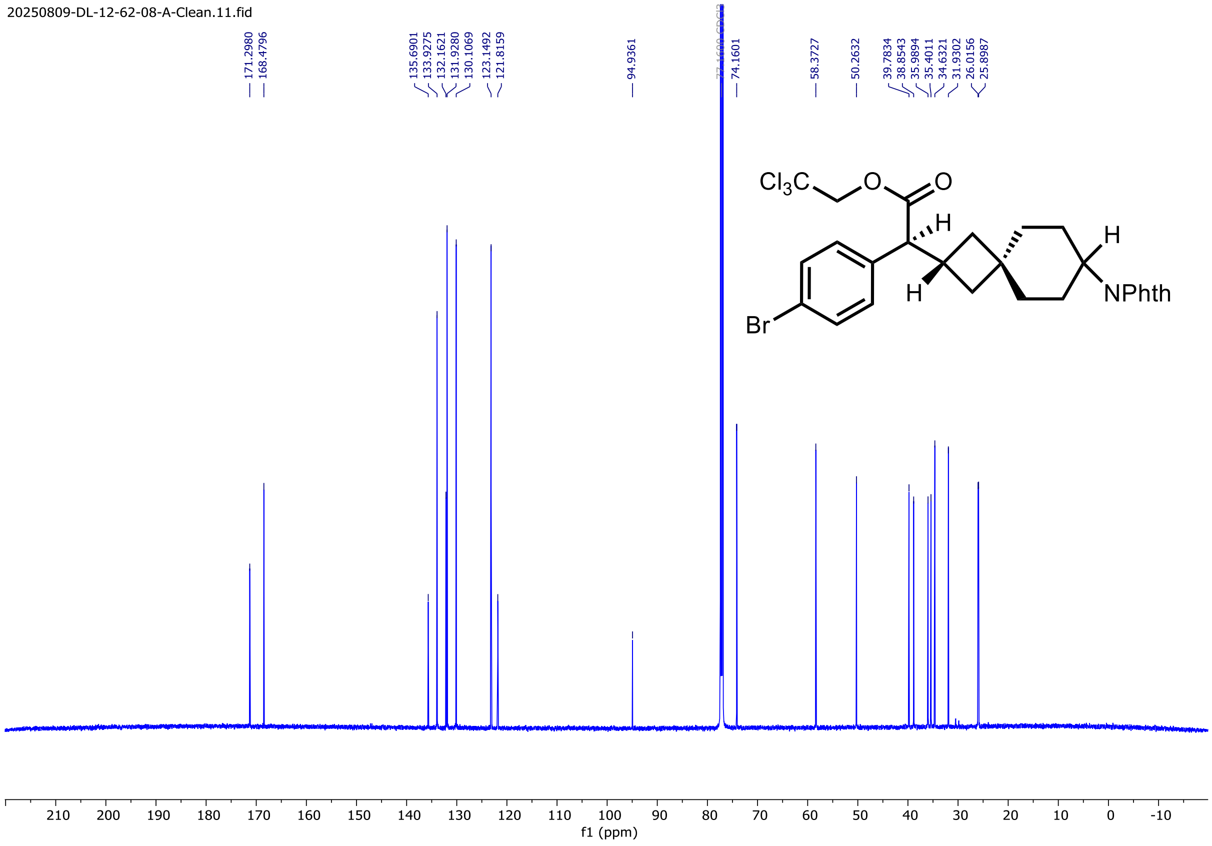
20250809-DL-12-62-08-A-Clean.11.fid
171.2980 168.4796 135.6901 133.9275 132.1621 131.9280 130.1069 123.1492 121.8159 94.9361 77.1600 CDCl3 74.1601 58.3727 50.2632 39.7834 38.8543 35.9894 35.4011 34.6321 31.9302 26.0156 25.8987
Cl3C
O
O
H
H
H
Br
NPhth
f1 (ppm)

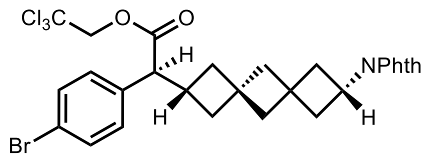

2,2,2-trichloroethyl (2S)-2-(4-bromophenyl)-2-(8-(1,3-dioxoisoindolin-2-yl)dispiro[3.1.36.14]decan-2-yl)acetate (Compound 48)

Prepared according to general procedure for C-H functionalization, 2-(dispiro[3.1.36.14]decan-2-yl)isoindoline-1,3-dione (56.3 mg, 0.2 mmol, 1.0 equiv), Rh_2(*S*-MegaBNP)$_4$ (3.4 mg, 0.0001 mmol, 0.005 equiv), molecular sieve 4Å (100 wt%) and 2,2,2- HFIP (5 µL, 8.40 mg, 0.05 mmol, 0.25 equiv) in 0.5 ml CH_2Cl_2 were added a solution of trichloroethyl 2-(4-bromophenyl)-2-diazoacetate (149.0 mg, 0.4 mmol, 2.0 equiv) in 2.0 ml CH_2Cl_2 at 39℃ in 3 hours. The crude mixture was purified by flash chromatography (SiO_2, gradient 0%-25% Et_2O in hexane) afforded **compound 48** as a white amorphous solid (73.9 mg, 59% yield, 95% ee, 2:1 dr). The major diastereomer was tentatively assigned as a cis isomer. The ratio of 2 diastereomers was determined by ^{13}C-NMR.

R$_f$ (1Hex/2Et_2O) = 0.50 (CAM, UV 254 nm)

$[\alpha]^{20}_D$: 15.0° (c = 0.68 g/100 ml, $CHCl_3$, 95% ee)

^{1}H NMR (600 MHz, $CDCl_3$) δ 7.79 (dd, *J* = 5.5, 3.0 Hz, 2H), 7.68 (dd, *J* = 5.5, 3.0 Hz, 2H), 7.44 (d, *J* = 8.2 Hz, 2H), 7.18 (d, *J* = 8.2 Hz, 2H), 4.74 (d, *J* = 12.0 Hz, 1H), 4.65 (d, *J* = 12.0 Hz, 1H), 4.63 – 4.53 (m, 1H), 3.56 (d, *J* = 11.0 Hz, 1H), 2.94 – 2.82 (m, 3H), 2.31 – 2.17 (m, 4H), 2.17 – 2.12 (m, 1H), 2.10 – 2.06 (m, 1H), 2.03 (td, *J* = 11.2, 2.3 Hz, 1H), 1.94 – 1.83 (m, 2H), 1.60 (dd, *J* = 11.6, 8.8 Hz, 1H). (analyzed as a mixture of 2 diastereomers)

^{13}C NMR (151 MHz, $CDCl_3$) δ 171.3, 171.3, 168.7, 135.8, 135.8, 134.0, 132.1, 131.9, 130.1, 123.2, 121.7, 94.9, 74.1, 57.6, 57.6, 47.7, 47.2, 46.8, 40.8, 40.7, 40.4, 40.4, 40.1, 40.1, 40.0, 40.0, 39.5, 39.4, 35.3, 35.2, 33.2, 33.1, 33.0. (analyzed as a mixture of 2 diastereomers)

HRMS (+p APCI) calcd. for [$C_{28}H_{26}O_4N^{79}Br^{35}Cl_3$] ([M+H]$^+$) 624.0105 found 624.0120.

SFC (AMY1, 30% (50% methanol in isopropanol with 0.2% Formic Acid) in CO_2, 1.5 mL/min, 1.0 mg/ml, UV 230 nm) retention times of 5.12 min (major) and 13.34 min (minor) 95% ee for major diastereomer and retention times of 6.83 min (minor) and 16.58 min (major) 95% ee for minor diastereomer.

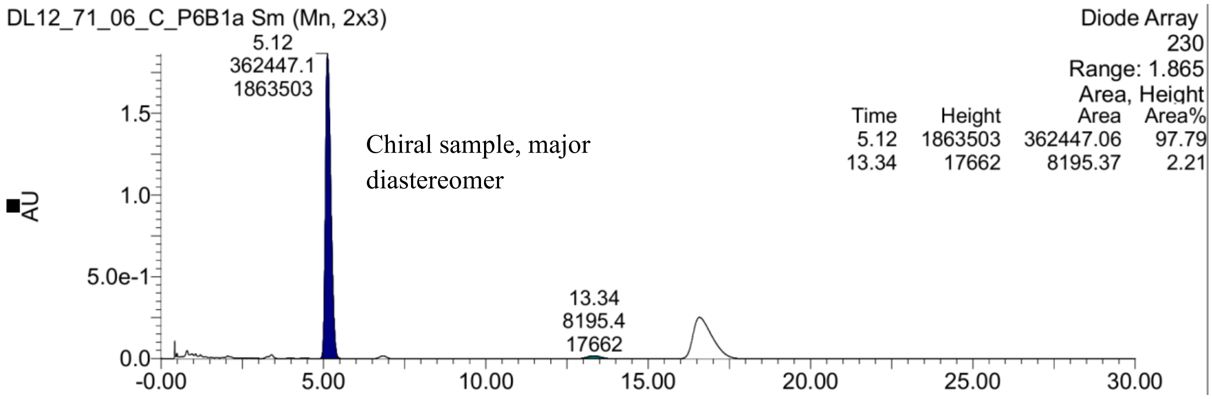
DL12_71_06_C_P6B1a Sm (Mn, 2x3)
Diode Array
230
Range: 1.865
Area, Height
Time Height Area Area%
5.12 1863503 362447.06 97.79
13.34 17662 8195.37 2.21
Chiral sample, major diastereomer
5.12
362447.1
1863503
13.34
8195.4
17662
AU

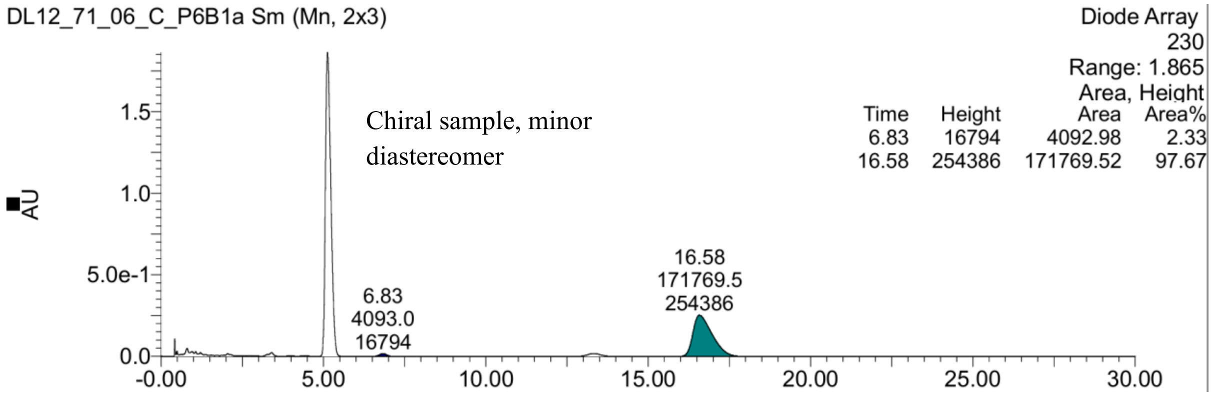
DL12_71_06_C_P6B1a Sm (Mn, 2x3)
Diode Array
230
Range: 1.865
Area, Height
Time Height Area Area%
6.83 16794 4092.98 2.33
16.58 254386 171769.52 97.67
Chiral sample, minor diastereomer
6.83
4093.0
16794
16.58
171769.5
254386
AU

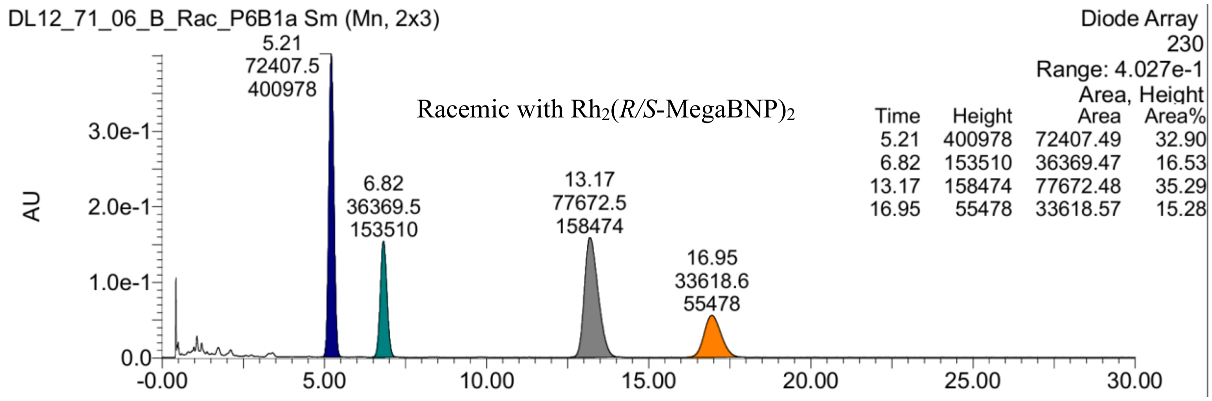
DL12_71_06_B_Rac_P6B1a Sm (Mn, 2x3)
Diode Array
230
Range: 4.027e-1
Area, Height
Racemic with Rh2(R/S-MegaBNP)2
Time Height Area Area%
5.21 400978 72407.49 32.90
6.82 153510 36369.47 16.53
13.17 158474 77672.48 35.29
16.95 55478 33618.57 15.28
5.21
72407.5
400978
6.82
36369.5
153510
13.17
77672.5
158474
16.95
33618.6
55478
AU

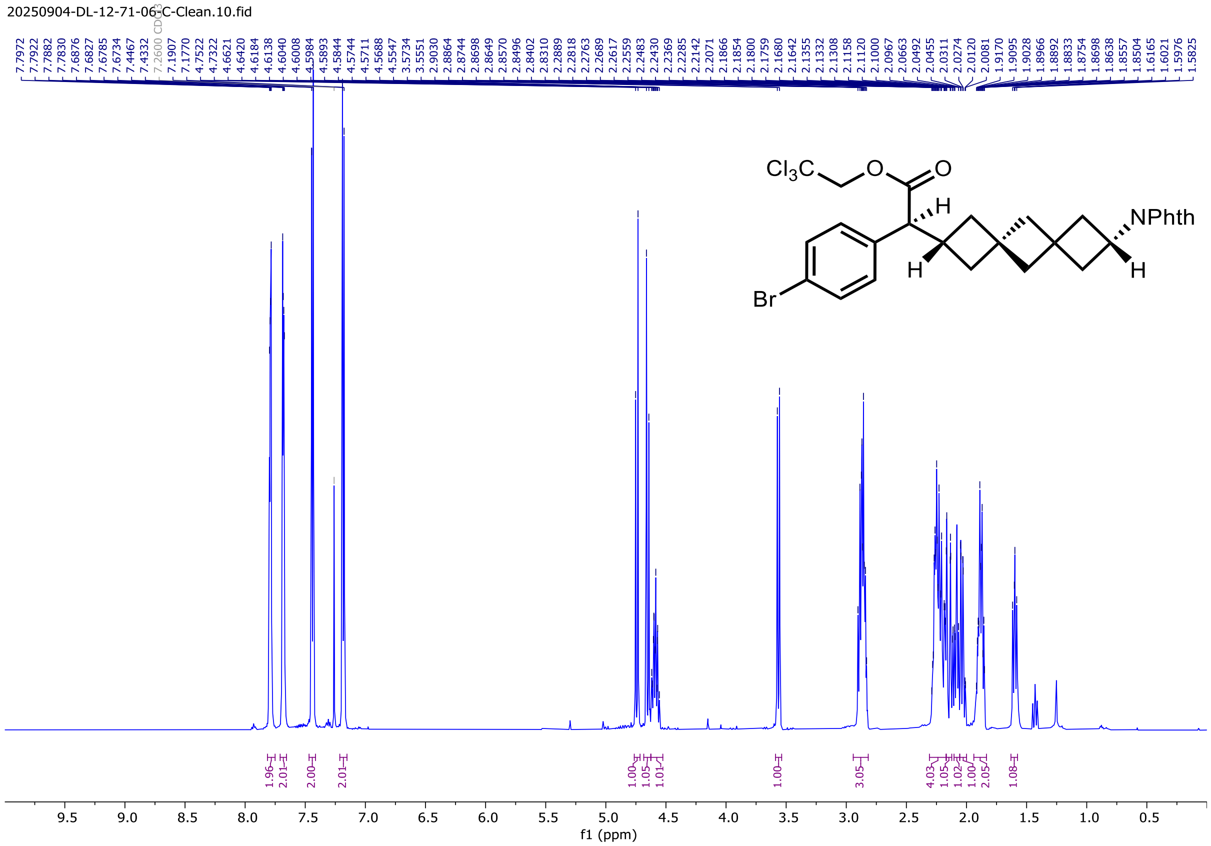
20250904-DL-12-71-06-C-Clean.10.fid
Cl3C
O
O
H
NPhth
H
H
Br
f1 (ppm)

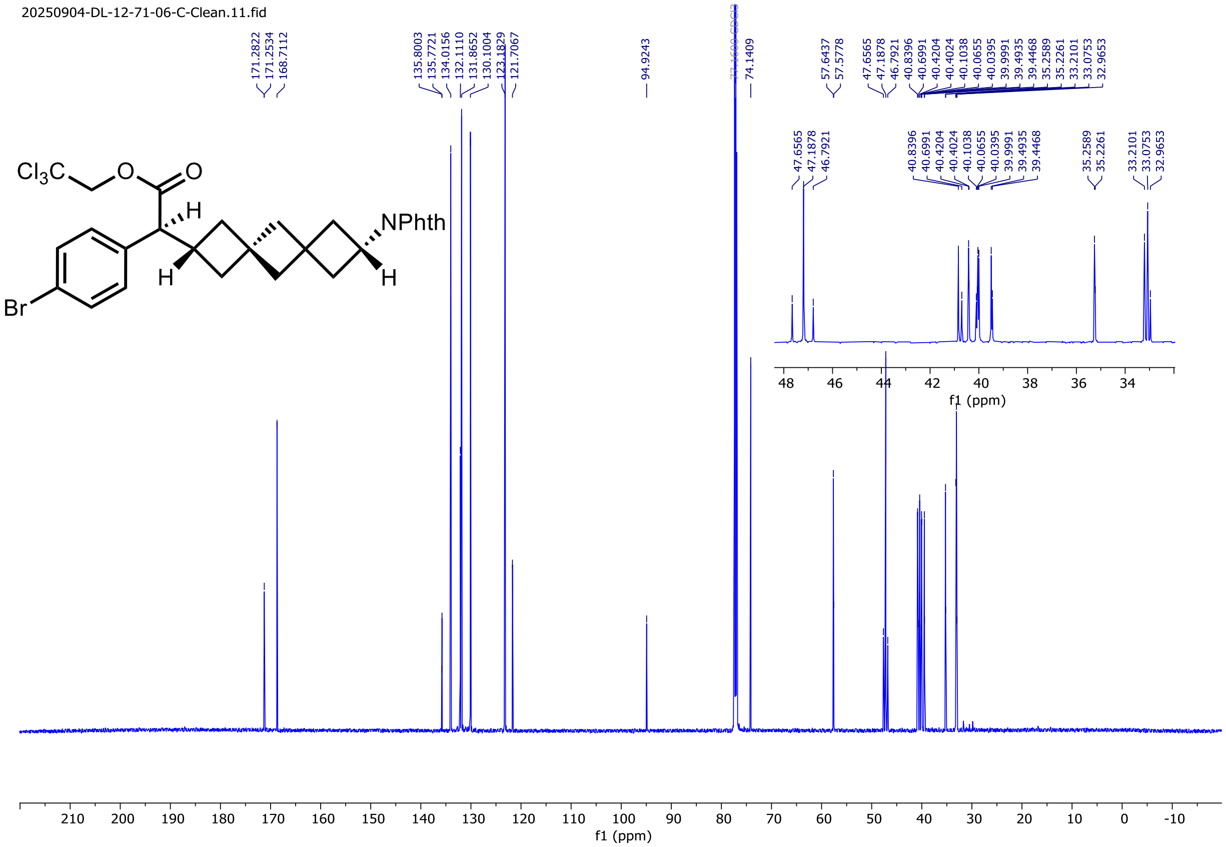
20250904-DL-12-71-06-C-Clean.11.fid
Cl3C
O
O
H
NPhth
H
H
Br
f1 (ppm)

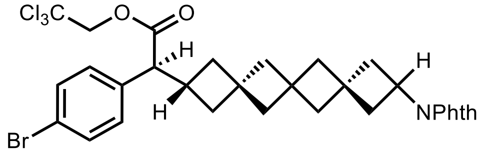

2,2,2-trichloroethyl (S)-2-(4-bromophenyl)-2-(10-(1,3-dioxoisoindolin-2-yl)trispiro[3.1.1.38.16.14]tridecan-2-yl)acetate (Compound 49)

Prepared according to general procedure for C-H functionalization, 2-(trispiro[3.1.1.38.16.14]tridecan-2-yl)isoindoline-1,3-dione (64.3 mg, 0.2 mmol, 1.0 equiv), Rh_2(*S*-MegaBNP$)_4$ (3.4 mg, 0.0001 mmol, 0.005 equiv), molecular sieve 4Å (100 wt%) and 2,2,2- HFIP (5 µL, 8.40 mg, 0.05 mmol, 0.25 equiv) in 0.5 ml CH_2Cl_2 were added a solution of trichloroethyl 2-(4-bromophenyl)-2-diazoacetate (149.0 mg, 0.4 mmol, 2.0 equiv) in 2.0 ml CH_2Cl_2 at 39℃ in 3 hours. The crude mixture was purified by flash chromatography (SiO_2, gradient 0%-25% Et_2O in hexane) afforded **compound 49** as a white amorphous solid (111.3 mg, 84% yield, 92% ee, 1:1 dr).

R_f (1Hex/2Et_2O) = 0.50 (CAM, UV 254 nm)

$[\alpha]^{20}_D$: 15.0° (c = 1.30 g/100 ml, $CHCl_3$, 92% ee)

^{1}H NMR (800 MHz, $CDCl_3$) δ 7.82 – 7.75 (m, 2H), 7.71 – 7.65 (m, 2H), 7.44 (d, *J* = 8.4 Hz, 2H), 7.17 (d, *J* = 8.4 Hz, 2H), 4.74 (d, *J* = 11.9 Hz, 1H), 4.65 (d, *J* = 11.9 Hz, 1H), 4.57 (h, *J* = 9.0 Hz, 1H), 3.55 (d, *J* = 11.1 Hz, 1H), 2.89 – 2.79 (m, 3H), 2.29 – 2.17 (m, 3H), 2.11 – 1.99 (m, 6H), 1.95 (t, *J* = 11.7 Hz, 1H), 1.91 – 1.84 (m, 2H), 1.83 (dd, *J* = 11.3, 8.3 Hz, 1H), 1.58 – 1.54 (m, 1H). (analyzed as a mixture of 2 diastereomers)

^{13}C NMR (151 MHz, $CDCl_3$) δ 171.32, 168.73, 135.86, 133.99, 132.15, 131.85, 130.09, 123.17, 121.68, 94.94, 74.15, 57.63, 48.06, 47.72, 47.60, 47.56, 47.20, 47.18, 40.90, 40.88, 40.52, 40.45, 40.15, 40.10, 39.60, 39.52, 35.18, 34.76, 33.24, 33.22, 33.21. (Analyzed as a mixture of 2 diastereomers

HRMS (+p APCI) calcd. for $[C_{31}H_{30}O_4N^{79}Br^{35}Cl_3]$ ($[M+H]^+$) 664.0418 found 664.0433.

HPLC (Chiralpak ADH column, 10% isopropanol in hexane, 1.0 $mLmin^{-1}$, 1.0 $mgmL^{-1}$, 60 min, UV 230 nm) retention times of 26.6 min (minor) and 30.6 min (major), 92% ee for one diastereomer and retention times of 32.8 (minor) and 35.1 (major), 92% for the second diastereomer.

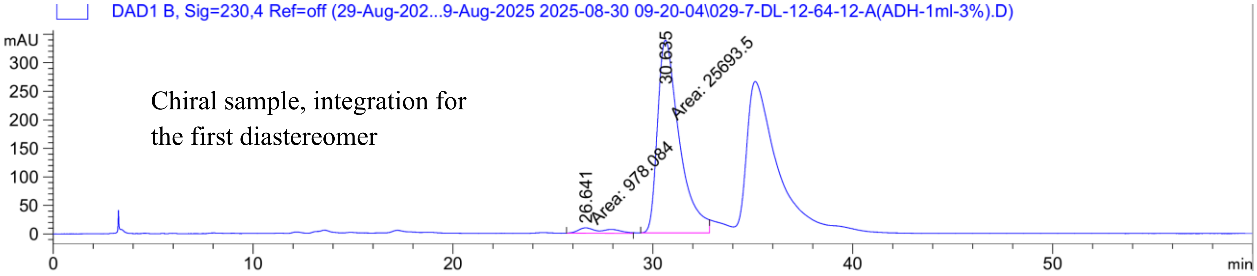

Signal 2: DAD1 B, Sig=230,4 Ref=off

Peak #	RetTime [min]	Type	Width [min]	Area [mAU*s]	Height [mAU]	Area %
1	26.641	MM	1.6972	978.08417	9.60500	3.6671
2	30.635	MF	1.2678	2.56935e4	337.77380	96.3329

Totals : 2.66716e4 347.37880

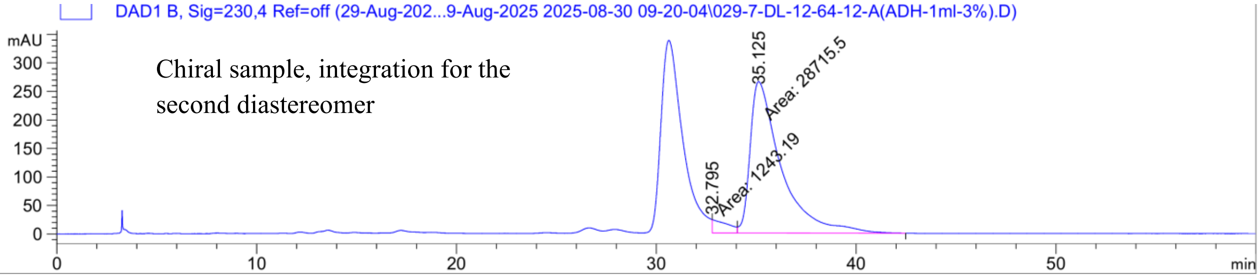

Signal 2: DAD1 B, Sig=230,4 Ref=off

Peak #	RetTime [min]	Type	Width [min]	Area [mAU*s]	Height [mAU]	Area %
1	32.795	FM	0.8889	1243.19360	23.31062	4.1497
2	35.125	FM	1.8045	2.87155e4	265.21634	95.8503

Totals : 2.99587e4 288.52696

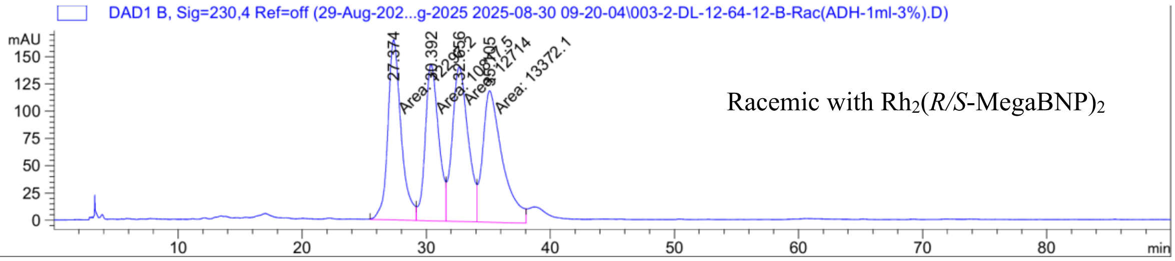

Signal 2: DAD1 B, Sig=230,4 Ref=off

Peak #	RetTime [min]	Type	Width [min]	Area [mAU*s]	Height [mAU]	Area %
1	27.374	MF	1.2399	1.22972e4	165.30406	24.9939
2	30.392	MF	1.2591	1.08175e4	143.19453	21.9864
3	32.656	MF	1.4946	1.27140e4	141.77908	25.8411
4	35.105	FM	1.8476	1.33721e4	120.62526	27.1786

Totals : 4.92007e4 570.90294

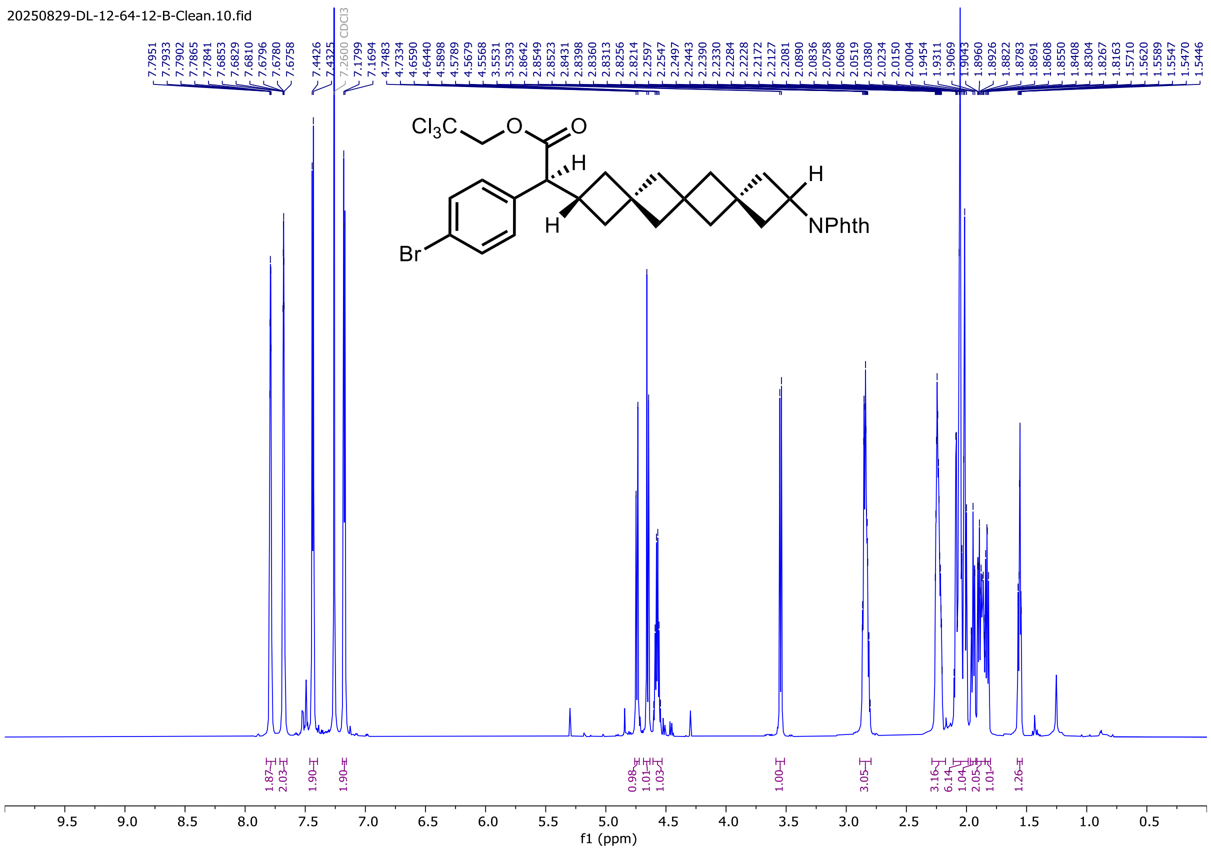
20250829-DL-12-64-12-B-Clean.10.fid
Cl3C
O
O
H
H
H
NPhth
Br
f1 (ppm)

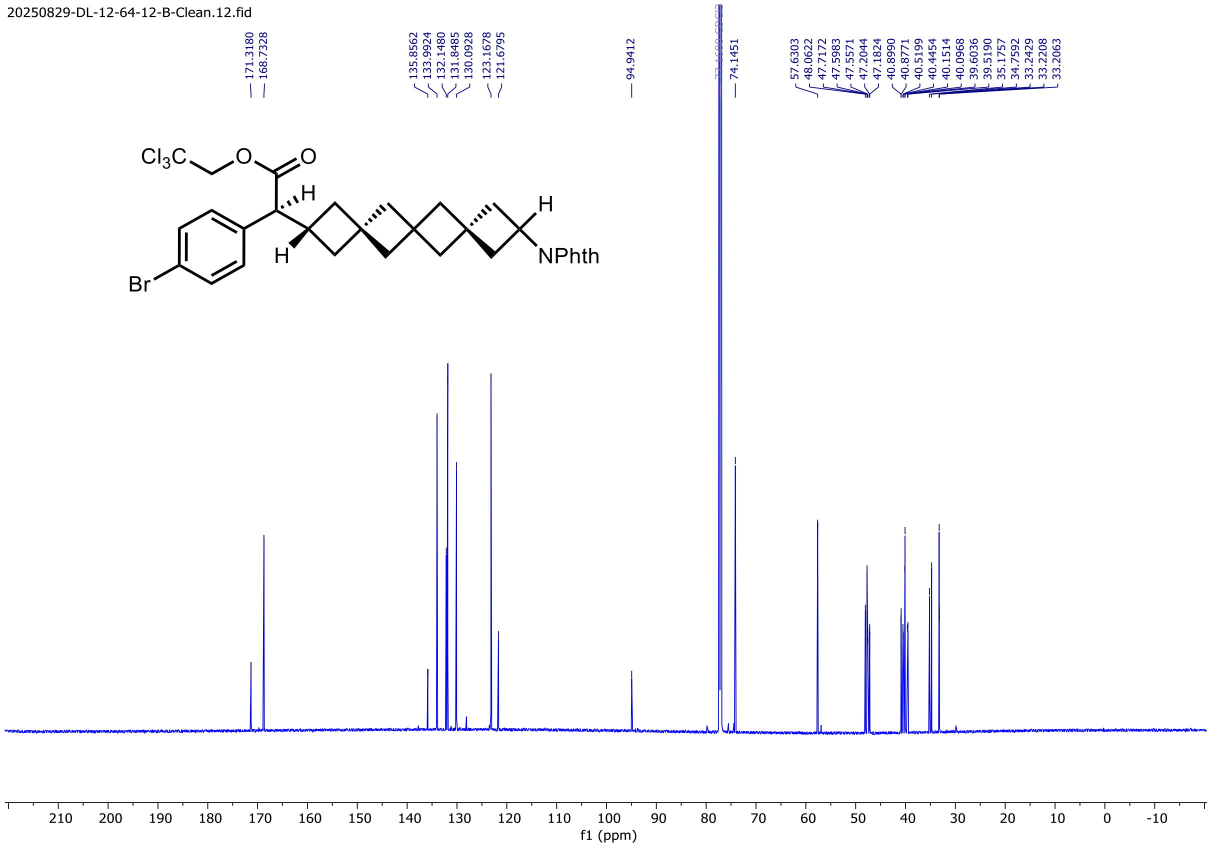
20250829-DL-12-64-12-B-Clean.12.fid
Cl3C
O
O
H
H
H
NPhth
Br
f1 (ppm)

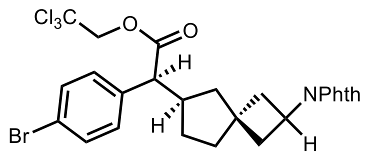

2,2,2-trichloroethyl (2S)-2-(4-bromophenyl)-2-((6R)-2-(1,3-dioxoisoindolin-2-yl)spiro[3.4]octan-6-yl)acetate (Compound 51)

Prepared according to general procedure for C-H functionalization, 2-(spiro[3.4]octan-2-yl)isoindoline-1,3-dione (51.0 mg, 0.2 mmol, 1.0 equiv), Rh_2(*S*-MegaBNP)$_4$ (3.4 mg, 0.0001 mmol, 0.005 equiv), molecular sieve 4Å (100 wt%) and 2,2,2- HFIP (5 µL, 8.40 mg, 0.05 mmol, 0.25 equiv) in 0.5 ml CH_2Cl_2 were added a solution of trichloroethyl 2-(4-bromophenyl)-2-diazoacetate (149.0 mg, 0.4 mmol, 2.0 equiv) in 2.0 ml CH_2Cl_2 at 39℃ in 3 hours. The crude mixture was purified by flash chromatography (SiO_2, gradient 0%-25% Et_2O in hexane) afforded **compound 51** as a white amorphous solid (95.2 mg, 79% yield, 98% ee, 5:1 dr). Note. *The relative stereocenter of the major diastereomer was assigned by the shielding effect of the most up field triplet proton signal. The cis and trans relationship of the carbene fragment and NPhth was determined by NOSEY correlation. See discussion below for structure elucidation.*

R_f (1Hex/2Et_2O) = 0.50 (CAM, UV 254 nm)

$[\alpha]^{20}_D$: 29.0° (c = 0.73 g/100 ml, $CHCl_3$, 98% ee)

^{1}H NMR (800 MHz, $CDCl_3$) δ 7.78 (dd, *J* = 5.5, 3.0 Hz, 2H), 7.68 (dd, *J* = 5.5, 3.0 Hz, 2H), 7.46 (d, *J* = 8.1 Hz, 2H), 7.24 (d, *J* = 8.1 Hz, 2H), 4.76 (d, *J* = 12.0 Hz, 1H), 4.69 (p, *J* = 9.0 Hz, 1H), 4.64 (d, *J* = 12.0 Hz, 1H), 3.40 (d, *J* = 11.3 Hz, 1H), 2.86 – 2.80 (m, 1H), 2.75 (t, *J* = 10.5 Hz, 1H), 2.72 – 2.66 (m, 1H), 2.23 – 2.15 (m, 2H), 2.08 – 2.02 (m, 1H), 1.85 (t, *J* = 7.3 Hz, 2H), 1.70 (dd, *J* = 13.4, 7.9 Hz, 1H), 1.49 – 1.41 (m, 1H), 1.27 (dd, *J* = 13.4, 9.2 Hz, 1H). (For clarity, only the major diastereomer is reported)

^{13}C NMR (201 MHz, $CDCl_3$) δ 171.8, 168.7, 136.8, 134.1, 132.1, 132.0, 130.2, 123.2, 121.8, 94.9, 74.2, 57.6, 45.0, 41.4, 40.5, 39.9, 39.3, 38.6, 38.2, 30.4. (For clarity, only the major diastereomer is reported)

HRMS (+p APCI) calcd. for $[C_{26}H_{24}O_4N^{79}Br^{35}Cl_3]$ ($[M+H]^+$) 597.9949 found 597.9964.

HPLC (Chiralpak ASH column, 2% isopropanol in hexane, 1.0 $mLmin^{-1}$, 1.0 $mgmL^{-1}$, 60 min, UV 230 nm) retention times of 17.4 min (minor) and 21.8 min (major), 98% ee.

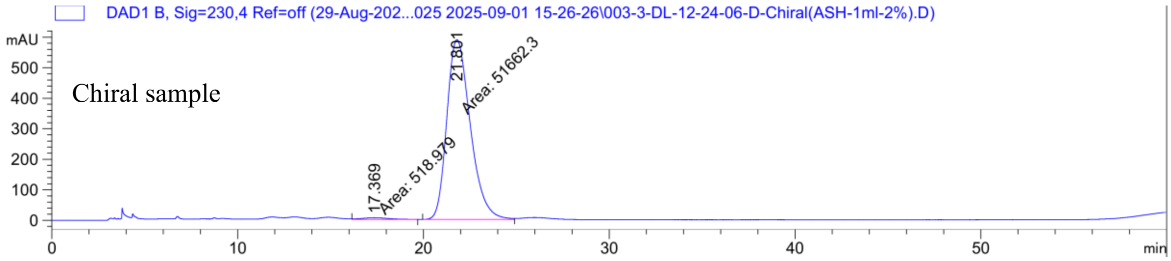

Signal 2: DAD1 B, Sig=230,4 Ref=off

Peak #	RetTime [min]	Type	Width [min]	Area [mAU*s]	Height [mAU]	Area %
1	17.369	FM	1.6653	518.97882	5.19407	0.9946
2	21.801	MF	1.4624	5.16623e4	588.79065	99.0054

Totals : 5.21813e4 593.98472

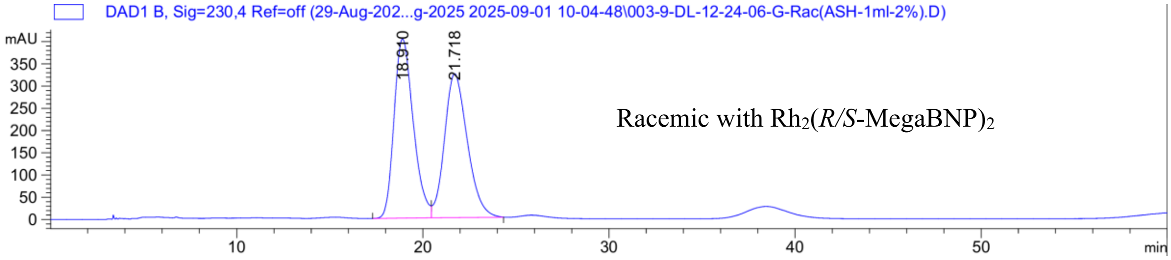

Signal 2: DAD1 B, Sig=230,4 Ref=off

Peak #	RetTime [min]	Type	Width [min]	Area [mAU*s]	Height [mAU]	Area %
1	18.910	VV R	0.8410	2.89722e4	403.35254	51.1592
2	21.718	VV R	0.9998	2.76593e4	323.89899	48.8408

Totals : 5.66315e4 727.25153

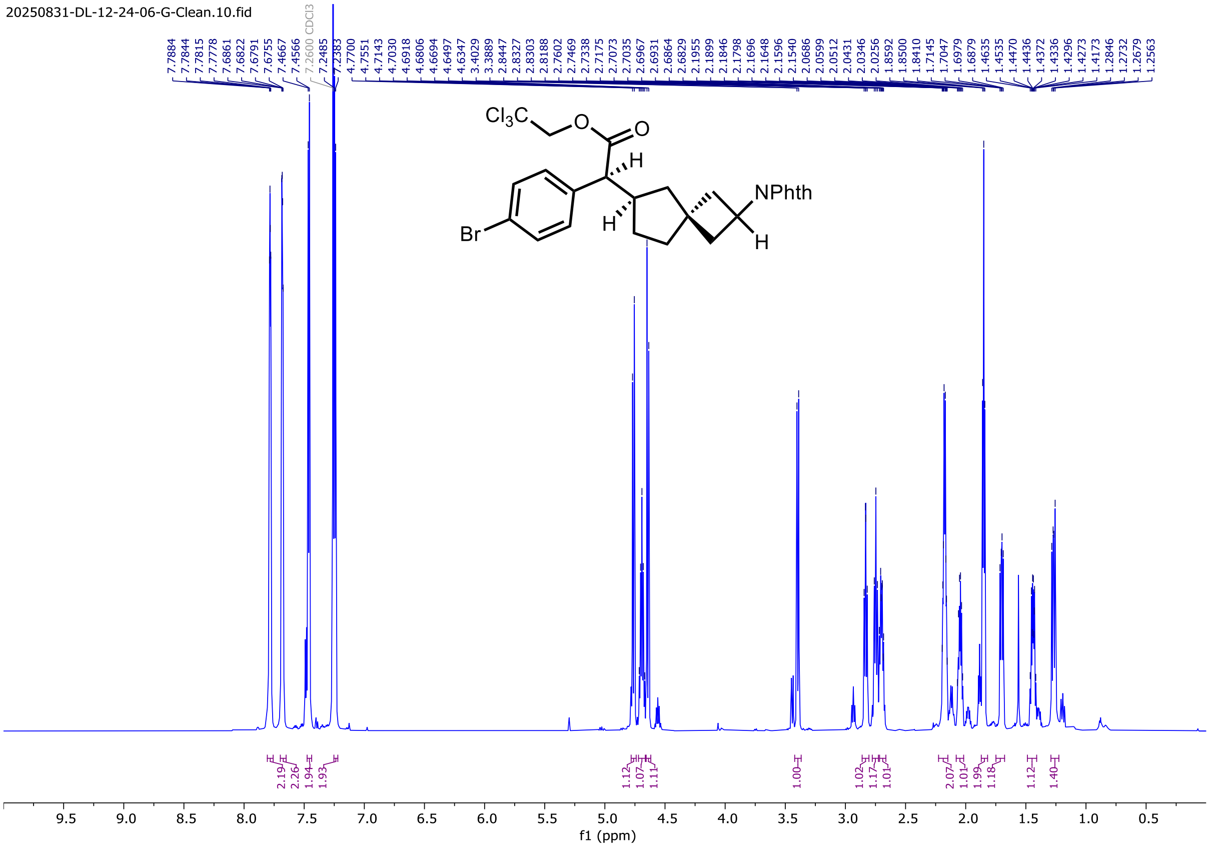
20250831-DL-12-24-06-G-Clean.10.fid
7.7884
7.7844
7.7815
7.7778
7.6861
7.6822
7.6791
7.6755
7.4667
7.4566
7.2600 CDCl3
7.2485
7.2383
4.7700
4.7551
4.7143
4.7030
4.6918
4.6806
4.6694
4.6497
4.6347
3.4029
3.3889
2.8447
2.8327
2.8303
2.8188
2.7602
2.7469
2.7338
2.7175
2.7073
2.7035
2.6967
2.6931
2.6864
2.6829
2.1955
2.1899
2.1846
2.1798
2.1696
2.1648
2.1596
2.1540
2.0686
2.0599
2.0512
2.0431
2.0346
2.0256
1.8592
1.8500
1.8410
1.7145
1.7047
1.6979
1.6879
1.4635
1.4535
1.4470
1.4436
1.4372
1.4336
1.4296
1.4273
1.4173
1.2846
1.2732
1.2679
1.2563
Cl3C O O H NPhth Br H H
2.19
2.26
1.94
1.93
1.12
1.07
1.11
1.00
1.02
1.17
1.01
2.07
1.01
1.99
1.18
1.12
1.40
9.5 9.0 8.5 8.0 7.5 7.0 6.5 6.0 5.5 5.0 4.5 4.0 3.5 3.0 2.5 2.0 1.5 1.0 0.5
f1 (ppm)

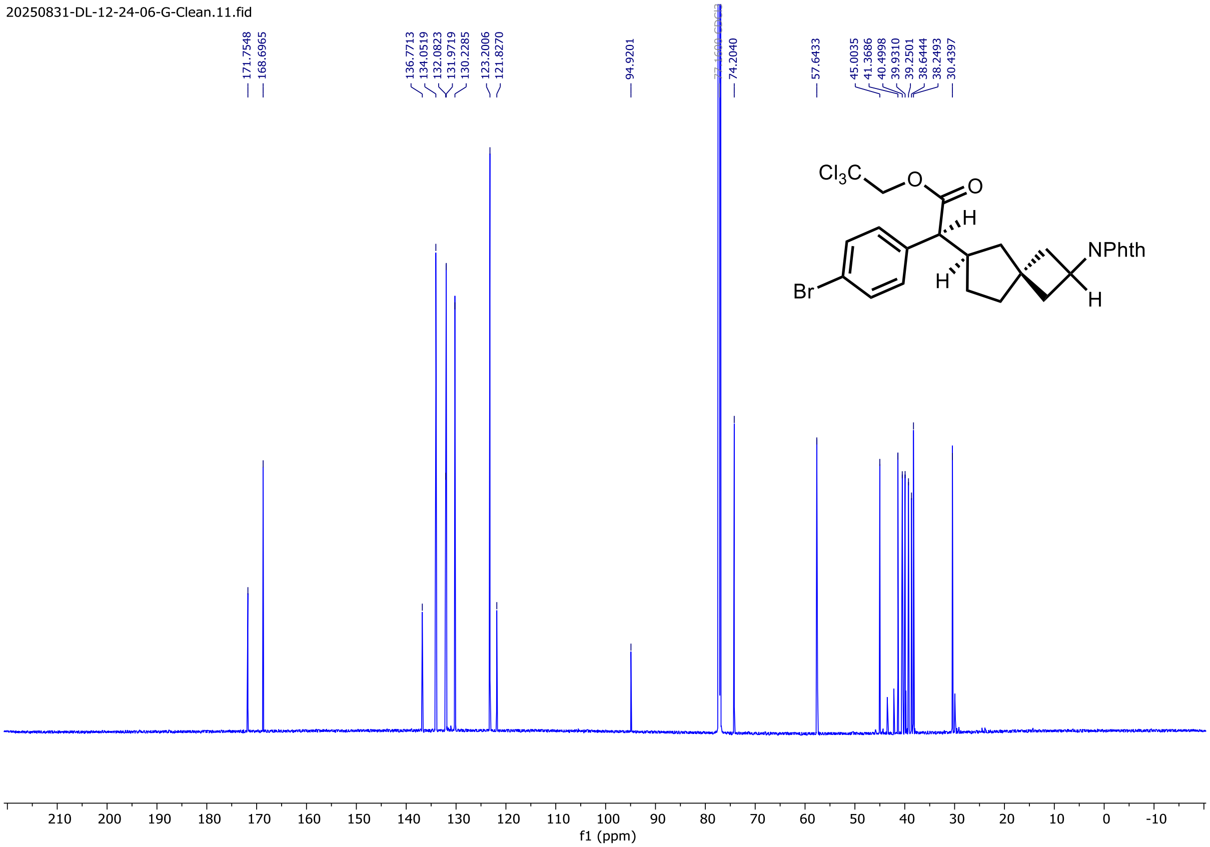
20250831-DL-12-24-06-G-Clean.11.fid
171.7548
168.6965
136.7713
134.0519
132.0823
131.9719
130.2285
123.2006
121.8270
94.9201
77.1600 CDCl3
74.2040
57.6433
45.0035
41.3686
40.4998
39.9310
39.2501
38.6444
38.2493
30.4397
Cl3C O O H NPhth Br H H
210 200 190 180 170 160 150 140 130 120 110 100 90 80 70 60 50 40 30 20 10 0 -10
f1 (ppm)

Structure elucidation of compound 51

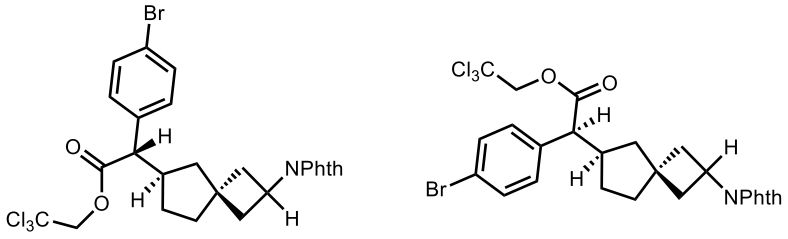

- The major diastereomer conformations were determined based on the NOSEY signal between H3 and (H25; H26), which showed that the carbene attack the methylene at the syn position to the *N*-phthalimido group. The 2nd stereocenter on the cyclopentane ring was determined based on the shielding effect. Particularly, (H41;H42) and (H39;40) have chemical shifts of 1.5-1.7 ppm. However, in the major diastereomer, H42 (dq) locating at the pseudo-equatorial position shifted downfield to 2.05 (0.3-0.5 ppm shifted), while the H39 moved up to 1.2 ppm (0.3-0.5 ppm shifted). This means that the phenyl group of the carbene are located at the syn position to H39. Because the H14 and H15 are in anti-position based on the J coupling, the final configuration for the major diastereomer is (C11(S) and C9 (R))
- The minor diastereomer has a NOSEY signal between H3 and (H39/H40). Therefore, its structure has the *N*-phthalimido anti with the carbene fragment.

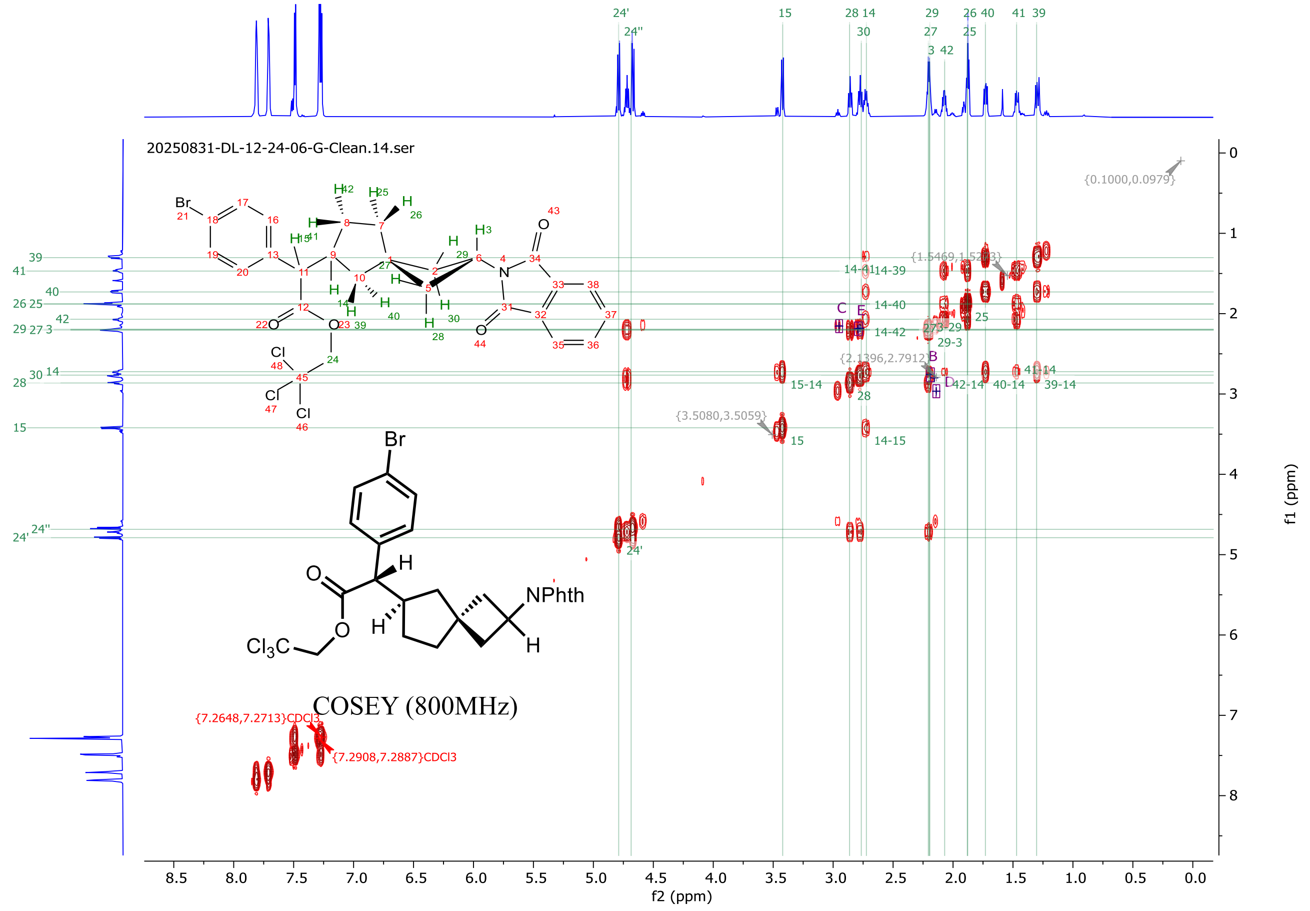
20250831-DL-12-24-06-G-Clean.14.ser
COSEY (800MHz)
NPhth
f2 (ppm)
f1 (ppm)

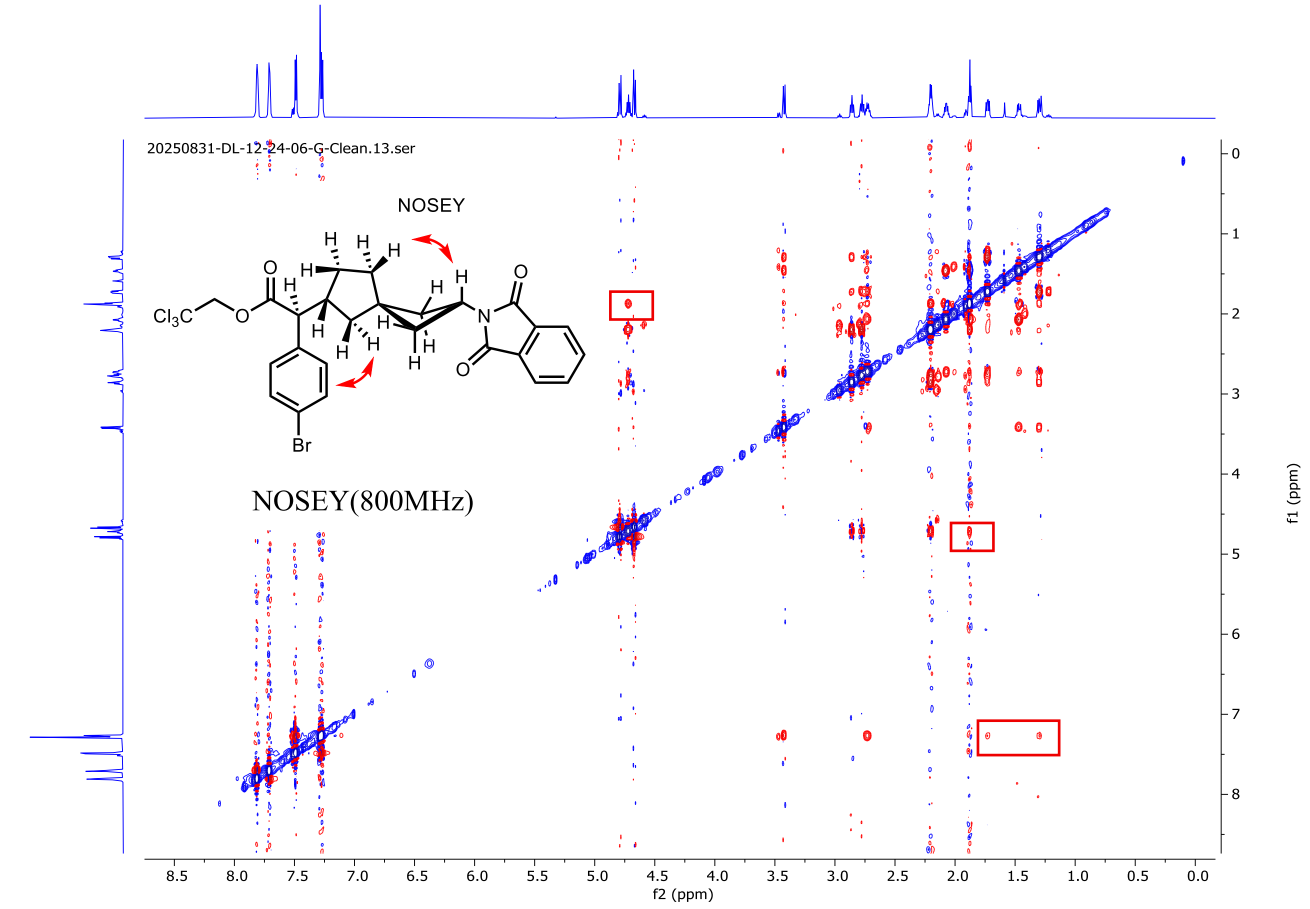
20250831-DL-12-24-06-G-Clean.13.ser
NOSEY
NOSEY(800MHz)
f2 (ppm)
f1 (ppm)

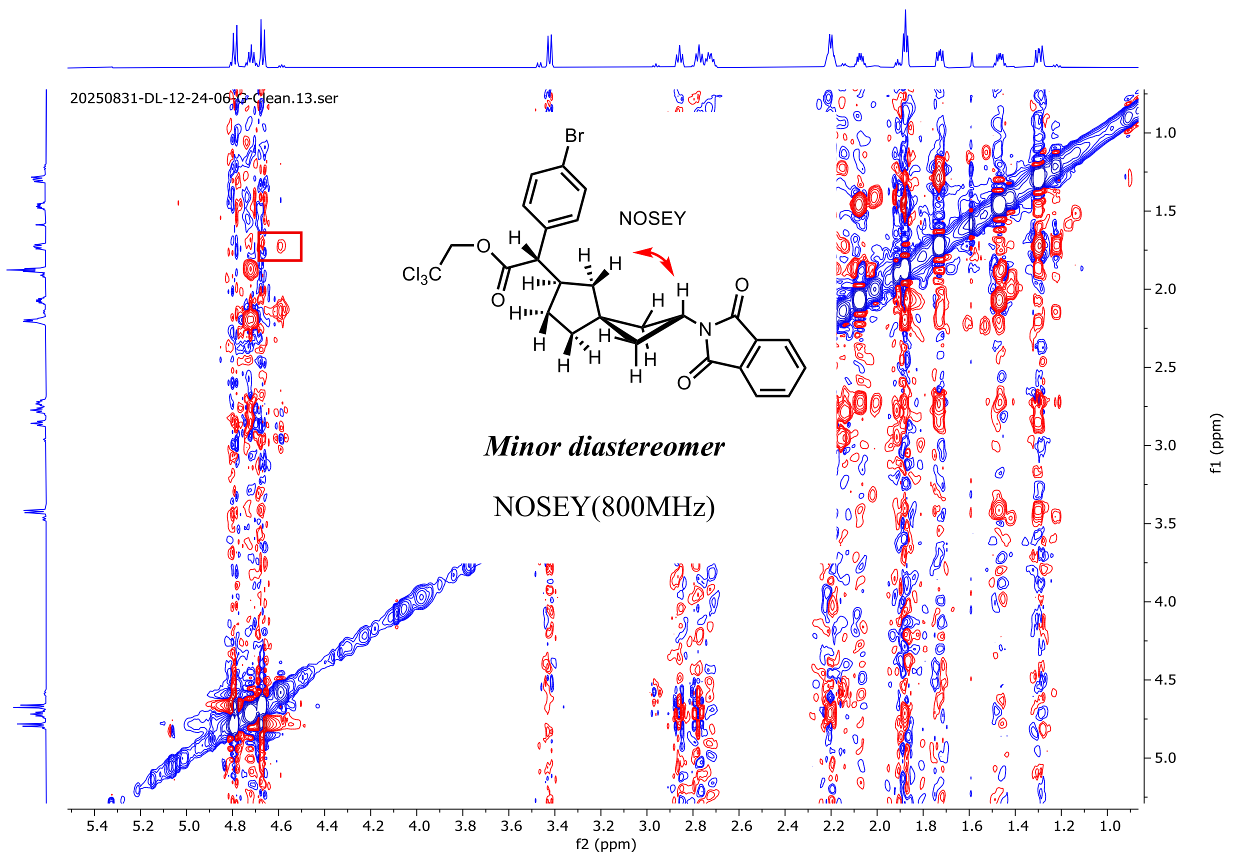
20250831-DL-12-24-06-G-Clean.13.ser
Br
NOSEY
Cl_3C
Minor diastereomer
NOSEY(800MHz)
f2 (ppm)
f1 (ppm)

7. Product transformation

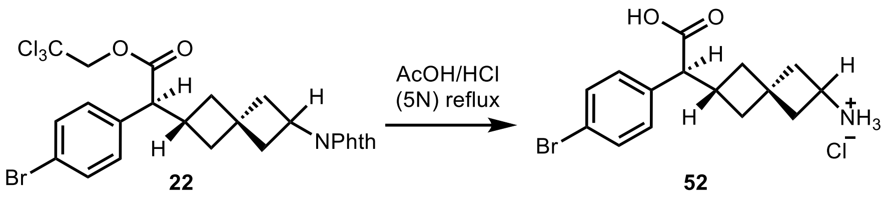

6-((S)-(4-bromophenyl)(carboxy)methyl)spiro[3.3]heptan-2-aminium chloride (Compound 52)

To a 25 mL round bottom flask equipped with a magnetic stir bar, 2,2,2-trichloroethyl 2-(4-bromophenyl)-2-(6-(1,3-dioxoisoindolin-2-yl)spiro[3.3]heptan-2-yl)acetate (117.0 mg, 0.20 mmol, 1.0 equiv) was dissolved in 4 mL of glacial acetic acid. The solution became cloudy upon the addition of 4.0 ml HCl(5 M, aq). The reaction flask was then heated to reflux at 135 °C by a heating block. The reaction became clear with some oil floating around. The reaction was stirring until the solution became homogenous (around 5-6 hours), then cooled to room temperature concentrated to dryness under reduced pressure (toluene can be used to strip acetic acid and water). The crude solid was washed with 5 mL of 3:1 ether:EtOAc (x3) and then dried under vaccum to afford pure **amino acid 52** as a fine white powder (quant. yield, 12:1 dr). The stereocenter of the carboxylic acid was slightly epimerized under the reaction conditions.

$[\alpha]^{20}_{D}$: 36.9° (c = 0.43 g/100 ml, MeOH)

^{1}H NMR (600 MHz, CD_3OD) δ 7.45 (d, J = 8.3 Hz, 2H), 7.21 (d, J = 8.3 Hz, 2H), 3.68 – 3.59 (m, 1H), 3.46 (d, J = 10.9 Hz, 1H), 2.81 (dp, J = 10.9, 8.3 Hz, 1H), 2.53 (ddd, J = 12.2, 7.6, 5.0 Hz, 1H), 2.37 (ddd, J = 11.7, 7.8, 4.2 Hz, 1H), 2.31 (ddd, J = 12.4, 7.6, 5.1 Hz, 1H), 2.12 (dd, J = 11.6, 8.5 Hz, 1H), 2.07 (dd, J = 12.0, 8.6 Hz, 1H), 1.95 (dd, J = 11.3, 8.5 Hz, 1H), 1.83 (ddd, J = 12.0, 7.9, 4.3 Hz, 1H), 1.68 (dd, J = 11.8, 8.9 Hz, 1H).

^{13}C NMR (151 MHz, CD_3OD) δ 176.1, 138.5, 132.6, 131.1, 122.0, 58.6, 42.4, 41.2, 40.7, 40.4, 39.0, 34.4, 34.2.

HRMS (+p APCI) calcd. for [$C_{15}H_{19}O_2N^{79}Br$] ($[M+H]^+$) 324.0594 found 324.0595.

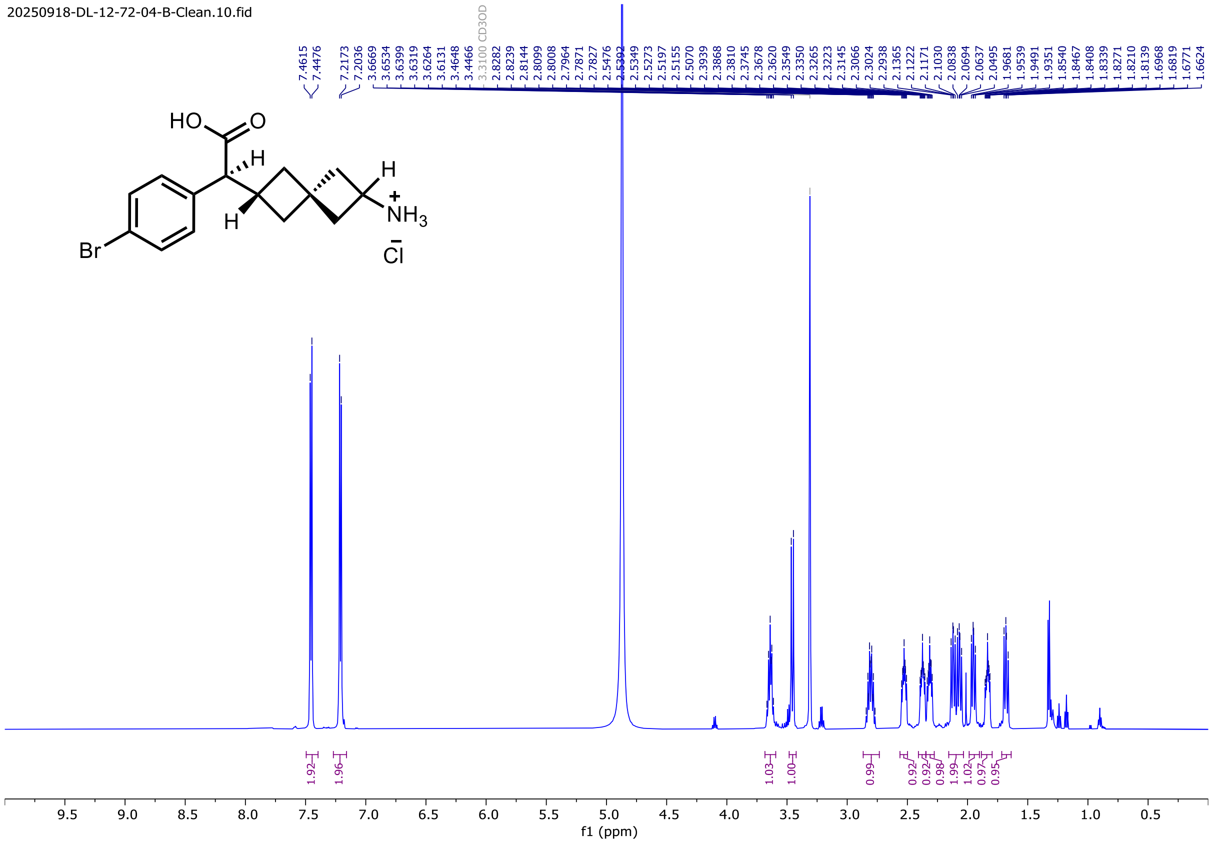
20250918-DL-12-72-04-B-Clean.10.fid
7.4615
7.4476
7.2173
7.2036
3.6669
3.6534
3.6399
3.6319
3.6264
3.6131
3.4648
3.4466
3.3100 CD3OD
2.8282
2.8239
2.8144
2.8099
2.8008
2.7964
2.7871
2.7827
2.5476
2.5392
2.5349
2.5273
2.5197
2.5155
2.5070
2.3939
2.3868
2.3810
2.3745
2.3678
2.3620
2.3549
2.3350
2.3265
2.3223
2.3145
2.3066
2.3024
2.2938
2.1365
2.1222
2.1171
2.1030
2.0838
2.0694
2.0637
2.0495
1.9681
1.9539
1.9491
1.9351
1.8540
1.8467
1.8408
1.8339
1.8271
1.8210
1.8139
1.6968
1.6819
1.6771
1.6624
HO
O
H
H
H
NH3
Br
Cl
1.92
1.96
1.03
1.00
0.99
0.92
0.92
0.98
1.99
1.02
0.97
0.95
9.5 9.0 8.5 8.0 7.5 7.0 6.5 6.0 5.5 5.0 4.5 4.0 3.5 3.0 2.5 2.0 1.5 1.0 0.5
f1 (ppm)

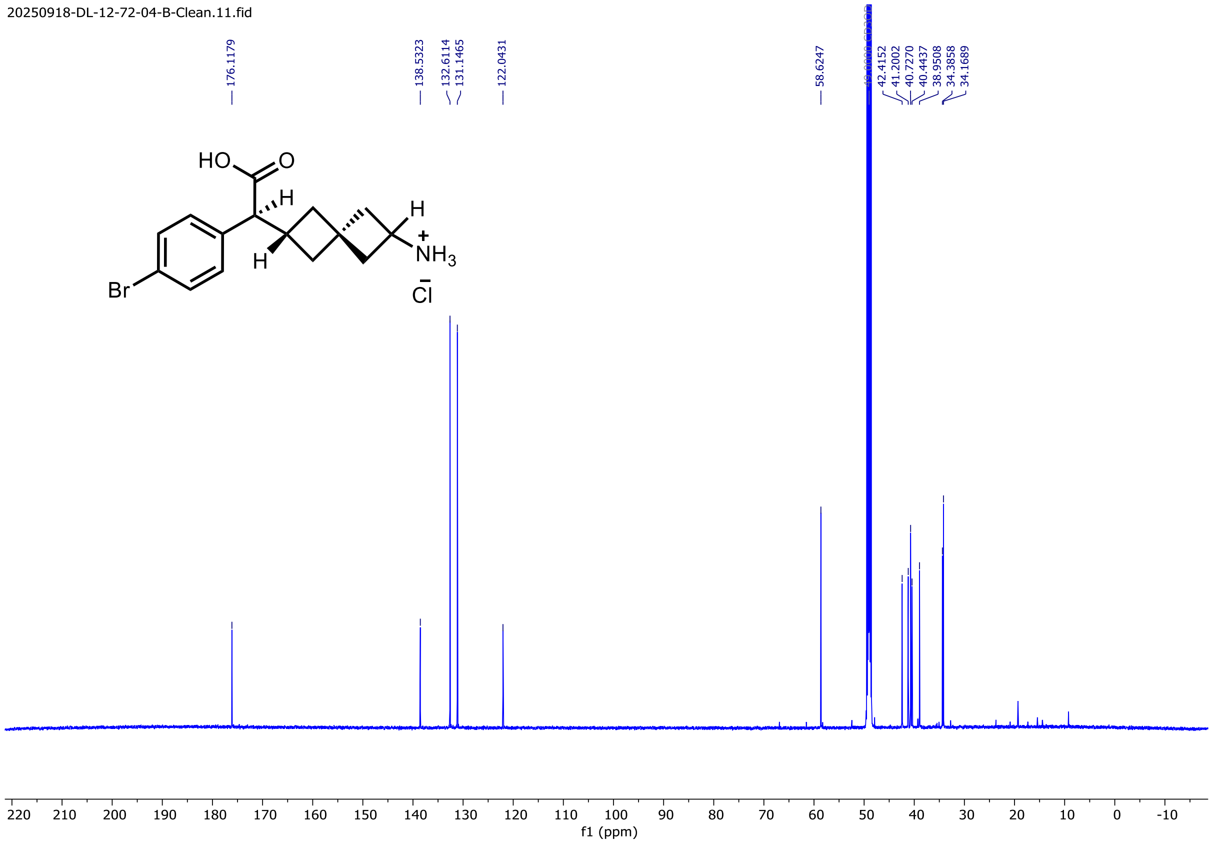
20250918-DL-12-72-04-B-Clean.11.fid
176.1179
138.5323
132.6114
131.1465
122.0431
58.6247
42.4152
41.2002
40.7270
40.4437
38.9508
34.3858
34.1689
HO
O
H
H
H
NH3
Br
Cl
220 210 200 190 180 170 160 150 140 130 120 110 100 90 80 70 60 50 40 30 20 10 0 -10
f1 (ppm)

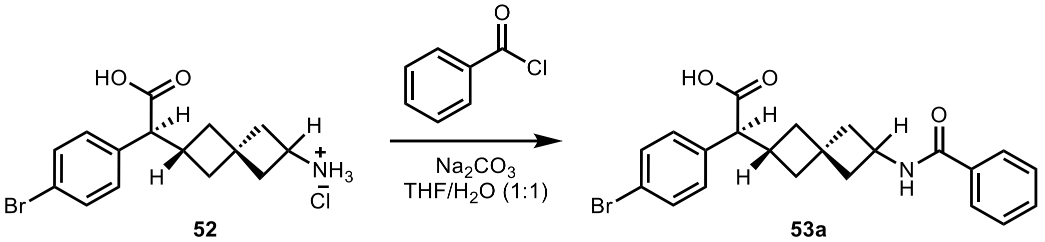

(2S)-2-(6-benzamidospiro[3.3]heptan-2-yl)-2-(4-bromophenyl)acetic acid (Compound 53a)

To a 8 ml vial was added 6-((4-bromophenyl)(carboxy)methyl)spiro[3.3]heptan-2-aminium chloride **52** (72.1 mg, 0.20 mmol, 1.0 equiv). THF (1.0 ml) and water (1.0 ml) were subsequently added. The mixture was stirred until all the amino acid was dissolved. Benzoyl chloride (56.2 mg, 0.4 mmol, 2.0 equiv) was added. The mixture was stirred at room temperature overnight. Upon completion, HCl 2M was added to acidify the reaction mixture. The crude mixture was extracted with ethyl acetate and washed with brine. Solvent was then removed under vacuum. The product was purified by flash chromatography (SiO_2, 0-50% acetone in hexane) to give the desired product **53a** as a clear oil (quant. yield, 12:1 dr).

R_f (1Hex/1Acetone) = 0.50 (CAM, UV 254 nm)

$[\alpha]^{20}_D$: 43.2° (c = 0.29 g/100 ml, MeOH)

^{1}H NMR (600 MHz, $CDCl_3$) δ 7.71 (d, J = 7.1 Hz, 2H), 7.49 – 7.45 (m, 1H), 7.43 (d, J = 8.4 Hz, 2H), 7.39 (t, J = 7.6 Hz, 2H), 7.17 (d, J = 8.4 Hz, 2H), 6.27 (d, J = 7.7 Hz, 1H), 4.49 (h, J = 8.1 Hz, 1H), 3.45 (d, J = 11.1 Hz, 1H), 2.85 (dp, J = 11.2, 8.3 Hz, 1H), 2.66 – 2.60 (m, 1H), 2.49 – 2.38 (m, 2H), 1.96 (dd, J = 11.3, 8.5 Hz, 1H), 1.92 (dd, J = 11.0, 8.9 Hz, 1H), 1.88 (dd, J = 11.3, 8.9 Hz, 1H), 1.82 (ddd, J = 11.9, 7.8, 4.2 Hz, 1H), 1.61 (dd, J = 11.7, 8.8 Hz, 1H).

^{13}C NMR (151 MHz, $CDCl_3$) δ 176.3, 167.2, 136.4, 134.2, 131.8, 131.8, 130.0, 128.7, 127.1, 121.5, 57.7, 43.7, 43.4, 41.2, 40.2, 38.2, 33.4, 33.2.

HRMS (+p APCI) calcd. for [$C_{22}H_{23}O_3N^{79}Br$] ($[M+H]^+$) 428.0856 found 428.0860.

20250918-DL-12-72-06-Clean.10.fid

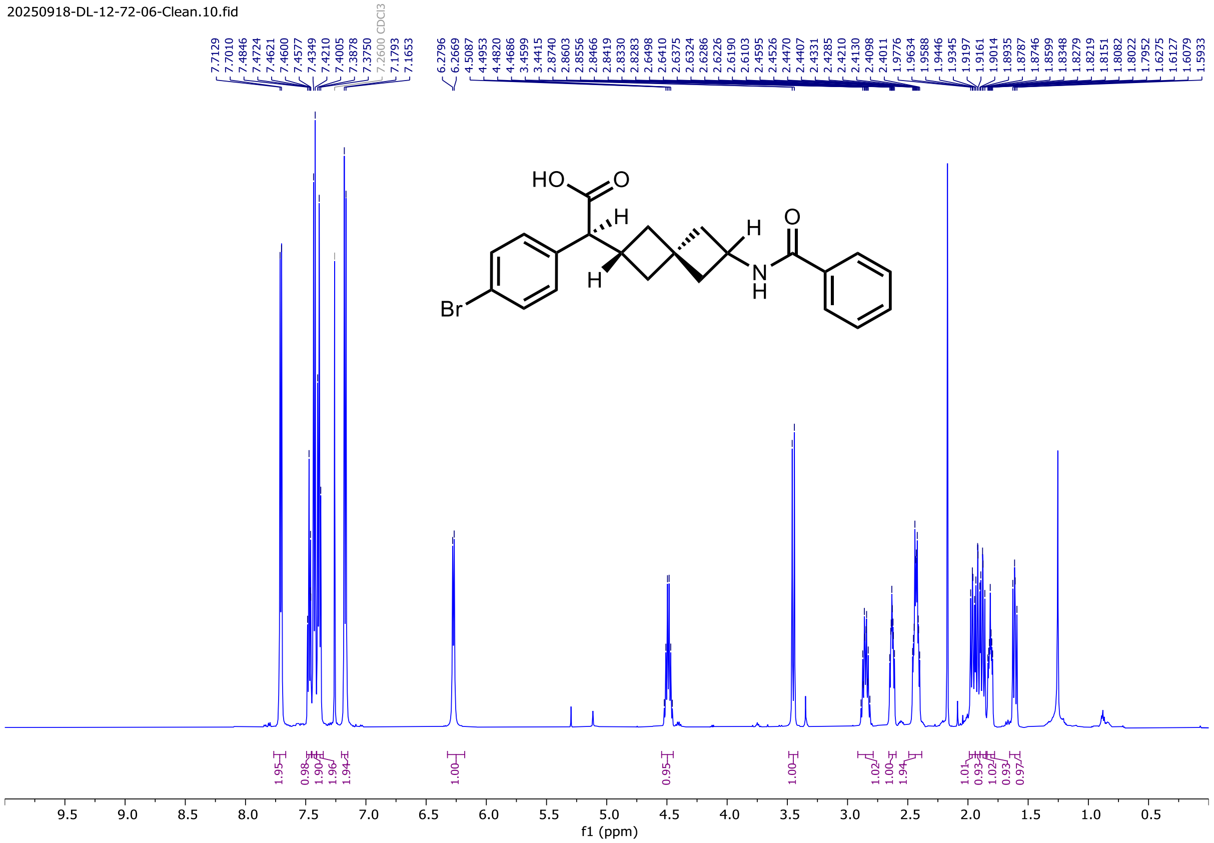

20250918-DL-12-72-06-Clean.11.fid

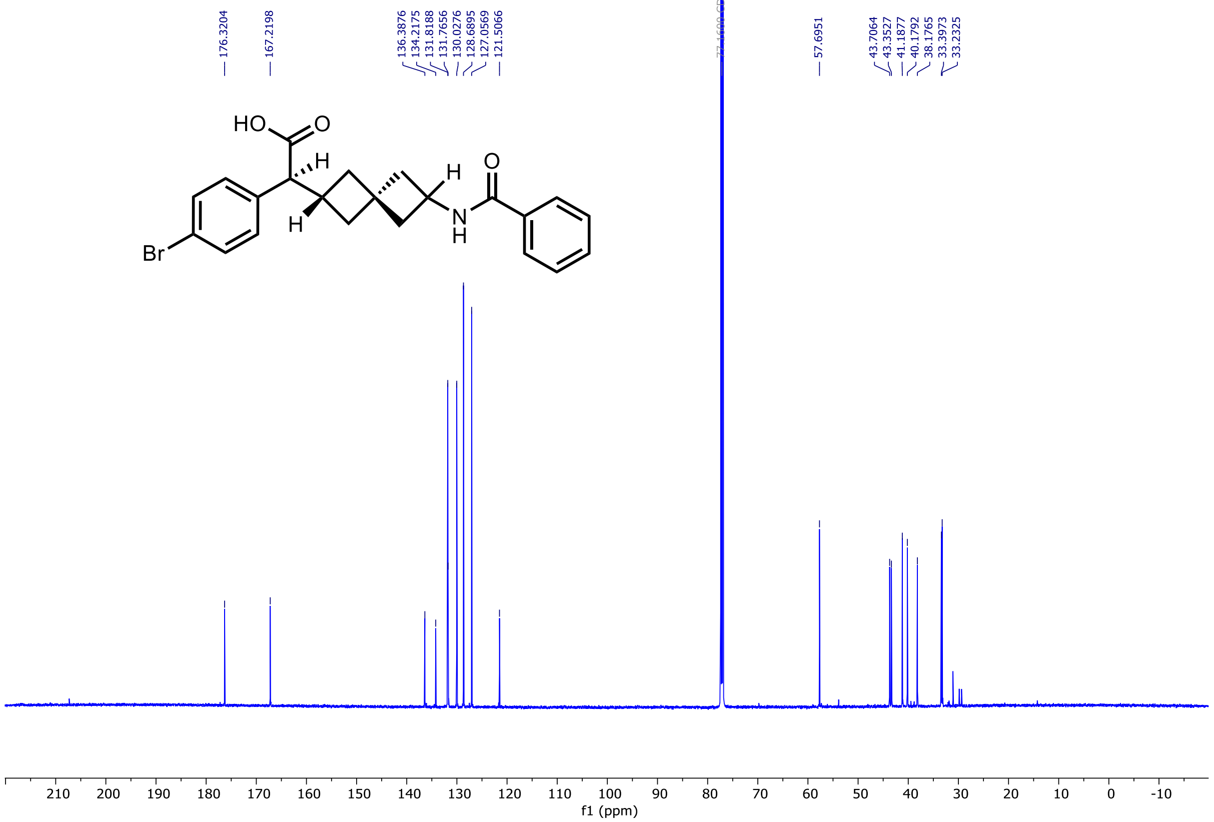

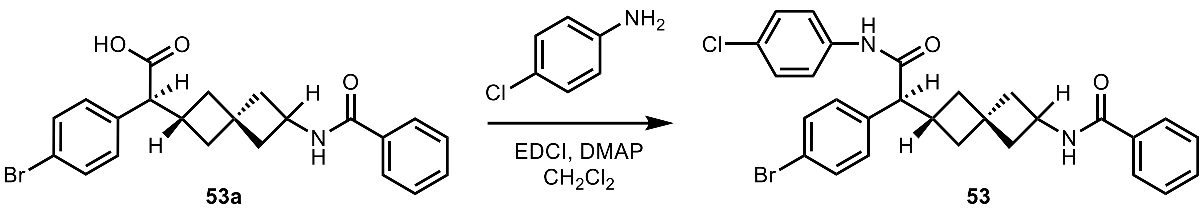

N-(6-((S)-1-(4-bromophenyl)-2-((4-chlorophenyl)amino)-2-oxoethyl)spiro[3.3]heptan-2-yl)benzamide (Compound 53)

To a 8-ml vial was added 2-(6-benzamidospiro[3.3]heptan-2-yl)-2-(4-bromophenyl)acetic acid **53a** (85.7 mg, 0.20 mmol, 1.0 equiv), EDCI (65.2 mg, 0.34 mmol, 1.7 equiv), DMAP (48.9 mg, 0.4 mmol, 2.0 equiv), and HOBt (36.8 mg, 0.24 mmol, 1.2 equiv). DCM (2.0 ml) was added to the above vial. The mixture was stirred for 10 min before 4-chloroaniline (38.3, 0.3 mmol, 1.5 equiv) was added. The mixture was stirred at room temperature overnight. The reaction is then quenched with HCl 2.0 N and extracted with DCM (3 times). The combined organic layers were concentrated and purified by flash chromatography (SiO_2, 0-40% ethyl acetate in hexane) to give the desired product **53** as a white solid (94.1 mg, 88% yield, 12:1 dr, 99% ee). (Note: the opposite enantiomer could be prepared with -98% ee from starting material with opposite enantiomer)

R_f (1Hex/1EA) = 0.50 (CAM, UV 254 nm)

$[\alpha]^{20}_D$: 44.6° (c = 0.26 g/100 ml, MeOH, 99% ee)

^{1}H NMR (600 MHz, CD_3OD) δ 7.78 (d, J = 8.0 Hz, 2H), 7.53 (d, J = 8.8 Hz, 2H), 7.50 (t, J = 7.4 Hz, 1H), 7.46 (d, J = 8.3 Hz, 2H), 7.44 – 7.41 (m, 2H), 7.33 – 7.25 (m, 4H), 4.36 (p, J = 8.2 Hz, 1H), 3.53 (d, J = 10.9 Hz, 1H), 2.94 (dp, J = 10.8, 8.2 Hz, 1H), 2.54 (ddd, J = 11.8, 7.5, 5.1 Hz, 1H), 2.40 – 2.32 (m, 2H), 2.11 (dd, J = 11.0, 8.8 Hz, 1H), 2.05 (dd, J = 11.2, 8.8 Hz, 1H), 1.95 (dd, J = 11.1, 8.2 Hz, 1H), 1.88 (ddd, J = 11.8, 7.8, 3.6 Hz, 1H), 1.67 (dd, J = 11.6, 8.6 Hz, 1H).

^{13}C NMR (151 MHz, CD_3OD) δ 169.6, 139.1, 138.6, 135.7, 132.6, 132.6, 132.6, 131.0, 130.1, 129.8, 129.5, 128.3, 122.6, 122.1, 60.6, 43.7, 43.5, 42.1, 40.8, 39.2, 34.6, 34.5.

HRMS (+p APCI) calcd. for [$C_{28}H_{27}O_2N_2{}^{79}Br^{35}Cl$] ([M+H]$^+$) 537.0940 found 537.0954.
SFC (CEL1, 30% (50% methanol in isopropanol with 0.2% Formic Acid) in CO_2, 2.5 mL/min, 1.0 mg/ml, UV 230 nm) retention times of 4.47 min (major) and 9.68 min (minor) 99% ee.

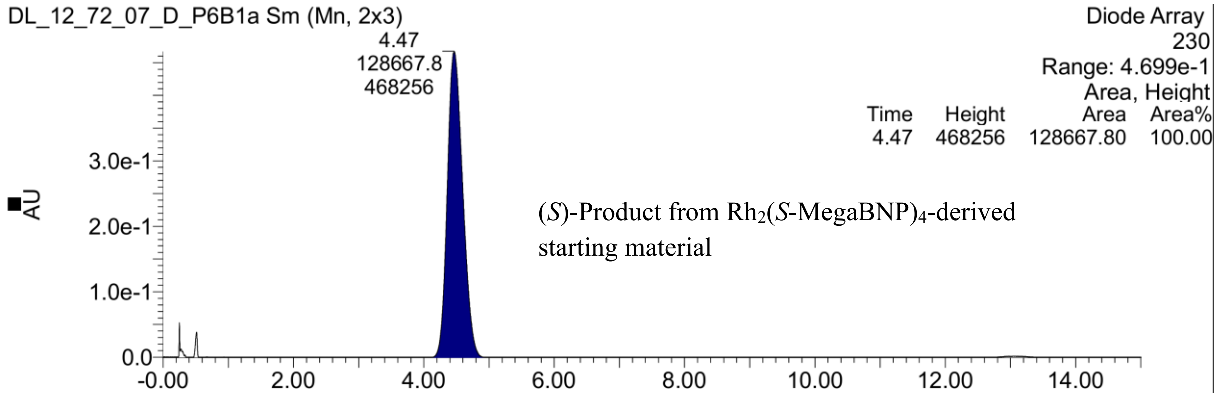
DL_12_72_07_D_P6B1a Sm (Mn, 2x3)
Diode Array
230
Range: 4.699e-1
Area, Height
Time Height Area Area%
4.47 468256 128667.80 100.00
4.47
128667.8
468256
(S)-Product from Rh2(S-MegaBNP)4-derived starting material
AU
3.0e-1
2.0e-1
1.0e-1
0.0
-0.00
2.00
4.00
6.00
8.00
10.00
12.00
14.00

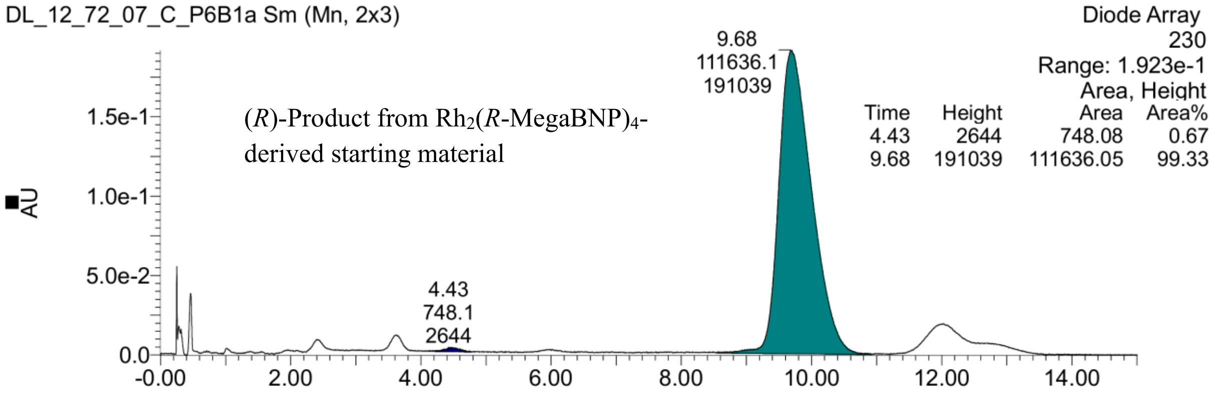
DL_12_72_07_C_P6B1a Sm (Mn, 2x3)
Diode Array
230
Range: 1.923e-1
Area, Height
Time Height Area Area%
4.43 2644 748.08 0.67
9.68 191039 111636.05 99.33
9.68
111636.1
191039
(R)-Product from Rh2(R-MegaBNP)4-derived starting material
4.43
748.1
2644
AU
1.5e-1
1.0e-1
5.0e-2
0.0
-0.00
2.00
4.00
6.00
8.00
10.00
12.00
14.00

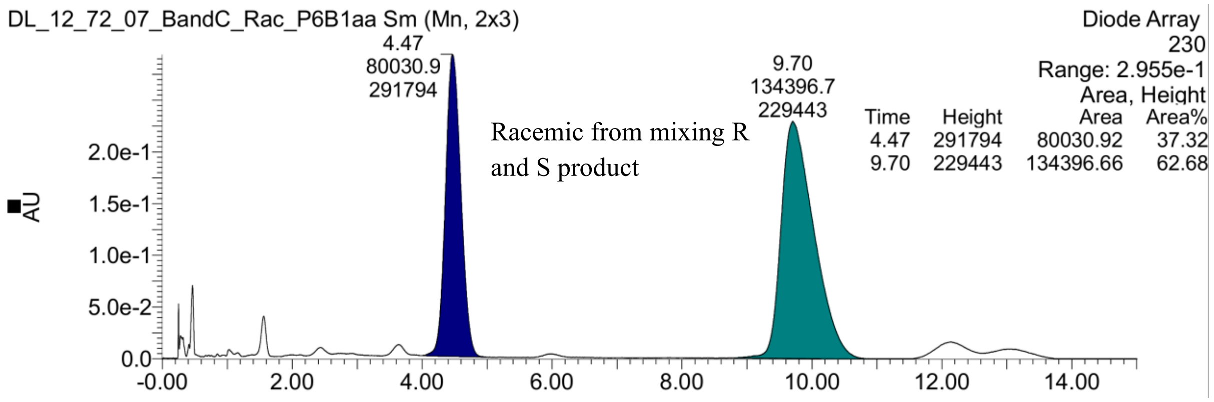
DL_12_72_07_BandC_Rac_P6B1aa Sm (Mn, 2x3)
Diode Array
230
Range: 2.955e-1
Area, Height
Time Height Area Area%
4.47 291794 80030.92 37.32
9.70 229443 134396.66 62.68
4.47
80030.9
291794
9.70
134396.7
229443
Racemic from mixing R and S product
AU
2.0e-1
1.5e-1
1.0e-1
5.0e-2
0.0
-0.00
2.00
4.00
6.00
8.00
10.00
12.00
14.00

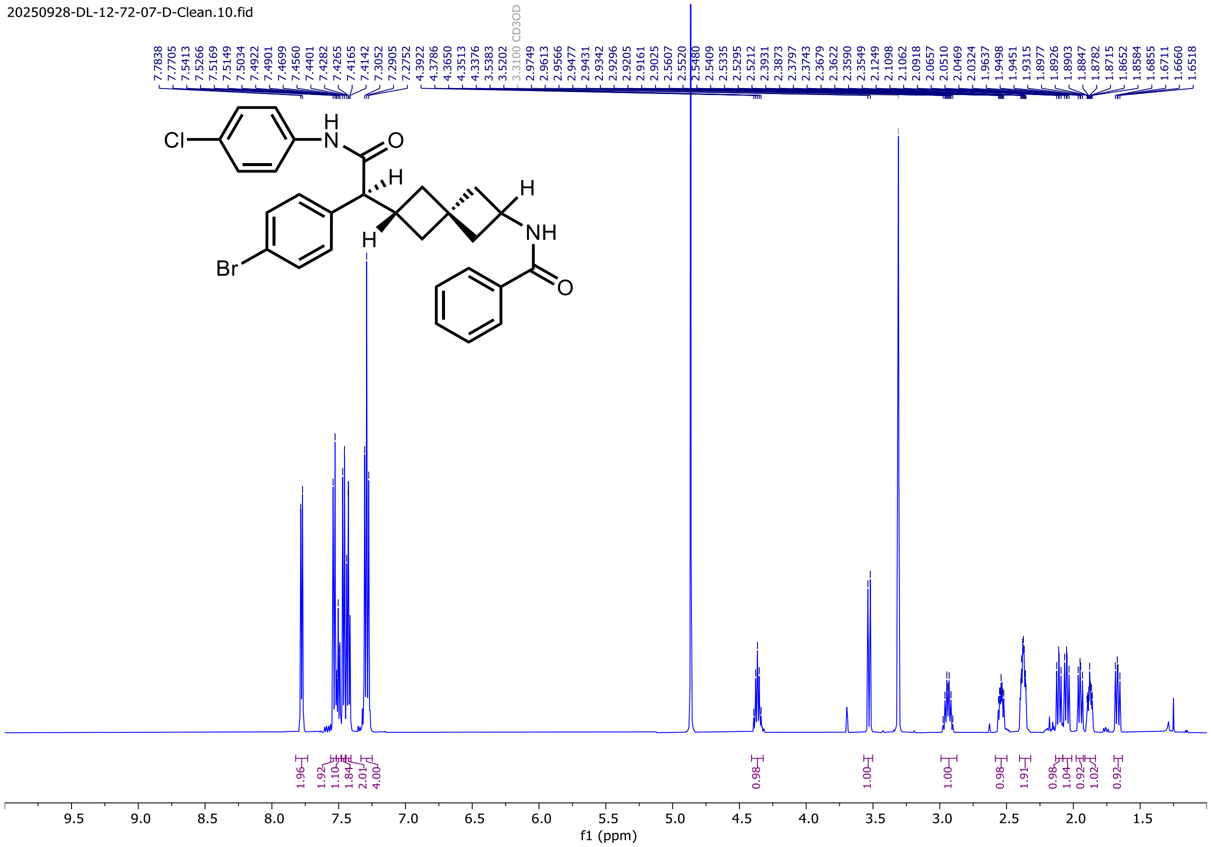
20250928-DL-12-72-07-D-Clean.10.fid
f1 (ppm)

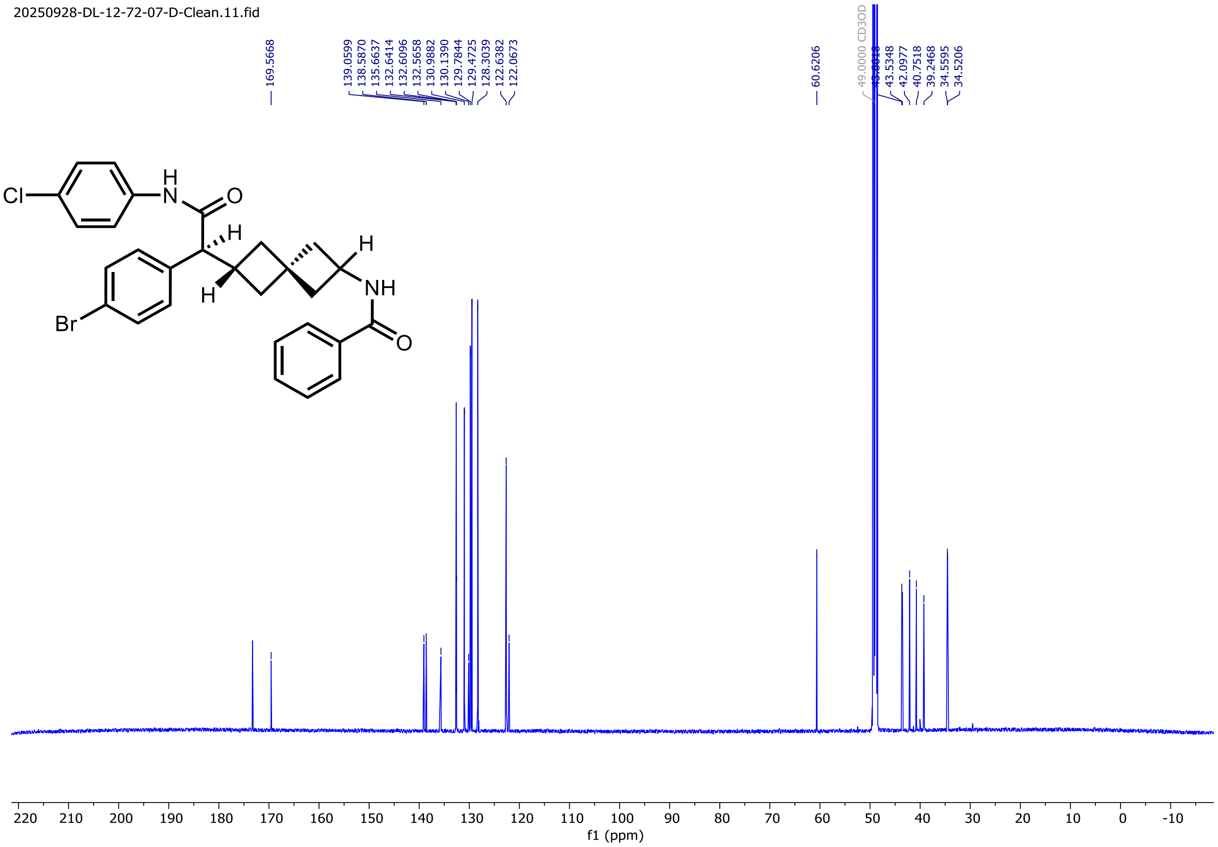
20250928-DL-12-72-07-D-Clean.11.fid
f1 (ppm)

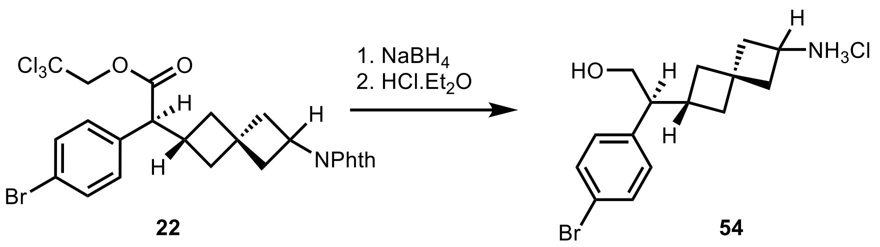

6-((S)-1-(4-bromophenyl)-2-hydroxyethyl)spiro[3.3]heptan-2-aminium chloride (Compound 54)

To a stirred solution 2,2,2-trichloroethyl 2-(4-bromophenyl)-2-(6-(1,3-dioxoisoindolin-2-yl)spiro[3.3]heptan-2-yl)acetate **22** (117 mg, 0.20 mmol, 1.0 equiv) in 6:1:1 iPrOH:H_2O:THF (2.0 ml) was added sodium borohydride (45.4 mg, 1.2 mmol, 6 equiv) in one portion at room temperature. The mixture was stirred at room temperature overnight. The reaction media was then quenched with HCl 2.0 N, extracted with ethyl acetate, and concentrated under reduced pressure. The resulting residue was then re-suspended in diethyl ether (5 mL) to which anhydrous HCl (4M in dioxane, 1.0 ml) was added. The reaction mixture was stirred at room temperature until a fine white ppt developed (ca 4 hours), then concentrated under reduced pressure. The solid was washed with 3:1 ether (contains phthalide) and discarded, then 3:1 acetone:MeOH (contains the product) which was then concentrated under reduced pressure. The off-white solid was then triturated with ether to afford the **pure amino alcohol 54** as white solid (63.2 mg, 91% yield, 20:1 dr, 99% ee). *Note: The enantioselectivity was determined on the Boc protected product due to high polarity of aminoalcohol. The aminoalcohol salt was dissolved in DCM. Et_3N (3.0 equiv) was subsequently added to the solution and stirred at room temperature for 5 mins. Boc_2O was then added to the above solution. The mixture was stirred at room temperature for 1 hour before purified by flash chromatography (SiO_2, 0-35% acetone in hexane) to give the Boc-protected product as clear oil (quant. yield)*

$[\alpha]^{20}_D$: 24.7° (c = 1.07 g/100 ml, MeOH, 99% ee)

^{1}H NMR (600 MHz, CD_3OD) δ 7.42 (d, J = 8.4 Hz, 2H), 7.11 (d, J = 8.4 Hz, 2H), 3.69 – 3.66 (m, 1H), 3.66 – 3.55 (m, 2H), 2.63 (ddd, J = 10.6, 7.5, 4.9 Hz, 1H), 2.56 – 2.43 (m, 2H), 2.36 (ddd, J = 11.7, 7.6, 4.5 Hz, 1H), 2.27 (ddd, J = 12.3, 7.6, 5.0 Hz, 1H), 2.12 (dd, J = 11.6, 8.5 Hz, 1H), 2.03 (dd, J = 11.9, 8.5 Hz, 1H), 1.92 (dd, J = 11.1, 8.6 Hz, 1H).

^{13}C NMR (151 MHz, CD_3OD) δ 142.3, 132.3, 131.4, 121.0, 65.3, 56.0, 42.5, 41.3, 41.1, 40.4, 39.7, 34.7, 33.3.

HRMS (+p APCI) calcd. for [$C_{15}H_{21}ON^{79}Br$] ([M+H]$^+$) 310.0801 found 310.0804.

SFC (CEL1, 5% (50% methanol in isopropanol with 0.2% Formic Acid) in CO_2, 2.5 mL/min, 1.0 mg/ml, UV 230 nm) retention times of 2.37 min (major) and 3.11 min (minor) 99% ee. (Analyzed on the Boc protected starting material)

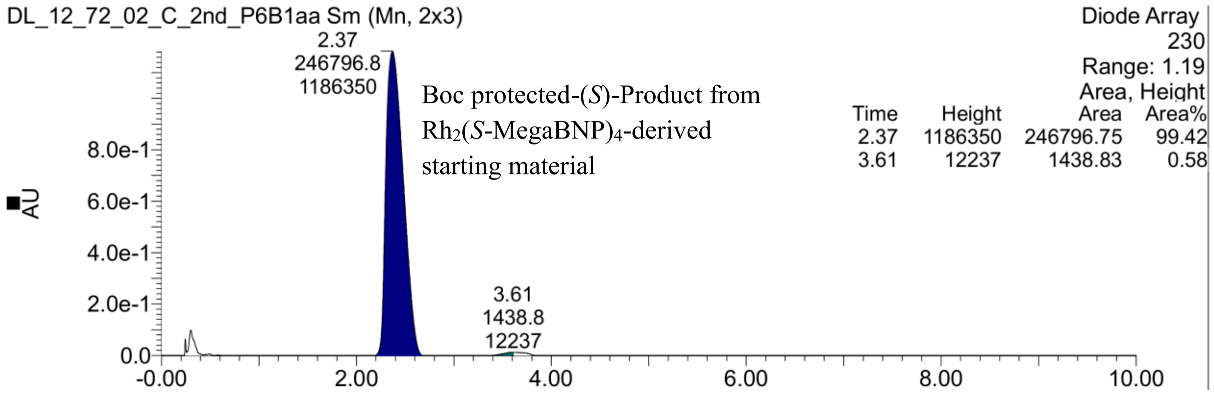

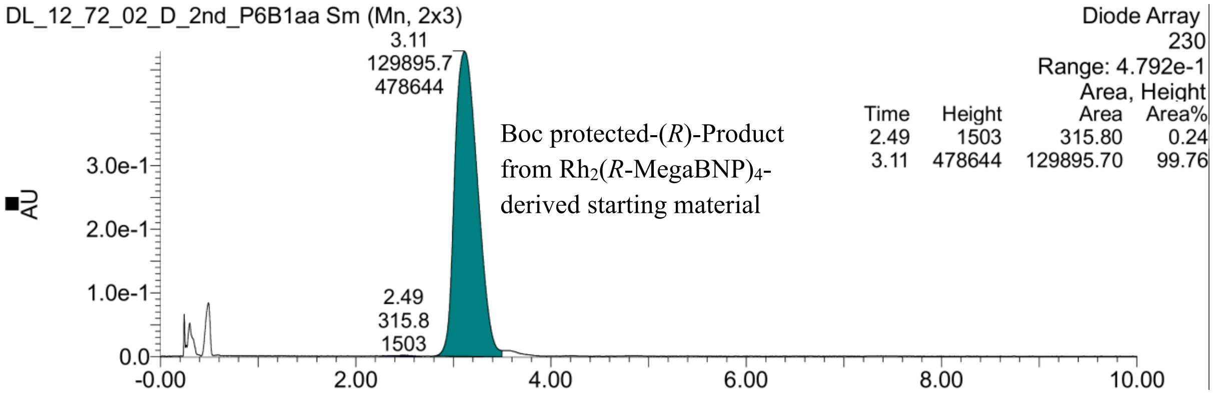

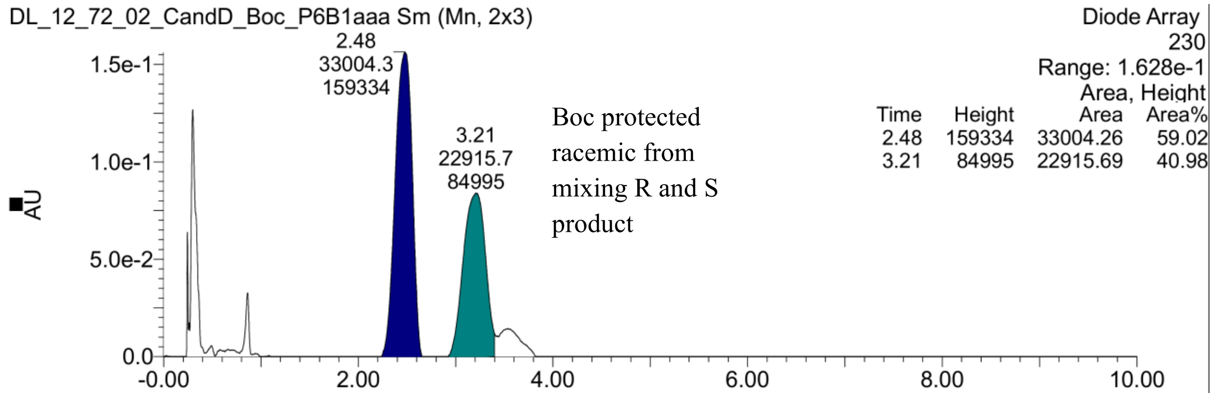

20250930-DL-12-72-02-C-Clean.10.fid

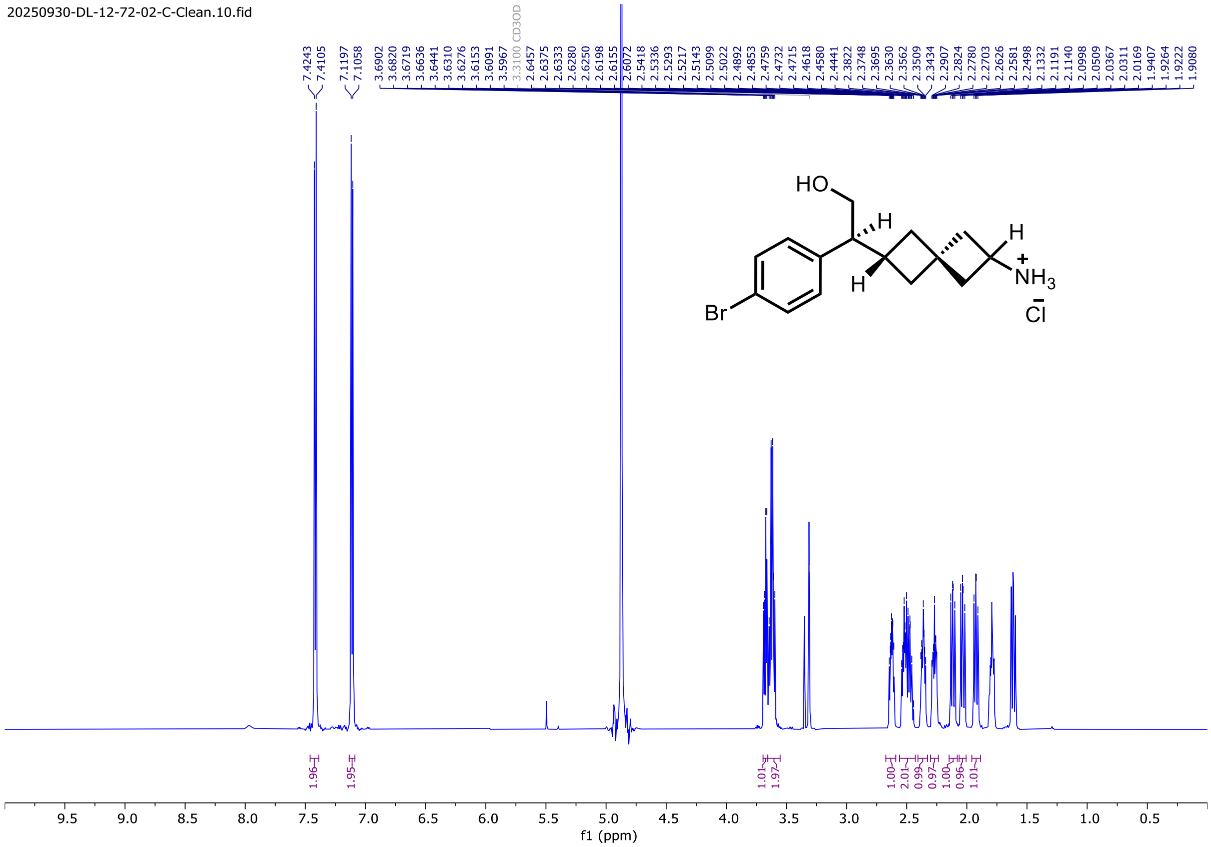

20250930-DL-12-72-02-C-Clean.11.fid

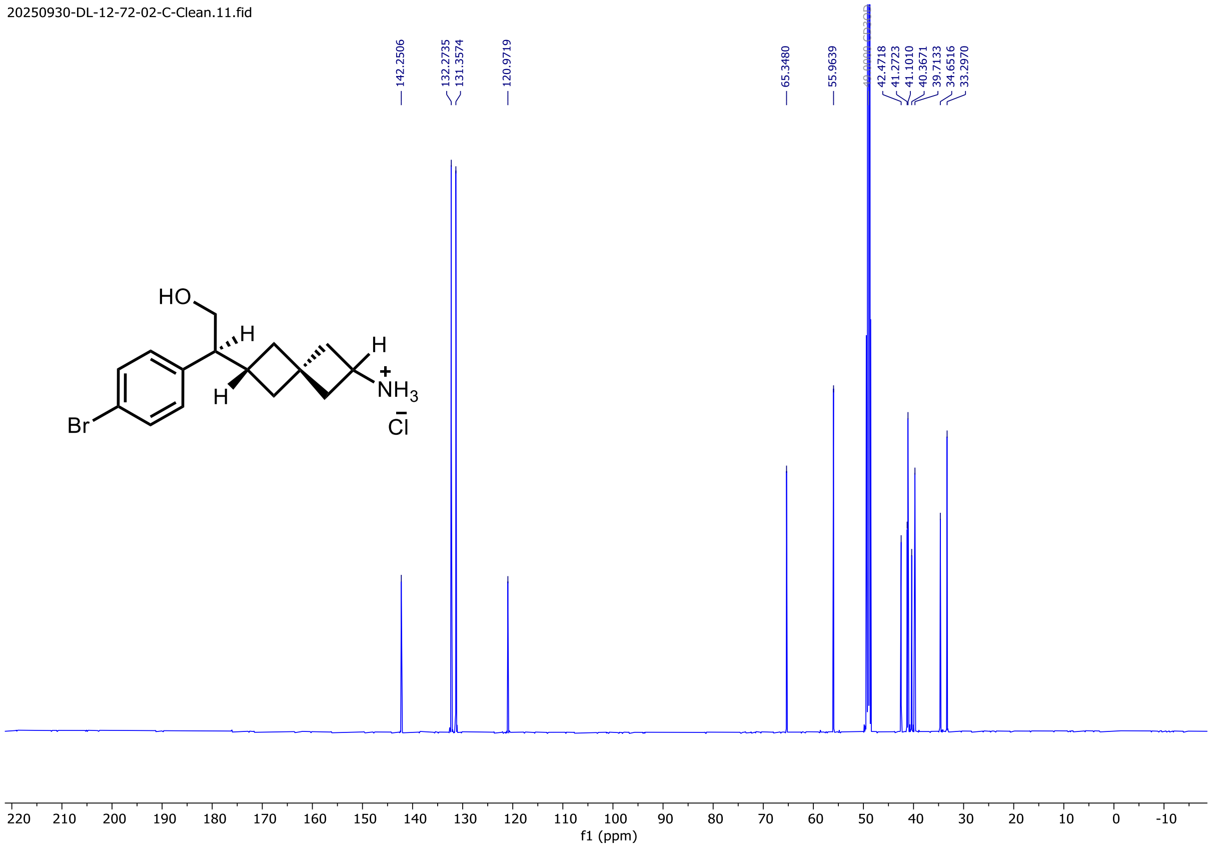

8. Xray Crystallography

R_1=3.10%

Compound 37

Crystal Data and Experimental

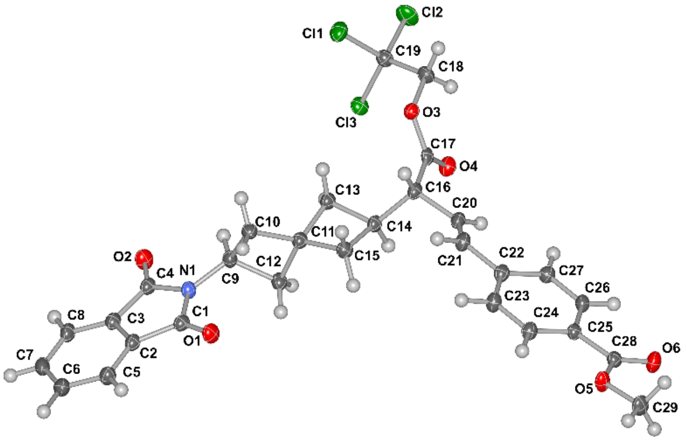

Experimental. Single colourless plate-shaped crystals of **COMPOUND 37** recrystallised from a mixture of heptane and chloroform by slow evaporation. A suitable crystal with dimensions 0.29 × 0.07 × 0.01 mm was selected and mounted on a loop with paratone on a XtaLAB Synergy, Dualflex, HyPix diffractometer. The crystal was kept at a steady T = 100(1) K during data collection. The structure was solved with ShelXT 2018/2 (Sheldrick, 2018) and Olex2 1.5-alpha (Dolomanov et al., 2009). The model was refined with olex2.refine 1.5-alpha (Bourhis et al., 2015) using full matrix least squares minimisation on $|F|^2$.

Crystal Data. $C_{29}H_{26}NO_6Cl_3$, M_r = 590.890, orthorhombic, $P2_12_12_1$ (No. 19), a = 5.8734(1) Å, b = 18.0573(3) Å, c = 25.5461(6) Å, $\alpha = \beta = \gamma = 90°$, V = 2709.36(9) Å^3, T = 100(1) K, Z = 4, Z' = 1, μ(Cu K_α) = 3.448, 19573 reflections measured, 5482 unique (R_{int} = 0.0572) which were used in all calculations. The final wR_2 was 0.0621 (all data) and R_1 was 0.0310 (I≥2σ(I)).

**Compound**	**COMPOUND 37**
Formula	$C_{29}H_{26}NO_6Cl_3$
$D_{calc.}$/ g cm^{-3}	1.449
μ/mm^{-1}	3.448
Formula Weight	590.890
Colour	colourless
Shape	plate-shaped
Size/mm	0.29×0.07×0.01
T/K	100(1)
Crystal System	orthorhombic
Flack Parameter	-0.006(8)
Hooft Parameter	-0.006(8)
Space Group	$P2_12_12_1$
a/Å	5.8734(1)
b/Å	18.0573(3)
c/Å	25.5461(6)
α/°	90
β/°	90
γ/°	90
V/Å^3	2709.36(9)
Z	4
Z'	1
Wavelength/Å	1.54184
Radiation type	Cu K_α
Θ_{min}/°	3.00
Θ_{max}/°	76.94
Index range h	-7 ≥ h ≥ 7
Index range k	-22 ≥ k ≥ 22
Index range l	-25 ≥ l ≥ 31
Measured Refl's.	19573
Indep't Refl's	5482
Refl's I≥2σ(I)	4891
R_{int}	0.0572
Parameters	646
Restraints	501
Largest Peak/eÅ^3	0.2154
Deepest Hole/eÅ^3	-0.2331
GooF	1.0386
R_1 (I≥2σ(I) / all)	0.0310 / 0.0382
wR_2 (I≥2σ(I) / all)	0.0601 / 0.0621

Structure Quality Indicators

**Reflections:**	d min (CuKα) 2Θ=153.9° 0.79	I/σ(I) 19.3	Rint m=3.56 5.72%	Full 135.4° 97% to 153.9° 99.8
**Refinement:**	Shift -0.000	Max Peak 0.2	Min Peak -0.2	GooF 1.039
	Hooft -.006(8)			

A colourless plate-shaped crystal with dimensions 0.29 × 0.07 × 0.01 mm was mounted on a loop with paratone. Data were collected using a XtaLAB Synergy, Dualflex, HyPix diffractometer equipped with an Oxford Cryosystems low-temperature device operating at T = 100(1) K.

Data were measured using ω scans with Cu K_α radiation. The diffraction pattern was indexed and the total number of runs and images was based on the strategy calculation from the program CrysAlis^{Pro} system (CCD 44.57a 64-bit (release 20-06-2024)). The maximum resolution achieved was Θ = 76.94° (0.79 Å).

The unit cell was refined using CrysAlis^{Pro} on 7183 reflections, 37% of the observed reflections.

Data reduction, scaling and absorption corrections were performed using CrysAlis^{Pro}. The final completeness is 99.96 % out to 76.94° in Θ. A numerical absorption correction based on gaussian integration over a multifaceted crystal model was performed using CrysAlisPro 1.171.42.74a (Rigaku Oxford Diffraction, 2022). An empirical absorption correction using spherical harmonics, implemented in SCALE3 ABSPACK scaling algorithm was also applied. The absorption coefficient μ of this material is 3.448 mm^{-1} at this wavelength (λ = 1.54184Å) and the minimum and maximum transmissions are 0.363 and 1.000.

The structure was solved in the space group $P2_12_12_1$ (# 19) by ShelXT 2018/2 (Sheldrick, 2018) using dual methods. It was refined by full matrix least squares minimisation on $|\boldsymbol{F}|^2$ using version of olex2.refine 1.5-alpha (Bourhis et al., 2015). All atoms, including hydrogens, were refined anisotropically. Most hydrogen atom positions were refined freely but some were refined using the riding model together with the Hirshfeld model. Refinement was by using NoSpherA2, an implementation of non-spherical atom-form-factors (F. Kleemiss, H. Puschmann, O. Dolomanov, S.Grabowsky - https://doi.org/10.1039/D0SC05526C – 2020). NoSpherA2 implementation of HAR makes use of tailor-made aspherical atomic form factors calculated from a Hirshfeld-partitioned electron density (ED) not from spherical-atom form factors. The ED was calculated from a Gaussian basis set single determinant SCF wavefunction from DFT using selected functionals for a fragment of this crystal. This fragment was embedded in an electrostatic crystal field by employing cluster charges. SOFTWARE: ORCA 5.0 PARTITIONING: NoSpherA2 INT ACCURACY: Normal METHOD: PBE BASIS SET: def2-SVP CHARGE: 0 MULTIPLICITY: 1 DATE: 2025-08-21_11-20-23

There is a single formula unit in the asymmetric unit (Z′ = 1) consistent with the empirical formula $C_{29}H_{26}Cl_3NO_6$.

The Flack parameter was refined to a value of -0.006(8). Determination of absolute structure using Bayesian statistics on Bijvoet differences using the Olex2 results in -0.006(8). Note: The Flack parameter is normally used to determine the absolute structure of a chiral crystal structure. Ideally, its value should be close to 0. A value of 1 indicates that the stereochemistry is incorrect, and the model should be inverted. The chiral atoms in this structure are: C14(R), C16(R).

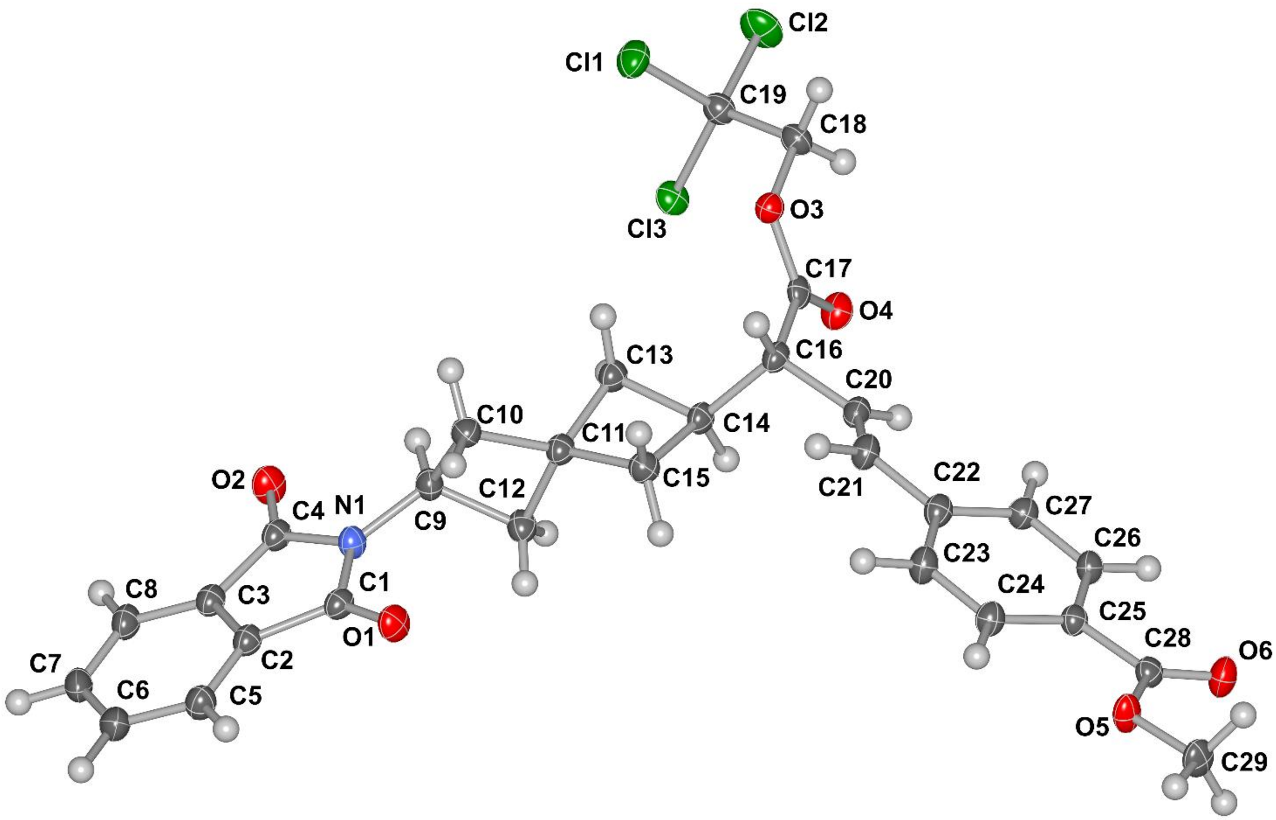

Molecular drawing of the structure with the C, Cl, O and N atoms shown as thermal ellipsoids at the 50% probability level. There is one crystallographically independent molecule in the asymmetric unit. The chiral atoms in this structure are: C14(R), C16(R).

Data Plots: Diffraction Data

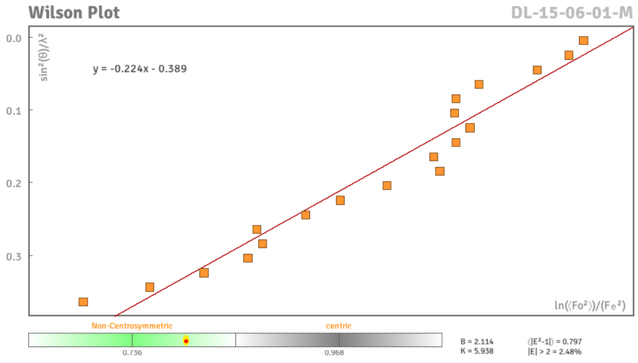

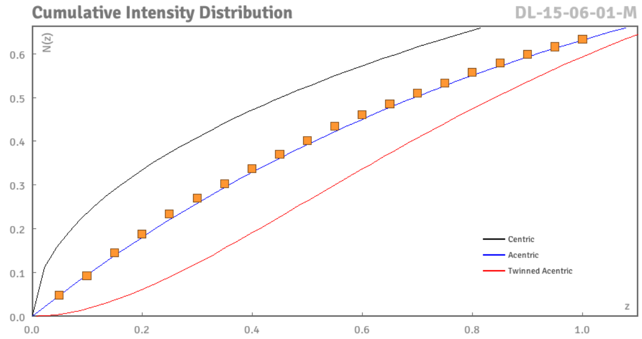

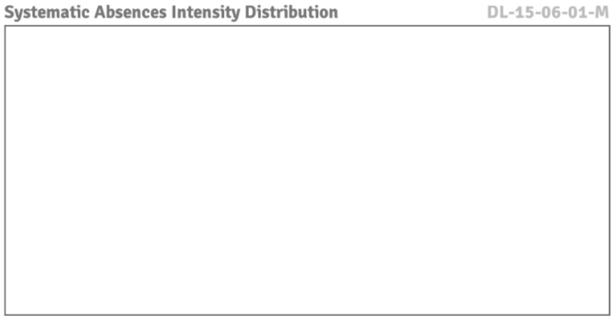

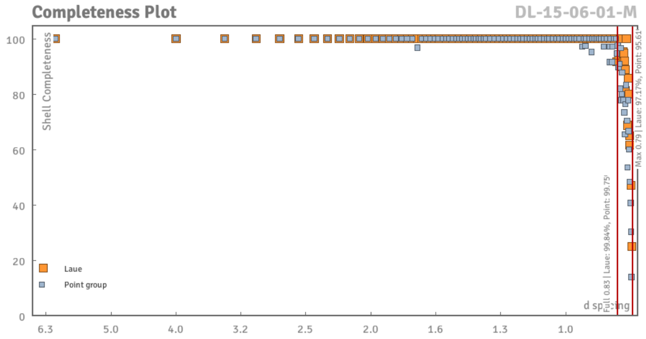

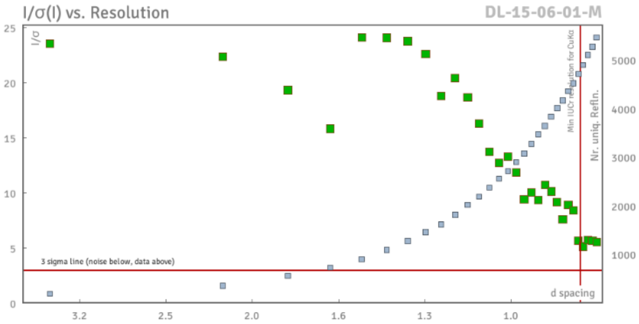

Data Plots: Refinement and Data

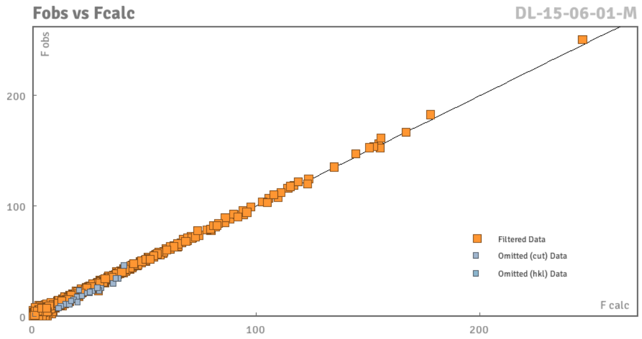

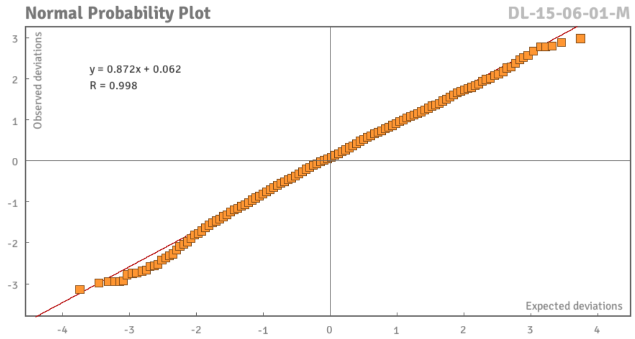

Reflection Statistics

Total reflections (after filtering)	19534	Unique reflections	5482
Completeness	0.956	Mean I/σ	14.06
hkl_{max} collected	(7, 22, 31)	hkl_{min} collected	(-7, -22, -25)
hkl_{max} used	(7, 22, 31)	hkl_{min} used	(-7, 0, 0)
Lim d_{max} collected	100.0	Lim d_{min} collected	0.77
d_{max} used	14.75	d_{min} used	0.79
Friedel pairs	2375	Friedel pairs merged	0
Inconsistent equivalents	1	R_{int}	0.0572
R_{sigma}	0.0517	Intensity transformed	0
Omitted reflections	0	Omitted by user (OMIT hkl)	39
Multiplicity	(5855, 3142, 1394, 469, 163, 76, 15)	Maximum multiplicity	11
Removed systematic absences	0	Filtered off (Shel/OMIT)	0

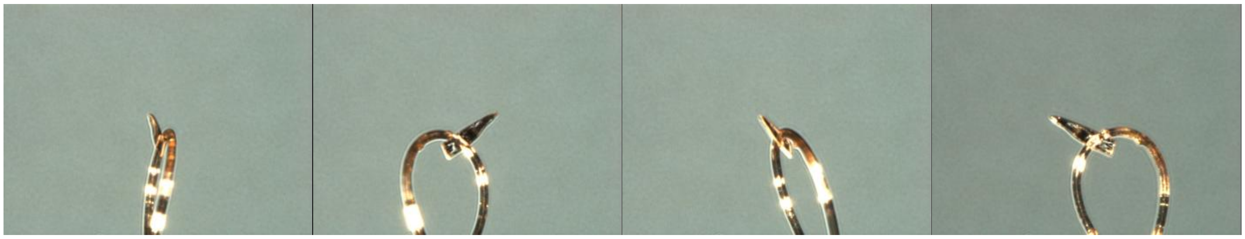

Table S3: Fractional Atomic Coordinates (×10^4) and Equivalent Isotropic Displacement Parameters (Å^2×10^3) for **COMPOUND 37**. U_{eq} is defined as 1/3 of the trace of the orthogonalised U_{ij}.

Atom	x	y	z	U_{eq}
Cl3	-1412.2(19)	4289.4(6)	7994.6(4)	23.3(3)
Cl1	1501(2)	5561.7(7)	8008.9(5)	29.8(3)
Cl2	-2930(2)	5659.4(7)	7538.2(5)	36.5(4)

Atom	x	y	z	U_{eq}
O3	2236(2)	4375.5(7)	7133.4(5)	19.0(3)
O4	-59(2)	3431.7(8)	6895.7(5)	24.7(3)
O1	9424(3)	1138.1(8)	8965.8(5)	25.7(3)
O6	6578(2)	720.1(8)	4079.9(5)	26.7(3)
O2	4090(3)	2049.1(8)	10106.1(5)	27.9(3)
O5	10052(2)	543.0(8)	4420.4(5)	24.3(3)
N1	6462(3)	1683.8(8)	9427.3(6)	19.2(3)
C1	8416(3)	1249.2(10)	9373.9(7)	19.4(4)
C4	5708(3)	1699.6(10)	9947.3(7)	19.6(4)
C26	5291(3)	1559.0(11)	4977.9(8)	19.3(4)
C17	1823(3)	3663.0(9)	6991.0(7)	18.0(4)
C2	8927(3)	950.3(10)	9902.9(7)	19.7(4)
C28	7910(3)	802.0(10)	4435.9(7)	18.4(4)
C25	7400(3)	1201.7(10)	4928.2(7)	17.6(4)
C24	8997(3)	1262.0(11)	5329.8(7)	21.4(4)
C13	3579(3)	3000.2(10)	8015.7(7)	20.4(4)
C20	4109(3)	2764.2(10)	6483.2(7)	20.0(4)
C22	6428(3)	2051.0(10)	5823.0(7)	20.2(4)
C18	359(3)	4871.5(11)	7097.8(8)	22.1(4)
C27	4814(3)	1979.6(10)	5420.2(7)	20.4(4)
C14	3891(3)	2675.7(10)	7458.2(7)	19.1(4)
C3	7273(3)	1207.9(10)	10246.0(7)	20.4(4)
C6	10690(4)	297.2(12)	10602.1(8)	27.5(4)
C5	10672(4)	489.8(11)	10071.7(8)	24.8(4)
C16	3956(3)	3201.7(9)	6985.6(7)	18.1(4)
C21	6083(4)	2512.6(11)	6292.2(8)	22.6(4)
C11	5417(3)	2453.1(10)	8213.2(7)	19.3(4)
C10	6963(3)	2645.3(10)	8682.2(8)	21.5(4)
C23	8507(4)	1679.6(11)	5771.3(8)	23.4(4)
C12	4555(4)	1769.4(11)	8520.3(8)	22.0(4)
C8	7261(4)	1014.3(11)	10769.3(8)	24.0(4)
C9	5449(3)	2138.1(10)	9023.3(7)	20.1(4)
C15	6204(4)	2349.4(11)	7642.8(7)	21.8(4)
C29	10754(4)	211.5(12)	3939.4(8)	27.4(5)
C7	9003(4)	547.7(11)	10944.2(8)	26.1(4)
C19	-546(3)	5078.5(11)	7637.0(8)	21.9(4)

Table S4: Anisotropic Displacement Parameters (×10^4) for **COMPOUND 37.** The anisotropic displacement factor exponent takes the form: $-2\pi^2[h^2a^{*2} \times U_{11} + ... + 2hka^* \times b^* \times U_{12}]$

Atom	U_{11}	U_{22}	U_{33}	U_{23}	U_{13}	U_{12}
Cl3	22.5(6)	27.4(7)	20.0(6)	-1.2(6)	7.1(5)	2.0(5)
Cl1	33.4(7)	30.8(7)	25.4(7)	-7.5(6)	-0.4(6)	-2.8(6)
Cl2	28.6(7)	39.9(8)	41.1(9)	16.8(6)	3.3(6)	5.5(7)
O3	19.2(6)	20.8(6)	16.9(7)	-1.1(5)	0.9(5)	-1.3(5)
O4	19.0(7)	30.3(7)	24.6(8)	-4.5(5)	-0.2(5)	-3.4(6)
O1	27.8(7)	32.9(7)	16.5(7)	5.0(6)	7.2(6)	1.2(6)
O6	23.8(7)	39.6(7)	16.7(7)	2.3(6)	-2.2(5)	-8.1(6)
O2	26.6(8)	37.0(7)	20.1(7)	8.7(6)	7.3(6)	1.1(6)
O5	21.8(7)	36.0(8)	15.3(6)	8.2(5)	1.3(5)	-3.6(6)
N1	18.9(8)	23.5(7)	15.2(7)	1.7(6)	2.4(6)	-0.3(6)
C1	20.8(9)	23.0(8)	14.3(9)	-0.4(7)	3.9(6)	-1.2(6)
C4	21.1(9)	23.3(8)	14.5(9)	1.0(6)	4.3(7)	-0.2(7)
C26	18.1(9)	25.6(9)	14.2(9)	2.0(7)	-0.4(7)	-2.8(7)
C17	17.0(9)	23.3(8)	13.6(8)	-2.5(6)	0.4(7)	0.9(6)
C2	21.0(9)	23.4(8)	14.9(9)	0.4(7)	1.9(6)	-0.6(6)

Atom	U_{11}	U_{22}	U_{33}	U_{23}	U_{13}	U_{12}
C28	19.8(8)	22.9(8)	12.5(8)	0.9(6)	0.7(6)	-1.2(6)
C25	16.4(9)	22.8(8)	13.6(9)	1.6(6)	0.2(6)	-2.6(6)
C24	18.6(10)	29.0(9)	16.6(9)	1.2(7)	-0.3(6)	-4.7(7)
C13	22.7(9)	22.9(8)	15.6(9)	-0.2(7)	2.2(7)	-1.4(7)
C20	21.1(10)	22.7(9)	16.2(10)	-1.1(7)	1.8(7)	-4.0(7)
C22	18.8(9)	25.9(8)	16.0(9)	-2.2(8)	1.3(7)	-3.7(7)
C18	22.1(10)	22.6(9)	21.5(10)	1.8(7)	-0.7(7)	2.3(7)
H18a	20(7)	26(4)	29(7)	2(2)	-2(3)	6(2)
H18b	30(5)	43(7)	37(7)	-4(2)	-5(2)	-6(3)
C27	19.2(10)	24.4(9)	17.7(10)	1.1(7)	0.1(7)	-4.5(7)
C14	22.1(9)	22.0(8)	13.2(8)	-1.0(7)	2.9(6)	-1.5(6)
C3	23.3(10)	24.0(8)	14.0(9)	-0.6(6)	2.0(6)	0.6(6)
C6	33.2(11)	29.1(10)	20.2(10)	3.4(8)	-4.1(7)	-1.1(7)
C5	25.9(10)	29.3(10)	19.1(10)	5.8(7)	-1.3(7)	-0.8(7)
C16	20.1(10)	20.7(8)	13.6(8)	-2.6(7)	1.9(8)	-2.3(7)
C21	19.2(10)	30.1(9)	18.4(10)	-0.9(7)	0.9(7)	-6.3(7)
C11	21.3(9)	21.3(8)	15.3(9)	-1.9(6)	1.0(6)	-0.7(6)
C10	23.1(10)	21.1(8)	20.4(10)	-2.3(7)	-2.4(7)	1.3(7)
C23	19.2(10)	33.0(9)	17.9(9)	1.6(8)	-0.1(8)	-5.7(7)
C12	23.9(11)	24.2(9)	17.9(9)	-3.9(7)	-0.9(7)	2.3(7)
C8	29.3(11)	28.9(9)	13.8(9)	-1.4(7)	0.9(7)	0.9(7)
C9	20.3(9)	23.5(8)	16.5(9)	0.5(7)	-1.7(6)	0.0(6)
H9	25(4)	26(4)	26(4)	2.0(14)	2.1(14)	-0.4(14)
C15	23.9(10)	23.1(9)	18.5(9)	2.5(7)	2.7(7)	0.3(7)
C29	29.8(12)	34.6(11)	17.8(10)	6.6(9)	5.2(8)	-3.7(8)
H29a	50(7)	50(5)	41(9)	-7(3)	5(3)	-10(3)
H29b	53(9)	49(6)	29(5)	7(3)	5(3)	6(2)
H29c	36(3)	59(9)	32(9)	15(2)	4.6(19)	-5(4)
C7	34.5(11)	27.7(9)	16.2(10)	0.8(7)	-3.7(7)	2.3(7)
C19	21.0(10)	23.4(9)	21.5(10)	2.5(7)	2.5(7)	1.8(6)
H24	25(4)	55(13)	22(10)	14(4)	-6(3)	-16(6)
H15a	28(9)	23(3)	23(9)	1.5(18)	8(4)	0.4(18)
H16	19(6)	19(6)	20(6)	-1.7(19)	0.0(19)	-0.2(19)
H10a	24(6)	22(2)	16(6)	-1.8(13)	-3(3)	2.4(13)
H14	26(6)	24(5)	29(8)	-4(3)	-1(3)	-1(3)
H12a	25(2)	23(6)	23(7)	-3.8(13)	-1.8(13)	4(3)
H12b	34(6)	29(4)	27(9)	3(2)	-3(3)	-1(2)
H13a	25(3)	44(9)	28(9)	-2(2)	7(2)	-3(4)
H21	23(4)	85(18)	50(10)	9(3)	-9(3)	-45(7)
H13b	20(7)	23(2)	22(9)	1.2(16)	4(4)	-2.5(16)
H5	47(9)	65(15)	27(6)	31(6)	9(3)	8(4)
H26	25(6)	40(12)	22(6)	8(5)	-8(3)	-10(4)
H23	25(7)	56(16)	29(8)	10(6)	-8(4)	-20(6)
H27	24(5)	44(12)	37(12)	11(4)	-9(4)	-23(7)
H6	39(8)	41(12)	27(9)	11(5)	-1(3)	6(4)
H7	55(12)	47(13)	18(3)	11(6)	-2(2)	7(2)
H20	23(4)	60(16)	37(12)	0(3)	-3(3)	-25(7)
H8	53(10)	74(17)	23(7)	27(7)	13(4)	9(5)
H15b	28(5)	30(6)	29(9)	-2(2)	4(3)	2(3)
H10b	25(3)	32(7)	33(10)	2.0(18)	-2.5(19)	-1(4)

Table S5: Bond Lengths in Å for **COMPOUND 37**.

Atom	Atom	Length/Å
Cl3	C19	1.767(2)
Cl1	C19	1.763(2)
Cl2	C19	1.767(2)
O3	C17	1.359(2)

Atom	Atom	Length/Å
O3	C18	1.424(2)
O4	C17	1.206(2)
O1	C1	1.216(2)
O6	C28	1.209(2)
O2	C4	1.211(2)
O5	C28	1.343(2)
O5	C29	1.428(2)
N1	C1	1.397(2)
N1	C4	1.401(2)
N1	C9	1.447(2)
C1	C2	1.486(3)
C4	C3	1.488(3)
C26	C25	1.403(3)
C26	C27	1.390(3)
C26	H26	1.09(2)
C17	C16	1.505(3)
C2	C3	1.389(3)
C2	C5	1.389(3)
C28	C25	1.480(3)
C25	C24	1.394(3)
C24	C23	1.387(3)
C24	H24	1.10(2)
C13	C14	1.551(3)
C13	C11	1.548(3)
C13	H13a	1.09(2)
C13	H13b	1.09(2)
C20	C16	1.510(3)
C20	C21	1.337(3)
C20	H20	1.05(2)
C22	C27	1.405(3)
C22	C21	1.474(3)
C22	C23	1.399(3)
C18	H18a	1.081(15)

Atom	Atom	Length/Å
C18	H18b	1.081(15)
C18	C19	1.523(3)
C27	H27	1.10(2)
C14	C16	1.537(3)
C14	C15	1.554(3)
C14	H14	1.12(2)
C3	C8	1.382(3)
C6	C5	1.399(3)
C6	C7	1.397(3)
C6	H6	1.11(2)
C5	H5	1.05(2)
C16	H16	1.10(2)
C21	H21	1.09(3)
C11	C10	1.543(3)
C11	C12	1.548(3)
C11	C15	1.540(3)
C10	C9	1.546(3)
C10	H10a	1.07(2)
C10	H10b	1.13(3)
C23	H23	1.13(2)
C12	C9	1.539(3)
C12	H12a	1.10(2)
C12	H12b	1.07(2)
C8	C7	1.399(3)
C8	H8	1.14(3)
C9	H9	1.14(2)
C15	H15a	1.11(2)
C15	H15b	1.09(2)
C29	H29a	1.057(13)
C29	H29b	1.057(13)
C29	H29c	1.057(13)
C7	H7	1.07(2)

Table S6: Bond Angles in ° for **COMPOUND 37**.

Atom	Atom	Atom	Angle/°
C18	O3	C17	116.11(14)
C29	O5	C28	116.24(15)
C4	N1	C1	111.33(15)
C9	N1	C1	125.93(16)
C9	N1	C4	122.36(16)
N1	C1	O1	125.21(17)
C2	C1	O1	128.45(18)
C2	C1	N1	106.32(15)
N1	C4	O2	125.20(18)
C3	C4	O2	128.60(17)
C3	C4	N1	106.20(16)
C27	C26	C25	120.21(18)
H26	C26	C25	119.3(12)
H26	C26	C27	120.5(12)
O4	C17	O3	123.07(16)
C16	C17	O3	112.20(15)
C16	C17	O4	124.70(16)
C3	C2	C1	108.13(17)
C5	C2	C1	130.47(18)
C5	C2	C3	121.39(18)

Atom	Atom	Atom	Angle/°
O5	C28	O6	122.79(17)
C25	C28	O6	124.61(17)
C25	C28	O5	112.60(16)
C28	C25	C26	118.67(17)
C24	C25	C26	119.45(17)
C24	C25	C28	121.81(17)
C23	C24	C25	120.06(18)
H24	C24	C25	119.5(11)
H24	C24	C23	120.5(11)
C11	C13	C14	88.61(14)
H13a	C13	C14	115.6(12)
H13a	C13	C11	119.2(12)
H13b	C13	C14	111.9(11)
H13b	C13	C11	112.3(11)
H13b	C13	H13a	108.2(16)
C21	C20	C16	122.67(18)
H20	C20	C16	116.2(13)
H20	C20	C21	121.0(13)
C21	C22	C27	123.69(18)
C23	C22	C27	118.40(17)

Atom	Atom	Atom	Angle/˚
C23	C22	C21	117.89(17)
H18a	C18	O3	109.32(9)
H18b	C18	O3	109.32(10)
H18b	C18	H18a	108.0
C19	C18	O3	111.52(15)
C19	C18	H18a	109.32(10)
C19	C18	H18b	109.32(11)
C22	C27	C26	120.62(18)
H27	C27	C26	121.5(11)
H27	C27	C22	117.8(11)
C16	C14	C13	119.40(15)
C15	C14	C13	88.15(14)
C15	C14	C16	116.82(16)
H14	C14	C13	109.0(11)
H14	C14	C16	109.1(11)
H14	C14	C15	113.0(11)
C2	C3	C4	107.95(16)
C8	C3	C4	130.07(18)
C8	C3	C2	121.98(18)
C7	C6	C5	121.3(2)
H6	C6	C5	119.3(12)
H6	C6	C7	119.3(12)
C6	C5	C2	117.09(19)
H5	C5	C2	119.4(13)
H5	C5	C6	123.5(13)
C20	C16	C17	110.32(16)
C14	C16	C17	108.32(15)
C14	C16	C20	110.24(14)
H16	C16	C17	108.9(11)
H16	C16	C20	108.5(11)
H16	C16	C14	110.6(11)
C22	C21	C20	127.44(19)
H21	C21	C20	118.4(13)
H21	C21	C22	114.1(13)
C10	C11	C13	121.34(15)
C12	C11	C13	116.48(16)
C12	C11	C10	88.76(14)
C15	C11	C13	88.76(14)
C15	C11	C10	125.85(17)
C15	C11	C12	118.73(16)
C9	C10	C11	88.03(14)
H10a	C10	C11	115.8(11)
H10a	C10	C9	118.6(11)

Atom	Atom	Atom	Angle/˚
H10b	C10	C11	113.3(12)
H10b	C10	C9	110.4(12)
H10b	C10	H10a	109.4(17)
C22	C23	C24	121.25(18)
H23	C23	C24	119.2(12)
H23	C23	C22	119.3(12)
C9	C12	C11	88.08(14)
H12a	C12	C11	118.0(11)
H12a	C12	C9	117.9(11)
H12b	C12	C11	112.5(12)
H12b	C12	C9	110.2(12)
H12b	C12	H12a	108.8(17)
C7	C8	C3	117.23(19)
H8	C8	C3	121.7(13)
H8	C8	C7	121.1(13)
C10	C9	N1	120.09(16)
C12	C9	N1	119.38(16)
C12	C9	C10	88.97(14)
H9	C9	N1	108.92(10)
H9	C9	C10	108.92(10)
H9	C9	C12	108.92(11)
C11	C15	C14	88.79(14)
H15a	C15	C14	117.2(12)
H15a	C15	C11	115.5(11)
H15b	C15	C14	111.3(12)
H15b	C15	C11	109.1(12)
H15b	C15	H15a	112.6(17)
H29a	C29	O5	109.5
H29b	C29	O5	109.5
H29b	C29	H29a	109.5
H29c	C29	O5	109.5
H29c	C29	H29a	109.5
H29c	C29	H29b	109.5
C8	C7	C6	120.95(18)
H7	C7	C6	117.7(14)
H7	C7	C8	121.3(14)
Cl1	C19	Cl3	108.47(12)
Cl2	C19	Cl3	108.93(12)
Cl2	C19	Cl1	108.87(12)
C18	C19	Cl3	111.72(14)
C18	C19	Cl1	111.76(14)
C18	C19	Cl2	107.03(14)

Table S7: Torsion Angles in ˚ for **COMPOUND 37**.

Atom	Atom	Atom	Atom	Angle/˚
Cl3	C19	C18	O3	-58.37(14)
Cl1	C19	C18	O3	63.37(14)
Cl2	C19	C18	O3	-177.53(12)
O3	C17	C16	C20	-132.01(15)
O3	C17	C16	C14	107.24(15)
O4	C17	O3	C18	-8.2(2)
O4	C17	C16	C20	50.0(2)
O4	C17	C16	C14	-70.8(2)
O1	C1	N1	C4	179.78(19)
O1	C1	N1	C9	-7.2(2)

Atom	Atom	Atom	Atom	Angle/˚
O1	C1	C2	C3	-178.0(2)
O1	C1	C2	C5	1.6(3)
O6	C28	O5	C29	4.9(2)
O6	C28	C25	C26	-4.7(2)
O6	C28	C25	C24	178.47(19)
O2	C4	N1	C1	177.9(2)
O2	C4	N1	C9	4.6(2)
O2	C4	C3	C2	-177.7(2)
O2	C4	C3	C8	1.4(3)
O5	C28	C25	C26	174.36(15)
O5	C28	C25	C24	-2.42(19)
N1	C1	C2	C3	0.53(16)
N1	C1	C2	C5	-179.87(15)
N1	C4	C3	C2	2.61(16)
N1	C4	C3	C8	-178.29(15)
N1	C9	C10	C11	142.5(2)
N1	C9	C12	C11	-143.0(2)
C1	N1	C4	C3	-2.33(16)
C1	N1	C9	C10	-45.7(2)
C1	N1	C9	C12	61.9(2)
C1	C2	C3	C4	-1.92(16)
C1	C2	C3	C8	178.90(15)
C1	C2	C5	C6	-179.5(2)
C4	N1	C1	C2	1.19(16)
C4	N1	C9	C10	126.64(17)
C4	N1	C9	C12	-125.76(18)
C4	C3	C2	C5	178.44(15)
C4	C3	C8	C7	-178.6(2)
C26	C25	C24	C23	-0.7(2)
C26	C27	C22	C21	177.27(18)
C26	C27	C22	C23	-1.0(2)
C17	O3	C18	C19	107.46(16)
C17	C16	C20	C21	156.85(16)
C17	C16	C14	C13	-55.34(17)
C17	C16	C14	C15	-159.43(14)
C2	C1	N1	C9	174.23(13)
C2	C3	C8	C7	0.4(2)
C2	C5	C6	C7	1.0(2)
C28	C25	C26	C27	-175.80(17)
C28	C25	C24	C23	176.01(18)
C25	C26	C27	C22	-0.2(2)
C25	C28	O5	C29	-174.27(16)
C25	C24	C23	C22	-0.5(2)
C24	C25	C26	C27	1.1(2)
C24	C23	C22	C27	1.3(2)
C24	C23	C22	C21	-177.05(19)
C13	C14	C16	C20	-176.15(17)
C13	C14	C15	C11	17.89(14)
C13	C11	C10	C9	102.05(19)
C13	C11	C12	C9	-106.15(18)
C13	C11	C15	C14	-17.92(14)
C20	C16	C14	C15	79.77(16)
C20	C21	C22	C27	19.2(3)
C20	C21	C22	C23	-162.5(2)
C22	C21	C20	C16	176.69(19)
C18	O3	C17	C16	173.78(15)
C14	C13	C11	C10	150.23(12)

Atom	Atom	Atom	Atom	Angle/°
C14	C13	C11	C12	-103.81(14)
C14	C13	C11	C15	17.96(14)
C14	C16	C20	C21	-83.55(18)
C14	C15	C11	C10	-146.68(12)
C14	C15	C11	C12	101.87(14)
C3	C4	N1	C9	-175.66(13)
C3	C2	C5	C6	0.0(2)
C3	C8	C7	C6	0.6(2)
C5	C2	C3	C8	-0.7(2)
C5	C6	C7	C8	-1.4(2)
C16	C14	C13	C11	-137.8(2)
C16	C14	C15	C11	140.17(18)
C11	C13	C14	C15	-17.79(14)
C11	C10	C9	C12	18.65(15)
C11	C12	C9	C10	-18.59(15)
C10	C11	C12	C9	18.63(15)
C12	C11	C10	C9	-18.55(15)
C9	C10	C11	C15	-143.83(12)
C9	C12	C11	C15	149.63(13)

Table S8: Hydrogen Fractional Atomic Coordinates (×10^4) and Equivalent Isotropic Displacement Parameters ($Å^2$×10^3) for **COMPOUND 37.** U_{eq} is defined as 1/3 of the trace of the orthogonalised U_{ij}.

Atom	x	y	z	U_{eq}
H18a	885(8)	5368(7)	6894(3)	25(4)
H18b	-987(19)	4615(4)	6873(3)	36(4)
H9	4030(30)	2485(7)	9204(4)	26(3)
H29a	9660(14)	-235(6)	3848.5(14)	47(4)
H29b	10681(4)	610(5)	3636(4)	44(4)
H29c	12440(20)	15(3)	3977.1(9)	42(4)
H24	10640(40)	970(14)	5295(9)	34(6)
H15a	6600(40)	1769(11)	7526(8)	25(4)
H16	5440(40)	3571(11)	7009(8)	19(5)
H10a	7070(40)	3219(11)	8779(8)	21(3)
H14	2560(40)	2241(12)	7390(8)	27(4)
H12a	2730(40)	1635(11)	8499(8)	24(3)
H12b	5520(40)	1280(12)	8436(9)	30(4)
H13a	1860(40)	2966(13)	8176(9)	32(4)
H21	7640(40)	2631(16)	6508(10)	53(8)
H13b	4140(30)	3574(11)	8039(8)	22(4)
H5	11900(50)	309(15)	9804(9)	46(7)
H26	4060(40)	1519(13)	4662(8)	29(6)
H23	9880(40)	1771(15)	6074(10)	37(7)
H27	3210(40)	2293(13)	5457(9)	35(6)
H6	12030(40)	-82(13)	10750(9)	36(6)
H7	9140(50)	395(14)	11347(9)	40(6)
H20	2550(40)	2632(15)	6302(9)	40(7)
H8	5890(50)	1220(16)	11047(10)	50(8)
H15b	7600(40)	2728(12)	7563(8)	29(4)
H10b	8730(40)	2407(12)	8647(9)	30(4)

Citations

CrysAlis^{Pro} (Rigaku Oxford Diffraction), Rigaku Oxford Diffraction, Poland (?).

L.J. Bourhis and O.V. Dolomanov and R.J. Gildea and J.A.K. Howard and H. Puschmann, The Anatomy of a Comprehensive Constrained, Restrained, Refinement Program for the Modern Computing Environment - Olex2 Disected, *Acta Cryst. A*, (2015), **A71**, 59-71.

O.V. Dolomanov and L.J. Bourhis and R.J. Gildea and J.A.K. Howard and H. Puschmann, Olex2: A complete structure solution, refinement and analysis program, *J. Appl. Cryst.*, (2009), **42**, 339-341.

Sheldrick, G.M., ShelXT-Integrated space-group and crystal-structure determination, *Acta Cryst.*, (2015), **A71**, 3-8.

9. Computational study

All calculations were performed using Gaussian-16 suite of programs.[4] The reported 3D structures were rendered using VMD[5]. Geometry and vibrational frequencies of the Rh_2(*S*-MegaBNP)$_4$ system with approximately over 450 atoms and their carbene complexes were calculated by using the two-layer ONIOM[6, 7] approach via partitioning of the complex Rh_2(*S*-MegaBNP)$_4$ into the two layers. (**Figure S8**) The highlighted catalyst structure, Rh-coordinated carbene fragment, and substrates were included in the model system (i.e. the first layer) and treated at the M06[8] level of theory in conjunction with Lanl2dz[9, 10] basis set for rhodium (Rh) and bromine (Br), and 6-31G(d,p)[11, 12] all-electron basis set for other atoms. The real system including all the atoms was calculated by using the molecular mechanics UFF[13] approach. The resulting approach is called the ONIOM(M06:UFF) approach. Frequency analyses and Gibbs free energy and zero-point energy corrections were calculated at 298.15K and 1 atm. The structure of rhodium carbene intermediate **I** was reproduced from *J. Am. Chem. Soc.* **2026**, 148, 2, 2709–2718 Copyright 2026 American Chemical Society.

ETS-NOCV[14], NCI-IGMH[15], and CHelpG[16] analyses were performed with Multiwfn 3.8(dev)[17] and visualized by VMD[5] using the default parameters and a grid resolution of 0.15 Bohr. The input for ETS-NOCV, NCI-IGMH, and CHelpG analyses were obtained from the computational output (.fchk) from Gaussian 16. sobEDAw[18] calculation was also performed with Multiwfn 3.8(dev)[17].

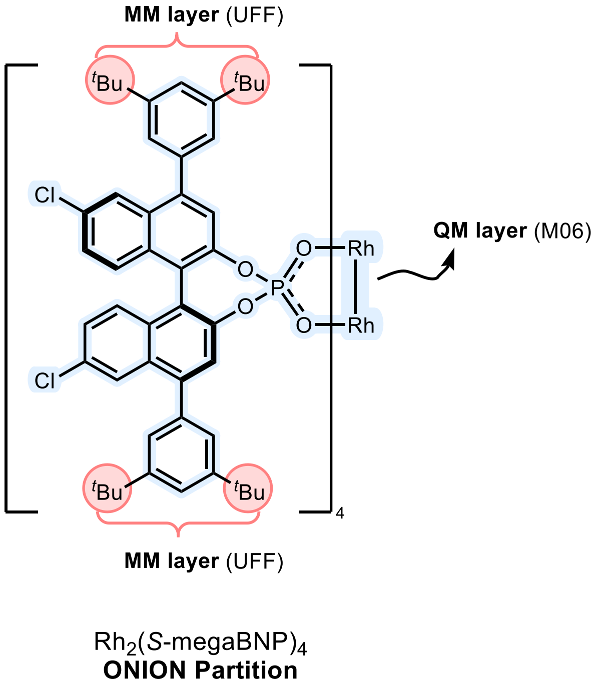

Figure S8. (A) ONIOM Partitioning scheme for the rest of study. The blue-highlighted atoms (QM layer) were treated at the M06 level, the rest (all 16 *t*-butyl groups, the second layer) highlighted in red circle were treated with UFF force fields (MM layer).

a. Analysis of two transition states

The key bond lengths in **TS1** and **TS2** are highlighted in **Figure S9**. Both transition states exhibit very similar geometries around the reactive center: the C(carbene)–H(substrate) and H(substrate)–C(substrate) bond lengths are 1.17 Å and 1.36 Å, respectively. This suggests minimal structural differences around the reactive center between **TS1** and **TS2**.

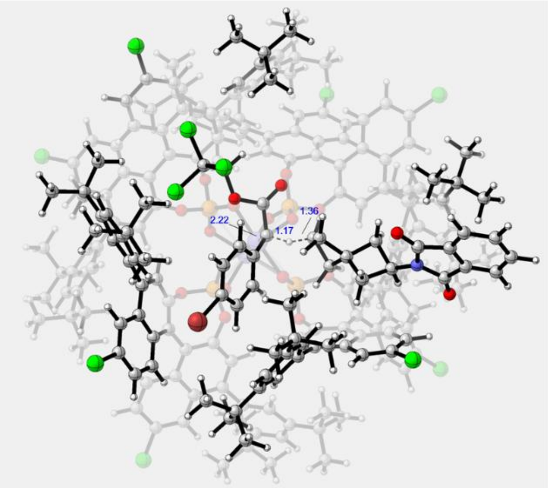

TS1
(0.0 kcal/mol)

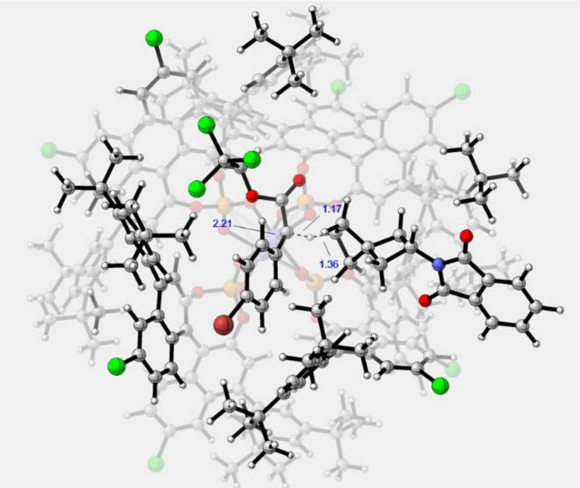

TS2
(1.7 kcal/mol)

Figure S9. The two transition states for the C-H functionalization leading to the two diastereomers. Highlight the key bond lengths.

Energy Decomposition Analysis (EDA)

The energy decomposition was applied here is a combination of an activation-strain model (ASM) by Houk-Bickelhaupt protocol and a sobEDAw model.[18, 19] The partitioning scheme are shown in **Figure S10**. The single-point energy calculations of each transition state and their separated fragments obtained from ONIOM(M06-UFF) were performed at the B3LYP-D3(BJ)/6-31G(d,p) (for all atoms) and Lan2ldz (for Rh and Br) level of theory (no solvation model). The results for ASM and sobEDAw models are shown in **Table S9**, **Table S10**.

The ASM model[19] separated the interaction of substrate and carbene fragments into distortion energy (ΔE_{dis}) and interaction energy (ΔE_{int}). The results shown that ΔE_{int} was a main component to make **TS1** more favorable than **TS2**. Therefore, sobEDAw method[18] was used to understand the interaction between the two fragments.

The sobEDAw method decomposed interaction between the fragments into four components: ΔE_{int} = ΔE_{els}(electrostatic) + ΔE_{xrep} (exchange-repulsion) + ΔE_{orb} (orbital) + ΔE_{disp} (dispersion). Particularly, ΔE_{els} represents columbic interactions between metal-carbene and substrate fragments; ΔE_{xrep} includes exchange interaction energy and electron-repulsion between metal-carbene and substrate fragments to satisfy Pauli exclusion principle. ΔE_{disp} shows the van der Waals interactions and other dispersion effects. The results of sobEDAw analysis showed that electronic, orbital, and dispersive interactions all contributed to stabilizing **TS1** while exchange-repulsion favored **TS2**. (**Table S10**)

Steric interaction energy (ΔE_{steric}) was calculated by combining the distortion energy (ΔE_{dis}) from ASM model with exchange-repulsion energy from sobEDAw model.(**Table S11**). The negative value of ΔE_{steric} shown that **TS2** is more favorable by less steric interaction compared to **TS1**.

CHelpG Charge

On one hand, CHelpG charge[16] of the substrate fragment was calculated to gain a deeper understanding about the electrostatic interaction component. The result shows that the substrate fragment charge in **TS1** is +0.312, while that number for **TS2** is +0.208. Although the transition state is neutral, substrate fragment in **TS1** is more polarized than in **TS2**, hence having a better columbic attraction.

ETS-NOCV analysis

On the other hand, The orbital interaction from sobEDAw (**Table S10**) was further studies by extended Transition State-Natural Orbital for Chemical Valance-method (ETS-NOCV)[14] method.(**Figure S11**) The dominant contribution to the ΔE_{orb} component in both transition states arises from the interaction between the substrate's σ(C–H) orbital and the π* orbital of the Rh–Rh–C superelectrophile.[20, 21] The stronger interaction in **TS1** relative to **TS2** is attributed to the ligand environment, which better orients the substrate for optimal orbital overlap.

NCI analysis

In addition, noncovalent interaction (NCI) analysis using the independent gradient model based on Hirshfeld partition of molecular density (IGMH)[15] were used to visualize the dispersive interaction in **TS1** and **TS2**. The results showed that there is much more dispersive interaction in **TS1** than **TS2**. (**Figure S12**)

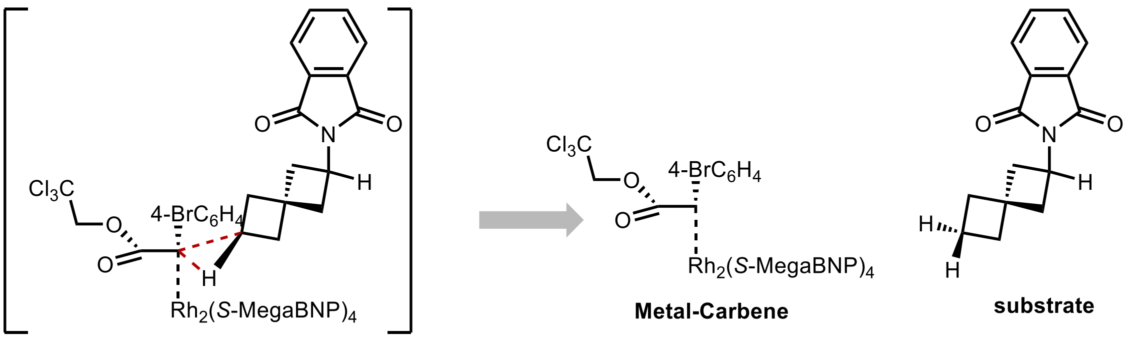

Figure S10. Partitioning scheme for activation strain model (ASM) and sobEDA analyses

Table S9. Distortion-interaction analysis. ΔE_{dis}, ΔE_{cat}, and ΔE_{sub} are differences (relative to that in **TS1**) in total distortion energy, distortion energy of metal-carbene fragment, and distortion energy of substrate fragment, respectively. The total distortion energy is defined as $\Delta E_{dis} = \Delta E_{sub} + \Delta E_{cat}$. $\Delta E_{int} = \Delta E_{TS} - \Delta E_{dis}$, and is difference in interaction energies. (All presented energies are in kcal/mol).

Structure	E_{TS} (hartree)	E_{cat} (hartree)	E_{sub}(hartree)	ΔE_{TS}	ΔE_{cat}	ΔE_{sub}	ΔE_{dis}	ΔE_{int}
**TS1**	-16623.51251	-15837.55593	-785.847273	0.0	0.0	0.0	0.0	0.0
**TS2**	-16623.50945	-15837.55899	-785.847149	1.92	-1.92	0.08	-1.84	3.76

Table S10. Results of the energy-decomposition analysis at the sobEDAw level for TS1, TS2. These analyses were performed by Multiwfn 3.8 program and computed at the B3LYP-D3(BJ)/6-31G(d,p)-Lan2ldz(Rh,Br) level of theory in the gas phase. (All presented energies are in kcal/mol).

	**TS1**	**TS2**	**(TS2-TS1)**	**Note**
ΔE_{int} interaction	-68.59	-64.83	3.76	Favor TS1
ΔE_{els} electrostatic	-89.76	-86.74	3.02	Favor TS1

ΔE_{xrep} exchange-repulsion	256.11	249.17	-6.94	Favor TS2
ΔE_{orb} orbital	-175.62	-171.87	3.75	Favor TS1
ΔE_{disp} dispersion	-59.32	-55.39	3.93	Favor TS1

Table S11. Steric interaction energy. $\Delta E_{steric} = \Delta E_{dis} + \Delta E_{xrep}$ (All presented energies are in kcal/mol).

ΔE_{steric}	ΔE_{dis}	ΔE_{xrep}
-8.78	-1.84	-6.94

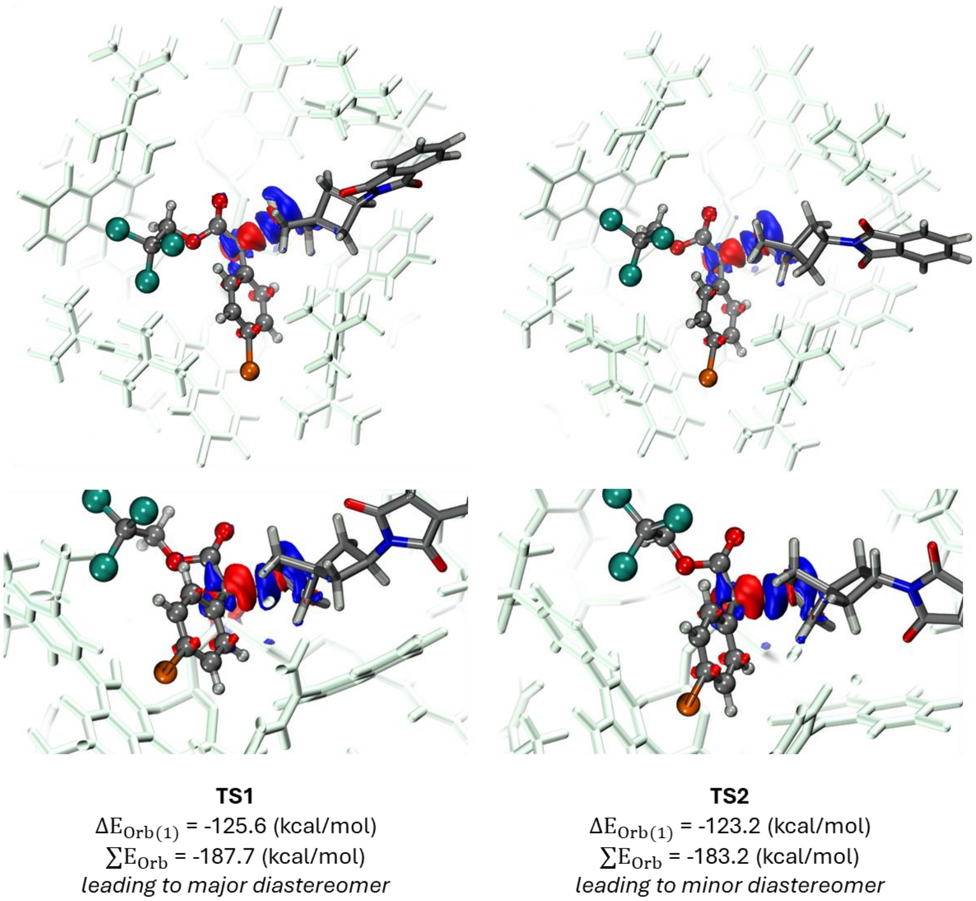

Figure S11. ETS-NOCV analysis. The sign of orbital is color coded of the charge flow is blue → red. $\Delta E_{Orb(1)}$ is the energy contributed by the orbital pair with largest eigenvalue. $\sum E_{Orb}$ is the total orbital-pair energy (isovalue = 0.005)

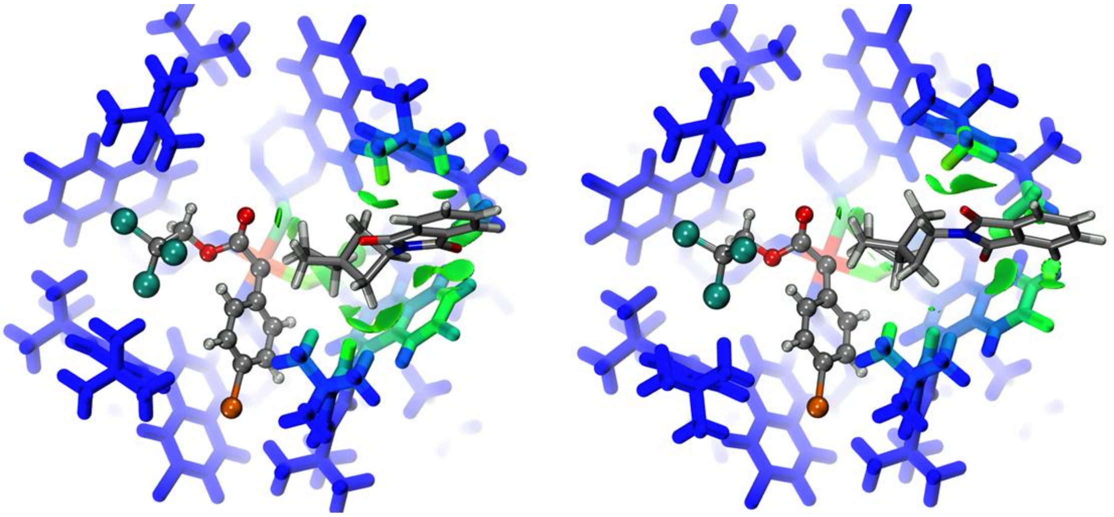

TS1 **TS2**

Figure S12. NCI-IGMH analysis. The green surface (isovalue = 0.005) represents noncovalent interactions between substrate and catalyst pocket. The brighter (greener) atoms the stronger their contribution to noncovalent interactions.

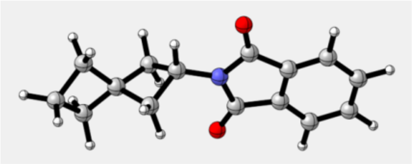

20a
(eclipse)
(0.0)

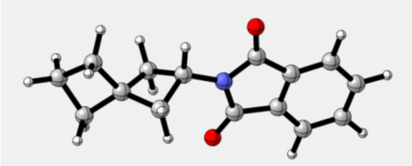

20b
(eclipse)
(0.0)

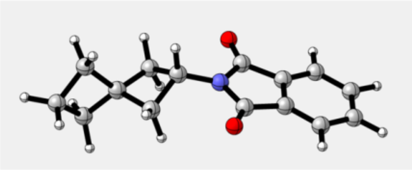

20d
(gauche)
(1.4)

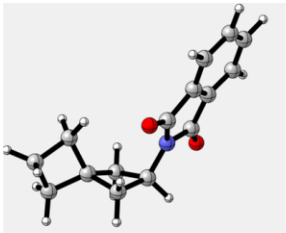

20c
(pseudo-axial)
(3.3)

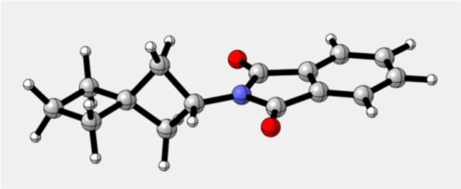

20TS

(1.5)

Figure S13. Substrate Conformations. Reported energies are free Gibbs energy in kcal/mol at room temperature.

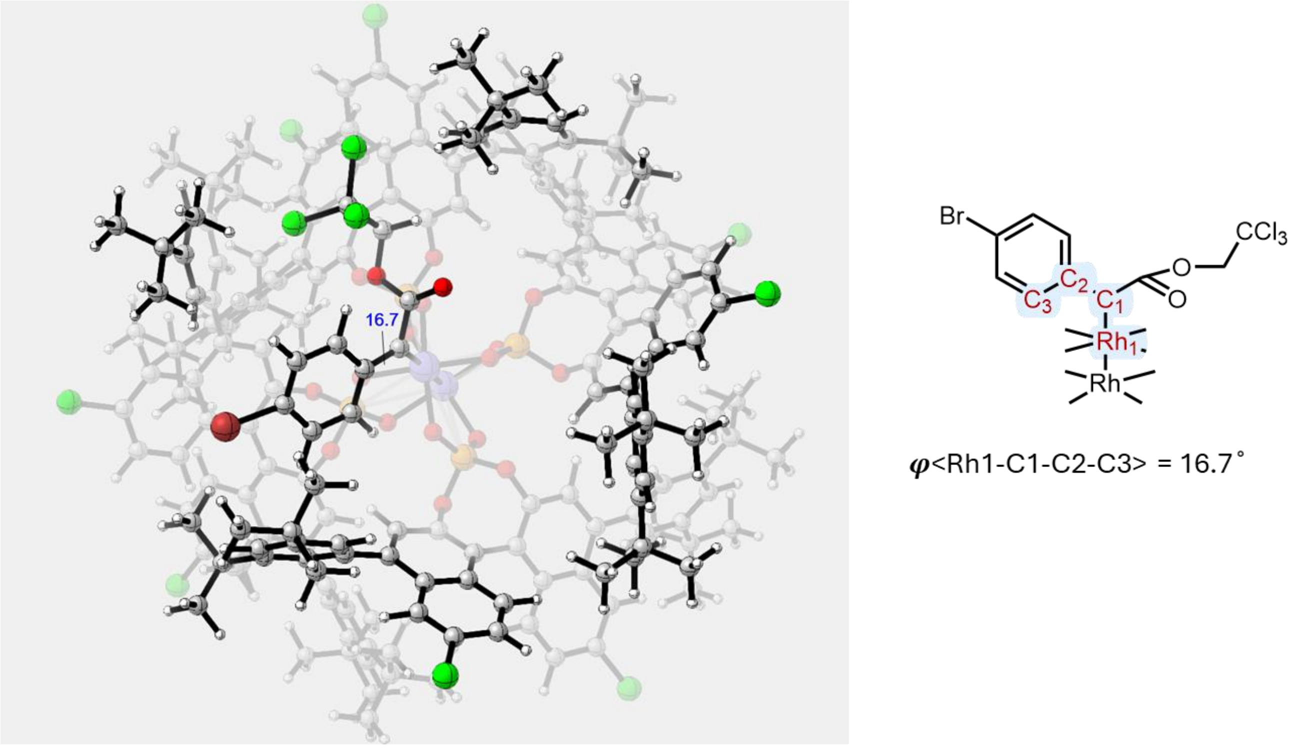

Intermediate **I**

Figure S14. Important dihedral angle in intermediate I

Table S12. Zero-point correction (ZPE), thermal correction to enthalpy (TCH), thermal correction to Gibbs free energy (TCG), electronic energies (E), enthalpies (H), and Gibbs free energies (G) (in Hartree) of the structures calculated at the ONIOM[M06:UFF] level of theory.

Structure	ZPE	TCH	TCG	E	H	G	Imaginary Frequency (in cm^{-1})
**Structure I**	3.694577	3.906510	3.435430	-13314.795111	-13310.888601	-13311.359681	-
**TS1**	3.964540	4.191931	3.693154	-14100.102623	-14095.910692	-14096.409469	-206.80
**TS2**	3.963864	4.191442	3.691111	-14100.097853	-14095.906411	-14096.406742	-227.44
**20a/20b**	0.269950	0.285546	0.227507	-785.303005	-785.017459	-785.075498	-
**20c**	0.269782	0.285340	0.227117	-785.297289	-785.011949	-785.070172	-
**20d**	0.269726	0.285396	0.227004	-785.300192	-785.014795	-785.073188	-
**20TS**	0.269538	0.284526	0.227667	-785.300759	-785.016233	-785.073092	-96.15

b. Coordination

Structure Intermediate I

O	-9.45750000	-1.83180000	-30.71530000
O	-8.71670000	-1.10790000	-33.13570000
Rh	-10.57220000	-0.09220000	-30.59240000
P	-8.53720000	-1.98760000	-31.92520000
C	-8.14610000	-4.44580000	-31.47060000
C	-6.78850000	-4.65080000	-31.33350000
C	-6.37040000	-5.58470000	-30.33210000
C	-7.34340000	-6.28420000	-29.54900000
C	-6.90340000	-7.27820000	-28.63950000
H	-7.63000000	-7.86730000	-28.08790000
C	-5.56620000	-7.50830000	-28.45850000
C	-4.59450000	-6.76380000	-29.15060000
H	-3.54050000	-6.94420000	-28.95930000
C	-4.99920000	-5.82800000	-30.06550000
H	-4.24980000	-5.25900000	-30.60720000
C	-8.73390000	-5.97880000	-29.70410000
C	-9.10630000	-5.07560000	-30.67100000
H	-10.14780000	-4.78310000	-30.80350000
Cl	-5.04030000	-8.74320000	-27.34080000
O	-8.61300000	-3.53060000	-32.39920000
C	-6.02620000	-2.45150000	-32.14010000
C	-5.86380000	-3.82410000	-32.14490000
C	-4.83740000	-4.35510000	-32.98580000
C	-3.96380000	-3.47520000	-33.70000000
C	-4.19060000	-2.06030000	-33.66010000
C	-5.22640000	-1.57990000	-32.88930000
H	-5.47660000	-0.51920000	-32.87480000
C	-2.87640000	-4.03660000	-34.41660000
H	-2.15180000	-3.38710000	-34.89790000
C	-2.72710000	-5.39470000	-34.50100000
C	-3.64120000	-6.27430000	-33.89290000
H	-3.52030000	-7.34730000	-34.01140000
C	-4.66470000	-5.75330000	-33.14690000
H	-5.36480000	-6.42570000	-32.65920000
Cl	-1.37850000	-6.06620000	-35.38610000
O	-7.02450000	-1.87920000	-31.36630000
C	-9.78310000	-6.56270000	-28.84330000
C	-10.93230000	-7.10000000	-29.42420000
H	-10.95930000	-7.07440000	-30.51250000
C	-11.94650000	-7.65560000	-28.63850000
C	-11.76300000	-7.64600000	-27.24490000
H	-12.53080000	-8.12000000	-26.64180000
C	-10.65010000	-7.06290000	-26.62120000
C	-9.66510000	-6.52610000	-27.45460000
H	-8.77200000	-6.05000000	-27.05380000
C	-13.21220000	-8.29180000	-29.24300000
C	-13.29220000	-8.13010000	-30.78070000
H	-13.27700000	-7.05590000	-31.05720000
H	-14.23420000	-8.56650000	-31.17850000
H	-12.44880000	-8.65030000	-31.28410000
C	-14.46980000	-7.63060000	-28.64100000
H	-14.55450000	-7.81020000	-27.55000000
H	-15.38750000	-8.04860000	-29.10560000
H	-14.44690000	-6.53330000	-28.81470000
C	-13.22620000	-9.80160000	-28.92550000
H	-12.31090000	-10.28890000	-29.32610000
H	-14.11230000	-10.29200000	-29.38390000
H	-13.26870000	-9.98740000	-27.83170000
C	-10.46320000	-7.00080000	-25.09380000
C	-9.18160000	-7.77080000	-24.70570000
H	-9.24220000	-8.82380000	-25.05680000
H	-9.04400000	-7.77750000	-23.60260000
H	-8.27590000	-7.30810000	-25.15110000
C	-11.64160000	-7.62580000	-24.30920000
H	-12.59320000	-7.09960000	-24.53600000
H	-11.47080000	-7.54780000	-23.21340000
H	-11.75170000	-8.70500000	-24.55110000
C	-10.32820000	-5.52850000	-24.64500000
H	-9.43190000	-5.04510000	-25.08640000
H	-10.23010000	-5.46070000	-23.53990000
H	-11.22190000	-4.94590000	-24.95010000
C	-3.36500000	-1.09830000	-34.42230000
C	-2.87230000	0.03920000	-33.78160000
H	-3.12720000	0.12620000	-32.72640000
C	-2.09600000	0.97760000	-34.46640000
C	-1.81440000	0.72000000	-35.81800000
H	-1.17990000	1.43330000	-36.33460000
C	-2.31540000	-0.39360000	-36.50670000
C	-3.10710000	-1.28650000	-35.77960000
H	-3.56340000	-2.15660000	-36.24780000
C	-1.54810000	2.25380000	-33.79870000
C	-2.02400000	2.41670000	-32.33590000
H	-1.64180000	3.36310000	-31.89480000
H	-3.13250000	2.45100000	-32.29560000
H	-1.65820000	1.58240000	-31.69900000
C	-2.02540000	3.49790000	-34.58170000
H	-1.59650000	3.53300000	-35.60430000
H	-1.71420000	4.43210000	-34.06620000
H	-3.13340000	3.49630000	-34.66920000
C	-0.00580000	2.21040000	-33.79090000
H	0.41000000	3.10510000	-33.27840000
H	0.35200000	1.30360000	-33.25680000
H	0.40730000	2.19300000	-34.82110000
C	-2.03700000	-0.66930000	-37.99630000
C	-1.33160000	-2.03600000	-38.14250000
H	-1.08030000	-2.23870000	-39.20630000
H	-0.38990000	-2.04970000	-37.55200000
H	-1.97510000	-2.86900000	-37.78950000
C	-1.13100000	0.39900000	-38.65490000
H	-0.95420000	0.15970000	-39.72620000
H	-1.60660000	1.40280000	-38.61290000
H	-0.13880000	0.43970000	-38.15530000
C	-3.37130000	-0.69520000	-38.77340000
H	-4.03100000	-1.51770000	-38.42640000
H	-3.19220000	-0.84960000	-39.85970000
H	-3.91350000	0.26620000	-38.64170000
O	-11.55940000	1.72500000	-30.68610000
O	-12.24820000	1.16530000	-33.15740000
P	-12.44520000	1.97570000	-31.90270000
C	-12.82130000	4.43890000	-31.34110000
C	-14.18310000	4.64830000	-31.22750000
C	-14.61290000	5.58720000	-30.23360000
C	-13.64910000	6.31740000	-29.46740000
C	-14.10200000	7.32840000	-28.58310000
H	-13.38160000	7.93810000	-28.04590000
C	-15.44230000	7.53740000	-28.39870000
C	-16.40430000	6.75540000	-29.06380000
H	-17.46030000	6.91570000	-28.86610000
C	-15.98740000	5.80800000	-29.96080000
H	-16.72890000	5.21030000	-30.48220000
C	-12.25550000	6.01380000	-29.59970000
C	-11.87150000	5.07630000	-30.53140000
H	-10.82990000	4.77010000	-30.62340000
Cl	-15.98530000	8.78590000	-27.30450000
O	-12.34010000	3.54200000	-32.27960000
C	-14.95220000	2.47810000	-32.11980000
C	-15.10520000	3.85100000	-32.07180000
C	-16.12780000	4.41690000	-32.89800000
C	-17.00820000	3.56760000	-33.64080000
C	-16.78190000	2.15250000	-33.66440000

C	-15.74790000	1.64150000	-32.91230000
H	-15.49410000	0.58270000	-32.95000000
C	-18.09350000	4.15830000	-34.33650000
H	-18.82220000	3.52860000	-34.83740000
C	-18.23270000	5.51940000	-34.37980000
C	-17.30790000	6.37210000	-33.75040000
H	-17.41690000	7.44920000	-33.83990000
C	-16.28810000	5.82060000	-33.02130000
H	-15.58010000	6.47420000	-32.52040000
Cl	-19.57710000	6.22800000	-35.24170000
O	-13.96760000	1.86740000	-31.35940000
C	-11.22110000	6.63350000	-28.74270000
C	-10.07930000	7.18590000	-29.32210000
H	-10.03960000	7.14170000	-30.40960000
C	-9.08100000	7.76700000	-28.53580000
C	-9.27900000	7.78260000	-27.14500000
H	-8.52050000	8.27260000	-26.54300000
C	-10.38940000	7.19640000	-26.52200000
C	-11.35080000	6.61840000	-27.35430000
H	-12.23130000	6.12200000	-26.94870000
C	-7.80280000	8.38540000	-29.13380000
C	-7.69160000	8.17430000	-30.66260000
H	-7.70070000	7.09110000	-30.90010000
H	-6.74140000	8.59700000	-31.05580000
H	-8.52450000	8.67670000	-31.20040000
C	-6.55700000	7.74020000	-28.48570000
H	-6.47140000	7.99060000	-27.40850000
H	-5.62700000	8.10250000	-28.97420000
H	-6.60310000	6.63420000	-28.58280000
C	-7.79370000	9.90400000	-28.86320000
H	-8.69660000	10.38000000	-29.30370000
H	-6.89390000	10.37800000	-29.31210000
H	-7.78160000	10.12320000	-27.77480000
C	-10.58900000	7.15180000	-24.99600000
C	-11.90590000	7.87110000	-24.62850000
H	-11.88920000	8.91950000	-24.99770000
H	-12.05040000	7.89050000	-23.52650000
H	-12.78820000	7.36130000	-25.06950000
C	-9.44370000	7.83660000	-24.21250000
H	-8.47150000	7.33680000	-24.41420000
H	-9.62650000	7.77960000	-23.11730000
H	-9.36790000	8.91250000	-24.48130000
C	-10.66210000	5.68050000	-24.53030000
H	-11.53600000	5.15530000	-24.96870000
H	-10.76050000	5.62080000	-23.42470000
H	-9.74150000	5.13410000	-24.82970000
C	-17.58810000	1.22430000	-34.48790000
C	-18.06340000	0.03850000	-33.92560000
H	-17.82550000	-0.10570000	-32.87300000
C	-18.80150000	-0.87720000	-34.68040000
C	-19.06640000	-0.54560000	-36.01930000
H	-19.66880000	-1.24440000	-36.59090000
C	-18.58690000	0.62190000	-36.62940000
C	-17.83040000	1.48810000	-35.83570000
H	-17.38800000	2.39490000	-36.24360000
C	-19.32250000	-2.20870000	-34.10430000
C	-18.88440000	-2.44120000	-32.63930000
H	-19.25410000	-3.41990000	-32.26270000
H	-17.77780000	-2.45290000	-32.56950000
H	-19.28820000	-1.65220000	-31.96850000
C	-18.77640000	-3.38760000	-34.94180000
H	-19.16270000	-3.36980000	-35.98170000
H	-19.07850000	-4.36100000	-34.49790000
H	-17.66610000	-3.35020000	-34.98200000
C	-20.86520000	-2.22030000	-34.14170000
H	-21.26250000	-3.15540000	-33.69040000
H	-21.27130000	-1.35690000	-33.57140000
H	-21.24970000	-2.16270000	-35.18130000
C	-18.85010000	0.98210000	-38.10350000
C	-19.58800000	2.33750000	-38.17730000
H	-19.82830000	2.59940000	-39.23060000
H	-20.53850000	2.29150000	-37.60270000
H	-18.97130000	3.16270000	-37.76320000
C	-19.71890000	-0.06530000	-38.84100000
H	-19.88760000	0.23620000	-39.89780000
H	-19.21760000	-1.05740000	-38.85500000
H	-20.71610000	-0.16270000	-38.35960000
C	-17.50560000	1.08880000	-38.85580000
H	-16.87240000	1.90450000	-38.44860000
H	-17.67260000	1.30500000	-39.93340000
H	-16.94080000	0.13480000	-38.77420000
O	-9.33300000	1.75410000	-32.98070000
O	-8.80540000	0.96470000	-30.52390000
Rh	-10.47480000	0.02330000	-33.19920000
P	-8.51130000	1.87010000	-31.71720000
C	-7.98360000	4.34300000	-32.00490000
C	-6.60990000	4.47670000	-32.05460000
C	-6.08970000	5.46120000	-32.95530000
C	-6.97660000	6.32970000	-33.66770000
C	-6.42230000	7.38380000	-34.43730000
H	-7.07620000	8.10520000	-34.91750000
C	-5.06590000	7.50220000	-34.57740000
C	-4.18360000	6.58550000	-33.97830000
H	-3.11400000	6.67730000	-34.14390000
C	-4.69620000	5.59670000	-33.18110000
H	-4.01840000	4.89630000	-32.70260000
C	-8.39240000	6.12380000	-33.58650000
C	-8.86180000	5.12070000	-32.76950000
H	-9.92210000	4.87590000	-32.73290000
Cl	-4.40170000	8.80970000	-35.52810000
O	-8.55400000	3.39070000	-31.17340000
C	-5.99760000	2.21570000	-31.26430000
C	-5.76990000	3.57850000	-31.22710000
C	-4.75910000	4.04280000	-30.32480000
C	-3.97620000	3.10830000	-29.57560000
C	-4.28320000	1.71000000	-29.63900000
C	-5.29220000	1.29490000	-30.47800000
H	-5.59470000	0.24880000	-30.52310000
C	-2.89230000	3.59450000	-28.80110000
H	-2.23730000	2.89670000	-28.28880000
C	-2.65790000	4.93890000	-28.70130000
C	-3.48380000	5.87650000	-29.34610000
H	-3.29850000	6.93900000	-29.21690000
C	-4.50450000	5.42570000	-30.14050000
H	-5.13590000	6.14750000	-30.64950000
Cl	-1.31140000	5.51840000	-27.74950000
O	-6.95550000	1.69590000	-32.11850000
C	-9.36620000	6.92350000	-34.36200000
C	-10.49450000	7.44090000	-33.72360000
H	-10.56290000	7.23100000	-32.65720000
C	-11.44780000	8.18400000	-34.42490000
C	-11.21470000	8.40710000	-35.79190000
H	-11.94130000	9.01280000	-36.32400000
C	-10.10970000	7.88300000	-36.47680000
C	-9.20170000	7.12560000	-35.73190000
H	-8.33830000	6.65220000	-36.19530000
C	-12.71590000	8.75620000	-33.76130000
C	-12.84500000	8.36150000	-32.27130000
H	-12.88220000	7.25800000	-32.17090000
H	-13.78050000	8.76910000	-31.83000000
H	-11.99490000	8.75830000	-31.67500000
C	-13.97060000	8.22390000	-34.49020000
H	-14.02200000	8.58030000	-35.53950000
H	-14.89740000	8.56920000	-33.98290000

H	-13.96830000	7.11230000	-34.49940000
C	-12.68950000	10.29730000	-33.83910000
H	-11.77360000	10.69260000	-33.34860000
H	-13.57550000	10.73230000	-33.32760000
H	-12.70320000	10.65420000	-34.89010000
C	-9.85910000	8.10000000	-37.98090000
C	-8.49750000	8.80290000	-38.17870000
H	-8.47790000	9.76850000	-37.62840000
H	-8.31290000	9.00990000	-39.25520000
H	-7.65660000	8.17720000	-37.81260000
C	-10.94120000	8.97460000	-38.65920000
H	-10.71910000	9.10850000	-39.74030000
H	-11.94220000	8.49740000	-38.58260000
H	-10.97860000	9.98600000	-38.19960000
C	-9.84070000	6.73450000	-38.70220000
H	-9.01010000	6.09240000	-38.34170000
H	-9.70500000	6.86870000	-39.79740000
H	-10.79770000	6.19520000	-38.53210000
C	-3.55670000	0.70530000	-28.83100000
C	-3.04730000	-0.43540000	-29.45150000
H	-3.22300000	-0.50140000	-30.52440000
C	-2.34670000	-1.40130000	-28.72370000
C	-2.16620000	-1.16140000	-27.35440000
H	-1.59500000	-1.88430000	-26.77710000
C	-2.68810000	-0.04410000	-26.68590000
C	-3.40220000	0.87810000	-27.45370000
H	-3.87950000	1.76180000	-27.03700000
C	-1.77270000	-2.67970000	-29.36100000
C	-2.15860000	-2.82970000	-30.85030000
H	-1.76610000	-3.78120000	-31.27090000
H	-3.26270000	-2.84330000	-30.95710000
H	-1.73970000	-2.00010000	-31.46000000
C	-2.31030000	-3.92100000	-28.61400000
H	-1.93080000	-3.97390000	-27.57270000
H	-1.99270000	-4.85670000	-29.12290000
H	-3.42050000	-3.89640000	-28.57750000
C	-0.23320000	-2.65170000	-29.26980000
H	0.20540000	-3.55300000	-29.75060000
H	0.16770000	-1.75090000	-29.78320000
H	0.11100000	-2.63190000	-28.21430000
C	-2.45880000	0.12360000	-25.17260000
C	-0.94310000	0.19490000	-24.88420000
H	-0.75550000	0.36760000	-23.80220000
H	-0.42730000	-0.74760000	-25.16250000
H	-0.48020000	1.02800000	-25.45640000
C	-3.07060000	-1.07830000	-24.42020000
H	-2.95940000	-0.95310000	-23.32120000
H	-4.15360000	-1.16930000	-24.65370000
H	-2.57350000	-2.03140000	-24.69700000
C	-3.10420000	1.41160000	-24.60570000
H	-2.67990000	2.31540000	-25.09470000
H	-2.91310000	1.50140000	-23.51410000
H	-4.20620000	1.39940000	-24.75080000
O	-11.62380000	-1.74690000	-33.29490000
O	-12.33580000	-1.14250000	-30.84080000
P	-12.50560000	-1.98870000	-32.09860000
C	-12.93090000	-4.50360000	-32.42530000
C	-14.30140000	-4.66770000	-32.50960000
C	-14.77650000	-5.65720000	-33.42590000
C	-13.85770000	-6.52830000	-34.09280000
C	-14.37990000	-7.58750000	-34.87810000
H	-13.70770000	-8.31450000	-35.32310000
C	-15.72910000	-7.70240000	-35.08010000
C	-16.63700000	-6.77980000	-34.52850000
H	-17.69780000	-6.86760000	-34.74580000
C	-16.15920000	-5.79010000	-33.71160000
H	-16.85340000	-5.08340000	-33.26540000
C	-12.44750000	-6.31770000	-33.94880000
C	-12.01770000	-5.28960000	-33.13880000
H	-10.95880000	-5.04360000	-33.06030000
Cl	-16.35160000	-9.01340000	-36.05270000
O	-12.39890000	-3.52930000	-31.59510000
C	-14.97160000	-2.44690000	-31.67280000
C	-15.17670000	-3.81260000	-31.67230000
C	-16.16320000	-4.32120000	-30.76520000
C	-16.89540000	-3.42280000	-29.92550000
C	-16.56920000	-2.02750000	-29.91580000
C	-15.62120000	-1.56500000	-30.79910000
H	-15.31190000	-0.51990000	-30.79910000
C	-17.95400000	-3.93460000	-29.13340000
H	-18.56350000	-3.25720000	-28.54320000
C	-18.22530000	-5.27580000	-29.11940000
C	-17.45570000	-6.18390000	-29.86630000
H	-17.67120000	-7.24720000	-29.81050000
C	-16.44750000	-5.70680000	-30.66220000
H	-15.85540000	-6.41230000	-31.23630000
Cl	-19.54520000	-5.88730000	-28.15220000
O	-14.04990000	-1.89860000	-32.55180000
C	-11.43240000	-7.13820000	-34.64570000
C	-10.33730000	-7.62170000	-33.92740000
H	-10.33260000	-7.37630000	-32.86670000
C	-9.33780000	-8.37550000	-34.54700000
C	-9.49450000	-8.65400000	-35.91480000
H	-8.73330000	-9.26990000	-36.38300000
C	-10.56590000	-8.16960000	-36.67800000
C	-11.51880000	-7.39170000	-36.01410000
H	-12.35560000	-6.94060000	-36.54380000
C	-8.09810000	-8.89770000	-33.79480000
C	-8.04500000	-8.42430000	-32.32230000
H	-8.03230000	-7.31590000	-32.27570000
H	-7.12450000	-8.79140000	-31.81810000
H	-8.91440000	-8.80710000	-31.74510000
C	-6.81450000	-8.38910000	-34.48960000
H	-6.70030000	-8.81150000	-35.50910000
H	-5.91050000	-8.68270000	-33.91330000
H	-6.83500000	-7.28100000	-34.57240000
C	-8.10700000	-10.44060000	-33.79490000
H	-9.03920000	-10.82100000	-33.32390000
H	-7.23950000	-10.83880000	-33.22490000
H	-8.04610000	-10.84900000	-34.82530000
C	-10.73280000	-8.44830000	-38.18360000
C	-12.07840000	-9.16770000	-38.42690000
H	-12.12390000	-10.11050000	-37.83980000
H	-12.20260000	-9.41920000	-39.50260000
H	-12.94100000	-8.53350000	-38.13330000
C	-9.61140000	-9.34230000	-38.76600000
H	-8.61840000	-8.85470000	-38.65680000
H	-9.77480000	-9.52300000	-39.85080000
H	-9.59320000	-10.33320000	-38.26260000
C	-10.71790000	-7.11300000	-38.95930000
H	-11.56990000	-6.46260000	-38.67080000
H	-10.79280000	-7.29190000	-40.05390000
H	-9.77400000	-6.56090000	-38.75920000
C	-17.19750000	-1.07970000	-28.97020000
C	-17.71660000	0.12740000	-29.43770000
H	-17.64240000	0.28260000	-30.51290000
C	-18.30240000	1.04740000	-28.56350000
C	-18.35750000	0.69270000	-27.20650000
H	-18.82980000	1.38410000	-26.51300000
C	-17.83110000	-0.50340000	-26.69300000
C	-17.23390000	-1.37490000	-27.60610000
H	-16.76210000	-2.31180000	-27.31960000
C	-18.88680000	2.39310000	-29.03180000
C	-18.69170000	2.63770000	-30.54770000

H	-19.10130000	3.62690000	-30.84730000
H	-17.61200000	2.63250000	-30.80370000
H	-19.21520000	1.86370000	-31.14960000
C	-18.19380000	3.55340000	-28.28360000
H	-18.36680000	3.50330000	-27.18900000
H	-18.58410000	4.53350000	-28.63290000
H	-17.09820000	3.52950000	-28.46530000
C	-20.40150000	2.42530000	-28.73910000
H	-20.85400000	3.37080000	-29.10940000
H	-20.90990000	1.57420000	-29.24190000
H	-20.60760000	2.36040000	-27.65020000
C	-17.94160000	-0.81570000	-25.18880000
C	-19.42980000	-0.83840000	-24.77590000
H	-19.53850000	-1.12230000	-23.70660000
H	-19.90580000	0.15600000	-24.90310000
H	-19.98980000	-1.57570000	-25.39120000
C	-17.19880000	0.26410000	-24.37270000
H	-17.27790000	0.05600000	-23.28370000
H	-16.12110000	0.27900000	-24.64180000
H	-17.61930000	1.27570000	-24.55050000
C	-17.33070000	-2.18850000	-24.81340000
H	-16.24570000	-2.21890000	-25.05290000
H	-17.84740000	-3.01260000	-25.35190000
H	-17.43580000	-2.38250000	-23.72380000
C	-11.00370000	-0.19280000	-28.64280000
C	-11.50030000	-1.32620000	-27.94810000
C	-11.19820000	1.15380000	-28.05470000
C	-11.41830000	-2.62610000	-28.51340000
C	-12.25270000	-1.16000000	-26.75330000
O	-10.32380000	1.85270000	-27.61700000
O	-12.51050000	1.47210000	-28.12930000
C	-12.12550000	-3.68510000	-27.97350000
H	-10.80490000	-2.77730000	-29.39690000
C	-12.96850000	-2.20740000	-26.21510000
H	-12.31420000	-0.18490000	-26.27520000
C	-12.88860000	2.80920000	-27.89290000
C	-12.92070000	-3.44660000	-26.85510000
H	-12.08300000	-4.67470000	-28.42740000
H	-13.57830000	-2.06780000	-25.32760000
H	-12.01130000	3.43440000	-27.68050000
H	-13.41540000	3.17660000	-28.78490000
C	-13.84700000	2.84590000	-26.71130000
Br	-14.03530000	-4.86780000	-26.15850000
Cl	-12.99410000	2.34760000	-25.21410000
Cl	-15.22480000	1.75840000	-26.99840000
Cl	-14.44100000	4.52030000	-26.52650000

Structure TS1

O	-9.42410000	-1.88820000	-30.76070000
O	-8.70430000	-1.33140000	-33.22730000
Rh	-10.29150000	-0.00630000	-30.70340000
P	-8.52450000	-2.13780000	-31.96340000
C	-8.16720000	-4.61350000	-31.44310000
C	-6.80810000	-4.82830000	-31.30820000
C	-6.40080000	-5.79290000	-30.33060000
C	-7.37990000	-6.54520000	-29.60730000
C	-6.94540000	-7.58330000	-28.74510000
H	-7.67570000	-8.21300000	-28.24600000
C	-5.60940000	-7.79760000	-28.53820000
C	-4.63420000	-6.99710000	-29.16000000
H	-3.58260000	-7.16650000	-28.94730000
C	-5.03270000	-6.02520000	-30.03910000
H	-4.28120000	-5.41610000	-30.53170000
C	-8.77000000	-6.23990000	-29.76250000
C	-9.13400000	-5.27230000	-30.67100000
H	-10.17390000	-4.96520000	-30.78070000
Cl	-5.08950000	-9.08300000	-27.47540000
O	-8.63080000	-3.69840000	-32.37080000
C	-6.01300000	-2.64700000	-32.15090000
C	-5.86720000	-4.02130000	-32.12210000
C	-4.83280000	-4.57870000	-32.93920000
C	-3.93540000	-3.72150000	-33.65200000
C	-4.14970000	-2.30450000	-33.65010000
C	-5.19400000	-1.79990000	-32.90860000
H	-5.43740000	-0.73760000	-32.92710000
C	-2.84340000	-4.30540000	-34.34250000
H	-2.10480000	-3.67020000	-34.82190000
C	-2.71030000	-5.66610000	-34.40760000
C	-3.64830000	-6.52470000	-33.80690000
H	-3.54330000	-7.60070000	-33.91330000
C	-4.67700000	-5.98080000	-33.08440000
H	-5.39670000	-6.63980000	-32.60750000
Cl	-1.35670000	-6.36670000	-35.26280000
O	-7.00870000	-2.04450000	-31.40370000
C	-9.82180000	-6.89840000	-28.95980000
C	-10.95170000	-7.41720000	-29.58840000
H	-10.96490000	-7.31690000	-30.67220000
C	-11.96850000	-8.03750000	-28.85380000
C	-11.79420000	-8.12360000	-27.45930000
H	-12.56280000	-8.64430000	-26.89820000
C	-10.69390000	-7.57020000	-26.78400000
C	-9.71760000	-6.95490000	-27.57260000
H	-8.84060000	-6.48330000	-27.13430000
C	-13.23760000	-8.62270000	-29.50920000
C	-13.32090000	-8.31840000	-31.02490000
H	-13.30360000	-7.22210000	-31.19980000
H	-14.26460000	-8.71400000	-31.45960000
H	-12.47970000	-8.79120000	-31.57660000
C	-14.49490000	-8.01780000	-28.84530000
H	-14.60210000	-8.33880000	-27.78890000
H	-15.41570000	-8.34290000	-29.37570000
H	-14.44420000	-6.90810000	-28.86750000
C	-13.24720000	-10.15400000	-29.32830000
H	-12.33750000	-10.60210000	-29.78350000
H	-14.14010000	-10.60160000	-29.81650000
H	-13.27290000	-10.43530000	-28.25450000
C	-10.52010000	-7.59580000	-25.25150000
C	-9.21190000	-8.33520000	-24.89450000
H	-9.22440000	-9.36550000	-25.31160000
H	-9.08690000	-8.40530000	-23.79210000
H	-8.32130000	-7.80810000	-25.29680000
C	-11.68210000	-8.31280000	-24.52420000
H	-12.64900000	-7.80170000	-24.72320000
H	-11.52170000	-8.30680000	-23.42410000
H	-11.75460000	-9.37470000	-24.84460000
C	-10.45240000	-6.14740000	-24.71780000
H	-9.57050000	-5.60400000	-25.11740000
H	-10.37260000	-6.13910000	-23.60910000
H	-11.36710000	-5.58600000	-25.00690000
C	-3.31840000	-1.36850000	-34.43890000
C	-2.82400000	-0.21030000	-33.83910000
H	-3.07280000	-0.09260000	-32.78570000
C	-2.05820000	0.71240000	-34.56150000
C	-1.79660000	0.41200000	-35.91130000
H	-1.18230000	1.11740000	-36.46050000
C	-2.29360000	-0.73060000	-36.55950000
C	-3.07120000	-1.60240000	-35.79000000
H	-3.52780000	-2.49160000	-36.22010000
C	-1.50300000	2.01450000	-33.94330000
C	-1.92450000	2.20140000	-32.46750000
H	-1.52580000	3.15420000	-32.05590000
H	-3.02950000	2.24080000	-32.38920000
H	-1.53950000	1.37480000	-31.83190000
C	-2.03170000	3.23210000	-34.73450000

H	-1.66080000	3.23660000	-35.78020000
H	-1.69940000	4.18300000	-34.26420000
H	-3.14300000	3.22300000	-34.75890000
C	0.03940000	1.99580000	-33.99330000
H	0.45970000	2.90450000	-33.51020000
H	0.43170000	1.10290000	-33.45990000
H	0.41470000	1.97050000	-35.03750000
C	-2.02850000	-1.05950000	-38.04370000
C	-1.31490000	-2.42560000	-38.14740000
H	-1.07400000	-2.66550000	-39.20580000
H	-0.36680000	-2.41100000	-37.56730000
H	-1.94880000	-3.24900000	-37.75640000
C	-1.13690000	-0.00820000	-38.74660000
H	-0.96600000	-0.28420000	-39.81000000
H	-1.62090000	0.99240000	-38.73640000
H	-0.14200000	0.05900000	-38.25550000
C	-3.37040000	-1.12250000	-38.80500000
H	-4.02040000	-1.93620000	-38.42080000
H	-3.20140000	-1.31510000	-39.88670000
H	-3.91810000	-0.16070000	-38.70240000
O	-11.27380000	1.81860000	-30.78140000
O	-12.00910000	1.26780000	-33.24750000
P	-12.20670000	2.03610000	-31.96910000
C	-12.58260000	4.39350000	-31.21470000
C	-13.92320000	4.53600000	-30.92130000
C	-14.25750000	5.21640000	-29.70750000
C	-13.22340000	5.76420000	-28.88320000
C	-13.58870000	6.44950000	-27.69800000
H	-12.82480000	6.91810000	-27.08480000
C	-14.89870000	6.49700000	-27.30140000
C	-15.92390000	5.91800000	-28.07040000
H	-16.94590000	5.95090000	-27.70620000
C	-15.59810000	5.30470000	-29.25330000
H	-16.38380000	4.85960000	-29.85730000
C	-11.84990000	5.58080000	-29.24680000
C	-11.55750000	4.90160000	-30.40610000
H	-10.52600000	4.68230000	-30.68270000
Cl	-15.31140000	7.25410000	-25.78280000
O	-12.20090000	3.62010000	-32.30180000
C	-14.74990000	2.48730000	-31.99590000
C	-14.91200000	3.84080000	-31.78010000
C	-15.99560000	4.48350000	-32.45020000
C	-16.90660000	3.71660000	-33.24550000
C	-16.68630000	2.31130000	-33.42510000
C	-15.60450000	1.72780000	-32.80250000
H	-15.36490000	0.67470000	-32.94920000
C	-18.03140000	4.37320000	-33.80500000
H	-18.77700000	3.80150000	-34.34900000
C	-18.19480000	5.72390000	-33.65070000
C	-17.26130000	6.50370000	-32.94350000
H	-17.40290000	7.57800000	-32.86590000
C	-16.19230000	5.88380000	-32.35310000
H	-15.47260000	6.47560000	-31.79290000
Cl	-19.59190000	6.51230000	-34.34270000
O	-13.70450000	1.82300000	-31.38040000
C	-10.73450000	6.06180000	-28.40600000
C	-9.72480000	6.82780000	-28.98320000
H	-9.84630000	7.02310000	-30.04680000
C	-8.66610000	7.32730000	-28.21620000
C	-8.67730000	7.03760000	-26.83750000
H	-7.88510000	7.47020000	-26.23670000
C	-9.65130000	6.22900000	-26.22670000
C	-10.66460000	5.73620000	-27.05440000
H	-11.44360000	5.07840000	-26.67290000
C	-7.54480000	8.20070000	-28.81630000
C	-7.60080000	8.26340000	-30.36350000
H	-7.54610000	7.24140000	-30.79660000

H	-6.74700000	8.84850000	-30.76930000
H	-8.53140000	8.76040000	-30.71260000
C	-6.16760000	7.62380000	-28.42730000
H	-5.96050000	7.73120000	-27.34280000
H	-5.34920000	8.14910000	-28.96490000
H	-6.12990000	6.54590000	-28.68340000
C	-7.67140000	9.63660000	-28.27090000
H	-8.66360000	10.06330000	-28.53350000
H	-6.88290000	10.29120000	-28.70180000
H	-7.56570000	9.65880000	-27.16550000
C	-9.68330000	5.90510000	-24.71800000
C	-10.99130000	6.45420000	-24.10690000
H	-11.07330000	7.54780000	-24.28830000
H	-11.01850000	6.27860000	-23.00960000
H	-11.88460000	5.96100000	-24.54510000
C	-8.49890000	6.52880000	-23.94290000
H	-7.52870000	6.15290000	-24.33430000
H	-8.55020000	6.26310000	-22.86460000
H	-8.51870000	7.63780000	-24.01350000
C	-9.62820000	4.37640000	-24.50770000
H	-10.49020000	3.86390000	-24.98120000
H	-9.64840000	4.12740000	-23.42460000
H	-8.69420000	3.96220000	-24.93760000
C	-17.57290000	1.45240000	-34.24090000
C	-18.01650000	0.24150000	-33.70890000
H	-17.68320000	0.03440000	-32.69330000
C	-18.83310000	-0.62320000	-34.44490000
C	-19.19630000	-0.21210000	-35.74080000
H	-19.84400000	-0.87690000	-36.30220000
C	-18.75760000	0.98940000	-36.32210000
C	-17.92780000	1.80250000	-35.54130000
H	-17.51080000	2.73120000	-35.92620000
C	-19.33630000	-1.97620000	-33.89530000
C	-18.81850000	-2.27070000	-32.46800000
H	-19.18320000	-3.25710000	-32.10700000
H	-17.70940000	-2.30160000	-32.45890000
H	-19.17090000	-1.50050000	-31.74830000
C	-18.85120000	-3.12150000	-34.81130000
H	-19.27990000	-3.04180000	-35.83170000
H	-19.15570000	-4.10900000	-34.40170000
H	-17.74280000	-3.10500000	-34.89600000
C	-20.87890000	-1.97350000	-33.84310000
H	-21.25920000	-2.92050000	-33.40210000
H	-21.24210000	-1.12740000	-33.22030000
H	-21.32300000	-1.87710000	-34.85570000
C	-19.13840000	1.44150000	-37.74770000
C	-19.86670000	2.80200000	-37.67750000
H	-20.19010000	3.12920000	-38.68950000
H	-20.76710000	2.72500000	-37.03030000
H	-19.20990000	3.59680000	-37.26580000
C	-20.07490000	0.44340000	-38.46950000
H	-20.32970000	0.80830000	-39.48840000
H	-19.58510000	-0.54780000	-38.58400000
H	-21.02830000	0.32060000	-37.91120000
C	-17.85960000	1.58740000	-38.60070000
H	-17.18720000	2.37330000	-38.19790000
H	-18.11280000	1.87010000	-39.64560000
H	-17.29960000	0.62740000	-38.62400000
O	-9.05230000	1.59760000	-33.31830000
O	-8.43810000	0.87750000	-30.86990000
Rh	-10.36300000	-0.05010000	-33.28310000
P	-8.22610000	1.78890000	-32.06960000
C	-7.80710000	4.33780000	-32.23400000
C	-6.43410000	4.50910000	-32.26890000
C	-5.94300000	5.56180000	-33.10490000
C	-6.84650000	6.49120000	-33.71250000
C	-6.30930000	7.59650000	-34.41940000

H	-6.97240000	8.35860000	-34.81730000
C	-4.95780000	7.70880000	-34.60660000
C	-4.06370000	6.74000000	-34.11460000
H	-3.00070000	6.83030000	-34.31950000
C	-4.55730000	5.70070000	-33.37150000
H	-3.87350000	4.95710000	-32.97160000
C	-8.25870000	6.28880000	-33.59170000
C	-8.70250000	5.19290000	-32.88750000
H	-9.76310000	4.94850000	-32.84490000
Cl	-4.31450000	9.07530000	-35.48610000
O	-8.37060000	3.30760000	-31.50550000
C	-5.78850000	2.24660000	-31.52130000
C	-5.57850000	3.60990000	-31.45690000
C	-4.60490000	4.06950000	-30.50920000
C	-3.89680000	3.12890000	-29.69570000
C	-4.24570000	1.74060000	-29.73880000
C	-5.17680000	1.32390000	-30.66260000
H	-5.49570000	0.28260000	-30.71380000
C	-2.84180000	3.59270000	-28.87110000
H	-2.25000000	2.88330000	-28.30090000
C	-2.55430000	4.92800000	-28.79630000
C	-3.30370000	5.87610000	-29.51260000
H	-3.07970000	6.93380000	-29.40670000
C	-4.30240000	5.44460000	-30.34550000
H	-4.87630000	6.18150000	-30.89830000
Cl	-1.24220000	5.48170000	-27.78310000
O	-6.66710000	1.74040000	-32.46790000
C	-9.27150000	7.18190000	-34.19640000
C	-10.34300000	7.61040000	-33.41180000
H	-10.31690000	7.27510000	-32.37660000
C	-11.36010000	8.41210000	-33.93960000
C	-11.24500000	8.78220000	-35.29280000
H	-12.02700000	9.41700000	-35.69540000
C	-10.19050000	8.36440000	-36.12150000
C	-9.21610000	7.54490000	-35.53960000
H	-8.38950000	7.13910000	-36.11970000
C	-12.57380000	8.88690000	-33.11020000
C	-12.54290000	8.37000000	-31.65200000
H	-12.55860000	7.26160000	-31.63400000
H	-13.43210000	8.72320000	-31.08570000
H	-11.63880000	8.73420000	-31.11780000
C	-13.87750000	8.37130000	-33.75870000
H	-14.02690000	8.79350000	-34.77400000
H	-14.76120000	8.65880000	-33.14870000
H	-13.85480000	7.26290000	-33.84170000
C	-12.59630000	10.42960000	-33.06010000
H	-11.64430000	10.81550000	-32.63540000
H	-13.43410000	10.79060000	-32.42480000
H	-12.73220000	10.87090000	-34.06930000
C	-10.06380000	8.75340000	-37.60970000
C	-8.72930000	9.49840000	-37.83400000
H	-8.67440000	10.39700000	-37.18190000
H	-8.63380000	9.82720000	-38.89160000
H	-7.85630000	8.85090000	-37.60760000
C	-11.20590000	9.67870000	-38.09310000
H	-11.07300000	9.94170000	-39.16520000
H	-12.19230000	9.17580000	-37.99430000
H	-11.21730000	10.62840000	-37.51560000
C	-10.09180000	7.47800000	-38.47960000
H	-9.22910000	6.81480000	-38.26050000
H	-10.04640000	7.73660000	-39.55990000
H	-11.02710000	6.90600000	-38.29600000
C	-3.64430000	0.74800000	-28.82340000
C	-3.15380000	-0.45050000	-29.34030000
H	-3.22920000	-0.55410000	-30.42100000
C	-2.59600000	-1.42590000	-28.50590000
C	-2.53670000	-1.12510000	-27.13490000

H	-2.09120000	-1.85980000	-26.46990000
C	-3.03190000	0.06330000	-26.57050000
C	-3.60460000	0.98920000	-27.44850000
H	-4.06010000	1.92420000	-27.12580000
C	-2.04200000	-2.76740000	-29.02820000
C	-2.26970000	-2.95460000	-30.54720000
H	-1.89240000	-3.94340000	-30.88770000
H	-3.35330000	-2.90910000	-30.78300000
H	-1.73520000	-2.17520000	-31.13230000
C	-2.74010000	-3.94070000	-28.30550000
H	-2.52860000	-3.94310000	-27.21670000
H	-2.38550000	-4.91370000	-28.70780000
H	-3.84000000	-3.88170000	-28.44670000
C	-0.52410000	-2.83190000	-28.76250000
H	-0.09430000	-3.77550000	-29.16350000
H	-0.00970000	-1.97720000	-29.25280000
H	-0.30060000	-2.79560000	-27.67550000
C	-2.94670000	0.28780000	-25.04680000
C	-1.47650000	0.18130000	-24.58280000
H	-1.38740000	0.40990000	-23.49860000
H	-1.06760000	-0.83870000	-24.73480000
H	-0.84180000	0.90060000	-25.14450000
C	-3.79560000	-0.78110000	-24.32690000
H	-3.77680000	-0.62330000	-23.22680000
H	-4.85210000	-0.73030000	-24.66980000
H	-3.41490000	-1.80480000	-24.52710000
C	-3.46760000	1.68050000	-24.61710000
H	-2.89250000	2.48920000	-25.11860000
H	-3.36170000	1.82060000	-23.51930000
H	-4.54380000	1.79510000	-24.85980000
O	-11.68910000	-1.66310000	-33.11880000
O	-12.15680000	-0.93390000	-30.63500000
P	-12.46990000	-1.81970000	-31.83510000
C	-12.97650000	-4.28960000	-32.18530000
C	-14.35030000	-4.42760000	-32.23670000
C	-14.87350000	-5.36690000	-33.18100000
C	-13.98890000	-6.20760000	-33.92750000
C	-14.54670000	-7.21990000	-34.74880000
H	-13.89540000	-7.92280000	-35.25900000
C	-15.90310000	-7.31880000	-34.90380000
C	-16.78200000	-6.42250000	-34.26920000
H	-17.85090000	-6.49450000	-34.44870000
C	-16.26680000	-5.47760000	-33.42200000
H	-16.94160000	-4.79130000	-32.91900000
C	-12.57250000	-6.01470000	-33.82900000
C	-12.09960000	-5.04450000	-32.97450000
H	-11.03810000	-4.80670000	-32.92810000
Cl	-16.57190000	-8.57240000	-35.92140000
O	-12.40850000	-3.35670000	-31.33120000
C	-14.96970000	-2.21420000	-31.33440000
C	-15.18920000	-3.57820000	-31.35990000
C	-16.18740000	-4.09560000	-30.47410000
C	-16.96570000	-3.20710000	-29.66740000
C	-16.66200000	-1.80680000	-29.65510000
C	-15.66640000	-1.34080000	-30.48560000
H	-15.37870000	-0.28910000	-30.49300000
C	-18.04600000	-3.73910000	-28.91800000
H	-18.70240000	-3.07450000	-28.36550000
C	-18.27960000	-5.08690000	-28.89650000
C	-17.45700000	-5.98450000	-29.59930000
H	-17.64300000	-7.05270000	-29.53330000
C	-16.44020000	-5.48710000	-30.37020000
H	-15.81140000	-6.17670000	-30.92460000
Cl	-19.62210000	-5.72050000	-27.97430000
O	-14.04090000	-1.65360000	-32.19150000
C	-11.60310000	-6.79170000	-34.63230000
C	-10.49270000	-7.36190000	-34.01000000

H	-10.43240000	-7.20170000	-32.93460000
C	-9.54600000	-8.09160000	-34.73860000
C	-9.77040000	-8.23300000	-36.12080000
H	-9.04830000	-8.82080000	-36.67720000
C	-10.85950000	-7.65210000	-36.79090000
C	-11.76010000	-6.91920000	-36.01080000
H	-12.61270000	-6.40640000	-36.45170000
C	-8.29890000	-8.73520000	-34.09360000
C	-8.18940000	-8.43930000	-32.57970000
H	-8.12360000	-7.34640000	-32.40780000
H	-7.27460000	-8.90070000	-32.14810000
H	-9.06280000	-8.84770000	-32.02660000
C	-7.02230000	-8.18460000	-34.76760000
H	-6.96560000	-8.46870000	-35.83860000
H	-6.11150000	-8.58670000	-34.27350000
H	-6.99840000	-7.07530000	-34.69960000
C	-8.35680000	-10.26720000	-34.27060000
H	-9.29010000	-10.67210000	-33.82260000
H	-7.49010000	-10.75370000	-33.77260000
H	-8.33050000	-10.55670000	-35.34180000
C	-11.10200000	-7.78240000	-38.30930000
C	-12.47340000	-8.44940000	-38.55500000
H	-12.51400000	-9.44310000	-38.05840000
H	-12.65270000	-8.59410000	-39.64240000
H	-13.30610000	-7.82990000	-38.16070000
C	-10.02790000	-8.63810000	-39.02250000
H	-9.01990000	-8.18320000	-38.90990000
H	-10.24080000	-8.71040000	-40.11140000
H	-10.01240000	-9.67280000	-38.61650000
C	-11.09050000	-6.37970000	-38.95490000
H	-11.91260000	-5.74380000	-38.56510000
H	-11.21980000	-6.45170000	-40.05670000
H	-10.12560000	-5.86770000	-38.74860000
C	-17.41360000	-0.85780000	-28.80340000
C	-17.95720000	0.28940000	-29.37930000
H	-17.75630000	0.40930000	-30.44230000
C	-18.73730000	1.18000000	-28.63310000
C	-18.95280000	0.86620000	-27.27850000
H	-19.58920000	1.54090000	-26.71580000
C	-18.39820000	-0.26140000	-26.64940000
C	-17.61020000	-1.10300000	-27.44460000
H	-17.13100000	-1.99150000	-27.03750000
C	-19.39070000	2.43680000	-29.24580000
C	-18.94560000	2.68500000	-30.70510000
H	-19.38850000	3.62370000	-31.10330000
H	-17.84060000	2.78210000	-30.75420000
H	-19.27400000	1.85860000	-31.37140000
C	-18.99720000	3.68220000	-28.42360000
H	-19.47340000	3.67960000	-27.42170000
H	-19.31660000	4.61770000	-28.93180000
H	-17.89630000	3.71110000	-28.28910000
C	-20.92440000	2.27550000	-29.23550000
H	-21.41510000	3.16090000	-29.69500000
H	-21.22120000	1.37190000	-29.81090000
H	-21.31420000	2.17150000	-28.20100000
C	-18.65330000	-0.62890000	-25.17160000
C	-19.35400000	-2.00300000	-25.09980000
H	-19.59050000	-2.27050000	-24.04700000
H	-20.30340000	-1.98300000	-25.67760000
H	-18.71260000	-2.80980000	-25.51290000
C	-19.54890000	0.39820000	-24.43830000
H	-19.69230000	0.10910000	-23.37440000
H	-19.08330000	1.40650000	-24.44930000
H	-20.55500000	0.45390000	-24.90720000
C	-17.31090000	-0.70020000	-24.41560000
H	-16.64300000	-1.48150000	-24.83380000
H	-17.47330000	-0.93760000	-23.34180000
H	-16.79080000	0.27550000	-24.47900000
C	-10.18810000	0.23410000	-28.50020000
C	-9.26830000	1.26220000	-27.98860000
C	-10.11130000	-1.16410000	-28.01040000
H	-11.27490000	0.60150000	-28.73710000
C	-9.17000000	2.52730000	-28.59280000
C	-8.41050000	0.98140000	-26.91300000
O	-11.02140000	-1.88100000	-27.66710000
O	-8.81750000	-1.55270000	-28.08010000
C	-12.18860000	0.74350000	-27.73880000
C	-8.19410000	3.43920000	-28.20830000
H	-9.83470000	2.78380000	-29.41320000
C	-7.42080000	1.87150000	-26.52810000
H	-8.48300000	0.03580000	-26.38510000
C	-8.49280000	-2.90430000	-27.87970000
C	-12.84660000	2.07170000	-28.04790000
C	-12.03190000	1.10380000	-26.29260000
H	-12.71120000	-0.18500000	-27.98410000
C	-7.31150000	3.08180000	-27.19780000
H	-8.11340000	4.40310000	-28.71200000
H	-6.75070000	1.61850000	-25.71230000
H	-9.33930000	-3.46670000	-27.46470000
H	-8.18580000	-3.34050000	-28.84060000
C	-7.31080000	-2.96280000	-26.92290000
H	-13.61720000	2.09300000	-28.82710000
H	-12.10530000	2.86410000	-28.23540000
C	-13.24270000	2.03410000	-26.55150000
H	-12.11180000	0.30260000	-25.55200000
H	-11.12740000	1.70470000	-26.11730000
Br	-5.88960000	4.30900000	-26.66720000
Cl	-7.80760000	-2.36090000	-25.30410000
Cl	-5.96000000	-1.98720000	-27.53920000
Cl	-6.79320000	-4.66670000	-26.77250000
C	-13.55970000	3.31220000	-25.75760000
C	-14.66120000	1.51040000	-26.25590000
C	-15.05250000	2.94750000	-25.86170000
H	-13.26620000	4.27240000	-26.19700000
H	-13.19320000	3.23870000	-24.72860000
H	-15.22180000	1.04420000	-27.07640000
H	-14.65350000	0.84410000	-25.38780000
H	-15.54110000	3.48320000	-26.68550000
N	-15.89350000	3.13390000	-24.70140000
C	-17.10030000	3.85130000	-24.72270000
C	-15.63880000	2.58190000	-23.43820000
C	-17.67530000	3.71300000	-23.36150000
O	-17.55150000	4.44320000	-25.67540000
O	-14.66360000	1.93020000	-23.13440000
C	-16.80320000	2.95050000	-22.59420000
C	-18.86810000	4.18700000	-22.84930000
C	-17.08420000	2.62770000	-21.28040000
H	-19.54130000	4.77980000	-23.46360000
C	-19.16410000	3.86570000	-21.52290000
H	-16.39250000	2.02750000	-20.69450000
C	-18.28780000	3.09920000	-20.75210000
H	-20.09350000	4.21540000	-21.08010000
H	-18.55110000	2.86600000	-19.72340000

Structure II

O	-9.56150000	-1.87210000	-30.73490000
O	-8.76010000	-1.18670000	-33.14590000
Rh	-10.62400000	-0.09140000	-30.66370000
P	-8.60490000	-2.03670000	-31.91290000
C	-8.25850000	-4.45820000	-31.34190000
C	-6.91810000	-4.66680000	-31.09040000
C	-6.58590000	-5.53390000	-29.99940000
C	-7.62610000	-6.14180000	-29.22700000
C	-7.27870000	-7.05440000	-28.20090000

H	-8.05930000	-7.55290000	-27.63370000
C	-5.96450000	-7.31860000	-27.93150000
C	-4.92870000	-6.69010000	-28.64180000
H	-3.89280000	-6.90980000	-28.39640000
C	-5.24250000	-5.81090000	-29.64370000
H	-4.44060000	-5.32280000	-30.18820000
C	-8.99530000	-5.84350000	-29.51640000
C	-9.28750000	-5.02180000	-30.57750000
H	-10.31620000	-4.74730000	-30.81320000
Cl	-5.54940000	-8.44010000	-26.65500000
O	-8.63400000	-3.58980000	-32.35520000
C	-6.07140000	-2.51910000	-31.94940000
C	-5.92840000	-3.89250000	-31.87740000
C	-4.85120000	-4.47300000	-32.61430000
C	-3.91110000	-3.63790000	-33.29720000
C	-4.11530000	-2.22010000	-33.33570000
C	-5.20000000	-1.69270000	-32.66940000
H	-5.43200000	-0.62900000	-32.71880000
C	-2.78270000	-4.24560000	-33.90390000
H	-2.01300000	-3.62810000	-34.35640000
C	-2.65110000	-5.60810000	-33.91730000
C	-3.62430000	-6.44710000	-33.34430000
H	-3.51460000	-7.52600000	-33.40900000
C	-4.69210000	-5.87920000	-32.70180000
H	-5.44110000	-6.51840000	-32.24260000
Cl	-1.25050000	-6.33690000	-34.66620000
O	-7.11520000	-1.89700000	-31.28330000
C	-10.10840000	-6.38780000	-28.71310000
C	-11.12410000	-7.09870000	-29.34670000
H	-11.01070000	-7.20220000	-30.42430000
C	-12.17970000	-7.65670000	-28.61660000
C	-12.17040000	-7.46040000	-27.22190000
H	-12.96770000	-7.92720000	-26.65440000
C	-11.19540000	-6.69940000	-26.55450000
C	-10.16710000	-6.16680000	-27.34050000
H	-9.36750000	-5.55770000	-26.91780000
C	-13.29910000	-8.48990000	-29.27500000
C	-13.23240000	-8.46110000	-30.82240000
H	-13.29130000	-7.41570000	-31.19410000
H	-14.07990000	-9.02600000	-31.26780000
H	-12.29560000	-8.93020000	-31.19320000
C	-14.67740000	-7.93330000	-28.86120000
H	-14.88970000	-8.10050000	-27.78530000
H	-15.49360000	-8.42690000	-29.43170000
H	-14.71310000	-6.84270000	-29.05730000
C	-13.17930000	-9.95640000	-28.81570000
H	-12.18570000	-10.36870000	-29.09550000
H	-13.96540000	-10.58230000	-29.29130000
H	-13.29420000	-10.04520000	-27.71460000
C	-11.20660000	-6.42850000	-25.03570000
C	-9.87440000	-6.90630000	-24.41420000
H	-9.70300000	-7.97970000	-24.64660000
H	-9.88790000	-6.78610000	-23.30920000
H	-9.01030000	-6.32400000	-24.79570000
C	-12.35550000	-7.15710000	-24.29910000
H	-13.34620000	-6.81720000	-24.67130000
H	-12.32120000	-6.94310000	-23.20860000
H	-12.27240000	-8.25780000	-24.42980000
C	-11.37450000	-4.91470000	-24.78760000
H	-10.60840000	-4.32460000	-25.32930000
H	-11.28830000	-4.68020000	-23.70460000
H	-12.37190000	-4.57540000	-25.13360000
C	-3.22320000	-1.30320000	-34.07810000
C	-2.77020000	-0.13860000	-33.45940000
H	-3.09710000	-0.00580000	-32.42950000
C	-1.94770000	0.77100000	-34.13330000
C	-1.57850000	0.44670000	-35.45200000
H	-0.91670000	1.13980000	-35.96030000
C	-2.02900000	-0.70420000	-36.11960000
C	-2.87260000	-1.55910000	-35.40210000
H	-3.30100000	-2.45120000	-35.85470000
C	-1.44540000	2.08360000	-33.49360000
C	-1.99880000	2.30060000	-32.06580000
H	-1.64260000	3.26500000	-31.64230000
H	-3.10790000	2.33200000	-32.08500000
H	-1.66630000	1.49200000	-31.37930000
C	-1.89470000	3.28650000	-34.35300000
H	-1.42580000	3.27310000	-35.35850000
H	-1.60720000	4.24560000	-33.87010000
H	-2.99850000	3.27660000	-34.48330000
C	0.09480000	2.05940000	-33.40410000
H	0.47410000	2.97790000	-32.90570000
H	0.43330000	1.17800000	-32.81740000
H	0.56170000	2.00840000	-34.40980000
C	-1.64660000	-1.05880000	-37.57210000
C	-0.93750000	-2.43090000	-37.59600000
H	-0.61300000	-2.68940000	-38.62730000
H	-0.03970000	-2.41310000	-36.94080000
H	-1.60740000	-3.24380000	-37.24520000
C	-0.69270000	-0.02480000	-38.21640000
H	-0.43750000	-0.31940000	-39.25770000
H	-1.16830000	0.97890000	-38.26180000
H	0.25930000	0.04440000	-37.64670000
C	-2.92270000	-1.12550000	-38.43890000
H	-3.60790000	-1.92870000	-38.09630000
H	-2.66770000	-1.33650000	-39.50020000
H	-3.46940000	-0.15860000	-38.39620000
O	-11.54990000	1.76150000	-30.70760000
O	-12.18780000	1.24240000	-33.20650000
P	-12.40750000	2.02910000	-31.93660000
C	-12.78730000	4.48210000	-31.36290000
C	-14.14950000	4.69850000	-31.26940000
C	-14.58990000	5.62100000	-30.26520000
C	-13.63500000	6.32210000	-29.46220000
C	-14.09310000	7.31340000	-28.55870000
H	-13.37590000	7.89940000	-27.99140000
C	-15.43420000	7.53320000	-28.39530000
C	-16.39050000	6.78120000	-29.10170000
H	-17.44820000	6.95120000	-28.92260000
C	-15.96650000	5.85290000	-30.01550000
H	-16.70420000	5.27880000	-30.56760000
C	-12.24280000	6.01080000	-29.57630000
C	-11.84760000	5.09370000	-30.52230000
H	-10.80690000	4.78100000	-30.60100000
Cl	-15.98600000	8.76030000	-27.28100000
O	-12.29550000	3.59790000	-32.30690000
C	-14.91110000	2.54140000	-32.19760000
C	-15.06020000	3.91460000	-32.13870000
C	-16.07010000	4.49080000	-32.97230000
C	-16.94230000	3.65140000	-33.73600000
C	-16.72560000	2.23510000	-33.76340000
C	-15.70340000	1.71330000	-33.00270000
H	-15.45840000	0.65180000	-33.03800000
C	-18.01340000	4.25250000	-34.44430000
H	-18.73520000	3.62990000	-34.96410000
C	-18.14830000	5.61440000	-34.47500000
C	-17.23360000	6.45790000	-33.81920000
H	-17.33990000	7.53620000	-33.89690000
C	-16.22650000	5.89590000	-33.08040000
H	-15.52560000	6.54160000	-32.55930000
Cl	-19.47610000	6.33610000	-35.35250000
O	-13.94210000	1.91910000	-31.43050000
C	-11.22040000	6.59800000	-28.68490000
C	-10.06710000	7.15960000	-29.22820000

H	-10.01770000	7.15190000	-30.31560000
C	-9.07100000	7.70200000	-28.40880000
C	-9.29040000	7.66320000	-27.01860000
H	-8.53570000	8.12160000	-26.38840000
C	-10.41630000	7.06300000	-26.43110000
C	-11.37170000	6.53110000	-27.30220000
H	-12.26360000	6.02600000	-26.93420000
C	-7.77630000	8.32980000	-28.96750000
C	-7.65170000	8.17550000	-30.50310000
H	-7.67010000	7.10250000	-30.78690000
H	-6.69340000	8.60350000	-30.86970000
H	-8.47410000	8.70780000	-31.02820000
C	-6.54700000	7.64350000	-28.33160000
H	-6.47510000	7.84520000	-27.24310000
H	-5.60680000	8.01690000	-28.79130000
H	-6.60280000	6.54340000	-28.47830000
C	-7.75290000	9.83580000	-28.63640000
H	-8.64480000	10.33970000	-29.06800000
H	-6.84220000	10.31620000	-29.05560000
H	-7.75170000	10.01060000	-27.54000000
C	-10.63700000	6.94960000	-24.90860000
C	-11.95220000	7.66270000	-24.52490000
H	-11.92220000	8.72680000	-24.84500000
H	-12.11070000	7.63240000	-23.42500000
H	-12.83250000	7.18130000	-25.00050000
C	-9.49480000	7.58850000	-24.08370000
H	-8.52480000	7.08950000	-24.29790000
H	-9.69000000	7.48440000	-22.99410000
H	-9.40660000	8.67430000	-24.30440000
C	-10.72750000	5.45920000	-24.51260000
H	-11.59940000	4.96260000	-24.98690000
H	-10.84100000	5.34930000	-23.41230000
H	-9.80740000	4.91940000	-24.82540000
C	-17.53300000	1.31450000	-34.59390000
C	-18.03000000	0.13900000	-34.03100000
H	-17.80740000	0.00310000	-32.97420000
C	-18.76740000	-0.77660000	-34.79020000
C	-19.00520000	-0.44760000	-36.13770000
H	-19.60080000	-1.14640000	-36.71530000
C	-18.50730000	0.71500000	-36.74900000
C	-17.75320000	1.57610000	-35.94450000
H	-17.29450000	2.47750000	-36.34590000
C	-19.31440000	-2.10120000	-34.21400000
C	-18.91270000	-2.32050000	-32.73680000
H	-19.30250000	-3.29010000	-32.35690000
H	-17.80830000	-2.34430000	-32.64100000
H	-19.32160000	-1.51770000	-32.08570000
C	-18.75730000	-3.29140000	-35.02720000
H	-19.11340000	-3.27550000	-36.07790000
H	-19.08250000	-4.25830000	-34.58530000
H	-17.64600000	-3.26610000	-35.03550000
C	-20.85580000	-2.10110000	-34.28830000
H	-21.27130000	-3.02800000	-33.83660000
H	-21.26820000	-1.22820000	-33.73730000
H	-21.21460000	-2.05190000	-35.33740000
C	-18.74620000	1.07550000	-38.23040000
C	-19.46950000	2.43770000	-38.31720000
H	-19.69180000	2.69970000	-39.37440000
H	-20.42850000	2.40210000	-37.75630000
H	-18.85040000	3.25710000	-37.89520000
C	-19.61500000	0.03330000	-38.97440000
H	-19.76830000	0.33250000	-40.03410000
H	-19.12230000	-0.96320000	-38.97820000
H	-20.61830000	-0.05390000	-38.50400000
C	-17.39040000	1.16680000	-38.96350000
H	-16.75510000	1.97680000	-38.54830000
H	-17.54000000	1.38240000	-40.04370000
H	-16.83660000	0.20730000	-38.87180000
O	-9.23670000	1.70220000	-32.98250000
O	-8.79540000	0.89790000	-30.51430000
Rh	-10.47250000	0.03540000	-33.23390000
P	-8.46520000	1.81260000	-31.68780000
C	-7.93340000	4.28920000	-31.95210000
C	-6.55790000	4.41990000	-31.97360000
C	-6.01360000	5.39300000	-32.87070000
C	-6.88010000	6.26660000	-33.60050000
C	-6.30240000	7.30810000	-34.37020000
H	-6.94110000	8.03370000	-34.86450000
C	-4.94290000	7.40690000	-34.49480000
C	-4.08010000	6.48260000	-33.87860000
H	-3.00750000	6.55720000	-34.03380000
C	-4.61560000	5.50760000	-33.07960000
H	-3.95380000	4.80030000	-32.58860000
C	-8.29870000	6.07560000	-33.53850000
C	-8.79130000	5.07570000	-32.73090000
H	-9.85450000	4.84110000	-32.71340000
Cl	-4.24920000	8.69710000	-35.44780000
O	-8.52370000	3.33360000	-31.13950000
C	-5.97650000	2.16980000	-31.13960000
C	-5.74170000	3.53150000	-31.11360000
C	-4.75780000	4.00520000	-30.18800000
C	-4.01870000	3.07980000	-29.38600000
C	-4.35540000	1.68650000	-29.41300000
C	-5.32580000	1.26050000	-30.29320000
H	-5.63860000	0.21630000	-30.33340000
C	-2.94300000	3.56960000	-28.60150000
H	-2.31000000	2.87740000	-28.05560000
C	-2.68180000	4.91110000	-28.53910000
C	-3.47380000	5.84420000	-29.23070000
H	-3.27100000	6.90650000	-29.12930000
C	-4.48290000	5.38840000	-30.03660000
H	-5.08770000	6.10600000	-30.58200000
Cl	-1.34290000	5.49130000	-27.57770000
O	-6.89230000	1.64890000	-32.03500000
C	-9.25170000	6.88450000	-34.32960000
C	-10.38050000	7.41820000	-33.70780000
H	-10.46200000	7.21090000	-32.64210000
C	-11.31830000	8.17050000	-34.42420000
C	-11.06260000	8.37930000	-35.79230000
H	-11.77650000	8.98740000	-36.33740000
C	-9.95320000	7.83860000	-36.46280000
C	-9.06520000	7.07550000	-35.69700000
H	-8.20030000	6.58760000	-36.14200000
C	-12.58910000	8.76690000	-33.78020000
C	-12.73510000	8.39040000	-32.28700000
H	-12.78850000	7.28890000	-32.17370000
H	-13.66820000	8.81610000	-31.85770000
H	-11.88350000	8.78130000	-31.68920000
C	-13.84130000	8.23970000	-34.51600000
H	-13.87510000	8.58170000	-35.57090000
H	-14.76950000	8.60340000	-34.02440000
H	-13.85240000	7.12810000	-34.50970000
C	-12.54390000	10.30640000	-33.87590000
H	-11.62820000	10.69680000	-33.38130000
H	-13.42970000	10.75750000	-33.37820000
H	-12.54330000	10.65050000	-34.93120000
C	-9.67670000	8.04150000	-37.96740000
C	-8.30440000	8.72690000	-38.14960000
H	-8.28180000	9.69630000	-37.60620000
H	-8.10120000	8.92350000	-39.22460000
H	-7.47690000	8.09400000	-37.76560000
C	-10.73970000	8.92390000	-38.66440000
H	-10.50190000	9.04910000	-39.74330000
H	-11.74690000	8.45840000	-38.59770000

H	-10.77200000	9.93800000	-38.21040000
C	-9.66420000	6.67080000	-38.67810000
H	-8.84700000	6.02180000	-38.29970000
H	-9.51020000	6.79530000	-39.77200000
H	-10.63010000	6.14430000	-38.51860000
C	-3.69140000	0.70630000	-28.52570000
C	-3.21070000	-0.48940000	-29.05770000
H	-3.37030000	-0.61840000	-30.12690000
C	-2.54800000	-1.42570000	-28.25550000
C	-2.36780000	-1.08840000	-26.90370000
H	-1.81460000	-1.77990000	-26.27300000
C	-2.85220000	0.09550000	-26.32120000
C	-3.54570000	0.97150000	-27.16230000
H	-3.99760000	1.89870000	-26.81930000
C	-2.00190000	-2.76320000	-28.79490000
C	-2.34880000	-2.98910000	-30.28480000
H	-1.97930000	-3.97760000	-30.63490000
H	-3.44900000	-2.96930000	-30.43070000
H	-1.88110000	-2.21190000	-30.92730000
C	-2.61130000	-3.93150000	-27.99150000
H	-2.30220000	-3.90470000	-26.92640000
H	-2.28230000	-4.90910000	-28.40490000
H	-3.71960000	-3.88490000	-28.03440000
C	-0.46570000	-2.78840000	-28.65580000
H	-0.04840000	-3.72830000	-29.07820000
H	-0.01490000	-1.93000000	-29.19930000
H	-0.15190000	-2.73100000	-27.59250000
C	-2.56980000	0.39870000	-24.83510000
C	-1.04460000	0.46660000	-24.60510000
H	-0.81650000	0.73570000	-23.55090000
H	-0.55620000	-0.50860000	-24.81170000
H	-0.58800000	1.23320000	-25.26820000
C	-3.17230000	-0.71310000	-23.95270000
H	-2.99790000	-0.50050000	-22.87570000
H	-4.26710000	-0.78030000	-24.11920000
H	-2.72180000	-1.70300000	-24.17300000
C	-3.17670000	1.74580000	-24.37430000
H	-2.74850000	2.59220000	-24.95420000
H	-2.95650000	1.93090000	-23.30040000
H	-4.28200000	1.74250000	-24.49330000
O	-11.71650000	-1.66080000	-33.35440000
O	-12.43520000	-1.03710000	-30.90710000
P	-12.59230000	-1.90240000	-32.14890000
C	-12.99040000	-4.44060000	-32.43610000
C	-14.36130000	-4.61320000	-32.51640000
C	-14.82980000	-5.62690000	-33.41030000
C	-13.90930000	-6.52440000	-34.03840000
C	-14.42800000	-7.60310000	-34.79900000
H	-13.75490000	-8.34780000	-35.21250000
C	-15.77490000	-7.71340000	-35.01720000
C	-16.68330000	-6.76800000	-34.50640000
H	-17.74150000	-6.85370000	-34.73660000
C	-16.20930000	-5.75850000	-33.71180000
H	-16.90400000	-5.03430000	-33.29560000
C	-12.50070000	-6.31930000	-33.88010000
C	-12.07560000	-5.25930000	-33.11090000
H	-11.01610000	-5.01650000	-33.03210000
Cl	-16.39380000	-9.04860000	-35.96010000
O	-12.45440000	-3.44180000	-31.64300000
C	-15.04470000	-2.38680000	-31.70120000
C	-15.24420000	-3.75270000	-31.69290000
C	-16.23840000	-4.25660000	-30.79110000
C	-16.98150000	-3.35390000	-29.96600000
C	-16.64790000	-1.96030000	-29.94950000
C	-15.69270000	-1.50170000	-30.82930000
H	-15.38190000	-0.45670000	-30.83570000
C	-18.05730000	-3.86200000	-29.19560000

H	-18.68460000	-3.18240000	-28.62770000
C	-18.32600000	-5.20290000	-29.17460000
C	-17.54070000	-6.11590000	-29.89790000
H	-17.75190000	-7.17960000	-29.83520000
C	-16.52510000	-5.64080000	-30.68520000
H	-15.92470000	-6.34860000	-31.24750000
Cl	-19.66450000	-5.80740000	-28.22660000
O	-14.13760000	-1.84260000	-32.59930000
C	-11.47890000	-7.18190000	-34.51270000
C	-10.41350000	-7.64440000	-33.74000000
H	-10.44050000	-7.34490000	-32.69380000
C	-9.40320000	-8.43960000	-34.29080000
C	-9.52310000	-8.77400000	-35.65270000
H	-8.75460000	-9.41640000	-36.06930000
C	-10.56650000	-8.31280000	-36.47240000
C	-11.52940000	-7.49590000	-35.86880000
H	-12.34700000	-7.05950000	-36.43900000
C	-8.19440000	-8.94840000	-33.47510000
C	-8.19820000	-8.43380000	-32.01570000
H	-8.17300000	-7.32510000	-31.99790000
H	-7.30460000	-8.79710000	-31.46260000
H	-9.09700000	-8.79020000	-31.46760000
C	-6.88300000	-8.46550000	-34.13390000
H	-6.74240000	-8.90420000	-35.14330000
H	-6.00260000	-8.76110000	-33.52310000
H	-6.88650000	-7.35840000	-34.23280000
C	-8.21280000	-10.49070000	-33.43140000
H	-9.16740000	-10.85260000	-32.99160000
H	-7.37400000	-10.87660000	-32.81240000
H	-8.10830000	-10.92940000	-34.44560000
C	-10.69450000	-8.65500000	-37.97180000
C	-12.03980000	-9.37160000	-38.22220000
H	-12.11150000	-10.28780000	-37.59690000
H	-12.13670000	-9.66760000	-39.28930000
H	-12.90320000	-8.71700000	-37.97970000
C	-9.56540000	-9.58340000	-38.47940000
H	-8.57140000	-9.10040000	-38.36030000
H	-9.69760000	-9.81030000	-39.55980000
H	-9.57250000	-10.55100000	-37.93230000
C	-10.64320000	-7.35470000	-38.80300000
H	-11.49600000	-6.68460000	-38.56640000
H	-10.68930000	-7.58000000	-39.89060000
H	-9.69950000	-6.80360000	-38.59970000
C	-17.30440000	-1.00560000	-29.03130000
C	-17.76770000	0.21330000	-29.52600000
H	-17.61370000	0.36760000	-30.59230000
C	-18.39570000	1.14550000	-28.69270000
C	-18.56760000	0.78540000	-27.34310000
H	-19.08520000	1.49650000	-26.70870000
C	-18.10390000	-0.42480000	-26.80000000
C	-17.45070000	-1.30040000	-27.67570000
H	-17.03170000	-2.24700000	-27.33490000
C	-18.90810000	2.51240000	-29.19490000
C	-18.59000000	2.75690000	-30.68890000
H	-18.94670000	3.75860000	-31.01370000
H	-17.49450000	2.72010000	-30.85960000
H	-19.08740000	2.00060000	-31.33400000
C	-18.24150000	3.64740000	-28.38660000
H	-18.50650000	3.60140000	-27.31040000
H	-18.57150000	4.63970000	-28.76180000
H	-17.13550000	3.58720000	-28.47590000
C	-20.43960000	2.58610000	-29.02070000
H	-20.83550000	3.54420000	-29.42210000
H	-20.93030000	1.75000000	-29.56450000
H	-20.73280000	2.52640000	-27.95180000
C	-18.31560000	-0.83750000	-25.32780000
C	-19.19930000	-2.10250000	-25.27890000

H	-19.39020000	-2.40940000	-24.22770000
H	-20.17710000	-1.91020000	-25.77120000
H	-18.71370000	-2.95740000	-25.79590000
C	-19.00920000	0.25970000	-24.48550000
H	-19.12800000	-0.06890000	-23.43010000
H	-18.40550000	1.19320000	-24.48100000
H	-20.02570000	0.47930000	-24.87770000
C	-16.95310000	-1.14220000	-24.66910000
H	-16.49050000	-2.04980000	-25.10640000
H	-17.07210000	-1.33290000	-23.58050000
H	-16.25650000	-0.28600000	-24.80080000
C	-10.82720000	-0.38320000	-28.48100000
C	-11.70340000	-1.47170000	-28.02690000
C	-11.00290000	0.99980000	-27.98140000
H	-9.70250000	-0.66340000	-28.65590000
C	-11.69970000	-2.73060000	-28.65500000
C	-12.62050000	-1.26270000	-26.98350000
O	-10.14210000	1.75890000	-27.60510000
O	-12.31560000	1.31680000	-28.08970000
C	-8.84580000	-0.82140000	-27.61310000
C	-12.63250000	-3.70730000	-28.32650000
H	-10.98570000	-2.93010000	-29.45020000
C	-13.57320000	-2.21610000	-26.66270000
H	-12.62970000	-0.32330000	-26.43810000
C	-12.71610000	2.64770000	-27.89130000
C	-9.04600000	-0.81550000	-26.12520000
C	-8.39400000	-2.26070000	-27.60030000
H	-8.19720000	-0.05370000	-28.05130000
C	-13.58790000	-3.41370000	-27.36230000
H	-12.62170000	-4.67200000	-28.83450000
H	-14.29240000	-2.02020000	-25.87520000
H	-11.86900000	3.28090000	-27.59520000
H	-13.15960000	3.01990000	-28.82530000
C	-13.78260000	2.67480000	-26.80420000
H	-10.04060000	-1.19140000	-25.84310000
C	-8.02370000	-1.98200000	-26.12520000
H	-8.82300000	0.11170000	-25.59010000
H	-7.61270000	-2.56860000	-28.30360000
H	-9.24550000	-2.95800000	-27.65560000
Br	-14.99420000	-4.70220000	-26.94940000
Cl	-13.06330000	2.17580000	-25.23620000
Cl	-15.13030000	1.58820000	-27.21220000
Cl	-14.39440000	4.34920000	-26.66860000
C	-8.02120000	-3.05620000	-25.02710000
C	-6.55800000	-1.63280000	-25.79820000
C	-6.66560000	-2.51380000	-24.53460000
H	-8.84360000	-3.02130000	-24.30480000
H	-7.93570000	-4.06420000	-25.44580000
H	-6.32370000	-0.57420000	-25.63470000
H	-5.85660000	-2.03990000	-26.53460000
H	-6.74820000	-1.93130000	-23.61100000
N	-5.61040000	-3.47160000	-24.29940000
C	-5.17760000	-4.41780000	-25.23560000
C	-4.87150000	-3.53360000	-23.10600000
C	-4.03770000	-5.12380000	-24.59550000
O	-5.66420000	-4.59120000	-26.33260000
O	-5.05520000	-2.84080000	-22.13280000
C	-3.85540000	-4.59310000	-23.32280000
C	-3.22430000	-6.13010000	-25.08160000
C	-2.85340000	-5.04190000	-22.48400000
H	-3.38390000	-6.54420000	-26.07480000
C	-2.20630000	-6.59030000	-24.24400000
H	-2.72530000	-4.61430000	-21.49270000
C	-2.02330000	-6.05540000	-22.96760000
H	-1.54390000	-7.38030000	-24.58920000
H	-1.22030000	-6.43710000	-22.34190000

20a

C	1.78561000	-1.53102300	-1.40180200
C	2.65905800	-0.54978000	-0.59774700
C	0.68360500	-1.33868400	-0.34132100
H	1.48851100	-1.10047200	-2.36548600
H	3.28600600	0.16648600	-1.14436900
H	3.28326100	-1.07812500	0.13505800
H	-0.36117300	-1.28537900	-0.66924000
H	0.76437600	-2.08447900	0.46017600
C	1.36604500	-0.02628300	0.05630800
C	1.39434300	0.61741200	1.44907500
C	0.85127600	1.31127800	-0.51324300
C	1.44266100	1.98080700	0.73825600
H	2.22077600	0.35912300	2.12118300
H	0.43648400	0.48138700	1.96404400
H	1.21912600	1.63810200	-1.49350300
H	-0.24393400	1.34874800	-0.49228200
H	2.47691400	2.30006300	0.56506500
N	0.77580600	3.12402900	1.32427700
C	1.43047800	4.34240000	1.57158700
C	-0.56800000	3.16611800	1.71491800
C	0.40739000	5.23589300	2.17228100
O	2.59123600	4.57677900	1.33044300
O	-1.36780100	2.26208800	1.61960300
C	-0.78739200	4.53283100	2.25829500
C	0.51232000	6.54434400	2.60381100
C	-1.93064500	5.10693400	2.78003300
H	1.45504800	7.08045500	2.52925800
C	-0.63642700	7.13532000	3.13285000
H	-2.85797200	4.54285700	2.84041700
C	-1.83717800	6.42871800	3.21948700
H	-0.59880200	8.16337800	3.48435800
H	-2.71291200	6.91937800	3.63693200
H	2.16942800	-2.54102400	-1.57529300

20b

C	1.78561000	-1.53102300	1.40180200
C	2.65905800	-0.54978000	0.59774700
C	0.68360500	-1.33868400	0.34132100
H	1.48851100	-1.10047200	2.36548600
H	3.28600600	0.16648600	1.14436900
H	3.28326100	-1.07812500	-0.13505800
H	-0.36117300	-1.28537900	0.66924000
H	0.76437600	-2.08447900	-0.46017600
C	1.36604500	-0.02628300	-0.05630800
C	1.39434300	0.61741200	-1.44907500
C	0.85127600	1.31127800	0.51324300
C	1.44266100	1.98080700	-0.73825600
H	2.22077600	0.35912300	-2.12118300
H	0.43648400	0.48138700	-1.96404400
H	1.21912600	1.63810200	1.49350300
H	-0.24393400	1.34874800	0.49228200
H	2.47691400	2.30006300	-0.56506500
N	0.77580600	3.12402900	-1.32427700
C	1.43047800	4.34240000	-1.57158700
C	-0.56800000	3.16611800	-1.71491800
C	0.40739000	5.23589300	-2.17228100
O	2.59123600	4.57677900	-1.33044300
O	-1.36780100	2.26208800	-1.61960300
C	-0.78739200	4.53283100	-2.25829500
C	0.51232000	6.54434400	-2.60381100
C	-1.93064500	5.10693400	-2.78003300
H	1.45504800	7.08045500	-2.52925800
C	-0.63642700	7.13532000	-3.13285000
H	-2.85797200	4.54285700	-2.84041700
C	-1.83717800	6.42871800	-3.21948700
H	-0.59880200	8.16337800	-3.48435800

H	-2.71291200	6.91937800	-3.63693200
H	2.16942800	-2.54102400	1.57529300

20c

C	1.23929000	-1.54534100	-1.65525900
C	2.38623000	-0.73673900	-1.02081800
C	0.44750600	-1.35087100	-0.34830000
H	0.77959700	-0.99453600	-2.48435400
H	2.94163300	-0.03430900	-1.65270300
H	3.09417500	-1.39279000	-0.49635100
H	-0.63275800	-1.16764500	-0.40729900
H	0.62747300	-2.17495100	0.35479600
C	1.35300700	-0.15430800	-0.03563300
C	1.76129800	0.33864900	1.35786900
C	0.86439200	1.27265700	-0.35532800
C	1.68065400	1.79281900	0.84463500
H	2.72867800	0.00909900	1.75470500
H	0.98761200	0.14651800	2.11101600
H	1.07231400	1.68505900	-1.34443500
H	-0.20360300	1.38259400	-0.12805100
H	1.44774600	-2.56665000	-1.98863500
H	1.17331100	2.50227100	1.50815700
N	2.99659300	2.35927800	0.55167200
C	3.81100100	2.73073100	1.63762600
C	3.60470700	2.67091700	-0.67354700
C	5.04947100	3.30109300	1.05517400
O	3.50915900	2.59883200	2.80083400
O	3.16386300	2.49592200	-1.78745300
C	4.92504400	3.26411400	-0.32579900
C	6.17836200	3.80790000	1.67029600
C	5.92358300	3.73289800	-1.15745000
H	6.25921000	3.83006700	2.75411300
C	7.19543700	4.28375300	0.84107500
H	5.80878900	3.69788200	-2.23785500
C	7.07006600	4.24691500	-0.54889800
H	8.10190200	4.69113500	1.28183300
H	7.88125600	4.62661400	-1.16514600

20d

C	1.73156000	-1.54758900	-1.37216400
C	2.63210000	-0.58627300	-0.57428200
C	0.62401300	-1.29262900	-0.33106300
H	1.46426700	-1.12387600	-2.34740900
H	3.29230100	0.09446300	-1.12690800
H	3.22645400	-1.12372800	0.17639600
H	-0.41306700	-1.20461500	-0.67691400
H	0.66340100	-2.02626100	0.48462900
C	1.35209600	-0.00081200	0.05339500
C	1.38657000	0.67067200	1.43082300
C	0.90036900	1.34717500	-0.53859400
C	1.51836600	2.01509200	0.69876700
H	2.16947000	0.38980800	2.14201800
H	0.41105600	0.60219400	1.92911000
H	1.26303100	1.64998800	-1.52646000
H	-0.19334500	1.43989700	-0.51411000
H	2.57568600	2.25779200	0.52051900
N	0.84147800	3.16910200	1.25873600
C	0.29251200	4.20198600	0.48470400
C	0.77261300	3.45067400	2.63119900
C	-0.24562900	5.18983200	1.45688100
O	0.28349300	4.25435800	-0.72323600
O	1.23152900	2.77121400	3.51975300
C	0.04101400	4.74063900	2.73944100
C	-0.91500800	6.37846800	1.23715600
C	-0.32905600	5.45997600	3.85957200
H	-1.12868400	6.71737700	0.22657200
C	-1.29677100	7.11279700	2.36134700
H	-0.09419400	5.09572600	4.85651900
C	-1.00880100	6.66122700	3.65045800
H	-1.82605600	8.05389300	2.23425600
H	-1.31910600	7.25876800	4.50403900
H	2.07738200	-2.57495900	-1.52159100

20TS

C	1.98931400	-1.88709300	-0.91318500
C	2.65700900	-0.52649800	-0.61686700
C	0.66421200	-1.36182600	-0.32010300
H	1.94336500	-2.15094800	-1.97437600
H	3.00242300	0.02119600	-1.50258600
H	3.48691800	-0.56532400	0.09972700
H	-0.17185500	-1.30522700	-1.02734500
H	0.31215300	-1.89096600	0.57344000
C	1.32610100	-0.00797100	-0.02453700
C	1.36764200	0.62013600	1.38049800
C	0.78219700	1.33075500	-0.55525400
C	1.40135000	1.99062600	0.68656400
H	2.20016400	0.35679900	2.04335000
H	0.41346700	0.47261500	1.89972600
H	1.11568000	1.67298400	-1.54193000
H	-0.31242500	1.35393500	-0.50028700
H	2.43252200	2.30830100	0.49143200
N	0.75232600	3.12925800	1.30113400
C	1.41677400	4.34274600	1.54606100
C	-0.58246500	3.17220800	1.72163400
C	0.41085800	5.23339000	2.17901200
O	2.57262700	4.57581500	1.28114200
O	-1.38794900	2.27243800	1.63434100
C	-0.78440000	4.53383400	2.28448000
C	0.53084100	6.53665900	2.62210300
C	-1.91316700	5.10660600	2.83832200
H	1.47381400	7.06992900	2.53186400
C	-0.60314200	7.12619500	3.18356900
H	-2.84115600	4.54542100	2.91375800
C	-1.80439500	6.42319000	3.28989100
H	-0.55341200	8.15018700	3.54529300
H	-2.66843400	6.91249600	3.73255700
H	2.42623500	-2.73561700	-0.37747500

10. References

(1) Lu, Y.-C.; West, J. G. Chemoselective Decarboxylative Protonation Enabled by Cooperative Earth-Abundant Element Catalysis. *Angew. Chem., Int. Ed.* **2023**, *62*, e202213055.
(2) Frank, W. B-LACTAMASE INHIBITOR AND APPLICATION THEREOF. EP3281942A1, 2015.
(3) BERNHARD, F.; THOMAS, R.; ANDREAS, R.; VLADIMIRAS, O. COMPOUNDS THAT MEDIATE PROTEIN DEGRADATION AND METHODS OF USE THEREOF. 2023.
(4) Frisch, M. J.; Trucks, G. W.; Schlegel, H. B.; Scuseria, G. E.; Robb, M. A.; Cheeseman, J. R.; Scalmani, G.; Barone, V.; Petersson, G. A.; Nakatsuji, H.; et al. Gaussian 16 Rev. C.01. **2016**.
(5) Humphrey, W.; Dalke, A.; Schulten, K. VMD: Visual molecular dynamics. *J. Mol. Graph.* **1996**, *14*, 33-38.
(6) Dapprich, S.; Komáromi, I.; Byun, K. S.; Morokuma, K.; Frisch, M. J. A new ONIOM implementation in Gaussian98. Part I. The calculation of energies, gradients, vibrational frequencies and electric field derivatives1Dedicated to Professor Keiji Morokuma in celebration of his 65th birthday.1. *J. Mol. Struct.: THEOCHEM* **1999**, *461-462*, 1-21.
(7) Vreven, T.; Byun, K. S.; Komáromi, I.; Dapprich, S.; Montgomery, J. A., Jr.; Morokuma, K.; Frisch, M. J. Combining Quantum Mechanics Methods with Molecular Mechanics Methods in ONIOM. *J. Chem. Theory Comput.* **2006**, *2*, 815-826.
(8) Zhao, Y.; Truhlar, D. G. The M06 suite of density functionals for main group thermochemistry, thermochemical kinetics, noncovalent interactions, excited states, and transition elements: two new functionals and systematic testing of four M06-class functionals and 12 other functionals. *Theor. Chem. Acc.* **2008**, *120*, 215-241.
(9) Hay, P. J.; Wadt, W. R. Ab initio effective core potentials for molecular calculations. Potentials for K to Au including the outermost core orbitals. *J. Chem. Phys.* **1985**, *82*, 299-310.
(10) Roy, L. E.; Hay, P. J.; Martin, R. L. Revised Basis Sets for the LANL Effective Core Potentials. *J. Chem. Theory Comput.* **2008**, *4*, 1029-1031.
(11) Hariharan, P. C.; Pople, J. A. The influence of polarization functions on molecular orbital hydrogenation energies. *Theoretica chimica acta* **1973**, *28*, 213-222.
(12) Hehre, W. J.; Ditchfield, R.; Pople, J. A. Self—Consistent Molecular Orbital Methods. XII. Further Extensions of Gaussian—Type Basis Sets for Use in Molecular Orbital Studies of Organic Molecules. *J. Chem. Phys.* **1972**, *56*, 2257-2261.
(13) Rappe, A. K.; Casewit, C. J.; Colwell, K. S.; Goddard, W. A., III; Skiff, W. M. UFF, a full periodic table force field for molecular mechanics and molecular dynamics simulations. *J. Am. Chem. Soc.* **1992**, *114*, 10024-10035.
(14) Mitoraj, M. P.; Michalak, A.; Ziegler, T. A Combined Charge and Energy Decomposition Scheme for Bond Analysis. *J. Chem. Theory Comput.* **2009**, *5*, 962-975.
(15) Lu, T.; Chen, Q. Independent gradient model based on Hirshfeld partition: A new method for visual study of interactions in chemical systems. *J. Comput. Chem.* **2022**, *43*, 539-555.
(16) Breneman, C. M.; Wiberg, K. B. Determining atom-centered monopoles from molecular electrostatic potentials. The need for high sampling density in formamide conformational analysis. *J. Comput. Chem.* **1990**, *11*, 361-373.
(17) Lu, T.; Chen, F. Multiwfn: A multifunctional wavefunction analyzer. *J. Comput. Chem.* **2012**, *33*, 580-592.
(18) Lu, T.; Chen, Q. Simple, Efficient, and Universal Energy Decomposition Analysis Method Based on Dispersion-Corrected Density Functional Theory. *The Journal of Physical Chemistry A* **2023**, *127*, 7023-7035.
(19) Bickelhaupt, F. M.; Houk, K. N. Analyzing Reaction Rates with the Distortion/Interaction-Activation Strain Model. *Angew. Chem., Int. Ed.* **2017**, *56*, 10070-10086.
(20) Brunard, E.; Boquet, V.; Saget, T.; Sosa Carrizo, E. D.; Sircoglou, M.; Dauban, P. Catalyst-Controlled Intermolecular Homobenzylic C(sp3)–H Amination for the Synthesis of β-Arylethylamines. *J. Am. Chem. Soc.* **2024**, *146*, 5843-5854.
(21) Berry, J. F. The role of three-center/four-electron bonds in superelectrophilic dirhodium carbene and nitrene catalytic intermediates. *Dalton Trans.* **2012**, *41*, 700-713, 10.1039/C1DT11434D.
